# Supplementary material for: Transcriptional Profiling and Molecular Characterization of the yccT Mutant Link: A Novel STY1099 Protein with the Peroxide Stress Response and Cell Division of Salmonella enterica Serovar Enteritidis
Source: Biology (Basel). 2019 Nov 13;8(4):86. doi: 10.3390/biology8040086 (PMC6955953; doi:10.3390/biology8040086)
Supplement: Supplementary file 1 [file biology-08-00086-s001.zip › supplementary files/Supplementary files description.docx]

**Supplementary files description**

Figure S1. mRNA expression levels of *yccT*, *gutM*, *napD*, *oxyR*, and *sodA* in the wild type *S*. Enteritidis strain during its exponential growth exposed to 4 mM of parquet. Values on the *y*-axis are relative expression levels (fold change) normalized to wild type during the oxidative treatment. The data correspond to the mean value of three biological replications. *Error bars* correspond to the standard deviation.

Table S1. Primers and their efficiency used for the gene expression assay.

Table S2. Expression data of the reference gene *rtcR* in the wild type and its *yccT* mutant.

Table S3. List of genes exhibiting statistically significant changes in expression between the *yccT* mutant treated and no treated with H_2_O_2_.

Table S4. List of the genes exhibiting statistically significant changes in expression between the wild type and *yccT* mutant treated with H_2_O_2_.

Table S5. List of the genes exhibiting statistically significant changes in expression between the *yccT* mutant and wild type during exponential growth with no H_2_O_2_ treatment.
