# Supplementary material for: Transcriptional Profiling and Molecular Characterization of the yccT Mutant Link: A Novel STY1099 Protein with the Peroxide Stress Response and Cell Division of Salmonella enterica Serovar Enteritidis
Source: Biology (Basel). 2019 Nov 13;8(4):86. doi: 10.3390/biology8040086 (PMC6955953; doi:10.3390/biology8040086)
Supplement: Supplementary file 1 [file biology-08-00086-s001.zip › supplementary files/Table S1.docx]

| **Gene** | **Forward primer sequence**  **(5’ – 3’)** | **Reverse primer sequence**  **(5’ – 3’)** | **Primer efficiency**  **(%)** |
| --- | --- | --- | --- |
| *pduA* | CAAGAAGCACTAGGAATGGTAGAA | TAGCCCACTAACATCACATTGG | 100.7 |
| *ydeZ* | TTCTGGCTTTCGGTGTGATTA | TAGCGGCAAAGCGACAATA | 98.8 |
| *yneC* | CTCAATTAAAGAGCCGGGTAACT | TAGTGTGGCGTTGTCTTGTG | 98.4 |
| *ego* | CCTCCGATCTGGAAGAGATAGA | GTTAATTTCCTCGCCGCATAAC | 101.7 |
| *ydeV* | GCCCATCTTCTCTGACGTTATG | CGCGAAATAGGGTCGCTTTA | 97.8 |
| *rplV* | GTCGCAGGCTCTGGATATT | CATCGTTGTGTTCAGCGTTAG | 105.7 |
| *rplP* | GGTAAGATCTGGATCCGTGTATTC | CCCAATACTCCACGTTACCTTTA | 106.9 |

1 of 1
