# Supplementary material for: Transcriptional Profiling and Molecular Characterization of the yccT Mutant Link: A Novel STY1099 Protein with the Peroxide Stress Response and Cell Division of Salmonella enterica Serovar Enteritidis
Source: Biology (Basel). 2019 Nov 13;8(4):86. doi: 10.3390/biology8040086 (PMC6955953; doi:10.3390/biology8040086)
Supplement: Supplementary file 1 [file biology-08-00086-s001.zip › supplementary files/Table S2.docx]

| **Strain name** | **Expression date**  **Ct (dRn)** | **Statistical differences**  ***P* value** |
| --- | --- | --- |
| Wild type | 25.44 | 0.3633 |
| Wild type | 25.89 |  |
| Wild type | 25.78 |  |
| yccT mutant | 26.58 |  |
| yccT mutant | 26.27 |  |
| yccT mutant | 26.6 |  |
