# Supplementary material for: Transcriptional Profiling and Molecular Characterization of the yccT Mutant Link: A Novel STY1099 Protein with the Peroxide Stress Response and Cell Division of Salmonella enterica Serovar Enteritidis
Source: Biology (Basel). 2019 Nov 13;8(4):86. doi: 10.3390/biology8040086 (PMC6955953; doi:10.3390/biology8040086)
Supplement: Supplementary file 1 [file biology-08-00086-s001.zip › supplementary files/Table S3.pdf]

Feature ID	Experiment - Range (original values)	Experiment - IQR (original values)	Experiment - Difference (original values)	Experiment - Fold Change (original values)	EDGE test: yccT H202 vs yccT NT , tagwise dispersions - P-value	EDGE test: yccT H202 vs yccT NT , tagwise dispersions - Fold change	EDGE test: yccT H202 vs yccT NT , tagwise dispersions - Weighted difference	EDGE test: yccT H202 vs yccT NT , tagwise dispersions - Bonferroni	EDGE test: yccT H202 vs yccT NT , tagwise dispersions - FDR p-value correction	yccT NT - yccT.1.S28 - Expression values	yccT NT - yccT.2.S29 - Expression values	yccT NT - yccT.3.S30 - Expression values	yccT NT - Means	yccT H202 - yccT.1.H2O2 - Expression values	yccT H202 - yccT.2.H2O2 - Expression values	yccT H202 - yccT.3.H2O2 - Expression values	yccT H202 - Means
aas	763	557	-658.333	-5.54023	1.81E-09	-2.35075	-3.6E-05	8E-06	8.71E-09	877	712	821	803.3333	114	155	166	145
abrB	841	315	607.6667	3.023307	6.14E-26	7.132753	0.000143	2.72E-22	1.06E-24	306	253	342	300.3333	1009	621	1094	908
accB	2918	2554	-2674	-12.9553	0	-5.46719	-0.00018	0	0	3106	2748	2839	2897.667	194	289	188	223.6667
aceE	13608	7206	9890.333	2.717967	1.56E-19	6.414761	0.002428	6.92E-16	1.86E-18	6268	5930	5073	5757	13136	18681	15125	15647.33
aceF	7609	2097	659	1.075429	6.75E-05	2.542948	0.001051	0.299219	0.000175	9241	9235	7734	8736.667	6720	14329	7138	9395.667
ackA	10009	8612	-9029.67	-7.15799	6.28E-08	-3.00996	-0.00055	0.000278	2.48E-07	11127	10122	10239	10496	1510	1771	1118	1466.333
acnA	42989	24589	30639.33	9.161783	2.03E-37	21.73779	0.006048	9.01E-34	5.82E-36	4217	3339	3706	3754	28295	46328	28557	34393.33
acnB	2798	599	155	1.010329	3.19E-06	2.390995	0.001623	0.014123	9.98E-06	16239	13441	15338	15006	14754	15376	15353	15161
acrE	38	3	-3.66667	-1.04661	5.21E-07	2.241262	7.99E-06	0.002307	1.82E-06	79	92	76	82.33333	78	60	98	78.66667
acrF	197	45	-8.66667	-1.02038	1.66E-08	2.296144	4.39E-05	7.35E-05	7.03E-08	423	447	432	434	387	346	543	425.3333
acrR	906	746	-781.333	-5.31676	2.43E-05	-2.27682	-4.2E-05	0.107545	6.72E-05	972	909	1006	962.3333	163	100	280	181
acs	362	106	-128.333	-1.16453	9.71E-06	2.020535	7.24E-05	0.043009	2.86E-05	810	888	1027	908.3333	665	893	782	780
ada	498	406	-442.333	-4.85756	3.2E-07	-2.06483	-2.2E-05	0.001419	1.16E-06	585	530	556	557	87	124	133	114.6667
adhE	19802	4782	10555.33	1.707842	7.4E-11	4.054309	0.003543	3.28E-07	4.11E-10	15768	13659	15309	14912	22850	33461	20091	25467.33
adiY	671	530	-591	-7.92578	2.04E-16	-3.36249	-3.7E-05	9.02E-13	1.95E-15	662	748	619	676.3333	90	77	89	85.33333
adk	1913	1766	-1833	-6.69255	6.9E-12	-2.84063	-0.00011	3.06E-08	4.31E-11	2197	2103	2165	2155	284	337	345	322
aegA	4152	3419	-3854.33	-15.9393	0	-6.72481	-0.00027	0	0	4401	3680	4256	4112.333	264	261	249	258
aes	1135	802	970.3333	8.742021	1.31E-81	20.56719	0.000191	5.79E-78	8.64E-80	131	135	110	125.3333	933	1245	1109	1095.667
ahpC	459247	115702	239173.3	16.47213	1.63E-25	39.17007	0.045925	7.23E-22	2.79E-24	16551	14915	14909	15458.33	159122	474156	130617	254631.7
ahpF	32160	25681	29865	18.60562	1.96E-77	44.00382	0.005676	8.67E-74	1.19E-75	1813	1584	1692	1696.333	33744	27373	33567	31561.33
aidB	4649	4067	-4254.33	-12.6027	0	-5.3668	-0.00029	0	0	4902	4532	4429	4621	362	253	485	366.6667
aldB	1622	1247	-1386	-4.84288	5.81E-06	-2.05198	-7E-05	0.025724	1.75E-05	1902	1706	1632	1746.667	385	280	417	360.6667
alkB	233	181	-199.667	-4.79114	2.16E-06	-2.03356	-1E-05	0.009559	6.93E-06	272	238	247	252.3333	39	57	62	52.66667
allA	287	229	-248.667	-6.56716	1.23E-10	-2.78923	-1.5E-05	5.43E-07	6.7E-10	320	290	270	293.3333	41	33	60	44.66667
allB	24	8	0.333333	1.001563	2.75E-10	2.364483	2.27E-05	1.22E-06	1.44E-09	218	215	207	213.3333	205	229	207	213.6667
allD	244	177	-196.333	-6.89	1.06E-10	-2.92522	-1.2E-05	4.7E-07	5.8E-10	210	208	271	229.6667	31	27	42	33.33333
allP	20	11	14.33333	1.754386	4.88E-13	4.109502	4.64E-06	2.16E-09	3.47E-12	21	19	17	19	30	33	37	33.33333
amiA	284	42	-53.3333	-1.07515	3.94E-07	2.189053	7.07E-05	0.001744	1.4E-06	804	740	745	763	703	571	855	709.6667
amiC	448	390	-421.333	-5.07742	3.59E-08	-2.14576	-2.2E-05	0.000159	1.47E-07	539	491	544	524.6667	101	113	96	103.3333
ampG	546	451	-498.333	-6.22727	1.45E-11	-2.64105	-2.9E-05	6.45E-08	8.82E-11	620	552	609	593.6667	74	111	101	95.33333

Feature ID	Experiment - Range (original values)	Experiment - IQR (original values)	Experiment - Difference (original values)	Experiment - Fold Change (original values)	EDGE test: yccT H202 vs yccT NT , tagwise dispersions - P-value	EDGE test: yccT H202 vs yccT NT , tagwise dispersions - Fold change	EDGE test: yccT H202 vs yccT NT , tagwise dispersions - Weighted difference	EDGE test: yccT H202 vs yccT NT , tagwise dispersions - Bonferroni	EDGE test: yccT H202 vs yccT NT , tagwise dispersions - FDR p-value correction	yccT NT - yccT.1.S28 - Expression values	yccT NT - yccT.2.S29 - Expression values	yccT NT - yccT.3.S30 - Expression values	yccT NT - Means	yccT H202 - yccT.1.H2O2 - Expression values	yccT H202 - yccT.2.H2O2 - Expression values	yccT H202 - yccT.3.H2O2 - Expression values	yccT H202 - Means
ampH	667	614	-621	-5.41469	1.09E-09	-2.30228	-3.4E-05	4.83E-06	5.4E-09	789	750	746	761.6667	122	132	168	140.6667
ansB	31747	24703	-27315	-5.19542	0.003061	-2.20199	-0.00144	1	0.005955	34442	34140	32895	33825.67	8645	2695	8192	6510.667
aphA	2517	2323	-2399.67	-7.78511	7.82E-14	-3.30345	-0.00015	3.46E-10	5.93E-13	2839	2752	2669	2753.333	346	322	393	353.6667
araB	48	8	5.666667	1.046448	3.95E-09	2.461374	1.39E-05	1.75E-05	1.82E-08	115	134	117	122	106	154	123	127.6667
araD	42	2	-17.3333	-1.16613	2.01E-06	2.017749	9.68E-06	0.008924	6.49E-06	111	133	121	121.6667	110	91	112	104.3333
arcC	36	16	-3	-1.05172	1.23E-05	2.24518	5.92E-06	0.054503	3.57E-05	66	50	67	61	43	79	52	58
argC	465	353	-410.333	-6.91827	1.1E-13	-2.9279	-2.5E-05	4.87E-10	8.21E-13	529	487	423	479.6667	64	74	70	69.33333
argI	466	318	-375	-4.83959	1.63E-06	-2.05586	-1.9E-05	0.007239	5.35E-06	550	465	403	472.6667	84	85	124	97.66667
argS	4184	3462	-3836	-10.5661	0	-4.48162	-0.00026	0	0	4311	3881	4519	4237	335	449	419	401
aroA	1437	1137	-1306	-6.71137	1.83E-12	-2.84049	-7.7E-05	8.13E-09	1.23E-11	1634	1369	1601	1534.667	197	257	232	228.6667
aroE	2066	1780	-1859.33	-5.42347	6.32E-07	-2.31075	-0.0001	0.0028	2.19E-06	2401	2157	2281	2279.667	377	335	549	420.3333
aroG	4301	3576	-3917.33	-9.46076	4E-15	-4.01656	-0.00026	1.77E-11	3.35E-14	4713	4012	4416	4380.333	412	436	541	463
aroH	1199	1050	-1104.33	-6.69244	3.4E-14	-2.83636	-6.5E-05	1.51E-10	2.66E-13	1378	1244	1273	1298.333	179	194	209	194
artI	1981	1674	-1810.67	-5.63086	2.01E-08	-2.38121	-9.9E-05	8.9E-05	8.49E-08	2359	2177	2069	2201.667	400	378	395	391
artJ	1000	652	-842.667	-5.94716	2.29E-10	-2.51401	-4.7E-05	1.01E-06	1.21E-09	1164	1050	825	1013	174	164	173	170.3333
artQ	689	577	-627.333	-5.43868	4.42E-10	-2.29933	-3.4E-05	1.96E-06	2.27E-09	826	718	762	768.6667	137	146	141	141.3333
asd	4301	3521	-3938.33	-5.111	2.14E-05	-2.15815	-0.0002	0.094643	5.97E-05	5150	4498	5041	4896.333	1048	849	977	958
asnA	38561	3762	25826	62.15075	4.74E-25	146.2481	0.004773	2.1E-21	7.86E-24	446	377	444	422.3333	35601	4206	38938	26248.33
asnB	12878	635	8621.667	8.222843	1.57E-13	19.34364	0.001707	6.94E-10	1.16E-12	1214	1185	1182	1193.667	13566	1820	14060	9815.333
asnC	242	37	135.3333	1.869379	2.54E-18	4.402744	4.12E-05	1.13E-14	2.8E-17	174	132	161	155.6667	301	198	374	291
aspA	217746	181737	-194982	-4.96853	0.000142	-2.1004	-0.00996	0.627977	0.00035	259595	239617	233130	244114	54154	41849	51393	49132
asrA	240	177	-197	-9.20833	3.11E-16	-3.87999	-1.3E-05	1.38E-12	2.92E-15	259	202	202	221	28	19	25	24
astC	47	15	-1.66667	-1.01931	3.5E-07	2.297474	8.93E-06	0.001553	1.26E-06	78	93	93	88	82	65	112	86.33333
atpB	4234	3544	-3819.67	-9.66138	1.33E-14	-4.0935	-0.00025	5.9E-11	1.08E-13	4583	4007	4192	4260.667	463	349	511	441
atpC	3968	1360	2729.667	1.82935	8.5E-13	4.358918	0.00086	3.76E-09	5.92E-12	3472	3087	3315	3291.333	7055	6333	4675	6021
atpD	4446	1712	481.6667	1.047829	1.04E-05	2.500626	0.001175	0.046052	3.05E-05	10881	9169	10162	10070.67	12172	11759	7726	10552.33
atpE	3325	2743	-3023.33	-5.53047	5.93E-06	-2.33272	-0.00016	0.026282	1.79E-05	3887	3330	3855	3690.667	587	853	562	667.3333
atpI	1619	1444	-1501	-9.05546	1.48E-13	-3.87079	-9.8E-05	6.57E-10	1.1E-12	1741	1708	1613	1687.333	169	122	268	186.3333
avrA	726	450	545	3.252066	1.25E-34	7.676183	0.000126	5.54E-31	3.2E-33	234	231	261	242	684	957	720	787
avtA	720	614	-654	-7.11215	8.59E-15	-3.00377	-4E-05	3.8E-11	7.02E-14	818	714	751	761	100	123	98	107

Feature ID	Experiment - Range (original values)	Experiment - IQR (original values)	Experiment - Difference (original values)	Experiment - Fold Change (original values)	EDGE test: yccT H202 vs yccT NT , tagwise dispersions - P-value	EDGE test: yccT H202 vs yccT NT , tagwise dispersions - Fold change	EDGE test: yccT H202 vs yccT NT , tagwise dispersions - Weighted difference	EDGE test: yccT H202 vs yccT NT , tagwise dispersions - Bonferroni	EDGE test: yccT H202 vs yccT NT , tagwise dispersions - FDR p-value correction	yccT NT - yccT.1.S28 - Expression values	yccT NT - yccT.2.S29 - Expression values	yccT NT - yccT.3.S30 - Expression values	yccT NT - Means	yccT H202 - yccT.1.H2O2 - Expression values	yccT H202 - yccT.2.H2O2 - Expression values	yccT H202 - yccT.3.H2O2 - Expression values	yccT H202 - Means
b2145	247	205	-221	-12.05	7.99E-22	-5.10011	-1.5E-05	3.54E-18	1.08E-20	262	223	238	241	15	18	27	20
barA	2062	1902	-1953	-6.83566	1.27E-11	-2.90769	-0.00012	5.63E-08	7.73E-11	2360	2206	2297	2287.667	298	304	402	334.6667
basS	697	270	-46.6667	-1.04584	3.15E-05	2.267771	0.000105	0.139572	8.6E-05	1142	979	1073	1064.667	803	1474	777	1018
bax	2665	2354	-2420	-5.17481	7.71E-05	-2.20457	-0.00013	0.341616	0.000198	3021	3009	2969	2999.667	615	356	768	579.6667
bcfA	66	13	-8	-1.08362	2.81E-05	2.191103	9.63E-06	0.124473	7.72E-05	115	104	92	103.6667	91	131	65	95.66667
bcp	3562	3184	-3307	-6.17797	4.6E-08	-2.61642	-0.00019	0.000204	1.86E-07	4127	3905	3805	3945.667	565	730	621	638.6667
bcsE	297	68	124.6667	1.091937	1.83E-08	2.585215	0.000167	8.11E-05	7.75E-08	1460	1215	1393	1356	1461	1512	1469	1480.667
bcsF	7	5	-1	-1.01852	6.93E-08	2.316239	5.66E-06	0.000307	2.71E-07	58	56	51	55	56	55	51	54
bfd	1162	579	842.3333	25.53398	9.73E-87	59.46251	0.000157	4.31E-83	7.31E-85	30	33	40	34.33333	826	612	1192	876.6667
bfr	1886	814	1335.667	3.612125	1.47E-26	8.487329	0.000298	6.52E-23	2.66E-25	546	496	492	511.3333	1853	1310	2378	1847
bigA	168	38	-25	-1.05474	1.3E-08	2.22973	4.61E-05	5.74E-05	5.59E-08	519	444	482	481.6667	367	468	535	456.6667
bioD	26	13	17.33333	2.268293	1E-14	5.315559	4.65E-06	4.45E-11	8.19E-14	16	10	15	13.66667	36	28	29	31
bioF	11	1	3.666667	1.261905	6.42E-06	2.959237	2.16E-06	0.028442	1.93E-05	17	9	16	14	16	20	17	17.66667
blc	1122	988	-1009.67	-8.55362	1.24E-14	-3.65654	-6.5E-05	5.5E-11	1.01E-13	1222	1095	1113	1143.333	100	107	194	133.6667
btuB	1864	1613	-1712	-5.24463	1.6E-06	-2.21349	-9E-05	0.007108	5.27E-06	2139	2016	2191	2115.333	480	327	403	403.3333
btuC	287	247	-264.333	-8.85149	9.48E-18	-3.75102	-1.7E-05	4.2E-14	9.86E-17	316	277	301	298	29	30	42	33.66667
cadA	42636	15347	27920.67	4.1286	5.69E-20	9.867561	0.006173	2.52E-16	6.93E-19	9166	9504	8103	8924.333	50739	35283	24513	36845
cadB	4716	1628	-943.333	-1.11854	0.0006	2.134226	0.000787	1	0.001339	9414	9504	7786	8901.333	9980	8630	5264	7958
caiE	2115	1720	-1879.33	-12.7704	5.56E-22	-5.38436	-0.00013	2.46E-18	7.55E-21	2231	1888	1998	2039	195	116	168	159.6667
caiF	5056	4245	-4599.33	-19.7473	0	-8.39152	-0.00033	0	0	5224	4809	4501	4844.667	256	168	312	245.3333
cbiA	8044	7720	-7880.33	-27.9874	0	-11.8488	-0.00058	0	0	8257	8045	8215	8172.333	338	213	325	292
cbiC	3241	2874	-3000	-18.8571	1.13E-24	-7.97167	-0.00022	5E-21	1.84E-23	3346	3093	3065	3168	208	105	191	168
cbiD	6042	4985	-5361.33	-11.6025	2.93E-12	-4.90233	-0.00036	1.3E-08	1.91E-11	6328	5574	5699	5867	642	286	589	505.6667
cbiE	1938	1702	-1797.67	-10.9319	1.7E-11	-4.60521	-0.00012	7.52E-08	1.02E-10	2014	1897	2025	1978.667	261	87	195	181
cbiL	595	377	-52	-1.03467	1.15E-05	2.305009	0.000157	0.050945	3.35E-05	1703	1282	1670	1551.667	1877	1293	1329	1499.667
cbiM	809	359	45	1.021054	4.48E-06	2.433442	0.000238	0.019863	1.37E-05	2279	1887	2246	2137.333	2606	2144	1797	2182.333
cbiT	1912	1412	-1635.67	-11.0967	5.39E-16	-4.66666	-0.00011	2.39E-12	4.94E-15	2015	1581	1797	1797.667	214	103	169	162
ccmH	49	19	5.666667	1.158879	0.000477	2.779798	4.94E-06	1	0.001088	46	26	35	35.66667	45	64	15	41.33333
cdaR	13961	12225	-13048.3	-24.8108	0	-10.4881	-0.00096	0	0	14256	12870	13663	13596.33	704	295	645	548
cdd	655	24	346.3333	1.544264	7.01E-12	3.658299	0.000132	3.1E-08	4.37E-11	664	640	605	636.3333	1260	654	1034	982.6667

Feature ID	Experiment - Range (original values)	Experiment - IQR (original values)	Experiment - Difference (original values)	Experiment - Fold Change (original values)	EDGE test: yccT H202 vs yccT NT , tagwise dispersions - P-value	EDGE test: yccT H202 vs yccT NT , tagwise dispersions - Fold change	EDGE test: yccT H202 vs yccT NT , tagwise dispersions - Weighted difference	EDGE test: yccT H202 vs yccT NT , tagwise dispersions - Bonferroni	EDGE test: yccT H202 vs yccT NT , tagwise dispersions - FDR p-value correction	yccT NT - yccT.1.S28 - Expression values	yccT NT - yccT.2.S29 - Expression values	yccT NT - yccT.3.S30 - Expression values	yccT NT - Means	yccT H202 - yccT.1.H2O2 - Expression values	yccT H202 - yccT.2.H2O2 - Expression values	yccT H202 - yccT.3.H2O2 - Expression values	yccT H202 - Means
cdsA	1829	1410	-1598.33	-4.74902	7.09E-06	-2.01202	-7.9E-05	0.031426	2.12E-05	2188	1854	2032	2024.667	359	476	444	426.3333
celB	6167	267	3249.333	3.559202	1.44E-11	8.27896	0.000722	6.38E-08	8.74E-11	1222	1346	1241	1269.667	4660	1508	7389	4519
celF	779	108	168.6667	1.271459	3.07E-06	2.989818	9.62E-05	0.013616	9.66E-06	687	579	598	621.3333	917	337	1116	790
cheM	7593	793	-1497.33	-1.08702	9.47E-05	2.16834	0.001701	0.419778	0.000241	19840	17741	18534	18705	18058	12986	20579	17207.67
cibB	2455	2137	-2289.33	-12.2775	0	-5.16813	-0.00016	0	0	2616	2330	2531	2492.333	255	161	193	203
cirA	1629	1353	1517.667	11.66276	2.89E-80	27.66329	0.000295	1.28E-76	1.88E-78	154	122	151	142.3333	1725	1751	1504	1660
citA	73	20	-28.3333	-1.07149	3.82E-09	2.202238	3.98E-05	1.69E-05	1.76E-08	432	410	432	424.6667	405	359	425	396.3333
citB	40	12	17.33333	1.348993	1.33E-10	3.196253	8.51E-06	5.91E-07	7.27E-10	59	47	43	49.66667	67	83	51	67
citC	3729	2571	-3169.33	-80.2333	1.1E-86	-33.8082	-0.00024	4.89E-83	8.15E-85	3765	2613	3250	3209.333	36	42	42	40
citD	900	525	-763.333	-61.2632	1.54E-48	-25.522	-5.8E-05	6.8E-45	6.24E-47	907	535	886	776	21	7	10	12.66667
citD2	39	35	-35	-11.5	1.95E-07	-4.78125	-2.4E-06	0.000862	7.23E-07	41	37	37	38.33333	6	2	2	3.333333
citE	3545	2099	-2912	-32.4245	1.39E-39	-13.6047	-0.00022	6.14E-36	4.58E-38	3623	2192	3199	3004.667	107	78	93	92.66667
citF	4935	2162	-3652.67	-8.73324	9.71E-07	-3.67511	-0.00023	0.004303	3.28E-06	5137	2765	4473	4125	603	202	612	472.3333
citG	749	313	-566.667	-6.12048	4.62E-08	-2.56632	-3.2E-05	0.000205	1.86E-07	857	423	752	677.3333	114	108	110	110.6667
clpA	122500	58609	79426.33	3.058086	1.66E-18	7.235759	0.01875	7.35E-15	1.85E-17	40094	38628	37055	38592.33	97264	159555	97237	118018.7
clpB	29105	8844	16308.33	2.638425	2.08E-14	6.250273	0.004075	9.2E-11	1.65E-13	10141	10283	9437	9953.667	21259	38542	18985	26262
cmk	807	636	-726.667	-6.35627	3.47E-11	-2.70557	-4.2E-05	1.54E-07	2.01E-10	915	757	915	862.3333	108	121	178	135.6667
coaA	1001	892	-952	-13.9231	3.53E-33	-5.88584	-6.6E-05	1.56E-29	8.32E-32	1070	1040	967	1025.667	75	77	69	73.66667
cobD	722	604	-668	-6.98209	6.7E-13	-2.94083	-4E-05	2.97E-09	4.7E-12	818	708	813	779.6667	135	96	104	111.6667
cobT	368	78	42.66667	1.048175	5.89E-08	2.483547	0.000102	0.000261	2.34E-07	902	824	931	885.6667	853	1150	782	928.3333
cof	3386	1826	2359.333	5.05616	7.93E-32	11.82645	0.000491	3.51E-28	1.76E-30	592	570	583	581.6667	2458	2409	3956	2941
copA	3368	143	950.3333	1.265506	1.25E-06	2.99388	0.000556	0.005524	4.15E-06	3733	3574	3431	3579.333	3347	6715	3527	4529.667
corE	623	474	-539	-7.54656	2.11E-12	-3.2064	-3.3E-05	9.36E-09	1.41E-11	677	554	633	621.3333	80	54	113	82.33333
cpsG	33	11	19.33333	1.568627	3.38E-14	3.693243	7.16E-06	1.5E-10	2.65E-13	36	30	36	34	47	63	50	53.33333
creA	110	88	89.33333	1.16813	5.38E-13	2.759635	7.28E-05	2.38E-09	3.81E-12	552	516	526	531.3333	614	622	626	620.6667
creB	226	90	126.3333	1.204093	2.52E-12	2.848022	8.91E-05	1.12E-08	1.66E-11	649	589	619	619	712	815	709	745.3333
crl	1019	391	-148	-1.10398	0.000271	2.144266	0.00014	1	0.00064	1562	1652	1500	1571.333	1171	2059	1040	1423.333
crp	17737	14902	-15885	-8.3964	2.38E-08	-3.58595	-0.00101	0.000106	9.99E-08	18989	18073	17036	18032.67	2134	1252	3057	2147.667
csgA	54	12	22.33333	1.492647	6.35E-11	3.515725	8.91E-06	2.81E-07	3.56E-10	43	38	55	45.33333	51	92	60	67.66667
csgB	8	4	5	1.833333	8.21E-06	4.239113	1.56E-06	0.036382	2.43E-05	7	5	6	6	13	10	10	11

Feature ID	Experiment - Range (original values)	Experiment - IQR (original values)	Experiment - Difference (original values)	Experiment - Fold Change (original values)	EDGE test: yccT H202 vs yccT NT , tagwise dispersions - P-value	EDGE test: yccT H202 vs yccT NT , tagwise dispersions - Fold change	EDGE test: yccT H202 vs yccT NT , tagwise dispersions - Weighted difference	EDGE test: yccT H202 vs yccT NT , tagwise dispersions - Bonferroni	EDGE test: yccT H202 vs yccT NT , tagwise dispersions - FDR p-value correction	yccT NT - yccT.1.S28 - Expression values	yccT NT - yccT.2.S29 - Expression values	yccT NT - yccT.3.S30 - Expression values	yccT NT - Means	yccT H202 - yccT.1.H2O2 - Expression values	yccT H202 - yccT.2.H2O2 - Expression values	yccT H202 - yccT.3.H2O2 - Expression values	yccT H202 - Means
csgG	339	10	131.6667	2.286645	3.37E-06	5.346273	3.47E-05	0.014914	1.05E-05	106	96	105	102.3333	287	38	377	234
csiE	1590	1391	-1439	-6.00812	2.55E-10	-2.54861	-8.2E-05	1.13E-06	1.35E-09	1858	1661	1660	1726.333	268	269	325	287.3333
cspA	26817	23565	-24186.7	-13.971	1.33E-07	-6.06072	-0.00169	0.00059	5.01E-07	27380	24906	25868	26051.33	1341	563	3690	1864.667
cspB	2165	1965	-2006.67	-17.8156	2.81E-15	-7.66398	-0.00014	1.24E-11	2.39E-14	2214	2065	2099	2126	100	49	209	119.3333
cspC	23398	19569	-21434.3	-19.1493	0	-8.1184	-0.00154	0	0	24468	20694	22684	22615.33	1125	1070	1348	1181
cspD	9726	8751	-8963	-7.84896	4.11E-09	-3.3537	-0.00056	1.82E-05	1.89E-08	10693	10211	9911	10271.67	1160	967	1799	1308.667
cspE	37910	34924	-35560.7	-12.2939	6.17E-14	-5.26648	-0.00244	2.74E-10	4.74E-13	40197	37494	38437	38709.33	2570	2287	4589	3148.667
cueO	272	74	-37	-1.02542	1.02E-06	2.305922	0.000152	0.004524	3.43E-06	1625	1463	1389	1492.333	1353	1603	1410	1455.333
cutA	641	576	-609	-10.7181	3.38E-26	-4.54414	-4.1E-05	1.5E-22	6.03E-25	680	637	698	671.6667	57	61	70	62.66667
cyaA	8187	6640	-7103.33	-5.26969	1.66E-05	-2.2426	-0.00038	0.073725	4.75E-05	9543	8257	8501	8767	1356	1617	2018	1663.667
cydA	83253	62901	-71024	-6.81132	7.37E-06	-2.87098	-0.00422	0.032646	2.19E-05	90265	76445	83027	83245.67	16109	7012	13544	12221.67
cyoA	1651	1106	1309	3.893884	5.89E-32	9.133652	0.000287	2.61E-28	1.32E-30	406	433	518	452.3333	1539	1688	2057	1761.333
cyoB	8886	1350	3887.333	3.84439	1.84E-11	9.093937	0.000865	8.16E-08	1.1E-10	1105	1349	1646	1366.667	3072	9991	2699	5254
cyoC	3428	124	1266.333	4.315009	2.44E-08	10.23621	0.000276	0.000108	1.02E-07	319	370	457	382	704	3747	494	1648.333
cyoD	2988	141	1120.667	5.271919	7.18E-09	12.52887	0.000236	3.18E-05	3.19E-08	236	238	313	262.3333	546	3224	379	1383
cyoE	5555	158	2041	3.6	6.77E-08	8.546742	0.000463	0.0003	2.66E-07	682	760	913	785	1323	6237	918	2826
cysA	32	5	-14.3333	-1.15751	3.56E-07	2.041142	8.54E-06	0.001578	1.28E-06	119	97	100	105.3333	87	94	92	91
cysC	42	1	-13.6667	-1.1627	1.47E-06	2.02398	7.81E-06	0.006527	4.85E-06	88	89	116	97.66667	74	88	90	84
cysG	1636	1400	-1496.33	-5.94383	1.63E-09	-2.51392	-8.4E-05	7.2E-06	7.86E-09	1794	1684	1919	1799	283	341	284	302.6667
cysM	523	429	-477.667	-11.2357	2.1E-21	-4.75544	-3.2E-05	9.3E-18	2.77E-20	554	481	538	524.3333	52	31	57	46.66667
cytR	2150	1787	-1860	-4.78819	6.66E-05	-2.03849	-9.3E-05	0.29518	0.000173	2490	2274	2289	2351	487	340	646	491
dacA	1310	1031	-1159.33	-6.23795	5.75E-10	-2.62508	-6.6E-05	2.55E-06	2.92E-09	1489	1253	1400	1380.667	222	263	179	221.3333
dacD	901	804	-835.333	-6.99522	3.3E-15	-2.96322	-5E-05	1.46E-11	2.79E-14	1028	943	953	974.6667	139	127	152	139.3333
dadA	3171	958	1889.667	5.128915	4.22E-23	11.9138	0.000391	1.87E-19	6.17E-22	370	469	534	457.6667	2074	1427	3541	2347.333
dadX	1071	629	758.3333	2.861702	6.57E-28	6.70353	0.000181	2.91E-24	1.24E-26	366	390	466	407.3333	1019	1041	1437	1165.667
dapA	7195	6335	-6658	-6.31365	1.06E-07	-2.6802	-0.00039	0.000469	4.02E-07	8284	7551	7898	7911	1216	1089	1454	1253
dapE	1749	1477	-1618	-5.63168	6.52E-09	-2.38306	-8.9E-05	2.89E-05	2.91E-08	2079	1833	1990	1967.333	330	362	356	349.3333
dctA	6357	5139	-5699.67	-7.10461	6.04E-07	-3.02415	-0.00035	0.002674	2.1E-06	6902	6127	6871	6633.333	988	545	1268	933.6667
dcuC	1656	1297	-1450	-5.47531	4.58E-06	-2.29788	-7.8E-05	0.020275	1.4E-05	1878	1632	1812	1774	335	415	222	324
ddg	1591	1231	-1352.67	-7.43106	6.88E-05	-3.21176	-8.4E-05	0.304753	0.000178	1641	1391	1657	1563	160	66	405	210.3333

Feature ID	Experiment - Range (original values)	Experiment - IQR (original values)	Experiment - Difference (original values)	Experiment - Fold Change (original values)	EDGE test: yccT H202 vs yccT NT , tagwise dispersions - P-value	EDGE test: yccT H202 vs yccT NT , tagwise dispersions - Fold change	EDGE test: yccT H202 vs yccT NT , tagwise dispersions - Weighted difference	EDGE test: yccT H202 vs yccT NT , tagwise dispersions - Bonferroni	EDGE test: yccT H202 vs yccT NT , tagwise dispersions - FDR p-value correction	yccT NT - yccT.1.S28 - Expression values	yccT NT - yccT.2.S29 - Expression values	yccT NT - yccT.3.S30 - Expression values	yccT NT - Means	yccT H202 - yccT.1.H2O2 - Expression values	yccT H202 - yccT.2.H2O2 - Expression values	yccT H202 - yccT.3.H2O2 - Expression values	yccT H202 - Means
deaD	3148	922	-412.667	-1.07212	0.000102	2.199678	0.000571	0.45339	0.000259	6887	5297	6219	6134.333	5897	4060	7208	5721.667
deoA	2814	347	1518	1.829508	8.1E-09	4.331419	0.000474	3.59E-05	3.58E-08	2054	1707	1729	1830	4395	1581	4068	3348
deoB	31835	28736	30500.33	6.869211	2.22E-39	16.27348	0.006177	9.84E-36	7.03E-38	5577	4995	5018	5196.667	36507	36830	33754	35697
deoC	2719	1037	2072.333	2.821565	1.49E-18	6.664239	0.000502	6.6E-15	1.67E-17	1210	1143	1060	1137.667	3779	2180	3671	3210
deoD	9714	6564	8220	4.11521	6.48E-28	9.774565	0.001802	2.87E-24	1.23E-26	2856	2539	2521	2638.667	11238	12235	9103	10858.67
dgoA	110	39	-19.6667	-1.16253	0.000504	2.049428	1.15E-05	1	0.001145	159	119	144	140.6667	105	184	74	121
dinF	2666	1452	2052.333	3.370812	3.1E-25	7.937938	0.000468	1.38E-21	5.21E-24	919	832	846	865.6667	2958	2298	3498	2918
dinG	1222	85	492.3333	1.279788	9.71E-09	3.002887	0.000274	4.3E-05	4.25E-08	1830	1698	1751	1759.667	2091	1745	2920	2252
dinI	1252	763	952.6667	4.528395	2.67E-41	10.60562	0.000202	1.18E-37	9.09E-40	266	274	270	270	1033	1117	1518	1222.667
dinP	4357	2300	3188	5.262032	4.31E-32	12.37887	0.000662	1.91E-28	9.75E-31	843	685	716	748	3750	3016	5042	3936
dlhH	996	796	-865.667	-7.64194	4.63E-15	-3.24052	-5.4E-05	2.05E-11	3.85E-14	1096	927	965	996	131	100	160	130.3333
dmsA	42632	38427	-39804	-9.3845	2.29E-08	-3.96285	-0.0026	0.000102	9.62E-08	45140	43742	44772	44551.33	6419	2508	5315	4747.333
dmsA1	9060	7673	-8200	-13.4305	4.44E-16	-5.67372	-0.00057	1.97E-12	4.09E-15	9517	8666	8396	8859.667	799	457	723	659.6667
dmsA2	1638	1444	-1541.33	-30.641	1.03E-62	-12.9104	-0.00011	4.58E-59	4.92E-61	1603	1495	1682	1593.333	61	51	44	52
dmsA3	4590	3885	-4038	-5.89652	3.54E-05	-2.46985	-0.00023	0.156737	9.57E-05	5175	4591	4822	4862.667	1183	585	706	824.6667
dmsC	3130	510	-346.667	-1.08966	0.001107	2.174908	0.000385	1	0.002353	4610	3958	4072	4213.333	5131	2001	4468	3866.667
dmsC1	979	153	494	1.502714	6.52E-11	3.572823	0.000197	2.89E-07	3.65E-10	1027	940	981	982.6667	1919	1134	1377	1476.667
dnaA	3981	3423	-3566.33	-5.37945	1.96E-05	-2.28751	-0.00019	0.08674	5.52E-05	4562	4317	4263	4380.667	840	581	1022	814.3333
dnaB	1061	939	-982.333	-8.22304	7.34E-17	-3.4793	-6.2E-05	3.25E-13	7.24E-16	1175	1066	1114	1118.333	114	167	127	136
dnaJ	2561	2056	2312.333	4.465035	1.06E-32	10.56891	0.000498	4.69E-29	2.43E-31	692	668	642	667.3333	3012	3203	2724	2979.667
dnaK	33464	30052	31645.33	10.42854	3.24E-48	24.61632	0.006187	1.44E-44	1.31E-46	3243	3488	3338	3356.333	36707	33390	34908	35001.67
dnaX	2718	2186	-2456.67	-5.81385	3.73E-08	-2.46317	-0.00014	0.000165	1.53E-07	3185	2695	3021	2967	467	509	555	510.3333
dniR	2866	2472	-2595.67	-6.27217	5.47E-09	-2.65981	-0.00015	2.43E-05	2.47E-08	3282	3000	2982	3088	416	551	510	492.3333
dpiA	1333	1032	-1124.67	-7.62868	1.99E-15	-3.21549	-6.9E-05	8.8E-12	1.71E-14	1487	1200	1196	1294.333	164	191	154	169.6667
dpiB	3339	2611	-2873.67	-17.5788	1.04E-30	-7.45526	-0.00021	4.62E-27	2.21E-29	3488	2880	2773	3047	162	149	209	173.3333
dppC	2233	1554	-1972	-6.24933	3.26E-09	-2.64001	-0.00011	1.44E-05	1.52E-08	2540	1947	2556	2347.667	393	323	411	375.6667
dppD	3190	2229	-2798.33	-7.04392	2.52E-10	-2.97226	-0.00017	1.12E-06	1.33E-09	3617	2685	3482	3261.333	427	506	456	463
dppF	2571	1437	-2024.67	-5.69397	6.1E-07	-2.38704	-0.00011	0.002703	2.12E-06	2939	1886	2543	2456	449	477	368	431.3333
dps	462319	108981	243745	83.37411	3.46E-39	198.2094	0.045511	1.53E-35	1.09E-37	3033	3114	2730	2959	163049	465049	112014	246704
dsbC	1370	1185	-1279.33	-4.78128	1.19E-06	-2.02476	-6.4E-05	0.005291	3.98E-06	1688	1533	1632	1617.667	318	348	349	338.3333

Feature ID	Experiment - Range (original values)	Experiment - IQR (original values)	Experiment - Difference (original values)	Experiment - Fold Change (original values)	EDGE test: yccT H202 vs yccT NT , tagwise dispersions - P-value	EDGE test: yccT H202 vs yccT NT , tagwise dispersions - Fold change	EDGE test: yccT H202 vs yccT NT , tagwise dispersions - Weighted difference	EDGE test: yccT H202 vs yccT NT , tagwise dispersions - Bonferroni	EDGE test: yccT H202 vs yccT NT , tagwise dispersions - FDR p-value correction	yccT NT - yccT.1.S28 - Expression values	yccT NT - yccT.2.S29 - Expression values	yccT NT - yccT.3.S30 - Expression values	yccT NT - Means	yccT H202 - yccT.1.H2O2 - Expression values	yccT H202 - yccT.2.H2O2 - Expression values	yccT H202 - yccT.3.H2O2 - Expression values	yccT H202 - Means
dsbD	1405	1202	-1273.33	-6.4964	3.43E-11	-2.74688	-7.4E-05	1.52E-07	1.99E-10	1604	1417	1494	1505	199	281	215	231.6667
eda	594	165	-84.3333	-1.08083	1.93E-05	2.199085	0.000105	0.085435	5.44E-05	1181	1047	1155	1127.667	990	1367	773	1043.333
efp	2174	1895	-1953	-5.03512	1.12E-05	-2.12663	-0.0001	0.049462	3.26E-05	2591	2315	2405	2437	417	615	420	484
ego	11827	8748	-10369.3	-141.76	1.06E-69	-60.2761	-0.0008	4.7E-66	5.6E-68	11876	8814	10639	10443	66	49	106	73.66667
emrA	1600	1320	-1439.33	-6.41103	3.72E-11	-2.72075	-8.4E-05	1.65E-07	2.14E-10	1832	1570	1714	1705.333	250	232	316	266
emrR	2002	1846	-1885	-9.16017	6.47E-13	-3.9072	-0.00012	2.87E-09	4.55E-12	2144	2077	2127	2116	231	142	320	231
eno	11159	805	842.6667	1.072517	9.88E-05	2.557027	0.001407	0.437886	0.00025	12456	10800	11605	11620.33	11294	18627	7468	12463
entA	598	26	228	6.516129	7.6E-10	15.49524	4.68E-05	3.37E-06	3.82E-09	43	33	48	41.33333	108	631	69	269.3333
entB	840	65	341.6667	8.824427	2.17E-12	20.94755	6.81E-05	9.6E-09	1.44E-11	47	43	41	43.66667	167	881	108	385.3333
entC	258	169	201	6.481818	4.63E-67	15.24584	4.09E-05	2.05E-63	2.31E-65	35	34	41	36.66667	204	292	217	237.6667
entD	34	5	15	1.357143	1.77E-11	3.199868	7.22E-06	7.82E-08	1.06E-10	46	37	43	42	48	71	52	57
entE	275	103	161	4.744186	6.29E-27	11.19281	3.43E-05	2.79E-23	1.17E-25	48	44	37	43	147	312	153	204
entF	2490	1032	1538	7.143808	5.57E-31	16.96173	0.000311	2.47E-27	1.2E-29	276	219	256	250.3333	1368	2709	1288	1788.333
envF	32	3	13.66667	1.745455	1.24E-07	4.068904	4.43E-06	0.000548	4.66E-07	17	18	20	18.33333	36	14	46	32
envZ	1725	1528	-1638	-6.1028	2.62E-10	-2.58597	-9.4E-05	1.16E-06	1.38E-09	2001	1858	2018	1959	293	340	330	321
eutD	1267	775	-1043	-14.0921	8.86E-25	-5.95991	-7.2E-05	3.93E-21	1.45E-23	1324	853	1191	1122.667	78	57	104	79.66667
eutE	2213	1340	-1828.67	-9.23724	7.24E-14	-3.91636	-0.00012	3.21E-10	5.51E-13	2371	1552	2229	2050.667	212	158	296	222
eutJ	777	390	-587.667	-5.1385	1.04E-05	-2.17451	-3.1E-05	0.046172	3.06E-05	871	533	785	729.6667	143	94	189	142
eutN	1135	697	-936	-15.2538	8.65E-17	-6.43799	-6.6E-05	3.83E-13	8.5E-16	1163	779	1063	1001.667	82	28	87	65.66667
eutP	1451	1150	-1307.33	-59.5373	1.24E-78	-25.2174	-9.9E-05	5.51E-75	7.87E-77	1464	1170	1355	1329.667	20	13	34	22.33333
eutQ	3554	2469	-2993.67	-35.5423	2.88E-36	-15.0463	-0.00022	1.28E-32	7.82E-35	3605	2562	3074	3080.333	93	51	116	86.66667
eutS	1009	757	-865.333	-47.3571	3.64E-73	-19.9823	-6.5E-05	1.61E-69	2.02E-71	1020	776	856	884	19	11	26	18.66667
eutT	2609	1716	-2185	-26.5058	1.76E-39	-11.2291	-0.00016	7.78E-36	5.64E-38	2677	1792	2343	2270.667	76	68	113	85.66667
exbB	3683	1475	2374.667	3.797016	1.14E-23	8.887166	0.000521	5.07E-20	1.72E-22	894	810	843	849	2860	2318	4493	3223.667
exbD	1156	762	886	3.360568	3.24E-34	7.911124	0.000202	1.44E-30	8.12E-33	410	341	375	375.3333	1137	1150	1497	1261.333
exo	535	424	-462.333	-5.90106	4.13E-09	-2.50967	-2.6E-05	1.83E-05	1.89E-08	604	510	556	556.6667	86	69	128	94.33333
fabG	5078	4514	-4569	-5.42447	2.26E-05	-2.29176	-0.00025	0.10008	6.29E-05	5954	5407	5444	5601.667	876	1329	893	1032.667
fabH	1162	977	-1070	-5.45215	2.39E-08	-2.30033	-5.8E-05	0.000106	1E-07	1349	1211	1371	1310.333	234	278	209	240.3333
fadD	2323	56	842.3333	2.222545	1.36E-06	5.163928	0.000224	0.006042	4.51E-06	671	670	726	689	1571	350	2673	1531.333
fadH	2123	1498	-1767	-8.21224	4.06E-13	-3.47943	-0.00011	1.8E-09	2.89E-12	2296	1752	1988	2012	254	173	308	245

Feature ID	Experiment - Range (original values)	Experiment - IQR (original values)	Experiment - Difference (original values)	Experiment - Fold Change (original values)	EDGE test: yccT H202 vs yccT NT , tagwise dispersions - P-value	EDGE test: yccT H202 vs yccT NT , tagwise dispersions - Fold change	EDGE test: yccT H202 vs yccT NT , tagwise dispersions - Weighted difference	EDGE test: yccT H202 vs yccT NT , tagwise dispersions - Bonferroni	EDGE test: yccT H202 vs yccT NT , tagwise dispersions - FDR p-value correction	yccT NT - yccT.1.S28 - Expression values	yccT NT - yccT.2.S29 - Expression values	yccT NT - yccT.3.S30 - Expression values	yccT NT - Means	yccT H202 - yccT.1.H2O2 - Expression values	yccT H202 - yccT.2.H2O2 - Expression values	yccT H202 - yccT.3.H2O2 - Expression values	yccT H202 - Means
fadR	3659	2948	-3109	-5.02373	0.000833	-2.1498	-0.00016	1	0.001818	4064	3684	3897	3881.667	736	405	1177	772.6667
fdnI	220	15	36	1.089256	8.98E-09	2.591089	5.01E-05	3.98E-05	3.95E-08	391	406	413	403.3333	567	404	347	439.3333
fdoG	26751	11646	16622.67	2.411931	7.75E-16	5.720279	0.004319	3.43E-12	6.98E-15	12676	10260	12383	11773	24147	37011	24029	28395.67
fdoH	5470	487	2371.333	1.987096	6.26E-10	4.732533	0.000697	2.77E-06	3.17E-09	2618	2048	2541	2402.333	3775	7518	3028	4773.667
fdoI	2000	336	561.6667	1.343948	9.82E-07	3.201843	0.000279	0.004349	3.31E-06	1843	1448	1608	1633	1784	3400	1400	2194.667
fdx	658	141	74.33333	1.158381	4.63E-05	2.754085	6.4E-05	0.204973	0.000123	507	413	488	469.3333	347	971	313	543.6667
feoA	161	56	-39.6667	-1.16436	1.52E-05	2.013336	2.22E-05	0.06742	4.37E-05	285	268	290	281	229	167	328	241.3333
fepA	245	174	204.6667	1.617085	2E-21	3.811962	7.28E-05	8.88E-18	2.65E-20	331	344	320	331.6667	505	565	539	536.3333
fepB	2897	1834	2387	32.96875	9.01E-85	77.74195	0.000446	3.99E-81	6.34E-83	85	56	83	74.66667	2515	1917	2953	2461.667
fepC	165	70	102.6667	1.365796	3.75E-17	3.232722	4.88E-05	1.66E-13	3.79E-16	297	247	298	280.6667	371	412	367	383.3333
fepD	1891	1539	1669.333	34.38667	5.9E-106	80.82413	0.000312	2.6E-102	5.4E-104	51	42	57	50	1635	1590	1933	1719.333
fepG	252	195	214.3333	2.229446	4.31E-32	5.274139	5.8E-05	1.91E-28	9.75E-31	186	149	188	174.3333	381	401	384	388.6667
fes	1470	1090	1291.667	51.98684	9.5E-114	121.3975	0.00024	4.2E-110	9.3E-112	20	28	28	25.33333	1118	1490	1343	1317
fhuA	13922	9647	11781.33	29.89943	3.48E-89	70.33547	0.002204	1.54E-85	2.66E-87	418	409	396	407.6667	12193	10056	14318	12189
fhuB	133	14	14.33333	1.085487	1.65E-07	2.554569	2.03E-05	0.000729	6.14E-07	172	173	158	167.6667	122	255	169	182
fhuC	298	225	265	3.564516	8.83E-46	8.378069	5.96E-05	3.91E-42	3.43E-44	96	109	105	103.3333	330	394	381	368.3333
fhuD	140	29	45.66667	1.32619	1.55E-10	3.13555	2.32E-05	6.87E-07	8.39E-10	160	119	141	140	131	259	167	185.6667
fhuE	344	209	249	2.540206	1.65E-34	5.968911	6.26E-05	7.29E-31	4.17E-33	175	159	151	161.6667	369	368	495	410.6667
fhuF	371	102	223.6667	2.336653	4.97E-22	5.46987	5.84E-05	2.2E-18	6.8E-21	158	161	183	167.3333	381	263	529	391
fimH	86	37	57.33333	2.178082	7.62E-24	5.135316	1.57E-05	3.38E-20	1.17E-22	53	35	58	48.66667	90	121	107	106
fimY	32	10	9	1.102662	4.16E-11	2.604994	1.09E-05	1.84E-07	2.38E-10	106	74	83	87.66667	93	91	106	96.66667
fis	410	341	-377	-8.64189	2.63E-19	-3.65794	-2.4E-05	1.16E-15	3.07E-18	454	391	434	426.3333	44	50	54	49.33333
fixA	117	93	-102.667	-5.8125	1.12E-06	-2.452	-5.7E-06	0.004978	3.77E-06	132	127	113	124	29	15	20	21.33333
fklB	620	464	-527.333	-5.27568	9.26E-09	-2.22493	-2.8E-05	4.1E-05	4.06E-08	735	584	633	650.6667	120	135	115	123.3333
fldA	2330	1890	-1992.67	-4.8125	0.000195	-2.05018	-0.0001	0.862663	0.000471	2652	2424	2470	2515.333	534	322	712	522.6667
flgH	448	370	-383.667	-8.06135	1.01E-06	-3.38859	-2.4E-05	0.004455	3.38E-06	429	471	414	438	44	96	23	54.33333
flgI	435	362	-376.333	-5.42745	0.000173	-2.27688	-2E-05	0.765891	0.000422	478	438	468	461.3333	76	136	43	85
flhC	2442	1971	-2161.33	-6.77382	5.48E-09	-2.88774	-0.00013	2.43E-05	2.47E-08	2708	2312	2587	2535.667	341	266	516	374.3333
flhD	2644	2256	-2342.33	-8.74752	5.11E-09	-3.74288	-0.00015	2.26E-05	2.32E-08	2794	2547	2593	2644.667	291	150	466	302.3333
fliM	579	512	-528.333	-5.14921	4.86E-07	-2.17285	-2.8E-05	0.002154	1.71E-06	644	678	645	655.6667	132	151	99	127.3333

Feature ID	Experiment - Range (original values)	Experiment - IQR (original values)	Experiment - Difference (original values)	Experiment - Fold Change (original values)	EDGE test: yccT H2O2 vs yccT NT , tagwise dispersions - P-value	EDGE test: yccT H2O2 vs yccT NT , tagwise dispersions - Fold change	EDGE test: yccT H2O2 vs yccT NT , tagwise dispersions - Weighted difference	EDGE test: yccT H2O2 vs yccT NT , tagwise dispersions - Bonferroni	EDGE test: yccT H2O2 vs yccT NT , tagwise dispersions - FDR p-value correction	yccT NT - yccT.1.S28 - Expression values	yccT NT - yccT.2.S29 - Expression values	yccT NT - yccT.3.S30 - Expression values	yccT NT - Means	yccT H2O2 - yccT.1.H2O2 - Expression values	yccT H2O2 - yccT.2.H2O2 - Expression values	yccT H2O2 - yccT.3.H2O2 - Expression values	yccT H2O2 - Means
fliQ	133	118	-122.667	-6.1831	4.76E-09	-2.61401	-7E-06	2.11E-05	2.16E-08	154	145	140	146.3333	21	28	22	23.66667
fliR	397	291	-346	-12.1613	1.92E-20	-5.17064	-2.4E-05	8.51E-17	2.4E-19	326	417	388	377	35	20	38	31
fljB	393083	60778	5219.667	1.013565	0.000579	2.417877	0.042364	1	0.001298	439248	339618	375481	384782.3	314703	624193	231110	390002
fnr	5403	523	618	1.148308	0.000279	2.686038	0.000547	1	0.000658	4422	4180	3899	4167	5160	1896	7299	4785
focA	2918	2788	-2860.33	-27.5666	7.09E-43	-11.651	-0.00021	3.14E-39	2.51E-41	2999	2890	3015	2968	102	124	97	107.6667
folB	134	85	-107	-5.58571	4.6E-07	-2.35393	-5.8E-06	0.002037	1.62E-06	155	109	127	130.3333	25	21	24	23.33333
folC	1163	919	-1018	-6.00656	7.29E-11	-2.5335	-5.7E-05	3.23E-07	4.06E-10	1349	1115	1200	1221.333	196	228	186	203.3333
folE	2402	1473	1799	2.371886	2.61E-19	5.587567	0.000468	1.16E-15	3.06E-18	1439	1276	1219	1311.333	2961	2749	3621	3110.333
folK	147	108	-121.667	-4.80208	3.46E-05	-2.04245	-6.1E-06	0.153451	9.4E-05	150	145	166	153.6667	19	37	40	32
folP	225	39	17.33333	1.027397	6.06E-09	2.429134	7.03E-05	2.68E-05	2.72E-08	696	584	618	632.6667	551	776	623	650
foxA	493	72	231.3333	1.332216	1.04E-12	3.150933	0.000117	4.6E-09	7.14E-12	742	625	722	696.3333	794	1118	871	927.6667
fpr	2938	1621	2030	2.296572	3.38E-18	5.392785	0.000536	1.5E-14	3.69E-17	1664	1531	1502	1565.667	3152	3195	4440	3595.667
frdA	61507	53011	-55920.7	-7.66225	5.49E-08	-3.22923	-0.00346	0.000243	2.19E-07	67528	63995	61420	64314.33	10751	6021	8409	8393.667
frr	3086	2595	-2764.67	-5.70715	2.19E-07	-2.42668	-0.00015	0.00097	8.08E-07	3589	3152	3315	3352	503	557	702	587.3333
fruF	667	424	-563	-10.1793	2.86E-20	-4.28742	-3.7E-05	1.27E-16	3.55E-19	662	487	724	624.3333	63	64	57	61.33333
fruK	350	230	-284.667	-5.49474	1.13E-06	-2.30982	-1.5E-05	0.005006	3.78E-06	355	295	394	348	65	81	44	63.33333
fruR	3964	3603	-3765.33	-13.851	0	-5.88376	-0.00026	0	0	4064	3875	4236	4058.333	272	272	335	293
fsr	734	624	-662.667	-6.52222	3.3E-11	-2.77747	-3.9E-05	1.46E-07	1.92E-10	790	735	823	782.6667	111	89	160	120
ftn	2248	2040	-2119	-15.2534	6.96E-29	-6.43757	-0.00015	3.08E-25	1.38E-27	2241	2180	2382	2267.667	172	134	140	148.6667
ftsJ	2647	260	29.66667	1.006936	2.21E-05	2.359357	0.000453	0.097935	6.17E-05	4495	4203	4133	4277	3943	3165	5812	4306.667
fucA	185	174	-179	-6.77419	4.44E-12	-2.87111	-1.1E-05	1.97E-08	2.84E-11	204	212	214	210	30	29	34	31
fucI	681	57	88.66667	1.119874	4.05E-06	2.653466	9.53E-05	0.017935	1.25E-05	760	703	756	739.6667	1121	440	924	828.3333
fucK	218	64	28.66667	1.060224	4.38E-09	2.508843	5.59E-05	1.94E-05	2E-08	513	466	449	476	595	377	542	504.6667
fucO	335	296	-312.667	-4.86008	6.38E-07	-2.05467	-1.6E-05	0.002829	2.21E-06	396	409	376	393.6667	89	80	74	81
fucU	75	8	20	1.167598	3.5E-09	2.779346	1.66E-05	1.55E-05	1.62E-08	123	115	120	119.3333	174	145	99	139.3333
fur	3075	166	553.6667	1.173183	5.12E-06	2.745435	0.000435	0.022674	1.55E-05	3285	3187	3119	3197	3671	2253	5328	3750.667
fxsA	595	44	370	2.12462	7.42E-15	5.00099	0.000103	3.29E-11	6.09E-14	345	315	327	329	816	371	910	699
galE	205	67	37	1.020605	6.91E-07	2.412421	0.000198	0.003063	2.38E-06	1825	1804	1758	1795.667	1937	1732	1829	1832.667
galF	2903	2687	-2720.33	-6.44793	2.36E-08	-2.7435	-0.00016	0.000105	9.9E-08	3277	3194	3188	3219.667	501	374	623	499.3333
gapA	12368	1401	-2170.33	-1.10108	0.000413	2.164899	0.002144	1	0.000951	24464	22531	23932	23642.33	23108	26838	14470	21472

Feature ID	Experiment - Range (original values)	Experiment - IQR (original values)	Experiment - Difference (original values)	Experiment - Fold Change (original values)	EDGE test: yccT H202 vs yccT NT , tagwise dispersions - P-value	EDGE test: yccT H202 vs yccT NT , tagwise dispersions - Fold change	EDGE test: yccT H202 vs yccT NT , tagwise dispersions - Weighted difference	EDGE test: yccT H202 vs yccT NT , tagwise dispersions - Bonferroni	EDGE test: yccT H202 vs yccT NT , tagwise dispersions - FDR p-value correction	yccT NT - yccT.1.S28 - Expression values	yccT NT - yccT.2.S29 - Expression values	yccT NT - yccT.3.S30 - Expression values	yccT NT - Means	yccT H202 - yccT.1.H2O2 - Expression values	yccT H202 - yccT.2.H2O2 - Expression values	yccT H202 - yccT.3.H2O2 - Expression values	yccT H202 - Means
garD	38023	31932	-34558.3	-49.6737	0	-21.0828	-0.00261	0	0	38369	32768	34668	35268.33	836	346	948	710
garK	16725	13111	-14922.7	-42.8393	0	-18.1501	-0.00112	0	0	16923	13523	15392	15279.33	412	198	460	356.6667
garL	20507	17280	-18941.3	-58.6308	0	-24.9318	-0.00144	0	0	20653	17657	19500	19270	377	146	463	328.6667
garR	37221	28401	-32999.3	-19.4974	2.22E-16	-8.26372	-0.00238	9.84E-13	2.11E-15	38125	30510	35715	34783.33	2109	904	2339	1784
gcd	730	17	194.3333	1.647778	2.85E-06	3.926471	6.82E-05	0.012627	9E-06	348	274	278	300	291	961	231	494.3333
gcl	25	18	-18.3333	-1.14139	9.35E-08	2.068482	1.23E-05	0.000414	3.59E-07	148	151	145	148	136	126	127	129.6667
gcpE	4396	3794	-4004.67	-5.32937	4.18E-06	-2.2611	-0.00021	0.01852	1.29E-05	5251	4664	4874	4929.667	870	855	1050	925
gcvH	16778	14252	-15253	-8.19029	2.2E-11	-3.46911	-0.00096	9.76E-08	1.3E-10	18767	16360	16996	17374.33	1989	2108	2267	2121.333
gcvR	1679	1460	-1530.67	-7.26467	1.52E-13	-3.0877	-9.3E-05	6.75E-10	1.13E-12	1897	1678	1750	1775	218	218	297	244.3333
gcvT	37517	33302	-34689.7	-11.8304	5.49E-13	-5.0438	-0.00237	2.43E-09	3.88E-12	39532	36515	37631	37892.67	3213	2015	4381	3203
gdhA	2794	2387	-2595	-6.84459	7.74E-11	-2.89576	-0.00015	3.43E-07	4.3E-10	3228	2823	3066	3039	436	434	462	444
gip	60	9	16.66667	1.154799	1.24E-08	2.744128	1.47E-05	5.51E-05	5.38E-08	110	111	102	107.6667	143	145	85	124.3333
glgA	4623	3712	-4070.67	-5.0558	0.000482	-2.14412	-0.00021	1	0.001097	5200	5099	4924	5074.333	1212	577	1222	1003.667
glgC	5703	4870	-5124.33	-6.64354	3.42E-06	-2.82106	-0.0003	0.015157	1.07E-05	6214	5930	5953	6032.333	1060	511	1153	908
glgS	2859	2021	-2278.67	-4.94916	0.000858	-2.12386	-0.00012	1	0.001863	2832	3159	2576	2855.667	555	300	876	577
glgX	2957	2655	-2752.33	-6.4038	1.65E-08	-2.72985	-0.00016	7.31E-05	7.01E-08	3372	3295	3118	3261.667	463	415	650	509.3333
glk	2181	2034	-2068	-5.53841	1.01E-07	-2.35605	-0.00011	0.000449	3.87E-07	2576	2533	2462	2523.667	428	395	544	455.6667
glmU	5268	4822	-4953.67	-7.38908	1.19E-09	-3.14141	-0.0003	5.29E-06	5.88E-09	5892	5584	5711	5729	762	624	940	775.3333
glnK	12	2	3	1.391304	0.00028	3.235728	1.36E-06	1	0.000659	7	4	12	7.666667	7	16	9	10.66667
gloA	2055	89	733	1.575654	1.13E-07	3.735555	0.000271	0.0005	4.28E-07	1333	1243	1244	1273.333	1388	3298	1333	2006.333
glpA	72457	62272	-66532	-13.5968	3.37E-12	-5.77914	-0.00462	1.49E-08	2.18E-11	75131	68385	71925	71813.67	6113	2674	7058	5281.667
glpB	25551	21165	-23359.7	-13.4629	2.04E-11	-5.7225	-0.00162	9.03E-08	1.21E-10	26410	23365	25927	25234	2200	859	2564	1874.333
glpC	18126	14937	-16146.7	-8.34051	1.16E-06	-3.53238	-0.00102	0.00513	3.87E-06	19102	17697	18240	18346.33	2863	976	2760	2199.667
glpE	350	102	187	1.685819	1.17E-17	3.951484	6.28E-05	5.17E-14	1.21E-16	253	247	318	272.6667	427	355	597	459.6667
glpF	34087	24371	-27006	-5.04827	0.02045	-2.16708	-0.00141	1	0.03381	35665	31264	34102	33677	6893	1578	11542	6671
gltA	45476	22604	30664.33	2.826635	2E-18	6.705016	0.007449	8.87E-15	2.22E-17	18126	15518	16718	16787.33	42039	60994	39322	47451.67
glxK	43	7	-8.33333	-1.11468	8.62E-05	2.13523	7.18E-06	0.381941	0.000221	82	77	84	81	88	85	45	72.66667
glyQ	1559	1350	-1462.33	-5.08473	2.42E-07	-2.1567	-7.6E-05	0.001072	8.86E-07	1895	1686	1880	1820.333	336	336	402	358
gmd	54	4	25.33333	1.535211	3.36E-12	3.611501	9.66E-06	1.49E-08	2.18E-11	48	42	52	47.33333	52	96	70	72.66667
gntR	1435	1196	-1243.67	-5.98131	1.24E-05	-2.56275	-7.1E-05	0.054915	3.6E-05	1559	1426	1495	1493.333	230	124	395	249.6667

Feature ID	Experiment - Range (original values)	Experiment - IQR (original values)	Experiment - Difference (original values)	Experiment - Fold Change (original values)	EDGE test: yccT H202 vs yccT NT , tagwise dispersions - P-value	EDGE test: yccT H202 vs yccT NT , tagwise dispersions - Fold change	EDGE test: yccT H202 vs yccT NT , tagwise dispersions - Weighted difference	EDGE test: yccT H202 vs yccT NT , tagwise dispersions - Bonferroni	EDGE test: yccT H202 vs yccT NT , tagwise dispersions - FDR p-value correction	yccT NT - yccT.1.S28 - Expression values	yccT NT - yccT.2.S29 - Expression values	yccT NT - yccT.3.S30 - Expression values	yccT NT - Means	yccT H202 - yccT.1.H2O2 - Expression values	yccT H202 - yccT.2.H2O2 - Expression values	yccT H202 - yccT.3.H2O2 - Expression values	yccT H202 - Means
gor	466	264	-21	-1.00713	3.04E-06	2.348579	0.000311	0.013474	9.56E-06	3175	2709	3014	2966	2750	3173	2912	2945
gpmA	66101	35117	45881.33	12.75841	9.98E-41	30.25281	0.008882	4.42E-37	3.35E-39	4257	3767	3682	3902	40683	69783	38884	49783.33
gppA	2386	2053	-2151	-5.05339	1.83E-05	-2.13718	-0.00011	0.08113	5.19E-05	2820	2530	2695	2681.667	434	681	477	530.6667
gpsA	5303	4435	-4834.33	-5.52512	2.77E-06	-2.33864	-0.00026	0.012289	8.79E-06	6274	5515	5919	5902.667	971	1154	1080	1068.333
gpt	1053	913	-967.333	-15.8061	1.41E-37	-6.69677	-6.8E-05	6.24E-34	4.11E-36	1109	981	1008	1032.667	56	68	72	65.33333
greA	1054	187	-335.333	-1.16109	0.000205	2.029775	0.000194	0.909701	0.000495	2591	2286	2374	2417	2187	1537	2521	2081.667
grxA	6520	5821	6174.667	6.091809	1.68E-39	14.38701	0.001264	7.43E-36	5.42E-38	1281	1189	1168	1212.667	7010	7688	7464	7387.333
grxB	3868	1487	-751.333	-1.1519	0.001378	2.062976	0.000471	1	0.002871	6220	5708	5165	5697.667	4221	7243	3375	4946.333
gtrA	50	22	-32.3333	-7.0625	0.000234	-2.99152	-2E-06	1	0.000557	27	54	32	37.66667	4	5	7	5.333333
gtrC	12	8	-4	-1.13636	0.000137	2.067084	2.78E-06	0.606078	0.000338	37	34	29	33.33333	25	26	37	29.33333
guaB	1254	928	-1046	-9.27968	3.61E-18	-3.904	-6.8E-05	1.6E-14	3.9E-17	1358	1104	1055	1172.333	127	148	104	126.3333
gudD	24443	17684	-21067.7	-10.038	7.66E-08	-4.27391	-0.00139	0.00034	2.98E-07	25415	20274	24507	23398.67	2590	972	3431	2331
gudP	26409	21117	-24401.3	-98.8663	0	-41.7822	-0.00187	0	0	26540	21422	25990	24650.67	312	131	305	249.3333
hemC	5182	4558	-4700.33	-5.81756	2.49E-06	-2.48111	-0.00026	0.011043	7.93E-06	5952	5424	5652	5676	866	770	1291	975.6667
hemD	2165	1744	-1923	-6.15089	1.52E-09	-2.60264	-0.00011	6.72E-06	7.41E-09	2489	2116	2284	2296.333	324	424	372	373.3333
hemH	3004	911	1870	5.004283	7.76E-25	11.71175	0.000389	3.44E-21	1.27E-23	507	431	463	467	2202	1374	3435	2337
hemX	3746	3099	-3302	-6.03098	1.02E-07	-2.55056	-0.00019	0.000452	3.89E-07	4328	3738	3809	3958.333	582	748	639	656.3333
hepA	288	150	19.33333	1.018176	7.86E-08	2.415237	0.000117	0.000348	3.05E-07	1134	945	1112	1063.667	1054	1233	962	1083
hflB	13796	5977	9027.333	1.674554	2.22E-12	3.943169	0.003065	9.83E-09	1.47E-11	14184	12677	13287	13382.67	21493	19264	26473	22410
hilA	25	5	7.666667	1.156463	2.98E-09	2.722712	6.6E-06	1.32E-05	1.4E-08	52	48	47	49	45	70	55	56.66667
hilC	414	350	-377	-7.31844	2.61E-12	-3.12049	-2.3E-05	1.16E-08	1.72E-11	406	457	447	436.6667	56	43	80	59.66667
hisS	4130	3423	-3749	-6.36338	3.87E-08	-2.68957	-0.00022	0.000172	1.58E-07	4764	4093	4487	4448	634	793	670	699
hmpA	1125	771	889.6667	6.045369	5.15E-64	14.2191	0.000181	2.28E-60	2.51E-62	200	169	160	176.3333	973	940	1285	1066
hnr	1300	1196	-1213.33	-5.03548	3.7E-07	-2.14217	-6.3E-05	0.001637	1.32E-06	1558	1473	1511	1514	277	258	367	300.6667
hns	28085	25433	-26394	-8.61219	1.15E-11	-3.64982	-0.00169	5.09E-08	7.04E-11	31167	29502	28915	29861.33	3082	3838	3482	3467.333
hopD	277	51	165.6667	3.070833	4.75E-21	7.203935	3.87E-05	2.1E-17	6.15E-20	87	68	85	80	256	136	345	245.6667
hpaE	247	109	-44	-1.14798	0.000153	2.070745	2.84E-05	0.679149	0.000377	360	306	358	341.3333	249	445	198	297.3333
hpaG	140	55	90.33333	1.918644	2.76E-23	4.50366	2.69E-05	1.22E-19	4.07E-22	102	100	93	98.33333	178	155	233	188.6667
hpaR	80	66	-39.6667	-1.13967	1.04E-07	2.06276	2.68E-05	0.000459	3.94E-07	338	337	296	323.6667	258	264	330	284
hpt	489	444	-453	-6.47984	7.97E-12	-2.7457	-2.7E-05	3.53E-08	4.95E-11	556	525	526	535.6667	67	100	81	82.66667

Feature ID	Experiment - Range (original values)	Experiment - IQR (original values)	Experiment - Difference (original values)	Experiment - Fold Change (original values)	EDGE test: yccT H202 vs yccT NT , tagwise dispersions - P-value	EDGE test: yccT H202 vs yccT NT , tagwise dispersions - Fold change	EDGE test: yccT H202 vs yccT NT , tagwise dispersions - Weighted difference	EDGE test: yccT H202 vs yccT NT , tagwise dispersions - Bonferroni	EDGE test: yccT H202 vs yccT NT , tagwise dispersions - FDR p-value correction	yccT NT - yccT.1.S28 - Expression values	yccT NT - yccT.2.S29 - Expression values	yccT NT - yccT.3.S30 - Expression values	yccT NT - Means	yccT H202 - yccT.1.H2O2 - Expression values	yccT H202 - yccT.2.H2O2 - Expression values	yccT H202 - yccT.3.H2O2 - Expression values	yccT H202 - Means
hrpA	1184	209	-104	-1.03899	2.13E-05	2.268724	0.000274	0.09457	5.97E-05	2925	2592	2798	2771.667	2801	2009	3193	2667.667
hscA	3153	49	1173.333	1.96863	5.77E-08	4.688105	0.000348	0.000256	2.3E-07	1284	1099	1251	1211.333	1667	4252	1235	2384.667
hscB	4586	519	2063.667	4.003882	2.69E-12	9.54363	0.000457	1.19E-08	1.77E-11	720	609	732	687	1818	5195	1239	2750.667
hscC	825	741	-780.667	-11.3628	1.9E-23	-4.78232	-5.3E-05	8.43E-20	2.86E-22	884	820	864	856	88	79	59	75.33333
hsdM	725	525	-639	-4.85714	2.3E-07	-2.05525	-3.2E-05	0.00102	8.47E-07	851	691	872	804.6667	166	147	184	165.6667
hslU	1657	104	871.6667	1.718999	6.66E-10	4.037672	0.000287	2.95E-06	3.36E-09	1272	1197	1168	1212.333	2289	1153	2810	2084
hslV	922	60	281.3333	1.565684	2.62E-07	3.656253	0.000103	0.00116	9.57E-07	530	492	470	497.3333	802	306	1228	778.6667
htpG	5443	1985	3104.333	3.87261	1.79E-18	9.146742	0.000688	7.93E-15	1.99E-17	1040	1183	1019	1080.667	3068	6462	3025	4185
htrB	1163	997	-1042.33	-8.02697	3.29E-12	-3.41103	-6.6E-05	1.46E-08	2.14E-11	1253	1158	1161	1190.667	161	90	194	148.3333
hutH	2978	2170	-2654.67	-6.78359	3.12E-10	-2.87294	-0.00016	1.38E-06	1.63E-09	3326	2618	3397	3113.667	419	448	510	459
hyaE	213	175	-177.667	-4.80714	0.000752	-2.02992	-8.9E-06	1	0.001654	222	207	244	224.3333	31	77	32	46.66667
hyaF	492	396	-435	-6.1992	2.6E-08	-2.618	-2.5E-05	0.000115	1.08E-07	536	464	556	518.6667	64	119	68	83.66667
hybA	6171	5152	-5678.33	-10.5434	1.15E-14	-4.44166	-0.00038	5.12E-11	9.39E-14	6687	5729	6404	6273.333	692	516	577	595
hybB	6503	5215	-5830.33	-10.3136	9.24E-14	-4.34633	-0.00039	4.09E-10	6.96E-13	7001	5853	6515	6456.333	742	498	638	626
hybF	872	645	-763.333	-5.20183	2.13E-07	-2.19135	-4E-05	0.000945	7.89E-07	1017	827	991	945	218	145	182	181.6667
hybG	906	666	-773	-5.21636	2.95E-06	-2.19612	-4.1E-05	0.013075	9.3E-06	1031	854	984	956.3333	237	125	188	183.3333
hycC	43	5	11	1.165	1.58E-09	2.753864	9.11E-06	7E-06	7.68E-09	76	57	67	66.66667	64	100	69	77.66667
hycD	33	14	17.33333	1.684211	1.97E-12	3.973979	5.89E-06	8.74E-09	1.32E-11	34	19	23	25.33333	39	52	37	42.66667
hycE	22	5	5.666667	1.061372	1.53E-10	2.509217	1.09E-05	6.77E-07	8.29E-10	100	81	96	92.33333	103	93	98	98
hycF	18	8	1	1.035714	1.21E-05	2.434816	3.14E-06	0.053561	3.52E-05	30	19	35	28	28	22	37	29
hycG	20	7	-0.66667	-1.0146	2.43E-06	2.333751	4.82E-06	0.010766	7.76E-06	56	43	40	46.33333	47	54	36	45.66667
hycH	27	4	3	1.085714	8.66E-06	2.566713	4.29E-06	0.03837	2.56E-05	35	31	39	35	35	53	26	38
hydG	2773	2461	-2556.67	-5.53578	3.02E-07	-2.3448	-0.00014	0.001338	1.1E-06	3294	3025	3042	3120.333	564	521	606	563.6667
hydH	2126	1869	-1942.67	-5.58537	4.46E-07	-2.37223	-0.00011	0.001975	1.57E-06	2437	2345	2317	2366.333	448	311	512	423.6667
hydN	27	13	-4.66667	-1.13084	0.000547	2.086804	3.42E-06	1	0.001232	43	33	45	40.33333	25	52	30	35.66667
hypA	1411	1250	-1321.67	-42.7368	3.39E-79	-18.0516	-1E-04	1.5E-75	2.18E-77	1342	1436	1282	1353.333	32	38	25	31.66667
hypB	10591	9857	-10153.7	-12.1375	0	-5.12723	-0.00069	0	0	11436	10756	11004	11065.33	991	845	899	911.6667
hypC	2684	2499	-2567	-9.4256	1.89E-14	-3.96188	-0.00017	8.36E-11	1.51E-13	2921	2817	2877	2871.667	359	318	237	304.6667
hypD	8971	7929	-8503.33	-11.8646	6.66E-16	-4.99239	-0.00058	2.95E-12	6.04E-15	9492	8737	9629	9286	882	808	658	782.6667
hypO	15119	13986	-14469.7	-19.2238	0	-8.17619	-0.00104	0	0	15750	14758	15283	15263.67	772	631	979	794

Feature ID	Experiment - Range (original values)	Experiment - IQR (original values)	Experiment - Difference (original values)	Experiment - Fold Change (original values)	EDGE test: yccT H202 vs yccT NT , tagwise dispersions - P-value	EDGE test: yccT H202 vs yccT NT , tagwise dispersions - Fold change	EDGE test: yccT H202 vs yccT NT , tagwise dispersions - Weighted difference	EDGE test: yccT H202 vs yccT NT , tagwise dispersions - Bonferroni	EDGE test: yccT H202 vs yccT NT , tagwise dispersions - FDR p-value correction	yccT NT - yccT.1.S28 - Expression values	yccT NT - yccT.2.S29 - Expression values	yccT NT - yccT.3.S30 - Expression values	yccT NT - Means	yccT H202 - yccT.1.H2O2 - Expression values	yccT H202 - yccT.2.H2O2 - Expression values	yccT H202 - yccT.3.H2O2 - Expression values	yccT H202 - Means
iacP	13	5	8	1.923077	2.22E-08	4.477068	2.39E-06	9.84E-05	9.34E-08	10	6	10	8.666667	19	15	16	16.66667
iagB	83	58	63	5.295455	1.36E-34	12.3762	1.32E-05	6.01E-31	3.45E-33	11	11	22	14.66667	69	94	70	77.66667
ibpA	20515	12324	16307.33	29.79459	1.36E-75	70.59932	0.003078	6E-72	7.8E-74	550	601	548	566.3333	16684	21063	12874	16873.67
ibpB	12251	8625	10984.67	41.23687	7.48E-85	97.68637	0.002064	3.31E-81	5.34E-83	249	294	276	273	12500	12372	8901	11257.67
iclR	1332	1102	-1188.67	-9.51074	5.25E-21	-4.02263	-7.8E-05	2.33E-17	6.78E-20	1452	1239	1294	1328.333	120	162	137	139.6667
idnD	577	474	-523.667	-7.54583	5.95E-15	-3.18867	-3.2E-05	2.64E-11	4.92E-14	640	560	611	603.6667	91	63	86	80
idnR	1252	1153	-1185.67	-6.94816	2.64E-14	-2.95136	-7.1E-05	1.17E-10	2.09E-13	1434	1337	1384	1385	182	184	232	199.3333
idnT	1392	1151	-1256	-10.7113	8.38E-13	-4.55711	-8.4E-05	3.71E-09	5.85E-12	1452	1296	1408	1385.333	145	60	183	129.3333
ilvB	7958	6143	-6903	-17.3578	0	-7.40032	-0.00049	0	0	8235	7197	6543	7325	400	277	589	422
ilvC	898	465	614.6667	1.495034	1.51E-12	3.550289	0.000246	6.68E-09	1.02E-11	1370	1075	1280	1241.667	1973	1851	1745	1856.333
ilvN	1099	819	-939	-9.07163	1.72E-20	-3.83908	-6.1E-05	7.6E-17	2.16E-19	1201	1029	936	1055.333	117	102	130	116.3333
infB	3005	1066	-857.333	-1.14361	0.000324	2.081612	0.000574	1	0.000755	7597	6032	6852	6827	5786	7531	4592	5969.667
invB	21	4	7	1.355932	3.29E-07	3.170714	3.36E-06	0.001457	1.19E-06	15	23	21	19.66667	19	36	25	26.66667
invC	17	5	-3.66667	-1.07333	8.91E-07	2.201483	5.03E-06	0.003946	3.03E-06	58	43	60	53.66667	51	52	47	50
invE	12	3	7.666667	1.178295	3.61E-10	2.778895	5.98E-06	1.6E-06	1.88E-09	45	43	41	43	53	53	46	50.66667
invH	40	8	15	1.343511	9.17E-11	3.137654	7.32E-06	4.07E-07	5.06E-10	35	52	44	43.66667	49	52	75	58.66667
invI	14	8	9.333333	2.866667	1.55E-09	6.594395	2.25E-06	6.88E-06	7.57E-09	9	2	4	5	15	16	12	14.33333
invJ	11	5	6.333333	1.204301	2.44E-09	2.829712	4.44E-06	1.08E-05	1.15E-08	31	33	29	31	36	36	40	37.33333
ipk	1486	1261	-1390.67	-8	2.22E-16	-3.38679	-8.7E-05	9.84E-13	2.11E-15	1653	1458	1657	1589.333	171	228	197	198.6667
iroB	5386	2252	3449	32.54573	4.15E-53	77.19256	0.00065	1.84E-49	1.78E-51	107	95	126	109.3333	2835	5481	2359	3558.333
iroC	9014	1401	4244.667	13.13918	8.26E-19	31.30125	0.000825	3.66E-15	9.33E-18	350	311	388	349.6667	2707	9325	1751	4594.333
iroD	648	85	290.3333	3.756329	1.04E-13	8.943861	6.51E-05	4.62E-10	7.81E-13	123	101	92	105.3333	261	740	186	395.6667
iroE	1469	436	833	10.83858	3.78E-31	25.68768	0.000163	1.67E-27	8.2E-30	80	82	92	84.66667	686	1549	518	917.6667
iroN	10681	5451	7237.333	40.19134	4.84E-70	95.01436	0.001355	2.15E-66	2.62E-68	184	186	184	184.6667	5766	10865	5635	7422
ispD	752	583	-668	-6.19171	1.26E-11	-2.6092	-3.8E-05	5.6E-08	7.71E-11	866	715	809	796.6667	132	140	114	128.6667
ispF	382	330	-357.667	-5.43388	2.2E-09	-2.30136	-1.9E-05	9.73E-06	1.05E-08	449	412	454	438.3333	72	88	82	80.66667
ivbL	754	675	-679	-10.7933	1.74E-09	-4.66355	-4.6E-05	7.72E-06	8.41E-09	724	739	782	748.3333	49	28	131	69.33333
katG	348615	264719	295089.3	11.07245	5.56E-47	26.24985	0.057518	2.46E-43	2.22E-45	32602	27641	27647	29296.67	304536	376256	292366	324386
kdgT	775	35	311	2.927686	4.24E-09	7.06443	7.6E-05	1.88E-05	1.94E-08	185	136	163	161.3333	911	150	356	472.3333
kdpB	42	12	-13.6667	-1.14286	6.2E-06	2.074862	9.16E-06	0.027451	1.86E-05	116	103	109	109.3333	97	116	74	95.66667

Feature ID	Experiment - Range (original values)	Experiment - IQR (original values)	Experiment - Difference (original values)	Experiment - Fold Change (original values)	EDGE test: yccT H202 vs yccT NT , tagwise dispersions - P-value	EDGE test: yccT H202 vs yccT NT , tagwise dispersions - Fold change	EDGE test: yccT H202 vs yccT NT , tagwise dispersions - Weighted difference	EDGE test: yccT H202 vs yccT NT , tagwise dispersions - Bonferroni	EDGE test: yccT H202 vs yccT NT , tagwise dispersions - FDR p-value correction	yccT NT - yccT.1.S28 - Expression values	yccT NT - yccT.2.S29 - Expression values	yccT NT - yccT.3.S30 - Expression values	yccT NT - Means	yccT H202 - yccT.1.H2O2 - Expression values	yccT H202 - yccT.2.H2O2 - Expression values	yccT H202 - yccT.3.H2O2 - Expression values	yccT H202 - Means
kdpC	35	9	2.666667	1.053333	3.6E-06	2.471229	5.76E-06	0.015968	1.12E-05	49	59	42	50	36	71	51	52.66667
kdsB	171	42	-75.6667	-1.08417	3.66E-07	2.179774	8.95E-05	0.001623	1.32E-06	1016	900	1008	974.6667	910	845	942	899
kdtA	1758	1400	-1545.33	-6.88325	2.12E-12	-2.91272	-9.2E-05	9.38E-09	1.41E-11	1982	1671	1771	1808	271	224	293	262.6667
kefB	265	75	53.33333	1.074977	2.31E-09	2.536842	8.52E-05	1.03E-05	1.1E-08	757	711	666	711.3333	644	909	741	764.6667
kgtP	418	78	36.66667	1.102041	1.54E-05	2.621508	4.53E-05	0.068072	4.4E-05	378	319	381	359.3333	300	653	235	396
ldhA	1763	116	786.3333	2.077661	1.57E-10	4.940542	0.000224	6.94E-07	8.48E-10	754	740	695	729.6667	1234	2458	856	1516
leuB	1444	1021	-1205	-5.47401	2.54E-08	-2.32105	-6.5E-05	0.000113	1.06E-07	1665	1450	1308	1474.333	221	300	287	269.3333
leuL	32	16	-23.6667	-7.45455	0.000458	-3.11394	-1.5E-06	1	0.001045	33	28	21	27.33333	5	1	5	3.666667
lexA	12242	2703	7473.667	3.179759	1.02E-16	7.454579	0.001723	4.53E-13	9.99E-16	3737	3473	3076	3428.667	11213	6176	15318	10902.33
livF	52	20	-5.33333	-1.05882	4.84E-06	2.236997	9.25E-06	0.021451	1.47E-05	106	86	96	96	76	124	72	90.66667
livG	41	23	12.66667	1.128814	4.69E-10	2.679324	1.29E-05	2.08E-06	2.41E-09	114	87	94	98.33333	114	128	91	111
livH	34	4	19.33333	1.215613	5.78E-12	2.864454	1.31E-05	2.56E-08	3.64E-11	87	93	89	89.66667	121	89	117	109
livK	178	66	111.3333	1.874346	5.56E-22	4.412086	3.39E-05	2.46E-18	7.55E-21	146	136	100	127.3333	236	202	278	238.6667
livM	27	8	-3.66667	-1.02689	9.13E-10	2.304446	1.42E-05	4.05E-06	4.55E-09	152	125	143	140	139	139	131	136.3333
lldR	159	91	-129	-5.11702	3.33E-06	-2.15967	-6.7E-06	0.014741	1.04E-05	184	123	174	160.3333	32	25	37	31.33333
lolB	904	811	-839.333	-7.4399	4.08E-16	-3.15629	-5.2E-05	1.81E-12	3.78E-15	1014	940	955	969.6667	129	110	152	130.3333
lonH	1138	1009	-1057.67	-7.58299	6.92E-13	-3.20583	-6.5E-05	3.07E-09	4.85E-12	1267	1148	1240	1218.333	129	214	139	160.6667
lpfA	14	10	-7.66667	-1.12921	6.65E-07	2.085977	5.68E-06	0.002948	2.3E-06	69	62	70	67	56	56	66	59.33333
lpfC	54	26	-22.3333	-1.12204	5.37E-08	2.105543	1.77E-05	0.000238	2.15E-07	225	171	220	205.3333	174	175	200	183
lplA	379	12	-31	-1.04718	1.86E-06	2.243902	6.68E-05	0.008232	6.02E-06	680	692	692	688	700	446	825	657
lpp	177555	164936	166982.7	2.001638	3E-15	4.736656	0.048496	1.33E-11	2.55E-14	176700	161692	161737	166709.7	339247	335157	326673	333692.3
lpxC	6720	3897	-3665.33	-1.14143	0.000131	2.070723	0.002467	0.581005	0.000326	30853	28829	29060	29580.67	24133	28681	24932	25915.33
lpxD	5289	4683	-4962.67	-5.65832	2.82E-06	-2.39395	-0.00027	0.01248	8.91E-06	6233	5692	6159	6028	944	1243	1009	1065.333
lpxH	494	414	-459.333	-8.78531	1.7E-19	-3.70813	-2.9E-05	7.54E-16	2.01E-18	549	475	531	518.3333	61	61	55	59
ltaA	1151	964	-1017.67	-4.83543	9.24E-07	-2.0593	-5.1E-05	0.004095	3.13E-06	1366	1211	1272	1283	215	247	334	265.3333
luxS	3330	1100	1763.667	1.548404	1.99E-10	3.667828	0.000668	8.81E-07	1.06E-09	3444	3102	3102	3216	4305	6432	4202	4979.667
lysA	1581	446	813.6667	1.591041	3.78E-11	3.766057	0.000297	1.67E-07	2.17E-10	1383	1292	1455	1376.667	1869	2873	1829	2190.333
lysP	1812	1469	-1593.33	-5.77046	6.53E-09	-2.447	-8.9E-05	2.9E-05	2.92E-08	2093	1798	1891	1927.333	329	281	392	334
lysR	164	119	-59.6667	-1.14378	1.23E-06	2.071462	3.96E-05	0.005438	4.09E-06	498	434	492	474.6667	373	518	354	415
lyxK	65	10	30	1.592105	1.22E-12	3.755237	1.09E-05	5.39E-09	8.3E-12	55	46	51	50.66667	61	111	70	80.66667

Feature ID	Experiment - Range (original values)	Experiment - IQR (original values)	Experiment - Difference (original values)	Experiment - Fold Change (original values)	EDGE test: yccT H202 vs yccT NT , tagwise dispersions - P-value	EDGE test: yccT H202 vs yccT NT , tagwise dispersions - Fold change	EDGE test: yccT H202 vs yccT NT , tagwise dispersions - Weighted difference	EDGE test: yccT H202 vs yccT NT , tagwise dispersions - Bonferroni	EDGE test: yccT H202 vs yccT NT , tagwise dispersions - FDR p-value correction	yccT NT - yccT.1.S28 - Expression values	yccT NT - yccT.2.S29 - Expression values	yccT NT - yccT.3.S30 - Expression values	yccT NT - Means	yccT H202 - yccT.1.H2O2 - Expression values	yccT H202 - yccT.2.H2O2 - Expression values	yccT H202 - yccT.3.H2O2 - Expression values	yccT H202 - Means
malF	24120	21657	-22296.7	-15.5666	5.93E-14	-6.53426	-0.00157	2.63E-10	4.56E-13	25015	23361	23106	23827.33	2248	895	1449	1530.667
malK	26284	24936	-24128.7	-5.27662	0.00085	-2.20785	-0.00127	1	0.00185	30186	29426	29700	29770.67	8534	3902	4490	5642
malT	13883	12225	-12561.3	-7.14747	4.95E-06	-3.05136	-0.00077	0.021914	1.5E-05	14894	14405	14515	14604.67	2180	1011	2939	2043.333
malY	20	2	3.333333	1.153846	2.06E-05	2.71836	2.92E-06	0.091225	5.8E-05	22	20	23	21.66667	21	37	17	25
manC	60	19	0	-1	0.000253	2.367239	6.41E-06	1	0.000599	65	65	50	60	46	97	37	60
manX	18134	1546	4361.667	1.353076	1.2E-05	3.232818	0.002146	0.053023	3.48E-05	13098	11552	12410	12353.33	15385	26447	8313	16715
manY	15765	1500	6311	1.721395	1.54E-07	4.120391	0.002123	0.000682	5.76E-07	9428	7928	8889	8748.333	14809	23067	7302	15059.33
manZ	32040	7334	20065.33	2.914387	7.39E-15	6.968306	0.004864	3.28E-11	6.08E-14	11355	9455	10634	10481.33	32177	41495	17968	30546.67
marR	224	174	-187.667	-5.29771	7.71E-07	-2.25475	-1E-05	0.003418	2.65E-06	255	226	213	231.3333	31	39	61	43.66667
mazG	172	66	-63.3333	-1.09401	2.21E-07	2.154007	6.63E-05	0.00098	8.15E-07	743	710	758	737	677	586	758	673.6667
mdaA	261	227	-240	-5.26036	1.09E-08	-2.23226	-1.3E-05	4.83E-05	4.76E-08	298	280	311	296.3333	50	53	66	56.33333
mdlA	395	239	317.3333	1.721212	5.39E-21	4.058011	0.000105	2.39E-17	6.95E-20	458	442	420	440	681	815	776	757.3333
mdlB	81	26	9.666667	1.031216	1.07E-10	2.443713	3.48E-05	4.74E-07	5.85E-10	336	273	320	309.6667	310	354	294	319.3333
mdoB	3158	2762	-2858	-6.27956	2.83E-08	-2.66721	-0.00017	0.000125	1.17E-07	3592	3305	3301	3399.333	539	434	651	541.3333
mdtI	572	394	-477.667	-6.0636	5.66E-11	-2.55426	-2.7E-05	2.51E-07	3.19E-10	660	488	568	572	94	101	88	94.33333
mdtJ	1139	1023	-1087.33	-28.4118	4.85E-43	-12.112	-8.1E-05	2.15E-39	1.75E-41	1164	1055	1162	1127	32	25	62	39.66667
melA	15406	13432	-14211	-14.9552	1.51E-13	-6.35805	-0.001	6.7E-10	1.12E-12	15911	15151	14626	15229.33	1194	505	1356	1018.333
melB	9408	7643	-8385.33	-12.419	1.2E-13	-5.26932	-0.00058	5.3E-10	8.93E-13	9851	9025	8483	9119.667	840	443	920	734.3333
menC	750	273	70	1.037709	3.32E-06	2.477282	0.000213	0.01472	1.04E-05	2104	1596	1869	1856.333	2331	1867	1581	1926.333
menD	4875	4393	-4557	-11.1568	4.44E-16	-4.70861	-0.00031	1.97E-12	4.09E-15	5264	4814	4939	5005.667	421	536	389	448.6667
metE	587	213	371.3333	2.048964	1.82E-22	4.836489	0.000105	8.04E-19	2.54E-21	408	294	360	354	722	573	881	725.3333
metF	152	50	42.66667	1.240602	2.65E-10	2.922851	2.65E-05	1.18E-06	1.39E-09	191	132	209	177.3333	217	159	284	220
metK	498	103	255.6667	1.377461	1.83E-12	3.270107	0.00012	8.11E-09	1.23E-11	737	656	639	677.3333	1137	759	903	933
metR	17	2	8	1.148148	5.44E-11	2.703013	7.18E-06	2.41E-07	3.07E-10	57	50	55	54	55	64	67	62
mglA	1056	876	-948.667	-10.6475	1.77E-25	-4.51076	-6.4E-05	7.85E-22	3.01E-24	1019	975	1147	1047	99	91	105	98.33333
mglB	3685	2527	-3069.67	-6.42983	0.000123	-2.71174	-0.00018	0.542944	0.000306	3912	3207	3786	3635	789	227	680	565.3333
mgtB	160	49	-24.3333	-1.07926	8.83E-07	2.200239	3.09E-05	0.003912	3.01E-06	363	301	330	331.3333	281	400	240	307
mgtC	67	37	45.66667	2.611765	1.75E-21	6.154881	1.14E-05	7.77E-18	2.35E-20	25	21	39	28.33333	72	88	62	74
mig-3A	123	43	72.33333	2.307229	1.07E-19	5.467263	1.93E-05	4.74E-16	1.29E-18	57	41	68	55.33333	119	164	100	127.6667
minC	2713	2532	-2608.33	-11.6318	0	-4.95213	-0.00018	0	0	2884	2910	2767	2853.667	235	197	304	245.3333

Feature ID	Experiment - Range (original values)	Experiment - IQR (original values)	Experiment - Difference (original values)	Experiment - Fold Change (original values)	EDGE test: yccT H202 vs yccT NT , tagwise dispersions - P-value	EDGE test: yccT H202 vs yccT NT , tagwise dispersions - Fold change	EDGE test: yccT H202 vs yccT NT , tagwise dispersions - Weighted difference	EDGE test: yccT H202 vs yccT NT , tagwise dispersions - Bonferroni	EDGE test: yccT H202 vs yccT NT , tagwise dispersions - FDR p-value correction	yccT NT - yccT.1.S28 - Expression values	yccT NT - yccT.2.S29 - Expression values	yccT NT - yccT.3.S30 - Expression values	yccT NT - Means	yccT H202 - yccT.1.H2O2 - Expression values	yccT H202 - yccT.2.H2O2 - Expression values	yccT H202 - yccT.3.H2O2 - Expression values	yccT H202 - Means
mioC	1200	973	-1044.67	-7.07364	5.93E-10	-3.01542	-6.3E-05	2.63E-06	3.01E-09	1307	1137	1206	1216.667	164	107	245	172
mlc	920	795	-842.667	-5.49023	2.51E-09	-2.33634	-4.6E-05	1.11E-05	1.18E-08	1074	974	1043	1030.333	154	179	230	187.6667
mltC	746	571	-642	-6.14973	5.43E-11	-2.60868	-3.7E-05	2.41E-07	3.07E-10	756	699	845	766.6667	99	147	128	124.6667
mntH	17953	6988	12400.33	48.26938	2.12E-69	113.1734	0.002288	9.39E-66	1.1E-67	279	221	287	262.3333	12547	7267	18174	12662.67
moaA	5649	5008	-5382.33	-16.1473	0	-6.84368	-0.00038	0	0	5977	5365	5871	5737.667	328	357	381	355.3333
mobA	928	883	-872	-6.04046	2.94E-09	-2.58581	-5E-05	1.3E-05	1.38E-08	1028	1060	1047	1045	132	145	242	173
mobB	783	723	-736.333	-5.39165	7.26E-09	-2.29798	-4E-05	3.22E-05	3.21E-08	919	877	916	904	136	154	213	167.6667
mopA	39374	3757	12775.67	1.658709	1.93E-06	3.93742	0.00445	0.008563	6.25E-06	19004	21469	17712	19395	24126	55880	16506	32170.67
mopB	4576	166	733	1.280986	0.000196	3.043567	0.000417	0.867727	0.000474	2533	2926	2367	2608.667	2471	6065	1489	3341.667
mppA	5920	5543	-5699	-7.12357	4.28E-09	-3.01261	-0.00035	1.9E-05	1.96E-08	6654	6772	6463	6629.667	1020	852	920	930.6667
mscL	881	57	272.6667	1.500306	6.32E-08	3.566909	0.000109	0.00028	2.49E-07	570	513	552	545	614	1360	479	817.6667
msgA	173	30	52.33333	1.273043	1.41E-10	2.983058	2.96E-05	6.24E-07	7.67E-10	204	174	197	191.6667	221	169	342	244
msrA	2581	614	1341.333	2.564541	4.23E-14	6.080488	0.00034	1.87E-10	3.28E-13	900	887	785	857.3333	1729	3366	1501	2198.667
mutM	373	210	297.6667	2.531732	5.07E-30	6.018617	7.59E-05	2.25E-26	1.05E-28	210	179	194	194.3333	552	520	404	492
mutY	528	469	-502	-4.89147	7.05E-08	-2.06842	-2.5E-05	0.000313	2.76E-07	641	598	654	631	132	129	126	129
mviM	3481	2975	-3215.33	-9.48373	8.88E-16	-4.00446	-0.00021	3.94E-12	7.93E-15	3848	3358	3577	3594.333	383	387	367	379
nadD	426	330	-384.333	-4.89527	7.11E-07	-2.06236	-1.9E-05	0.003151	2.45E-06	510	436	503	483	106	106	84	98.66667
nagA	8508	1836	5186	3.869421	4.4E-18	9.05698	0.001134	1.95E-14	4.67E-17	1879	1733	1810	1807.333	7093	3646	10241	6993.333
nagB	21897	14239	17188.33	12.36294	4.81E-60	29.00074	0.003302	2.13E-56	2.2E-58	1545	1526	1467	1512.667	16974	15765	23364	18701
nagC	2051	200	942.6667	1.538359	2.38E-09	3.607678	0.000355	1.06E-05	1.13E-08	1887	1679	1687	1751	2639	1712	3730	2693.667
nagE	24691	965	12642.33	2.666828	2.92E-09	6.240769	0.003095	1.29E-05	1.37E-08	8118	7483	7153	7584.667	22666	6662	31353	20227
nanE	3529	2764	-3145.67	-12.2748	1.47E-14	-5.19992	-0.00022	6.49E-11	1.18E-13	3688	3484	3102	3424.667	340	159	338	279
nanK	2337	1788	-1988.33	-8.10119	5.23E-10	-3.41961	-0.00012	2.32E-06	2.67E-09	2503	2198	2104	2268.333	358	166	316	280
napA	1224	133	329.3333	1.400162	1.88E-06	3.352044	0.000151	0.008336	6.09E-06	832	752	885	823	1201	1740	516	1152.333
napB	170	23	88.33333	2.090535	3.95E-10	5.016229	2.53E-05	1.75E-06	2.04E-09	85	65	93	81	203	235	70	169.3333
napC	176	13	47.66667	1.386486	2.01E-07	3.308817	2.22E-05	0.000893	7.46E-07	131	118	121	123.3333	169	260	84	171
napG	201	15	95	2.055556	5.56E-11	4.911231	2.74E-05	2.47E-07	3.14E-10	101	83	86	90	183	284	88	185
napH	163	36	57	1.564356	3.86E-08	3.733729	2.15E-05	0.000171	1.58E-07	97	85	121	101	159	239	76	158
narG	1160	221	-222.333	-1.1815	0.002564	2.022845	0.000115	1	0.005086	1544	1356	1442	1447.333	1221	1807	647	1225
narH	1034	36	65.66667	1.103903	0.001285	2.648314	8.1E-05	1	0.002697	704	578	614	632	613	1257	223	697.6667

Feature ID	Experiment - Range (original values)	Experiment - IQR (original values)	Experiment - Difference (original values)	Experiment - Fold Change (original values)	EDGE test: yccT H202 vs yccT NT , tagwise dispersions - P-value	EDGE test: yccT H202 vs yccT NT , tagwise dispersions - Fold change	EDGE test: yccT H202 vs yccT NT , tagwise dispersions - Weighted difference	EDGE test: yccT H202 vs yccT NT , tagwise dispersions - Bonferroni	EDGE test: yccT H202 vs yccT NT , tagwise dispersions - FDR p-value correction	yccT NT - yccT.1.S28 - Expression values	yccT NT - yccT.2.S29 - Expression values	yccT NT - yccT.3.S30 - Expression values	yccT NT - Means	yccT H202 - yccT.1.H2O2 - Expression values	yccT H202 - yccT.2.H2O2 - Expression values	yccT H202 - yccT.3.H2O2 - Expression values	yccT H202 - Means
narI	1594	83	667	3.163243	1.29E-08	7.594921	0.000158	5.7E-05	5.55E-08	354	300	271	308.3333	846	1837	243	975.3333
narJ	543	26	29.66667	1.099776	0.002861	2.634531	3.78E-05	1	0.005599	324	288	280	297.3333	262	631	88	327
narK	95	13	31	1.624161	2.4E-07	3.820503	1.1E-05	0.001065	8.82E-07	44	65	40	49.66667	57	135	50	80.66667
ndh	10552	7445	9066.333	11.67046	5.65E-60	27.47209	0.001754	2.51E-56	2.56E-58	879	890	780	849.6667	10092	8324	11332	9916
ndk	941	772	-859.333	-5.77407	1.7E-09	-2.44078	-4.8E-05	7.53E-06	8.21E-09	1095	939	1084	1039.333	154	219	167	180
nemA	10202	2252	5157	6.148419	1.13E-17	14.64867	0.001063	4.99E-14	1.17E-16	1124	910	971	1001.667	4141	11112	3223	6158.667
nifJ	5199	4655	4894.333	7.735321	4.62E-45	18.25871	0.000977	2.05E-41	1.75E-43	727	683	770	726.6667	5599	5382	5882	5621
nifS	6196	4254	4961.667	2.626598	1.07E-20	6.213358	0.001237	4.75E-17	1.36E-19	3336	2838	2977	3050.333	7231	9034	7771	8012
nifU	10714	3374	6004.333	5.353069	1.53E-20	12.70119	0.001257	6.79E-17	1.93E-19	1447	1368	1323	1379.333	5372	12037	4742	7383.667
nirB	412	122	-16.3333	-1.0528	0.001596	2.260692	3.2E-05	1	0.003281	346	301	330	325.6667	208	566	154	309.3333
nirD	206	22	32	1.328767	0.000177	3.165358	1.64E-05	0.783203	0.000431	104	86	102	97.33333	80	257	51	129.3333
nlpC	263	199	-234.667	-5.34568	1.3E-08	-2.26368	-1.3E-05	5.77E-05	5.61E-08	301	255	310	288.6667	47	56	59	54
nlpD	49277	14952	28392.33	3.070972	2.71E-16	7.258122	0.006675	1.2E-12	2.56E-15	14718	12855	13556	13709.67	28508	62132	35666	42102
nrdD	2387	2133	-2217.33	-5.47043	3.95E-07	-2.3083	-0.00012	0.001749	1.4E-06	2842	2643	2655	2713.333	523	510	455	496
nrdE	54	28	42	1.133758	2.02E-13	2.674677	4.1E-05	8.95E-10	1.48E-12	312	316	314	314	360	342	366	356
nrdF	126	45	75	1.618132	4.15E-18	3.807981	2.66E-05	1.84E-14	4.44E-17	111	130	123	121.3333	168	237	184	196.3333
nrdH	51	31	41.33333	42.33333	1.64E-36	85.09676	7.69E-06	7.26E-33	4.51E-35	3	0	0	1	31	51	45	42.33333
nrdI	58	41	46.66667	8.777778	1.42E-39	20.18469	9.22E-06	6.28E-36	4.66E-38	8	7	3	6	48	61	49	52.66667
nrfA	3480	2958	-3161	-13.3799	0	-5.60909	-0.00022	0	0	3358	3238	3653	3416.333	313	280	173	255.3333
nrfB	809	712	-772	-8.98621	2.62E-21	-3.79546	-5E-05	1.16E-17	3.44E-20	896	809	901	868.6667	101	97	92	96.66667
nrfG	39	13	3.333333	1.042194	6.27E-08	2.464487	9.03E-06	0.000278	2.48E-07	79	71	87	79	101	62	84	82.33333
nupC	1537	187	558	1.484936	2.17E-07	3.494893	0.000223	0.000962	8.02E-07	1216	1029	1207	1150.667	1993	798	2335	1708.667
nupG	1165	975	-1037	-7.41443	1.98E-11	-3.14722	-6.4E-05	8.77E-08	1.18E-10	1267	1153	1176	1198.667	178	102	205	161.6667
nusB	646	148	-51.6667	-1.0551	2.75E-05	2.255591	9.65E-05	0.121722	7.56E-05	1103	886	979	989.3333	831	1314	668	937.6667
oat	52	7	4	1.044776	2.5E-08	2.4454	1.01E-05	0.000111	1.04E-07	91	92	85	89.33333	72	84	124	93.33333
ogt	605	569	-566	-5.05251	1.11E-07	-2.15112	-2.9E-05	0.000493	4.22E-07	717	702	698	705.6667	129	112	178	139.6667
ompA	208950	27154	58475.67	1.532881	1.34E-05	3.679907	0.02284	0.059544	3.87E-05	123437	96283	109485	109735	162760	275411	66461	168210.7
ompX	71472	3183	14181.67	1.616183	0.000502	3.855646	0.005117	1	0.00114	24396	22444	22206	23015.33	19261	81901	10429	37197
oppA	78790	58656	-65899.3	-5.59955	6.2E-05	-2.37331	-0.00361	0.274888	0.000162	88083	74668	77929	80226.67	16012	9293	17677	14327.33
oppB	10475	8332	-9225.33	-10.5336	2.72E-12	-4.46221	-0.00062	1.21E-08	1.79E-11	11106	9425	10048	10193	1093	631	1179	967.6667

Feature ID	Experiment - Range (original values)	Experiment - IQR (original values)	Experiment - Difference (original values)	Experiment - Fold Change (original values)	EDGE test: yccT H202 vs yccT NT , tagwise dispersions - P-value	EDGE test: yccT H202 vs yccT NT , tagwise dispersions - Fold change	EDGE test: yccT H202 vs yccT NT , tagwise dispersions - Weighted difference	EDGE test: yccT H202 vs yccT NT , tagwise dispersions - Bonferroni	EDGE test: yccT H202 vs yccT NT , tagwise dispersions - FDR p-value correction	yccT NT - yccT.1.S28 - Expression values	yccT NT - yccT.2.S29 - Expression values	yccT NT - yccT.3.S30 - Expression values	yccT NT - Means	yccT H202 - yccT.1.H2O2 - Expression values	yccT H202 - yccT.2.H2O2 - Expression values	yccT H202 - yccT.3.H2O2 - Expression values	yccT H202 - Means
oppC	7004	5797	-6389.33	-11.643	0	-4.93108	-0.00043	0	0	7529	6398	7042	6989.667	601	525	675	600.3333
oppD	8549	6544	-7574.67	-6.3683	9.78E-08	-2.68567	-0.00044	0.000433	3.75E-07	9902	7948	9107	8985.667	1404	1476	1353	1411
orf242	238	84	134.3333	1.206138	2.07E-12	2.852633	9.4E-05	9.17E-09	1.38E-11	673	606	676	651.6667	757	844	757	786
orf408	190	149	-169.667	-10.0893	8.17E-16	-4.2515	-1.1E-05	3.62E-12	7.32E-15	195	168	202	188.3333	25	12	19	18.66667
orfX	431	350	-386.667	-36.1515	3E-50	-15.2427	-2.9E-05	1.33E-46	1.23E-48	359	395	439	397.6667	9	16	8	11
osmC	398	138	243	2.345018	2.95E-22	5.567206	6.43E-05	1.31E-18	4.08E-21	207	184	151	180.6667	400	549	322	423.6667
osmY	837	7	235.6667	1.515682	4.95E-07	3.595202	9.23E-05	0.002193	1.73E-06	504	430	437	457	404	1241	433	692.6667
otsB	181	24	77.66667	1.809028	3.6E-12	4.262883	2.44E-05	1.6E-08	2.32E-11	93	78	117	96	108	259	154	173.6667
pagC	2082	752	1436.667	6.307882	3.24E-29	15.03537	0.000296	1.44E-25	6.5E-28	285	271	256	270.6667	1761	2338	1023	1707.333
pagD	5	3	0	-1	0.009628	2.329025	8.82E-07	1	0.016945	6	11	8	8.333333	10	6	9	8.333333
pagK	861	314	528.3333	3.897623	7.99E-27	9.229687	0.000117	3.54E-23	1.46E-25	180	191	176	182.3333	601	1037	494	710.6667
pagO	81	41	57	1.806604	3.5E-20	4.254795	1.8E-05	1.55E-16	4.32E-19	60	76	76	70.66667	141	117	125	127.6667
pagP	650	618	-629	-8.82988	4.75E-20	-3.74824	-4.1E-05	2.1E-16	5.8E-19	718	696	714	709.3333	78	68	95	80.33333
panB	552	526	-528.333	-5.3188	7.13E-08	-2.24147	-2.8E-05	0.000316	2.79E-07	654	641	657	650.6667	147	115	105	122.3333
panC	905	789	-842	-6.10303	1.27E-11	-2.57548	-4.8E-05	5.61E-08	7.71E-11	1056	959	1006	1007	174	170	151	165
panD	965	867	-914.667	-5.92639	3.61E-11	-2.5071	-5.2E-05	1.6E-07	2.08E-10	1101	1052	1148	1100.333	189	185	183	185.6667
pcm	1118	1038	-1064.67	-6.95896	1.15E-14	-2.95576	-6.4E-05	5.09E-11	9.35E-14	1279	1208	1243	1243.333	161	170	205	178.6667
pcnB	1241	1041	-1139.33	-8.52863	2.47E-18	-3.6262	-7.3E-05	1.1E-14	2.73E-17	1361	1193	1318	1290.667	120	152	182	151.3333
pdhR	3522	1542	2372.667	9.596618	1E-39	22.43986	0.000461	4.43E-36	3.33E-38	286	254	288	276	2342	1828	3776	2648.667
pduA	240	160	-204.667	-23.7407	1.21E-26	-10.0484	-1.5E-05	5.37E-23	2.2E-25	170	246	225	213.6667	10	6	11	9
pduF	526	426	-459	-12.1951	3.71E-14	-5.218	-3.2E-05	1.64E-10	2.9E-13	467	543	490	500	41	17	65	41
pduJ	87	56	62.66667	3.26506	1.26E-25	7.682049	1.45E-05	5.57E-22	2.16E-24	25	15	43	27.66667	81	102	88	90.33333
pduK	97	63	79.33333	3.288462	6.3E-34	7.755947	1.83E-05	2.79E-30	1.52E-32	38	38	28	34.66667	125	116	101	114
pduL	62	48	53.33333	1.898876	5.93E-24	4.468543	1.61E-05	2.63E-20	9.19E-23	59	63	56	59.33333	113	107	118	112.6667
pduM	35	7	16	1.761905	1.01E-10	4.150999	5.19E-06	4.46E-07	5.53E-10	28	22	13	21	34	48	29	37
pduN	14	3	6	2	3.52E-06	4.618533	1.74E-06	0.015599	1.1E-05	9	6	3	6	8	17	11	12
pduO	20	12	14.66667	1.427184	1.3E-12	3.361085	6.35E-06	5.74E-09	8.81E-12	34	34	35	34.33333	54	46	47	49
pduP	136	49	92.33333	2.964539	1.04E-26	6.980252	2.19E-05	4.6E-23	1.89E-25	54	35	52	47	146	101	171	139.3333
pduQ	68	40	55	2.793478	4.78E-26	6.600303	1.34E-05	2.12E-22	8.47E-25	35	27	30	30.66667	92	95	70	85.66667
pduS	54	2	26.33333	1.849462	8.64E-13	4.349463	8.1E-06	3.83E-09	6E-12	36	21	36	31	38	75	59	57.33333

Feature ID	Experiment - Range (original values)	Experiment - IQR (original values)	Experiment - Difference (original values)	Experiment - Fold Change (original values)	EDGE test: yccT H202 vs yccT NT , tagwise dispersions - P-value	EDGE test: yccT H202 vs yccT NT , tagwise dispersions - Fold change	EDGE test: yccT H202 vs yccT NT , tagwise dispersions - Weighted difference	EDGE test: yccT H202 vs yccT NT , tagwise dispersions - Bonferroni	EDGE test: yccT H202 vs yccT NT , tagwise dispersions - FDR p-value correction	yccT NT - yccT.1.S28 - Expression values	yccT NT - yccT.2.S29 - Expression values	yccT NT - yccT.3.S30 - Expression values	yccT NT - Means	yccT H202 - yccT.1.H2O2 - Expression values	yccT H202 - yccT.2.H2O2 - Expression values	yccT H202 - yccT.3.H2O2 - Expression values	yccT H202 - Means
pduT	32	6	14.33333	1.934783	2.3E-09	4.540206	4.27E-06	1.02E-05	1.09E-08	17	15	14	15.33333	22	46	21	29.66667
pduU	28	3	6.666667	1.555556	0.000134	3.620895	2.49E-06	0.592905	0.000332	13	13	10	12	6	34	16	18.66667
pduV	15	6	1	1.085714	0.003224	2.557019	1.44E-06	1	0.006248	9	11	15	11.66667	15	19	4	12.66667
pduW	42	35	37.66667	1.530516	7.88E-19	3.606075	1.45E-05	3.49E-15	8.93E-18	72	73	68	71	107	109	110	108.6667
pduX	449	53	171	1.145739	3.63E-09	2.69962	0.000155	1.61E-05	1.68E-08	1209	1100	1211	1173.333	1326	1158	1549	1344.333
pegB	15	6	1.666667	1.090909	0.000137	2.554026	2.25E-06	0.608463	0.00034	9	24	22	18.33333	22	22	16	20
pegC	50	6	20.66667	1.285714	1.52E-11	3.034964	1.15E-05	6.72E-08	9.18E-11	73	79	65	72.33333	87	115	77	93
pegD	20	5	-4.33333	-1.04483	1.33E-08	2.260093	9.92E-06	5.88E-05	5.7E-08	109	96	98	101	89	108	93	96.66667
pepE	1517	1361	-1432.67	-8.88624	3.2E-19	-3.77311	-9.3E-05	1.42E-15	3.71E-18	1616	1677	1550	1614.333	160	196	189	181.6667
pepT	6889	6496	-6497	-5.15675	0.000182	-2.16654	-0.00034	0.804483	0.000442	8204	8160	7816	8060	2054	1320	1315	1563
pfkA	342	165	-201.667	-1.13207	1.59E-05	2.087634	0.000146	0.070611	4.56E-05	1776	1634	1776	1728.667	1434	1656	1491	1527
pflB	8125	3643	449.3333	1.022291	1.81E-05	2.438859	0.002255	0.080157	5.14E-05	22138	18495	19841	20158	23642	22663	15517	20607.33
pflF	269	52	-29.3333	-1.06241	4.49E-07	2.206073	4.7E-05	0.00199	1.58E-06	491	501	506	499.3333	449	346	615	470
pgi	705	159	-305.333	-1.09259	2.08E-05	2.163226	0.000326	0.092377	5.85E-05	3895	3475	3439	3603	3280	3190	3423	3297.667
pgsA	1536	1321	-1396	-6.53236	5.73E-09	-2.78751	-8.2E-05	2.54E-05	2.58E-08	1708	1553	1684	1648.333	232	172	353	252.3333
pgtA	949	690	-804.333	-5.30125	5.75E-09	-2.2483	-4.3E-05	2.55E-05	2.59E-08	1110	868	996	991.3333	161	178	222	187
pgtB	1950	1488	-1710.67	-8.94427	2.22E-16	-3.78543	-0.00011	9.84E-13	2.11E-15	2116	1716	1946	1926	228	166	252	215.3333
pgtC	1220	1008	-1122.33	-13.95	4.56E-23	-5.92076	-7.8E-05	2.02E-19	6.64E-22	1253	1103	1271	1209	95	51	114	86.66667
pgtE	230	10	60.66667	1.598684	1.83E-06	3.802846	2.22E-05	0.008119	5.95E-06	104	95	105	101.3333	112	302	72	162
phnA	330	311	-304.667	-5.29108	5.57E-06	-2.23427	-1.6E-05	0.024673	1.68E-05	382	376	369	375.6667	58	103	52	71
phnS	45	12	28.66667	1.562092	6.44E-16	3.684111	1.07E-05	2.85E-12	5.86E-15	53	43	57	51	88	65	86	79.66667
phnT	293	172	208	2.714286	1.16E-30	6.386447	5.09E-05	5.15E-27	2.45E-29	114	95	155	121.3333	314	286	388	329.3333
phnU	40	14	22.66667	1.790698	1.06E-15	4.196776	7.17E-06	4.72E-12	9.4E-15	32	25	29	28.66667	43	46	65	51.33333
phoH	3187	2671	2756	2.989892	2.06E-23	7.095333	0.000655	9.11E-20	3.08E-22	1644	1184	1327	1385	3998	4371	4054	4141
phoN	43	4	-17.6667	-1.16667	5.34E-06	2.029973	9.95E-06	0.023643	1.61E-05	124	129	118	123.6667	118	114	86	106
phsA	11223	8632	-9665.67	-5.56646	0.0011	-2.35411	-0.00053	1	0.002342	12073	12130	11144	11782.33	2931	907	2512	2116.667
pipA	577	498	-507	-5.27247	2.33E-05	-2.25733	-2.7E-05	0.103425	6.48E-05	643	629	605	625.6667	107	66	183	118.6667
pipB	29	9	14.66667	1.382609	2.61E-12	3.240665	6.73E-06	1.16E-08	1.72E-11	37	43	35	38.33333	46	49	64	53
pipD	2567	2454	-2480	-21.9577	3.41E-34	-9.36215	-0.00018	1.51E-30	8.49E-33	2653	2562	2580	2598.333	108	86	161	118.3333
pocR	2850	2750	-2801.67	-50.4412	3.66E-83	-21.4023	-0.00021	1.62E-79	2.54E-81	2868	2902	2805	2858.333	52	55	63	56.66667

Feature ID	Experiment - Range (original values)	Experiment - IQR (original values)	Experiment - Difference (original values)	Experiment - Fold Change (original values)	EDGE test: yccT H202 vs yccT NT , tagwise dispersions - P-value	EDGE test: yccT H202 vs yccT NT , tagwise dispersions - Fold change	EDGE test: yccT H202 vs yccT NT , tagwise dispersions - Weighted difference	EDGE test: yccT H202 vs yccT NT , tagwise dispersions - Bonferroni	EDGE test: yccT H202 vs yccT NT , tagwise dispersions - FDR p-value correction	yccT NT - yccT.1.S28 - Expression values	yccT NT - yccT.2.S29 - Expression values	yccT NT - yccT.3.S30 - Expression values	yccT NT - Means	yccT H202 - yccT.1.H2O2 - Expression values	yccT H202 - yccT.2.H2O2 - Expression values	yccT H202 - yccT.3.H2O2 - Expression values	yccT H202 - Means
polB	4948	3200	4266.333	6.192292	1.31E-36	14.61124	0.000871	5.82E-33	3.64E-35	860	803	802	821.6667	5511	4003	5750	5088
potA	601	488	-560	-14.8843	4.42E-33	-6.29435	-3.9E-05	1.96E-29	1.03E-31	635	529	637	600.3333	36	41	44	40.33333
potC	820	714	-759.667	-6.25115	2.19E-12	-2.64742	-4.4E-05	9.69E-09	1.45E-11	946	901	866	904.3333	152	126	156	144.6667
potE	36537	20767	28736	21.83325	2.6E-68	51.72658	0.005456	1.15E-64	1.33E-66	1356	1363	1419	1379.333	37893	22130	30323	30115.33
potF	23	11	-5	-1.03261	3.03E-09	2.285996	1.59E-05	1.34E-05	1.42E-08	160	166	149	158.3333	164	143	153	153.3333
pphB	25	10	15.66667	2.516129	9.63E-14	5.864458	3.97E-06	4.27E-10	7.25E-13	14	5	12	10.33333	26	22	30	26
ppiA	913	878	-895	-6.11429	5.49E-12	-2.58814	-5.1E-05	2.43E-08	3.48E-11	1088	1053	1069	1070	175	175	175	175
ppiC	552	466	-503.333	-5.27762	5.47E-08	-2.24319	-2.7E-05	0.000242	2.18E-07	639	633	591	621	87	141	125	117.6667
pps	24121	19939	-21491.7	-5.17368	7.29E-05	-2.19034	-0.00113	0.323027	0.000189	28015	26211	25697	26641	5796	3894	5758	5149.333
pqaA	108	19	55	1.816832	8.05E-11	4.262593	1.72E-05	3.57E-07	4.46E-10	64	59	79	67.33333	140	60	167	122.3333
prc	3912	3130	-3610	-4.96413	2.66E-05	-2.09864	-0.00018	0.117835	7.33E-05	4777	4043	4742	4520.667	865	954	913	910.6667
prfB	1916	1527	-1725	-5.29817	2.03E-06	-2.23835	-9.1E-05	0.009006	6.55E-06	2248	1887	2244	2126.333	332	512	360	401.3333
prgH	46	14	-14.6667	-1.09821	3.53E-08	2.152568	1.47E-05	0.000156	1.45E-07	168	139	185	164	154	140	154	149.3333
prgI	20	7	5.666667	1.10559	6.58E-09	2.607553	6.75E-06	2.91E-05	2.94E-08	49	50	62	53.66667	57	69	52	59.33333
prgJ	47	21	-12	-1.18274	0.00034	2.009286	6.11E-06	1	0.00079	83	63	87	77.66667	62	91	44	65.66667
prgK	47	16	-6.66667	-1.0489	7.97E-08	2.250706	1.4E-05	0.000353	3.09E-07	133	140	156	143	124	166	119	136.3333
priC	75	21	28.33333	1.141903	5.41E-12	2.678123	2.62E-05	2.4E-08	3.43E-11	178	217	204	199.6667	196	235	253	228
prlC	11435	8227	9870.667	3.620995	7.12E-27	8.538761	0.002212	3.15E-23	1.31E-25	3911	3740	3647	3766	13861	11967	15082	13636.67
proB	1552	1175	-1350.67	-4.81185	3.55E-06	-2.03535	-6.7E-05	0.015737	1.1E-05	1853	1525	1737	1705	301	412	350	354.3333
proC	4631	605	241	1.065943	0.000601	2.539096	0.000438	1	0.001342	3957	3599	3408	3654.667	2994	6662	2031	3895.667
proP	905	201	98.33333	1.161999	0.000133	2.757043	8.3E-05	0.590614	0.000331	645	562	614	607	399	1304	413	705.3333
proQ	2808	2338	-2610.67	-4.91796	1.31E-05	-2.08291	-0.00013	0.057835	3.77E-05	3397	3022	3412	3277	604	711	684	666.3333
prpB	348	188	-272	-6.40397	2.81E-08	-2.70985	-1.6E-05	0.000125	1.16E-07	346	241	380	322.3333	53	32	66	50.33333
prpD	509	246	-384	-5.15884	1.26E-06	-2.1824	-2E-05	0.005561	4.16E-06	499	344	586	476.3333	77	98	102	92.33333
psd	988	794	-872.333	-6.10136	8.08E-11	-2.58537	-5E-05	3.58E-07	4.47E-10	1122	972	1036	1043.333	178	134	201	171
pspE	1417	1274	-1298.67	-10.0394	5.61E-16	-4.29852	-8.6E-05	2.49E-12	5.13E-15	1401	1512	1414	1442.333	127	95	209	143.6667
pspF	61	16	-27.3333	-1.09762	2.8E-08	2.144452	2.75E-05	0.000124	1.16E-07	280	322	320	307.3333	283	261	296	280
pssA	4475	3902	-4115	-7.95101	1.63E-11	-3.36825	-0.00026	7.2E-08	9.8E-11	4996	4616	4509	4707	607	521	648	592
pstB	506	404	-448.667	-6.19691	4.67E-12	-2.626	-2.6E-05	2.07E-08	2.98E-11	580	493	532	535	74	89	96	86.33333
pth	563	447	-480.667	-5.82274	7.51E-08	-2.47533	-2.7E-05	0.000333	2.93E-07	625	549	567	580.3333	102	62	135	99.66667

Feature ID	Experiment - Range (original values)	Experiment - IQR (original values)	Experiment - Difference (original values)	Experiment - Fold Change (original values)	EDGE test: yccT H202 vs yccT NT , tagwise dispersions - P-value	EDGE test: yccT H202 vs yccT NT , tagwise dispersions - Fold change	EDGE test: yccT H202 vs yccT NT , tagwise dispersions - Weighted difference	EDGE test: yccT H202 vs yccT NT , tagwise dispersions - Bonferroni	EDGE test: yccT H202 vs yccT NT , tagwise dispersions - FDR p-value correction	yccT NT - yccT.1.S28 - Expression values	yccT NT - yccT.2.S29 - Expression values	yccT NT - yccT.3.S30 - Expression values	yccT NT - Means	yccT H202 - yccT.1.H2O2 - Expression values	yccT H202 - yccT.2.H2O2 - Expression values	yccT H202 - yccT.3.H2O2 - Expression values	yccT H202 - Means
ptrB	903	78	557	1.376351	6.83E-10	3.244604	0.000259	3.03E-06	3.45E-09	1521	1462	1457	1480	2211	1540	2360	2037
pudB	558	359	-470.333	-10.4698	7.02E-18	-4.45683	-3.2E-05	3.11E-14	7.35E-17	414	597	549	520	55	39	55	49.66667
purA	1565	469	-142.333	-1.02133	5.86E-06	2.306227	0.000693	0.025948	1.77E-05	7129	6430	6884	6814.333	5776	6899	7341	6672
purE	118	90	-101.333	-7.6087	1.77E-10	-3.21492	-6.3E-06	7.84E-07	9.52E-10	114	107	129	116.6667	17	11	18	15.33333
purR	645	472	-560.667	-5.4147	4.41E-09	-2.29153	-3E-05	1.95E-05	2E-08	750	603	710	687.6667	105	145	131	127
purT	115	18	35	1.216049	3.39E-10	2.879554	2.37E-05	1.5E-06	1.77E-09	159	146	181	162	174	261	156	197
putP	771	522	-629.333	-6.30337	9.95E-10	-2.68561	-3.7E-05	4.41E-06	4.95E-09	644	743	857	748	122	86	148	118.6667
pyrB	1030	657	-829.333	-6.23789	1.39E-06	-2.64835	-4.8E-05	0.006149	4.59E-06	1016	838	1109	987.6667	181	79	215	158.3333
pyrD	440	407	-418.667	-8.34503	1.34E-18	-3.53486	-2.7E-05	5.92E-15	1.51E-17	492	471	464	475.6667	57	52	62	57
pyrH	839	646	-753	-8.03738	3.17E-16	-3.39025	-4.7E-05	1.41E-12	2.97E-15	901	746	933	860	100	127	94	107
pyrI	717	570	-644.333	-5.22976	4.62E-08	-2.21385	-3.4E-05	0.000205	1.86E-07	816	737	837	796.6667	167	120	170	152.3333
pyrL	1577	1297	-1393.67	-6.71956	1.62E-06	-2.86081	-8.3E-05	0.007158	5.29E-06	1688	1575	1649	1637.333	278	111	342	243.6667
queA	544	457	-501.667	-7.93548	4E-17	-3.35077	-3.1E-05	1.77E-13	4.02E-16	611	527	584	574	70	80	67	72.33333
rarD	150	31	32.33333	1.146747	4.1E-10	2.693479	2.9E-05	1.82E-06	2.11E-09	250	195	216	220.3333	226	191	341	252.6667
rbn	253	127	-119	-1.14127	1.81E-06	2.074717	8.04E-05	0.008037	5.91E-06	1049	901	934	961.3333	802	929	796	842.3333
rbsA	390	7	153.6667	1.881453	8.56E-09	4.390027	4.61E-05	3.79E-05	3.77E-08	170	176	177	174.3333	354	120	510	328
rbsB	9733	2099	-1827.33	-1.13163	0.000586	2.071418	0.001311	1	0.001312	16269	15251	15608	15709.33	13509	9202	18935	13882
rbsD	380	5	194.3333	3.175373	3.53E-12	7.399385	4.46E-05	1.56E-08	2.27E-11	94	85	89	89.33333	295	91	465	283.6667
rbsK	6653	6037	-6272.33	-14.0492	0	-5.95298	-0.00044	0	0	7072	6529	6658	6753	492	419	531	480.6667
rbsR	3351	3009	-3150.67	-14.3315	0	-6.05802	-0.00022	0	0	3570	3250	3341	3387	249	219	241	236.3333
rcsF	617	538	-557	-10.0815	2.34E-13	-4.31697	-3.7E-05	1.04E-09	1.7E-12	594	649	612	618.3333	56	32	96	61.33333
rdgC	1018	916	-941.667	-4.85929	1.33E-07	-2.06154	-4.8E-05	0.000588	4.99E-07	1241	1150	1166	1185.667	234	223	275	244
recA	55715	15084	35970.67	6.631856	8.6E-24	15.52599	0.007226	3.81E-20	1.31E-22	6720	6324	6117	6387	43833	21408	61832	42357.67
recN	19932	8810	14643.67	15.76176	1.43E-45	37.00624	0.002782	6.33E-42	5.51E-44	1040	961	975	992	16229	9785	20893	15635.67
rfaL	1160	989	-1081	-4.7448	1.61E-06	-2.00491	-5.4E-05	0.00713	5.28E-06	1427	1396	1286	1369.667	297	302	267	288.6667
rhaR	384	321	-339	-6.29688	1.49E-10	-2.67756	-2E-05	6.62E-07	8.12E-10	384	393	432	403	63	48	81	64
rhlB	4772	4142	-4370	-7.68878	6.69E-11	-3.26667	-0.00027	2.97E-07	3.74E-10	5357	4740	4973	5023.333	598	585	777	653.3333
ribA	713	142	132.6667	1.063025	4.37E-07	2.503774	0.000246	0.001938	1.54E-06	2268	2008	2039	2105	2150	1925	2638	2237.667
ribD	381	191	-59.6667	-1.04578	1.43E-06	2.253983	0.000133	0.006352	4.72E-06	1472	1213	1404	1363	1229	1150	1531	1303.333
rimJ	3962	3607	-3800.33	-12.0797	0	-5.14475	-0.00026	0	0	4254	4254	3922	4143.333	315	292	422	343

Feature ID	Experiment - Range (original values)	Experiment - IQR (original values)	Experiment - Difference (original values)	Experiment - Fold Change (original values)	EDGE test: yccT H202 vs yccT NT , tagwise dispersions - P-value	EDGE test: yccT H202 vs yccT NT , tagwise dispersions - Fold change	EDGE test: yccT H202 vs yccT NT , tagwise dispersions - Weighted difference	EDGE test: yccT H202 vs yccT NT , tagwise dispersions - Bonferroni	EDGE test: yccT H202 vs yccT NT , tagwise dispersions - FDR p-value correction	yccT NT - yccT.1.S28 - Expression values	yccT NT - yccT.2.S29 - Expression values	yccT NT - yccT.3.S30 - Expression values	yccT NT - Means	yccT H202 - yccT.1.H2O2 - Expression values	yccT H202 - yccT.2.H2O2 - Expression values	yccT H202 - yccT.3.H2O2 - Expression values	yccT H202 - Means
rluC	419	367	-383.333	-12.2745	1.72E-24	-5.20734	-2.6E-05	7.61E-21	2.77E-23	444	398	410	417.3333	31	25	46	34
rmbA	15	6	9.333333	2.12	3.8E-09	4.945607	2.61E-06	1.68E-05	1.76E-08	12	6	7	8.333333	21	19	13	17.66667
rna-AM93	377	346	-349.333	-9.25197	2.36E-16	-3.93774	-2.3E-05	1.04E-12	2.23E-15	406	383	386	391.6667	37	29	61	42.33333
rna-AM93	913	693	-780.333	-9.45126	2.25E-10	-4.02557	-5.1E-05	9.96E-07	1.2E-09	870	796	952	872.6667	103	39	135	92.33333
rna-AM93	539	484	-489.333	-7.95735	4.73E-11	-3.41135	-3.1E-05	2.09E-07	2.7E-10	538	556	585	559.6667	54	46	111	70.33333
rna-AM93	244	206	-228.667	-5.76389	8.33E-10	-2.43614	-1.3E-05	3.69E-06	4.16E-09	288	252	290	276.6667	46	52	46	48
rna-AM93	554	434	-482.333	-11.6397	6.2E-25	-4.94051	-3.3E-05	2.75E-21	1.02E-23	513	479	591	527.6667	37	45	54	45.33333
rna-AM93	203	161	-184.667	-21.5185	1.87E-29	-9.05348	-1.3E-05	8.29E-26	3.82E-28	210	200	171	193.6667	10	7	10	9
rna-AM93	107	102	-103.333	-12.4815	1.31E-16	-5.26311	-7.1E-06	5.82E-13	1.27E-15	113	112	112	112.3333	6	10	11	9
rna-AM93	7	1	1	1.083333	0.000542	2.535241	1.46E-06	1	0.001224	10	10	16	12	11	17	11	13
rna-AM93	143	61	-90	-7.58537	3.7E-05	-3.2443	-5.6E-06	0.163834	9.98E-05	92	71	148	103.6667	5	10	26	13.66667
rna-AM93	39	33	-35.3333	-4.92593	0.00147	-2.08174	-1.8E-06	1	0.003054	42	46	45	44.33333	9	7	11	9
rna-AM93	118	76	-94	-5.47619	2.97E-06	-2.32642	-5.1E-06	0.013149	9.34E-06	113	135	97	115	17	21	25	21
rna-AM93	1437	1232	-1300.33	-7.18225	4.35E-14	-3.03952	-7.9E-05	1.93E-10	3.37E-13	1442	1448	1642	1510.667	216	210	205	210.3333
rna-AM93	384	318	-346	-10.9808	2.29E-23	-4.65307	-2.3E-05	1.01E-19	3.41E-22	353	373	416	380.6667	32	37	35	34.66667
rna-AM93	29	24	-26.6667	-12.4286	3.23E-06	-5.13919	-1.8E-06	0.014325	1.01E-05	30	31	26	29	2	2	3	2.333333
rna-AM93	250	186	-207.667	-15.8333	5.79E-23	-6.69957	-1.5E-05	2.56E-19	8.35E-22	201	206	258	221.6667	8	19	15	14
rna-AM93	1086	912	-1004.33	-24	2.66E-45	-10.2068	-7.4E-05	1.18E-41	1.01E-43	1077	948	1119	1048	36	33	62	43.66667
rna-AM93	1267	1165	-1200.67	-30.7686	1.83E-62	-13.0945	-8.9E-05	8.09E-59	8.61E-61	1225	1200	1298	1241	31	35	55	40.33333
rna-AM93	426	388	-396.333	-9.49286	3.26E-19	-4.03165	-2.6E-05	1.44E-15	3.77E-18	461	436	432	443	44	35	61	46.66667
rna-AM93	73	47	-56	-5	0.00053	-2.10791	-2.9E-06	1	0.0012	63	66	81	70	16	18	8	14
rna-AM93	61	40	-47.3333	-5.17647	0.000225	-2.18379	-2.5E-06	0.995315	0.000536	70	51	55	58.66667	9	11	14	11.33333
rna-AM93	19	4	0.666667	1.017699	2.71E-06	2.38477	4.09E-06	0.012009	8.6E-06	35	39	39	37.66667	27	42	46	38.33333
rna-AM93	4	2	2.333333	2	0.003843	4.463405	6.76E-07	1	0.007352	2	1	4	2.333333	5	5	4	4.666667
rna-AM93	309	279	-282.667	-7.89431	2.8E-09	-3.37466	-1.8E-05	1.24E-05	1.32E-08	317	326	328	323.6667	38	19	66	41
rna-AM93	712	655	-674.333	-7.30218	9.47E-17	-3.09574	-4.1E-05	4.19E-13	9.28E-16	810	770	764	781.3333	98	109	114	107
rna-AM93	122	18	73.66667	3.428571	3.59E-17	7.965439	1.65E-05	1.59E-13	3.64E-16	36	26	29	30.33333	47	117	148	104
rna-AM93	21	7	12.33333	1.616667	3.98E-11	3.797375	4.39E-06	1.76E-07	2.28E-10	25	20	15	20	36	27	34	32.33333
rna-AM93	145	119	-133.333	-8.69231	6.42E-14	-3.66905	-8.5E-06	2.85E-10	4.92E-13	161	155	136	150.6667	19	17	16	17.33333
rna-AM93	838	788	-806.667	-20.6748	9.41E-47	-8.77761	-5.9E-05	4.17E-43	3.69E-45	846	826	871	847.6667	38	33	52	41

Feature ID	Experiment - Range (original values)	Experiment - IQR (original values)	Experiment - Difference (original values)	Experiment - Fold Change (original values)	EDGE test: yccT H202 vs yccT NT , tagwise dispersions - P-value	EDGE test: yccT H202 vs yccT NT , tagwise dispersions - Fold change	EDGE test: yccT H202 vs yccT NT , tagwise dispersions - Weighted difference	EDGE test: yccT H202 vs yccT NT , tagwise dispersions - Bonferroni	EDGE test: yccT H202 vs yccT NT , tagwise dispersions - FDR p-value correction	yccT NT - yccT.1.S28 - Expression values	yccT NT - yccT.2.S29 - Expression values	yccT NT - yccT.3.S30 - Expression values	yccT NT - Means	yccT H202 - yccT.1.H2O2 - Expression values	yccT H202 - yccT.2.H2O2 - Expression values	yccT H202 - yccT.3.H2O2 - Expression values	yccT H202 - Means
rna-AM93	693	567	-626	-22.3409	1.59E-35	-9.48163	-4.6E-05	7.06E-32	4.23E-34	601	656	709	655.3333	34	16	38	29.33333
rna-AM93	221	165	-192.333	-18.4848	2.3E-25	-7.82171	-1.4E-05	1.02E-21	3.88E-24	176	206	228	203.3333	11	7	15	11
rna-AM93	1210	1099	-1154.33	-41.2674	2.99E-38	-17.6806	-8.7E-05	1.32E-34	9.07E-37	1207	1117	1225	1183	18	15	53	28.66667
rna-AM93	1179	1063	-1119.67	-42.9875	5.85E-61	-18.2362	-8.4E-05	2.59E-57	2.7E-59	1155	1091	1193	1146.333	28	14	38	26.66667
rna-AM93	745	674	-703.667	-15.3605	5.27E-35	-6.52402	-5E-05	2.34E-31	1.37E-33	721	790	747	752.6667	45	47	55	49
rna-AM93	932	843	-870.333	-5.84416	4.86E-10	-2.49161	-4.9E-05	2.15E-06	2.48E-09	1062	1016	1072	1050	140	173	226	179.6667
rna-AM93	909	798	-826.667	-8.60736	1.71E-13	-3.67899	-5.3E-05	7.56E-10	1.25E-12	982	888	936	935.3333	90	73	163	108.6667
rna-AM93	176	70	112.6667	1.651252	3.19E-21	3.881411	3.89E-05	1.41E-17	4.17E-20	178	186	155	173	278	248	331	285.6667
rna-AM93	358	134	261.6667	1.686189	4.07E-19	3.969603	8.84E-05	1.8E-15	4.68E-18	368	394	382	381.3333	687	516	726	643
rna-AM93	62	22	43	1.431438	2.88E-17	3.370695	1.84E-05	1.28E-13	2.93E-16	101	102	96	99.66667	147	123	158	142.6667
rna-AM93	182	140	-153	-7.03947	2.07E-10	-2.98247	-9.2E-06	9.18E-07	1.1E-09	168	168	199	178.3333	28	17	31	25.33333
rna-AM93	124	102	-110	-6.5	2.01E-09	-2.75185	-6.5E-06	8.92E-06	9.66E-09	127	122	141	130	17	20	23	20
rna-AM93	225	180	-195.667	-5.48092	7.76E-08	-2.32876	-1.1E-05	0.000344	3.02E-07	255	235	228	239.3333	30	48	53	43.66667
rna-AM93	166	148	-153.333	-6.22727	5.42E-10	-2.63262	-8.8E-06	2.4E-06	2.76E-09	179	176	193	182.6667	33	27	28	29.33333
rna-AM93	335	302	-313.333	-10.0385	4.62E-22	-4.25347	-2.1E-05	2.05E-18	6.33E-21	366	344	334	348	32	31	41	34.66667
rna-AM93	1102	1033	-1053	-9.49194	1.88E-17	-4.02585	-6.9E-05	8.34E-14	1.93E-16	1194	1188	1149	1177	92	164	116	124
rna-AM93	576	493	-533	-8.87685	3.5E-18	-3.77352	-3.4E-05	1.55E-14	3.81E-17	609	626	567	600.6667	50	79	74	67.66667
rna-AM93	149	114	-133.333	-9.88889	1.35E-13	-4.15685	-8.8E-06	5.99E-10	1.01E-12	158	129	158	148.3333	15	21	9	15
rna-AM93	37	19	-27	-10	3.53E-05	-4.15999	-1.8E-06	0.156303	9.55E-05	30	38	22	30	5	3	1	3
rna-AM93	51	41	-43.3333	-5.33333	0.000534	-2.24821	-2.3E-06	1	0.001207	50	54	56	53.33333	9	16	5	10
rnb	1915	1521	-1702.67	-5.21105	3.05E-07	-2.21193	-9E-05	0.001351	1.11E-06	2107	1951	2263	2107	348	430	435	404.3333
rnc	739	721	-713.333	-5.68271	1.39E-09	-2.40602	-3.9E-05	6.14E-06	6.79E-09	864	874	859	865.6667	135	184	138	152.3333
rnd	342	323	-322.667	-4.872	3.1E-07	-2.07016	-1.6E-05	0.001375	1.13E-06	403	403	412	406	80	70	100	83.33333
rnk	517	423	-451	-7.53623	2.54E-14	-3.20956	-2.8E-05	1.13E-10	2.02E-13	502	487	571	520	54	64	89	69
rnpA	970	812	-889.333	-26.6538	3.84E-39	-11.3458	-6.6E-05	1.7E-35	1.2E-37	990	841	941	924	29	20	55	34.66667
rph	344	322	-331.333	-10.5577	9.25E-23	-4.4713	-2.2E-05	4.1E-19	1.32E-21	369	357	372	366	28	41	35	34.66667
rpiA	2169	1910	-1997.33	-5.55665	9.19E-08	-2.35061	-0.00011	0.000407	3.54E-07	2552	2385	2370	2435.667	472	383	460	438.3333
rplK	2616	2176	-2263	-5.26177	0.000158	-2.2116	-0.00012	0.700488	0.000387	2964	2645	2773	2794	469	776	348	531
rplN	6826	5355	-5843.33	-5.54498	0.000156	-2.32923	-0.00032	0.691198	0.000382	7689	6496	7202	7129	1141	1853	863	1285.667
rplO	4642	527	119.3333	1.032113	0.00106	2.466547	0.000424	1	0.002266	4008	3363	3777	3716	3250	6449	1807	3835.333

Feature ID	Experiment - Range (original values)	Experiment - IQR (original values)	Experiment - Difference (original values)	Experiment - Fold Change (original values)	EDGE test: yccT H202 vs yccT NT , tagwise dispersions - P-value	EDGE test: yccT H202 vs yccT NT , tagwise dispersions - Fold change	EDGE test: yccT H202 vs yccT NT , tagwise dispersions - Weighted difference	EDGE test: yccT H202 vs yccT NT , tagwise dispersions - Bonferroni	EDGE test: yccT H202 vs yccT NT , tagwise dispersions - FDR p-value correction	yccT NT - yccT.1.S28 - Expression values	yccT NT - yccT.2.S29 - Expression values	yccT NT - yccT.3.S30 - Expression values	yccT NT - Means	yccT H202 - yccT.1.H2O2 - Expression values	yccT H202 - yccT.2.H2O2 - Expression values	yccT H202 - yccT.3.H2O2 - Expression values	yccT H202 - Means
rplP	2631	89	223.6667	1.115272	0.000455	2.662249	0.000252	1	0.00104	1895	1984	1942	1940.333	2025	3549	918	2164
rplT	5354	1155	627.3333	1.067639	8.12E-06	2.536734	0.001108	0.035969	2.41E-05	9959	8355	9510	9274.667	8976	13042	7688	9902
rplU	4672	4017	-4196.33	-7.5228	1.24E-09	-3.17711	-0.00026	5.5E-06	6.11E-09	5235	4590	4694	4839.667	563	794	573	643.3333
rplX	7346	6035	-6268	-6.50952	2.16E-05	-2.73675	-0.00037	0.095824	6.04E-05	8085	6950	7182	7405.667	915	1759	739	1137.667
rpmA	4218	3353	-3588	-5.66176	8.59E-06	-2.38512	-0.0002	0.038052	2.54E-05	4848	4036	4189	4357.667	683	996	630	769.6667
rpmB	3395	2898	-2959	-6.03802	2.59E-05	-2.5412	-0.00017	0.114771	7.15E-05	3789	3370	3480	3546.333	472	896	394	587.3333
rpmC	1017	46	215	1.300279	3.13E-06	3.096637	0.000117	0.013854	9.8E-06	702	746	700	716	848	1481	464	931
rpmD	966	262	-121	-1.15599	0.004427	2.062861	7.42E-05	1	0.008348	937	864	889	896.6667	627	1333	367	775.6667
rpmE2	92	22	43.66667	1.503846	1.06E-11	3.520821	1.71E-05	4.71E-08	6.53E-11	82	104	74	86.66667	139	86	166	130.3333
rpmH	714	645	-654.333	-5.40135	6.79E-07	-2.28802	-3.5E-05	0.003009	2.34E-06	822	777	810	803	108	206	132	148.6667
rpmI	4243	1521	-601.333	-1.07387	0.000108	2.210615	0.000823	0.477405	0.000271	9490	8034	8702	8742	7181	10742	6499	8140.667
rpmJ2	121	47	7.666667	1.036741	4.55E-08	2.445359	2.35E-05	0.000202	1.84E-07	235	185	206	208.6667	232	148	269	216.3333
rpoB	5488	1629	1282.333	1.083447	1.61E-06	2.578601	0.001886	0.007135	5.28E-06	16718	13675	15708	15367	19163	16207	14578	16649.33
rpoC	16770	1880	706.3333	1.022555	3.87E-05	2.433622	0.003499	0.171291	0.000104	32199	31429	30319	31315.67	34216	39310	22540	32022
rpoH	10372	5405	8232.333	2.013751	4.18E-15	4.756372	0.002374	1.85E-11	3.5E-14	8653	7805	7904	8120.667	17573	13309	18177	16353
rpoS	26990	1677	3041	1.095687	2.76E-05	2.586158	0.00392	0.12214	7.58E-05	35038	30062	30242	31780.67	22868	49858	31739	34821.67
rpsC	4388	491	-25	-1.00755	0.003076	2.368427	0.000356	1	0.005973	3290	3295	3424	3336.333	2804	5759	1371	3311.333
rpsG	3351	2610	-2985	-4.81714	4.97E-05	-2.03209	-0.00015	0.220378	0.000132	4080	3366	3855	3767	756	861	729	782
rpsL	2631	2159	-2372.67	-6.50077	3.14E-10	-2.75551	-0.00014	1.39E-06	1.64E-09	3030	2566	2816	2804	407	399	488	431.3333
rpsO	2763	2359	-2437	-5.13752	0.00014	-2.16792	-0.00013	0.618173	0.000345	3217	2819	3042	3026	454	853	460	589
rpsP	1273	1101	-1135.67	-5.07049	7.36E-05	-2.13717	-5.9E-05	0.326068	0.00019	1473	1322	1449	1414.667	221	416	200	279
rpsQ	2909	90	799	1.434475	3.87E-06	3.419977	0.000347	0.017151	1.2E-05	1835	1886	1796	1839	2435	4194	1285	2638
rpsT	4034	3406	-3644.67	-13.7883	0	-5.88755	-0.00025	0	0	4231	3690	3868	3929.667	197	284	374	285
rpsU	2480	2124	-2292.33	-14.0246	2.39E-29	-5.94419	-0.00016	1.06E-25	4.81E-28	2637	2303	2465	2468.333	157	179	192	176
rpsV	1353	246	717.6667	1.348326	4.11E-09	3.192791	0.000352	1.82E-05	1.89E-08	2055	2069	2057	2060.333	2623	3408	2303	2778
rrmA	239	172	-209.667	-7.1068	5.28E-12	-3.01068	-1.3E-05	2.34E-08	3.35E-11	260	207	265	244	26	35	42	34.33333
rsmC	93	37	-26	-1.09059	2.16E-08	2.165643	2.84E-05	9.55E-05	9.08E-08	319	275	345	313	297	252	312	287
rspB	14	10	-0.33333	-1.01786	0.001236	2.31846	1.97E-06	1	0.002605	20	24	13	19	23	23	10	18.66667
rstA	692	621	-632	-5.87404	4.4E-09	-2.51039	-3.6E-05	1.95E-05	2E-08	785	737	763	761.6667	93	116	180	129.6667
rtcA	296	57	-48.6667	-1.10125	6.93E-06	2.131823	4.66E-05	0.030706	2.07E-05	557	487	544	529.3333	303	599	540	480.6667

Feature ID	Experiment - Range (original values)	Experiment - IQR (original values)	Experiment - Difference (original values)	Experiment - Fold Change (original values)	EDGE test: yccT H202 vs yccT NT , tagwise dispersions - P-value	EDGE test: yccT H202 vs yccT NT , tagwise dispersions - Fold change	EDGE test: yccT H202 vs yccT NT , tagwise dispersions - Weighted difference	EDGE test: yccT H202 vs yccT NT , tagwise dispersions - Bonferroni	EDGE test: yccT H202 vs yccT NT , tagwise dispersions - FDR p-value correction	yccT NT - yccT.1.S28 - Expression values	yccT NT - yccT.2.S29 - Expression values	yccT NT - yccT.3.S30 - Expression values	yccT NT - Means	yccT H202 - yccT.1.H2O2 - Expression values	yccT H202 - yccT.2.H2O2 - Expression values	yccT H202 - yccT.3.H2O2 - Expression values	yccT H202 - Means
rtcB	722	101	219.3333	1.467994	2.54E-08	3.410782	8.79E-05	0.000113	1.06E-07	529	449	428	468.6667	321	700	1043	688
ruvA	213	69	-58.6667	-1.14321	1.59E-06	2.053344	3.85E-05	0.007036	5.22E-06	473	465	467	468.3333	398	309	522	409.6667
safA	53	5	24	2.945946	1.87E-10	6.944585	5.77E-06	8.28E-07	1E-09	14	7	16	12.33333	30	60	19	36.33333
safD	41	13	-0.66667	-1.01227	6.07E-05	2.335199	5.73E-06	0.269102	0.000159	55	44	66	55	40	81	42	54.33333
sanA	1083	928	-978.333	-5.27843	9.24E-08	-2.24572	-5.2E-05	0.000409	3.55E-07	1258	1146	1217	1207	218	175	293	228.6667
sapA	1498	1266	-1372	-5.10778	3.95E-07	-2.16735	-7.2E-05	0.00175	1.4E-06	1774	1600	1744	1706	334	276	392	334
sapB	317	292	-304	-10.5	1.12E-22	-4.44238	-2E-05	4.97E-19	1.6E-21	337	324	347	336	30	34	32	32
sapC	229	175	-198.667	-6.13793	2.06E-10	-2.59081	-1.1E-05	9.13E-07	1.1E-09	267	213	232	237.3333	38	40	38	38.66667
sapD	361	217	-288	-5.34171	5.3E-08	-2.25876	-1.5E-05	0.000235	2.12E-07	417	282	364	354.3333	56	65	78	66.33333
sbmC	6657	713	3615.667	2.985176	3.09E-13	6.970242	0.000847	1.37E-09	2.22E-12	1914	1821	1729	1821.333	5391	2534	8386	5437
sbp	65	23	21.33333	1.15534	5.62E-11	2.735618	1.86E-05	2.49E-07	3.17E-10	148	121	143	137.3333	186	125	165	158.6667
scsA	275	173	-224.667	-7.29907	3.21E-12	-3.07492	-1.4E-05	1.42E-08	2.09E-11	305	211	265	260.3333	39	30	38	35.66667
scsD	29	7	-3.33333	-1.06993	4.14E-06	2.214573	4.82E-06	0.018338	1.27E-05	66	37	50	51	46	54	43	47.66667
sdhA	3747	279	2092.333	1.574396	1.04E-09	3.715009	0.000769	4.62E-06	5.18E-09	3719	3211	3998	3642.667	6509	3738	6958	5735
sdhB	675	261	183.6667	1.065893	5.73E-07	2.523402	0.00033	0.00254	2E-06	2944	2408	3010	2787.333	3083	2749	3081	2971
sdhC	6063	269	2882.333	2.957664	6.6E-08	6.911686	0.000678	0.000292	2.6E-07	1426	1361	1630	1472.333	5109	946	7009	4354.667
sdhD	2767	93	1446.333	2.596394	2.05E-09	6.077216	0.000359	9.09E-06	9.84E-09	877	874	967	906	2746	772	3539	2352.333
secD	4130	3509	-3716	-5.74787	4.37E-06	-2.42772	-0.00021	0.01935	1.34E-05	4789	4191	4516	4498.667	659	1007	682	782.6667
secE	1659	1564	-1574.33	-5.38126	1.23E-07	-2.29309	-8.5E-05	0.000543	4.63E-07	1951	1880	1970	1933.667	316	311	451	359.3333
secG	4415	4282	-4325.33	-6.3115	2.8E-08	-2.6751	-0.00025	0.000124	1.16E-07	5202	5095	5122	5139.667	787	813	843	814.3333
selA	3318	2859	-3040.67	-5.33555	3.51E-06	-2.25815	-0.00016	0.015564	1.09E-05	3906	3617	3703	3742	758	588	758	701.3333
selB	3953	3395	-3593.33	-5.54469	3.34E-06	-2.33607	-0.0002	0.014811	1.04E-05	4688	4136	4328	4384	896	735	741	790.6667
SEN0014	7	4	4.333333	2.444444	1.82E-05	5.501525	1.11E-06	0.080778	5.17E-05	2	2	5	3	7	6	9	7.333333
SEN0015	23	16	17.66667	3.409091	3.36E-16	7.888979	4.03E-06	1.49E-12	3.13E-15	5	7	10	7.333333	28	24	23	25
SEN0016	11	3	5.666667	1.653846	1.41E-06	3.845189	1.96E-06	0.006254	4.65E-06	11	8	7	8.666667	10	18	15	14.33333
SEN0017	521	322	442.3333	2.434595	2.25E-29	5.751591	0.000114	9.98E-26	4.56E-28	326	298	301	308.3333	819	623	810	750.6667
SEN0029	15	10	12	1.972973	3.11E-11	4.608124	3.52E-06	1.38E-07	1.81E-10	12	13	12	12.33333	27	22	24	24.33333
SEN0031	8	5	0.666667	1.117647	0.014069	2.591556	7.22E-07	1	0.023968	10	2	5	5.666667	3	8	8	6.333333
SEN0032	26	11	13.66667	1.121662	2.9E-12	2.645923	1.44E-05	1.28E-08	1.9E-11	110	106	121	112.3333	121	132	125	126
SEN0034	19	18	16.66667	3.777778	6.79E-16	8.683745	3.7E-06	3.01E-12	6.14E-15	4	10	4	6	23	23	22	22.66667

Feature ID	Experiment - Range (original values)	Experiment - IQR (original values)	Experiment - Difference (original values)	Experiment - Fold Change (original values)	EDGE test: yccT H202 vs yccT NT , tagwise dispersions - P-value	EDGE test: yccT H202 vs yccT NT , tagwise dispersions - Fold change	EDGE test: yccT H202 vs yccT NT , tagwise dispersions - Weighted difference	EDGE test: yccT H202 vs yccT NT , tagwise dispersions - Bonferroni	EDGE test: yccT H202 vs yccT NT , tagwise dispersions - FDR p-value correction	yccT NT - yccT.1.S28 - Expression values	yccT NT - yccT.2.S29 - Expression values	yccT NT - yccT.3.S30 - Expression values	yccT NT - Means	yccT H202 - yccT.1.H2O2 - Expression values	yccT H202 - yccT.2.H2O2 - Expression values	yccT H202 - yccT.3.H2O2 - Expression values	yccT H202 - Means
SEN0055	9	4	-0.66667	-1.08	0.026408	2.165705	8.36E-07	1	0.042644	5	13	9	9	13	8	4	8.333333
SEN0085	1041	710	848.3333	5.85687	9.29E-54	13.87487	0.000175	4.12E-50	4.08E-52	177	154	193	174.6667	987	1195	887	1023
SEN0101	38	4	16.66667	1.943396	2.45E-10	4.509985	4.89E-06	1.08E-06	1.3E-09	13	19	21	17.66667	29	23	51	34.33333
SEN0110A	1630	114	920	2.087899	3.5E-12	4.890227	0.000257	1.55E-08	2.26E-11	839	892	806	845.6667	1908	953	2436	1765.667
SEN0159	10	2	-0.66667	-1.02273	1.09E-05	2.299013	3.05E-06	0.04849	3.2E-05	32	30	28	30	24	34	30	29.33333
SEN0163	459	96	243.6667	1.812222	4.53E-18	4.283841	7.66E-05	2.01E-14	4.8E-17	320	254	326	300	416	713	502	543.6667
SEN0164	67	32	50	2	3.07E-22	4.683187	1.44E-05	1.36E-18	4.24E-21	46	57	47	50	79	108	113	100
SEN0167	1784	14	747.3333	3.591908	1.62E-09	8.651186	0.000172	7.17E-06	7.84E-09	287	277	301	288.3333	2061	301	745	1035.667
SEN0168	2523	24	952.6667	2.887715	6.78E-08	6.981652	0.000235	0.0003	2.66E-07	520	498	496	504.6667	3004	481	887	1457.333
SEN0216	3925	3189	-3590.33	-10.1981	0	-4.31084	-0.00024	0	0	4286	3587	4069	3980.667	398	361	412	390.3333
SEN0249	1872	1687	-1733.33	-4.80117	1.05E-05	-2.04242	-8.7E-05	0.046571	3.08E-05	2247	2120	2201	2189.333	433	375	560	456
SEN0268	67	14	-32	-1.17235	3.46E-07	2.012746	1.72E-05	0.001535	1.25E-06	223	200	230	217.6667	190	163	204	185.6667
SEN0270	17	7	8.666667	1.412698	5.3E-09	3.314218	3.82E-06	2.35E-05	2.4E-08	18	19	26	21	26	35	28	29.66667
SEN0271	34	13	24.33333	2.351852	5.84E-18	5.496889	6.36E-06	2.59E-14	6.15E-17	19	15	20	18	32	46	49	42.33333
SEN0276	17	12	13	3.052632	9.68E-13	7.051017	3.06E-06	4.29E-09	6.67E-12	9	4	6	6.333333	19	21	18	19.33333
SEN0277	55	38	39.66667	4.305556	2.71E-25	10.09354	8.58E-06	1.2E-21	4.56E-24	21	7	8	12	47	62	46	51.66667
SEN0277A	17	8	13.33333	3.857143	3.4E-13	8.800076	2.94E-06	1.51E-09	2.44E-12	5	4	5	4.666667	13	21	20	18
SEN0278	20	10	13.33333	2.6	1.26E-12	6.034224	3.33E-06	5.58E-09	8.57E-12	13	9	3	8.333333	19	23	23	21.66667
SEN0280	6	2	2.666667	5	0.002512	9.562377	5.61E-07	1	0.005001	2	0	0	0.666667	2	6	2	3.333333
SEN0286	10	4	4.666667	2.75	2.21E-05	6.164158	1.14E-06	0.098051	6.17E-05	6	2	0	2.666667	6	10	6	7.333333
SEN0310	2810	2377	-2642.33	-31.1407	8.36E-37	-13.3151	-0.0002	3.7E-33	2.36E-35	2870	2869	2451	2730	74	60	129	87.66667
SEN0311	266	195	-232	-9.09302	7.94E-16	-3.85946	-1.5E-05	3.52E-12	7.13E-15	286	271	225	260.6667	20	30	36	28.66667
SEN0313	66	29	-4	-1.04633	7.36E-05	2.257889	8.83E-06	0.326322	0.00019	107	74	90	90.33333	103	45	111	86.33333
SEN0317	12	6	1.666667	1.056818	1.84E-06	2.486872	3.41E-06	0.00817	5.98E-06	35	28	25	29.33333	25	37	31	31
SEN0327	190	19	76.33333	1.269729	2.27E-13	3.006826	4.42E-05	1E-09	1.65E-12	296	251	302	283	322	441	315	359.3333
SEN0328	46	10	21	1.273913	3.91E-12	3.001952	1.2E-05	1.73E-08	2.51E-11	75	81	74	76.66667	85	120	88	97.66667
SEN0329	14	0	0.333333	1.037037	0.010306	2.400303	1E-06	1	0.017993	8	11	8	9	8	3	17	9.333333
SEN0330	19	10	-1	-1.03261	0.000126	2.261727	3.14E-06	0.55914	0.000314	26	36	33	31.66667	18	37	37	30.66667
SEN0331	19	7	11	3.2	1.64E-10	7.342902	2.55E-06	7.27E-07	8.85E-10	7	3	5	5	12	22	14	16
SEN0332	23	7	12	1.243243	6.57E-11	2.938552	7.47E-06	2.91E-07	3.68E-10	55	48	45	49.33333	64	68	52	61.33333

Feature ID	Experiment - Range (original values)	Experiment - IQR (original values)	Experiment - Difference (original values)	Experiment - Fold Change (original values)	EDGE test: yccT H202 vs yccT NT , tagwise dispersions - P-value	EDGE test: yccT H202 vs yccT NT , tagwise dispersions - Fold change	EDGE test: yccT H202 vs yccT NT , tagwise dispersions - Weighted difference	EDGE test: yccT H202 vs yccT NT , tagwise dispersions - Bonferroni	EDGE test: yccT H202 vs yccT NT , tagwise dispersions - FDR p-value correction	yccT NT - yccT.1.S28 - Expression values	yccT NT - yccT.2.S29 - Expression values	yccT NT - yccT.3.S30 - Expression values	yccT NT - Means	yccT H202 - yccT.1.H2O2 - Expression values	yccT H202 - yccT.2.H2O2 - Expression values	yccT H202 - yccT.3.H2O2 - Expression values	yccT H202 - Means
SEN0335	8	3	-1.33333	-1.11765	0.008321	2.090691	1.09E-06	1	0.014837	11	13	14	12.66667	10	8	16	11.33333
SEN0336	85	16	42.66667	1.258065	2.8E-14	2.967981	2.54E-05	1.24E-10	2.21E-13	178	164	154	165.3333	180	239	205	208
SEN0337	233	194	207.6667	2.253521	3.47E-33	5.308	5.57E-05	1.54E-29	8.22E-32	159	160	178	165.6667	354	374	392	373.3333
SEN0338	270	204	231.6667	3.118902	6.49E-42	7.354889	5.42E-05	2.87E-38	2.28E-40	109	101	118	109.3333	313	371	339	341
SEN0339	260	74	183.3333	1.726552	1.91E-20	4.055043	6.01E-05	8.48E-17	2.4E-19	251	234	272	252.3333	325	494	488	435.6667
SEN0342	20	5	8.666667	1.565217	8.52E-08	3.659541	3.22E-06	0.000378	3.29E-07	13	19	14	15.33333	19	33	20	24
SEN0346	3403	2284	2703.333	10.50762	1.66E-68	24.68538	0.000524	7.37E-65	8.57E-67	306	261	286	284.3333	2570	2729	3664	2987.667
SEN0385	20239	14859	17436.33	2.073908	1.19E-15	4.917549	0.00495	5.28E-12	1.05E-14	17377	15453	15879	16236.33	34588	35692	30738	33672.67
SEN0419	26	8	-12	-1.1629	9.55E-07	2.022704	6.84E-06	0.004231	3.23E-06	82	84	91	85.66667	65	74	82	73.66667
SEN0440	1308	298	813.3333	6.865385	1.68E-27	16.03005	0.000163	7.44E-24	3.17E-26	146	143	127	138.6667	980	441	1435	952
SEN0478	92	10	9.666667	1.06988	3.61E-08	2.515684	1.63E-05	0.00016	1.48E-07	172	120	123	138.3333	130	111	203	148
SEN0501	44	6	23	1.579832	6.99E-14	3.711471	8.39E-06	3.1E-10	5.33E-13	47	31	41	39.66667	46	67	75	62.66667
SEN0532	16	6	9	2.588235	2.37E-09	5.93908	2.25E-06	1.05E-05	1.12E-08	6	7	4	5.666667	12	12	20	14.66667
SEN0534A	8	2	0.333333	1.027778	0.001259	2.407459	1.33E-06	1	0.00265	14	10	12	12	9	17	11	12.33333
SEN0535	9	1	-0.66667	-1.02817	4.64E-05	2.291389	2.46E-06	0.205523	0.000123	30	22	21	24.33333	26	22	23	23.66667
SEN0537A	160	45	101.3333	3.62069	1.67E-22	8.488507	2.26E-05	7.41E-19	2.34E-21	38	28	50	38.66667	149	83	188	140
SEN0538	116	95	101.6667	5.295775	8.97E-52	12.45595	2.12E-05	3.97E-48	3.75E-50	28	17	26	23.66667	121	133	122	125.3333
SEN0538A	281	159	210.3333	3.124579	3.68E-33	7.385934	4.94E-05	1.63E-29	8.62E-32	90	96	111	99	302	371	255	309.3333
SEN0539	1097	877	956.6667	4.78628	8.01E-45	11.33356	0.000203	3.55E-41	3.01E-43	272	226	260	252.6667	1168	1323	1137	1209.333
SEN0540	11758	7492	10163.33	75.36585	1.34E-92	179.2521	0.0019	5.95E-89	1.1E-90	139	134	137	136.6667	11892	11379	7629	10300
SEN0541	56	34	44.33333	23.16667	6.47E-37	50.61847	8.41E-06	2.87E-33	1.84E-35	1	2	3	2	57	46	36	46.33333
SEN0542	602	202	389.6667	5.087413	5.8E-31	12.11934	8.25E-05	2.57E-27	1.24E-29	104	83	99	95.33333	469	685	301	485
SEN0618	28	8	-19.3333	-5.14286	0.023498	-2.16615	-1E-06	1	0.038335	28	32	12	24	4	4	6	4.666667
SEN0655	54	13	19.33333	1.174699	8.77E-12	2.771716	1.53E-05	3.88E-08	5.42E-11	120	101	111	110.6667	107	155	128	130
SEN0663	1836	1286	1637.667	5.683508	4.06E-38	13.48936	0.00034	1.8E-34	1.22E-36	348	336	365	349.6667	2156	2172	1634	1987.333
SEN0681	6708	3704	5156.333	3.70721	5E-26	8.71448	0.001145	2.21E-22	8.78E-25	1952	1934	1828	1904.667	7009	5638	8536	7061
SEN0708	847	718	-760.333	-6.41805	1.85E-11	-2.73252	-4.5E-05	8.21E-08	1.11E-10	890	955	857	900.6667	139	108	174	140.3333
SEN0714	18	2	-1.33333	-1.03846	5.1E-06	2.274659	3.58E-06	0.022616	1.55E-05	47	29	32	36	34	37	33	34.66667
SEN0715	19	1	-6	-1.10843	1.63E-06	2.123059	5.39E-06	0.00724	5.35E-06	58	69	57	61.33333	58	50	58	55.33333
SEN0716	1942	1432	1702.667	14.44211	1.02E-81	34.01705	0.000326	4.5E-78	6.82E-80	123	113	144	126.6667	1878	1555	2055	1829.333

Feature ID	Experiment - Range (original values)	Experiment - IQR (original values)	Experiment - Difference (original values)	Experiment - Fold Change (original values)	EDGE test: yccT H202 vs yccT NT , tagwise dispersions - P-value	EDGE test: yccT H202 vs yccT NT , tagwise dispersions - Fold change	EDGE test: yccT H202 vs yccT NT , tagwise dispersions - Weighted difference	EDGE test: yccT H202 vs yccT NT , tagwise dispersions - Bonferroni	EDGE test: yccT H202 vs yccT NT , tagwise dispersions - FDR p-value correction	yccT NT - yccT.1.S28 - Expression values	yccT NT - yccT.2.S29 - Expression values	yccT NT - yccT.3.S30 - Expression values	yccT NT - Means	yccT H202 - yccT.1.H2O2 - Expression values	yccT H202 - yccT.2.H2O2 - Expression values	yccT H202 - yccT.3.H2O2 - Expression values	yccT H202 - Means
SEN0722	29	8	13.66667	1.394231	4.81E-11	3.284858	6.19E-06	2.13E-07	2.74E-10	40	33	31	34.66667	41	60	44	48.33333
SEN0735	3488	2818	-3193.67	-6.19014	2.64E-08	-2.63054	-0.00018	0.000117	1.1E-07	4007	3401	4019	3809	531	583	732	615.3333
SEN0744A	23	4	10	2.666667	1.11E-08	6.157895	2.48E-06	4.93E-05	4.86E-08	9	7	2	6	11	25	12	16
SEN0764	840	757	-786.667	-5.96842	6.12E-12	-2.52734	-4.4E-05	2.71E-08	3.85E-11	991	918	926	945	151	161	163	158.3333
SEN0801	36	4	9.666667	1.193333	1.34E-08	2.805758	7.07E-06	5.93E-05	5.74E-08	41	51	58	50	49	77	53	59.66667
SEN0802	138	101	112.3333	2.811828	9.1E-38	6.605141	2.72E-05	4.03E-34	2.69E-36	63	66	57	62	164	164	195	174.3333
SEN0803	58	35	40.66667	1.382445	5.03E-16	3.263708	1.88E-05	2.23E-12	4.61E-15	96	101	122	106.3333	151	154	136	147
SEN0804	59	18	31	1.273529	8.97E-14	3.009225	1.78E-05	3.98E-10	6.77E-13	106	115	119	113.3333	165	133	135	144.3333
SEN0806	157	77	105	1.367561	6.34E-17	3.234677	4.97E-05	2.81E-13	6.3E-16	285	266	306	285.6667	423	362	387	390.6667
SEN0814	463	385	-409.333	-6.60731	2.16E-13	-2.80032	-2.4E-05	9.58E-10	1.58E-12	528	455	464	482.3333	65	70	84	73
SEN0815	232	169	-203	-6.4375	2.25E-10	-2.72118	-1.2E-05	9.97E-07	1.2E-09	250	210	261	240.3333	41	29	42	37.33333
SEN0816	624	136	-114.667	-1.15896	0.000314	2.017513	6.63E-05	1	0.000733	851	834	823	836	698	421	1045	721.3333
SEN0829	23	5	-3.66667	-1.10185	5.91E-05	2.133936	3.51E-06	0.261946	0.000155	48	36	35	39.66667	25	40	43	36
SEN0854	360	92	-101.667	-1.14166	1.73E-05	2.086137	6.92E-05	0.076556	4.93E-05	894	736	828	819.3333	739	880	534	717.6667
SEN0893	3604	3256	-3419.33	-12.3098	0	-5.23155	-0.00023	0	0	3847	3760	3558	3721.667	302	243	362	302.3333
SEN0905	416	366	-387.667	-8.75333	2.64E-17	-3.72257	-2.5E-05	1.17E-13	2.7E-16	456	447	410	437.6667	44	40	66	50
SEN0906	11	6	-6.66667	-1.16529	2.23E-05	2.020386	3.75E-06	0.098719	6.21E-05	47	47	47	47	36	44	41	40.33333
SEN0912	37	14	25.66667	3.333333	4.07E-18	7.806246	5.92E-06	1.8E-14	4.36E-17	12	8	13	11	39	45	26	36.66667
SEN0912A	96	25	51	1.9	1.17E-16	4.499959	1.55E-05	5.18E-13	1.14E-15	61	44	65	56.66667	97	140	86	107.6667
SEN0914	10	4	-1.66667	-1.12821	0.006997	2.07307	1.24E-06	1	0.0127	15	13	16	14.66667	9	11	19	13
SEN0916A	160	35	76.33333	1.88417	1.71E-14	4.452413	2.32E-05	7.56E-11	1.37E-13	94	85	80	86.33333	120	240	128	162.6667
SEN0917	42	2	14.33333	1.234973	4.03E-11	2.923646	9.13E-06	1.79E-07	2.31E-10	69	43	71	61	72	85	69	75.33333
SEN0920	80	18	29.33333	1.14966	3.43E-12	2.697562	2.6E-05	1.52E-08	2.21E-11	190	208	190	196	208	198	270	225.3333
SEN0921	37	25	30.33333	6.352941	3.45E-28	14.57875	6.18E-06	1.53E-24	6.6E-27	6	5	6	5.666667	31	42	35	36
SEN0957	441	339	-389.333	-84.4286	1.85E-65	-35.2815	-3E-05	8.21E-62	9.12E-64	396	342	444	394	3	3	8	4.666667
SEN0973	81	48	64.66667	1.259705	1.93E-15	2.978306	3.84E-05	8.55E-12	1.67E-14	249	245	253	249	326	318	297	313.6667
SEN0988	1241	1005	-1101	-7.197	2.77E-11	-3.06854	-6.7E-05	1.23E-07	1.63E-10	1304	1170	1362	1278.667	165	121	247	177.6667
SEN0990	1202	631	-871.333	-6.249	0.000892	-2.67928	-5.1E-05	1	0.001933	1043	1244	825	1037.333	194	42	262	166
SEN0992	299	50	142.6667	2.226361	5.17E-07	5.211906	3.84E-05	0.002291	1.81E-06	107	146	96	116.3333	359	60	358	259
SEN0993	96	5	31	1.5	4.13E-08	3.506686	1.21E-05	0.000183	1.68E-07	63	59	64	62	97	43	139	93

Feature ID	Experiment - Range (original values)	Experiment - IQR (original values)	Experiment - Difference (original values)	Experiment - Fold Change (original values)	EDGE test: yccT H202 vs yccT NT , tagwise dispersions - P-value	EDGE test: yccT H202 vs yccT NT , tagwise dispersions - Fold change	EDGE test: yccT H202 vs yccT NT , tagwise dispersions - Weighted difference	EDGE test: yccT H202 vs yccT NT , tagwise dispersions - Bonferroni	EDGE test: yccT H202 vs yccT NT , tagwise dispersions - FDR p-value correction	yccT NT - yccT.1.S28 - Expression values	yccT NT - yccT.2.S29 - Expression values	yccT NT - yccT.3.S30 - Expression values	yccT NT - Means	yccT H202 - yccT.1.H2O2 - Expression values	yccT H202 - yccT.2.H2O2 - Expression values	yccT H202 - yccT.3.H2O2 - Expression values	yccT H202 - Means
SEN0994	1419	62	826.3333	3.6829	9.78E-14	8.632755	0.000183	4.33E-10	7.35E-13	341	324	259	308	1339	386	1678	1134.333
SEN0996	45	20	-2.66667	-1.02204	4.21E-08	2.306731	1.26E-05	0.000187	1.71E-07	122	109	140	123.6667	129	95	139	121
SEN0998	5	4	2.666667	1.296296	0.000116	3.024584	1.44E-06	0.513841	0.00029	12	8	7	9	12	12	11	11.66667
SEN0999	9	5	1	1.1875	0.015067	2.733641	7.45E-07	1	0.025504	5	10	1	5.333333	3	8	8	6.333333
SEN1000	8	3	4.666667	1.388889	4.42E-06	3.253429	2.13E-06	0.019571	1.35E-05	14	11	11	12	18	19	13	16.66667
SEN1001	40	16	1.666667	1.032468	1.61E-05	2.406307	5.66E-06	0.071226	4.6E-05	43	52	59	51.33333	30	59	70	53
SEN1002	16	3	-2	-1.11111	0.002845	2.117505	1.76E-06	1	0.005571	18	23	19	20	16	27	11	18
SEN1005	11	2	2.333333	1.145833	4.63E-05	2.6832	2.12E-06	0.205309	0.000123	16	20	12	16	15	23	17	18.33333
SEN1007	11	5	2.333333	1.28	0.00033	2.987671	1.31E-06	1	0.000768	15	6	4	8.333333	10	11	11	10.66667
SEN1008	9	5	6.333333	2.117647	5.94E-07	4.881456	1.77E-06	0.002631	2.06E-06	5	6	6	5.666667	14	11	11	12
SEN1009	16	5	11.33333	2.7	9.76E-11	6.215379	2.78E-06	4.33E-07	5.37E-10	5	8	7	6.666667	12	21	21	18
SEN1013	45	18	27.33333	1.288732	2.79E-15	3.033789	1.5E-05	1.24E-11	2.39E-14	101	92	91	94.66667	110	120	136	122
SEN1013A	6	1	3	3.25	0.000745	6.899191	6.93E-07	1	0.001641	1	1	2	1.333333	7	1	5	4.333333
SEN1013B	34	9	19	4	6.01E-13	9.302068	4.19E-06	2.66E-09	4.23E-12	10	5	4	6.333333	24	38	14	25.33333
SEN1016	226	97	161.6667	2.328767	4.71E-27	5.497759	4.26E-05	2.09E-23	8.81E-26	139	105	121	121.6667	301	218	331	283.3333
SEN1022	118	43	3.666667	1.006358	1.27E-09	2.370545	6.16E-05	5.64E-06	6.25E-09	593	590	547	576.6667	573	525	643	580.3333
SEN1026	60	13	-6	-1.04545	6.28E-08	2.249555	1.34E-05	0.000278	2.48E-07	161	131	122	138	118	109	169	132
SEN1063	62	43	-52	-6.37931	1.49E-05	-2.70478	-3E-06	0.065877	4.27E-05	52	66	67	61.66667	9	5	15	9.666667
SEN1065	570	517	-543.333	-9.62434	3.7E-20	-4.07404	-3.6E-05	1.64E-16	4.55E-19	614	587	618	606.3333	71	48	70	63
SEN1107	27	11	17.33333	2.130435	3.31E-14	4.974798	4.8E-06	1.47E-10	2.6E-13	16	19	11	15.33333	33	27	38	32.66667
SEN1133	235	117	-56.6667	-1.07293	4.74E-07	2.20707	7.84E-05	0.002099	1.66E-06	872	790	839	833.6667	722	922	687	777
SEN1134	427	353	-380.667	-5.02113	2.09E-06	-2.13692	-2E-05	0.009256	6.72E-06	490	443	493	475.3333	90	66	128	94.66667
SEN1140	2396	2303	-2331.67	-10.1678	0	-4.32567	-0.00016	0	0	2588	2619	2551	2586	223	248	292	254.3333
SEN1141	125	105	-112.333	-5.61644	1.65E-07	-2.37948	-6.2E-06	0.000732	6.15E-07	130	147	133	136.6667	26	22	25	24.33333
SEN1142	429	360	-382	-8.34615	5.41E-17	-3.53294	-2.4E-05	2.4E-13	5.4E-16	417	409	476	434	47	60	49	52
SEN1155	23	13	-4	-1.11215	0.000229	2.121838	3.47E-06	1	0.000547	42	29	48	39.66667	26	49	32	35.66667
SEN1156	6	1	2.333333	8	0.002548	12.6695	4.63E-07	1	0.005057	0	1	0	0.333333	2	0	6	2.666667
SEN1157	6	1	3.333333	1.47619	9.49E-05	3.423271	1.35E-06	0.420699	0.000242	7	8	6	7	12	8	11	10.33333
SEN1162	9	4	3.333333	1.263158	2.07E-05	2.953173	1.95E-06	0.091936	5.83E-05	9	11	18	12.66667	15	18	15	16
SEN1163	41	20	29.33333	4.259259	8.38E-24	9.859207	6.33E-06	3.71E-20	1.28E-22	12	10	5	9	30	39	46	38.33333

Feature ID	Experiment - Range (original values)	Experiment - IQR (original values)	Experiment - Difference (original values)	Experiment - Fold Change (original values)	EDGE test: yccT H202 vs yccT NT , tagwise dispersions - P-value	EDGE test: yccT H202 vs yccT NT , tagwise dispersions - Fold change	EDGE test: yccT H202 vs yccT NT , tagwise dispersions - Weighted difference	EDGE test: yccT H202 vs yccT NT , tagwise dispersions - Bonferroni	EDGE test: yccT H202 vs yccT NT , tagwise dispersions - FDR p-value correction	yccT NT - yccT.1.S28 - Expression values	yccT NT - yccT.2.S29 - Expression values	yccT NT - yccT.3.S30 - Expression values	yccT NT - Means	yccT H202 - yccT.1.H2O2 - Expression values	yccT H202 - yccT.2.H2O2 - Expression values	yccT H202 - yccT.3.H2O2 - Expression values	yccT H202 - Means
SEN1171A	26	3	8.666667	1.440678	4.24E-07	3.389245	3.69E-06	0.00188	1.5E-06	23	17	19	19.66667	20	43	22	28.33333
SEN1181	159	98	-121.667	-15.6	3.62E-16	-6.60885	-8.7E-06	1.6E-12	3.37E-15	107	165	118	130	6	10	9	8.333333
SEN1183	252	142	199.3333	2.728324	5.91E-35	6.417486	4.88E-05	2.62E-31	1.52E-33	117	118	111	115.3333	322	259	363	314.6667
SEN1193	971	314	198	1.104137	4.14E-07	2.600669	0.000237	0.001835	1.47E-06	2057	1743	1904	1901.333	2101	1613	2584	2099.333
SEN1201	870	706	-766.333	-6.06388	3.18E-11	-2.5756	-4.4E-05	1.41E-07	1.85E-10	987	872	894	917.6667	117	166	171	151.3333
SEN1204	363	275	-317.333	-11.1277	1.04E-13	-4.71504	-2.1E-05	4.62E-10	7.81E-13	375	312	359	348.6667	37	12	45	31.33333
SEN1228	553	37	159.6667	1.198838	1.25E-08	2.834529	0.000115	5.55E-05	5.41E-08	815	842	752	803	805	1305	778	962.6667
SEN1229	425	277	342.3333	2.837209	1.19E-36	6.682143	8.26E-05	5.26E-33	3.31E-35	177	175	207	186.3333	454	600	532	528.6667
SEN1244	75	21	-3	-1.01875	7.92E-08	2.311026	1.67E-05	0.000351	3.08E-07	171	168	150	163	173	116	191	160
SEN1245	38	12	-6.66667	-1.0565	2.71E-08	2.232828	1.2E-05	0.00012	1.12E-07	123	113	138	124.6667	125	100	129	118
SEN1294	1180	961	-1067.67	-6.3206	2.96E-11	-2.67108	-6.2E-05	1.31E-07	1.73E-10	1354	1148	1303	1268.333	174	241	187	200.6667
SEN1301	557	479	-506	-6.70677	1.91E-13	-2.85054	-3E-05	8.47E-10	1.4E-12	629	564	591	594.6667	72	85	109	88.66667
SEN1303	4536	3645	-4146.67	-9.05178	9.37E-13	-3.81005	-0.00027	4.15E-09	6.47E-12	4847	4132	5006	4661.667	588	487	470	515
SEN1335	30	10	17.66667	1.519608	1.97E-12	3.578343	6.87E-06	8.72E-09	1.32E-11	33	38	31	34	51	61	43	51.66667
SEN1336	1393	1114	-1193.33	-4.89554	0.000199	-2.10017	-6.1E-05	0.881725	0.000481	1573	1548	1378	1499.667	264	180	475	306.3333
SEN1358	382	336	355.3333	5.696035	2.33E-85	13.42428	7.33E-05	1.03E-81	1.69E-83	75	65	87	75.66667	411	435	447	431
SEN1359	265	137	178.6667	2.042802	1.35E-24	4.845608	5.12E-05	5.98E-21	2.19E-23	196	153	165	171.3333	330	418	302	350
SEN1360	245	64	122.3333	1.755144	4.95E-16	4.152999	3.98E-05	2.2E-12	4.55E-15	177	151	158	162	222	396	235	284.3333
SEN1360A	1762	536	971.6667	1.906687	5.34E-13	4.513838	0.000293	2.37E-09	3.79E-12	1083	1048	1084	1071.667	1701	2810	1619	2043.333
SEN1361	16	2	6.666667	1.909091	1.23E-06	4.430306	2.01E-06	0.005465	4.11E-06	4	8	10	7.333333	20	10	12	14
SEN1362	12	5	7	2.3125	2.01E-07	5.32921	1.86E-06	0.000891	7.45E-07	5	4	7	5.333333	16	11	10	12.33333
SEN1364	10	0	4	1.461538	3.21E-05	3.3968	1.65E-06	0.142359	8.76E-05	10	10	6	8.666667	12	10	16	12.66667
SEN1365	24	8	3.333333	1.063291	4.66E-08	2.506533	6.2E-06	0.000206	1.87E-07	46	44	68	52.66667	54	61	53	56
SEN1381	5	3	2	2.5	0.005595	5.322345	5.08E-07	1	0.010338	0	0	4	1.333333	2	3	5	3.333333
SEN1386	557	446	-488.333	-7.31466	1.84E-12	-3.10913	-3E-05	8.17E-09	1.24E-11	556	611	530	565.6667	84	54	94	77.33333
SEN1387	55487	26947	41444.67	3.767467	3.04E-23	8.853535	0.009169	1.35E-19	4.47E-22	14490	14411	16026	14975.67	57926	41437	69898	56420.33
SEN1392	51	7	28	1.360515	1.16E-12	3.193444	1.33E-05	5.12E-09	7.92E-12	77	74	82	77.66667	75	125	117	105.6667
SEN1398	125	100	-104.333	-6.39655	8.23E-08	-2.71865	-6.1E-06	0.000365	3.19E-07	115	117	139	123.6667	15	14	29	19.33333
SEN1399	759	691	-710	-12.0363	5.19E-26	-5.11641	-4.9E-05	2.3E-22	9.06E-25	762	753	808	774.3333	62	49	82	64.33333
SEN1409	181	23	80.33333	1.416235	3.72E-14	3.311967	3.48E-05	1.65E-10	2.9E-13	194	193	192	193	231	216	373	273.3333

Feature ID	Experiment - Range (original values)	Experiment - IQR (original values)	Experiment - Difference (original values)	Experiment - Fold Change (original values)	EDGE test: yccT H202 vs yccT NT , tagwise dispersions - P-value	EDGE test: yccT H202 vs yccT NT , tagwise dispersions - Fold change	EDGE test: yccT H202 vs yccT NT , tagwise dispersions - Weighted difference	EDGE test: yccT H202 vs yccT NT , tagwise dispersions - Bonferroni	EDGE test: yccT H202 vs yccT NT , tagwise dispersions - FDR p-value correction	yccT NT - yccT.1.S28 - Expression values	yccT NT - yccT.2.S29 - Expression values	yccT NT - yccT.3.S30 - Expression values	yccT NT - Means	yccT H202 - yccT.1.H2O2 - Expression values	yccT H202 - yccT.2.H2O2 - Expression values	yccT H202 - yccT.3.H2O2 - Expression values	yccT H202 - Means
SEN1410	1873	317	1017.333	5.986928	2.97E-21	13.94038	0.000205	1.32E-17	3.89E-20	228	184	200	204	1090	517	2057	1221.333
SEN1415	29	15	19	1.74026	6.5E-14	4.074005	6.21E-06	2.88E-10	4.97E-13	17	32	28	25.66667	45	43	46	44.66667
SEN1417	43	5	23.66667	2.365385	1.76E-11	5.566664	6.22E-06	7.82E-08	1.06E-10	19	17	16	17.33333	59	22	42	41
SEN1418	61	26	46.66667	3.058824	1.32E-22	7.155441	1.1E-05	5.87E-19	1.88E-21	19	26	23	22.66667	79	49	80	69.33333
SEN1419	18	4	9.333333	1.491228	2.42E-09	3.489905	3.72E-06	1.07E-05	1.15E-08	15	19	23	19	29	23	33	28.33333
SEN1423	15	9	11.66667	3.916667	5E-12	8.904294	2.57E-06	2.22E-08	3.18E-11	6	4	2	4	17	13	17	15.66667
SEN1423A	59	12	36.33333	1.832061	1.76E-15	4.348947	1.14E-05	7.79E-12	1.52E-14	48	35	48	43.66667	94	86	60	80
SEN1424	81	31	50.66667	2.56701	1.39E-18	6.083061	1.28E-05	6.14E-15	1.56E-17	44	25	28	32.33333	106	59	84	83
SEN1425	585	207	346.3333	2.048436	1.47E-18	4.88104	9.95E-05	6.51E-15	1.65E-17	403	299	289	330.3333	650	874	506	676.6667
SEN1428	718	616	-645	-8.99587	1.56E-11	-3.82696	-4.2E-05	6.91E-08	9.43E-11	757	714	706	725.6667	90	39	113	80.66667
SEN1429	251	195	-227	-4.84746	2.71E-07	-2.05002	-1.1E-05	0.0012	9.88E-07	305	255	298	286	54	63	60	59
SEN1433	59	34	5.666667	1.064639	1.1E-06	2.523926	1.04E-05	0.004888	3.7E-06	102	68	93	87.66667	117	58	105	93.33333
SEN1434	23	8	5.333333	1.122137	1.32E-08	2.641265	5.59E-06	5.85E-05	5.67E-08	53	42	36	43.66667	48	40	59	49
SEN1435	101	16	47	1.444795	3.01E-11	3.397647	1.98E-05	1.33E-07	1.76E-10	112	109	96	105.6667	173	92	193	152.6667
SEN1436	386	78	242	2.207987	4.53E-19	5.210029	6.55E-05	2.01E-15	5.19E-18	233	176	192	200.3333	495	270	562	442.3333
SEN1439	206	157	-170.667	-5.65455	3.03E-08	-2.40027	-9.4E-06	0.000134	1.25E-07	191	193	238	207.3333	34	32	44	36.66667
SEN1441	372	338	-348	-6.67391	2.92E-13	-2.83492	-2.1E-05	1.29E-09	2.1E-12	407	396	425	409.3333	53	58	73	61.33333
SEN1442	675	581	-615	-16.6356	1.56E-33	-7.02621	-4.4E-05	6.9E-30	3.73E-32	704	624	635	654.3333	46	29	43	39.33333
SEN1443	178	161	-169.333	-19.1429	3.09E-27	-8.07158	-1.2E-05	1.37E-23	5.8E-26	181	185	170	178.6667	9	7	12	9.333333
SEN1444	355	341	-343	-8.62222	5.45E-18	-3.66527	-2.2E-05	2.42E-14	5.75E-17	383	389	392	388	37	42	56	45
SEN1469	4832	1457	2621	9.199166	3.24E-24	21.76215	0.000518	1.44E-20	5.11E-23	302	330	327	319.6667	1904	5134	1784	2940.667
SEN1470	31	11	19.66667	1.728395	3.96E-15	4.052916	6.46E-06	1.75E-11	3.33E-14	30	29	22	27	40	47	53	46.66667
SEN1472	1242	89	487	1.484256	4.4E-09	3.51458	0.000197	1.95E-05	2E-08	1038	1019	960	1005.667	1168	2202	1108	1492.667
SEN1482	887	733	-831.667	-19.7594	6.7E-43	-8.3518	-6E-05	2.97E-39	2.39E-41	926	778	924	876	45	39	49	44.33333
SEN1493	800	140	-18.3333	-1.02922	0.001364	2.30667	6.6E-05	1	0.002846	626	744	567	645.6667	486	1098	298	627.3333
SEN1500	1049	894	-934	-7	3.51E-10	-2.97875	-5.6E-05	1.56E-06	1.83E-09	1145	1068	1056	1089.667	162	96	209	155.6667
SEN1512	345	32	31.66667	1.063376	1.01E-07	2.514015	5.89E-05	0.000449	3.87E-07	536	483	480	499.6667	399	744	451	531.3333
SEN1521	52	37	-42.6667	-9	4E-07	-3.76323	-2.7E-06	0.001774	1.42E-06	56	41	47	48	8	4	4	5.333333
SEN1523	15	3	4.666667	1.14	8.06E-08	2.692603	4.41E-06	0.000357	3.13E-07	38	31	31	33.33333	46	34	34	38
SEN1538	57	13	33.66667	1.980583	8.1E-15	4.686606	9.9E-06	3.59E-11	6.63E-14	39	35	29	34.33333	70	86	48	68

Feature ID	Experiment - Range (original values)	Experiment - IQR (original values)	Experiment - Difference (original values)	Experiment - Fold Change (original values)	EDGE test: yccT H202 vs yccT NT , tagwise dispersions - P-value	EDGE test: yccT H202 vs yccT NT , tagwise dispersions - Fold change	EDGE test: yccT H202 vs yccT NT , tagwise dispersions - Weighted difference	EDGE test: yccT H202 vs yccT NT , tagwise dispersions - Bonferroni	EDGE test: yccT H202 vs yccT NT , tagwise dispersions - FDR p-value correction	yccT NT - yccT.1.S28 - Expression values	yccT NT - yccT.2.S29 - Expression values	yccT NT - yccT.3.S30 - Expression values	yccT NT - Means	yccT H202 - yccT.1.H2O2 - Expression values	yccT H202 - yccT.2.H2O2 - Expression values	yccT H202 - yccT.3.H2O2 - Expression values	yccT H202 - Means
SEN1543A	81	14	43	1.383929	4.61E-14	3.28563	1.99E-05	2.04E-10	3.55E-13	124	102	110	112	160	183	122	155
SEN1557	502	418	-443.333	-6.9375	3.01E-13	-2.94995	-2.7E-05	1.33E-09	2.16E-12	503	484	567	518	66	65	93	74.66667
SEN1558	2113	1851	-1977.67	-31.901	2.05E-76	-13.4948	-0.00015	9.09E-73	1.23E-74	2170	1916	2039	2041.667	57	70	65	64
SEN1564A	45	10	-9.33333	-1.09241	1.24E-06	2.148721	9.89E-06	0.005491	4.13E-06	115	111	105	110.3333	71	116	116	101
SEN1568	410	289	-349.333	-5.15873	2.92E-08	-2.17928	-1.8E-05	0.000129	1.2E-07	491	370	439	433.3333	81	81	90	84
SEN1635	13	2	0.666667	1.032787	7.53E-05	2.423536	2.27E-06	0.333459	0.000194	20	14	27	20.33333	19	18	26	21
SEN1715	70	61	64.66667	2.716814	5.79E-33	6.391582	1.59E-05	2.57E-29	1.34E-31	36	36	41	37.66667	106	97	104	102.3333
SEN1726	684	30	308.6667	1.925075	1.78E-12	4.495609	9.08E-05	7.88E-09	1.2E-11	360	311	330	333.6667	591	341	995	642.3333
SEN1734	2517	399	1411.667	3.777049	5.81E-17	8.84334	0.00031	2.57E-13	5.79E-16	580	451	494	508.3333	1899	893	2968	1920
SEN1754	534	388	-451	-6.45565	3.46E-09	-2.70459	-2.6E-05	1.54E-05	1.61E-08	593	473	535	533.6667	104	85	59	82.66667
SEN1755	553	451	-483	-6.46792	1.97E-11	-2.72779	-2.8E-05	8.75E-08	1.18E-10	628	533	553	571.3333	82	108	75	88.33333
SEN1756	432	395	-406.333	-7.625	1.05E-11	-3.20271	-2.5E-05	4.63E-08	6.42E-11	476	454	473	467.6667	81	59	44	61.33333
SEN1757	291	259	-271.333	-5.35294	1.16E-08	-2.26243	-1.5E-05	5.13E-05	5.04E-08	350	332	319	333.6667	68	59	60	62.33333
SEN1778	23	5	10.33333	1.449275	1.56E-09	3.386215	4.31E-06	6.92E-06	7.61E-09	22	25	22	23	28	27	45	33.33333
SEN1786	1291	1073	-1151.33	-8.74439	3.46E-07	-3.7554	-7.4E-05	0.001534	1.25E-06	1340	1342	1218	1300	145	51	250	148.6667
SEN1790	16	5	3.333333	1.039683	7.89E-10	2.456389	9.55E-06	3.5E-06	3.96E-09	87	86	79	84	95	86	81	87.33333
SEN1800	14185	4399	8194.667	7.153692	2.44E-22	16.91592	0.00166	1.08E-18	3.4E-21	1185	1600	1210	1331.667	7600	15370	5609	9526.333
SEN1801	27724	9088	16089.67	39.30873	8.86E-42	93.49308	0.003023	3.92E-38	3.09E-40	459	398	403	420	11916	28122	9491	16509.67
SEN1805	230	184	-202.667	-19.4242	2.68E-28	-8.22722	-1.5E-05	1.19E-24	5.13E-27	196	210	235	213.6667	5	12	16	11
SEN1809	13	4	-1.66667	-1.1087	0.002888	2.110594	1.49E-06	1	0.005648	14	15	22	17	9	19	18	15.33333
SEN1821	2662	2330	-2495.67	-11.6349	0	-4.9173	-0.00017	0	0	2867	2544	2780	2730.333	205	285	214	234.6667
SEN1861	631	490	-559.667	-15.7281	7.59E-27	-6.69724	-4E-05	3.36E-23	1.4E-25	614	523	656	597.6667	25	33	56	38
SEN1890	26	4	2.666667	1.094118	2.57E-05	2.565638	3.47E-06	0.113971	7.1E-05	31	27	27	28.33333	17	43	33	31
SEN1917	60	7	27.66667	2.66	5.29E-11	6.137865	6.74E-06	2.34E-07	3E-10	16	16	18	16.66667	34	23	76	44.33333
SEN1918	44	9	21.33333	2.122807	2.54E-08	4.935908	5.9E-06	0.000112	1.06E-07	11	19	27	19	48	18	55	40.33333
SEN1919	205	35	79.33333	1.504237	1.01E-10	3.523058	3.09E-05	4.47E-07	5.54E-10	176	155	141	157.3333	231	137	342	236.6667
SEN1971	3673	398	632	1.208535	2.09E-05	2.848561	0.000436	0.092396	5.85E-05	3250	3120	2722	3030.667	2111	5784	3093	3662.667
SEN1975	409	300	-340.333	-5.72685	1.22E-08	-2.4452	-1.9E-05	5.4E-05	5.28E-08	405	466	366	412.3333	57	66	93	72
SEN1977	147	89	112.6667	2.953757	9.44E-36	6.962567	2.68E-05	4.18E-32	2.55E-34	60	50	63	57.66667	149	197	165	170.3333
SEN1978	10	5	5.666667	2.416667	1.92E-06	5.494083	1.46E-06	0.00851	6.22E-06	3	7	2	4	9	8	12	9.666667

Feature ID	Experiment - Range (original values)	Experiment - IQR (original values)	Experiment - Difference (original values)	Experiment - Fold Change (original values)	EDGE test: yccT H202 vs yccT NT , tagwise dispersions - P-value	EDGE test: yccT H202 vs yccT NT , tagwise dispersions - Fold change	EDGE test: yccT H202 vs yccT NT , tagwise dispersions - Weighted difference	EDGE test: yccT H202 vs yccT NT , tagwise dispersions - Bonferroni	EDGE test: yccT H202 vs yccT NT , tagwise dispersions - FDR p-value correction	yccT NT - yccT.1.S28 - Expression values	yccT NT - yccT.2.S29 - Expression values	yccT NT - yccT.3.S30 - Expression values	yccT NT - Means	yccT H202 - yccT.1.H2O2 - Expression values	yccT H202 - yccT.2.H2O2 - Expression values	yccT H202 - yccT.3.H2O2 - Expression values	yccT H202 - Means
SEN1979	9	4	3.666667	2.222222	0.000313	5.01796	9.96E-07	1	0.000731	1	6	2	3	8	10	2	6.666667
SEN1980	117	84	94.66667	1.956229	5.09E-26	4.611173	2.79E-05	2.26E-22	8.92E-25	98	103	96	99	182	213	186	193.6667
SEN1981	164	16	67.66667	1.600592	2.86E-12	3.793255	2.45E-05	1.27E-08	1.87E-11	128	116	94	112.6667	151	258	132	180.3333
SEN1981A	14	7	8.666667	1.702703	3.7E-09	3.973842	2.9E-06	1.64E-05	1.71E-08	10	11	16	12.33333	18	24	21	21
SEN1982	42	19	28.66667	2.409836	1.63E-19	5.624359	7.39E-06	7.22E-16	1.93E-18	20	21	20	20.33333	39	46	62	49
SEN1983	11	5	1.666667	1.208333	0.001764	2.819406	1.15E-06	1	0.0036	15	5	4	8	10	6	13	9.666667
SEN1984	9	0	4	1.461538	3.43E-05	3.39279	1.65E-06	0.152131	9.33E-05	9	9	8	8.666667	9	12	17	12.66667
SEN1985	208	54	119.3333	1.355865	1.49E-15	3.203606	5.77E-05	6.61E-12	1.3E-14	350	361	295	335.3333	503	404	457	454.6667
SEN1986	421	155	281	1.782004	9.51E-20	4.217858	9.02E-05	4.21E-16	1.14E-18	373	369	336	359.3333	757	524	640	640.3333
SEN1989	6	0	2.666667	2.6	0.000696	5.658145	6.68E-07	1	0.001539	1	2	2	1.666667	7	4	2	4.333333
SEN1992	6	2	0.333333	1.038462	0.006482	2.418234	9.77E-07	1	0.011858	8	11	7	8.666667	6	12	9	9
SEN1994	190	57	-31.3333	-1.13092	5.55E-05	2.060969	2.24E-05	0.245883	0.000146	267	305	240	270.6667	210	159	349	239.3333
SEN1996	4	2	0.333333	1.052632	0.015069	2.445997	7.32E-07	1	0.025504	9	5	5	6.333333	5	8	7	6.666667
SEN1998	11	2	0.333333	1.05	0.022846	2.439036	7.68E-07	1	0.037341	6	8	6	6.666667	3	14	4	7
SEN2003	3	1	0.333333	1.0625	0.019627	2.460956	6.26E-07	1	0.032584	6	5	5	5.333333	7	4	6	5.666667
SEN2004	27	5	7	1.132911	6.6E-09	2.677517	6.9E-06	2.93E-05	2.94E-08	57	47	54	52.66667	55	74	50	59.66667
SEN2005	15	4	4.333333	1.056769	1.98E-10	2.493817	8.9E-06	8.79E-07	1.06E-09	76	69	84	76.33333	80	83	79	80.66667
SEN2006	26	10	12.66667	1.14786	1.45E-12	2.708334	1.14E-05	6.43E-09	9.84E-12	94	78	85	85.66667	95	96	104	98.33333
SEN2130	64	28	-12	-1.07595	3.78E-07	2.205369	1.59E-05	0.001676	1.35E-06	202	141	167	170	139	197	138	158
SEN2133	35	22	24	1.525547	9.75E-16	3.599447	9.26E-06	4.32E-12	8.66E-15	57	40	40	45.66667	72	62	75	69.66667
SEN2135	12	3	7.333333	3.75	2.44E-08	8.361433	1.63E-06	0.000108	1.02E-07	3	2	3	2.666667	6	14	10	10
SEN2144	432	260	-113	-1.16432	0.000115	2.031811	6.44E-05	0.510977	0.000289	823	845	734	800.6667	563	966	534	687.6667
SEN2155	15	1	4	1.4	3.27E-05	3.258305	1.79E-06	0.144889	8.9E-05	10	5	15	10	11	11	20	14
SEN2168	186	44	-30.6667	-1.06327	1.5E-07	2.233864	4.94E-05	0.000664	5.61E-07	582	462	502	515.3333	458	591	405	484.6667
SEN2169	111	38	-7	-1.04357	8.23E-06	2.277811	1.66E-05	0.036456	2.44E-05	196	138	169	167.6667	131	231	120	160.6667
SEN2170	106	39	-11.3333	-1.05954	2.09E-06	2.235382	1.94E-05	0.009248	6.72E-06	225	196	184	201.6667	157	260	154	190.3333
SEN2171	126	19	28.33333	1.148342	3.54E-10	2.724985	2.56E-05	1.57E-06	1.84E-09	220	143	210	191	191	269	198	219.3333
SEN2172	32	8	6	1.144	1.57E-07	2.682623	5.47E-06	0.000695	5.86E-07	44	34	47	41.66667	41	36	66	47.66667
SEN2173	222	90	145.6667	2.456667	1.63E-26	5.786741	3.72E-05	7.2E-23	2.92E-25	119	86	95	100	244	185	308	245.6667
SEN2179	1416	1345	-1363.33	-14.8176	2.16E-29	-6.25183	-9.6E-05	9.57E-26	4.39E-28	1494	1441	1451	1462	122	78	96	98.66667

Feature ID	Experiment - Range (original values)	Experiment - IQR (original values)	Experiment - Difference (original values)	Experiment - Fold Change (original values)	EDGE test: yccT H202 vs yccT NT , tagwise dispersions - P-value	EDGE test: yccT H202 vs yccT NT , tagwise dispersions - Fold change	EDGE test: yccT H202 vs yccT NT , tagwise dispersions - Weighted difference	EDGE test: yccT H202 vs yccT NT , tagwise dispersions - Bonferroni	EDGE test: yccT H202 vs yccT NT , tagwise dispersions - FDR p-value correction	yccT NT - yccT.1.S28 - Expression values	yccT NT - yccT.2.S29 - Expression values	yccT NT - yccT.3.S30 - Expression values	yccT NT - Means	yccT H202 - yccT.1.H2O2 - Expression values	yccT H202 - yccT.2.H2O2 - Expression values	yccT H202 - yccT.3.H2O2 - Expression values	yccT H202 - Means
SEN2191	140	79	101.3333	2.9	9.6E-31	6.853081	2.44E-05	4.25E-27	2.04E-29	58	48	54	53.33333	143	188	133	154.6667
SEN2201	23	4	10	1.461538	1.02E-07	3.415553	4.1E-06	0.00045	3.88E-07	24	20	21	21.66667	17	40	38	31.66667
SEN2218	736	663	-695	-5.28131	3.18E-09	-2.23905	-3.7E-05	1.41E-05	1.49E-08	866	829	877	857.3333	166	141	180	162.3333
SEN2227	23	4	-3.66667	-1.11224	0.000272	2.114652	3.17E-06	1	0.000641	35	32	42	36.33333	22	45	31	32.66667
SEN2228	63	13	39	3.34	1.2E-15	7.892669	9.03E-06	5.32E-12	1.05E-14	20	18	12	16.66667	61	75	31	55.66667
SEN2237	162	18	70	2.122995	9.05E-09	5.078827	1.98E-05	4.01E-05	3.98E-08	63	53	71	62.33333	137	211	49	132.3333
SEN2256	7	2	0	-1	0.002153	2.348635	1.31E-06	1	0.004333	14	11	12	12.33333	17	10	10	12.33333
SEN2263	372	340	-357.333	-7.30588	3.28E-15	-3.09576	-2.2E-05	1.45E-11	2.77E-14	423	422	397	414	51	62	57	56.66667
SEN2269	507	396	-430.333	-10.563	8.71E-20	-4.50015	-2.9E-05	3.86E-16	1.05E-18	445	542	439	475.3333	43	35	57	45
SEN2282	1982	192	306.3333	1.358984	0.000425	3.238807	0.000149	1	0.000976	871	806	883	853.3333	679	2391	409	1159.667
SEN2284	97	27	-17.3333	-1.19403	0.00408	2.007503	8.34E-06	1	0.007765	127	83	110	106.6667	89	138	41	89.33333
SEN2322	13207	1904	7236.333	12.04784	4.68E-20	28.82168	0.001421	2.07E-16	5.72E-19	684	702	579	655	7300	13786	2588	7891.333
SEN2323	4800	239	2324.667	6.315549	2.35E-12	15.15855	0.000482	1.04E-08	1.55E-11	469	409	434	437.3333	2404	5209	673	2762
SEN2324	5151	134	2275	5.484231	1.67E-10	13.134	0.00048	7.41E-07	9.01E-10	539	501	482	507.3333	2079	5633	635	2782.333
SEN2325	550	7	132.6667	2.275641	0.000341	5.425546	3.59E-05	1	0.000793	105	102	105	104	98	581	31	236.6667
SEN2380	100	67	80.66667	7.205128	6.08E-53	16.73243	1.62E-05	2.7E-49	2.59E-51	9	16	14	13	91	81	109	93.66667
SEN2392	1818	1002	1386.333	2.307862	1.38E-19	5.437504	0.000366	6.11E-16	1.65E-18	1088	999	1093	1060	2432	2090	2817	2446.333
SEN2420	391	284	-323.667	-8.24627	1.52E-14	-3.49567	-2E-05	6.73E-11	1.22E-13	420	336	349	368.3333	29	53	52	44.66667
SEN2454	308	299	-301	-10.1212	1.3E-21	-4.2927	-2E-05	5.78E-18	1.76E-20	337	329	336	334	29	30	40	33
SEN2457	729	691	-698.333	-5.88345	2.23E-11	-2.49993	-3.9E-05	9.9E-08	1.32E-10	825	838	861	841.3333	134	132	163	143
SEN2465	304	47	-95.3333	-1.09368	8.83E-07	2.160013	0.0001	0.003913	3.01E-06	1216	1038	1085	1113	912	1100	1041	1017.667
SEN2471	300	105	150.6667	1.137637	1.17E-09	2.684055	0.000144	5.2E-06	5.79E-09	1166	1061	1057	1094.667	1216	1163	1357	1245.333
SEN2472	24	3	-8	-1.13714	1.82E-06	2.079492	5.58E-06	0.008071	5.93E-06	81	58	60	66.33333	57	61	57	58.33333
SEN2484	1305	1124	-1211	-9.86098	2.82E-20	-4.16199	-8E-05	1.25E-16	3.51E-19	1420	1365	1258	1347.667	134	161	115	136.6667
SEN2488	245	176	-207.667	-5.35664	1.62E-07	-2.26604	-1.1E-05	0.000716	6.03E-07	280	227	259	255.3333	51	35	57	47.66667
SEN2507	137	90	112.3333	3.067485	1.3E-37	7.224276	2.64E-05	5.74E-34	3.8E-36	61	50	52	54.33333	171	142	187	166.6667
SEN2508	20	6	9	1.375	5.88E-09	3.221387	4.19E-06	2.61E-05	2.64E-08	16	25	31	24	35	28	36	33
SEN2510	928	874	-890.667	-9.87708	9.85E-21	-4.18025	-5.9E-05	4.36E-17	1.25E-19	997	964	1012	991	84	127	90	100.3333
SEN2525	2252	263	-33.6667	-1.01077	7.36E-05	2.321841	0.000325	0.326216	0.00019	3227	2964	3290	3160.333	3186	1971	4223	3126.667
SEN2527	379	354	-367	-9.34091	2.82E-19	-3.94435	-2.4E-05	1.25E-15	3.28E-18	416	416	401	411	48	47	37	44

Feature ID	Experiment - Range (original values)	Experiment - IQR (original values)	Experiment - Difference (original values)	Experiment - Fold Change (original values)	EDGE test: yccT H202 vs yccT NT , tagwise dispersions - P-value	EDGE test: yccT H202 vs yccT NT , tagwise dispersions - Fold change	EDGE test: yccT H202 vs yccT NT , tagwise dispersions - Weighted difference	EDGE test: yccT H202 vs yccT NT , tagwise dispersions - Bonferroni	EDGE test: yccT H202 vs yccT NT , tagwise dispersions - FDR p-value correction	yccT NT - yccT.1.S28 - Expression values	yccT NT - yccT.2.S29 - Expression values	yccT NT - yccT.3.S30 - Expression values	yccT NT - Means	yccT H202 - yccT.1.H2O2 - Expression values	yccT H202 - yccT.2.H2O2 - Expression values	yccT H202 - yccT.3.H2O2 - Expression values	yccT H202 - Means
SEN2550	612	463	-541	-6.32131	6.03E-12	-2.68649	-3.1E-05	2.67E-08	3.79E-11	697	667	564	642.6667	85	101	119	101.6667
SEN2551	408	338	-363.333	-7.08939	1.86E-14	-2.99341	-2.2E-05	8.25E-11	1.49E-13	465	398	406	423	60	62	57	59.66667
SEN2553	40	1	19.66667	1.217712	1.24E-12	2.866597	1.32E-05	5.51E-09	8.47E-12	93	92	86	90.33333	92	126	112	110
SEN2554	21	1	11.66667	1.416667	7.61E-10	3.327982	5.11E-06	3.37E-06	3.82E-09	29	26	29	28	44	28	47	39.66667
SEN2560	825	724	-756.333	-5.25704	4.95E-07	-2.22154	-4E-05	0.002192	1.73E-06	970	875	957	934	145	237	151	177.6667
SEN2610	407	190	297.3333	1.584535	4.36E-18	3.745152	0.000109	1.93E-14	4.65E-17	545	464	517	508.6667	840	707	871	806
SEN2624	154	31	32	1.115802	1.56E-09	2.643564	3.54E-05	6.93E-06	7.61E-09	292	253	284	276.3333	265	407	253	308.3333
SEN2633	58	5	6	1.086124	4.33E-06	2.529442	8.33E-06	0.019194	1.33E-05	58	62	89	69.66667	57	56	114	75.66667
SEN2641	274	45	-14.3333	-1.06555	0.000836	2.182624	2.15E-05	1	0.001825	251	219	229	233	184	99	373	218.6667
SEN2644	1701	58	-58.3333	-1.01989	3.18E-05	2.300252	0.000303	0.141049	8.69E-05	2937	2879	3156	2990.667	2882	2107	3808	2932.333
SEN2647	254	188	223.6667	1.812349	5.72E-26	4.280492	7.04E-05	2.54E-22	9.95E-25	284	263	279	275.3333	513	467	517	499
SEN2648	148	107	125.6667	4.115702	2.11E-44	9.652527	2.73E-05	9.36E-41	7.73E-43	38	46	37	40.33333	168	145	185	166
SEN2649	108	89	96.33333	18	1.43E-74	41.23294	1.83E-05	6.33E-71	8.11E-73	6	3	8	5.666667	95	100	111	102
SEN2669	537	9	210	1.960366	5.06E-09	4.56614	6.08E-05	2.24E-05	2.3E-08	221	222	213	218.6667	435	157	694	428.6667
SEN2681	80	4	36	1.197802	2.76E-14	2.818569	2.58E-05	1.22E-10	2.18E-13	190	170	186	182	190	214	250	218
SEN2743	265	185	219.6667	2.204753	2.57E-30	5.209412	5.99E-05	1.14E-26	5.38E-29	186	188	173	182.3333	397	438	371	402
SEN2744	280	212	236.6667	1.753715	2.5E-24	4.133744	7.67E-05	1.11E-20	3.96E-23	320	317	305	314	538	529	585	550.6667
SEN2750	60	25	37.33333	1.571429	2.63E-17	3.690232	1.38E-05	1.17E-13	2.7E-16	53	67	76	65.33333	103	92	113	102.6667
SEN2753	3375	2619	-2794.33	-5.41675	0.000722	-2.32018	-0.00015	1	0.001592	3651	3387	3243	3427	624	276	998	632.6667
SEN2781	554	527	-526	-4.79327	2.87E-06	-2.02225	-2.6E-05	0.012699	9.04E-06	667	672	655	664.6667	170	118	128	138.6667
SEN2797	6	1	2	1.666667	0.004092	3.761626	6.83E-07	1	0.007786	2	4	3	3	4	3	8	5
SEN2804	5131	3868	-4482.33	-12.7956	1.55E-15	-5.44076	-0.00031	6.89E-12	1.35E-14	5374	4247	4966	4862.333	379	243	518	380
SEN2819A	486	410	-448.333	-9.15152	6.59E-17	-3.89591	-2.9E-05	2.92E-13	6.53E-16	465	520	525	503.3333	55	39	71	55
SEN2830	789	715	-758	-13.6333	2.03E-33	-5.7644	-5.3E-05	8.97E-30	4.83E-32	849	775	830	818	60	60	60	60
SEN2864	1101	854	-939	-13.3553	2.24E-31	-5.65428	-6.5E-05	9.93E-28	4.92E-30	1170	951	924	1015	70	69	89	76
SEN2868	48	7	20.33333	1.15099	3.34E-12	2.713223	1.8E-05	1.48E-08	2.17E-11	134	141	129	134.6667	140	177	148	155
SEN2869	55	36	40.66667	2.452381	8.79E-24	5.788427	1.05E-05	3.89E-20	1.34E-22	35	21	28	28	76	66	64	68.66667
SEN2870	73	35	47.66667	1.922581	3.25E-21	4.532941	1.42E-05	1.44E-17	4.23E-20	61	49	45	51.66667	84	118	96	99.33333
SEN2877	1306	503	801.3333	1.55125	1.03E-12	3.649553	0.0003	4.56E-09	7.09E-12	1569	1355	1437	1453.667	1940	2164	2661	2255
SEN2878	1850	843	1280.667	2.315753	5.18E-19	5.430209	0.000336	2.3E-15	5.91E-18	1022	1015	883	973.3333	1858	2171	2733	2254

Feature ID	Experiment - Range (original values)	Experiment - IQR (original values)	Experiment - Difference (original values)	Experiment - Fold Change (original values)	EDGE test: yccT H202 vs yccT NT , tagwise dispersions - P-value	EDGE test: yccT H202 vs yccT NT , tagwise dispersions - Fold change	EDGE test: yccT H202 vs yccT NT , tagwise dispersions - Weighted difference	EDGE test: yccT H202 vs yccT NT , tagwise dispersions - Bonferroni	EDGE test: yccT H202 vs yccT NT , tagwise dispersions - FDR p-value correction	yccT NT - yccT.1.S28 - Expression values	yccT NT - yccT.2.S29 - Expression values	yccT NT - yccT.3.S30 - Expression values	yccT NT - Means	yccT H202 - yccT.1.H2O2 - Expression values	yccT H202 - yccT.2.H2O2 - Expression values	yccT H202 - yccT.3.H2O2 - Expression values	yccT H202 - Means
SEN2879	31	14	18.33333	1.55	3.49E-13	3.65877	6.93E-06	1.55E-09	2.5E-12	39	31	30	33.33333	61	45	49	51.66667
SEN2914	270	197	-227.667	-12.0161	1.52E-19	-5.0657	-1.6E-05	6.76E-16	1.82E-18	243	216	286	248.3333	19	27	16	20.66667
SEN2915	380	320	-342.667	-8.90769	5.19E-19	-3.77431	-2.2E-05	2.3E-15	5.91E-18	375	365	418	386	38	47	45	43.33333
SEN2916	415	305	-360.333	-5.8914	1.94E-10	-2.49208	-2E-05	8.6E-07	1.04E-09	439	380	483	434	75	68	78	73.66667
SEN2923	24	17	-2.33333	-1.10769	0.00936	2.146624	2.16E-06	1	0.016524	33	30	9	24	29	24	12	21.66667
SEN2926	28	7	-5.66667	-1.09341	2.22E-06	2.149109	5.96E-06	0.009854	7.13E-06	57	64	78	66.33333	61	50	71	60.66667
SEN2935	214	177	-191.667	-6.37383	1.16E-09	-2.71648	-1.1E-05	5.14E-06	5.73E-09	212	239	231	227.3333	25	35	47	35.66667
SEN2962	273	242	-258.667	-5.26374	9.57E-09	-2.22533	-1.4E-05	4.24E-05	4.19E-08	331	303	324	319.3333	61	63	58	60.66667
SEN2974	266	231	-241.333	-11.1972	3.97E-20	-4.73721	-1.6E-05	1.76E-16	4.87E-19	254	286	255	265	28	20	23	23.66667
SEN2975	50	19	22.66667	1.194286	2.64E-13	2.821709	1.66E-05	1.17E-09	1.91E-12	130	111	109	116.6667	126	159	133	139.3333
SEN2980	2643	137	983.3333	1.455598	2.44E-07	3.452206	0.000412	0.001079	8.91E-07	2240	2103	2132	2158.333	4327	1684	3414	3141.667
SEN2984	36	12	-5	-1.03817	1.17E-08	2.278047	1.35E-05	5.17E-05	5.06E-08	149	128	131	136	119	155	119	131
SEN2997	1170	696	899.3333	1.855151	7.34E-17	4.365822	0.000276	3.25E-13	7.24E-16	1091	1025	1039	1051.667	1735	1923	2195	1951
SEN2998	760	669	-695.667	-10.4009	4.08E-19	-4.42924	-4.6E-05	1.81E-15	4.68E-18	810	761	738	769.6667	69	50	103	74
SEN2999	857	757	-783.333	-11.6818	6.61E-27	-4.95955	-5.3E-05	2.93E-23	1.23E-25	917	825	828	856.6667	68	60	92	73.33333
SEN3009	19	3	8.666667	1.245283	2.97E-10	2.928289	5.33E-06	1.32E-06	1.55E-09	36	31	39	35.33333	43	39	50	44
SEN3018	627	204	-36	-1.05628	0.000208	2.251505	6.57E-05	0.921415	0.000501	742	605	680	675.6667	476	1035	408	639.6667
SEN3034	571	478	-514.333	-5.57864	2.21E-10	-2.36169	-2.8E-05	9.79E-07	1.18E-09	673	594	613	626.6667	102	119	116	112.3333
SEN3093	358	256	-307	-5.47087	2.15E-08	-2.30475	-1.7E-05	9.52E-05	9.06E-08	417	323	387	375.6667	67	80	59	68.66667
SEN3094	383	315	-351.667	-20.9057	4E-39	-8.80762	-2.6E-05	1.77E-35	1.24E-37	397	377	334	369.3333	20	19	14	17.66667
SEN3095	1417	1144	-1237	-6.05586	3.49E-08	-2.55606	-7E-05	0.000155	1.43E-07	1584	1408	1453	1481.667	303	167	264	244.6667
SEN3097	1268	1244	-1238.33	-7.26476	3.55E-15	-3.08851	-7.6E-05	1.57E-11	2.99E-14	1432	1427	1449	1436	181	183	229	197.6667
SEN3112	15	3	2	1.09375	4.29E-05	2.578963	2.65E-06	0.190119	0.000115	20	24	20	21.33333	31	23	16	23.33333
SEN3135	405	315	-339.333	-5.73488	1.62E-09	-2.43509	-1.9E-05	7.19E-06	7.85E-09	463	388	382	411	58	67	90	71.66667
SEN3166	413	333	363	1.963717	5.85E-25	4.645327	0.000107	2.59E-21	9.67E-24	398	354	378	376.6667	767	711	741	739.6667
SEN3176	1570	1338	-1443.33	-14.1212	1.32E-15	-6.06529	-0.0001	5.87E-12	1.16E-14	1624	1605	1431	1553.333	93	54	183	110
SEN3184	41	30	33.33333	2.25	1.18E-22	5.280565	8.95E-06	5.21E-19	1.67E-21	24	25	31	26.66667	55	65	60	60
SEN3185	86	33	43	2.007813	2.73E-13	4.762307	1.25E-05	1.21E-09	1.98E-12	64	32	32	42.66667	65	118	74	85.66667
SEN3189	15	7	0	-1	2.53E-06	2.357329	3.74E-06	0.011211	8.04E-06	43	28	35	35.33333	29	41	36	35.33333
SEN3195	2970	528	1422	14.33125	1.7E-21	34.12264	0.000275	7.54E-18	2.29E-20	120	99	101	106.6667	888	3069	629	1528.667

Feature ID	Experiment - Range (original values)	Experiment - IQR (original values)	Experiment - Difference (original values)	Experiment - Fold Change (original values)	EDGE test: yccT H202 vs yccT NT , tagwise dispersions - P-value	EDGE test: yccT H202 vs yccT NT , tagwise dispersions - Fold change	EDGE test: yccT H202 vs yccT NT , tagwise dispersions - Weighted difference	EDGE test: yccT H202 vs yccT NT , tagwise dispersions - Bonferroni	EDGE test: yccT H202 vs yccT NT , tagwise dispersions - FDR p-value correction	yccT NT - yccT.1.S28 - Expression values	yccT NT - yccT.2.S29 - Expression values	yccT NT - yccT.3.S30 - Expression values	yccT NT - Means	yccT H202 - yccT.1.H2O2 - Expression values	yccT H202 - yccT.2.H2O2 - Expression values	yccT H202 - yccT.3.H2O2 - Expression values	yccT H202 - Means
SEN3273	5345	443	-524.333	-1.06499	0.000223	2.237036	0.000827	0.990328	0.000534	9275	8124	8378	8592.333	7935	10807	5462	8068
SEN3288	2384	2040	-2198.33	-6.48213	1.35E-08	-2.74382	-0.00013	6E-05	5.81E-08	2690	2719	2389	2599.333	335	519	349	401
SEN3343	37	13	16	1.036613	4.28E-11	2.444445	4.92E-05	1.9E-07	2.44E-10	434	432	445	437	429	466	464	453
SEN3346	1516	198	803.3333	4.549337	2.59E-18	10.51673	0.000168	1.15E-14	2.84E-17	251	211	217	226.3333	415	947	1727	1029.667
SEN3354	33	16	-8.66667	-1.08609	4.78E-07	2.171408	1E-05	0.002118	1.68E-06	110	124	94	109.3333	96	115	91	100.6667
SEN3370	519	51	304.6667	5.154545	5.4E-17	12.00315	6.32E-05	2.39E-13	5.4E-16	71	81	68	73.33333	425	122	587	378
SEN3371	1193	407	918.3333	11.51527	3.83E-38	27.11326	0.000178	1.7E-34	1.15E-36	94	92	76	87.33333	1269	499	1249	1005.667
SEN3372	173	54	121.6667	3.534722	1.8E-24	8.340779	2.75E-05	7.98E-21	2.89E-23	50	47	47	48	220	101	188	169.6667
SEN3373	106	25	56.66667	1.311355	8.76E-13	3.099363	2.97E-05	3.88E-09	6.08E-12	199	166	181	182	272	174	270	238.6667
SEN3381	687	384	519.6667	4.756627	1.51E-39	11.23784	0.000111	6.7E-36	4.93E-38	127	159	129	138.3333	647	814	513	658
SEN3421	1792	1267	-1544	-9.8906	9.22E-13	-4.1625	-0.0001	4.09E-09	6.39E-12	1831	1402	1920	1717.667	135	258	128	173.6667
SEN3422	3556	2373	-3145	-44.4793	1.84E-52	-18.7586	-0.00024	8.17E-49	7.78E-51	3613	2450	3589	3217.333	77	57	83	72.33333
SEN3423	5132	3879	-4653.67	-167.202	4.91E-90	-70.6403	-0.00036	2.18E-86	3.89E-88	5154	3906	4985	4681.667	27	22	35	28
SEN3424	10313	7782	-9220.33	-39.9044	0	-16.7661	-0.00069	0	0	10520	8028	9824	9457.333	258	246	207	237
SEN3447A	74	10	24.66667	1.173709	6.58E-13	2.765953	1.95E-05	2.91E-09	4.62E-12	147	117	162	142	152	157	191	166.6667
SEN3455	127	29	70.66667	1.397749	9.6E-16	3.289821	3.18E-05	4.25E-12	8.56E-15	165	182	186	177.6667	211	292	242	248.3333
SEN3456	37	14	-16.6667	-1.15723	1.26E-06	2.04553	1E-05	0.005591	4.18E-06	129	115	124	122.6667	106	120	92	106
SEN3476B	6	0	-1.33333	-1.10811	0.005771	2.111186	1.2E-06	1	0.010637	13	15	13	13.66667	9	13	15	12.33333
SEN3492	28	4	10	1.37037	4.01E-08	3.215076	4.69E-06	0.000178	1.63E-07	23	27	31	27	27	51	33	37
SEN3500	789	727	-745	-7.85583	1.94E-16	-3.33423	-4.7E-05	8.6E-13	1.86E-15	846	840	875	853.6667	113	86	127	108.6667
SEN3510	223	104	151	2.429022	1.57E-26	5.719048	3.9E-05	6.93E-23	2.82E-25	99	105	113	105.6667	209	322	239	256.6667
SEN3512	6	3	3	1.375	0.000177	3.191088	1.4E-06	0.785712	0.000432	7	10	7	8	9	11	13	11
SEN3513	217	43	-105.333	-1.14589	1.01E-06	2.055186	6.81E-05	0.004454	3.38E-06	799	813	870	827.3333	653	757	756	722
SEN3518	629	573	-588	-4.76119	5.7E-07	-2.02319	-2.9E-05	0.002524	1.98E-06	756	726	751	744.3333	153	127	189	156.3333
SEN3572	69	44	49.66667	2.146154	1.5E-24	5.033552	1.37E-05	6.67E-21	2.43E-23	43	52	35	43.33333	87	88	104	93
SEN3589	112	26	54.66667	1.504615	7.85E-12	3.545003	2.15E-05	3.48E-08	4.88E-11	126	104	95	108.3333	182	100	207	163
SEN3590	153	28	77.66667	1.618037	1.31E-11	3.812048	2.75E-05	5.81E-08	7.96E-11	144	131	102	125.6667	239	116	255	203.3333
SEN3591	187	13	121.6667	2.025281	2.4E-14	4.769947	3.49E-05	1.06E-10	1.91E-13	117	124	115	118.6667	302	130	289	240.3333
SEN3592	146	45	102	2.302128	1.93E-21	5.425771	2.7E-05	8.53E-18	2.55E-20	89	76	70	78.33333	204	121	216	180.3333
SEN3593	104	47	63	1.710526	1.76E-16	4.006992	2.09E-05	7.79E-13	1.69E-15	81	117	68	88.66667	155	128	172	151.6667

Feature ID	Experiment - Range (original values)	Experiment - IQR (original values)	Experiment - Difference (original values)	Experiment - Fold Change (original values)	EDGE test: yccT H202 vs yccT NT , tagwise dispersions - P-value	EDGE test: yccT H202 vs yccT NT , tagwise dispersions - Fold change	EDGE test: yccT H202 vs yccT NT , tagwise dispersions - Weighted difference	EDGE test: yccT H202 vs yccT NT , tagwise dispersions - Bonferroni	EDGE test: yccT H202 vs yccT NT , tagwise dispersions - FDR p-value correction	yccT NT - yccT.1.S28 - Expression values	yccT NT - yccT.2.S29 - Expression values	yccT NT - yccT.3.S30 - Expression values	yccT NT - Means	yccT H202 - yccT.1.H2O2 - Expression values	yccT H202 - yccT.2.H2O2 - Expression values	yccT H202 - yccT.3.H2O2 - Expression values	yccT H202 - Means
SEN3594	84	16	47.33333	2.067669	2.8E-15	4.82498	1.33E-05	1.24E-11	2.39E-14	35	54	44	44.33333	96	60	119	91.66667
SEN3595	1896	1605	-1710.67	-5.76067	7.2E-09	-2.43454	-9.5E-05	3.19E-05	3.19E-08	2222	2016	1972	2070	385	326	367	359.3333
SEN3597	679	552	-624	-5.33333	7.16E-09	-2.26142	-3.3E-05	3.17E-05	3.18E-08	800	801	703	768	151	122	159	144
SEN3614	573	91	133.3333	1.345722	2.81E-06	3.160358	6.48E-05	0.012464	8.9E-06	427	336	394	385.6667	586	199	772	519
SEN3649	27	6	3.666667	1.063218	1.16E-07	2.501636	6.82E-06	0.000515	4.41E-07	50	64	60	58	54	77	54	61.66667
SEN3650	73	41	23.33333	1.119658	7.69E-10	2.651126	2.52E-05	3.41E-06	3.86E-09	195	216	174	195	233	247	175	218.3333
SEN3651	2349	1946	-2085.33	-6.48772	1.17E-07	-2.76327	-0.00012	0.000518	4.43E-07	2585	2470	2341	2465.333	395	236	509	380
SEN3658	269	216	-237.667	-24.7667	1.58E-37	-10.4223	-1.7E-05	7.02E-34	4.56E-36	275	226	242	247.6667	10	6	14	10
SEN3673	25	7	-1	-1.03571	0.000289	2.258165	2.85E-06	1	0.000679	32	24	31	29	15	29	40	28
SEN3761	319	253	-273.667	-4.96618	1.2E-07	-2.10211	-1.4E-05	0.000533	4.54E-07	380	321	327	342.6667	68	61	78	69
SEN3797	63	19	16.33333	1.204167	3.94E-09	2.831451	1.14E-05	1.75E-05	1.82E-08	92	75	73	80	98	64	127	96.33333
SEN3819	174	15	28	1.099408	3.84E-09	2.565086	3.45E-05	1.7E-05	1.77E-08	265	308	272	281.6667	257	249	423	309.6667
SEN3820	965	280	558.6667	3.240642	5.83E-24	7.546447	0.000127	2.58E-20	9.09E-23	242	254	252	249.3333	685	532	1207	808
SEN3832	50	6	11.66667	1.090206	3.65E-11	2.558305	1.57E-05	1.62E-07	2.1E-10	130	138	120	129.3333	124	129	170	141
SEN3833	130	21	-37.6667	-1.0547	2.77E-08	2.238053	7E-05	0.000123	1.15E-07	769	694	716	726.3333	639	712	715	688.6667
SEN3835	3338	2902	-3017.67	-5.51521	1.07E-05	-2.34696	-0.00017	0.047609	3.14E-05	3649	3789	3620	3686	718	451	836	668.3333
SEN3842	38	13	22	2.466667	1.57E-13	5.784153	5.63E-06	6.95E-10	1.16E-12	25	15	5	15	40	28	43	37
SEN3843	20	10	3	1.051429	8.16E-09	2.466519	6.69E-06	3.62E-05	3.6E-08	52	62	61	58.33333	51	62	71	61.33333
SEN3844	22	1	8.333333	1.238095	4.11E-09	2.900508	5.21E-06	1.82E-05	1.89E-08	35	35	35	35	37	36	57	43.33333
SEN3855	26	12	-6	-1.07087	1.03E-07	2.200753	8.5E-06	0.000456	3.92E-07	96	94	82	90.66667	90	70	94	84.66667
SEN3858	955	788	-869	-5.23902	2.66E-08	-2.22764	-4.6E-05	0.000118	1.1E-07	1133	969	1120	1074	178	181	256	205
SEN3861	4342	2958	-3659.33	-17.9675	0	-7.67538	-0.00026	0	0	4469	3138	4018	3875	180	127	340	215.6667
SEN3870	8875	5113	-7324	-21.5538	0	-9.12236	-0.00053	0	0	9183	5427	8431	7680.333	314	308	447	356.3333
SEN3893	59	41	46.33333	3.206349	1.71E-28	7.519959	1.08E-05	7.59E-25	3.33E-27	20	20	23	21	61	79	62	67.33333
SEN3896	29	8	10	1.170455	1.28E-10	2.771477	8.09E-06	5.68E-07	7E-10	72	45	59	58.66667	74	65	67	68.66667
SEN3897	15	7	-4.33333	-1.07429	2.19E-07	2.194495	5.84E-06	0.000972	8.09E-07	63	55	70	62.66667	58	55	62	58.33333
SEN3975	456	398	-412.333	-8.49697	2.27E-15	-3.62393	-2.6E-05	1.01E-11	1.95E-14	447	497	458	467.3333	49	41	75	55
SEN3978	617	537	-570.667	-4.77093	1.99E-07	-2.02307	-2.8E-05	0.000881	7.38E-07	726	692	748	722	131	168	155	151.3333
SEN3982	299	241	-263.333	-10.4048	2.29E-19	-4.41203	-1.8E-05	1.02E-15	2.69E-18	270	324	280	291.3333	30	25	29	28
SEN3984	5	2	-1	-1.07692	0.003808	2.17275	1.3E-06	1	0.007301	12	14	16	14	11	12	16	13

Feature ID	Experiment - Range (original values)	Experiment - IQR (original values)	Experiment - Difference (original values)	Experiment - Fold Change (original values)	EDGE test: yccT H202 vs yccT NT , tagwise dispersions - P-value	EDGE test: yccT H202 vs yccT NT , tagwise dispersions - Fold change	EDGE test: yccT H202 vs yccT NT , tagwise dispersions - Weighted difference	EDGE test: yccT H202 vs yccT NT , tagwise dispersions - Bonferroni	EDGE test: yccT H202 vs yccT NT , tagwise dispersions - FDR p-value correction	yccT NT - yccT.1.S28 - Expression values	yccT NT - yccT.2.S29 - Expression values	yccT NT - yccT.3.S30 - Expression values	yccT NT - Means	yccT H202 - yccT.1.H2O2 - Expression values	yccT H202 - yccT.2.H2O2 - Expression values	yccT H202 - yccT.3.H2O2 - Expression values	yccT H202 - Means
SEN3985	25	4	-1.66667	-1.02994	1.59E-07	2.29247	5.78E-06	0.000706	5.95E-07	59	44	69	57.33333	54	58	55	55.66667
SEN4008	45	23	32.33333	1.76378	7.95E-18	4.147488	1.04E-05	3.52E-14	8.29E-17	36	41	50	42.33333	81	64	79	74.66667
SEN4022	57	15	10.33333	1.137168	6.31E-08	2.669982	9.84E-06	0.00028	2.49E-07	68	75	83	75.33333	58	115	84	85.66667
SEN4026	32	15	20.33333	2.297872	9.86E-16	5.380971	5.4E-06	4.37E-12	8.74E-15	18	17	12	15.66667	32	44	32	36
SEN4027	64	15	28.33333	1.876289	8.85E-12	4.434426	8.67E-06	3.92E-08	5.47E-11	44	33	20	32.33333	50	84	48	60.66667
SEN4028	46	38	41	2.242424	1.76E-25	5.273024	1.1E-05	7.78E-22	2.99E-24	36	33	30	33	71	76	75	74
SEN4029	30	4	12.66667	1.550725	1.27E-09	3.646701	4.77E-06	5.63E-06	6.25E-09	27	19	23	23	26	49	32	35.66667
SEN4032	52	11	31.66667	1.688406	1.09E-15	3.955533	1.06E-05	4.84E-12	9.63E-15	39	47	52	46	58	91	84	77.66667
SEN4037	37	23	27.33333	1.136667	2.51E-13	2.682392	2.62E-05	1.11E-09	1.82E-12	205	201	194	200	227	224	231	227.3333
SEN4040	116	59	-60.6667	-1.10551	5.82E-08	2.128965	5.6E-05	0.000258	2.31E-07	632	646	629	635.6667	530	570	625	575
SEN4052	38	16	8.333333	1.129534	2.57E-08	2.687291	8.44E-06	0.000114	1.07E-07	88	55	50	64.33333	85	71	62	72.66667
SEN4074	1003	729	-841.667	-7.99446	3.69E-07	-3.45041	-5.3E-05	0.001636	1.32E-06	1008	1056	822	962	93	53	215	120.3333
SEN4084	7	4	2	1.222222	0.000545	2.849394	1.32E-06	1	0.001229	11	7	9	9	12	7	14	11
SEN4087	21	7	0.666667	1.033333	0.00076	2.414811	2.24E-06	1	0.001669	12	33	15	20	22	23	17	20.66667
SEN4089	174	41	-32.6667	-1.08805	1.13E-06	2.175199	3.7E-05	0.00502	3.79E-06	375	382	454	403.6667	341	473	299	371
SEN4138	14	6	5	1.133929	1.52E-08	2.66265	4.86E-06	6.73E-05	6.47E-08	36	35	41	37.33333	35	43	49	42.33333
SEN4156	9	0	1.333333	1.059701	1.29E-05	2.491289	2.61E-06	0.057341	3.74E-05	22	23	22	22.33333	22	29	20	23.66667
SEN4182	454	381	-408	-8.41818	3.6E-18	-3.5658	-2.6E-05	1.59E-14	3.9E-17	499	440	450	463	45	61	59	55
SEN4190	86	72	-77	-5.2	7.48E-06	-2.19633	-4E-06	0.033143	2.22E-05	103	93	90	95.33333	20	17	18	18.33333
SEN4191	67	46	-55.6667	-9.35	1.46E-08	-3.92257	-3.6E-06	6.49E-05	6.25E-08	63	52	72	62.33333	9	5	6	6.666667
SEN4199	294	258	-272.333	-4.78241	5.43E-07	-2.02093	-1.4E-05	0.002405	1.89E-06	360	329	344	344.3333	71	79	66	72
SEN4200	483	397	-431.333	-5.95785	3.97E-10	-2.51405	-2.4E-05	1.76E-06	2.05E-09	513	480	562	518.3333	99	83	79	87
SEN4213	9991	7041	-8549.67	-9.69752	2.2E-07	-4.13438	-0.00056	0.000976	8.12E-07	10102	8110	10386	9532.667	1069	395	1485	983
SEN4216	69713	44544	-55241.7	-4.70244	0.008633	-2.00237	-0.00273	1	0.015337	73050	61370	76066	70162	16826	6353	21582	14920.33
SEN4217	51200	34317	-42481.3	-7.05435	3.98E-05	-2.99917	-0.00257	0.176178	0.000107	51848	42471	54175	49498	8154	2975	9921	7016.667
SEN4218	94674	75658	-85048.7	-15.3147	7.51E-10	-6.50739	-0.00599	3.33E-06	3.78E-09	93114	83144	96712	90990	7486	2038	8300	5941.333
SEN4231	239	192	-213	-4.94444	9.08E-07	-2.09103	-1.1E-05	0.004025	3.08E-06	288	272	241	267	49	64	49	54
SEN4241	69	13	-9.66667	-1.04149	2.16E-08	2.250109	2.37E-05	9.58E-05	9.09E-08	218	279	231	242.6667	218	210	271	233
SEN4243	275	60	-103.667	-1.11036	2.1E-06	2.120229	9.12E-05	0.009324	6.77E-06	1042	1109	978	1043	834	1022	962	939.3333
SEN4243A	43	5	15.33333	1.204444	5.61E-10	2.845944	1.08E-05	2.49E-06	2.85E-09	72	77	76	75	113	70	88	90.33333

Feature ID	Experiment - Range (original values)	Experiment - IQR (original values)	Experiment - Difference (original values)	Experiment - Fold Change (original values)	EDGE test: yccT H202 vs yccT NT , tagwise dispersions - P-value	EDGE test: yccT H202 vs yccT NT , tagwise dispersions - Fold change	EDGE test: yccT H202 vs yccT NT , tagwise dispersions - Weighted difference	EDGE test: yccT H202 vs yccT NT , tagwise dispersions - Bonferroni	EDGE test: yccT H202 vs yccT NT , tagwise dispersions - FDR p-value correction	yccT NT - yccT.1.S28 - Expression values	yccT NT - yccT.2.S29 - Expression values	yccT NT - yccT.3.S30 - Expression values	yccT NT - Means	yccT H202 - yccT.1.H2O2 - Expression values	yccT H202 - yccT.2.H2O2 - Expression values	yccT H202 - yccT.3.H2O2 - Expression values	yccT H202 - Means
SEN4244	89	27	31.33333	1.211236	6.83E-12	2.855855	2.14E-05	3.03E-08	4.27E-11	163	124	158	148.3333	136	213	190	179.6667
SEN4246	43	11	14.33333	1.263804	4.96E-10	2.980268	8.39E-06	2.2E-06	2.53E-09	62	45	56	54.33333	51	88	67	68.66667
SEN4247	14627	9302	12232.67	2.230197	1.11E-16	5.264561	0.003308	4.92E-13	1.08E-15	9533	9949	10349	9943.667	24160	19251	23118	22176.33
SEN4250	10	5	2	1.206897	0.000623	2.81579	1.4E-06	1	0.001383	6	13	10	9.666667	12	16	7	11.66667
SEN4251	42	15	26.66667	2.428571	2.79E-17	5.665184	6.85E-06	1.24E-13	2.85E-16	17	19	20	18.66667	43	34	59	45.33333
SEN4254	34	3	-8	-1.05229	2.9E-09	2.238789	1.56E-05	1.29E-05	1.37E-08	155	152	176	161	142	155	162	153
SEN4262	590	523	-538.667	-11.7733	1.75E-12	-5.04358	-3.7E-05	7.77E-09	1.19E-11	610	588	568	588.6667	45	20	85	50
SEN4264	26	5	12.33333	1.228395	3.05E-12	2.898251	7.99E-06	1.35E-08	1.99E-11	61	45	56	54	61	71	67	66.33333
SEN4265	1713	1037	1261.333	3.762044	3.96E-29	8.891902	0.000281	1.75E-25	7.9E-28	454	438	478	456.6667	1491	2151	1512	1718
SEN4266	1271	419	709.6667	2.117585	6.92E-16	5.008697	0.000198	3.07E-12	6.25E-15	642	642	621	635	1081	1892	1061	1344.667
SEN4269	8	3	1	1.333333	0.023226	3.025971	5.01E-07	1	0.037934	1	0	8	3	3	4	5	4
SEN4270	989	935	-942	-13.7873	1.73E-28	-5.87993	-6.6E-05	7.65E-25	3.34E-27	996	1002	1049	1015.667	60	61	100	73.66667
SEN4271	481	382	-432.667	-9.01235	6.84E-19	-3.82459	-2.8E-05	3.03E-15	7.77E-18	502	432	526	486.6667	45	50	67	54
SEN4279	54	21	-22	-1.15566	8.07E-07	2.037354	1.32E-05	0.003574	2.76E-06	156	162	172	163.3333	118	165	141	141.3333
SEN4280	80	56	-32.6667	-1.12129	2.69E-07	2.1077	2.61E-05	0.001192	9.83E-07	308	315	283	302	252	318	238	269.3333
SEN4291	354	315	-337	-7.39873	4.53E-15	-3.13315	-2.1E-05	2.01E-11	3.77E-14	400	398	371	389.6667	56	46	56	52.66667
SEN4292	1507	1402	1454.333	4.310319	1.81E-35	10.17104	0.000315	8.02E-32	4.77E-34	426	469	423	439.3333	1923	1930	1828	1893.667
SEN4293	144	19	-61.6667	-1.15302	3.77E-07	2.046804	3.8E-05	0.001671	1.35E-06	468	490	436	464.6667	441	346	422	403
SEN4294	116	31	-25.6667	-1.06649	3.93E-08	2.212161	3.89E-05	0.000174	1.6E-07	425	419	391	411.6667	422	310	426	386
SEN4299	7176	6586	-6724	-7.11458	3.3E-09	-3.02038	-0.00041	1.46E-05	1.54E-08	8118	7676	7677	7823.667	1090	942	1267	1099.667
SEN4303	7263	2908	-4699.33	-5.76767	0.021956	-2.50096	-0.00027	1	0.036019	5778	7408	3869	5685	961	145	1851	985.6667
SEN4304	10595	5288	-7407.33	-6.44657	0.021407	-2.80927	-0.00044	1	0.035183	9161	10728	6413	8767.333	1125	133	2822	1360
SEN4305	15415	10493	-12655.7	-11.635	1.87E-05	-5.06052	-0.00086	0.08283	5.29E-05	15677	14537	11323	13845.67	830	262	2478	1190
SEN4310	431	319	-377	-5.43529	9.81E-08	-2.3116	-2E-05	0.000435	3.76E-07	500	389	497	462	69	70	116	85
SEN4317	513	137	311.3333	4.288732	2.3E-24	10.00549	6.66E-05	1.02E-20	3.66E-23	80	84	120	94.66667	404	221	593	406
SEN4331A	25	11	1.666667	1.008897	1.02E-10	2.378601	2.01E-05	4.54E-07	5.61E-10	187	175	200	187.3333	178	189	200	189
SEN4351	36	9	-9	-1.17532	0.000434	2.005543	4.73E-06	1	0.000995	62	53	66	60.33333	61	30	63	51.33333
SEN4353	360	312	-319	-4.72374	4.24E-05	-2.02215	-1.6E-05	0.188031	0.000113	412	394	408	404.6667	52	82	123	85.66667
seqA	2468	2253	-2347.33	-11.7841	0	-5.02272	-0.00016	0	0	2654	2598	2443	2565	190	186	277	217.6667
serB	536	476	-496.333	-5.36657	1.66E-08	-2.28686	-2.7E-05	7.34E-05	7.03E-08	599	620	611	610	84	123	134	113.6667

Feature ID	Experiment - Range (original values)	Experiment - IQR (original values)	Experiment - Difference (original values)	Experiment - Fold Change (original values)	EDGE test: yccT H202 vs yccT NT , tagwise dispersions - P-value	EDGE test: yccT H202 vs yccT NT , tagwise dispersions - Fold change	EDGE test: yccT H202 vs yccT NT , tagwise dispersions - Weighted difference	EDGE test: yccT H202 vs yccT NT , tagwise dispersions - Bonferroni	EDGE test: yccT H202 vs yccT NT , tagwise dispersions - FDR p-value correction	yccT NT - yccT.1.S28 - Expression values	yccT NT - yccT.2.S29 - Expression values	yccT NT - yccT.3.S30 - Expression values	yccT NT - Means	yccT H202 - yccT.1.H2O2 - Expression values	yccT H202 - yccT.2.H2O2 - Expression values	yccT H202 - yccT.3.H2O2 - Expression values	yccT H202 - Means
serS	16464	14767	-15470.7	-6.06405	3.39E-07	-2.56739	-0.00088	0.001502	1.23E-06	19442	17786	18349	18525.67	2978	3019	3168	3055
sgbH	21	6	11	1.589286	5.38E-10	3.73754	4.01E-06	2.38E-06	2.74E-09	22	16	18	18.66667	28	37	24	29.66667
sgbU	34	6	17.66667	1.779412	5.38E-11	4.20034	5.69E-06	2.38E-07	3.05E-10	27	22	19	22.66667	40	53	28	40.33333
sicP	102	56	71.66667	6.243902	4.63E-38	14.49097	1.45E-05	2.05E-34	1.38E-36	13	11	17	13.66667	74	69	113	85.33333
sifA	125	65	83.66667	1.850847	2.93E-22	4.370027	2.59E-05	1.3E-18	4.08E-21	105	99	91	98.33333	164	216	166	182
sifB	36	13	25	2.923077	3.29E-18	6.80805	5.96E-06	1.46E-14	3.61E-17	16	14	9	13	27	45	42	38
sinH	44	11	19.66667	1.430657	2.82E-11	3.368188	8.43E-06	1.25E-07	1.65E-10	56	36	45	45.66667	45	80	71	65.33333
sinR	156	39	76.66667	1.694864	6.82E-15	3.997944	2.59E-05	3.02E-11	5.62E-14	99	112	120	110.3333	155	255	151	187
sipD	41	24	4	1.042553	6.08E-09	2.456868	1.06E-05	2.69E-05	2.72E-08	112	75	95	94	77	101	116	98
sitA	12896	8525	10489.33	63.18972	9.4E-99	149.0167	0.001946	4.15E-95	8.14E-97	172	156	178	168.6667	8697	13052	10225	10658
sitB	10117	5532	7205.667	147.0608	1.54E-91	346.3724	0.001332	6.82E-88	1.24E-89	54	54	40	49.33333	5586	10157	6022	7255
sitC	3613	1986	2537	53.13014	4.54E-78	125.5167	0.000473	2.01E-74	2.8E-76	52	44	50	48.66667	2064	3657	2036	2585.667
sitD	3637	1730	2381.667	24.04839	1.47E-54	56.69389	0.00045	6.5E-51	6.5E-53	98	111	101	103.3333	1831	3735	1889	2485
slrB	321	68	170.3333	2.67541	1.28E-16	6.36637	4.26E-05	5.69E-13	1.24E-15	100	109	96	101.6667	417	168	231	272
slsA	291	232	-257	-5.28333	1.4E-08	-2.24276	-1.4E-05	6.2E-05	5.99E-08	339	317	295	317	48	63	69	60
slyB	3300	1633	2324.667	1.829251	4.39E-14	4.3165	0.000725	1.94E-10	3.39E-13	2916	2872	2622	2803.333	4505	5922	4957	5128
smpA	1517	1043	1242	1.419878	6.07E-11	3.351102	0.000542	2.69E-07	3.41E-10	3131	2889	2854	2958	3932	4297	4371	4200
smtA	1178	987	-1086	-5.91403	8.45E-11	-2.51056	-6.1E-05	3.75E-07	4.67E-10	1363	1220	1338	1307	185	233	245	221
sodA	75471	34367	49805	100.8096	3.34E-78	238.9256	0.009257	1.48E-74	2.09E-76	514	511	472	499	40091	75943	34878	50304
sodB	26352	20073	-22187	-5.54217	0.000104	-2.34412	-0.00121	0.461956	0.000263	29338	26082	25795	27071.67	5946	2986	5722	4884.667
sodC	5340	1200	-739.667	-1.12542	0.001769	2.118151	0.000578	1	0.003608	7339	6557	6016	6637.333	5357	8838	3498	5897.667
sopB	221	184	-199	-6.37838	7.24E-09	-2.69924	-1.2E-05	3.21E-05	3.21E-08	237	227	244	236	45	23	43	37
sopD	28	8	4	1.045977	7.11E-09	2.47052	9.99E-06	3.15E-05	3.16E-08	80	79	102	87	87	107	79	91
sopE2	37	12	24	1.712871	6.83E-14	4.037098	8.02E-06	3.03E-10	5.22E-13	27	34	40	33.66667	64	63	46	57.66667
soxR	318	115	215	4.706897	5.15E-34	11.02575	4.54E-05	2.28E-30	1.25E-32	59	51	64	58	276	174	369	273
soxS	956	375	665	4.881323	4.51E-34	11.41531	0.00014	2E-30	1.11E-32	164	191	159	171.3333	855	539	1115	836.3333
spaO	14	5	9.333333	1.56	8.86E-10	3.651806	3.48E-06	3.93E-06	4.42E-09	18	15	17	16.66667	22	27	29	26
spaP	11	1	4.666667	1.333333	1.83E-06	3.121851	2.34E-06	0.008114	5.95E-06	16	11	15	14	15	22	19	18.66667
spaQ	5	1	1.666667	1.25	0.001279	2.902813	1.01E-06	1	0.002689	7	4	9	6.666667	8	9	8	8.333333
spaR	18	5	9.333333	1.965517	1.41E-08	4.574872	2.74E-06	6.24E-05	6.02E-08	9	8	12	9.666667	14	26	17	19

Feature ID	Experiment - Range (original values)	Experiment - IQR (original values)	Experiment - Difference (original values)	Experiment - Fold Change (original values)	EDGE test: yccT H202 vs yccT NT , tagwise dispersions - P-value	EDGE test: yccT H202 vs yccT NT , tagwise dispersions - Fold change	EDGE test: yccT H202 vs yccT NT , tagwise dispersions - Weighted difference	EDGE test: yccT H202 vs yccT NT , tagwise dispersions - Bonferroni	EDGE test: yccT H202 vs yccT NT , tagwise dispersions - FDR p-value correction	yccT NT - yccT.1.S28 - Expression values	yccT NT - yccT.2.S29 - Expression values	yccT NT - yccT.3.S30 - Expression values	yccT NT - Means	yccT H202 - yccT.1.H2O2 - Expression values	yccT H202 - yccT.2.H2O2 - Expression values	yccT H202 - yccT.3.H2O2 - Expression values	yccT H202 - Means
spaS	19	7	12.66667	2.809524	1.99E-11	6.508548	3.07E-06	8.84E-08	1.18E-10	9	6	6	7	25	13	21	19.66667
speC	671	585	-626.667	-5.15011	8.85E-09	-2.18696	-3.3E-05	3.92E-05	3.89E-08	798	743	792	777.6667	127	158	168	151
speF	15139	5879	10456.33	5.763705	4.65E-26	13.74331	0.002169	2.06E-22	8.27E-25	2502	1704	2379	2195	16843	8258	12853	12651.33
spr	4991	4801	-4887.67	-17.1132	0	-7.26311	-0.00035	0	0	5121	5215	5237	5191	320	246	344	303.3333
sptP	532	282	391.3333	4.025773	9.97E-39	9.536007	8.61E-05	4.42E-35	3.07E-37	129	124	135	129.3333	495	656	411	520.6667
spy	1207	35	304	1.398079	1.15E-06	3.307064	0.000137	0.005103	3.85E-06	792	732	767	763.6667	624	1831	748	1067.667
srlA	187	20	44.33333	1.305046	3.5E-07	3.101712	2.39E-05	0.001553	1.26E-06	138	158	140	145.3333	293	106	170	189.6667
srlD	2544	1143	1703	4.235592	1.91E-24	10.10453	0.000374	8.46E-21	3.04E-23	523	530	526	526.3333	3067	1952	1669	2229.333
srlE	530	52	265.6667	1.998747	2.27E-13	4.746327	7.79E-05	1E-09	1.65E-12	252	283	263	266	782	315	498	531.6667
srlR	1957	1804	-1855.33	-8.0545	1.63E-13	-3.41276	-0.00012	7.23E-10	1.2E-12	2153	2100	2102	2118.333	296	196	297	263
ssaB	22	7	12.66667	2.809524	8.05E-11	6.493544	3.08E-06	3.57E-07	4.46E-10	4	10	7	7	26	14	19	19.66667
ssaC	50	21	30.33333	2.568966	3.43E-17	6.064336	7.67E-06	1.52E-13	3.48E-16	24	14	20	19.33333	64	41	44	49.66667
ssaD	10	1	3.666667	1.123596	7.41E-08	2.644474	3.82E-06	0.000328	2.89E-07	32	26	31	29.66667	31	36	33	33.33333
ssaE	7	4	1.333333	1.114286	0.000472	2.606404	1.48E-06	1	0.001078	14	10	11	11.66667	14	9	16	13
ssaJ	16	4	-3	-1.15254	0.001756	2.0535	1.87E-06	1	0.003585	32	16	20	22.66667	24	19	16	19.66667
ssaK	5	0	-2	-1.16667	0.010536	2.013144	1.12E-06	1	0.018352	15	13	14	14	13	13	10	12
ssaM	35	26	27	4.115385	4.94E-23	9.56577	5.89E-06	2.19E-19	7.16E-22	13	6	7	8.666667	33	41	33	35.66667
ssaT	12	2	0.333333	1.037037	0.007563	2.406228	1E-06	1	0.013606	9	8	10	9	3	10	15	9.333333
ssaV	26	11	-0.33333	-1.00424	1.1E-08	2.356256	8.34E-06	4.88E-05	4.81E-08	89	63	85	79	76	86	74	78.66667
ssb	2154	927	1345	1.531481	2.34E-11	3.619483	0.000516	1.04E-07	1.38E-10	2667	2483	2442	2530.667	3410	4596	3621	3875.667
sscA	9	7	-1	-1.02778	3.52E-06	2.297243	3.75E-06	0.015614	1.1E-05	40	32	39	37	38	39	31	36
sseAb	30	8	13.66667	1.672131	1.37E-09	3.918095	4.67E-06	6.08E-06	6.73E-09	17	25	19	20.33333	28	47	27	34
sseBb	39	3	12.66667	1.304	2.83E-08	3.072411	6.77E-06	0.000125	1.17E-07	38	44	43	41.66667	45	77	41	54.33333
sseJ	38	13	21	2.086207	1.34E-09	4.854881	5.89E-06	5.95E-06	6.59E-09	16	29	13	19.33333	48	22	51	40.33333
sspA	6230	5484	-5759	-6.09796	4.49E-07	-2.57511	-0.00033	0.001988	1.58E-06	7255	6551	6860	6888.667	1067	1297	1025	1129.667
ssrA	142	98	115.3333	1.615658	6.75E-21	3.817761	4.12E-05	2.99E-17	8.67E-20	178	182	202	187.3333	308	320	280	302.6667
ssrB	262	19	120.3333	2.164516	4.88E-12	5.138715	3.34E-05	2.16E-08	3.11E-11	92	101	117	103.3333	197	354	120	223.6667
stbA	12	4	6	1.857143	1.25E-06	4.302885	1.84E-06	0.005536	4.15E-06	10	6	5	7	9	17	13	13
stbB	9	5	6.333333	2.727273	2.9E-07	6.194323	1.55E-06	0.001287	1.06E-06	4	2	5	3.666667	11	9	10	10
stbC	122	24	14.66667	1.049217	7.99E-10	2.48487	3.44E-05	3.54E-06	4E-09	306	249	339	298	285	371	282	312.6667

Feature ID	Experiment - Range (original values)	Experiment - IQR (original values)	Experiment - Difference (original values)	Experiment - Fold Change (original values)	EDGE test: yccT H2O2 vs yccT NT , tagwise dispersions - P-value	EDGE test: yccT H2O2 vs yccT NT , tagwise dispersions - Fold change	EDGE test: yccT H2O2 vs yccT NT , tagwise dispersions - Weighted difference	EDGE test: yccT H2O2 vs yccT NT , tagwise dispersions - Bonferroni	EDGE test: yccT H2O2 vs yccT NT , tagwise dispersions - FDR p-value correction	yccT NT - yccT.1.S28 - Expression values	yccT NT - yccT.2.S29 - Expression values	yccT NT - yccT.3.S30 - Expression values	yccT NT - Means	yccT H2O2 - yccT.1.H2O2 - Expression values	yccT H2O2 - yccT.2.H2O2 - Expression values	yccT H2O2 - yccT.3.H2O2 - Expression values	yccT H2O2 - Means
stdB	49	6	-4	-1.02941	1.12E-08	2.294796	1.41E-05	4.96E-05	4.88E-08	162	130	128	140	113	161	134	136
stdC	36	12	8.666667	1.346667	1.44E-05	3.138642	4.22E-06	0.063678	4.14E-05	16	29	30	25	18	52	31	33.66667
stfA	16	5	10.66667	1.842105	7.97E-10	4.312792	3.31E-06	3.53E-06	3.99E-09	13	12	13	12.66667	24	28	18	23.33333
stfC	27	12	-9.33333	-1.07	3.49E-09	2.201803	1.34E-05	1.55E-05	1.62E-08	148	139	141	142.6667	129	122	149	133.3333
sthA	26	14	4.666667	1.091503	6.83E-08	2.583973	6.29E-06	0.000303	2.68E-07	61	41	51	51	67	43	57	55.66667
stiH	101	34	-33.3333	-1.15723	2.45E-06	2.036084	1.98E-05	0.010868	7.82E-06	256	233	247	245.3333	161	262	213	212
sufA	6883	3571	5014.333	207.0685	4.94E-96	479.4175	0.000917	2.19E-92	4.21E-94	21	31	21	24.33333	4620	3592	6904	5038.667
sufB	26036	15321	20317	119.5817	3.4E-107	280.0163	0.003734	1.5E-103	3.2E-105	170	182	162	171.3333	19776	15491	26198	20488.33
sufC	8443	7224	7846.667	108.4886	7E-118	255.705	0.001448	3.1E-114	7E-116	80	62	77	73	7301	7953	8505	7919.667
sufD	15770	11274	12945.67	45.28392	3.97E-94	107.2291	0.002415	1.76E-90	3.32E-92	322	257	298	292.3333	11572	16027	12115	13238
sufS	21659	7602	12869	49.62343	6.27E-47	117.7615	0.002409	2.78E-43	2.48E-45	271	261	262	264.6667	9617	21920	7864	13133.67
sulA	22332	3750	12738.33	13.05901	1.04E-23	30.37823	0.002418	4.61E-20	1.57E-22	1117	1058	994	1056.333	13250	4808	23326	13794.67
sun	1228	1073	-1126	-5.39844	2.11E-08	-2.29482	-6.1E-05	9.33E-05	8.88E-08	1441	1314	1391	1382	241	213	314	256
surE	1019	801	-921	-4.90806	1.23E-07	-2.08171	-4.7E-05	0.000544	4.63E-07	1198	1052	1220	1156.667	201	251	255	235.6667
syd	647	552	-566.667	-4.81166	1.39E-06	-2.04938	-2.9E-05	0.006167	4.6E-06	764	698	684	715.3333	132	117	197	148.6667
tag	2265	2194	-2183	-6.57362	3.67E-10	-2.80135	-0.00013	1.63E-06	1.9E-09	2603	2541	2580	2574.667	347	338	490	391.6667
tatA	2833	2594	-2604.33	-5.78152	3.64E-05	-2.44474	-0.00015	0.161413	9.84E-05	3224	3218	3005	3149	391	832	411	544.6667
tatB	2767	2647	-2576	-5.31732	4.39E-05	-2.24833	-0.00014	0.194419	0.000117	3234	3132	3152	3172.667	467	838	485	596.6667
tatC	1758	1524	-1596.67	-5.20175	8.28E-07	-2.20522	-8.4E-05	0.003669	2.83E-06	2061	1898	1971	1976.667	303	463	374	380
tctD	140	26	-5.66667	-1.04899	0.000602	2.21251	1.15E-05	1	0.001342	115	123	126	121.3333	97	55	195	115.6667
tdcA	12614	11462	-11974.7	-145.273	4.79E-82	-61.6843	-0.00093	2.12E-78	3.26E-80	11551	12679	11943	12057.67	89	65	95	83
tdcC	79104	6396	43185	2.762581	1.18E-08	6.513028	0.010565	5.23E-05	5.11E-08	23003	28448	22052	24501	99545	20441	83072	67686
tdcD	29380	6160	11090.33	1.624632	2E-06	3.83362	0.003941	0.00886	6.46E-06	16071	21677	15517	17755	41931	12551	32054	28845.33
tdcE	153287	20285	103124	3.014206	6.63E-12	7.071398	0.024335	2.94E-08	4.16E-11	46924	61256	45415	51198.33	197056	67209	198702	154322.3
tdcG	92998	19431	61905.67	1.788052	2.57E-10	4.202548	0.019683	1.14E-06	1.35E-09	71871	90402	73393	78555.33	164869	92824	163690	140461
tdk	791	727	-754.333	-6.29977	7.58E-13	-2.66755	-4.4E-05	3.36E-09	5.3E-12	920	894	876	896.6667	149	129	149	142.3333
tgt	1712	1379	-1528.67	-6.40802	2.56E-10	-2.71234	-8.9E-05	1.14E-06	1.35E-09	1942	1655	1837	1811.333	230	342	276	282.6667
thiC	135	59	-25.6667	-1.08388	2.95E-07	2.175467	3.04E-05	0.001308	1.08E-06	320	339	336	331.6667	253	388	277	306
thiD	34	28	-11.3333	-1.09164	3.61E-08	2.156718	1.22E-05	0.00016	1.48E-07	142	122	141	135	113	112	146	123.6667
thiE	43	3	-1.33333	-1.03252	0.000838	2.293977	4.29E-06	1	0.001826	40	46	41	42.33333	38	64	21	41

Feature ID	Experiment - Range (original values)	Experiment - IQR (original values)	Experiment - Difference (original values)	Experiment - Fold Change (original values)	EDGE test: yccT H202 vs yccT NT , tagwise dispersions - P-value	EDGE test: yccT H202 vs yccT NT , tagwise dispersions - Fold change	EDGE test: yccT H202 vs yccT NT , tagwise dispersions - Weighted difference	EDGE test: yccT H202 vs yccT NT , tagwise dispersions - Bonferroni	EDGE test: yccT H202 vs yccT NT , tagwise dispersions - FDR p-value correction	yccT NT - yccT.1.S28 - Expression values	yccT NT - yccT.2.S29 - Expression values	yccT NT - yccT.3.S30 - Expression values	yccT NT - Means	yccT H202 - yccT.1.H2O2 - Expression values	yccT H202 - yccT.2.H2O2 - Expression values	yccT H202 - yccT.3.H2O2 - Expression values	yccT H202 - Means
thiG	32	13	3.666667	1.069182	1.01E-06	2.533766	6.35E-06	0.004462	3.39E-06	52	45	62	53	58	72	40	56.66667
thrA	23377	19179	-21248.3	-20.346	0	-8.61744	-0.00154	0	0	24411	20227	22402	22346.67	1034	1048	1213	1098.333
thrB	9566	7556	-8606.33	-8.17194	3.24E-11	-3.45495	-0.00054	1.44E-07	1.89E-10	10664	8764	9991	9806.333	1098	1294	1208	1200
thrC	14414	11004	-12800	-6.04865	6.52E-07	-2.55786	-0.00073	0.002888	2.25E-06	16624	13524	15858	15335.33	2210	2876	2520	2535.333
thrS	51384	40204	-44735.7	-7.30642	3.48E-09	-3.09012	-0.00272	1.54E-05	1.62E-08	57947	47242	50299	51829.33	7038	6563	7680	7093.667
tonB	339	25	166.6667	1.778816	2.3E-13	4.174636	5.29E-05	1.02E-09	1.68E-12	235	197	210	214	393	213	536	380.6667
torA	1269	1151	-1186.67	-5.59948	1.54E-09	-2.37595	-6.5E-05	6.83E-06	7.52E-09	1499	1402	1433	1444.667	251	230	293	258
torC	423	364	-387.667	-8.45513	1.36E-17	-3.58247	-2.5E-05	6.03E-14	1.4E-16	420	432	467	439.6667	56	44	56	52
tpx	3185	318	661.6667	1.28152	1.93E-05	3.041535	0.000373	0.085396	5.44E-05	2532	2214	2305	2350.333	1987	5117	1932	3012
treB	2097	139	417.6667	1.643885	0.000359	3.911808	0.000147	1	0.000831	630	641	675	648.6667	502	2397	300	1066.333
treC	2306	192	394.6667	1.551981	0.001338	3.710426	0.000151	1	0.002797	753	641	751	715	559	2538	232	1109.667
treR	84	47	-24	-1.08955	2.04E-08	2.156706	2.63E-05	9.04E-05	8.61E-08	303	287	286	292	240	240	324	268
trmU	1746	1367	-1566	-6.63986	5.61E-12	-2.80281	-9.2E-05	2.49E-08	3.55E-11	2019	1645	1867	1843.667	282	273	278	277.6667
trpC	643	577	-605.333	-4.81513	1.19E-07	-2.04325	-3E-05	0.000526	4.49E-07	776	729	787	764	152	144	180	158.6667
trxB	3826	2490	2966	2.275882	6.16E-18	5.387007	0.000794	2.73E-14	6.47E-17	2416	2213	2345	2324.667	4998	6039	4835	5290.667
trxC	14039	5198	9630	38.03846	7.18E-64	89.20218	0.001783	3.18E-60	3.46E-62	285	226	269	260	9938	5467	14265	9890
tsx	1790	299	556.6667	1.32681	4.06E-07	3.184014	0.000289	0.001799	1.44E-06	1884	1520	1706	1703.333	3310	1885	1585	2260
ttrB	229	196	-208.667	-18.3889	7.37E-30	-7.7546	-1.5E-05	3.27E-26	1.51E-28	238	207	217	220.6667	11	9	16	12
ttrC	161	119	-133	-7.23438	2.56E-10	-3.06676	-8.1E-06	1.14E-06	1.35E-09	141	146	176	154.3333	22	15	27	21.33333
ttrR	425	374	-394.333	-5.39777	2.17E-09	-2.28328	-2.1E-05	9.61E-06	1.04E-08	510	462	480	484	85	96	88	89.66667
ttrS	5169	4722	-4818	-10.1078	3.31E-13	-4.31209	-0.00032	1.46E-09	2.37E-12	5554	5211	5276	5347	489	385	713	529
tyrP	1385	1226	-1292.33	-5.21413	1.91E-07	-2.20127	-6.8E-05	0.000845	7.09E-07	1593	1540	1664	1599	327	314	279	306.6667
ubiA	897	370	568.3333	1.642669	4.31E-15	3.866939	0.000197	1.91E-11	3.6E-14	958	859	836	884.3333	1396	1229	1733	1452.667
ubiC	1215	400	726.3333	1.828517	1.02E-15	4.298937	0.000225	4.51E-12	9E-15	939	806	885	876.6667	1503	1285	2021	1603
ucpA	13440	10926	-11682	-7.31118	5.92E-06	-3.12241	-0.00072	0.026244	1.78E-05	14307	12853	13439	13533	1927	867	2759	1851
udhA	7704	399	3881	2.43052	2.73E-11	5.677152	0.000988	1.21E-07	1.61E-10	2846	2537	2756	2713	6386	3155	10241	6594
udk	856	774	-799.333	-10.3307	9.43E-20	-4.40681	-5.3E-05	4.18E-16	1.14E-18	921	844	890	885	70	65	122	85.66667
udp	8610	3817	6084.667	2.670847	4.8E-18	6.332024	0.001513	2.13E-14	5.08E-17	3803	3708	3414	3641.667	12024	7525	9630	9726.333
ugpQ	122	27	-24	-1.05577	5.41E-09	2.235209	4.37E-05	2.4E-05	2.44E-08	498	446	419	454.3333	376	475	440	430.3333
ugtL	32	16	21.33333	4.2	4.41E-17	9.710407	4.64E-06	1.95E-13	4.42E-16	5	5	10	6.666667	26	37	21	28

Feature ID	Experiment - Range (original values)	Experiment - IQR (original values)	Experiment - Difference (original values)	Experiment - Fold Change (original values)	EDGE test: yccT H202 vs yccT NT , tagwise dispersions - P-value	EDGE test: yccT H202 vs yccT NT , tagwise dispersions - Fold change	EDGE test: yccT H202 vs yccT NT , tagwise dispersions - Weighted difference	EDGE test: yccT H202 vs yccT NT , tagwise dispersions - Bonferroni	EDGE test: yccT H202 vs yccT NT , tagwise dispersions - FDR p-value correction	yccT NT - yccT.1.S28 - Expression values	yccT NT - yccT.2.S29 - Expression values	yccT NT - yccT.3.S30 - Expression values	yccT NT - Means	yccT H202 - yccT.1.H2O2 - Expression values	yccT H202 - yccT.2.H2O2 - Expression values	yccT H202 - yccT.3.H2O2 - Expression values	yccT H202 - Means
uppS	2926	2635	-2734	-17.503	1.82E-24	-7.47492	-0.0002	8.07E-21	2.91E-23	3038	2782	2879	2899.667	147	112	238	165.6667
uspA	15933	1311	5917.333	1.174888	3.53E-07	2.777821	0.004688	0.001562	1.27E-06	35001	34197	32307	33835	35508	48240	35509	39752.33
uspB	903	645	735	1.767758	1.07E-16	4.170149	0.000236	4.74E-13	1.04E-15	1007	944	921	957.3333	1664	1589	1824	1692.333
uvrA	337	105	101.6667	1.046522	3.95E-07	2.474289	0.000251	0.00175	1.4E-06	2323	2050	2183	2185.333	2186	2387	2288	2287
uvrB	679	282	390.3333	1.206344	4.07E-09	2.843696	0.000272	1.8E-05	1.87E-08	1965	1825	1885	1891.667	2167	2175	2504	2282
uvrC	5488	4739	-5181	-7.38054	2.9E-10	-3.12275	-0.00032	1.29E-06	1.52E-09	6270	5547	6162	5993	808	782	846	812
uvrD	629	212	395	1.150706	4.04E-08	2.714564	0.00035	0.000179	1.64E-07	2774	2562	2527	2621	2754	3138	3156	3016
uvrY	8166	7429	-7616	-7.63607	1.94E-09	-3.24509	-0.00047	8.57E-06	9.31E-09	9038	8646	8607	8763.667	1178	872	1393	1147.667
virK	532	418	-468.333	-7.65877	4.06E-16	-3.231	-2.9E-05	1.8E-12	3.77E-15	598	488	530	538.6667	70	75	66	70.33333
wcaD	11	4	0.333333	1.008929	1.17E-06	2.367285	4E-06	0.005199	3.92E-06	34	42	36	37.33333	32	38	43	37.66667
wcaE	40	20	30	2.836735	9.65E-21	6.646833	7.25E-06	4.28E-17	1.24E-19	20	16	13	16.33333	53	36	50	46.33333
wcaF	17	9	11.66667	3.058824	2.95E-11	7.046081	2.75E-06	1.31E-07	1.73E-10	5	5	7	5.666667	22	14	16	17.33333
wcaG	43	8	14.33333	1.43	8.14E-08	3.362104	6.16E-06	0.000361	3.15E-07	34	29	37	33.33333	29	72	42	47.66667
wcaH	18	3	2	1.125	0.000297	2.653286	2.07E-06	1	0.000696	22	10	16	16	13	28	13	18
wcaI	26	2	3	1.087379	7.5E-06	2.567804	4.2E-06	0.033249	2.23E-05	41	30	32	34.33333	28	54	30	37.33333
wcaJ	12	7	-0.66667	-1.01739	1.84E-06	2.312379	4E-06	0.008135	5.96E-06	42	41	34	39	32	44	39	38.33333
wza	13	10	1.666667	1.098039	0.000164	2.573265	2.1E-06	0.726757	0.000401	20	10	21	17	11	22	23	18.66667
wzb	5	2	1.333333	1.333333	0.01247	3.052001	6.67E-07	1	0.021477	3	3	6	4	5	3	8	5.333333
wzc	24	4	-3.66667	-1.07857	7.9E-06	2.18183	4.64E-06	0.035007	2.35E-05	56	46	49	50.33333	33	57	50	46.66667
wzxC	29	4	6.666667	1.16	3.1E-07	2.724019	5.64E-06	0.001373	1.13E-06	36	49	40	41.66667	38	65	42	48.33333
xapA	12	7	5.666667	1.548387	3.09E-06	3.609264	2.14E-06	0.013683	9.69E-06	8	15	8	10.33333	16	20	12	16
xapR	106	85	-89	-4.81429	6.79E-05	-2.04582	-4.5E-06	0.300922	0.000176	120	109	108	112.3333	14	23	33	23.33333
xseA	1421	1050	-1247.33	-5.28146	4.79E-08	-2.23852	-6.6E-05	0.000212	1.93E-07	1673	1347	1596	1538.667	252	297	325	291.3333
xthA	289	155	-91.3333	-1.07833	3.08E-06	2.191996	0.000117	0.013652	9.68E-06	1236	1258	1278	1257.333	1103	1342	1053	1166
yaaA	1400	279	523.3333	1.21122	6.42E-08	2.850189	0.000357	0.000284	2.53E-07	2586	2307	2540	2477.667	2993	2305	3705	3001
yacE	692	663	-666.333	-9.47034	1.83E-21	-4.03173	-4.4E-05	8.11E-18	2.43E-20	741	756	738	745	64	75	97	78.66667
yacF	962	933	-943	-7.21758	8.12E-16	-3.0653	-5.8E-05	3.6E-12	7.28E-15	1077	1102	1105	1094.667	143	144	168	151.6667
yacG	249	214	-230	-5.82517	4.74E-10	-2.4636	-1.3E-05	2.1E-06	2.43E-09	295	277	261	277.6667	47	50	46	47.66667
yadB	373	341	-352.333	-5.47881	2.26E-09	-2.3268	-1.9E-05	1E-05	1.07E-08	443	412	438	431	71	70	95	78.66667
yadE	1835	1629	-1734	-9.28344	0	-3.94119	-0.00011	0	0	2023	1829	1978	1943.333	188	200	240	209.3333

Feature ID	Experiment - Range (original values)	Experiment - IQR (original values)	Experiment - Difference (original values)	Experiment - Fold Change (original values)	EDGE test: yccT H202 vs yccT NT , tagwise dispersions - P-value	EDGE test: yccT H202 vs yccT NT , tagwise dispersions - Fold change	EDGE test: yccT H202 vs yccT NT , tagwise dispersions - Weighted difference	EDGE test: yccT H202 vs yccT NT , tagwise dispersions - Bonferroni	EDGE test: yccT H202 vs yccT NT , tagwise dispersions - FDR p-value correction	yccT NT - yccT.1.S28 - Expression values	yccT NT - yccT.2.S29 - Expression values	yccT NT - yccT.3.S30 - Expression values	yccT NT - Means	yccT H202 - yccT.1.H2O2 - Expression values	yccT H202 - yccT.2.H2O2 - Expression values	yccT H202 - yccT.3.H2O2 - Expression values	yccT H202 - Means
yadI	1287	1174	-1237.67	-24.3522	8.88E-76	-10.3005	-9.1E-05	3.93E-72	5.17E-74	1338	1228	1306	1290.667	51	54	54	53
yadQ	611	495	-528.333	-5.21543	8.27E-08	-2.21776	-2.8E-05	0.000367	3.2E-07	710	608	643	653.6667	113	99	164	125.3333
yaeB	530	410	-454	-7.21918	8.05E-14	-3.0658	-2.8E-05	3.57E-10	6.1E-13	511	479	591	527	69	61	89	73
yaeH	5824	4953	-5239.67	-9.41038	7.37E-11	-3.96904	-0.00034	3.27E-07	4.1E-10	6307	5804	5477	5862.667	524	862	483	623
yaeJ	525	440	-478.333	-4.72727	7.46E-07	-2.0092	-2.4E-05	0.003307	2.56E-06	637	553	630	606.6667	112	113	160	128.3333
yaeL	4071	3400	-3655.67	-5.07999	2.36E-05	-2.16227	-0.00019	0.10463	6.55E-05	4847	4188	4620	4551.667	776	788	1124	896
yafD	2434	1962	-2145.33	-4.80603	1.16E-05	-2.03987	-0.00011	0.051609	3.39E-05	2937	2498	2692	2709	503	536	652	563.6667
yafH	822	38	79	1.087422	2.22E-06	2.541302	0.000109	0.009853	7.13E-06	909	872	930	903.6667	910	608	1430	982.6667
yahN	1157	950	-1013.67	-6.18058	5.26E-11	-2.62381	-5.8E-05	2.33E-07	2.99E-10	1315	1139	1174	1209.333	189	158	240	195.6667
yaiC	737	222	-60.6667	-1.09579	0.001204	2.17431	6.34E-05	1	0.002539	745	632	705	694	483	1077	340	633.3333
yaiU	958	102	348	1.180155	5.38E-08	2.787729	0.000269	0.000239	2.15E-07	2008	1833	1954	1931.667	1906	2791	2142	2279.667
yaiY	458	101	7.333333	1.011765	1.72E-06	2.355168	6.6E-05	0.007605	5.6E-06	581	670	619	623.3333	518	458	916	630.6667
yaiZ	1143	1034	-1037.33	-7.90022	7.47E-08	-3.397	-6.5E-05	0.000331	2.91E-07	1213	1160	1190	1187.667	126	70	255	150.3333
yajB	120	44	76.66667	1.268065	3.34E-16	2.993206	4.44E-05	1.48E-12	3.12E-15	294	257	307	286	338	373	377	362.6667
yajD	904	790	-811.667	-7.5107	7.63E-12	-3.2127	-5E-05	3.38E-08	4.75E-11	995	926	888	936.3333	98	91	185	124.6667
yajG	609	552	-570.333	-6.09226	3.9E-11	-2.58993	-3.3E-05	1.73E-07	2.24E-10	685	700	662	682.3333	110	91	135	112
yaoF	430	339	-375.333	-5.36434	1.32E-08	-2.28631	-2E-05	5.85E-05	5.67E-08	421	505	458	461.3333	75	82	101	86
ybaD	616	102	-88.3333	-1.09539	2.67E-05	2.142639	9.03E-05	0.11851	7.37E-05	1028	948	1067	1014.333	926	618	1234	926
ybaE	784	733	-749.333	-5.23352	3.43E-09	-2.22081	-4E-05	1.52E-05	1.6E-08	920	953	906	926.3333	173	169	189	177
ybaJ	1060	312	-240.667	-1.10106	9.67E-05	2.124897	0.000231	0.428274	0.000245	2415	2907	2544	2622	2232	1926	2986	2381.333
ybaL	331	79	179	1.27454	9.33E-13	3.019572	0.000103	4.13E-09	6.45E-12	682	605	669	652	809	936	748	831
ybaM	31	8	4.333333	1.052	7.5E-10	2.471564	9.57E-06	3.32E-06	3.78E-09	89	85	76	83.33333	77	79	107	87.66667
ybaN	63	7	24	1.28125	8.58E-13	3.009382	1.34E-05	3.8E-09	5.97E-12	93	77	86	85.33333	95	93	140	109.3333
ybaO	635	431	509.6667	3.165722	1.18E-36	7.45909	0.000119	5.22E-33	3.3E-35	236	255	215	235.3333	667	850	718	745
ybaX	544	447	-493.333	-5.06593	6.35E-08	-2.13748	-2.5E-05	0.000282	2.51E-07	652	568	624	614.6667	121	135	108	121.3333
ybaY	1512	487	42.33333	1.028571	0.000222	2.439947	0.000166	0.984612	0.000532	1579	1341	1525	1481.667	1011	2523	1038	1524
ybbN	713	45	311.6667	1.286371	2.1E-09	3.053089	0.000174	9.3E-06	1E-08	1099	1022	1144	1088.333	1361	1735	1104	1400
ybbO	887	790	-817.667	-5.6109	1.82E-08	-2.393	-4.5E-05	8.08E-05	7.73E-08	1017	947	1021	995	157	134	241	177.3333
ybbY	23	8	1	1.016043	1.4E-08	2.402744	6.81E-06	6.21E-05	6E-08	75	52	60	62.33333	68	57	65	63.33333
ybdA	552	470	496.3333	8.482412	9.48E-90	20.09198	9.84E-05	4.2E-86	7.37E-88	82	47	70	66.33333	540	599	549	562.6667

Feature ID	Experiment - Range (original values)	Experiment - IQR (original values)	Experiment - Difference (original values)	Experiment - Fold Change (original values)	EDGE test: yccT H202 vs yccT NT , tagwise dispersions - P-value	EDGE test: yccT H202 vs yccT NT , tagwise dispersions - Fold change	EDGE test: yccT H202 vs yccT NT , tagwise dispersions - Weighted difference	EDGE test: yccT H202 vs yccT NT , tagwise dispersions - Bonferroni	EDGE test: yccT H202 vs yccT NT , tagwise dispersions - FDR p-value correction	yccT NT - yccT.1.S28 - Expression values	yccT NT - yccT.2.S29 - Expression values	yccT NT - yccT.3.S30 - Expression values	yccT NT - Means	yccT H202 - yccT.1.H2O2 - Expression values	yccT H202 - yccT.2.H2O2 - Expression values	yccT H202 - yccT.3.H2O2 - Expression values	yccT H202 - Means
ybdB	360	23	137.6667	5.916667	1.59E-09	13.966	2.85E-05	7.07E-06	7.74E-09	22	25	37	28	67	382	48	165.6667
ybdQ	3601	2914	-1723.33	-1.18036	0.0004	2.000223	0.00088	1	0.000924	11592	11937	10306	11278.33	8336	11651	8678	9555
ybdZ	101	67	85	52	1.12E-54	111.6102	1.59E-05	4.96E-51	5.01E-53	1	3	1	1.666667	90	102	68	86.66667
ybeD	3190	2243	2576.333	5.470214	1.25E-35	12.9411	0.000536	5.56E-32	3.37E-34	592	552	585	576.3333	2888	3742	2828	3152.667
ybeQ	1209	1018	-1052	-4.91078	3.85E-06	-2.09195	-5.4E-05	0.017075	1.19E-05	1407	1286	1270	1321	252	198	357	269
ybeR	8	3	2.666667	1.105263	1.26E-06	2.596122	3.17E-06	0.005593	4.18E-06	22	26	28	25.33333	30	25	29	28
ybfE	387	334	-356.667	-6.87912	1.04E-11	-2.92743	-2.1E-05	4.6E-08	6.38E-11	427	420	405	417.3333	40	71	71	60.66667
ybfM	2434	2079	-2038.33	-5.2377	0.001356	-2.26265	-0.00011	1	0.002833	2453	2457	2648	2519.333	374	214	855	481
ybgE	5224	3553	-4240.67	-4.89171	0.00072	-2.06944	-0.00021	1	0.001589	5875	4849	5267	5330.333	1296	651	1322	1089.667
ybgI	1083	871	-936.333	-5.05339	3.52E-07	-2.12564	-4.8E-05	0.001559	1.27E-06	1284	1097	1121	1167.333	266	226	201	231
ybgS	280	5	91.33333	1.481547	6.37E-09	3.505859	3.71E-05	2.82E-05	2.85E-08	201	192	176	189.6667	191	456	196	281
ybhF	1100	908	-972.667	-5.81518	4.1E-08	-2.44963	-5.4E-05	0.000182	1.66E-07	1257	1091	1176	1174.667	183	266	157	202
ybhK	1319	1264	-1284.67	-12.8951	5.73E-30	-5.4544	-8.9E-05	2.54E-26	1.18E-28	1420	1366	1392	1392.667	102	121	101	108
ybhQ	1467	1176	-1236	-6.60121	1.26E-05	-2.83622	-7.3E-05	0.055755	3.64E-05	1562	1366	1442	1456.667	190	95	377	220.6667
ybiB	934	346	-68	-1.06417	0.00022	2.228882	0.000108	0.974956	0.000528	1174	1074	1135	1127.667	789	1662	728	1059.667
ybiH	328	282	-291.333	-5.6738	6.63E-09	-2.41446	-1.6E-05	2.94E-05	2.95E-08	377	336	348	353.6667	49	54	84	62.33333
ybiJ	5061	2682	3659.667	18.85203	6.03E-70	44.11498	0.000687	2.67E-66	3.22E-68	226	182	207	205	2889	3462	5243	3864.667
ybiS	1023	896	-959.333	-6.64314	8.26E-14	-2.81536	-5.7E-05	3.66E-10	6.24E-13	1175	1074	1139	1129.333	152	180	178	170
ybiU	176	40	94.66667	1.726343	3.16E-17	4.070009	3.12E-05	1.4E-13	3.21E-16	140	133	118	130.3333	173	294	208	225
ybiV(1)	189	45	90.33333	1.239188	6.62E-15	2.918611	5.64E-05	2.93E-11	5.46E-14	389	346	398	377.6667	435	434	535	468
ybjE	577	531	-541	-5.48343	1.12E-09	-2.33395	-3E-05	4.96E-06	5.53E-09	646	682	657	661.6667	105	115	142	120.6667
ybjM	598	100	-0.33333	-1.0004	1.1E-05	2.351347	8.78E-05	0.048824	3.22E-05	883	837	783	834.3333	938	483	1081	834
ybjN	376	352	-364.667	-4.85211	1.47E-07	-2.05416	-1.8E-05	0.000651	5.51E-07	464	449	465	459.3333	98	89	97	94.66667
ybjP	498	318	419	1.503404	4.91E-15	3.55211	0.000165	2.18E-11	4.07E-14	863	807	827	832.3333	1305	1145	1304	1251.333
ybjX	3968	3510	-3742.67	-9.03722	1.71E-14	-3.84032	-0.00024	7.58E-11	1.37E-13	4382	3948	4295	4208.333	414	438	545	465.6667
ycaO	436	293	-372.667	-6.48039	6.72E-12	-2.74125	-2.2E-05	2.98E-08	4.21E-11	493	364	465	440.6667	57	71	76	68
ycbC	1104	994	-1044.33	-10.8833	1.26E-25	-4.60945	-7E-05	5.57E-22	2.16E-24	1150	1107	1193	1150	113	89	115	105.6667
ycbK	2485	2122	-2290.33	-6.51445	3.64E-10	-2.76818	-0.00013	1.61E-06	1.89E-09	2818	2576	2723	2705.667	333	454	459	415.3333
ycbL	1797	1451	-1625.67	-4.81911	4.29E-06	-2.03459	-8.1E-05	0.018996	1.32E-05	2202	1863	2089	2051.333	412	460	405	425.6667
ycbW	929	776	-847.667	-13.5271	5.5E-32	-5.72064	-5.9E-05	2.44E-28	1.24E-30	912	841	993	915.3333	64	74	65	67.66667

Feature ID	Experiment - Range (original values)	Experiment - IQR (original values)	Experiment - Difference (original values)	Experiment - Fold Change (original values)	EDGE test: yccT H202 vs yccT NT , tagwise dispersions - P-value	EDGE test: yccT H202 vs yccT NT , tagwise dispersions - Fold change	EDGE test: yccT H202 vs yccT NT , tagwise dispersions - Weighted difference	EDGE test: yccT H202 vs yccT NT , tagwise dispersions - Bonferroni	EDGE test: yccT H202 vs yccT NT , tagwise dispersions - FDR p-value correction	yccT NT - yccT.1.S28 - Expression values	yccT NT - yccT.2.S29 - Expression values	yccT NT - yccT.3.S30 - Expression values	yccT NT - Means	yccT H202 - yccT.1.H2O2 - Expression values	yccT H202 - yccT.2.H2O2 - Expression values	yccT H202 - yccT.3.H2O2 - Expression values	yccT H202 - Means
yccR	357	325	-338.667	-4.95331	6.03E-08	-2.10004	-1.7E-05	0.000267	2.39E-07	425	411	437	424.3333	80	86	91	85.66667
yccV	1113	566	813.6667	2.073439	2.47E-19	4.883923	0.000229	1.09E-15	2.9E-18	810	768	696	758	1572	1334	1809	1571.667
ycdZ	1029	41	182	1.298851	3.49E-05	3.024049	9.61E-05	0.15482	9.47E-05	626	616	585	609	788	278	1307	791
yceA	28	13	-8.33333	-1.11261	4.13E-07	2.120503	7.19E-06	0.001831	1.46E-06	88	67	92	82.33333	64	80	78	74
yceH	4256	3796	-3936	-9.71439	2.89E-15	-4.09952	-0.00026	1.28E-11	2.45E-14	4689	4231	4243	4387.667	487	433	435	451.6667
yceI	612	45	260	3.626263	1.57E-11	8.646218	5.89E-05	6.96E-08	9.48E-11	114	95	88	99	237	700	140	359
yceO	61	51	-54.6667	-5.82353	1.25E-05	-2.46206	-3.1E-06	0.055445	3.62E-05	69	61	68	66	8	10	16	11.33333
yceP	6738	810	2853.333	1.237917	5.13E-08	2.922749	0.001798	0.000228	2.06E-07	12486	12768	10725	11993	13296	17463	13780	14846.33
ycfF	801	683	-721	-4.93273	4.46E-08	-2.09288	-3.7E-05	0.000198	1.81E-07	959	875	879	904.3333	158	192	200	183.3333
ycfJ	332	78	169.3333	1.175172	4.24E-10	2.761546	0.000133	1.88E-06	2.18E-09	888	1045	967	966.6667	1009	1220	1179	1136
ycfQ	1343	70	-0.33333	-1.00022	0.000154	2.345065	0.00016	0.680896	0.000378	1565	1495	1522	1527.333	1662	788	2131	1527
ycfR	5672	3688	4599.333	271.549	2.4E-110	636.113	0.000847	1.1E-106	2.3E-108	20	12	19	17	3707	5684	4458	4616.333
ycfU	763	663	-698.667	-12.2086	6.98E-29	-5.17732	-4.8E-05	3.09E-25	1.38E-27	813	730	740	761	50	67	70	62.33333
ycfV	452	342	-377.667	-9.5188	1.54E-19	-4.02158	-2.5E-05	6.81E-16	1.83E-18	488	387	391	422	36	52	45	44.33333
ycfW	672	559	-609	-6.60429	1.02E-11	-2.78724	-3.6E-05	4.53E-08	6.3E-11	729	661	763	717.6667	102	133	91	108.6667
ycfX	1035	866	-937.333	-7.12636	1.3E-15	-3.01331	-5.7E-05	5.76E-12	1.14E-14	1182	1019	1070	1090.333	153	147	159	153
ychA	670	558	-602	-5.39416	3.01E-09	-2.28627	-3.2E-05	1.33E-05	1.41E-08	784	733	700	739	142	114	155	137
ychE	472	381	-399.667	-5.49064	2.27E-06	-2.34147	-2.2E-05	0.010064	7.27E-06	525	476	465	488.6667	84	53	130	89
ychH	7623	268	1334	1.379624	0.00164	3.20836	0.000605	1	0.003365	3596	3607	3339	3514	5235	843	8466	4848
ychJ	577	552	-553.667	-7.51373	1.72E-15	-3.19757	-3.4E-05	7.61E-12	1.49E-14	649	640	627	638.6667	75	72	108	85
ychK	2441	2292	-2322.67	-8.65714	6.66E-16	-3.67662	-0.00015	2.95E-12	6.04E-15	2711	2582	2585	2626	290	270	350	303.3333
ychM	5784	321	892.3333	1.303205	0.001969	3.040831	0.000467	1	0.003991	3117	2796	2916	2943	4288	717	6501	3835.333
ychN	506	407	-443.333	-4.86628	4.83E-06	-2.07309	-2.3E-05	0.021393	1.47E-05	568	585	521	558	114	79	151	114.6667
yciB	904	696	-760	-4.75	2.47E-06	-2.0161	-3.8E-05	0.010934	7.87E-06	1056	899	933	962.6667	203	152	253	202.6667
yciC	1261	976	-1095.67	-5.1555	3.82E-06	-2.19738	-5.8E-05	0.016934	1.18E-05	1442	1219	1417	1359.333	243	181	367	263.6667
yciG	14	4	7.666667	1.418182	5.2E-08	3.314152	3.34E-06	0.00023	2.08E-07	17	21	17	18.33333	18	31	29	26
yciI	1297	1167	-1222	-9.3508	4.35E-18	-3.96068	-8E-05	1.93E-14	4.65E-17	1404	1369	1332	1368.333	167	107	165	146.3333
yciK	1680	1552	-1594.33	-6.02416	6.14E-10	-2.55608	-9.1E-05	2.72E-06	3.11E-09	1893	1969	1873	1911.667	289	342	321	317.3333
yciO	489	48	-124	-1.08665	9.74E-06	2.170991	0.000142	0.043149	2.87E-05	1639	1489	1537	1555	1506	1150	1637	1431
ycjX	1244	968	-1075.33	-5.15187	3.88E-07	-2.17666	-5.6E-05	0.001718	1.38E-06	1450	1311	1242	1334.333	297	206	274	259

Feature ID	Experiment - Range (original values)	Experiment - IQR (original values)	Experiment - Difference (original values)	Experiment - Fold Change (original values)	EDGE test: yccT H202 vs yccT NT , tagwise dispersions - P-value	EDGE test: yccT H202 vs yccT NT , tagwise dispersions - Fold change	EDGE test: yccT H202 vs yccT NT , tagwise dispersions - Weighted difference	EDGE test: yccT H202 vs yccT NT , tagwise dispersions - Bonferroni	EDGE test: yccT H202 vs yccT NT , tagwise dispersions - FDR p-value correction	yccT NT - yccT.1.S28 - Expression values	yccT NT - yccT.2.S29 - Expression values	yccT NT - yccT.3.S30 - Expression values	yccT NT - Means	yccT H202 - yccT.1.H2O2 - Expression values	yccT H202 - yccT.2.H2O2 - Expression values	yccT H202 - yccT.3.H2O2 - Expression values	yccT H202 - Means
ydcI	915	103	380	1.444271	8.87E-11	3.393824	0.000159	3.93E-07	4.9E-10	937	754	875	855.3333	1203	834	1669	1235.333
yddG	166	156	-159	-13.5526	9.26E-22	-5.7286	-1.1E-05	4.11E-18	1.25E-20	176	172	167	171.6667	10	11	17	12.66667
ydeI	155	55	89.66667	2.280952	9.77E-21	5.389983	2.4E-05	4.33E-17	1.25E-19	81	77	52	70	140	207	132	159.6667
ydeJ	15	4	-1.66667	-1.0495	9.38E-06	2.241156	3.43E-06	0.041556	2.76E-05	42	30	34	35.33333	27	33	41	33.66667
ydeV	17466	11403	-14577.3	-33.1323	4.44E-16	-14.2383	-0.00108	1.97E-12	4.09E-15	17635	11766	15692	15031	363	169	829	453.6667
ydeW	9662	7208	-8499.67	-87.4373	3.13E-74	-36.9299	-0.00065	1.39E-70	1.76E-72	9748	7307	8739	8598	99	86	110	98.33333
ydeY	6411	4122	-5408.33	-61.316	4.6E-34	-26.1514	-0.00041	2.04E-30	1.13E-32	6460	4197	5837	5498	75	49	145	89.66667
ydeZ	8432	5450	-7115.33	-68.1258	1.33E-22	-29.1549	-0.00054	5.91E-19	1.88E-21	8464	5547	7653	7221.333	97	32	189	106
ydfH	1856	1556	-1630.67	-12.3241	8.14E-17	-5.28296	-0.00011	3.61E-13	8.01E-16	1953	1708	1663	1774.667	107	97	228	144
ydfZ	228	41	-18.6667	-1.04523	8.33E-07	2.266746	4.26E-05	0.003692	2.85E-06	407	433	454	431.3333	392	537	309	412.6667
ydgC	56	41	-42	-4.81818	0.002865	-2.04887	-2.1E-06	1	0.005606	49	48	62	53	7	6	20	11
ydgH	3887	3342	-3573	-6.9385	1.55E-08	-2.93787	-0.00021	6.87E-05	6.6E-08	4321	4026	4177	4174.667	687	434	684	601.6667
ydgT	281	244	-256.333	-10.859	7.31E-18	-4.61568	-1.7E-05	3.24E-14	7.64E-17	270	297	280	282.3333	26	16	36	26
ydhD	1741	259	705.6667	1.419457	1.01E-08	3.364115	0.00031	4.5E-05	4.44E-08	1814	1622	1611	1682.333	1931	3352	1881	2388
ydhF	1940	1616	-1739	-6.0848	1.62E-08	-2.57826	-9.9E-05	7.18E-05	6.89E-08	2188	2056	1999	2081	383	248	395	342
ydhH	1171	1062	-1108	-4.77727	8.63E-07	-2.02409	-5.5E-05	0.003824	2.95E-06	1440	1358	1406	1401.333	296	269	315	293.3333
ydhM	604	112	301.3333	1.770673	2.38E-16	4.154096	9.59E-05	1.05E-12	2.25E-15	438	352	383	391	626	495	956	692.3333
ydhO	482	412	-433.333	-6.01931	6.8E-11	-2.55737	-2.5E-05	3.01E-07	3.79E-10	510	493	556	519.6667	81	74	104	86.33333
ydiA	1609	1400	-1467.67	-9.84137	2.79E-20	-4.18512	-9.7E-05	1.24E-16	3.48E-19	1748	1549	1604	1633.667	149	139	210	166
ydiE	704	486	560.6667	20.11364	1.4E-110	47.00698	0.000106	6E-107	1.3E-108	32	26	30	29.33333	516	524	730	590
ydiJ	5516	4619	-5053.67	-5.55422	2.61E-06	-2.35824	-0.00028	0.011561	8.29E-06	6516	5666	6308	6163.333	1000	1047	1282	1109.667
ydiP	136	104	-113.667	-5.37179	1.79E-06	-2.27242	-6.1E-06	0.00791	5.82E-06	153	133	133	139.6667	29	17	32	26
ydiU	1529	1066	1289.667	4.341105	1.47E-37	10.23572	0.000278	6.53E-34	4.27E-36	403	360	395	386	1677	1461	1889	1675.667
ydiY	335	237	-279.667	-7.40458	1.31E-13	-3.13805	-1.7E-05	5.81E-10	9.77E-13	320	279	371	323.3333	42	36	53	43.66667
ydjA	167	77	-91.3333	-1.17519	3.8E-07	2.004074	4.79E-05	0.001682	1.36E-06	637	594	607	612.6667	470	517	577	521.3333
ydjM	281	100	176.3333	2.406915	4.37E-25	5.628199	4.53E-05	1.94E-21	7.28E-24	119	133	124	125.3333	281	224	400	301.6667
yeaA	791	59	-74.6667	-1.09643	0.000579	2.131113	7.48E-05	1	0.001297	835	801	911	849	776	378	1169	774.3333
yeaC	322	33	-15	-1.01972	8.72E-08	2.30578	7.89E-05	0.000387	3.36E-07	797	753	777	775.6667	744	608	930	760.6667
yeaJ	637	569	-590.333	-12	5.89E-24	-5.08446	-4E-05	2.61E-20	9.16E-23	628	676	628	644	63	39	59	53.66667
yeaK	359	269	-325.667	-4.80156	4.16E-07	-2.02851	-1.6E-05	0.001842	1.47E-06	435	355	444	411.3333	85	86	86	85.66667

Feature ID	Experiment - Range (original values)	Experiment - IQR (original values)	Experiment - Difference (original values)	Experiment - Fold Change (original values)	EDGE test: yccT H202 vs yccT NT , tagwise dispersions - P-value	EDGE test: yccT H202 vs yccT NT , tagwise dispersions - Fold change	EDGE test: yccT H202 vs yccT NT , tagwise dispersions - Weighted difference	EDGE test: yccT H202 vs yccT NT , tagwise dispersions - Bonferroni	EDGE test: yccT H202 vs yccT NT , tagwise dispersions - FDR p-value correction	yccT NT - yccT.1.S28 - Expression values	yccT NT - yccT.2.S29 - Expression values	yccT NT - yccT.3.S30 - Expression values	yccT NT - Means	yccT H202 - yccT.1.H2O2 - Expression values	yccT H202 - yccT.2.H2O2 - Expression values	yccT H202 - yccT.3.H2O2 - Expression values	yccT H202 - Means
yeaM	79	19	-22.6667	-1.12078	3.91E-07	2.101247	1.8E-05	0.001732	1.39E-06	220	200	211	210.3333	192	146	225	187.6667
yeaR	20	6	4.666667	1.147368	1.28E-07	2.700986	4.21E-06	0.000565	4.8E-07	33	25	37	31.66667	29	45	35	36.33333
yeaS	1352	1250	-1270	-6.66122	5.8E-13	-2.82786	-7.5E-05	2.57E-09	4.09E-12	1555	1465	1463	1494.333	213	203	257	224.3333
yeaZ	749	647	-675.333	-8.73282	1.6E-19	-3.71127	-4.3E-05	7.1E-16	1.9E-18	820	730	738	762.6667	71	83	108	87.33333
yebA	4196	3866	-4035.33	-17.5609	0	-7.45304	-0.00029	0	0	4420	4100	4317	4279	224	234	273	243.6667
yebB	50	28	-14	-1.15498	3.8E-05	2.04492	8.52E-06	0.168166	0.000102	105	111	97	104.3333	77	122	72	90.33333
yebE	4515	2328	3463.333	6.465544	1.9E-35	15.22322	0.000701	8.4E-32	4.97E-34	672	578	651	633.6667	4219	2979	5093	4097
yebF	7424	3982	5696	21.27046	3.1E-70	49.83391	0.001072	1.37E-66	1.69E-68	274	295	274	281	5977	4256	7698	5977
yebG	4971	1579	3282.333	22.54705	2.53E-41	52.82658	0.000614	1.12E-37	8.69E-40	176	137	144	152.3333	3473	1723	5108	3434.667
yebK	1496	1395	-1434.67	-7.74608	3.91E-14	-3.28418	-8.9E-05	1.73E-10	3.04E-13	1651	1663	1628	1647.333	233	167	238	212.6667
yebN	652	581	-613.333	-31.6667	9.57E-68	-13.3893	-4.6E-05	4.24E-64	4.82E-66	666	603	631	633.3333	14	24	22	20
yecA	8456	7758	-8120.33	-8.39332	1.24E-11	-3.56298	-0.00052	5.5E-08	7.58E-11	9360	9447	8849	9218.667	1091	991	1213	1098.333
yecF	548	464	-492	-4.88421	7.34E-08	-2.06999	-2.5E-05	0.000325	2.87E-07	663	605	588	618.6667	124	115	141	126.6667
yecH	512	409	-449.333	-12.2333	5.05E-24	-5.16626	-3.1E-05	2.24E-20	7.91E-23	543	446	479	489.3333	31	52	37	40
yedF	20	10	4.666667	1.09589	4.84E-08	2.568328	5.99E-06	0.000215	1.95E-07	42	61	43	48.66667	53	45	62	53.33333
yedI	502	398	-442	-5.44966	6.83E-08	-2.31326	-2.4E-05	0.000302	2.68E-07	572	548	504	541.3333	106	70	122	99.33333
yedJ	71	38	-28.3333	-1.1114	8.58E-08	2.126966	2.48E-05	0.00038	3.31E-07	278	268	302	282.6667	240	292	231	254.3333
yeeA	3052	120	1142.333	1.982793	1.25E-06	4.634964	0.000329	0.005553	4.16E-06	1222	1102	1163	1162.333	2572	645	3697	2304.667
yeeI	4471	3934	-4137	-17.7716	0	-7.55236	-0.0003	0	0	4624	4324	4203	4383.667	269	153	318	246.6667
yeeX	2334	289	946.3333	1.260315	2.84E-08	2.961922	0.000556	0.000126	1.17E-07	3877	3660	3369	3635.333	4454	3588	5703	4581.667
yegQ	480	371	-421.333	-5.73408	3.82E-10	-2.42969	-2.3E-05	1.69E-06	1.97E-09	513	459	559	510.3333	88	79	100	89
yehE	2446	849	1727.333	14.0529	1.85E-39	32.84884	0.00033	8.19E-36	5.89E-38	117	136	144	132.3333	2031	985	2563	1859.667
yehT	300	265	-278.667	-5.72316	4.22E-10	-2.42852	-1.5E-05	1.87E-06	2.18E-09	327	334	352	337.6667	52	62	63	59
yehW	884	607	750	4.975265	1.5E-53	11.67431	0.000157	6.63E-50	6.5E-52	176	186	204	188.6667	793	963	1060	938.6667
yehX	248	138	187	2.329384	2.59E-28	5.526965	4.96E-05	1.15E-24	4.99E-27	156	128	138	140.6667	331	376	276	327.6667
yeiB	1148	971	1034	3.042133	1.18E-30	7.19236	0.000244	5.23E-27	2.48E-29	553	454	512	506.3333	1483	1536	1602	1540.333
yejF	1925	1506	1661.333	2.847294	2.27E-23	6.765864	0.000402	1.01E-19	3.39E-22	1029	746	923	899.3333	2671	2582	2429	2560.667
yejG	3394	1750	2673	6.197019	5.84E-35	14.60471	0.000545	2.59E-31	1.51E-33	540	472	531	514.3333	3415	2281	3866	3187.333
yejK	1755	1535	-1646	-6.32686	6.13E-11	-2.68156	-9.5E-05	2.72E-07	3.44E-10	1986	1843	2036	1955	308	281	338	309
yfaW	477	131	114	1.092084	1.43E-07	2.588945	0.000153	0.000634	5.37E-07	1283	1284	1147	1238	1624	1152	1280	1352

Feature ID	Experiment - Range (original values)	Experiment - IQR (original values)	Experiment - Difference (original values)	Experiment - Fold Change (original values)	EDGE test: yccT H202 vs yccT NT , tagwise dispersions - P-value	EDGE test: yccT H202 vs yccT NT , tagwise dispersions - Fold change	EDGE test: yccT H202 vs yccT NT , tagwise dispersions - Weighted difference	EDGE test: yccT H202 vs yccT NT , tagwise dispersions - Bonferroni	EDGE test: yccT H202 vs yccT NT , tagwise dispersions - FDR p-value correction	yccT NT - yccT.1.S28 - Expression values	yccT NT - yccT.2.S29 - Expression values	yccT NT - yccT.3.S30 - Expression values	yccT NT - Means	yccT H202 - yccT.1.H2O2 - Expression values	yccT H202 - yccT.2.H2O2 - Expression values	yccT H202 - yccT.3.H2O2 - Expression values	yccT H202 - Means
yfaZ	61	9	20	1.080537	7.03E-12	2.542264	2.99E-05	3.12E-08	4.38E-11	246	245	254	248.3333	228	289	288	268.3333
yfbB	1007	882	-921.667	-7.46028	4.18E-15	-3.15695	-5.7E-05	1.85E-11	3.5E-14	1127	1017	1049	1064.333	120	173	135	142.6667
yfbG	3539	897	-300	-1.11379	0.008218	2.14247	0.000261	1	0.014677	3111	2779	2919	2936.333	2022	4713	1174	2636.333
yfbS	1751	1586	-1637.33	-7.37922	3.6E-14	-3.12488	-0.0001	1.59E-10	2.81E-13	1985	1855	1842	1894	234	280	256	256.6667
yfbU	2258	1956	-2056.33	-6.38777	2.12E-09	-2.71808	-0.00012	9.41E-06	1.02E-08	2542	2383	2389	2438	284	434	427	381.6667
yfcE	724	595	-647.667	-4.96531	2.4E-07	-2.10019	-3.3E-05	0.001065	8.82E-07	863	750	820	811	139	196	155	163.3333
yfcZ	3466	2968	-3136.67	-9.50045	2.64E-14	-4.04751	-0.00021	1.17E-10	2.09E-13	3754	3457	3306	3505.667	338	288	481	369
yfeC	1668	1585	-1615.67	-15.1725	1.12E-28	-6.46567	-0.00011	4.95E-25	2.19E-27	1753	1689	1747	1729.667	104	85	153	114
yfeD	1802	1706	-1731.67	-16.1458	9.68E-32	-6.86084	-0.00012	4.29E-28	2.13E-30	1891	1819	1828	1846	113	89	141	114.3333
yfeN	20	4	8.333333	1.287356	2.01E-09	3.029522	4.61E-06	8.91E-06	9.66E-09	35	31	21	29	35	41	36	37.33333
yffB	652	634	-636.333	-8.79184	9.87E-21	-3.73298	-4.1E-05	4.37E-17	1.25E-19	715	728	711	718	76	77	92	81.66667
yffH	340	291	-305.667	-7.36806	1.58E-10	-3.12999	-1.9E-05	7.01E-07	8.55E-10	350	344	367	353.6667	53	27	64	48
yfgB	1222	1110	-1161.33	-7.61101	2.75E-16	-3.23692	-7.2E-05	1.22E-12	2.59E-15	1368	1284	1359	1337	146	174	207	175.6667
yfgJ	108	46	69.33333	1.936937	1.47E-22	4.576244	2.06E-05	6.51E-19	2.07E-21	81	61	80	74	126	169	135	143.3333
yfhF	11992	2572	5935.667	4.737825	2.24E-15	11.25091	0.001268	9.94E-12	1.93E-14	1643	1559	1562	1588	4886	13551	4134	7523.667
yfhJ	302	130	-47	-1.16927	0.001571	2.035467	2.62E-05	1	0.003234	341	283	350	324.6667	211	462	160	277.6667
yfhL	365	313	-331.333	-6.68	2.33E-10	-2.834	-2E-05	1.03E-06	1.23E-09	394	371	404	389.6667	39	78	58	58.33333
yfhP	3266	743	1842	1.785055	2.28E-12	4.192503	0.000583	1.01E-08	1.51E-11	2549	2319	2171	2346.333	4066	3062	5437	4188.333
yfiC	616	500	-551.333	-4.73363	1.4E-06	-2.00642	-2.7E-05	0.006205	4.62E-06	732	649	716	699	116	178	149	147.6667
yfiE	207	79	-31	-1.09328	2.01E-06	2.137742	3.22E-05	0.008892	6.48E-06	355	348	387	363.3333	276	257	464	332.3333
yfiF	1052	814	-933	-4.87137	3.76E-07	-2.05289	-4.7E-05	0.001665	1.35E-06	1276	1056	1190	1174	257	242	224	241
yfiK	347	175	263.3333	4.779904	1.82E-44	11.22147	5.56E-05	8.08E-41	6.74E-43	74	71	64	69.66667	342	246	411	333
yfiN	818	757	-773.333	-5.38563	2.34E-09	-2.28993	-4.2E-05	1.04E-05	1.11E-08	971	919	959	949.6667	162	153	214	176.3333
yfiO	2805	2262	-2407.33	-5.99102	8.71E-07	-2.55061	-0.00014	0.003858	2.97E-06	3125	2740	2804	2889.667	478	320	649	482.3333
yfiP	2945	864	1942.667	5.421851	4.12E-25	12.71497	0.000401	1.82E-21	6.88E-24	463	411	444	439.3333	2482	1308	3356	2382
yfiQ	9543	7703	-8421	-7.44136	9.47E-07	-3.15854	-0.00052	0.004196	3.2E-06	10231	9244	9710	9728.333	1541	688	1693	1307.333
yfiR	988	863	-898.333	-5.95404	6.91E-08	-2.54566	-5.1E-05	0.000306	2.71E-07	1110	1018	1111	1079.667	155	123	266	181.3333
yfjB	1196	1043	-1074.33	-4.8369	5.56E-06	-2.06229	-5.4E-05	0.024629	1.68E-05	1404	1355	1304	1354.333	261	208	371	280
yfjD	888	660	-771.667	-5.67677	1.41E-09	-2.40265	-4.3E-05	6.27E-06	6.92E-09	1021	827	962	936.6667	133	195	167	165
yfjF	260	18	70	1.067939	5.32E-09	2.527948	0.000122	2.36E-05	2.41E-08	1108	935	1048	1030.333	1044	1195	1062	1100.333

Feature ID	Experiment - Range (original values)	Experiment - IQR (original values)	Experiment - Difference (original values)	Experiment - Fold Change (original values)	EDGE test: yccT H202 vs yccT NT , tagwise dispersions - P-value	EDGE test: yccT H202 vs yccT NT , tagwise dispersions - Fold change	EDGE test: yccT H202 vs yccT NT , tagwise dispersions - Weighted difference	EDGE test: yccT H202 vs yccT NT , tagwise dispersions - Bonferroni	EDGE test: yccT H202 vs yccT NT , tagwise dispersions - FDR p-value correction	yccT NT - yccT.1.S28 - Expression values	yccT NT - yccT.2.S29 - Expression values	yccT NT - yccT.3.S30 - Expression values	yccT NT - Means	yccT H202 - yccT.1.H2O2 - Expression values	yccT H202 - yccT.2.H2O2 - Expression values	yccT H202 - yccT.3.H2O2 - Expression values	yccT H202 - Means
ygaA	114	54	-26.6667	-1.06809	7.7E-09	2.208171	3.93E-05	3.41E-05	3.41E-08	444	377	434	418.3333	330	431	414	391.6667
ygaC	432	105	-17	-1.01297	9.15E-07	2.326287	0.000137	0.004055	3.1E-06	1329	1232	1421	1327.333	1337	1081	1513	1310.333
ygaM	205	132	154.6667	1.419151	3.79E-18	3.353615	6.76E-05	1.68E-14	4.07E-17	405	359	343	369	532	491	548	523.6667
ygaP	698	149	208.6667	1.230062	3.41E-09	2.887049	0.000133	1.51E-05	1.59E-08	987	838	896	907	1039	805	1503	1115.667
ygaU	42	13	-12	-1.11111	4.07E-07	2.12301	1.05E-05	0.001804	1.44E-06	133	120	107	120	91	126	107	108
ygbA	559	185	380.3333	3.524336	3.98E-30	8.267304	8.54E-05	1.76E-26	8.29E-29	157	153	142	150.6667	554	338	701	531
ygbJ	2390	430	816.6667	1.961161	2.27E-05	4.559992	0.000237	0.100579	6.32E-05	855	1062	632	849.6667	1921	344	2734	1666.333
ygbK	3828	418	1571.667	2.17934	7.02E-06	5.098687	0.000427	0.031084	2.1E-05	1352	1532	1114	1332.667	3729	578	4406	2904.333
ygbL	1876	122	336.3333	1.36426	0.001154	3.180872	0.000157	1	0.002442	952	970	848	923.3333	1457	223	2099	1259.667
ygbQ	668	598	-618.333	-6.92652	3.37E-12	-2.94183	-3.7E-05	1.49E-08	2.18E-11	717	709	742	722.6667	111	74	128	104.3333
ygcB	1405	1115	-1236.33	-8.44779	2.56E-09	-3.59471	-7.9E-05	1.13E-05	1.21E-08	1481	1301	1425	1402.333	186	76	236	166
ygcY	19646	15047	-17528	-27.8423	0	-11.8186	-0.00129	0	0	19988	15779	18776	18181	732	342	885	653
ygfE1031:	4869	4112	-4292	-4.67781	0.000496	-2.00116	-0.00021	1	0.001129	5792	5518	5067	5459	955	923	1623	1167
yggA	1030	778	-868.333	-4.83652	0.000112	-2.06739	-4.4E-05	0.498013	0.000282	1122	1160	1002	1094.667	224	130	325	226.3333
yggH	767	693	-693.333	-5.45396	9.68E-08	-2.30492	-3.7E-05	0.000429	3.71E-07	896	826	825	849	129	206	132	155.6667
yggM	1598	1517	-1548	-20.9313	1.41E-41	-8.88506	-0.00011	6.23E-38	4.86E-40	1622	1596	1659	1625.667	79	61	93	77.66667
yggR	86	4	26.33333	1.189904	1.79E-10	2.807392	1.96E-05	7.94E-07	9.62E-10	126	139	151	138.6667	143	212	140	165
yggS	653	571	-603.333	-6.04178	1.68E-11	-2.55468	-3.4E-05	7.44E-08	1.01E-10	758	695	716	723	130	105	124	119.6667
yghA	765	600	-656.667	-5.02041	1.34E-07	-2.13544	-3.4E-05	0.000592	5.02E-07	899	747	814	820	134	147	209	163.3333
yghB	1379	185	-113.333	-1.04827	4.08E-05	2.241212	0.000238	0.180893	0.000109	2635	2291	2458	2461.333	2273	1696	3075	2348
ygiB	5007	4360	-4407.67	-5.82239	3.5E-05	-2.49262	-0.00025	0.155289	9.5E-05	5558	5187	5220	5321.667	827	551	1364	914
ygiC	5970	5436	-5503	-5.95171	1.7E-06	-2.54141	-0.00031	0.007535	5.55E-06	6891	6365	6587	6614.333	929	921	1484	1111.333
ygiD	364	47	36.66667	1.115911	2.63E-06	2.597792	3.93E-05	0.011634	8.33E-06	338	289	322	316.3333	275	210	574	353
ygiH	1080	952	-984.333	-5.36834	1.17E-08	-2.28312	-5.3E-05	5.16E-05	5.06E-08	1268	1198	1163	1209.667	211	188	277	225.3333
ygiM	1726	1522	-1607	-10.642	4.88E-15	-4.55529	-0.00011	2.16E-11	4.05E-14	1679	1827	1815	1773.667	157	101	242	166.6667
ygiN	1170	233	-31.6667	-1.02959	0.000513	2.313505	0.000112	1	0.001164	1265	1026	1015	1102	793	1794	624	1070.333
ygiW	1479	197	96.66667	1.077046	0.000143	2.561188	0.000153	0.635458	0.000354	1321	1222	1221	1254.667	1025	2254	775	1351.333
ygjR	4517	3957	-4123.33	-9.25217	1.47E-08	-3.9375	-0.00027	6.5E-05	6.26E-08	4752	4579	4538	4623	581	235	683	499.6667
yhaK	1760	448	1011.333	7.414376	6.8E-29	17.20772	0.000199	3.01E-25	1.35E-27	158	162	153	157.6667	988	606	1913	1169
yhbC	2110	1844	-1966.33	-10.2752	2.22E-16	-4.38554	-0.00013	9.84E-13	2.11E-15	2260	2032	2243	2178.333	188	150	298	212

Feature ID	Experiment - Range (original values)	Experiment - IQR (original values)	Experiment - Difference (original values)	Experiment - Fold Change (original values)	EDGE test: yccT H202 vs yccT NT , tagwise dispersions - P-value	EDGE test: yccT H202 vs yccT NT , tagwise dispersions - Fold change	EDGE test: yccT H202 vs yccT NT , tagwise dispersions - Weighted difference	EDGE test: yccT H202 vs yccT NT , tagwise dispersions - Bonferroni	EDGE test: yccT H202 vs yccT NT , tagwise dispersions - FDR p-value correction	yccT NT - yccT.1.S28 - Expression values	yccT NT - yccT.2.S29 - Expression values	yccT NT - yccT.3.S30 - Expression values	yccT NT - Means	yccT H202 - yccT.1.H2O2 - Expression values	yccT H202 - yccT.2.H2O2 - Expression values	yccT H202 - yccT.3.H2O2 - Expression values	yccT H202 - Means
yhbL	1622	178	131.6667	1.045771	1.27E-05	2.488376	0.000334	0.056166	3.67E-05	2973	2862	2795	2876.667	3153	3747	2125	3008.333
yhbP	685	614	-636.667	-6.21858	5E-10	-2.61964	-3.7E-05	2.22E-06	2.55E-09	778	755	743	758.6667	129	144	93	122
yhbQ	1045	881	-948	-4.97762	4.9E-08	-2.10491	-4.8E-05	0.000217	1.96E-07	1278	1122	1159	1186.333	233	241	241	238.3333
yhbS	5402	4880	-5079.67	-7.18968	9.14E-10	-3.04434	-0.00031	4.05E-06	4.55E-09	6155	5717	5829	5900.333	837	753	872	820.6667
yhbT	5964	5471	-5757	-12.7251	0	-5.41109	-0.0004	0	0	6400	6416	5928	6248	452	457	564	491
yhbU	781	571	-651	-10.3894	1.66E-15	-4.36669	-4.3E-05	7.34E-12	1.44E-14	694	642	825	720.3333	71	93	44	69.33333
yhcH	1641	1194	-1399	-5.69463	1.98E-06	-2.41224	-7.7E-05	0.008764	6.39E-06	1824	1718	1549	1697	356	183	355	298
yhcM	1264	1107	-1152.33	-10.4712	8.01E-23	-4.45176	-7.7E-05	3.55E-19	1.15E-21	1364	1217	1241	1274	110	100	155	121.6667
yhcN	1779	425	898.3333	9.555556	4.58E-23	22.59556	0.000177	2.03E-19	6.65E-22	97	109	109	105	600	1876	534	1003.333
yhcO	31	8	0.333333	1.008475	3.52E-05	2.368922	4.21E-06	0.155985	9.53E-05	42	34	42	39.33333	23	54	42	39.66667
yhcQ	186	66	120.3333	1.9025	7.33E-20	4.500993	3.65E-05	3.25E-16	8.89E-19	128	137	135	133.3333	246	314	201	253.6667
yhcR	176	79	112.6667	2.942529	4.8E-26	6.965325	2.7E-05	2.13E-22	8.47E-25	67	55	52	58	150	228	134	170.6667
yhdG	1304	1100	-1202.67	-14.9845	1.31E-35	-6.34861	-8.4E-05	5.79E-32	3.49E-34	1383	1180	1303	1288.667	80	79	99	86
yheN	306	241	-276	-4.98077	2.35E-07	-2.10197	-1.4E-05	0.001042	8.64E-07	357	308	371	345.3333	76	67	65	69.33333
yheO	1683	1563	-1552.33	-7.39698	8.13E-09	-3.16942	-9.6E-05	3.6E-05	3.59E-08	1825	1781	1779	1795	216	142	370	242.6667
yheR	698	500	621	12.79114	8.7E-100	30.07397	0.000119	3.84E-96	7.68E-98	63	49	46	52.66667	549	744	728	673.6667
yheS	1401	1340	-1355	-4.92375	9.1E-07	-2.08648	-6.9E-05	0.004032	3.09E-06	1710	1679	1712	1700.333	311	386	339	345.3333
yhfA	2534	2294	-2302.67	-6.34261	3.92E-08	-2.71001	-0.00013	0.000174	1.6E-07	2853	2668	2680	2733.667	374	319	600	431
yhfC	85	9	25	1.220588	3.61E-11	2.860656	1.64E-05	1.6E-07	2.08E-10	124	117	99	113.3333	120	111	184	138.3333
yhfG	97	87	-88	-5.71429	7.15E-07	-2.42287	-4.9E-06	0.003167	2.46E-06	106	103	111	106.6667	16	14	26	18.66667
yhfK	2743	2414	-2517.67	-7.25766	2.71E-11	-3.07925	-0.00015	1.2E-07	1.59E-10	3073	2822	2865	2920	408	330	469	402.3333
yhgG	592	99	370.3333	2.222222	1.17E-17	5.212573	9.98E-05	5.18E-14	1.21E-16	269	308	332	303	752	407	861	673.3333
yhgH	83	34	57.33333	1.374728	1.18E-16	3.246028	2.68E-05	5.21E-13	1.14E-15	167	148	144	153	222	182	227	210.3333
yhgI	2509	767	1590.667	2.887658	3.65E-19	6.765048	0.000379	1.62E-15	4.21E-18	881	882	765	842.6667	2378	1648	3274	2433.333
yhhA	852	469	651	2.044385	1.81E-21	4.814631	0.000185	8.02E-18	2.42E-20	691	628	551	623.3333	1097	1323	1403	1274.333
yhhV	279	115	191	5.374046	7.79E-33	12.7337	4.01E-05	3.45E-29	1.8E-31	41	39	51	43.66667	230	318	156	234.6667
yhhW	832	27	281.3333	1.865641	1.25E-08	4.317185	8.4E-05	5.55E-05	5.41E-08	337	310	328	325	455	266	1098	606.3333
yhiP	28880	23539	-25373	-8.37516	5.48E-07	-3.55525	-0.00161	0.002428	1.91E-06	30486	28212	27742	28813.33	4203	1606	4512	3440.333
yhjC	56	15	18	1.124138	4.26E-12	2.65398	1.87E-05	1.89E-08	2.73E-11	154	125	156	145	167	141	181	163
yhjE	39	9	-13	-1.07943	5.33E-09	2.186369	1.63E-05	2.36E-05	2.41E-08	194	174	162	176.6667	155	165	171	163.6667

Feature ID	Experiment - Range (original values)	Experiment - IQR (original values)	Experiment - Difference (original values)	Experiment - Fold Change (original values)	EDGE test: yccT H202 vs yccT NT , tagwise dispersions - P-value	EDGE test: yccT H202 vs yccT NT , tagwise dispersions - Fold change	EDGE test: yccT H202 vs yccT NT , tagwise dispersions - Weighted difference	EDGE test: yccT H202 vs yccT NT , tagwise dispersions - Bonferroni	EDGE test: yccT H202 vs yccT NT , tagwise dispersions - FDR p-value correction	yccT NT - yccT.1.S28 - Expression values	yccT NT - yccT.2.S29 - Expression values	yccT NT - yccT.3.S30 - Expression values	yccT NT - Means	yccT H202 - yccT.1.H2O2 - Expression values	yccT H202 - yccT.2.H2O2 - Expression values	yccT H202 - yccT.3.H2O2 - Expression values	yccT H202 - Means
yhjJ	2257	1965	-2110.67	-7.07678	1.82E-11	-2.99924	-0.00013	8.08E-08	1.09E-10	2541	2329	2504	2458	364	284	394	347.3333
yhjW	1893	1707	-1692.67	-4.73657	0.000119	-2.02589	-8.5E-05	0.526045	0.000297	2210	2102	2125	2145.667	395	317	647	453
yiaC	1559	1403	-1463.67	-5.75731	2.1E-09	-2.43314	-8.1E-05	9.29E-06	1E-08	1856	1714	1744	1771.333	311	315	297	307.6667
yiaL	18	4	2	1.103448	0.000167	2.583247	2.41E-06	0.741217	0.000409	12	20	26	19.33333	16	30	18	21.33333
yiaM	21	3	5	1.555556	0.000127	3.643832	1.88E-06	0.563565	0.000317	12	4	11	9	8	25	9	14
yiaN	35	1	16	1.64	4.28E-09	3.867897	5.63E-06	1.9E-05	1.96E-08	24	26	25	25	38	59	26	41
yiaO	61	9	29	2.851064	8.65E-12	6.716496	7.04E-06	3.83E-08	5.36E-11	21	18	8	15.66667	38	69	27	44.66667
yicH	2090	1840	-1924.33	-4.99792	2.39E-06	-2.12698	-9.9E-05	0.010599	7.65E-06	2521	2422	2274	2405.667	431	434	579	481.3333
yidE	3196	2866	-2967.33	-9.09273	3.55E-11	-3.88353	-0.00019	1.57E-07	2.05E-10	3422	3217	3363	3334	351	226	523	366.6667
yidF	1651	1386	-1519.67	-13.4904	3.01E-20	-5.79619	-0.00011	1.33E-16	3.73E-19	1508	1722	1694	1641.333	71	122	172	121.6667
yidQ	732	658	-676	-5.06413	8.94E-08	-2.15963	-3.5E-05	0.000396	3.44E-07	850	875	802	842.3333	143	144	212	166.3333
yidY	93	32	-27	-1.12366	6.17E-07	2.083307	2.08E-05	0.002732	2.14E-06	221	250	265	245.3333	172	218	265	218.3333
yidZ	220	80	136	1.283333	3.28E-15	3.019246	7.56E-05	1.45E-11	2.77E-14	487	484	469	480	564	595	689	616
yieE	554	487	-485.333	-5.13636	1.02E-06	-2.19356	-2.6E-05	0.004535	3.44E-06	646	583	579	602.6667	92	92	168	117.3333
yifZ	141	68	101	1.751861	4.46E-21	4.125773	3.28E-05	1.97E-17	5.79E-20	126	124	153	134.3333	247	194	265	235.3333
yigI	34	15	23	2.642857	3.61E-18	6.15097	5.69E-06	1.6E-14	3.9E-17	11	15	16	14	30	36	45	37
yigW	491	362	-418.333	-4.78012	3.59E-07	-2.02206	-2.1E-05	0.00159	1.29E-06	588	473	526	529	111	97	124	110.6667
yihI	597	513	-514	-4.83582	8.79E-05	-2.07498	-2.6E-05	0.389571	0.000225	690	608	646	648	93	95	214	134
yihX	324	100	-53.6667	-1.05162	8.72E-07	2.253391	0.000107	0.003864	2.97E-06	1173	1036	1071	1093.333	971	1236	912	1039.667
yiiG	17	4	6.333333	1.452381	7.35E-07	3.399971	2.65E-06	0.003258	2.53E-06	15	18	9	14	26	14	21	20.33333
yiiQ	259	211	-229.333	-5.74483	1.82E-09	-2.43052	-1.3E-05	8.06E-06	8.76E-09	299	271	263	277.6667	52	40	53	48.33333
yiiR	105	15	21.33333	1.061185	1.78E-11	2.497896	4.07E-05	7.89E-08	1.07E-10	368	329	349	348.6667	342	334	434	370
yiiU	1124	666	-189.333	-1.06141	2.52E-05	2.209249	0.000309	0.111653	6.96E-05	3381	3391	3045	3272.333	2705	2715	3829	3083
yijF	3444	2366	2846.667	4.398329	7.34E-32	10.35291	0.00061	3.25E-28	1.63E-30	865	825	823	837.6667	3595	3191	4267	3684.333
yjaB	1247	1022	1095.333	3.215779	3.2E-31	7.576984	0.000254	1.42E-27	6.98E-30	515	521	447	494.3333	1538	1537	1694	1589.667
yjbE	141	111	118.6667	4.178571	3.47E-51	9.817423	2.57E-05	1.54E-47	1.44E-49	43	35	34	37.33333	147	146	175	156
yjbG	8	3	1.333333	1.072727	3.61E-05	2.514975	2.18E-06	0.159947	9.76E-05	20	20	15	18.33333	19	17	23	19.66667
yjbH	118	25	49	1.157388	1.14E-12	2.724453	4.19E-05	5.04E-09	7.81E-12	324	321	289	311.3333	299	407	375	360.3333
yjbJ	534	201	374	1.505861	2.73E-15	3.541892	0.000147	1.21E-11	2.34E-14	766	765	687	739.3333	966	1153	1221	1113.333
yjcB	1689	257	803	17.84615	3.97E-22	42.48645	0.000154	1.76E-18	5.46E-21	51	39	53	47.66667	516	1728	308	850.6667

Feature ID	Experiment - Range (original values)	Experiment - IQR (original values)	Experiment - Difference (original values)	Experiment - Fold Change (original values)	EDGE test: yccT H202 vs yccT NT , tagwise dispersions - P-value	EDGE test: yccT H202 vs yccT NT , tagwise dispersions - Fold change	EDGE test: yccT H202 vs yccT NT , tagwise dispersions - Weighted difference	EDGE test: yccT H202 vs yccT NT , tagwise dispersions - Bonferroni	EDGE test: yccT H202 vs yccT NT , tagwise dispersions - FDR p-value correction	yccT NT - yccT.1.S28 - Expression values	yccT NT - yccT.2.S29 - Expression values	yccT NT - yccT.3.S30 - Expression values	yccT NT - Means	yccT H202 - yccT.1.H2O2 - Expression values	yccT H202 - yccT.2.H2O2 - Expression values	yccT H202 - yccT.3.H2O2 - Expression values	yccT H202 - Means
yjcC	241	17	106.6667	1.557491	3.62E-12	3.648155	3.95E-05	1.6E-08	2.32E-11	201	189	184	191.3333	297	178	419	298
yjeA	833	735	-778.333	-7.57746	1.87E-16	-3.21668	-4.8E-05	8.29E-13	1.8E-15	934	905	851	896.6667	116	101	138	118.3333
yjeE	634	547	-582.333	-4.83956	1.06E-07	-2.0466	-2.9E-05	0.000472	4.05E-07	781	725	696	734	149	159	147	151.6667
yjeH	442	6	247.3333	1.910429	9.54E-12	4.510272	7.44E-05	4.23E-08	5.89E-11	268	273	274	271.6667	691	249	617	519
yjeM	339	281	-303.667	-5.0852	3.81E-08	-2.14934	-1.6E-05	0.000169	1.56E-07	411	368	355	378	74	77	72	74.33333
yjeQ	1855	1649	-1683.33	-5.29057	4.75E-06	-2.26185	-9E-05	0.021059	1.45E-05	2142	1983	2102	2075.667	334	287	556	392.3333
yjfI	8	1	1	1.111111	0.002254	2.590937	1.14E-06	1	0.004521	8	9	10	9	7	15	8	10
yjfJ	16	9	2	1.086957	2.89E-05	2.546456	2.79E-06	0.128011	7.92E-05	22	16	31	23	17	26	32	25
yjfN	10202	9146	-9652.33	-27.4689	0	-11.7133	-0.00071	0	0	10477	10090	9484	10017	338	275	481	364.6667
yjfO	5720	5288	-5456.67	-32.0626	2.68E-36	-13.6724	-0.00041	1.19E-32	7.34E-35	5861	5594	5442	5632.333	154	141	232	175.6667
yjfQ	84	39	47.66667	1.074908	1.63E-10	2.537329	7.63E-05	7.22E-07	8.79E-10	626	625	658	636.3333	665	709	678	684
yjfR	2220	1481	1825	15.91826	2.43E-76	37.70626	0.00035	1.08E-72	1.44E-74	122	114	131	122.3333	1905	2334	1603	1947.333
yjfY	57	4	-6.33333	-1.0669	2.95E-06	2.20459	9.49E-06	0.01308	9.3E-06	107	96	100	101	99	64	121	94.66667
yjgA	172	120	133.6667	1.359641	4.35E-17	3.202274	6.39E-05	1.93E-13	4.37E-16	362	385	368	371.6667	494	488	534	505.3333
yjgD	1449	156	788.3333	1.697434	5.7E-12	3.985841	0.000263	2.52E-08	3.6E-11	1197	1149	1045	1130.333	1957	1305	2494	1918.667
yjiG	263	186	-206	-5.944	5.46E-08	-2.52926	-1.2E-05	0.000242	2.18E-07	224	225	294	247.6667	38	31	56	41.66667
yjiH	300	195	-241	-5.30357	9.84E-08	-2.24117	-1.3E-05	0.000436	3.76E-07	288	251	352	297	60	52	56	56
yjiJ	356	153	238.3333	1.853222	6.24E-24	4.350786	7.29E-05	2.77E-20	9.63E-23	278	261	299	279.3333	431	505	617	517.6667
yjiN	325	54	140.3333	1.706376	2.1E-11	4.010925	4.64E-05	9.32E-08	1.25E-10	233	164	199	198.6667	349	179	489	339
yjjI	4524	4212	-4272.67	-6.81579	1.9E-07	-2.86486	-0.00025	0.000843	7.08E-07	5131	5044	4847	5007.333	962	607	635	734.6667
yjjP	623	517	-571.667	-6.51447	3.79E-13	-2.75829	-3.4E-05	1.68E-09	2.7E-12	691	621	714	675.3333	91	116	104	103.6667
yjjQ	12	4	6.333333	1.575758	4.27E-07	3.670791	2.33E-06	0.001892	1.51E-06	8	11	14	11	17	15	20	17.33333
yjjW	1389	1239	-1269.33	-6.28155	1.08E-09	-2.64275	-7.3E-05	4.77E-06	5.34E-09	1601	1455	1473	1509.667	293	216	212	240.3333
yjjY	1312	108	32	1.019831	5.69E-05	2.38881	0.000175	0.251947	0.000149	1575	1683	1583	1613.667	1753	936	2248	1645.667
yljA	3085	1549	2052.333	1.8386	4.34E-14	4.309938	0.000632	1.92E-10	3.36E-13	2410	2619	2313	2447.333	3959	4142	5398	4499.667
ymdF	930	46	258.3333	1.773453	4.13E-06	4.212747	8.36E-05	0.018316	1.27E-05	368	340	294	334	305	1201	271	592.3333
ynaF	17165	14840	-16036.7	-5.99948	8.88E-07	-2.54943	-0.00091	0.003936	3.02E-06	19865	19830	18038	19244.33	3198	2700	3725	3207.667
ynaJ	2773	540	1281	1.911311	1.49E-10	4.526595	0.000386	6.61E-07	8.11E-10	1460	1404	1353	1405.667	1990	4126	1944	2686.667
yncC	19	9	3	1.113924	4.79E-06	2.606615	3.32E-06	0.021234	1.46E-05	30	29	20	26.33333	20	29	39	29.33333
yncJ	43	8	26	1.987342	1.18E-12	4.656761	7.55E-06	5.24E-09	8.09E-12	24	23	32	26.33333	59	32	66	52.33333

Feature ID	Experiment - Range (original values)	Experiment - IQR (original values)	Experiment - Difference (original values)	Experiment - Fold Change (original values)	EDGE test: yccT H202 vs yccT NT , tagwise dispersions - P-value	EDGE test: yccT H202 vs yccT NT , tagwise dispersions - Fold change	EDGE test: yccT H202 vs yccT NT , tagwise dispersions - Weighted difference	EDGE test: yccT H202 vs yccT NT , tagwise dispersions - Bonferroni	EDGE test: yccT H202 vs yccT NT , tagwise dispersions - FDR p-value correction	yccT NT - yccT.1.S28 - Expression values	yccT NT - yccT.2.S29 - Expression values	yccT NT - yccT.3.S30 - Expression values	yccT NT - Means	yccT H202 - yccT.1.H2O2 - Expression values	yccT H202 - yccT.2.H2O2 - Expression values	yccT H202 - yccT.3.H2O2 - Expression values	yccT H202 - Means
yneA	19485	12071	-16227	-39.3617	0	-16.868	-0.00121	0	0	19631	12442	17877	16650	371	146	752	423
yneB	19671	11547	-15999	-30.7933	1.91E-14	-13.2482	-0.00118	8.46E-11	1.52E-13	19867	11944	17797	16536	397	196	1018	537
yneC	3328	1903	-2705.33	-39.283	2.16E-32	-16.6977	-0.0002	9.56E-29	4.93E-31	3369	1963	2996	2776	60	41	111	70.66667
yneG	78	5	28.66667	1.219949	1.19E-11	2.888808	1.92E-05	5.26E-08	7.26E-11	135	119	137	130.3333	148	197	132	159
yneH	935	217	451	2.919149	9.75E-16	6.909892	0.000108	4.32E-12	8.66E-15	231	221	253	235	448	1156	454	686
yneI	1797	1116	1346.667	3.606452	1.33E-28	8.519718	0.000303	5.89E-25	2.6E-27	505	489	556	516.6667	1621	2286	1683	1863.333
ynfA	93	57	-70.6667	-8.06667	5.46E-08	-3.40155	-4.4E-06	0.000242	2.18E-07	99	64	79	80.66667	7	6	17	10
ynfK	3500	3210	-3333.67	-35.4862	2.15E-61	-15.0838	-0.00025	9.53E-58	1E-59	3581	3405	3305	3430.333	81	95	114	96.66667
ynhA	6145	1821	3427.333	24.96737	3.7E-34	59.27076	0.00065	1.64E-30	9.16E-33	147	137	145	143	2463	6282	1966	3570.333
ynhG	12063	1631	5620	4.605646	3.84E-13	10.96595	0.00121	1.7E-09	2.73E-12	1633	1547	1496	1558.667	4799	13559	3178	7178.667
yniB	2121	1855	-1913.67	-5.79215	1.42E-06	-2.47246	-0.00011	0.006276	4.67E-06	2381	2317	2241	2313	386	260	552	399.3333
yoaE	630	495	-555.667	-6.14506	4.34E-12	-2.59648	-3.2E-05	1.92E-08	2.78E-11	729	607	655	663.6667	112	99	113	108
yobF	6920	6562	-6669.33	-24.0242	0	-10.2118	-0.00049	0	0	7172	6835	6870	6959	273	252	344	289.6667
yohC	25	13	-6.66667	-1.13889	4.31E-05	2.07349	4.58E-06	0.190784	0.000115	61	47	56	54.66667	38	63	43	48
yohG	24	1	13.33333	1.184332	2.01E-12	2.789062	1.01E-05	8.89E-09	1.34E-11	73	71	73	72.33333	88	74	95	85.66667
yohL	158	38	-30	-1.13353	3.84E-05	2.07206	2.13E-05	0.169972	0.000103	272	265	227	254.6667	240	138	296	224.6667
yojN	2416	2168	-2223	-5.78751	9.64E-08	-2.46419	-0.00012	0.000427	3.7E-07	2786	2602	2674	2687.333	434	370	589	464.3333
ypeC	4253	2242	3266.667	8.759303	6.42E-41	20.5685	0.000642	2.84E-37	2.17E-39	417	408	438	421	3743	2659	4661	3687.667
yqaE	23	7	-0.66667	-1.01923	4.07E-05	2.298202	3.59E-06	0.180325	0.000109	35	28	43	35.33333	26	29	49	34.66667
yqfB	776	630	-670	-7.12805	4.41E-10	-3.04753	-4.1E-05	1.95E-06	2.27E-09	760	843	735	779.3333	105	67	156	109.3333
yqgA	135	37	76	1.429379	6.22E-16	3.38369	3.29E-05	2.75E-12	5.67E-15	179	166	186	177	242	301	216	253
yqgB	171	128	-146	-5.61053	7.86E-07	-2.36	-8E-06	0.003482	2.69E-06	181	157	195	177.6667	42	29	24	31.66667
yqgE	1144	981	-1021.33	-6.58106	2.45E-11	-2.80835	-6E-05	1.08E-07	1.44E-10	1287	1184	1142	1204.333	143	161	245	183
yqgF	619	538	-569.333	-7.4697	1.2E-14	-3.17286	-3.5E-05	5.32E-11	9.74E-14	684	655	633	657.3333	65	104	95	88
yqhA	220	56	30.33333	1.04943	1.08E-09	2.47212	7.03E-05	4.78E-06	5.35E-09	652	574	615	613.6667	630	541	761	644
yqhC	682	476	544.6667	2.011765	7.44E-23	4.759115	0.000157	3.3E-19	1.07E-21	587	493	535	538.3333	1011	1175	1063	1083
yqhD	1508	390	756.3333	1.620963	4.73E-11	3.836516	0.000269	2.1E-07	2.7E-10	1263	1210	1181	1218	1634	2689	1600	1974.333
yqhE	1048	96	393	1.395505	6.04E-09	3.309159	0.000179	2.68E-05	2.71E-08	1064	968	949	993.6667	1125	1997	1038	1386.667
yqiA	1491	1325	-1384	-4.7677	6.71E-06	-2.02421	-6.9E-05	0.029724	2.01E-05	1767	1705	1782	1751.333	380	291	431	367.3333
yqjH	283	198	240	4.090129	1.03E-44	9.678968	5.26E-05	4.57E-41	3.84E-43	83	82	68	77.66667	351	322	280	317.6667

Feature ID	Experiment - Range (original values)	Experiment - IQR (original values)	Experiment - Difference (original values)	Experiment - Fold Change (original values)	EDGE test: yccT H202 vs yccT NT , tagwise dispersions - P-value	EDGE test: yccT H202 vs yccT NT , tagwise dispersions - Fold change	EDGE test: yccT H202 vs yccT NT , tagwise dispersions - Weighted difference	EDGE test: yccT H202 vs yccT NT , tagwise dispersions - Bonferroni	EDGE test: yccT H202 vs yccT NT , tagwise dispersions - FDR p-value correction	yccT NT - yccT.1.S28 - Expression values	yccT NT - yccT.2.S29 - Expression values	yccT NT - yccT.3.S30 - Expression values	yccT NT - Means	yccT H202 - yccT.1.H2O2 - Expression values	yccT H202 - yccT.2.H2O2 - Expression values	yccT H202 - yccT.3.H2O2 - Expression values	yccT H202 - Means
yqjI	161	54	91.33333	1.211583	7.34E-14	2.861255	6.26E-05	3.25E-10	5.58E-13	451	421	423	431.6667	477	582	510	523
yrbB	154	64	-73.3333	-1.14589	2.29E-07	2.058851	4.75E-05	0.001013	8.41E-07	574	552	602	576	448	562	498	502.6667
yrdC	2024	1619	-1796.33	-5.08877	1.03E-06	-2.15868	-9.3E-05	0.004585	3.47E-06	2394	2092	2221	2235.667	370	473	475	439.3333
yrfI	127	64	-51.3333	-1.12623	1.17E-07	2.098089	3.92E-05	0.00052	4.44E-07	485	438	451	458	358	475	387	406.6667
ysaA	1193	1001	-1099	-12.7331	4.53E-24	-5.3536	-7.5E-05	2.01E-20	7.12E-23	1261	1098	1219	1192.667	97	116	68	93.66667
yshA	26	16	19.33333	4.625	3.67E-18	10.62005	4.13E-06	1.62E-14	3.95E-17	4	7	5	5.333333	30	23	21	24.66667
ytfF	326	232	282.3333	2.404643	5.12E-34	5.666447	7.31E-05	2.27E-30	1.25E-32	201	184	218	201	433	507	510	483.3333
zntA	458	28	-136.333	-1.10626	9.55E-06	2.133666	0.000125	0.042309	2.81E-05	1514	1366	1378	1419.333	1350	1056	1443	1283
znuA	1738	1700	-1694.67	-13.8709	9.73E-24	-5.93123	-0.00012	4.31E-20	1.48E-22	1838	1807	1834	1826.333	107	100	188	131.6667
zraP	1147	355	740.3333	2.063188	3.43E-18	4.861772	0.000209	1.52E-14	3.75E-17	743	614	732	696.3333	1462	1087	1761	1436.667
zwf	649	229	242	1.115808	1.84E-07	2.63924	0.000267	0.000815	6.85E-07	2222	1993	2054	2089.667	2578	1929	2488	2331.667
16S rRNA-4	33	14	22.33333	3.16129	2.24E-18	7.347457	5.19E-06	9.94E-15	2.48E-17	13	13	5	10.33333	33	27	38	32.66667
16S rRNA-(	6	0	2.666667	#DIV/0!	0.0026	36.9285	4.91E-07	1	0.005149	0	0	0	0	6	0	2	2.666667
23S rRNA	261	143	200.6667	5.666667	3.48E-44	13.38583	4.16E-05	1.54E-40	1.26E-42	44	41	44	43	302	187	242	243.6667
23S rRNA-1	51	38	42.33333	4.735294	5.12E-31	11.04877	9E-06	2.27E-27	1.11E-29	11	10	13	11.33333	61	49	51	53.66667
23S rRNA-1	7	2	2.333333	1.777778	0.002929	4.019118	7.46E-07	1	0.005724	4	2	3	3	2	9	5	5.333333
23S rRNA-4	80	57	64.66667	3.811594	2.06E-34	8.969932	1.44E-05	9.15E-31	5.2E-33	29	22	18	23	98	79	86	87.66667
23S rRNA-(	1337	713	993.6667	5.9933	2.13E-38	14.15371	0.000205	9.43E-35	6.51E-37	166	217	214	199	1503	927	1148	1192.667
5S rRNA-7	5	2	3	10	0.000343	15.82564	5.87E-07	1	0.000795	0	0	1	0.333333	5	3	2	3.333333
SEN0280A	8	2	4.333333	1.619048	2.73E-05	3.757861	1.54E-06	0.121186	7.53E-05	7	9	5	7	13	13	8	11.33333
SEN0710	57	15	29.33333	2.1	2.87E-13	4.940611	8.25E-06	1.27E-09	2.07E-12	27	29	24	26.66667	45	81	42	56
SEN0918	8	2	-3.33333	-1.10101	1.51E-05	2.138707	3.24E-06	0.066874	4.33E-05	39	35	35	36.33333	33	31	35	33
SEN1714	7	3	4	2.714286	3.9E-05	6.03738	9.83E-07	0.172681	0.000105	2	0	5	2.333333	7	7	5	6.333333
SEN1759	120	38	75.66667	2.681481	2.6E-23	6.3213	1.86E-05	1.15E-19	3.86E-22	54	28	53	45	123	91	148	120.6667
SEN1971A	13	6	1.333333	1.067797	9.43E-05	2.508058	2.33E-06	0.417851	0.00024	22	16	21	19.66667	24	13	26	21
SEN2225	8	2	-1.33333	-1.08333	0.001529	2.165783	1.59E-06	1	0.003161	20	16	16	17.33333	12	18	18	16
SEN2375A	41	5	20.33333	1.267544	2.95E-14	2.990605	1.18E-05	1.31E-10	2.32E-13	83	65	80	76	85	106	98	96.33333
SEN3573	114	26	-62.3333	-1.1374	4.58E-08	2.077114	4.33E-05	0.000203	1.85E-07	551	477	520	516	437	451	473	453.6667
SEN3578	4	2	2.333333	1.28	0.000236	2.981724	1.31E-06	1	0.000563	10	7	8	8.333333	11	10	11	10.66667
SEN3742A	74	12	3	1.022333	1.13E-08	2.396117	1.46E-05	5.01E-05	4.92E-08	136	133	134	134.3333	122	108	182	137.3333

Feature ID	Experiment - Range (original values)	Experiment - IQR (original values)	Experiment - Difference (original values)	Experiment - Fold Change (original values)	EDGE test: yccT H202 vs yccT NT , tagwise dispersions - P-value	EDGE test: yccT H202 vs yccT NT , tagwise dispersions - Fold change	EDGE test: yccT H202 vs yccT NT , tagwise dispersions - Weighted difference	EDGE test: yccT H202 vs yccT NT , tagwise dispersions - Bonferroni	EDGE test: yccT H202 vs yccT NT , tagwise dispersions - FDR p-value correction	yccT NT - yccT.1.S28 - Expression values	yccT NT - yccT.2.S29 - Expression values	yccT NT - yccT.3.S30 - Expression values	yccT NT - Means	yccT H202 - yccT.1.H2O2 - Expression values	yccT H202 - yccT.2.H2O2 - Expression values	yccT H202 - yccT.3.H2O2 - Expression values	yccT H202 - Means
SEN3859	782	553	-682.667	-18.9649	3E-23	-8.08181	-4.9E-05	1.33E-19	4.41E-22	802	583	777	720.6667	30	20	64	38
SEN4259	318	259	-285.667	-4.98605	4.99E-07	-2.11925	-1.5E-05	0.00221	1.75E-06	374	321	377	357.3333	62	59	94	71.66667
