# Supplementary material for: Transcriptional Profiling and Molecular Characterization of the yccT Mutant Link: A Novel STY1099 Protein with the Peroxide Stress Response and Cell Division of Salmonella enterica Serovar Enteritidis
Source: Biology (Basel). 2019 Nov 13;8(4):86. doi: 10.3390/biology8040086 (PMC6955953; doi:10.3390/biology8040086)
Supplement: Supplementary file 1 [file biology-08-00086-s001.zip › supplementary files/Table S4.pdf]

Feature ID	Experiment - Range (original values)	Experiment - IQR (original values)	Experiment - Difference (original values)	Experiment - Fold Change (original values)	EDGE test: yccT H202 vs WT H2O2 , tagwise dispersions - P-value	EDGE test: yccT H202 vs WT H2O2 , tagwise dispersions - Fold change	EDGE test: yccT H202 vs WT H2O2 , tagwise dispersions - Weighted difference	EDGE test: yccT H202 vs WT H2O2 , tagwise dispersions - Bonferroni	EDGE test: yccT H202 vs WT H2O2 , tagwise dispersions - FDR p-value correction	WT H2O2 - WT.1.H2O2 - Expression values	WT H2O2 - WT.2.H2O2 - Expression values	WT H2O2 - WT.3.H2O2 - Expression values	WT H2O2 - Means	yccT H202 - yccT.1.H2O2 - Expression values	yccT H202 - yccT.2.H2O2 - Expression values	yccT H202 - yccT.3.H2O2 - Expression values	yccT H202 - Means
citE	83	41	59	2.752475	1.22E-06	2.678776	8.19E-06	0.005428	0.001809	40	37	24	33.66667	107	78	93	92.66667
citF	576	150	359.6667	4.192308	0.000132	4.186078	5.05E-05	0.584529	0.030765	226	76	36	112.6667	603	202	612	472.3333
citT	401	151	247.3333	2.562105	0.000215	2.551634	3.46E-05	0.95106	0.04135	263	100	112	158.3333	459	257	501	405.6667
dmsA3	1097	458	605.3333	3.759878	9.9E-05	3.871091	8.7E-05	0.438689	0.027483	445	86	127	219.3333	1183	585	706	824.6667
hycD	36	19	25	2.415094	7.69E-05	2.355582	3.46E-06	0.340625	0.027483	19	16	18	17.66667	39	52	37	42.66667
hycF	30	11	18.33333	2.71875	9.08E-05	2.640485	2.53E-06	0.402513	0.027483	11	7	14	10.66667	28	22	37	29
kdgT	856	55	382.3333	5.248148	4.11E-06	5.334886	5.52E-05	0.018209	0.004552	95	55	120	90	911	150	356	472.3333
narG	20431	905	-11391.7	-10.2993	2.56E-05	-10.9844	-0.00174	0.113638	0.016234	902	21078	15870	12616.67	1221	1807	647	1225
narH	15737	793	-9396.33	-14.4682	3.22E-05	-15.3186	-0.00142	0.142576	0.017822	464	15960	13858	10094	613	1257	223	697.6667
narI	14939	991	-9475.33	-10.715	3.63E-05	-11.2411	-0.00143	0.161	0.017889	1179	15182	14991	10450.67	846	1837	243	975.3333
narJ	7943	369	-4926.33	-16.0652	1.83E-05	-16.9614	-0.00074	0.081128	0.016226	284	8031	7445	5253.333	262	631	88	327
narK	906	78	-481	-6.96281	2.31E-05	-7.4809	-7.4E-05	0.102441	0.016234	76	956	653	561.6667	57	135	50	80.66667
nirC	197	14	-102	-3.37209	7.77E-05	-3.58006	-1.6E-05	0.344069	0.027483	52	230	153	145	41	55	33	43
SEN0167	1946	129	812.3333	4.637313	0.000105	4.751957	0.000118	0.466928	0.027483	213	115	342	223.3333	2061	301	745	1035.667
SEN0271	38	14	24.66667	2.396226	0.000124	2.336934	3.39E-06	0.55161	0.030645	18	11	24	17.66667	32	46	49	42.33333
SEN0541	113	34	-60	-2.29496	0.000101	-2.31435	-8.6E-06	0.447532	0.027483	90	80	149	106.3333	57	46	36	46.33333
SEN0992	329	75	203.3333	4.652695	0.000105	4.667723	2.86E-05	0.467212	0.027483	106	30	31	55.66667	359	60	358	259
SEN1163	456	8	-282.667	-8.37391	3.92E-07	-8.98573	-4.3E-05	0.001735	0.000867	47	486	430	321	30	39	46	38.33333
SEN1249	46	21	32.66667	2.166667	0.000243	2.121918	4.51E-06	1	0.044862	37	23	24	28	68	45	69	60.66667
SEN3184	41	29	31.66667	2.117647	0.000143	2.062383	4.35E-06	0.634249	0.031712	24	26	35	28.33333	55	65	60	60
yccT	230	167	-188	#DIV/0!	2.05E-59	-1525.68	-2.7E-05	9.08E-56	9.08E-56	167	167	230	188	0	0	0	0
23S rRNA-	41	24	29	2.175676	0.000181	2.123705	4.01E-06	0.802655	0.037892	29	25	20	24.66667	61	49	51	53.66667
