# Supplementary material for: Transcriptional Profiling and Molecular Characterization of the yccT Mutant Link: A Novel STY1099 Protein with the Peroxide Stress Response and Cell Division of Salmonella enterica Serovar Enteritidis
Source: Biology (Basel). 2019 Nov 13;8(4):86. doi: 10.3390/biology8040086 (PMC6955953; doi:10.3390/biology8040086)
Supplement: Supplementary file 1 [file biology-08-00086-s001.zip › supplementary files/Table S5.pdf]

Feature ID	Experiment - Range (original values)	Experiment - IQR (original values)	Experiment - Difference (original values)	Experiment - Fold Change (original values)	EDGE test: yccT NT vs WT NT , tagwise dispersions - P-value	EDGE test: yccT NT vs WT NT , tagwise dispersions - Fold change	yccT NT vs WT NT ABS|FC|	yccT NT vs WT NT Log2FC	yccT NT vs WT NT Log2FC +-	EDGE test: yccT NT vs WT NT , tagwise dispersions - FDR p-value correction	WT NT - WT.1.S22 - Expression values	WT NT - WT.2.S23 - Expression values	WT NT - WT.3.S24 - Expression values	WT NT - Means	yccT NT - yccT.1.S28 - Expression values	yccT NT - yccT.2.S29 - Expression values	yccT NT - yccT.3.S30 - Expression values	yccT NT - Means
aadA	116	87	86.66667	1.825397	0.000199	1.619549	1.619549438	0.695592508	0.695593	0.002032	139	95	81	105	197	196	182	191.6667
aarF	1711	661	-1009.67	-1.21045	0.047462	-1.38852	1.388517399	0.473545255	-0.47355	0.151081	5367	5830	6225	5807.333	5173	4706	4514	4797.667
aas	248	33	106.3333	1.152559	0.926377	1.011941	1.011940795	0.017124886	0.017125	0.98554	717	745	629	697	877	712	821	803.3333
aat	116	5	14.33333	1.035159	0.466804	-1.09256	1.092557862	0.127709687	-0.12771	0.674848	461	417	345	407.6667	425	422	419	422
abc	216	40	-118.667	-1.47278	0.000747	-1.70051	1.700513312	0.765970299	-0.76597	0.006051	280	392	437	369.6667	246	221	286	251
abrB	134	30	68.33333	1.29454	0.276288	1.139427	1.139426923	0.1883084	0.188308	0.48944	259	229	208	232	306	253	342	300.3333
accA	486	179	309.6667	1.242306	0.539545	1.086148	1.086148456	0.119221305	0.119221	0.729102	1309	1179	1346	1278	1665	1488	1610	1587.667
accB	999	352	553.3333	1.23603	0.624775	1.079238	1.079238268	0.11001341	0.110013	0.788712	2396	2107	2530	2344.333	3106	2748	2839	2897.667
accC	1197	831	578	1.14254	0.964975	-1.00749	1.007488494	0.010763363	-0.01076	1	3823	3688	4654	4055	4885	4134	4880	4633
accD	770	352	370	1.143541	0.958946	1.007841	1.007840717	0.011267648	0.011268	1	2879	2527	2327	2577.667	3097	2841	2905	2947.667
aceA	170	48	66.33333	1.414583	0.194237	1.254645	1.254645022	0.327279239	0.327279	0.388046	177	215	88	160	258	167	254	226.3333
aceB	231	87	122.6667	1.618487	0.014829	1.44033	1.440330442	0.526399834	0.5264	0.062518	275	188	132	198.3333	347	253	363	321
aceE	18650	1178	-7101	-2.23345	0.000474	-2.45639	2.456388316	1.296538646	-1.29654	0.004196	23723	7743	7108	12858	6268	5930	5073	5757
aceF	27998	2615	-6238.67	-1.71408	0.068964	-1.85138	1.851381155	0.888601942	-0.8886	0.196768	33152	6620	5154	14975.33	9241	9235	7734	8736.667
aceK	130	55	81.66667	1.371775	0.097795	1.208682	1.208682423	0.273435231	0.273435	0.24691	236	227	196	219.6667	296	282	326	301.3333
ackA	3893	1355	-2687.33	-1.25603	0.035938	-1.44154	1.441539793	0.527610662	-0.52761	0.12276	11594	14015	13941	13183.33	11127	10122	10239	10496
acnA	2467	1513	1143	1.437763	0.174224	1.286483	1.286482793	0.363432161	0.363432	0.361757	3890	2193	1750	2611	4217	3339	3706	3754
acnB	9138	2485	-5427.33	-1.36168	0.009685	-1.54912	1.549115879	0.631445066	-0.63145	0.045383	20898	22579	17823	20433.33	16239	13441	15338	15006
acpP	2983	1361	-1424	-1.08569	0.201427	-1.24282	1.24281802	0.313615065	-0.31362	0.398911	17611	17829	18689	18043	17756	15706	16395	16619
acpS	234	64	85.66667	1.110253	0.867741	-1.02097	1.020969685	0.02994003	-0.02994	0.955348	876	773	682	777	916	835	837	862.6667
acpT	118	44	-51.6667	-1.10908	0.049534	-1.26528	1.265283	0.339460102	-0.33946	0.155223	549	515	512	525.3333	519	431	471	473.6667
acrA	1365	818	881	1.264485	0.493251	1.112322	1.112321512	0.153573853	0.153574	0.697625	3769	3014	3210	3331	4379	4028	4229	4212
acrB	2288	902	1042.333	1.193838	0.770375	1.048447	1.048446713	0.068253538	0.068254	0.887872	6159	4716	5257	5377.333	7004	5891	6364	6419.667
acrD	272	133	149.3333	1.284264	0.310803	1.133871	1.133870803	0.181256265	0.181256	0.526038	628	495	453	525.3333	725	612	687	674.6667
acrE	32	14	-5	-1.06073	0.218268	-1.21459	1.214585967	0.280464605	-0.28046	0.421599	70	90	102	87.33333	79	92	76	82.33333
acrF	144	83	63.66667	1.171917	0.75742	1.039518	1.039518419	0.055915321	0.055915	0.880907	453	349	309	370.3333	423	447	432	434
acrR	293	206	170	1.214556	0.575033	1.072315	1.072315462	0.100729391	0.100729	0.753615	935	729	713	792.3333	972	909	1006	962.3333
acs	739	390	527.6667	2.386165	4.19E-08	2.117956	2.11795616	1.082672727	1.082673	1.7E-06	420	434	288	380.6667	810	888	1027	908.3333
ada	273	102	102	1.224176	0.522828	1.090556	1.090555736	0.125063506	0.125064	0.718393	592	454	319	455	585	530	556	557
add	1146	404	449	1.236856	0.524468	1.098477	1.09847651	0.13550402	0.135504	0.719276	2407	1832	1448	1895.667	2594	2236	2204	2344.667
adhE	2434	1517	767.6667	1.054274	0.63517	-1.08214	1.082136861	0.113882973	-0.11388	0.797616	13923	15176	13334	14144.33	15768	13659	15309	14912
adhP	6827	3350	2467	1.426718	0.258898	1.292673	1.292672785	0.370357131	0.370357	0.470697	9802	4567	2975	5781.333	8996	7832	7917	8248.333
adi	95	54	-10.6667	-1.02063	0.210081	-1.15941	1.159405221	0.213384887	-0.21338	0.410933	573	532	478	527.6667	537	480	534	517
adiY	523	345	375	2.244469	3.66E-06	1.993317	1.993317353	0.995171418	0.995171	7.99E-05	405	225	274	301.3333	662	748	619	676.3333
adk	464	167	277.3333	1.147701	0.973954	1.004868	1.004867617	0.00700545	0.007005	1	1936	1733	1964	1877.667	2197	2103	2165	2155
aefA	961	444	600.6667	1.443952	0.064389	1.276513	1.276513192	0.352208447	0.352208	0.187578	1589	1350	1120	1353	2081	1794	1986	1953.667

Feature ID	Experiment - Range (original values)	Experiment - IQR (original values)	Experiment - Difference (original values)	Experiment - Fold Change (original values)	EDGE test: yccT NT vs WT NT , tagwise dispersions - P-value	EDGE test: yccT NT vs WT NT , tagwise dispersions - Fold change	yccT NT vs WT NT ABS|FC|	yccT NT vs WT NT Log2FC	yccT NT vs WT NT Log2FC +-	EDGE test: yccT NT vs WT NT , tagwise dispersions - FDR p-value correction	WT NT - WT.1.S22 - Expression values	WT NT - WT.2.S23 - Expression values	WT NT - WT.3.S24 - Expression values	WT NT - Means	yccT NT - yccT.1.S28 - Expression values	yccT NT - yccT.2.S29 - Expression values	yccT NT - yccT.3.S30 - Expression values	yccT NT - Means
aegA	2185	1002	1305.333	1.465028	0.121383	1.290997	1.290996544	0.368485138	0.368485	0.284598	3527	2216	2678	2807	4401	3680	4256	4112.333
aer	1516	651	627.6667	1.321496	0.377892	1.1737	1.173700479	0.231064289	0.231064	0.594928	1906	2726	1225	1952.333	2557	2442	2741	2580
aes	30	4	14.66667	1.13253	1	-1.0033	1.003295216	0.004746176	-0.00475	1	114	113	105	110.6667	131	135	110	125.3333
agp	824	137	-526.667	-1.22594	0.034796	-1.40822	1.40822208	0.493874868	-0.49387	0.119798	2445	3041	3087	2857.667	2422	2263	2308	2331
ahpC	5190	1863	1900	1.140135	0.957436	1.008958	1.008957917	0.012866002	0.012866	1	16262	13052	11361	13558.33	16551	14915	14909	15458.33
ahpF	482	243	271.6667	1.190688	0.722034	1.047392	1.047391828	0.066801253	0.066801	0.855666	1593	1350	1331	1424.667	1813	1584	1692	1696.333
aidB	1663	379	-306	-1.06622	0.23594	-1.20512	1.205122985	0.269180384	-0.26918	0.444591	5818	4808	4155	4927	4902	4532	4429	4621
ais	495	268	297	1.375791	0.147399	1.221608	1.221607881	0.288781275	0.288781	0.321896	975	689	707	790.3333	956	1184	1122	1087.333
alaS	1675	721	1140	1.203996	0.766727	1.050186	1.050185794	0.070644586	0.070645	0.886011	5422	5377	5966	5588.333	7052	6143	6990	6728.333
aldB	972	662	454	1.351212	0.25546	1.204426	1.204425629	0.268345313	0.268345	0.467472	1903	931	1044	1292.667	1902	1706	1632	1746.667
alkA	43	10	13.33333	1.089286	0.708864	-1.04701	1.047008862	0.066273653	-0.06627	0.847948	155	145	148	149.3333	188	155	145	162.6667
alkB	104	28	39.66667	1.18652	0.686924	1.049489	1.049489264	0.069687408	0.069687	0.833222	251	219	168	212.6667	272	238	247	252.3333
allA	138	32	74	1.337386	0.222631	1.170944	1.170944345	0.227672506	0.227673	0.426986	238	182	238	219.3333	320	290	270	293.3333
allB	123	95	82.66667	1.632653	0.007481	1.454499	1.4544994	0.540522701	0.540523	0.036954	185	112	95	130.6667	218	215	207	213.3333
allC	273	144	174	1.75762	0.00143	1.545561	1.545560864	0.628130468	0.62813	0.010103	286	172	231	229.6667	445	375	391	403.6667
allD	158	91	103.3333	1.817942	0.000253	1.603524	1.6035237	0.681245676	0.681246	0.002428	149	113	117	126.3333	210	208	271	229.6667
allP	13	6	8	1.727273	0.077531	1.515525	1.51552549	0.599818118	0.599818	0.213115	14	8	11	11	21	19	17	19
allR	371	295	219.6667	1.20067	0.638366	1.061951	1.061950624	0.086716688	0.086717	0.80017	1301	1006	977	1094.667	1329	1348	1266	1314.333
alr	115	48	45.33333	1.076447	0.633508	-1.06459	1.064594422	0.090303913	-0.0903	0.796785	547	592	640	593	643	610	662	638.3333
amiA	224	85	108.6667	1.166072	0.820462	1.028179	1.028179209	0.040091745	0.040092	0.92341	728	655	580	654.3333	804	740	745	763
amiB	221	48	-40.6667	-1.02714	0.225297	-1.17417	1.174167362	0.231638061	-0.23164	0.429927	1511	1574	1533	1539.333	1579	1358	1559	1498.667
amiC	230	143	168.6667	1.473783	0.028724	1.300058	1.300057784	0.378575749	0.378576	0.103898	406	348	314	356	539	491	544	524.6667
amn	1741	111	212.3333	1.074468	0.804084	-1.04259	1.042592577	0.060175494	-0.06018	0.910528	3664	2967	1923	2851.333	3248	3027	2916	3063.667
ampD	733	402	327	1.23039	0.517184	1.094595	1.094595091	0.130397292	0.130397	0.715467	1798	1371	1089	1419.333	1822	1773	1644	1746.333
ampE	725	202	207.3333	1.136583	0.945846	1.009408	1.009408062	0.013509515	0.01351	0.998362	1901	1477	1176	1518	1833	1664	1679	1725.333
ampG	227	99	155.6667	1.355403	0.168239	1.187403	1.187403344	0.247810081	0.24781	0.353198	468	393	453	438	620	552	609	593.6667
ampH	336	259	238.3333	1.455414	0.041277	1.287315	1.287315198	0.364365339	0.364365	0.136501	630	487	453	523.3333	789	750	746	761.6667
amtB	56	30	41	1.3	0.267774	1.144211	1.144211362	0.194353575	0.194354	0.480367	137	144	129	136.6667	167	181	185	177.6667
amyA	706	22	201.3333	1.188103	0.696933	1.058805	1.058804762	0.082436589	0.082437	0.839617	1321	1195	695	1070.333	1401	1217	1197	1271.667
ansA	175	43	106	1.213853	0.624729	1.063115	1.063115452	0.088298278	0.088298	0.788712	502	502	483	495.6667	658	545	602	601.6667
ansB	20454	10713	-16090	-1.47567	0.003399	-1.68896	1.688957508	0.756133032	-0.75613	0.02003	44853	53349	51545	49915.67	34442	34140	32895	33825.67
ansP	86	23	45	1.238516	0.450522	1.092921	1.092920717	0.128188749	0.128189	0.659356	204	205	157	188.6667	243	227	231	233.6667
apaG	127	39	47.66667	1.068651	0.605624	-1.06549	1.065486501	0.091512316	-0.09151	0.777315	696	739	648	694.3333	775	716	735	742
apaH	340	129	-112	-1.07381	0.130202	-1.22876	1.228758814	0.297201765	-0.2972	0.297537	1507	1766	1615	1629.333	1640	1426	1486	1517.333
apbA	298	139	91.66667	1.105891	0.870372	-1.02142	1.021417599	0.030572822	-0.03057	0.957136	1033	829	735	865.6667	998	968	906	957.3333
apbE	78	15	29.33333	1.268293	0.378271	1.120923	1.120922722	0.164686821	0.164687	0.595011	131	116	81	109.3333	159	125	132	138.6667

Feature ID	Experiment - Range (original values)	Experiment - IQR (original values)	Experiment - Difference (original values)	Experiment - Fold Change (original values)	EDGE test: yccT NT vs WT NT , tagwise dispersions - P-value	EDGE test: yccT NT vs WT NT , tagwise dispersions - Fold change	yccT NT vs WT NT ABS|FC|	yccT NT vs WT NT Log2FC	yccT NT vs WT NT Log2FC +-	EDGE test: yccT NT vs WT NT , tagwise dispersions - FDR p-value correction	WT NT - WT.1.S22 - Expression values	WT NT - WT.2.S23 - Expression values	WT NT - WT.3.S24 - Expression values	WT NT - Means	yccT NT - yccT.1.S28 - Expression values	yccT NT - yccT.2.S29 - Expression values	yccT NT - yccT.3.S30 - Expression values	yccT NT - Means
apeE	250	149	143	1.265142	0.388191	1.114661	1.114660634	0.156604538	0.156605	0.60502	642	483	493	539.3333	733	610	704	682.3333
aphA	991	490	418.3333	1.179158	0.890735	1.024181	1.02418101	0.034470714	0.034471	0.968063	2262	1876	2867	2335	2839	2752	2669	2753.333
apt	85	53	4.666667	1.00944	0.320191	-1.13338	1.133379182	0.180630608	-0.18063	0.53498	459	535	489	494.3333	535	450	512	499
araA	100	27	56	1.438642	0.048534	1.270421	1.270421256	0.345306957	0.345307	0.152954	143	138	102	127.6667	202	184	165	183.6667
araB	46	11	22	1.22	0.561852	1.076755	1.076755292	0.106690413	0.10669	0.745379	104	108	88	100	115	134	117	122
araC	152	66	88.33333	1.101222	0.793707	-1.0334	1.033396863	0.047394409	-0.04739	0.903705	878	916	824	872.6667	963	944	976	961
araD	65	17	34.33333	1.39313	0.106395	1.234188	1.234187556	0.303561654	0.303562	0.261329	100	94	68	87.33333	111	133	121	121.6667
araE	109	64	66.66667	1.348432	0.134797	1.193839	1.193838602	0.255607808	0.255608	0.303498	233	186	155	191.3333	264	250	260	258
araH	74	20	37.33333	1.118268	0.875647	-1.01958	1.019578036	0.027972199	-0.02797	0.959152	310	330	307	315.6667	381	355	323	353
araJ	160	35	83.66667	1.236347	0.468234	1.090486	1.090485867	0.124971072	0.124971	0.676072	394	367	301	354	461	402	450	437.6667
arcA	3724	508	-355.333	-1.02702	0.354396	-1.17719	1.177193835	0.235351892	-0.23535	0.569372	11443	15167	13914	13508	13270	12840	13348	13152.67
arcB	526	215	131	1.038115	0.552543	-1.0952	1.095204869	0.131200766	-0.1312	0.738781	3767	3241	3303	3437	3688	3518	3498	3568
arcC	28	16	1.666667	1.02809	0.532595	-1.10146	1.1014556	0.13941134	-0.13941	0.724572	73	60	45	59.33333	66	50	67	61
argA	252	101	78.66667	1.222222	0.542039	1.091086	1.091086394	0.125765341	0.125765	0.730911	491	332	239	354	461	404	433	432.6667
argB	81	36	-41.6667	-1.11131	0.05051	-1.26652	1.266518985	0.340868703	-0.34087	0.157501	428	413	407	416	405	371	347	374.3333
argC	175	76	35.66667	1.08033	0.734501	-1.04544	1.045442515	0.064113735	-0.06411	0.865693	548	411	373	444	529	487	423	479.6667
argD	42	21	-4.66667	-1.01515	0.223562	-1.1566	1.156601425	0.209891785	-0.20989	0.427722	331	293	314	312.6667	308	289	327	308
argE	904	280	251.6667	1.051659	0.6165	-1.08233	1.082334852	0.114146908	-0.11415	0.78418	5321	4551	4743	4871.667	5455	5023	4892	5123.333
argG	570	263	-129	-1.11824	0.09839	-1.25863	1.258631471	0.331855922	-0.33186	0.248102	1452	1326	882	1220	1217	1102	954	1091
argH	205	102	-135.667	-1.16902	0.022714	-1.33762	1.337621376	0.419669808	-0.41967	0.086169	900	933	982	938.3333	833	777	798	802.6667
argI	271	181	105.6667	1.28792	0.388958	1.147301	1.147300933	0.198243855	0.198244	0.60579	538	279	284	367	550	465	403	472.6667
argR	402	6	-67.6667	-1.05179	0.182249	-1.20101	1.201013984	0.264252949	-0.26425	0.371457	1254	1617	1252	1374.333	1447	1215	1258	1306.667
argS	2995	1801	2132.333	2.013145	0.000988	1.775053	1.775052791	0.827861932	0.827862	0.007561	2710	1524	2080	2104.667	4311	3881	4519	4237
argT	138	65	92.33333	1.535783	0.009585	1.35496	1.354959634	0.438249873	0.43825	0.044988	188	183	146	172.3333	262	248	284	264.6667
aroA	687	228	414	1.369423	0.144962	1.208297	1.208297082	0.272975211	0.272975	0.318499	1274	1141	947	1120.667	1634	1369	1601	1534.667
aroB	1180	259	-539.667	-1.13874	0.103773	-1.30476	1.304755516	0.383779501	-0.38378	0.257602	3956	4848	4484	4429.333	4130	3668	3871	3889.667
aroC	431	183	190	1.213084	0.589721	1.07075	1.070749546	0.098621066	0.098621	0.765947	1051	868	756	891.6667	1187	968	1090	1081.667
aroD	180	39	99.66667	1.188287	0.704798	1.04782	1.047820036	0.067390954	0.067391	0.845374	565	546	477	529.3333	645	657	585	629
aroE	557	234	265	1.131535	0.963983	-1.0066	1.006601789	0.009493067	-0.00949	1	1983	2217	1844	2014.667	2401	2157	2281	2279.667
aroF	127	24	45.66667	1.049584	0.506937	-1.08662	1.086620275	0.119847871	-0.11985	0.70721	943	917	903	921	1030	929	941	966.6667
aroG	2603	1467	1689	1.62757	0.021792	1.444261	1.444261379	0.530331861	0.530332	0.083384	3419	2545	2110	2691.333	4713	4012	4416	4380.333
aroH	691	420	398	1.442058	0.066909	1.282928	1.282927565	0.359439718	0.35944	0.193269	1190	824	687	900.3333	1378	1244	1273	1298.333
aroK	764	262	-264.667	-1.08248	0.184183	-1.24371	1.243709548	0.314649602	-0.31465	0.373404	3009	3638	3773	3473.333	3364	3102	3160	3208.667
aroL	178	57	60.66667	1.078078	0.678504	-1.05256	1.052561503	0.073904535	-0.0739	0.82754	861	766	704	777	882	823	808	837.6667
aroM	238	55	107.3333	1.11358	0.870517	-1.02065	1.020653606	0.029493321	-0.02949	0.957136	1005	958	872	945	1110	1034	1013	1052.333
aroP	399	240	246.6667	1.602116	0.010951	1.43165	1.431649666	0.517678499	0.517678	0.049413	550	397	282	409.6667	637	651	681	656.3333

Feature ID	Experiment - Range (original values)	Experiment - IQR (original values)	Experiment - Difference (original values)	Experiment - Fold Change (original values)	EDGE test: yccT NT vs WT NT , tagwise dispersions - P-value	EDGE test: yccT NT vs WT NT , tagwise dispersions - Fold change	yccT NT vs WT NT ABS|FC|	yccT NT vs WT NT Log2FC	yccT NT vs WT NT Log2FC +-	EDGE test: yccT NT vs WT NT , tagwise dispersions - FDR p-value correction	WT NT - WT.1.S22 - Expression values	WT NT - WT.2.S23 - Expression values	WT NT - WT.3.S24 - Expression values	WT NT - Means	yccT NT - yccT.1.S28 - Expression values	yccT NT - yccT.2.S29 - Expression values	yccT NT - yccT.3.S30 - Expression values	yccT NT - Means
artI	870	284	-516	-1.23437	0.026134	-1.41304	1.413037042	0.498799286	-0.4988	0.096099	2461	2939	2753	2717.667	2359	2177	2069	2201.667
artJ	667	385	252	1.331143	0.28811	1.193228	1.193228191	0.254869968	0.25487	0.504591	1121	665	497	761	1164	1050	825	1013
artM	240	66	-79.3333	-1.11621	0.064045	-1.27645	1.27645465	0.352142283	-0.35214	0.187011	684	877	725	762	752	637	659	682.6667
artP	299	53	73	1.067844	0.644678	-1.05937	1.059369732	0.083206194	-0.08321	0.804454	1212	1103	913	1076	1192	1154	1101	1149
artQ	189	78	-106.667	-1.13877	0.037617	-1.3024	1.302397972	0.381170359	-0.38117	0.12714	840	907	879	875.3333	826	718	762	768.6667
asd	1153	562	-383.667	-1.07836	0.204427	-1.23925	1.239245856	0.309462435	-0.30946	0.403114	4588	5601	5651	5280	5150	4498	5041	4896.333
asmA	556	376	400.3333	1.295959	0.304448	1.142103	1.142102591	0.191692249	0.191692	0.519849	1488	1329	1241	1352.667	1757	1705	1797	1753
asnA	169	71	89.66667	1.269539	0.388112	1.116444	1.116444082	0.158910995	0.158911	0.60502	396	277	325	332.6667	446	377	444	422.3333
asnB	348	276	222.6667	1.229317	0.512571	1.086292	1.086291781	0.119411668	0.119412	0.712869	1141	906	866	971	1214	1185	1182	1193.667
asnC	61	13	-25.6667	-1.16488	0.01651	-1.32774	1.327739888	0.408972542	-0.40897	0.067551	193	177	174	181.3333	174	132	161	155.6667
asnS	3182	2577	2377.667	1.424609	0.149996	1.25705	1.257050233	0.330042302	0.330042	0.325833	6535	5166	5098	5599.667	8280	7743	7909	7977.333
aspA	108452	64883	-81397	-1.33344	0.016746	-1.52565	1.525654246	0.609428046	-0.60943	0.068244	304500	341582	330451	325511	259595	239617	233130	244114
aspC	1261	434	854	1.227693	0.668155	1.071635	1.071634955	0.099813546	0.099814	0.821019	3538	3810	3904	3750.667	4771	4244	4799	4604.667
aspS	1342	606	-413.667	-1.07098	0.216774	-1.23067	1.23067017	0.299444159	-0.29944	0.420174	5630	6306	6788	6241.333	6236	5446	5801	5827.667
asrA	172	84	103	1.872881	9.91E-05	1.656806	1.656805686	0.72840441	0.728404	0.001175	149	118	87	118	259	202	202	221
asrB	86	51	61.66667	1.719844	0.00064	1.517773	1.517773263	0.601956285	0.601956	0.00536	104	82	71	85.66667	152	133	157	147.3333
asrC	112	53	61.33333	1.287051	0.273734	1.137961	1.137960834	0.186450905	0.186451	0.487114	248	210	183	213.6667	263	267	295	275
astA	75	47	58.33333	1.883838	3.16E-05	1.660002	1.660001993	0.731184974	0.731185	0.000481	73	70	55	66	126	117	130	124.3333
astB	56	23	32	1.484848	0.048678	1.306789	1.306789156	0.386026388	0.386026	0.153246	74	61	63	66	91	86	117	98
astC	57	19	36.33333	1.703226	0.004563	1.508859	1.508859333	0.593458313	0.593458	0.024981	60	59	36	51.66667	78	93	93	88
astD	65	30	38.33333	1.454545	0.04788	1.282825	1.282824959	0.359324329	0.359324	0.151677	95	83	75	84.33333	113	115	140	122.6667
astE	44	9	14	1.256098	0.496995	1.117348	1.117347828	0.160078364	0.160078	0.699966	75	55	34	54.66667	64	64	78	68.66667
atpA	18220	4193	-11750.7	-2.05324	2.83E-05	-2.38526	2.38525906	1.254145964	-1.25415	0.000444	15778	24775	28169	22907.33	11936	9949	11585	11156.67
atpB	2984	402	-1616.33	-1.37936	0.011026	-1.59541	1.595410091	0.673927308	-0.67393	0.04965	4594	6046	6991	5877	4583	4007	4192	4260.667
atpC	6700	5396	-5900.33	-2.79269	2.38E-12	-3.19276	3.192764057	1.674805943	-1.67481	2.06E-10	8711	9787	9077	9191.667	3472	3087	3315	3291.333
atpD	18671	12328	-15686.7	-2.55766	2.57E-09	-2.93814	2.938136798	1.554901568	-1.5549	1.41E-07	22490	27840	26942	25757.33	10881	9169	10162	10070.67
atpE	6205	574	-3421.67	-1.92711	0.000188	-2.2502	2.250196207	1.170050803	-1.17005	0.00196	4429	7373	9535	7112.333	3887	3330	3855	3690.667
atpF	7727	1230	-4555	-1.96648	7.61E-05	-2.29109	2.291088786	1.196033368	-1.19603	0.000952	6104	9789	11911	9268	5081	4184	4874	4713
atpG	9254	3263	-6350	-2.24315	9.91E-07	-2.60039	2.600392047	1.378729147	-1.37873	2.71E-05	8540	12037	13797	11458	5504	4543	5277	5108
atpH	7118	1076	-4106.67	-2.02097	5.85E-05	-2.35738	2.35737634	1.237182094	-1.23718	0.000776	5254	8414	10719	8129	4288	3601	4178	4022.333
atpI	445	149	-262.333	-1.15547	0.047444	-1.3189	1.318900699	0.399335947	-0.39934	0.151081	1857	2058	1934	1949.667	1741	1708	1613	1687.333
avrA	169	99	106.6667	1.788177	0.000634	1.592983	1.592983341	0.67173118	0.671731	0.005324	182	132	92	135.3333	234	231	261	242
avtA	251	84	-152.333	-1.20018	0.009871	-1.36601	1.366008395	0.44996635	-0.44997	0.045847	940	965	835	913.3333	818	714	751	761
b2145	124	59	81.33333	1.509395	0.011599	1.329543	1.329543412	0.410930884	0.410931	0.051756	177	164	138	159.6667	262	223	238	241
bacA	117	18	-46.3333	-1.09411	0.091278	-1.2551	1.255103838	0.327806727	-0.32781	0.236799	486	527	603	538.6667	487	486	504	492.3333
baeR	263	137	127.3333	1.273443	0.33797	1.126742	1.126741715	0.172156842	0.172157	0.551582	576	439	382	465.6667	645	540	594	593

Feature ID	Experiment - Range (original values)	Experiment - IQR (original values)	Experiment - Difference (original values)	Experiment - Fold Change (original values)	EDGE test: yccT NT vs WT NT , tagwise dispersions - P-value	EDGE test: yccT NT vs WT NT , tagwise dispersions - Fold change	yccT NT vs WT NT ABS|FC|	yccT NT vs WT NT Log2FC	yccT NT vs WT NT Log2FC +-	EDGE test: yccT NT vs WT NT , tagwise dispersions - FDR p-value correction	WT NT - WT.1.S22 - Expression values	WT NT - WT.2.S23 - Expression values	WT NT - WT.3.S24 - Expression values	WT NT - Means	yccT NT - yccT.1.S28 - Expression values	yccT NT - yccT.2.S29 - Expression values	yccT NT - yccT.3.S30 - Expression values	yccT NT - Means
baeS	289	42	120	1.18191	0.733774	1.042707	1.042706935	0.060333729	0.060334	0.865412	734	692	553	659.6667	842	718	779	779.6667
barA	354	104	29.66667	1.013138	0.425136	-1.12231	1.122310504	0.166471875	-0.16647	0.637704	2310	2409	2055	2258	2360	2206	2297	2287.667
basR	353	132	72.66667	1.078417	0.723518	-1.04662	1.046620641	0.065738616	-0.06574	0.856966	1126	881	773	926.6667	1016	969	1013	999.3333
basS	372	215	138	1.148921	0.899472	1.016688	1.016687572	0.023876408	0.023876	0.971377	1147	858	775	926.6667	1142	979	1073	1064.667
bax	674	353	229.6667	1.082912	0.768572	-1.04562	1.045621833	0.064361171	-0.06436	0.887283	3164	2656	2490	2770	3021	3009	2969	2999.667
bcfA	81	26	43.33333	1.718232	0.01928	1.51096	1.510959713	0.595465194	0.595465	0.076141	81	34	66	60.33333	115	104	92	103.6667
bcfB	44	22	29.66667	2.236111	4.25E-05	1.961391	1.961391088	0.971877228	0.971877	0.000606	29	19	24	24	63	46	52	53.66667
bcfC	68	19	31.33333	1.286585	0.301009	1.136578	1.136577753	0.184696382	0.184696	0.515966	129	110	89	109.3333	139	126	157	140.6667
bcfD	70	9	20.33333	1.246964	0.522196	1.100491	1.100491011	0.138147362	0.138147	0.718141	89	103	55	82.33333	125	87	96	102.6667
bcfF	51	26	33	1.818182	0.001109	1.606801	1.606801374	0.6841916	0.684192	0.008314	50	42	29	40.33333	68	72	80	73.33333
bcfG	67	26	35.33333	1.469027	0.057164	1.304265	1.304265236	0.383237286	0.383237	0.17184	98	73	55	75.33333	111	122	99	110.6667
bcfH	19	6	7.333333	1.095238	0.78882	-1.03674	1.036736013	0.052048584	-0.05205	0.900807	79	85	67	77	86	81	86	84.33333
bcfHb	36	19	16.33333	1.365672	0.299631	1.199088	1.199087587	0.261937043	0.261937	0.515478	34	62	38	44.66667	57	56	70	61
bcp	533	172	199	1.053114	0.610741	-1.08247	1.08246597	0.114321672	-0.11432	0.781195	3913	3594	3733	3746.667	4127	3905	3805	3945.667
bcr	273	148	191.6667	1.524635	0.014033	1.345429	1.345429	0.42806626	0.428066	0.060078	429	351	316	365.3333	583	499	589	557
bcsA	277	92	177	1.525223	0.02622	1.335687	1.335687145	0.417582128	0.417582	0.096176	363	302	346	337	525	438	579	514
bcsB	347	191	138.3333	1.197243	0.687996	1.053857	1.053856956	0.075679058	0.075679	0.834066	851	611	642	701.3333	958	728	833	839.6667
bcsC	720	235	-1	-1.00062	0.379161	-1.12838	1.128383215	0.174257111	-0.17426	0.595639	1925	1713	1205	1614.333	1758	1478	1604	1613.333
bcsE	618	254	433.6667	1.470184	0.039989	1.291095	1.291095225	0.368595411	0.368595	0.133227	961	964	842	922.3333	1460	1215	1393	1356
bcsF	24	15	12	1.27907	0.448121	1.126448	1.126448015	0.171780736	0.171781	0.658363	40	55	34	43	58	56	51	55
bcsG	327	147	244.6667	1.393144	0.117258	1.218578	1.218578077	0.28519869	0.285199	0.27859	609	650	608	622.3333	935	756	910	867
bcsZ	276	125	108	1.22995	0.506953	1.094156	1.094155626	0.129817953	0.129818	0.70721	629	427	353	469.6667	629	552	552	577.6667
bfd	25	5	11.33333	1.492754	0.135337	1.318281	1.318280719	0.398657615	0.398658	0.304252	29	25	15	23	30	33	40	34.33333
bfr	215	162	111	1.277269	0.352551	1.133711	1.133710774	0.181052635	0.181053	0.567643	536	334	331	400.3333	546	496	492	511.3333
bglA	260	35	94	1.094409	0.783876	-1.03544	1.035443238	0.050248469	-0.05025	0.896874	1081	1046	860	995.6667	1120	1067	1082	1089.667
bglJ	113	29	62.33333	1.722008	0.001758	1.518266	1.518265603	0.602424196	0.602424	0.011931	98	98	63	86.33333	176	127	143	148.6667
bglX	336	219	243.6667	1.215825	0.598684	1.068031	1.0680306	0.094952982	0.094953	0.773381	1212	1076	1099	1129	1412	1318	1388	1372.667
bigA	117	17	54	1.126267	0.923015	-1.01205	1.012052165	0.017283653	-0.01728	0.984632	432	449	402	427.6667	519	444	482	481.6667
bioA	55	9	19	1.238494	0.502315	1.0928	1.092800026	0.128029423	0.128029	0.704228	95	83	61	79.66667	116	88	92	98.66667
bioB	25	10	13.33333	1.470588	0.175993	1.287039	1.287038639	0.364055366	0.364055	0.364064	23	27	35	28.33333	37	40	48	41.66667
bioC	6	1	1.333333	1.166667	1	1.024591	1.024590601	0.035047563	0.035048	1	5	10	9	8	11	9	8	9.333333
bioD	6	4	0	-1	0.679577	-1.13471	1.134710334	0.182324058	-0.18232	0.827568	15	15	11	13.66667	16	10	15	13.66667
bioF	10	8	-1.66667	-1.11905	0.356196	-1.27312	1.273122084	0.348370771	-0.34837	0.571436	19	9	19	15.66667	17	9	16	14
bioH	181	76	50	1.101215	0.840827	-1.02576	1.025760981	0.036694598	-0.03669	0.938702	601	461	420	494	560	535	537	544
birA	246	10	2	1.001632	0.310293	-1.14056	1.140561604	0.189744372	-0.18974	0.525577	1173	1324	1180	1225.667	1373	1183	1127	1227.667
bisC	258	69	106.6667	1.067912	0.607723	-1.0709	1.070896954	0.098819665	-0.09882	0.778497	1535	1604	1573	1570.667	1778	1520	1734	1677.333

Feature ID	Experiment - Range (original values)	Experiment - IQR (original values)	Experiment - Difference (original values)	Experiment - Fold Change (original values)	EDGE test: yccT NT vs WT NT , tagwise dispersions - P-value	EDGE test: yccT NT vs WT NT , tagwise dispersions - Fold change	yccT NT vs WT NT ABS|FC|	yccT NT vs WT NT Log2FC	yccT NT vs WT NT Log2FC +-	EDGE test: yccT NT vs WT NT , tagwise dispersions - FDR p-value correction	WT NT - WT.1.S22 - Expression values	WT NT - WT.2.S23 - Expression values	WT NT - WT.3.S24 - Expression values	WT NT - Means	yccT NT - yccT.1.S28 - Expression values	yccT NT - yccT.2.S29 - Expression values	yccT NT - yccT.3.S30 - Expression values	yccT NT - Means
blc	369	119	-152.333	-1.13324	0.047737	-1.28673	1.286729753	0.363709082	-0.36371	0.151609	1320	1464	1103	1295.667	1222	1095	1113	1143.333
bolA	371	238	156	1.224353	0.53715	1.088525	1.0885254	0.122375072	0.122375	0.727641	887	649	550	695.3333	921	912	721	851.3333
brnQ	195	89	117.3333	1.206331	0.632292	1.060721	1.060720979	0.085045208	0.085045	0.796042	630	531	545	568.6667	698	634	726	686
btuB	1163	175	-279.333	-1.13205	0.129725	-1.31126	1.311256956	0.390950427	-0.39095	0.296753	1854	2313	3017	2394.667	2139	2016	2191	2115.333
btuC	128	73	83	1.386047	0.093214	1.220215	1.220215324	0.287135753	0.287136	0.239522	253	188	204	215	316	277	301	298
btuD	80	44	47.33333	1.244406	0.442496	1.096309	1.096309445	0.132655072	0.132655	0.653349	214	186	181	193.6667	232	230	261	241
btuE	84	31	46.66667	1.202312	0.641719	1.05565	1.055649896	0.078131447	0.078131	0.802528	244	228	220	230.6667	304	259	269	277.3333
btuF	163	26	-97.6667	-1.24396	0.008934	-1.42951	1.429505386	0.515516056	-0.51552	0.04255	421	527	546	498	422	383	396	400.3333
btuR	383	230	287	1.222711	0.574552	1.075201	1.075201096	0.104606514	0.104607	0.753445	1346	1308	1212	1288.667	1594	1538	1595	1575.667
cadA	51543	19697	-38109.3	-5.27027	6.22E-15	-6.08618	6.086180668	2.605537161	-2.60554	8.1E-13	28863	59646	52592	47033.67	9166	9504	8103	8924.333
cadB	43479	19990	-31196.7	-4.50472	3.55E-15	-5.16504	5.165038228	2.368779027	-2.36878	4.92E-13	29404	51265	39625	40098	9414	9504	7786	8901.333
cadC	156	66	51.66667	1.05039	0.509636	-1.08739	1.087386826	0.120865254	-0.12087	0.709455	1003	1072	1001	1025.333	1157	1005	1069	1077
cafA	419	88	-205.667	-1.11888	0.08894	-1.28352	1.283521517	0.360107481	-0.36011	0.232091	1710	2018	2079	1935.667	1732	1660	1798	1730
caiA	152	101	101.6667	1.271111	0.342734	1.12097	1.12096957	0.164747116	0.164747	0.557099	421	362	342	375	473	463	494	476.6667
caiB	193	98	140.3333	1.367044	0.121392	1.202158	1.202157867	0.265626363	0.265626	0.284598	401	392	354	382.3333	531	490	547	522.6667
caiC	262	99	167.6667	1.356991	0.142984	1.198004	1.198003873	0.260632573	0.260633	0.315494	508	509	392	469.6667	651	607	654	637.3333
caiD	101	28	63.33333	1.530726	0.037765	1.334771	1.334771426	0.416592708	0.416593	0.127443	122	101	135	119.3333	202	150	196	182.6667
caiE	1225	660	752	1.584305	0.013707	1.405399	1.405399384	0.490980171	0.49098	0.05891	1627	1228	1006	1287	2231	1888	1998	2039
caiF	2385	1360	1496.667	1.447033	0.119376	1.280875	1.280874669	0.357129318	0.357129	0.281808	4064	3141	2839	3348	5224	4809	4501	4844.667
caiT	49	25	37.33333	1.965517	0.000214	1.723802	1.723802085	0.785594144	0.785594	0.002136	42	32	42	38.66667	80	67	81	76
carA	115	60	32.33333	1.177331	0.748914	1.04725	1.047249619	0.06660536	0.066605	0.876484	251	160	136	182.3333	247	220	177	214.6667
carB	137	29	52	1.108258	0.84882	-1.02339	1.023387332	0.03335228	-0.03335	0.943819	526	492	423	480.3333	560	521	516	532.3333
cbiA	2071	1845	1608.667	1.245087	0.564445	1.096668	1.09666775	0.133126509	0.133127	0.745911	7305	6200	6186	6563.667	8257	8045	8215	8172.333
cbiC	1044	394	503	1.188743	0.828733	1.036085	1.036085475	0.051143028	0.051143	0.930121	2671	2302	3022	2665	3346	3093	3065	3168
cbiD	2321	699	991.6667	1.203405	0.789713	1.047307	1.047307475	0.066685059	0.066685	0.901396	4960	4007	5659	4875.333	6328	5574	5699	5867
cbiE	765	518	436.6667	1.283182	0.517748	1.111697	1.11169719	0.152763873	0.152764	0.715578	1424	1260	1942	1542	2014	1897	2025	1978.667
cbiF	2320	1263	1013	1.425035	0.387069	1.220983	1.220982601	0.288042643	0.288043	0.604066	2226	1314	3610	2383.333	3634	3066	3489	3396.333
cbiG	3884	2112	1652	1.494413	0.353398	1.275789	1.275789462	0.351390267	0.35139	0.568182	3150	1495	5379	3341.333	5317	4401	5262	4993.333
cbiH	2627	1535	1190.667	1.635135	0.249126	1.388634	1.388633929	0.473666327	0.473666	0.460333	1677	717	3230	1874.667	3212	2640	3344	3065.333
cbiJ	2012	1079	912.6667	1.669438	0.230713	1.418524	1.418523546	0.504390098	0.50439	0.437623	1278	455	2357	1363.333	2411	1950	2467	2276
cbiK	2276	1305	931.3333	1.67635	0.300111	1.411347	1.411346915	0.497072652	0.497073	0.51552	1113	371	2647	1377	2418	2054	2453	2308.333
cbiL	1447	1002	677.3333	1.774685	0.220829	1.488982	1.488981772	0.574326093	0.574326	0.424878	668	256	1699	874.3333	1703	1282	1670	1551.667
cbiM	1957	1224	1054.667	1.974138	0.115	1.660991	1.660991068	0.732044316	0.732044	0.275142	851	322	2075	1082.667	2279	1887	2246	2137.333
cbiN	797	508	405.6667	1.95451	0.151946	1.641231	1.641230777	0.714778114	0.714778	0.328426	333	101	841	425	872	722	898	830.6667
cbiO	1224	703	719	2.068351	0.050265	1.75309	1.753089635	0.809899763	0.8099	0.156847	572	243	1204	673	1434	1275	1467	1392
cbiP	1439	814	944	2.024231	0.006879	1.73846	1.738460293	0.797810116	0.79781	0.034483	869	545	1351	921.6667	1984	1683	1930	1865.667

Feature ID	Experiment - Range (original values)	Experiment - IQR (original values)	Experiment - Difference (original values)	Experiment - Fold Change (original values)	EDGE test: yccT NT vs WT NT , tagwise dispersions - P-value	EDGE test: yccT NT vs WT NT , tagwise dispersions - Fold change	yccT NT vs WT NT ABS|FC|	yccT NT vs WT NT Log2FC	yccT NT vs WT NT Log2FC +-	EDGE test: yccT NT vs WT NT , tagwise dispersions - FDR p-value correction	WT NT - WT.1.S22 - Expression values	WT NT - WT.2.S23 - Expression values	WT NT - WT.3.S24 - Expression values	WT NT - Means	yccT NT - yccT.1.S28 - Expression values	yccT NT - yccT.2.S29 - Expression values	yccT NT - yccT.3.S30 - Expression values	yccT NT - Means
cbiT	947	476	325	1.220688	0.791835	1.049451	1.049451025	0.069634842	0.069635	0.902424	1321	1075	2022	1472.667	2015	1581	1797	1797.667
cboQ	1140	563	682.6667	2.253366	0.021151	1.909506	1.909506098	0.933199527	0.9332	0.081425	477	201	956	544.6667	1341	1040	1301	1227.333
cbpA	769	384	322	1.142184	0.945753	1.010198	1.01019828	0.014638491	0.014638	0.998362	2670	2201	1923	2264.667	2692	2483	2585	2586.667
cca	897	74	-520	-1.34083	0.010599	-1.54776	1.547764458	0.630185936	-0.63019	0.048122	1484	2272	2381	2045.667	1500	1503	1574	1525.667
ccmA	50	28	35	2.666667	7.15E-07	2.339969	2.339968904	1.226489358	1.226489	2.06E-05	28	17	18	21	67	46	55	56
ccmB	0	0	0	1	1	1	1	0	0	1	0	0	0	0	0	0	0	0
ccmD	0	0	0	1	1	1	1	0	0	1	0	0	0	0	0	0	0	0
ccmE	0	0	0	1	1	1	1	0	0	1	0	0	0	0	0	0	0	0
ccmF	0	0	0	1	1	1	1	0	0	1	0	0	0	0	0	0	0	0
ccmG	0	0	0	1	1	1	1	0	0	1	0	0	0	0	0	0	0	0
ccmH	71	13	-20.3333	-1.57009	0.031145	-1.8583	1.858297907	0.893981801	-0.89398	0.110344	29	42	97	56	46	26	35	35.66667
cdaR	7693	5144	3078.333	1.292673	0.460053	1.152259	1.152258678	0.204464633	0.204465	0.668359	15364	7671	8519	10518	14256	12870	13663	13596.33
cdd	3070	92	-1255.33	-2.97276	2.61E-05	-3.33931	3.339312166	1.739550966	-1.73955	0.000415	1268	3675	732	1891.667	664	640	605	636.3333
cdsA	460	214	198	1.108394	0.839369	-1.02819	1.028193583	0.040111913	-0.04011	0.937824	1983	1728	1769	1826.667	2188	1854	2032	2024.667
cedA	68	10	22.66667	1.159624	0.873733	1.021709	1.021708895	0.030984203	0.030984	0.958058	154	146	126	142	156	144	194	164.6667
celA	1455	888	-1063.67	-1.47942	0.000849	-1.68464	1.684636959	0.752437723	-0.75244	0.006727	3187	3506	3154	3282.333	2266	2339	2051	2218.667
celB	717	301	-436.667	-1.34392	0.002072	-1.52594	1.525937026	0.609695425	-0.6097	0.013425	1638	1939	1542	1706.333	1222	1346	1241	1269.667
celC	482	116	-263	-1.39949	0.000907	-1.60258	1.602581728	0.680397933	-0.6804	0.007114	766	1096	902	921.3333	711	650	614	658.3333
celD	698	123	-451	-1.50598	0.000373	-1.73085	1.730848146	0.791479157	-0.79148	0.003396	1011	1584	1432	1342.333	886	888	900	891.3333
celF	935	316	-663.333	-2.0676	1.71E-07	-2.39469	2.394687984	1.259837692	-1.25984	5.83E-06	914	1426	1514	1284.667	687	579	598	621.3333
cfa	726	332	249	1.068033	0.657132	-1.07525	1.07524916	0.104671004	-0.10467	0.814931	3360	3598	4022	3660	4086	3711	3930	3909
chaA	88	31	48	1.273245	0.407143	1.117357	1.117357339	0.160090644	0.160091	0.621646	199	138	190	175.6667	226	221	224	223.6667
chaB	70	33	38.33333	1.635359	0.019265	1.45636	1.456359874	0.542366897	0.542367	0.076141	88	55	38	60.33333	108	102	86	98.66667
cheA	8138	2074	3491	1.244285	0.594824	1.094971	1.094971329	0.130893095	0.130893	0.770662	14518	16592	11762	14290.67	19900	15949	17496	17781.67
cheB	1338	477	593.6667	1.252159	0.533935	1.102002	1.10200152	0.140126214	0.140126	0.725949	2329	2806	1928	2354.333	3266	2713	2865	2948
cheM	10969	4632	5002	1.36503	0.283171	1.220835	1.220834669	0.287867837	0.287868	0.498106	18435	13803	8871	13703	19840	17741	18534	18705
cheR	3976	1463	2148.667	1.495961	0.099488	1.334663	1.334662778	0.41647527	0.416475	0.249903	5341	4823	2833	4332.333	6809	6286	6348	6481
cheW	2003	487	928	1.252517	0.562781	1.097392	1.097392338	0.134079408	0.134079	0.745719	3629	4116	3280	3675	5283	3942	4584	4603
cheY	672	256	-25.3333	-1.01188	0.336081	-1.16281	1.162812482	0.217618463	-0.21762	0.550119	1908	2400	2164	2157.333	2454	1782	2160	2132
cheZ	1578	588	-93.3333	-1.02239	0.348015	-1.17346	1.173458366	0.230766656	-0.23077	0.563204	3714	4999	4070	4261	4780	3421	4302	4167.667
cibB	912	481	669	1.36691	0.209027	1.197389	1.19738855	0.25989138	0.259891	0.409823	1917	1704	1849	1823.333	2616	2330	2531	2492.333
cigR	510	306	364	1.855129	0.000107	1.645668	1.645668168	0.718673461	0.718673	0.001243	488	441	348	425.6667	764	858	747	789.6667
cirA	72	23	45	1.462329	0.043799	1.285872	1.285871853	0.362746874	0.362747	0.142418	99	111	82	97.33333	154	122	151	142.3333
citA	146	107	122.6667	1.406181	0.074739	1.235144	1.235143993	0.304679241	0.304679	0.207369	303	317	286	302	432	410	432	424.6667
citB	43	11	22.33333	1.817073	0.006116	1.603078	1.603078022	0.680844643	0.680845	0.031473	34	32	16	27.33333	59	47	43	49.66667
citC	3445	2087	2274	3.431219	0.000205	3.147967	3.147967097	1.654420462	1.65442	0.002079	1960	526	320	935.3333	3765	2613	3250	3209.333

Feature ID	Experiment - Range (original values)	Experiment - IQR (original values)	Experiment - Difference (original values)	Experiment - Fold Change (original values)	EDGE test: yccT NT vs WT NT , tagwise dispersions - P-value	EDGE test: yccT NT vs WT NT , tagwise dispersions - Fold change	yccT NT vs WT NT ABS|FC|	yccT NT vs WT NT Log2FC	yccT NT vs WT NT Log2FC +-	EDGE test: yccT NT vs WT NT , tagwise dispersions - FDR p-value correction	WT NT - WT.1.S22 - Expression values	WT NT - WT.2.S23 - Expression values	WT NT - WT.3.S24 - Expression values	WT NT - Means	yccT NT - yccT.1.S28 - Expression values	yccT NT - yccT.2.S29 - Expression values	yccT NT - yccT.3.S30 - Expression values	yccT NT - Means
citC2	196	86	123.3333	1.671506	0.001455	1.477843	1.477843154	0.563493162	0.563493	0.010232	225	183	143	183.6667	339	269	313	307
citD	794	378	533	3.193416	2.66E-05	2.891091	2.891090837	1.531613938	1.531614	0.000421	459	157	113	243	907	535	886	776
citD2	30	13	18.33333	1.916667	0.009234	1.679292	1.679292485	0.747853529	0.747854	0.043712	24	11	25	20	41	37	37	38.33333
citE	3080	1515	2022	3.057666	1.11E-05	2.754301	2.754300961	1.46168621	1.461686	0.000197	1728	677	543	982.6667	3623	2192	3199	3004.667
citE2	119	60	76.66667	1.804196	0.00122	1.592983	1.592983021	0.671730889	0.671731	0.00895	130	70	86	95.33333	181	146	189	172
citF	4402	1813	2823.667	3.169826	9.79E-06	2.846043	2.846043412	1.508957668	1.508958	0.000181	2217	952	735	1301.333	5137	2765	4473	4125
citF2	163	90	72.33333	1.35342	0.252121	1.200276	1.200276151	0.263366369	0.263366	0.463289	296	133	185	204.6667	293	263	275	277
citG	721	261	455.6667	3.055639	2.62E-06	2.728118	2.728117783	1.447905932	1.447906	6.01E-05	367	162	136	221.6667	857	423	752	677.3333
citG2	59	47	29	1.335907	0.251671	1.18685	1.186850032	0.247137651	0.247138	0.462913	124	70	65	86.33333	117	106	123	115.3333
citT	1402	569	935.6667	2.624421	7.26E-06	2.330257	2.330257302	1.220489263	1.220489	0.000142	875	425	428	576	1827	997	1711	1511.667
citX	798	324	493.3333	2.944809	4.44E-06	2.633254	2.633253799	1.396846578	1.396847	9.23E-05	427	179	155	253.6667	953	503	785	747
citX2	47	21	3.666667	1.057592	0.754983	-1.06485	1.06485324	0.090654609	-0.09065	0.880582	95	48	48	63.66667	69	50	83	67.33333
clpA	10882	1708	4856.333	1.143951	0.959151	1.008799	1.008799389	0.012639307	0.012639	1	35347	36649	29212	33736	40094	38628	37055	38592.33
clpB	7326	846	-3099.67	-1.31141	0.030544	-1.46904	1.469041547	0.554875198	-0.55488	0.108832	16397	13692	9071	13053.33	10141	10283	9437	9953.667
clpP	1385	546	-201.333	-1.03044	0.323627	-1.16826	1.16826075	0.224362313	-0.22436	0.538312	7487	6860	6102	6816.333	6866	6665	6314	6615
clpX	1236	668	-355.667	-1.03065	0.327256	-1.17455	1.174547819	0.23210545	-0.23211	0.541273	12341	12067	11476	11961.33	12313	11105	11399	11605.67
cls	359	250	217.3333	1.469065	0.04873	1.304517	1.304517331	0.383516111	0.383516	0.153246	616	402	372	463.3333	731	652	659	680.6667
cmk	328	140	220.3333	1.343198	0.188333	1.179715	1.179715482	0.238438959	0.238439	0.379147	722	587	617	642	915	757	915	862.3333
coaA	213	89	33.66667	1.033938	0.454398	-1.10932	1.109318603	0.149673776	-0.14967	0.663407	951	906	1119	992	1070	1040	967	1025.667
cobC	307	226	172.6667	1.279396	0.330036	1.136909	1.136909389	0.185117277	0.185117	0.544044	799	558	497	618	804	784	784	790.6667
cobD	139	101	18	1.023632	0.390525	-1.12069	1.120688325	0.164385106	-0.16439	0.60759	679	809	797	761.6667	818	708	813	779.6667
cobS	425	216	294.6667	1.962963	0.000607	1.708163	1.708162945	0.772445603	0.772446	0.00513	327	217	374	306	617	543	642	600.6667
cobT	542	360	442.6667	1.999248	1.49E-05	1.751354	1.751354151	0.808470848	0.808471	0.000257	476	389	464	443	902	824	931	885.6667
cobU	275	171	187.3333	1.878125	0.006812	1.619703	1.619702774	0.695729094	0.695729	0.03422	196	145	299	213.3333	415	367	420	400.6667
cof	380	257	248.3333	1.745	0.003383	1.563426	1.563426022	0.644710956	0.644711	0.019983	475	313	212	333.3333	592	570	583	581.6667
copA	964	343	-605	-1.16903	0.065642	-1.33033	1.330330329	0.41178452	-0.41178	0.190354	4241	4395	3917	4184.333	3733	3574	3431	3579.333
copR	249	163	155.6667	1.330035	0.19807	1.177267	1.177267428	0.23544208	0.235442	0.394449	582	433	400	471.6667	649	596	637	627.3333
copS	362	192	247.3333	1.589357	0.006162	1.409053	1.409052761	0.494725634	0.494726	0.031676	489	448	322	419.6667	677	640	684	667
corA	167	39	-55.6667	-1.09483	0.079587	-1.25542	1.255417691	0.328167445	-0.32817	0.217107	636	596	696	642.6667	635	529	597	587
corE	248	81	144.6667	1.303497	0.263754	1.146654	1.146654484	0.197430737	0.197431	0.475513	528	473	429	476.6667	677	554	633	621.3333
cpdB	8511	2280	-5246.33	-2.01745	1.93E-05	-2.32276	2.322763378	1.215842193	-1.21584	0.000318	7278	13330	10600	10402.67	5652	4998	4819	5156.333
cpsG	21	12	13.66667	1.672131	0.038608	1.472748	1.472748109	0.558510701	0.558511	0.129209	28	18	15	20.33333	36	30	36	34
cpxA	305	72	131.3333	1.083865	0.685514	-1.05605	1.056049992	0.078678131	-0.07868	0.832652	1558	1510	1630	1566	1815	1572	1705	1697.333
cpxP	1333	781	625.6667	1.272068	0.469251	1.124968	1.12496785	0.169883771	0.169884	0.676399	2969	1817	2113	2299.667	3150	2894	2732	2925.333
cpxR	300	84	35	1.022359	0.412918	-1.11892	1.118916074	0.162101829	-0.1621	0.625818	1623	1399	1674	1565.333	1699	1539	1563	1600.333
crcB	159	124	113	1.630112	0.005284	1.447058	1.447057726	0.533122475	0.533122	0.027976	243	152	143	179.3333	302	276	299	292.3333

Feature ID	Experiment - Range (original values)	Experiment - IQR (original values)	Experiment - Difference (original values)	Experiment - Fold Change (original values)	EDGE test: yccT NT vs WT NT , tagwise dispersions - P-value	EDGE test: yccT NT vs WT NT , tagwise dispersions - Fold change	yccT NT vs WT NT ABS|FC|	yccT NT vs WT NT Log2FC	yccT NT vs WT NT Log2FC +-	EDGE test: yccT NT vs WT NT , tagwise dispersions - FDR p-value correction	WT NT - WT.1.S22 - Expression values	WT NT - WT.2.S23 - Expression values	WT NT - WT.3.S24 - Expression values	WT NT - Means	yccT NT - yccT.1.S28 - Expression values	yccT NT - yccT.2.S29 - Expression values	yccT NT - yccT.3.S30 - Expression values	yccT NT - Means
creA	97	71	-74.3333	-1.1399	0.031611	-1.2997	1.299696455	0.378174721	-0.37817	0.11161	613	607	597	605.6667	552	516	526	531.3333
creB	97	30	22	1.036851	0.442058	-1.09738	1.097382255	0.134066152	-0.13407	0.65292	616	623	552	597	649	589	619	619
creC	63	20	-8	-1.02771	0.183635	-1.1714	1.171398278	0.228231678	-0.22823	0.372909	292	326	272	296.6667	318	263	285	288.6667
creD	109	63	49.33333	1.217647	0.531977	1.076606	1.076605741	0.106490023	0.10649	0.724397	278	215	187	226.6667	296	253	279	276
crl	293	90	-165.667	-1.10543	0.086397	-1.25537	1.255367668	0.328109958	-0.32811	0.228443	1793	1792	1626	1737	1562	1652	1500	1571.333
crp	3465	1373	-1799.33	-1.09978	0.181048	-1.25708	1.257084403	0.330081519	-0.33008	0.369874	19549	19446	20501	19832	18989	18073	17036	18032.67
crr	2535	1751	-1615	-1.13518	0.120155	-1.29616	1.296157212	0.374240715	-0.37424	0.282894	13443	13974	13270	13562.33	12884	11439	11519	11947.33
csdA	605	296	362.6667	1.34376	0.171673	1.184631	1.18463097	0.244437708	0.244438	0.3578	1187	1050	928	1055	1533	1346	1374	1417.667
csgA	33	14	19	1.721519	0.014118	1.515912	1.515912147	0.600186146	0.600186	0.060359	33	24	22	26.33333	43	38	55	45.33333
csgB	5	3	3	2	0.262419	1.733458	1.733457857	0.793652763	0.793653	0.474024	5	2	2	3	7	5	6	6
csgC	20	14	15.33333	3.421053	4.94E-05	2.971709	2.971709409	1.571293048	1.571293	0.000687	7	3	9	6.333333	23	21	21	21.66667
csgD	41	19	26.66667	2.777778	6.39E-06	2.428947	2.428947119	1.280331081	1.280331	0.000127	17	13	15	15	54	37	34	41.66667
csgE	19	5	7.666667	2.352941	0.024347	2.061669	2.06166912	1.043812812	1.043813	0.091093	10	5	2	5.666667	10	9	21	13.33333
csgF	17	6	9.333333	3.545455	0.001146	3.059686	3.059685654	1.613383441	1.613383	0.008501	7	1	3	3.666667	12	9	18	13
csgG	111	10	-16	-1.15635	0.154907	-1.30246	1.302457394	0.38123618	-0.38124	0.33296	112	177	66	118.3333	106	96	105	102.3333
csiE	1031	272	207	1.136244	0.923979	1.014628	1.014627511	0.020950184	0.02095	0.984632	2100	1389	1069	1519.333	1858	1661	1660	1726.333
cspA	10704	3700	316.6667	1.012305	0.516902	-1.12591	1.125910694	0.1710924	-0.17109	0.715467	22166	32870	22168	25734.67	27380	24906	25868	26051.33
cspB	1634	1101	1087	2.046198	0.000711	1.844022	1.844022347	0.882856139	0.882856	0.005868	1573	964	580	1039	2214	2065	2099	2126
cspC	8136	2659	-3762.33	-1.16636	0.106218	-1.34296	1.342958297	0.425414506	-0.42541	0.261184	21809	28830	28494	26377.67	24468	20694	22684	22615.33
cspD	5543	2928	3348.667	1.483702	0.132691	1.307238	1.307237804	0.38652161	0.386522	0.30059	8636	5150	6983	6923	10693	10211	9911	10271.67
cspE	15309	11254	10774.33	1.385693	0.243997	1.221416	1.221415504	0.288554063	0.288554	0.454647	32677	24888	26240	27935	40197	37494	38437	38709.33
csrA	608	65	-50.6667	-1.01673	0.34301	-1.15651	1.156508629	0.20977603	-0.20978	0.557344	3429	2821	2987	3079	3205	2958	2922	3028.333
cstA	1383	525	598.3333	1.222594	0.619725	1.078728	1.078727727	0.109330772	0.109331	0.786369	3152	2627	2285	2688	3668	2945	3246	3286.333
cueO	384	155	-148.667	-1.09962	0.087336	-1.25053	1.250525645	0.322534643	-0.32253	0.229842	1773	1618	1532	1641	1625	1463	1389	1492.333
cueR	346	220	144	1.135296	0.993732	1.001294	1.001294253	0.001866006	0.001866	1	1260	928	1005	1064.333	1274	1225	1126	1208.333
cutA	253	133	145.6667	1.276933	0.324423	1.129798	1.12979806	0.176064928	0.176065	0.539103	629	504	445	526	680	637	698	671.6667
cutC	704	209	-500.333	-1.86215	1.43E-06	-2.14287	2.142872663	1.099546123	-1.09955	3.59E-05	788	1282	1172	1080.667	584	579	578	580.3333
cvpA	274	44	139.6667	1.229589	0.52945	1.079968	1.079967955	0.110988505	0.110989	0.722956	641	631	553	608.3333	827	675	742	748
cyaA	1608	365	102	1.011771	0.466342	-1.12624	1.126236306	0.171509565	-0.17151	0.6744	8622	9438	7935	8665	9543	8257	8501	8767
cyaY	454	304	-333.667	-1.48079	3.47E-05	-1.69006	1.690055705	0.757070799	-0.75707	0.000519	982	1112	989	1027.667	746	678	658	694
cybB	151	52	76	1.225966	0.507065	1.081113	1.081113338	0.112517775	0.112518	0.70721	373	345	291	336.3333	442	397	398	412.3333
cybC	634	160	-261.667	-1.21952	0.022864	-1.37599	1.375990816	0.460470841	-0.46047	0.086588	1553	1721	1087	1453.667	1256	1224	1096	1192
cycA	586	327	386	1.561047	0.010302	1.384696	1.384696056	0.469569336	0.469569	0.047207	819	703	542	688	1064	1030	1128	1074
cydA	31108	6119	15742	1.233202	0.6651	1.078922	1.078922244	0.109590896	0.109591	0.819576	73028	59157	70326	67503.67	90265	76445	83027	83245.67
cydB	19325	5716	6268.333	1.126599	0.954333	-1.01009	1.010090602	0.014484704	-0.01448	1	58086	41911	48543	49513.33	61236	51850	54259	55781.67
cydC	212	64	96.66667	1.140845	0.991417	1.001868	1.001867569	0.002691819	0.002692	1	741	677	641	686.3333	853	702	794	783

Feature ID	Experiment - Range (original values)	Experiment - IQR (original values)	Experiment - Difference (original values)	Experiment - Fold Change (original values)	EDGE test: yccT NT vs WT NT , tagwise dispersions - P-value	EDGE test: yccT NT vs WT NT , tagwise dispersions - Fold change	yccT NT vs WT NT ABS|FC|	yccT NT vs WT NT Log2FC	yccT NT vs WT NT Log2FC +-	EDGE test: yccT NT vs WT NT , tagwise dispersions - FDR p-value correction	WT NT - WT.1.S22 - Expression values	WT NT - WT.2.S23 - Expression values	WT NT - WT.3.S24 - Expression values	WT NT - Means	yccT NT - yccT.1.S28 - Expression values	yccT NT - yccT.2.S29 - Expression values	yccT NT - yccT.3.S30 - Expression values	yccT NT - Means
cydD	435	169	298.3333	1.347573	0.179078	1.182459	1.182458641	0.241789723	0.24179	0.367019	901	866	808	858.3333	1243	1035	1192	1156.667
cyoA	1365	95	-521	-2.15181	0.000364	-2.44382	2.443818925	1.289137392	-1.28914	0.003329	528	1771	621	973.3333	406	433	518	452.3333
cyoB	3932	297	-1364	-1.99805	0.002297	-2.26301	2.263013444	1.178245155	-1.17825	0.01446	1481	5037	1674	2730.667	1105	1349	1646	1366.667
cyoC	1489	134	-570	-2.49215	0.000131	-2.81365	2.813650949	1.492443364	-1.49244	0.001463	544	1808	504	952	319	370	457	382
cyoD	1154	128	-476	-2.81449	1.4E-05	-3.17589	3.175885762	1.667159019	-1.66716	0.000244	459	1390	366	738.3333	236	238	313	262.3333
cyoE	3830	492	-1595.33	-3.03227	6.45E-06	-3.42437	3.424370974	1.775839002	-1.77584	0.000127	1377	4512	1252	2380.333	682	760	913	785
cypD	1082	415	666.6667	1.176929	0.838885	1.032164	1.032164496	0.045672911	0.045673	0.937824	3952	3699	3653	3768	4735	4114	4455	4434.667
cysA	52	4	17.66667	1.201521	0.659931	1.059774	1.059773636	0.083756143	0.083756	0.816798	96	100	67	87.66667	119	97	100	105.3333
cysB	1205	839	625	1.303791	0.352238	1.156373	1.156373113	0.20960697	0.209607	0.567346	2680	1798	1694	2057.333	2899	2511	2637	2682.333
cysC	30	2	7	1.077206	0.700319	-1.05408	1.054077671	0.075981178	-0.07598	0.84255	99	87	86	90.66667	88	89	116	97.66667
cysD	15	11	7.666667	1.20354	0.709198	1.0615	1.061500166	0.086104596	0.086105	0.847948	47	33	33	37.66667	44	44	48	45.33333
cysE	475	138	-86.3333	-1.02755	0.302714	-1.17828	1.178277582	0.236679454	-0.23668	0.518087	3033	3171	3457	3220.333	3286	2982	3134	3134
cysG	395	118	240.3333	1.154192	0.933212	1.011503	1.011503392	0.016501158	0.016501	0.989486	1586	1524	1566	1558.667	1794	1684	1919	1799
cysH	38	6	-12.3333	-1.11145	0.083048	-1.27341	1.273412802	0.348700173	-0.3487	0.222167	115	109	145	123	110	107	115	110.6667
cysI	142	30	-78.3333	-1.26644	0.003707	-1.44921	1.449212096	0.535268753	-0.53527	0.021344	327	418	372	372.3333	309	276	297	294
cysJ	144	39	-70.6667	-1.28726	0.004659	-1.47388	1.473881903	0.559620931	-0.55962	0.025424	270	361	319	316.6667	241	217	280	246
cysK	403	281	-290	-1.14995	0.051805	-1.31078	1.310779281	0.390424774	-0.39042	0.159963	2289	2201	2182	2224	2015	1886	1901	1934
cysM	345	240	250.6667	1.915956	7.6E-05	1.702765	1.7027651	0.767879426	0.767879	0.000952	371	241	209	273.6667	554	481	538	524.3333
cysN	81	29	26.33333	1.087973	0.7356	-1.04109	1.041092614	0.058098415	-0.0581	0.866182	344	291	263	299.3333	337	320	320	325.6667
cysP	177	39	81.66667	1.379257	0.146272	1.232831	1.232831301	0.301975396	0.301975	0.320382	273	238	135	215.3333	277	302	312	297
cysQ	1132	40	112	1.040901	0.611704	-1.08203	1.082029168	0.11373939	-0.11374	0.781788	3262	2823	2130	2738.333	2885	2813	2853	2850.333
cysS	1621	646	808.3333	1.211292	0.664271	1.069981	1.069981179	0.09758542	0.097585	0.819355	4425	3779	3273	3825.667	4894	4370	4638	4634
cysU	42	24	13.33333	1.213904	0.638091	1.077609	1.077608952	0.107833741	0.107834	0.800052	87	55	45	62.33333	85	79	63	75.66667
cysW	43	12	22	1.392857	0.140828	1.232491	1.23249101	0.301577123	0.301577	0.312787	68	58	42	56	79	85	70	78
cysZ	204	142	144	1.458599	0.033045	1.28822	1.288219608	0.365378556	0.365379	0.115112	377	290	275	314	479	432	463	458
cytR	2088	370	-899.333	-1.38253	0.009741	-1.57384	1.573837379	0.654286478	-0.65429	0.045499	2730	4362	2659	3250.333	2490	2274	2289	2351
dacA	295	37	164.6667	1.135417	0.965521	-1.0058	1.00579761	0.00834003	-0.00834	1	1238	1216	1194	1216	1489	1253	1400	1380.667
dacB	172	63	39.66667	1.046214	0.502851	-1.08487	1.08487373	0.117527135	-0.11753	0.704228	919	899	757	858.3333	929	851	914	898
dacC	725	447	479.6667	1.398836	0.108138	1.236353	1.23635318	0.306090927	0.306091	0.264438	1455	1112	1041	1202.667	1766	1559	1722	1682.333
dacD	468	294	312	1.470825	0.035172	1.300483	1.300483343	0.379047921	0.379048	0.120538	779	649	560	662.6667	1028	943	953	974.6667
dadA	380	159	256.3333	2.273179	1.34E-06	2.017564	2.017563841	1.012614325	1.012614	3.44E-05	211	239	154	201.3333	370	469	534	457.6667
dadX	249	149	149.6667	1.580854	0.026954	1.390735	1.390735246	0.4758478	0.475848	0.098625	217	339	217	257.6667	366	390	466	407.3333
dam	264	208	-69	-1.08011	0.129682	-1.24394	1.243937653	0.314914179	-0.31491	0.296753	767	1031	993	930.3333	992	784	808	861.3333
damX	1529	260	-801.667	-1.25031	0.031401	-1.43946	1.439458864	0.525526561	-0.52553	0.111019	3333	4397	4283	4004.333	3500	2868	3240	3202.667
dapA	1150	347	170.6667	1.022049	0.50291	-1.11234	1.112335487	0.153591979	-0.15359	0.704228	8320	7731	7170	7740.333	8284	7551	7898	7911
dapB	329	218	184.6667	1.244916	0.446596	1.099602	1.099602253	0.136981768	0.136982	0.656559	910	692	660	754	989	865	962	938.6667

Feature ID	Experiment - Range (original values)	Experiment - IQR (original values)	Experiment - Difference (original values)	Experiment - Fold Change (original values)	EDGE test: yccT NT vs WT NT , tagwise dispersions - P-value	EDGE test: yccT NT vs WT NT , tagwise dispersions - Fold change	yccT NT vs WT NT ABS|FC|	yccT NT vs WT NT Log2FC	yccT NT vs WT NT Log2FC +-	EDGE test: yccT NT vs WT NT , tagwise dispersions - FDR p-value correction	WT NT - WT.1.S22 - Expression values	WT NT - WT.2.S23 - Expression values	WT NT - WT.3.S24 - Expression values	WT NT - Means	yccT NT - yccT.1.S28 - Expression values	yccT NT - yccT.2.S29 - Expression values	yccT NT - yccT.3.S30 - Expression values	yccT NT - Means
dapD	1508	645	798	1.181199	0.793871	1.041419	1.041419114	0.058550791	0.058551	0.903705	4960	4315	3937	4404	5445	4938	5223	5202
dapE	393	233	159.6667	1.088327	0.734989	-1.04789	1.047889527	0.06748663	-0.06749	0.865693	1752	1985	1686	1807.667	2079	1833	1990	1967.333
dapF	746	286	-317.333	-1.07485	0.191755	-1.22331	1.223312472	0.29079296	-0.29079	0.384465	4759	4682	4230	4557	4496	4013	4210	4239.667
dbpA	79	8	43.66667	1.247637	0.459364	1.09337	1.093369965	0.12878165	0.128782	0.668015	183	177	169	176.3333	248	185	227	220
dcd	362	180	224.3333	1.189898	0.716658	1.046264	1.046263641	0.065246433	0.065246	0.852028	1277	1157	1110	1181.333	1472	1337	1408	1405.667
dcm	590	380	355.6667	1.289709	0.327685	1.137096	1.137096037	0.185354107	0.185354	0.541781	1443	1123	1117	1227.667	1707	1503	1540	1583.333
dcoC	9	1	4.666667	2.75	0.051403	2.361632	2.361632385	1.23978441	1.239784	0.159076	3	4	1	2.666667	8	4	10	7.333333
dcp	451	84	-62.6667	-1.03317	0.241396	-1.17193	1.171925899	0.22888135	-0.22888	0.451668	2205	1896	1754	1951.667	1993	1812	1862	1889
dctA	4176	775	-1688.67	-1.25457	0.059123	-1.45172	1.451717319	0.537760557	-0.53776	0.176176	5636	9812	9518	8322	6902	6127	6871	6633.333
dcuA	4396	802	-1779.33	-1.10318	0.175628	-1.25722	1.257222308	0.330239777	-0.33024	0.363649	20756	17339	18979	19024.67	18089	16360	17287	17245.33
dcuB	9951	4130	183.3333	1.009	0.524958	-1.12893	1.1289328	0.174959612	-0.17496	0.719276	24612	14661	21837	20370	22686	21267	17707	20553.33
dcuC	589	176	339	1.236237	0.585394	1.078765	1.078764927	0.109380522	0.109381	0.764059	1456	1289	1560	1435	1878	1632	1812	1774
dcuR	168	86	3.333333	1.003951	0.324832	-1.12982	1.129819682	0.176092538	-0.17609	0.539103	953	785	793	843.6667	875	877	789	847
dcuS	277	179	185.3333	1.18614	0.719668	1.045185	1.045184651	0.063757843	0.063758	0.854232	1082	991	914	995.6667	1191	1170	1182	1181
ddg	651	285	452	1.406841	0.142021	1.226211	1.226211121	0.294207394	0.294207	0.314175	1106	1006	1221	1111	1641	1391	1657	1563
ddlA	206	47	53.66667	1.0322	0.448896	-1.10586	1.105855944	0.145163462	-0.14516	0.658847	1697	1653	1650	1666.667	1810	1604	1747	1720.333
ddlB	1093	223	54.66667	1.022207	0.522149	-1.1028	1.10279812	0.141168713	-0.14117	0.718141	3095	2288	2002	2461.667	2636	2402	2511	2516.333
deaD	3682	1323	2147.667	1.538712	0.057369	1.359989	1.359988829	0.443594801	0.443595	0.172341	4781	3974	3205	3986.667	6887	5297	6219	6134.333
dedA	84	45	48.33333	1.278311	0.328157	1.128321	1.128320595	0.174177046	0.174177	0.542157	174	205	142	173.6667	219	226	221	222
dedD	302	191	154	1.131661	0.968601	-1.00512	1.005123024	0.007372094	-0.00737	1	1298	1107	1104	1169.667	1406	1224	1341	1323.667
def	736	510	451	1.322681	0.242211	1.171386	1.171385987	0.228216541	0.228217	0.452335	1740	1263	1190	1397.667	1926	1773	1847	1848.667
degQ	1087	466	-769	-1.16705	0.07574	-1.33457	1.334568346	0.416373191	-0.41637	0.209361	5084	5481	5552	5372.333	4727	4618	4465	4603.333
degS	196	106	66	1.059034	0.563296	-1.07665	1.076646502	0.106544645	-0.10654	0.745911	1084	1207	1063	1118	1190	1103	1259	1184
deoA	6572	421	-2735.33	-2.49472	9.23E-05	-2.81533	2.8153338	1.493305986	-1.49331	0.001114	3267	8279	2150	4565.333	2054	1707	1729	1830
deoB	10232	2154	-4748.67	-1.91379	0.000232	-2.17604	2.176040348	1.121705307	-1.12171	0.002273	7437	15227	7172	9945.333	5577	4995	5018	5196.667
deoC	4127	460	-1837.67	-2.61529	6.45E-06	-2.9526	2.952599852	1.56198585	-1.56199	0.000127	2136	5187	1603	2975.333	1210	1143	1060	1137.667
deoD	5406	1414	-2683.33	-2.01693	4.04E-05	-2.29786	2.297859487	1.200290581	-1.20029	0.000587	3953	7927	4086	5322	2856	2539	2521	2638.667
deoR	388	274	257	1.399896	0.086023	1.24159	1.241590156	0.312189024	0.312189	0.228294	786	614	528	642.6667	895	888	916	899.6667
dfp	313	32	-114.333	-1.10426	0.092283	-1.2666	1.266602286	0.340963588	-0.34096	0.23829	1021	1334	1278	1211	1109	1077	1104	1096.667
dgkA	306	91	32	1.033391	0.487874	-1.09297	1.092974398	0.128259607	-0.12826	0.692606	1134	913	828	958.3333	1004	1026	941	990.3333
dgoA	105	21	-44.3333	-1.31517	0.002094	-1.50126	1.501262261	0.586176029	-0.58618	0.013516	166	224	165	185	159	119	144	140.6667
dgoK	115	7	-49	-1.25744	0.008552	-1.43423	1.434229697	0.520276096	-0.52028	0.0411	203	287	228	239.3333	196	203	172	190.3333
dgoR	222	111	-158.333	-1.64363	1.25E-06	-1.87914	1.87914039	0.910072854	-0.91007	3.28E-05	358	451	404	404.3333	262	247	229	246
dgoT	88	12	-43.3333	-1.30733	0.004937	-1.50145	1.501449404	0.586355859	-0.58636	0.026386	145	211	197	184.3333	144	123	156	141
dgt	267	85	68.33333	1.04212	0.524104	-1.08785	1.087849858	0.121479453	-0.12148	0.719276	1779	1576	1512	1622.333	1650	1761	1661	1690.667
dinF	269	17	35	1.042135	0.504026	-1.08527	1.085268197	0.118051613	-0.11805	0.704746	956	849	687	830.6667	919	832	846	865.6667

Feature ID	Experiment - Range (original values)	Experiment - IQR (original values)	Experiment - Difference (original values)	Experiment - Fold Change (original values)	EDGE test: yccT NT vs WT NT , tagwise dispersions - P-value	EDGE test: yccT NT vs WT NT , tagwise dispersions - Fold change	yccT NT vs WT NT ABS|FC|	yccT NT vs WT NT Log2FC	yccT NT vs WT NT Log2FC +-	EDGE test: yccT NT vs WT NT , tagwise dispersions - FDR p-value correction	WT NT - WT.1.S22 - Expression values	WT NT - WT.2.S23 - Expression values	WT NT - WT.3.S24 - Expression values	WT NT - Means	yccT NT - yccT.1.S28 - Expression values	yccT NT - yccT.2.S29 - Expression values	yccT NT - yccT.3.S30 - Expression values	yccT NT - Means
dinG	406	132	-170	-1.09661	0.106855	-1.24375	1.243747051	0.314693105	-0.31469	0.262201	2051	2072	1666	1929.667	1830	1698	1751	1759.667
dinI	92	49	60	1.285714	0.327227	1.129921	1.129921355	0.176222361	0.176222	0.541273	231	182	217	210	266	274	270	270
dinP	342	138	125.6667	1.201928	0.633252	1.06439	1.06438966	0.090026401	0.090026	0.796785	788	578	501	622.3333	843	685	716	748
djlA	290	75	81.33333	1.076802	0.673598	-1.05379	1.053785247	0.075580887	-0.07558	0.824506	1195	1031	951	1059	1241	1106	1074	1140.333
dksA	3479	2051	-2631.33	-1.45363	0.002142	-1.65982	1.659822852	0.731029275	-0.73103	0.013758	7910	9040	8346	8432	5982	5859	5561	5800.667
dld	1310	168	112.3333	1.03698	0.600857	-1.08606	1.08605835	0.119101616	-0.1191	0.77508	3616	3191	2306	3037.667	3318	3109	3023	3150
dlhH	321	45	68.33333	1.073662	0.660166	-1.06025	1.060250329	0.084404931	-0.0844	0.816798	1088	775	920	927.6667	1096	927	965	996
dmsA	5550	4683	3252.333	1.078751	0.757574	-1.05406	1.054057819	0.075954007	-0.07595	0.880907	44495	39812	39590	41299	45140	43742	44772	44551.33
dmsA1	4315	419	-239	-1.02698	0.38846	-1.15625	1.15624776	0.209450571	-0.20945	0.605226	11398	8815	7083	9098.667	9517	8666	8396	8859.667
dmsA2	614	108	-61.6667	-1.0387	0.240983	-1.18172	1.181717554	0.240885254	-0.24089	0.451455	1513	2033	1419	1655	1603	1495	1682	1593.333
dmsA3	2003	1111	-1378	-1.28338	0.018494	-1.47101	1.471013641	0.556810625	-0.55681	0.073694	5933	6195	6594	6240.667	5175	4591	4822	4862.667
dmsB	1051	253	573	1.160985	0.914336	1.017126	1.017125786	0.024498106	0.024498	0.980499	3780	3264	3634	3559.333	4315	3887	4195	4132.333
dmsB1	443	108	-200	-1.39113	0.0018	-1.61115	1.6111529	0.688093413	-0.68809	0.012139	621	642	871	711.3333	593	428	513	511.3333
dmsB2	83	27	-17	-1.12409	0.097866	-1.28732	1.287320495	0.364371275	-0.36437	0.24695	118	199	145	154	143	116	152	137
dmsC	1040	298	377.3333	1.098366	0.788994	-1.04443	1.044431005	0.062717191	-0.06272	0.900807	3774	3570	4164	3836	4610	3958	4072	4213.333
dmsC1	500	68	-197	-1.20047	0.030381	-1.38273	1.382734344	0.467524007	-0.46752	0.108477	1140	959	1440	1179.667	1027	940	981	982.6667
dmsC2	50	8	-26.6667	-1.2649	0.005384	-1.44811	1.448105902	0.534167113	-0.53417	0.028401	112	137	133	127.3333	111	87	104	100.6667
dnaA	494	17	175.3333	1.041693	0.562472	-1.09704	1.097040236	0.133616441	-0.13362	0.745608	4068	4280	4268	4205.333	4562	4317	4263	4380.667
dnaB	111	21	-7.66667	-1.00686	0.270131	-1.14976	1.149761437	0.201334548	-0.20133	0.483616	1093	1177	1108	1126	1175	1066	1114	1118.333
dnaC	278	69	140.6667	1.072558	0.659458	-1.06287	1.062867453	0.087961693	-0.08796	0.816445	1945	1995	1876	1938.667	2154	2014	2070	2079.333
dnaE	551	257	-71	-1.03114	0.271387	-1.18497	1.184968262	0.244848418	-0.24485	0.484104	2157	2414	2482	2351	2546	1995	2299	2280
dnaG	437	205	-71	-1.03756	0.23995	-1.19239	1.192387417	0.253853056	-0.25385	0.450517	1672	2103	2109	1961.333	1989	1784	1898	1890.333
dnaJ	1023	544	-834.667	-2.25075	4.48E-13	-2.54307	2.543070878	1.346571672	-1.34657	4.51E-11	1665	1629	1212	1502	692	668	642	667.3333
dnaK	10837	5695	-7902.33	-3.35445	6.44E-13	-3.8304	3.830396862	1.937493876	-1.93749	6.2E-11	9033	14080	10663	11258.67	3243	3488	3338	3356.333
dnaN	304	160	-15.3333	-1.0077	0.317812	-1.15657	1.156573304	0.209856708	-0.20986	0.533014	1847	2029	2143	2006.333	2151	1869	1953	1991
dnaQ	495	310	276.3333	1.359809	0.155368	1.209905	1.20990497	0.274893737	0.274894	0.33296	1006	706	592	768	1087	1030	1016	1044.333
dnaT	348	67	147.6667	1.137792	0.975602	1.004036	1.004036178	0.005811254	0.005811	1	1178	1111	926	1071.667	1233	1151	1274	1219.333
dnaX	621	245	218.3333	1.079432	0.718123	-1.05656	1.056559588	0.079374135	-0.07937	0.853045	2742	2940	2564	2748.667	3185	2695	3021	2967
dniR	1158	697	599.3333	1.240825	0.561715	1.095215	1.09521521	0.131214388	0.131214	0.745379	3039	2124	2303	2488.667	3282	3000	2982	3088
dpiA	1011	708	599	1.861457	0.005206	1.669108	1.669107693	0.739077043	0.739077	0.027592	1122	488	476	695.3333	1487	1200	1196	1294.333
dpiB	2657	1827	1564	2.054619	0.007814	1.86088	1.860879875	0.895984928	0.895985	0.038259	2672	946	831	1483	3488	2880	2773	3047
dppA	22256	15717	10865	1.444352	0.204638	1.298484	1.298483839	0.376828058	0.376828	0.403179	37514	20582	15258	24451.33	37024	32626	36299	35316.33
dppB	3164	1619	1773.333	1.743536	0.02775	1.57208	1.572080329	0.652674937	0.652675	0.101036	3736	2117	1302	2385	4347	3662	4466	4158.333
dppC	1605	627	926.3333	1.651735	0.017666	1.473642	1.473642202	0.559386283	0.559386	0.070967	1970	1343	951	1421.333	2540	1947	2556	2347.667
dppD	2043	793	1298	1.661121	0.014561	1.468774	1.46877378	0.55461221	0.554612	0.061919	2424	1892	1574	1963.333	3617	2685	3482	3261.333
dppF	1684	589	1022	1.712692	0.006259	1.506243	1.506242759	0.590954306	0.590954	0.032056	1750	1297	1255	1434	2939	1886	2543	2456

Feature ID	Experiment - Range (original values)	Experiment - IQR (original values)	Experiment - Difference (original values)	Experiment - Fold Change (original values)	EDGE test: yccT NT vs WT NT , tagwise dispersions - P-value	EDGE test: yccT NT vs WT NT , tagwise dispersions - Fold change	yccT NT vs WT NT ABS|FC|	yccT NT vs WT NT Log2FC	yccT NT vs WT NT Log2FC +-	EDGE test: yccT NT vs WT NT , tagwise dispersions - FDR p-value correction	WT NT - WT.1.S22 - Expression values	WT NT - WT.2.S23 - Expression values	WT NT - WT.3.S24 - Expression values	WT NT - Means	yccT NT - yccT.1.S28 - Expression values	yccT NT - yccT.2.S29 - Expression values	yccT NT - yccT.3.S30 - Expression values	yccT NT - Means
dps	895	325	38	1.013009	0.480531	-1.11713	1.117132946	0.159800886	-0.1598	0.686186	3475	2580	2708	2921	3033	3114	2730	2959
dsbA	439	253	-275.333	-1.13764	0.074083	-1.30161	1.301605542	0.380292298	-0.38029	0.206064	2222	2258	2347	2275.667	2124	1908	1969	2000.333
dsbB	303	117	146.3333	1.168393	0.845623	1.025116	1.025115915	0.035787052	0.035787	0.94192	955	814	838	869	1117	907	1022	1015.333
dsbC	283	91	125.6667	1.084227	0.705747	-1.04953	1.049527141	0.069739476	-0.06974	0.845636	1581	1490	1405	1492	1688	1533	1632	1617.667
dsbD	452	202	239	1.188784	0.720015	1.046985	1.046984672	0.066240321	0.06624	0.854416	1424	1222	1152	1266	1604	1417	1494	1505
dsbG	311	147	110	1.109927	0.869068	-1.02154	1.021541486	0.030747794	-0.03075	0.956255	1184	915	903	1000.667	1214	1062	1056	1110.667
dsdA	435	22	-53	-1.08038	0.177913	-1.22281	1.222807046	0.29019677	-0.2902	0.365816	662	955	520	712.3333	678	640	660	659.3333
dsdC	56	26	-19.3333	-1.12033	0.060923	-1.28306	1.283059788	0.359588399	-0.35959	0.179847	156	197	187	180	182	159	141	160.6667
dsdX	135	9	-26	-1.21311	0.091166	-1.37318	1.373177085	0.457517687	-0.45752	0.236648	119	230	95	148	120	111	135	122
dut	307	97	-144.667	-1.13088	0.059607	-1.29967	1.299672441	0.378148064	-0.37815	0.17726	1066	1332	1352	1250	1163	1045	1108	1105.333
dxr	619	187	181.6667	1.125605	0.99141	-1.00175	1.001751968	0.002525344	-0.00253	1	1763	1432	1144	1446.333	1713	1552	1619	1628
dxs	627	273	337.3333	1.101271	0.82216	-1.0353	1.035301637	0.050051161	-0.05005	0.924153	3278	3551	3164	3331	3791	3472	3742	3668.333
ecnB	119	39	60.33333	1.660584	0.011549	1.47816	1.47815999	0.56380243	0.563802	0.051586	128	92	54	91.33333	173	131	151	151.6667
ecnR	99	32	55.33333	1.276206	0.324688	1.129847	1.129847437	0.176127979	0.176128	0.539103	228	196	177	200.3333	225	276	266	255.6667
eco	1818	187	-969.333	-1.21912	0.042159	-1.38351	1.383513097	0.468336302	-0.46834	0.138271	5546	5819	4814	5393	4627	4643	4001	4423.667
eda	668	380	-482	-1.42743	0.000303	-1.63517	1.635167436	0.709438371	-0.70944	0.002818	1579	1535	1715	1609.667	1181	1047	1155	1127.667
edd	503	247	-371	-1.5129	6.17E-05	-1.73619	1.736192777	0.795927146	-0.79593	0.000806	969	1123	1191	1094.333	760	722	688	723.3333
efp	995	200	-500.333	-1.20531	0.051369	-1.38924	1.389242523	0.474298476	-0.4743	0.159076	2391	3111	3310	2937.333	2591	2315	2405	2437
ego	11525	8402	8052.333	4.368238	0.006492	4.103561	4.103561096	2.036876433	2.036876	0.032989	6409	412	351	2390.667	11876	8814	10639	10443
elaA	57	8	-24.3333	-1.09825	0.0557	-1.24786	1.247862135	0.319458553	-0.31946	0.168238	272	299	245	272	253	248	242	247.6667
elaB	85	14	-9.66667	-1.03558	0.180739	-1.17721	1.177211001	0.23537293	-0.23537	0.369739	327	242	275	281.3333	292	261	262	271.6667
elaC	221	109	93.33333	1.208333	0.582598	1.073118	1.073117994	0.101808716	0.101809	0.761737	571	423	350	448	566	526	532	541.3333
emrA	420	142	252	1.173394	0.829657	1.028776	1.028775816	0.040928633	0.040929	0.930686	1520	1428	1412	1453.333	1832	1570	1714	1705.333
emrB	204	40	13.66667	1.025593	0.402028	-1.12162	1.121618074	0.165581503	-0.16558	0.616823	511	519	572	534	648	444	551	547.6667
emrD	389	253	304.3333	1.846154	8.97E-05	1.626572	1.626571549	0.701834285	0.701834	0.001092	426	327	326	359.6667	697	580	715	664
emrR	491	381	392	1.227378	0.576393	1.07895	1.07894952	0.109627368	0.109627	0.755173	1823	1696	1653	1724	2144	2077	2127	2116
emtA	114	25	57.66667	1.220382	0.529904	1.076494	1.076493928	0.106340182	0.10634	0.723325	290	270	225	261.6667	324	295	339	319.3333
endA	34	12	1.666667	1.018182	0.449454	-1.11406	1.114063314	0.155831226	-0.15583	0.65923	85	112	78	91.66667	82	94	104	93.33333
engA	484	99	257.6667	1.282736	0.391618	1.120046	1.120046044	0.163558042	0.163558	0.60865	947	830	957	911.3333	1314	1046	1147	1169
eno	22145	3814	-12516.7	-2.07713	7.57E-05	-2.42641	2.426409084	1.278822804	-1.27882	0.000952	15419	24047	32945	24137	12456	10800	11605	11620.33
entA	23	12	12.33333	1.425287	0.182701	1.254993	1.25499339	0.327679766	0.32768	0.371904	37	25	25	29	43	33	48	41.33333
entB	15	4	1.666667	1.039683	0.635017	-1.09035	1.0903528	0.124795016	-0.1248	0.797616	48	45	33	42	47	43	41	43.66667
entC	25	7	-1	-1.02727	0.428894	-1.15882	1.158823846	0.212661277	-0.21266	0.640136	48	42	23	37.66667	35	34	41	36.66667
entD	17	4	6.333333	1.17757	0.866243	1.036673	1.036672629	0.051960377	0.05196	0.954335	37	41	29	35.66667	46	37	43	42
entE	19	4	8.666667	1.252427	0.534691	1.103306	1.103305696	0.141832578	0.141833	0.726308	39	35	29	34.33333	48	44	37	43
entF	146	53	83	1.496016	0.023941	1.322822	1.322822138	0.403619095	0.403619	0.089748	206	166	130	167.3333	276	219	256	250.3333

Feature ID	Experiment - Range (original values)	Experiment - IQR (original values)	Experiment - Difference (original values)	Experiment - Fold Change (original values)	EDGE test: yccT NT vs WT NT , tagwise dispersions - P-value	EDGE test: yccT NT vs WT NT , tagwise dispersions - Fold change	yccT NT vs WT NT ABS|FC|	yccT NT vs WT NT Log2FC	yccT NT vs WT NT Log2FC +-	EDGE test: yccT NT vs WT NT , tagwise dispersions - FDR p-value correction	WT NT - WT.1.S22 - Expression values	WT NT - WT.2.S23 - Expression values	WT NT - WT.3.S24 - Expression values	WT NT - Means	yccT NT - yccT.1.S28 - Expression values	yccT NT - yccT.2.S29 - Expression values	yccT NT - yccT.3.S30 - Expression values	yccT NT - Means
envE	216	170	174.3333	2.644654	2.96E-10	2.354435	2.354435499	1.2353812	1.235381	1.99E-08	145	99	74	106	282	290	269	280.3333
envF	16	12	13.33333	3.666667	4.19E-05	3.178693	3.178693052	1.66843371	1.668434	0.000599	6	4	5	5	17	18	20	18.33333
envR	16	5	5.666667	1.239437	0.674897	1.092565	1.092565096	0.12771924	0.127719	0.825117	29	25	17	23.66667	31	24	33	29.33333
envZ	240	85	71.66667	1.037972	0.497075	-1.09672	1.096722514	0.13319855	-0.1332	0.699966	1941	1943	1778	1887.333	2001	1858	2018	1959
epd	764	179	86.66667	1.027939	0.523336	-1.10159	1.101592202	0.139590252	-0.13959	0.718594	3397	3276	2633	3102	3268	3089	3209	3188.667
erfK	378	271	295	1.703498	0.000828	1.503875	1.50387535	0.588684992	0.588685	0.006598	493	398	367	419.3333	745	669	729	714.3333
eutA	383	256	281	3.554545	1.7E-05	3.221955	3.221954615	1.687936172	1.687936	0.000286	223	46	61	110	427	317	429	391
eutB	737	487	527	3.90625	4.4E-05	3.56775	3.567750062	1.835014551	1.835015	0.000623	392	68	84	181.3333	805	571	749	708.3333
eutC	270	183	186.3333	3.740196	0.000177	3.433109	3.433108759	1.779515562	1.779516	0.001886	154	29	21	68	291	212	260	254.3333
eutD	1227	723	848	4.087379	3.54E-05	3.747572	3.747571946	1.905956175	1.905956	0.000526	597	130	97	274.6667	1324	853	1191	1122.667
eutE	2179	1299	1553.333	4.123324	1.52E-05	3.755242	3.755241511	1.908905695	1.908906	0.000261	1047	192	253	497.3333	2371	1552	2229	2050.667
eutG	650	339	416.3333	3.739035	0.000248	3.412023	3.412023344	1.770627517	1.770628	0.002391	339	49	68	152	699	407	599	568.3333
eutH	566	341	371.6667	3.423913	3.78E-05	3.113488	3.11348772	1.638531589	1.638532	0.000555	313	78	69	153.3333	635	419	521	525
eutJ	798	449	559.3333	4.283757	3.82E-06	3.890838	3.890838052	1.960080933	1.960081	8.21E-05	354	73	84	170.3333	871	533	785	729.6667
eutK	255	156	174.3333	2.494286	6.16E-05	2.242581	2.242580927	1.165160048	1.16516	0.000806	203	80	67	116.6667	315	236	322	291
eutL	321	188	211	2.543902	3.14E-05	2.275595	2.27559483	1.186243708	1.186244	0.00048	233	84	93	136.6667	405	281	357	347.6667
eutM	1232	750	815	3.683864	0.000615	3.379635	3.379634588	1.756867268	1.756867	0.005192	695	85	131	303.6667	1317	881	1158	1118.667
eutN	1075	663	748.3333	3.953947	7.7E-05	3.614379	3.614379412	1.853747958	1.853748	0.000959	556	88	116	253.3333	1163	779	1063	1001.667
eutP	1339	1044	966	3.656279	0.000572	3.376955	3.376954708	1.75572283	1.755723	0.004859	840	125	126	363.6667	1464	1170	1355	1329.667
eutQ	3380	2273	2261	3.759561	0.001136	3.48325	3.483250001	1.800434022	1.800434	0.008463	1944	289	225	819.3333	3605	2562	3074	3080.333
eutR	192	138	136.6667	2.070496	0.00011	1.850114	1.85011392	0.887614107	0.887614	0.001281	196	102	85	127.6667	276	240	277	264.3333
eutS	945	671	610.6667	3.234146	0.002564	2.994364	2.994364003	1.58224961	1.58225	0.015874	640	105	75	273.3333	1020	776	856	884
eutT	2484	1582	1708	4.035545	0.000192	3.716594	3.716593915	1.893981064	1.893981	0.001988	1285	210	193	562.6667	2677	1792	2343	2270.667
exbB	416	84	-120.667	-1.14213	0.054625	-1.29375	1.293750434	0.371559346	-0.37156	0.165784	975	1175	759	969.6667	894	810	843	849
exbD	168	52	-36.3333	-1.0968	0.093754	-1.24699	1.246991088	0.318451155	-0.31845	0.240408	393	505	337	411.6667	410	341	375	375.3333
exo	245	146	152	1.375618	0.1176	1.213825	1.213825106	0.279560566	0.279561	0.278805	491	364	359	404.6667	604	510	556	556.6667
exoX	246	114	145.3333	1.181667	0.737945	1.041525	1.041524991	0.058697457	0.058697	0.868252	873	807	720	800	966	921	949	945.3333
fabA	687	43	-285.667	-1.2664	0.019492	-1.46665	1.466649124	0.552523767	-0.55252	0.076704	1015	1357	1702	1358	1095	1052	1070	1072.333
fabB	12476	4296	-8070.33	-1.93252	4.29E-05	-2.23575	2.235752855	1.160760718	-1.16076	0.00061	13006	16200	20968	16724.67	8710	8761	8492	8654.333
fabD	340	93	-122.333	-1.0747	0.13328	-1.23235	1.232345408	0.301406679	-0.30141	0.301153	1631	1794	1855	1760	1724	1515	1674	1637.667
fabF	1732	505	958.3333	1.14141	0.991438	1.001763	1.001762685	0.002540778	0.002541	1	7181	6676	6474	6777	8206	7115	7885	7735.333
fabG	898	472	-391	-1.0698	0.211059	-1.21851	1.218507692	0.285115358	-0.28512	0.412165	6305	5916	5757	5992.667	5954	5407	5444	5601.667
fabH	222	138	26.33333	1.020509	0.398623	-1.12503	1.125029903	0.169963348	-0.16996	0.614365	1188	1254	1410	1284	1349	1211	1371	1310.333
fabI	1920	425	-1082	-1.2655	0.028823	-1.45761	1.457607177	0.543601968	-0.5436	0.104086	4382	5239	5851	5157.333	4338	3931	3957	4075.333
fabZ	625	255	262.6667	1.104426	0.842089	-1.03012	1.030115667	0.04280634	-0.04281	0.939636	2744	2423	2379	2515.333	3004	2678	2652	2778
fadA	993	218	-38	-1.02495	0.411672	-1.14604	1.146035191	0.196651345	-0.19665	0.62552	1896	1884	903	1561	1521	1415	1633	1523

Feature ID	Experiment - Range (original values)	Experiment - IQR (original values)	Experiment - Difference (original values)	Experiment - Fold Change (original values)	EDGE test: yccT NT vs WT NT , tagwise dispersions - P-value	EDGE test: yccT NT vs WT NT , tagwise dispersions - Fold change	yccT NT vs WT NT ABS|FC|	yccT NT vs WT NT Log2FC	yccT NT vs WT NT Log2FC +-	EDGE test: yccT NT vs WT NT , tagwise dispersions - FDR p-value correction	WT NT - WT.1.S22 - Expression values	WT NT - WT.2.S23 - Expression values	WT NT - WT.3.S24 - Expression values	WT NT - Means	yccT NT - yccT.1.S28 - Expression values	yccT NT - yccT.2.S29 - Expression values	yccT NT - yccT.3.S30 - Expression values	yccT NT - Means
fadB	1104	283	4.333333	1.001987	0.444766	-1.12901	1.129013098	0.175062223	-0.17506	0.655826	2210	2718	1614	2180.667	2260	1977	2318	2185
fadD	268	184	206	1.426501	0.065598	1.25375	1.253750048	0.326249756	0.32625	0.19035	486	505	458	483	671	670	726	689
fadH	1208	540	-1.33333	-1.00066	0.47008	-1.12659	1.126594306	0.171968085	-0.17197	0.677153	2292	2478	1270	2013.333	2296	1752	1988	2012
fadL	749	134	-276.333	-1.26143	0.019641	-1.4405	1.440498279	0.526567937	-0.52657	0.077019	1037	1742	1221	1333.333	993	1022	1156	1057
fadR	1144	639	560	1.16859	0.839214	1.031594	1.031593682	0.044874843	0.044875	0.937824	3842	3203	2920	3321.667	4064	3684	3897	3881.667
fba	24512	9256	-18260	-1.83537	0.000111	-2.11898	2.118976767	1.08336777	-1.08337	0.001288	30502	44600	45254	40118.67	23588	20742	21246	21858.67
fbaB	141	105	82.66667	1.574074	0.028622	1.405634	1.40563383	0.491220818	0.491221	0.103615	218	113	101	144	242	218	220	226.6667
fbp	5001	1524	-2851	-1.31081	0.019536	-1.49924	1.499244756	0.584235927	-0.58424	0.076742	10488	13927	11656	12023.67	9628	8926	8964	9172.667
fdhD	318	21	134	1.146555	0.952285	1.00783	1.007830118	0.011252476	0.011252	1	955	965	823	914.3333	1141	976	1028	1048.333
fdhE	657	212	-0.66667	-1.00027	0.39317	-1.13387	1.133865296	0.181249257	-0.18125	0.61	2775	2552	2118	2481.667	2700	2340	2403	2481
fdhF	878	309	-199.667	-1.04552	0.268531	-1.19029	1.19028688	0.25130933	-0.25131	0.481141	5053	4175	4529	4585.667	4554	4220	4384	4386
fdnG	501	57	-189.667	-1.1076	0.103451	-1.27084	1.27083743	0.345779487	-0.34578	0.257234	1865	1802	2190	1952.333	1771	1689	1828	1762.667
fdnH	306	66	-144	-1.34839	0.007046	-1.54123	1.541228576	0.62408084	-0.62408	0.035036	671	365	636	557.3333	455	396	389	413.3333
fdnI	264	22	-112.667	-1.27934	0.01229	-1.45436	1.454357555	0.540382001	-0.54038	0.054002	629	365	554	516	391	406	413	403.3333
fdoG	7217	1326	-1621	-1.13769	0.157973	-1.30051	1.300512601	0.379080378	-0.37908	0.337502	11057	17477	11648	13394	12676	10260	12383	11773
fdoH	1112	493	76.66667	1.032965	0.554551	-1.10339	1.103392624	0.141946241	-0.14195	0.740126	2065	3012	1900	2325.667	2618	2048	2541	2402.333
fdoI	837	216	99.33333	1.064769	0.667612	-1.06825	1.068247922	0.09524651	-0.09525	0.820909	1392	2023	1186	1533.667	1843	1448	1608	1633
fdrA	22	17	14	1.336	0.274127	1.177691	1.177690826	0.235960844	0.235961	0.487423	52	36	37	41.66667	58	54	55	55.66667
fdx	284	55	-148.667	-1.31676	0.002271	-1.5092	1.509202261	0.593786167	-0.59379	0.014337	543	697	614	618	507	413	488	469.3333
feoA	127	38	52	1.227074	0.497477	1.090315	1.090314752	0.124744672	0.124745	0.699966	281	243	163	229	285	268	290	281
feoB	1144	168	506.6667	1.212587	0.648176	1.071447	1.071447218	0.099560781	0.099561	0.806988	2585	2646	1919	2383.333	3063	2753	2854	2890
fepA	105	54	48.33333	1.170588	0.767293	1.036502	1.03650182	0.051722649	0.051723	0.886077	334	277	239	283.3333	331	344	320	331.6667
fepB	29	11	1.666667	1.022831	0.441399	-1.11335	1.113351919	0.154909685	-0.15491	0.652816	78	74	67	73	85	56	83	74.66667
fepC	126	47	75	1.364668	0.117541	1.204841	1.204841298	0.268843126	0.268843	0.278805	245	200	172	205.6667	297	247	298	280.6667
fepD	44	11	-14	-1.28	0.034543	-1.46322	1.463224445	0.549151083	-0.54915	0.1193	46	86	60	64	51	42	57	50
fepE	753	534	561.3333	1.903918	7.5E-05	1.682177	1.682176959	0.75032948	0.750329	0.000949	786	526	551	621	1279	1085	1183	1182.333
fepG	124	21	-58.3333	-1.33461	0.001252	-1.52493	1.524932068	0.608744976	-0.60874	0.009143	207	273	218	232.6667	186	149	188	174.3333
fes	14	8	-1	-1.03947	0.454027	-1.17461	1.17460519	0.232175917	-0.23218	0.663083	29	32	18	26.33333	20	28	28	25.33333
ffh	1558	579	388.6667	1.058773	0.662161	-1.07165	1.071648608	0.099831926	-0.09983	0.817507	7591	6215	6033	6613	7489	6722	6794	7001.667
fhlA	3886	475	-2362.67	-1.64501	0.000859	-1.89942	1.899416963	0.925556643	-0.92556	0.006785	4177	7306	6594	6025.667	3867	3420	3702	3663
fhuA	140	78	90.33333	1.284664	0.283955	1.134137	1.134137099	0.18159505	0.181595	0.498898	356	318	278	317.3333	418	409	396	407.6667
fhuB	45	8	22.33333	1.15367	0.898699	1.016691	1.016690679	0.023880816	0.023881	0.971085	150	158	128	145.3333	172	173	158	167.6667
fhuC	40	13	5	1.050847	0.612081	-1.07334	1.073341192	0.102108751	-0.10211	0.78182	112	111	72	98.33333	96	109	105	103.3333
fhuD	73	22	2	1.014493	0.424285	-1.11343	1.113434119	0.155016198	-0.15502	0.636644	173	141	100	138	160	119	141	140
fhuE	78	25	39	1.317935	0.200095	1.164999	1.164999328	0.220329122	0.220329	0.3973	145	126	97	122.6667	175	159	151	161.6667
fhuF	77	41	49.33333	1.418079	0.05912	1.250552	1.250551843	0.322564867	0.322565	0.176176	131	117	106	118	158	161	183	167.3333

Feature ID	Experiment - Range (original values)	Experiment - IQR (original values)	Experiment - Difference (original values)	Experiment - Fold Change (original values)	EDGE test: yccT NT vs WT NT , tagwise dispersions - P-value	EDGE test: yccT NT vs WT NT , tagwise dispersions - Fold change	yccT NT vs WT NT ABS|FC|	yccT NT vs WT NT Log2FC	yccT NT vs WT NT Log2FC +-	EDGE test: yccT NT vs WT NT , tagwise dispersions - FDR p-value correction	WT NT - WT.1.S22 - Expression values	WT NT - WT.2.S23 - Expression values	WT NT - WT.3.S24 - Expression values	WT NT - Means	yccT NT - yccT.1.S28 - Expression values	yccT NT - yccT.2.S29 - Expression values	yccT NT - yccT.3.S30 - Expression values	yccT NT - Means
fic	90	41	33	1.290323	0.37277	1.147612	1.147611807	0.198634716	0.198635	0.590331	166	94	81	113.6667	171	135	134	146.6667
fidL	95	51	53.66667	1.609848	0.013399	1.421412	1.421412174	0.507324961	0.507325	0.05781	119	66	79	88	161	134	130	141.6667
fimA	232	44	5	1.018138	0.550419	-1.11488	1.114882704	0.156891933	-0.15689	0.737684	384	152	291	275.6667	293	249	300	280.6667
fimC	61	35	37.66667	2.506667	0.002192	2.223239	2.223239409	1.152663313	1.152663	0.013966	45	7	23	25	68	58	62	62.66667
fimD	80	54	57	1.863636	0.000142	1.643273	1.643273431	0.716572556	0.716573	0.001582	82	55	61	66	119	115	135	123
fimF	27	18	13.33333	1.4	0.23388	1.234461	1.234460566	0.303880751	0.303881	0.441551	46	28	26	33.33333	53	34	53	46.66667
fimH	23	11	5	1.114504	0.925425	-1.02011	1.020114911	0.028731674	-0.02873	0.985238	50	42	39	43.66667	53	35	58	48.66667
fimI	59	14	32.66667	1.924528	0.006763	1.689209	1.689208933	0.756347782	0.756348	0.034055	49	22	35	35.33333	77	46	81	68
fimW	34	17	26.33333	1.908046	0.001077	1.680231	1.680231326	0.74865987	0.74866	0.008089	30	31	26	29	47	59	60	55.33333
fimY	71	33	40	1.839161	0.00181	1.624404	1.624403926	0.699910419	0.69991	0.012187	67	41	35	47.66667	106	74	83	87.66667
fimZ	47	25	28.66667	2.433333	0.003221	2.151258	2.151257616	1.1051803	1.10518	0.019226	35	5	20	20	49	45	52	48.66667
fis	146	10	58.66667	1.159565	0.959214	1.008529	1.008528902	0.012252429	0.012252	1	308	394	401	367.6667	454	391	434	426.3333
fixA	78	55	52.33333	1.730233	0.003714	1.536605	1.536604554	0.619745934	0.619746	0.021344	103	58	54	71.66667	132	127	113	124
fixB	98	66	67	1.975728	2.94E-05	1.748529	1.74852882	0.806141576	0.806142	0.000458	93	62	51	68.66667	149	128	130	135.6667
fixC	106	82	74	1.720779	0.002213	1.525694	1.525694323	0.609465943	0.609466	0.014025	144	82	82	102.6667	188	164	178	176.6667
fixX	21	5	12.66667	1.863636	0.021461	1.633343	1.633343336	0.707828083	0.707828	0.082316	16	12	16	14.66667	33	28	21	27.33333
fklB	295	102	-160.333	-1.24641	0.009863	-1.43522	1.435216075	0.521267955	-0.52127	0.045847	686	868	879	811	735	584	633	650.6667
fkpA	8895	2328	-5753.67	-2.18121	7.68E-06	-2.53596	2.535961792	1.342533009	-1.34253	0.000149	7126	11185	13563	10624.67	5147	4668	4798	4871
fldA	323	62	-35	-1.01391	0.331197	-1.15358	1.153578665	0.206116388	-0.20612	0.545487	2744	2486	2421	2550.333	2652	2424	2470	2515.333
fldB	654	73	48	1.023758	0.471966	-1.10514	1.105138317	0.144226946	-0.14423	0.678436	2343	2029	1689	2020.333	2216	1958	2031	2068.333
flgA	143	92	-114	-1.57	4.31E-06	-1.79197	1.791966969	0.841544045	-0.84154	9.09E-05	292	323	327	314	200	216	184	200
flgB	277	78	-168.333	-2.30155	7.45E-08	-2.67172	2.671717654	1.417767553	-1.41777	2.78E-06	208	281	404	297.6667	130	131	127	129.3333
flgC	249	69	-158.333	-2.36103	8.95E-07	-2.71143	2.711426629	1.439052132	-1.43905	2.46E-05	179	308	337	274.6667	88	151	110	116.3333
flgD	869	434	-638	-2.56757	2.24E-12	-2.92848	2.928479267	1.55015168	-1.55015	1.99E-10	867	1212	1056	1045	343	445	433	407
flgE	4517	2713	-3372	-3.07977	2.01E-13	-3.50943	3.509429198	1.811236398	-1.81124	2.23E-11	4350	6077	4553	4993.333	1637	1667	1560	1621.333
flgF	1559	979	-1193.67	-2.91293	0	-3.31977	3.319772949	1.731084574	-1.73108	0	1616	2134	1703	1817.667	637	660	575	624
flgG	1319	875	-1049	-2.60561	4.66E-15	-2.971	2.970996135	1.570946729	-1.57095	6.26E-13	1520	1951	1636	1702.333	645	683	632	653.3333
flgH	606	318	-416.333	-1.95053	5.61E-09	-2.21376	2.213757837	1.146497414	-1.1465	2.86E-07	796	1020	747	854.3333	429	471	414	438
flgI	731	316	-466.667	-2.01156	3.36E-09	-2.2887	2.288698132	1.194527191	-1.19453	1.77E-07	831	1169	784	928	478	438	468	461.3333
flgJ	762	400	-530.667	-2.30492	2.56E-12	-2.62221	2.622214299	1.390785594	-1.39079	2.18E-10	845	1156	811	937.3333	411	394	415	406.6667
flgK	7713	275	2887.667	1.239548	0.583436	1.101547	1.101546588	0.139530512	0.139531	0.762148	13904	13930	8330	12054.67	16043	14179	14605	14942.33
flgL	6202	534	2498.333	1.264832	0.508053	1.12006	1.120060042	0.163576071	0.163576	0.707919	10537	10790	6974	9433.667	13176	11071	11549	11932
flgM	4072	1781	1973.333	1.410427	0.21015	1.267086	1.2670865	0.341515016	0.341515	0.410933	6672	4891	2861	4808	6933	6805	6606	6781.333
flgN	6567	2394	2984.333	1.403197	0.227873	1.258739	1.258738748	0.331978882	0.331979	0.433536	10106	7712	4387	7401.667	10954	10072	10132	10386
flhA	235	110	-151.333	-1.42193	0.000149	-1.62078	1.620781147	0.696689298	-0.69669	0.001635	472	581	477	510	362	346	368	358.6667
flhB	82	33	32.66667	1.168675	0.89709	1.020303	1.020302875	0.028997477	0.028997	0.97046	160	227	194	193.6667	234	203	242	226.3333

Feature ID	Experiment - Range (original values)	Experiment - IQR (original values)	Experiment - Difference (original values)	Experiment - Fold Change (original values)	EDGE test: yccT NT vs WT NT , tagwise dispersions - P-value	EDGE test: yccT NT vs WT NT , tagwise dispersions - Fold change	yccT NT vs WT NT ABS|FC|	yccT NT vs WT NT Log2FC	yccT NT vs WT NT Log2FC +-	EDGE test: yccT NT vs WT NT , tagwise dispersions - FDR p-value correction	WT NT - WT.1.S22 - Expression values	WT NT - WT.2.S23 - Expression values	WT NT - WT.3.S24 - Expression values	WT NT - Means	yccT NT - yccT.1.S28 - Expression values	yccT NT - yccT.2.S29 - Expression values	yccT NT - yccT.3.S30 - Expression values	yccT NT - Means
flhC	904	378	557.6667	1.281935	0.419518	1.125225	1.125224535	0.170212916	0.170213	0.632274	2196	1804	1934	1978	2708	2312	2587	2535.667
flhD	882	334	476	1.21949	0.65705	1.0695	1.069500354	0.09693696	0.096937	0.814931	2381	1912	2213	2168.667	2794	2547	2593	2644.667
flhE	87	40	-55.6667	-1.87435	3.04E-08	-2.12981	2.129807617	1.090723119	-1.09072	1.33E-06	113	143	102	119.3333	73	62	56	63.66667
fliA	4246	930	1451.667	1.219395	0.629943	1.089475	1.089475198	0.123633354	0.123633	0.793879	8573	6950	4327	6616.667	8508	7817	7880	8068.333
fliB	893	228	-291.333	-1.13546	0.089371	-1.29345	1.293447088	0.371221038	-0.37122	0.232944	2335	2869	2122	2442	2369	1976	2107	2150.667
fliD	16050	4168	1473.667	1.042022	0.661772	-1.08209	1.082086313	0.113815581	-0.11382	0.817313	38118	41569	25519	35068.67	39098	36579	33950	36542.33
fliE	65	22	-31.6667	-2.11765	0.00029	-2.47156	2.471564342	1.305424465	-1.30542	0.002741	39	57	84	60	44	22	19	28.33333
fliF	391	199	-289.667	-2.45318	2.2E-11	-2.82697	2.826973427	1.499258322	-1.49926	1.62E-09	390	495	582	489	191	191	216	199.3333
fliG	572	361	-466.333	-2.41599	2.82E-13	-2.76097	2.760972287	1.465176407	-1.46518	2.97E-11	687	890	810	795.6667	318	344	326	329.3333
fliH	638	368	-468	-2.77722	1.7E-16	-3.16722	3.167215565	1.663215063	-1.66322	2.6E-14	633	892	669	731.3333	265	254	271	263.3333
fliI	731	315	-472.333	-2.74938	2.39E-13	-3.14596	3.145960902	1.653500741	-1.6535	2.58E-11	589	961	677	742.3333	274	230	306	270
fliJ	552	317	-414.333	-4.19537	2.94E-25	-4.78685	4.78684912	2.259076333	-2.25908	7.66E-23	447	681	504	544	130	129	130	129.6667
fliK	1996	1003	-1399	-3.74673	0	-4.27324	4.273243561	2.095331548	-2.09533	0	1502	2494	1729	1908.333	498	531	499	509.3333
fliL	601	299	-418.667	-2.84978	2.78E-15	-3.25127	3.251268792	1.701002833	-1.701	3.98E-13	527	824	584	645	228	223	228	226.3333
fliM	1643	676	-1100.67	-2.6787	4.67E-12	-3.06274	3.062736562	1.61482128	-1.61482	3.83E-10	1321	2287	1661	1756.333	644	678	645	655.6667
fliN	856	345	-590.667	-2.97327	1.02E-13	-3.41558	3.415575505	1.772128685	-1.77213	1.22E-11	639	1139	892	890	321	294	283	299.3333
fliO	341	79	-197.333	-2.41289	1.01E-08	-2.77268	2.772681606	1.471281958	-1.47128	4.88E-07	220	468	323	337	151	127	141	139.6667
fliP	437	100	-249.333	-1.78571	5.86E-06	-2.04008	2.040076089	1.028622961	-1.02862	0.000118	428	731	541	566.6667	294	328	330	317.3333
fliQ	54	13	-3	-1.0205	0.25871	-1.162	1.161995554	0.216604548	-0.2166	0.470584	131	185	132	149.3333	154	145	140	146.3333
fliR	169	62	25.66667	1.073055	0.741989	-1.04924	1.04923854	0.069342706	-0.06934	0.871621	357	433	264	351.3333	326	417	388	377
fliS	1719	504	108	1.023651	0.528416	-1.10739	1.107386334	0.147158623	-0.14716	0.722435	4774	5322	3603	4566.333	5169	4270	4584	4674.333
fliT	2659	284	124	1.023173	0.552452	-1.10658	1.106581943	0.146110287	-0.14611	0.738781	5420	6646	3987	5351	6095	5136	5194	5475
fliY	1599	417	729	1.19581	0.717447	1.058293	1.058293046	0.081739171	0.081739	0.852738	4226	3917	3026	3723	4625	4334	4397	4452
fliZ	1866	295	792.6667	1.250606	0.51573	1.108114	1.108114437	0.148106878	0.148107	0.715445	3688	3393	2408	3163	4274	3658	3935	3955.667
fljB	150330	74713	-5784	-1.01503	0.43141	-1.15091	1.150908264	0.202772845	-0.20277	0.642547	414331	453849	303519	390566.3	439248	339618	375481	384782.3
flk	333	108	206.6667	1.416667	0.070437	1.250571	1.250571429	0.322587462	0.322587	0.1993	559	519	410	496	738	627	743	702.6667
fmt	1446	874	902.3333	1.347497	0.259795	1.192246	1.192246221	0.25368221	0.253682	0.471204	3221	2347	2222	2596.667	3668	3214	3615	3499
fnr	1760	1512	981.3333	1.308046	0.366604	1.161873	1.161872593	0.216451876	0.216452	0.583568	4227	2662	2668	3185.667	4422	4180	3899	4167
focA	1074	869	932.3333	1.457999	0.110655	1.275164	1.27516384	0.350682625	0.350683	0.26793	2021	1941	2145	2035.667	2999	2890	3015	2968
folA	101	30	69.33333	1.232662	0.524563	1.079788	1.07978831	0.110748503	0.110749	0.719276	306	294	294	298	395	324	383	367.3333
folB	46	9	13.66667	1.117143	0.848805	-1.02455	1.024550025	0.034990428	-0.03499	0.943819	115	113	122	116.6667	155	109	127	130.3333
folC	368	122	224.3333	1.225008	0.59575	1.070397	1.070396615	0.098145459	0.098145	0.771186	993	981	1017	997	1349	1115	1200	1221.333
folD	611	352	336.3333	1.260791	0.403877	1.115752	1.115751719	0.15801603	0.158016	0.618162	1560	1215	1094	1289.667	1705	1567	1606	1626
folE	635	201	314.6667	1.315719	0.232369	1.164363	1.164362617	0.219540426	0.21954	0.439582	1168	1018	804	996.6667	1439	1276	1219	1311.333
folK	66	34	39.66667	1.347953	0.165895	1.18624	1.186240273	0.246396258	0.246396	0.350372	111	131	100	114	150	145	166	153.6667
folP	238	96	-148.333	-1.23446	0.00764	-1.41542	1.41541788	0.501228049	-0.50123	0.037617	714	822	807	781	696	584	618	632.6667

Feature ID	Experiment - Range (original values)	Experiment - IQR (original values)	Experiment - Difference (original values)	Experiment - Fold Change (original values)	EDGE test: yccT NT vs WT NT , tagwise dispersions - P-value	EDGE test: yccT NT vs WT NT , tagwise dispersions - Fold change	yccT NT vs WT NT ABS|FC|	yccT NT vs WT NT Log2FC	yccT NT vs WT NT Log2FC +-	EDGE test: yccT NT vs WT NT , tagwise dispersions - FDR p-value correction	WT NT - WT.1.S22 - Expression values	WT NT - WT.2.S23 - Expression values	WT NT - WT.3.S24 - Expression values	WT NT - Means	yccT NT - yccT.1.S28 - Expression values	yccT NT - yccT.2.S29 - Expression values	yccT NT - yccT.3.S30 - Expression values	yccT NT - Means
foxA	362	177	222	1.468025	0.040132	1.300423	1.300422732	0.37898068	0.378981	0.133401	595	448	380	474.3333	742	625	722	696.3333
fpr	379	29	118.3333	1.08176	0.704608	-1.04967	1.049674922	0.069942603	-0.06994	0.845374	1532	1525	1285	1447.333	1664	1531	1502	1565.667
frdA	43582	23924	-34135	-1.53075	0.001818	-1.75574	1.755739299	0.812078642	-0.81208	0.012207	87919	102427	105002	98449.33	67528	63995	61420	64314.33
frdB	14211	10330	-10995	-1.36527	0.01081	-1.56304	1.563036931	0.644351866	-0.64435	0.048926	39627	40988	42673	41096	32544	29297	28462	30101
frdC	6501	2315	-3815.33	-1.33926	0.014683	-1.53707	1.537071455	0.620184235	-0.62018	0.062259	14527	13640	17017	15061.33	11897	11325	10516	11246
frdD	6750	4281	-4761.67	-1.23569	0.043119	-1.40965	1.409654349	0.495341453	-0.49534	0.140702	25940	24070	24884	24964.67	21630	19789	19190	20203
frr	437	111	43.66667	1.013199	0.435443	-1.12537	1.125371741	0.170401642	-0.1704	0.646382	3443	3278	3204	3308.333	3589	3152	3315	3352
fruA	1022	175	-119.667	-1.16491	0.20946	-1.38395	1.383949696	0.468791505	-0.46879	0.410309	428	658	1450	845.3333	747	572	858	725.6667
fruF	543	168	326	2.092737	0.001538	1.809529	1.80952936	0.855614516	0.855615	0.010685	319	181	395	298.3333	662	487	724	624.3333
fruK	250	100	137	1.649289	0.050551	1.421783	1.421782552	0.507700836	0.507701	0.15752	195	144	294	211	355	295	394	348
fruR	380	203	124.6667	1.031692	0.521768	-1.10583	1.105828614	0.145127808	-0.14513	0.717998	3856	4084	3861	3933.667	4064	3875	4236	4058.333
frwB	134	44	-51.3333	-1.14995	0.044052	-1.32259	1.322594629	0.403370948	-0.40337	0.142686	337	402	442	393.6667	381	338	308	342.3333
frwC	50	19	-35.3333	-1.1271	0.033702	-1.28126	1.281258873	0.357561996	-0.35756	0.11694	323	318	299	313.3333	273	280	281	278
frwD	51	22	18.33333	1.061043	0.554071	-1.07285	1.072848587	0.101446481	-0.10145	0.739849	321	281	299	300.3333	332	321	303	318.6667
fsr	444	335	289	1.585415	0.018597	1.413653	1.413653286	0.499428327	0.499428	0.073984	702	400	379	493.6667	790	735	823	782.6667
ftn	577	189	329.3333	1.169905	0.880422	1.022389	1.022388991	0.031944207	0.031944	0.962171	1805	1991	2019	1938.333	2241	2180	2382	2267.667
ftnB	210	167	170	1.81862	0.000163	1.601012	1.601011504	0.678983675	0.678984	0.001766	241	175	207	207.6667	374	374	385	377.6667
ftsA	1167	325	-7.66667	-1.00195	0.414027	-1.13437	1.134372211	0.181894096	-0.18189	0.626983	4515	3926	3348	3929.667	4115	3663	3988	3922
ftsE	347	40	100	1.077022	0.667695	-1.05677	1.056769437	0.079660648	-0.07966	0.820909	1355	1355	1185	1298.333	1532	1315	1348	1398.333
ftsI	979	275	491.3333	1.171256	0.834702	1.032042	1.0320423	0.045502103	0.045502	0.934689	3154	2901	2552	2869	3531	3176	3374	3360.333
ftsJ	656	338	-358.333	-1.08378	0.176289	-1.23526	1.235258182	0.304812612	-0.30481	0.364166	4789	4576	4541	4635.333	4495	4203	4133	4277
ftsK	2704	1251	1367.667	1.261487	0.500126	1.114196	1.114196261	0.156003379	0.156003	0.703064	6245	4994	4452	5230.333	7156	5940	6698	6598
ftsL	488	229	231	1.197605	0.647097	1.059966	1.059966473	0.084018633	0.084019	0.80587	1385	1156	966	1169	1454	1328	1418	1400
ftsN	619	135	-200	-1.12487	0.081259	-1.28734	1.287335039	0.364387575	-0.36439	0.219815	1541	2125	1739	1801.667	1676	1506	1623	1601.667
ftsQ	712	205	79.66667	1.054244	0.630975	-1.06757	1.067566533	0.094325984	-0.09433	0.794727	1874	1370	1162	1468.667	1584	1486	1575	1548.333
ftsW	931	488	387.3333	1.251733	0.456755	1.114011	1.114010941	0.155763401	0.155763	0.665313	2009	1488	1119	1538.667	2050	1752	1976	1926
ftsX	180	43	87	1.074359	0.634185	-1.06321	1.063205499	0.088420472	-0.08842	0.797185	1121	1210	1179	1170	1301	1248	1222	1257
ftsY	110	63	-29.3333	-1.01209	0.337802	-1.15535	1.155351496	0.208331834	-0.20833	0.551582	2392	2502	2470	2454.667	2463	2413	2400	2425.333
ftsZ	6320	3442	2391	1.196698	0.721522	1.063671	1.063671259	0.089052337	0.089052	0.85563	15887	11013	9567	12155.67	15386	13799	14455	14546.67
fucA	60	31	40.33333	1.237721	0.451402	1.091167	1.09116659	0.125871377	0.125871	0.660337	182	173	154	169.6667	204	212	214	210
fucI	186	26	-105.667	-1.14286	0.03931	-1.30771	1.307713944	0.387046993	-0.38705	0.131259	782	889	865	845.3333	760	703	756	739.6667
fucK	64	30	-21	-1.04412	0.155962	-1.1938	1.193796028	0.255556358	-0.25556	0.33372	483	496	512	497	513	466	449	476
fucO	143	82	80.33333	1.256383	0.381368	1.112957	1.112956845	0.154397653	0.154398	0.59775	378	296	266	313.3333	396	409	376	393.6667
fucR	251	143	164.3333	1.358025	0.182752	1.192095	1.192095056	0.253499279	0.253499	0.371904	427	511	439	459	582	610	678	623.3333
fucU	28	11	-15.6667	-1.13128	0.038291	-1.29134	1.291336551	0.368865048	-0.36887	0.128346	131	131	143	135	123	115	120	119.3333
fumA	8545	2438	-4920.33	-1.84464	6.28E-05	-2.11413	2.114134082	1.080066878	-1.08007	0.000814	8330	13895	10012	10745.67	6234	5350	5892	5825.333

Feature ID	Experiment - Range (original values)	Experiment - IQR (original values)	Experiment - Difference (original values)	Experiment - Fold Change (original values)	EDGE test: yccT NT vs WT NT , tagwise dispersions - P-value	EDGE test: yccT NT vs WT NT , tagwise dispersions - Fold change	yccT NT vs WT NT ABS|FC|	yccT NT vs WT NT Log2FC	yccT NT vs WT NT Log2FC +-	EDGE test: yccT NT vs WT NT , tagwise dispersions - FDR p-value correction	WT NT - WT.1.S22 - Expression values	WT NT - WT.2.S23 - Expression values	WT NT - WT.3.S24 - Expression values	WT NT - Means	yccT NT - yccT.1.S28 - Expression values	yccT NT - yccT.2.S29 - Expression values	yccT NT - yccT.3.S30 - Expression values	yccT NT - Means
fumB	14419	3582	3416	1.14555	0.998317	-1.00044	1.00043564	0.000628359	-0.00063	1	27079	15492	27838	23469.67	29911	27164	23582	26885.67
fumC	1467	394	-262	-1.06234	0.237709	-1.21022	1.210224872	0.27527514	-0.27528	0.446686	4266	5256	3872	4464.667	4575	3789	4244	4202.667
fur	498	231	313.3333	1.108658	0.861502	-1.02696	1.026957184	0.038376034	-0.03838	0.951	2976	2888	2787	2883.667	3285	3187	3119	3197
fusA	28827	7668	-17653.7	-2.2995	2.07E-06	-2.64603	2.646027074	1.403827823	-1.40383	4.97E-05	21850	41020	30846	31238.67	14380	12193	14182	13585
fxsA	312	90	-197.667	-1.60081	5.01E-05	-1.84786	1.84786017	0.88585559	-0.88586	0.000689	417	536	627	526.6667	345	315	327	329
gabD	92	52	63	1.945	4.94E-05	1.707465	1.707464828	0.77185586	0.771856	0.000687	78	53	69	66.66667	145	121	123	129.6667
gabP	56	29	27.33333	1.328	0.22416	1.176515	1.176514717	0.234519368	0.234519	0.428125	108	79	63	83.33333	119	113	100	110.6667
gabT	131	47	65.66667	1.360806	0.141766	1.203967	1.203967491	0.267796437	0.267796	0.31408	225	178	143	182	258	211	274	247.6667
galE	793	67	-337	-1.18767	0.040063	-1.33866	1.338659396	0.420788933	-0.42079	0.133272	2448	2295	1655	2132.667	1825	1804	1758	1795.667
galF	710	89	-107.667	-1.03344	0.296704	-1.18223	1.182230448	0.241511282	-0.24151	0.512736	2959	3669	3354	3327.333	3277	3194	3188	3219.667
galK	1294	295	-730.667	-1.3559	0.004458	-1.54163	1.541632393	0.624458791	-0.62446	0.024479	2729	3209	2413	2783.667	2126	1915	2118	2053
galM	1440	602	-876.333	-1.37264	0.004235	-1.56645	1.566448455	0.647497297	-0.6475	0.023546	2990	3667	3027	3228	2440	2227	2388	2351.667
galP	857	203	-24.6667	-1.0128	0.37441	-1.13988	1.139879767	0.188881658	-0.18888	0.592301	2249	2216	1392	1952.333	2043	1900	1840	1927.667
galR	1159	270	344.3333	1.128675	0.988587	1.002307	1.00230734	0.003324953	0.003325	1	3212	2763	2053	2676	3092	2936	3033	3020.333
galS	379	169	213	1.298738	0.311446	1.142639	1.142639069	0.192369765	0.19237	0.526523	687	856	596	713	975	856	947	926
galT	664	166	-325	-1.20644	0.020572	-1.36929	1.369292865	0.453431044	-0.45343	0.079681	1979	2131	1588	1899.333	1711	1467	1545	1574.333
galU	723	232	-276.667	-1.05883	0.242184	-1.21113	1.211128029	0.276351381	-0.27635	0.452335	4594	5189	5155	4979.333	4826	4816	4466	4702.667
gapA	61176	26039	-45687.3	-2.93244	2.24E-09	-3.37899	3.378988282	1.756591347	-1.75659	1.24E-07	49971	83707	74311	69329.67	24464	22531	23932	23642.33
garD	31265	11060	14838	1.726273	0.12496	1.527265	1.527265306	0.610950699	0.610951	0.289893	32479	7104	21708	20430.33	38369	32768	34668	35268.33
garK	13926	4032	6717.667	1.784621	0.096848	1.57271	1.572709859	0.65325254	0.653253	0.245781	13197	2997	9491	8561.667	16923	13523	15392	15279.33
garL	16782	4373	8121.667	1.728509	0.127104	1.517298	1.517297758	0.601504231	0.601504	0.293493	16290	3871	13284	11148.33	20653	17657	19500	19270
garR	31954	10401	15156.67	1.772249	0.119522	1.56614	1.566139895	0.647213086	0.647213	0.281854	31555	6171	21154	19626.67	38125	30510	35715	34783.33
gcd	117	11	30	1.111111	0.850198	-1.02327	1.023269506	0.033186168	-0.03319	0.94484	285	294	231	270	348	274	278	300
gcl	19	5	0.333333	1.002257	0.288891	-1.13529	1.135290861	0.183061963	-0.18306	0.505193	156	137	150	147.6667	148	151	145	148
gcp	355	171	179.3333	1.232297	0.485833	1.088994	1.088994383	0.122996513	0.122997	0.690863	913	742	661	772	1016	911	927	951.3333
gcpE	1637	1020	863.6667	1.212412	0.665131	1.070137	1.070137438	0.097796094	0.097796	0.819576	4802	3782	3614	4066	5251	4664	4874	4929.667
gcvA	123	12	-32.6667	-1.05358	0.132174	-1.20373	1.203727291	0.267508581	-0.26751	0.299576	638	663	626	642.3333	661	540	628	609.6667
gcvH	2615	770	434.6667	1.02566	0.51848	-1.11592	1.115916185	0.158228673	-0.15823	0.715919	16152	17537	17130	16939.67	18767	16360	16996	17374.33
gcvP	15374	907	5964	1.135023	0.990659	-1.002	1.002001773	0.002885061	-0.00289	1	46851	46262	39398	44170.33	54772	45944	49687	50134.33
gcvR	498	249	191	1.120581	0.933506	-1.01121	1.011208959	0.01608115	-0.01608	0.989544	1852	1501	1399	1584	1897	1678	1750	1775
gcvT	11523	7319	9012.333	1.312058	0.407462	1.150966	1.150965569	0.202844676	0.202845	0.621646	29436	29196	28009	28880.33	39532	36515	37631	37892.67
gdhA	1714	1171	1151.667	1.610208	0.023926	1.430158	1.430158111	0.516174653	0.516175	0.089748	2496	1652	1514	1887.333	3228	2823	3066	3039
ggt	80	73	37.66667	1.184641	0.712603	1.047297	1.04729717	0.066670865	0.066671	0.849487	257	178	177	204	251	223	251	241.6667
ghmA	410	157	-179	-1.06813	0.194668	-1.22144	1.221443473	0.288587099	-0.28859	0.388372	2756	2754	2909	2806.333	2784	2599	2499	2627.333
gidA	174	27	33.66667	1.044849	0.481225	-1.09687	1.096872226	0.133395477	-0.1334	0.686713	671	810	771	750.6667	845	744	764	784.3333
gidB	120	70	-33	-1.07657	0.11878	-1.23863	1.238628118	0.308743102	-0.30874	0.280552	393	487	512	464	463	392	438	431

Feature ID	Experiment - Range (original values)	Experiment - IQR (original values)	Experiment - Difference (original values)	Experiment - Fold Change (original values)	EDGE test: yccT NT vs WT NT , tagwise dispersions - P-value	EDGE test: yccT NT vs WT NT , tagwise dispersions - Fold change	yccT NT vs WT NT ABS|FC|	yccT NT vs WT NT Log2FC	yccT NT vs WT NT Log2FC +-	EDGE test: yccT NT vs WT NT , tagwise dispersions - FDR p-value correction	WT NT - WT.1.S22 - Expression values	WT NT - WT.2.S23 - Expression values	WT NT - WT.3.S24 - Expression values	WT NT - Means	yccT NT - yccT.1.S28 - Expression values	yccT NT - yccT.2.S29 - Expression values	yccT NT - yccT.3.S30 - Expression values	yccT NT - Means
gip	51	36	18.66667	1.209738	0.621899	1.073471	1.073471202	0.102283488	0.102283	0.787112	122	74	71	89	110	111	102	107.6667
gldA	3582	469	-1506.67	-1.15184	0.109792	-1.30601	1.306011284	0.385167362	-0.38517	0.266861	12970	10449	10869	11429.33	9980	10400	9388	9922.667
glgA	2078	1794	1291.667	1.34147	0.290745	1.192769	1.192768918	0.254314569	0.254315	0.5074	5010	3216	3122	3782.667	5200	5099	4924	5074.333
glgB	995	201	-392	-1.14656	0.090206	-1.31929	1.319287341	0.399758818	-0.39976	0.234705	2834	2878	3488	3066.667	2866	2493	2665	2674.667
glgC	2929	2076	1463.667	1.320371	0.350031	1.178625	1.178625216	0.237105038	0.237105	0.56564	6379	3877	3450	4568.667	6214	5930	5953	6032.333
glgP	2380	1472	1281	1.154102	0.924033	1.015661	1.015660964	0.022418899	0.022419	0.984632	9376	7658	7904	8312.667	10038	9256	9487	9593.667
glgS	2129	1583	1182.667	1.706914	0.037425	1.545446	1.545446104	0.628023343	0.628023	0.126588	2786	1203	1030	1673	2832	3159	2576	2855.667
glgX	1027	777	415.3333	1.145919	0.925979	1.014783	1.014782986	0.021171236	0.021171	0.985354	3524	2518	2497	2846.333	3372	3295	3118	3261.667
glk	600	459	324.6667	1.147643	0.927036	1.013705	1.013705343	0.01963836	0.019638	0.986005	2547	2074	1976	2199	2576	2533	2462	2523.667
glmS	2096	1114	-473.667	-1.03947	0.306193	-1.19027	1.190274585	0.251294428	-0.25129	0.521303	11462	13384	12576	12474	12782	11288	11931	12000.33
glmU	1239	790	955.3333	1.200126	0.757238	1.050795	1.050795332	0.071481696	0.071482	0.880907	4653	4874	4794	4773.667	5892	5584	5711	5729
glnA	4054	3496	2653	1.785376	0.026891	1.608176	1.60817632	0.685425592	0.685426	0.098476	5639	2229	2266	3378	6283	5762	6048	6031
glnB	99	55	71.33333	1.059149	0.556349	-1.07789	1.077887052	0.108206011	-0.10821	0.741429	1197	1193	1228	1206	1292	1252	1288	1277.333
glnD	1082	533	661.3333	1.292108	0.371279	1.141418	1.141418142	0.190827399	0.190827	0.58839	2587	2260	1945	2264	3027	2793	2956	2925.333
glnE	738	262	270.6667	1.113725	0.893391	-1.01995	1.019953104	0.028502821	-0.0285	0.968783	2635	2373	2132	2380	2870	2444	2638	2650.667
glnG	174	43	96.33333	1.153887	0.929766	1.011505	1.011505148	0.016503662	0.016504	0.98796	656	613	609	626	783	655	729	722.3333
glnH	1107	946	872	1.55565	0.024398	1.376932	1.376932228	0.461457553	0.461458	0.091153	1899	1415	1394	1569.333	2462	2501	2361	2441.333
glnK	13	3	0	-1	0.796683	-1.12737	1.127366549	0.172956666	-0.17296	0.905332	15	2	6	7.666667	7	4	12	7.666667
glnL	134	85	93	1.251578	0.412623	1.103535	1.103534505	0.14213174	0.142132	0.625818	401	373	335	369.6667	458	461	469	462.6667
glnP	328	202	225.3333	1.650626	0.004366	1.462264	1.462264269	0.548204067	0.548204	0.024002	454	299	286	346.3333	600	501	614	571.6667
glnQ	333	181	252.6667	1.598736	0.006449	1.403216	1.403215692	0.488736787	0.488737	0.032844	435	427	404	422	679	608	737	674.6667
glnS	933	276	451.6667	1.090484	0.77386	-1.04665	1.046649562	0.065778482	-0.06578	0.889719	4935	5184	4856	4991.667	5789	5211	5330	5443.333
gloA	345	223	-237.333	-1.18639	0.021258	-1.35341	1.35341288	0.436602024	-0.4366	0.081693	1477	1588	1467	1510.667	1333	1243	1244	1273.333
gloB	166	147	141.6667	1.238095	0.49305	1.088156	1.088156386	0.12188591	0.121886	0.697625	627	577	581	595	743	739	728	736.6667
glpA	22107	5709	6764.667	1.103993	0.891363	-1.02365	1.023646584	0.033717708	-0.03372	0.968079	75519	66216	53412	65049	75131	68385	71925	71813.67
glpB	7620	2979	2835	1.126568	0.978444	-1.00458	1.004584544	0.006598984	-0.0066	1	25693	22714	18790	22399	26410	23365	25927	25234
glpC	6126	2820	3417	1.228878	0.622138	1.085054	1.085054265	0.117767196	0.117767	0.787112	16935	14877	12976	14929.33	19102	17697	18240	18346.33
glpD	9131	1169	-4155.67	-1.12869	0.144821	-1.28869	1.288690231	0.365905518	-0.36591	0.318499	34079	40064	35198	36447	33031	30933	32910	32291.33
glpE	71	48	-26.3333	-1.09658	0.077494	-1.24746	1.247464266	0.31899849	-0.319	0.213115	301	315	281	299	253	247	318	272.6667
glpF	6058	1089	-830.333	-1.02466	0.361072	-1.17038	1.170380261	0.226977343	-0.22698	0.577584	33013	37322	33187	34507.33	35665	31264	34102	33677
glpG	176	31	-33.3333	-1.03464	0.194587	-1.18163	1.181628857	0.240776964	-0.24078	0.388372	951	1063	973	995.6667	982	887	1018	962.3333
glpK	34394	8699	-14067.3	-1.15652	0.112295	-1.31568	1.315677456	0.39580585	-0.39581	0.27057	107051	114641	90143	103945	98842	80247	90544	89877.67
glpQ	876	442	-582.667	-1.18262	0.053242	-1.34761	1.347608005	0.430400903	-0.4304	0.163376	3752	3943	3625	3773.333	3322	3183	3067	3190.667
glpR	251	120	-122.667	-1.1735	0.037918	-1.35221	1.352206311	0.435315286	-0.43532	0.127769	663	914	912	829.6667	783	663	675	707
glpT	1136	501	-765	-1.39157	0.002935	-1.59487	1.594867182	0.673436284	-0.67344	0.017816	2386	3010	2760	2718.667	2102	1885	1874	1953.667
glpX	1286	452	-319.667	-1.12759	0.129555	-1.29552	1.295517046	0.373527998	-0.37353	0.296753	2254	3442	2779	2825	2706	2156	2654	2505.333

Feature ID	Experiment - Range (original values)	Experiment - IQR (original values)	Experiment - Difference (original values)	Experiment - Fold Change (original values)	EDGE test: yccT NT vs WT NT , tagwise dispersions - P-value	EDGE test: yccT NT vs WT NT , tagwise dispersions - Fold change	yccT NT vs WT NT ABS|FC|	yccT NT vs WT NT Log2FC	yccT NT vs WT NT Log2FC +-	EDGE test: yccT NT vs WT NT , tagwise dispersions - FDR p-value correction	WT NT - WT.1.S22 - Expression values	WT NT - WT.2.S23 - Expression values	WT NT - WT.3.S24 - Expression values	WT NT - Means	yccT NT - yccT.1.S28 - Expression values	yccT NT - yccT.2.S29 - Expression values	yccT NT - yccT.3.S30 - Expression values	yccT NT - Means
gltA	8635	2608	-3217.33	-1.19165	0.086828	-1.34385	1.343853477	0.426375847	-0.42638	0.229281	23765	21119	15130	20004.67	18126	15518	16718	16787.33
gltB	678	259	382.6667	1.171139	0.84934	1.028349	1.028349439	0.040330584	0.040331	0.94416	2409	2169	2130	2236	2808	2428	2620	2618.667
gltD	386	137	83	1.084464	0.713577	-1.0491	1.049103228	0.069156641	-0.06916	0.849962	1017	1101	830	982.6667	1216	922	1059	1065.667
gltI	1089	647	804.3333	2.371802	6.36E-08	2.109642	2.109642188	1.076998327	1.076998	2.41E-06	714	581	464	586.3333	1228	1391	1553	1390.667
gltJ	322	180	205.6667	1.69482	0.001542	1.504034	1.504034217	0.588837389	0.588837	0.010695	369	286	233	296	484	466	555	501.6667
gltK	203	128	149.3333	1.965517	2.6E-05	1.724959	1.72495856	0.786561703	0.786562	0.000415	143	171	150	154.6667	278	288	346	304
gltL	283	181	210	2.027732	3.05E-05	1.794828	1.794828044	0.843845632	0.843846	0.000471	198	261	154	204.3333	379	427	437	414.3333
gltP	147	78	81.66667	1.13992	0.993443	1.001645	1.001645017	0.002371309	0.002371	1	633	547	571	583.6667	694	653	649	665.3333
gltS	409	125	-257.667	-1.27916	0.004603	-1.46868	1.468680015	0.554520107	-0.55452	0.025148	1058	1222	1262	1180.667	983	853	933	923
gltX	771	402	479.3333	1.161537	0.905036	1.018617	1.018617453	0.026612341	0.026612	0.974536	2902	3177	2823	2967.333	3594	3304	3442	3446.667
glxK	45	16	11.33333	1.162679	0.826519	1.031657	1.031657202	0.044963674	0.044964	0.928106	94	66	49	69.66667	82	77	84	81
glxR	46	26	13	1.104839	0.8667	-1.02452	1.0245208	0.034949275	-0.03495	0.954598	154	110	108	124	148	136	127	137
glyA	5053	339	-3094.67	-1.35459	0.015529	-1.5616	1.561604974	0.643029553	-0.64303	0.06461	9314	12934	13218	11822	9042	8165	8975	8727.333
glyQ	272	104	88.66667	1.051203	0.53915	-1.08937	1.089369232	0.123493025	-0.12349	0.72879	1623	1790	1782	1731.667	1895	1686	1880	1820.333
glyS	783	316	-275.667	-1.09936	0.155046	-1.26524	1.265241405	0.339412674	-0.33941	0.33296	2605	3157	3388	3050	2951	2635	2737	2774.333
gmd	23	8	-8.66667	-1.1831	0.050048	-1.34109	1.34109068	0.423406791	-0.42341	0.15639	59	65	44	56	48	42	52	47.33333
gmk	547	153	-222	-1.08341	0.175549	-1.243	1.242997083	0.313822911	-0.31382	0.363649	2621	2898	3132	2883.667	2774	2626	2585	2661.667
gnd	4385	1044	-2925.67	-1.54915	0.001526	-1.78236	1.782359089	0.833788023	-0.83379	0.010658	6318	9568	8874	8253.333	5526	5274	5183	5327.667
gntK	43	12	-23	-1.26953	0.007697	-1.45107	1.451072189	0.537119293	-0.53712	0.037811	99	102	124	108.3333	81	88	87	85.33333
gntR	397	70	82	1.058101	0.598899	-1.07004	1.070038035	0.097662078	-0.09766	0.773381	1603	1425	1206	1411.333	1559	1426	1495	1493.333
gntT	573	50	-282.333	-1.45611	0.000871	-1.66859	1.668587286	0.738627157	-0.73863	0.006871	683	1158	863	901.3333	633	585	639	619
gntU	106	10	-53.3333	-1.25806	0.007693	-1.44007	1.440069151	0.52613809	-0.52614	0.037811	213	304	263	260	206	198	216	206.6667
gor	708	271	-374	-1.1261	0.100873	-1.28628	1.286279477	0.363204139	-0.3632	0.252952	3417	3285	3318	3340	3175	2709	3014	2966
gph	126	66	-73.3333	-1.11224	0.055391	-1.26997	1.269965236	0.344789005	-0.34479	0.167649	705	757	718	726.6667	690	631	639	653.3333
gpmA	6389	1368	-3200.33	-1.82018	0.000159	-2.06482	2.06482495	1.046019479	-1.04602	0.001726	6101	10071	5135	7102.333	4257	3767	3682	3902
gpmB	394	86	220.6667	1.180136	0.770736	1.038165	1.038164855	0.054035555	0.054036	0.887872	1289	1266	1120	1225	1471	1352	1514	1445.667
gppA	869	125	-455	-1.16967	0.067542	-1.3419	1.341897834	0.424274835	-0.42427	0.194336	2746	3399	3265	3136.667	2820	2530	2695	2681.667
gpsA	3155	1021	-2075	-1.35154	0.009552	-1.55148	1.551475055	0.633640501	-0.63364	0.04493	6940	8670	8323	7977.667	6274	5515	5919	5902.667
gpt	473	298	301.3333	1.412033	0.084715	1.244483	1.244483467	0.315547064	0.315547	0.225659	875	636	683	731.3333	1109	981	1008	1032.667
greA	473	217	-205.667	-1.08509	0.159567	-1.24434	1.244343352	0.315384623	-0.31538	0.339923	2377	2759	2732	2622.667	2591	2286	2374	2417
greB	165	24	48	1.087167	0.755939	-1.03983	1.039834698	0.056354202	-0.05635	0.880907	602	587	463	550.6667	596	628	572	598.6667
grpE	1088	281	-664.667	-1.29101	0.017232	-1.4829	1.482903489	0.568424707	-0.56842	0.069795	2550	2969	3327	2948.667	2269	2344	2239	2284
grxA	638	247	-457.333	-1.37713	0.001376	-1.58175	1.5817547	0.661525883	-0.66153	0.009769	1436	1768	1806	1670	1281	1189	1168	1212.667
grxB	2486	1218	479.6667	1.091925	0.853516	-1.03174	1.031735289	0.045072868	-0.04507	0.946945	6825	4490	4339	5218	6220	5708	5165	5697.667
grxC	794	84	-429.333	-1.26227	0.018623	-1.45275	1.452751103	0.53878755	-0.53879	0.074007	1708	2092	2399	2066.333	1624	1682	1605	1637
gshA	627	133	-132	-1.04202	0.262228	-1.19517	1.195171612	0.257217787	-0.25722	0.474024	3201	3068	3552	3273.667	3357	2925	3143	3141.667

Feature ID	Experiment - Range (original values)	Experiment - IQR (original values)	Experiment - Difference (original values)	Experiment - Fold Change (original values)	EDGE test: yccT NT vs WT NT , tagwise dispersions - P-value	EDGE test: yccT NT vs WT NT , tagwise dispersions - Fold change	yccT NT vs WT NT ABS|FC|	yccT NT vs WT NT Log2FC	yccT NT vs WT NT Log2FC +-	EDGE test: yccT NT vs WT NT , tagwise dispersions - FDR p-value correction	WT NT - WT.1.S22 - Expression values	WT NT - WT.2.S23 - Expression values	WT NT - WT.3.S24 - Expression values	WT NT - Means	yccT NT - yccT.1.S28 - Expression values	yccT NT - yccT.2.S29 - Expression values	yccT NT - yccT.3.S30 - Expression values	yccT NT - Means
gshB	395	250	277.3333	1.166567	0.865644	1.023195	1.023194563	0.033080503	0.033081	0.954146	1642	1737	1616	1665	2011	1924	1892	1942.333
gsk	355	133	189	1.292419	0.283652	1.143137	1.143137385	0.192998801	0.192999	0.498755	762	636	541	646.3333	841	769	896	835.3333
gsp	1011	180	-522.667	-1.22035	0.037153	-1.39774	1.397743776	0.483099921	-0.4831	0.12608	2481	3315	2888	2894.667	2496	2304	2316	2372
gst	1965	1597	979.6667	1.208203	0.679154	1.071001	1.071001055	0.098959902	0.09896	0.827568	6012	4047	4057	4705.333	5974	5654	5427	5685
gtrA	40	8	20	2.132075	0.004931	1.888607	1.888606988	0.917322514	0.917323	0.026386	20	19	14	17.66667	27	54	32	37.66667
gtrB	8	2	-0.33333	-1.05882	0.749292	-1.19332	1.19332079	0.254981922	-0.25498	0.876484	11	4	3	6	6	7	4	5.666667
gtrC	19	10	13	1.639344	0.049781	1.440082	1.440081824	0.526150786	0.526151	0.155887	19	24	18	20.33333	37	34	29	33.33333
guaA	459	177	-195	-1.09377	0.139519	-1.25489	1.254888964	0.327559716	-0.32756	0.310589	2055	2391	2378	2274.667	2232	1932	2075	2079.667
guaB	913	439	508.6667	1.766449	0.002902	1.576798	1.576798252	0.656998082	0.656998	0.017637	930	616	445	663.6667	1358	1104	1055	1172.333
guaC	2538	1313	-1958.33	-1.81552	1.6E-05	-2.08794	2.087942189	1.062081767	-1.06208	0.000271	3706	4456	4917	4359.667	2432	2379	2393	2401.333
gudD	22415	11921	10887	1.870148	0.114436	1.673434	1.673434192	0.742811818	0.742812	0.274031	23228	3000	11307	12511.67	25415	20274	24507	23398.67
gudP	22144	12806	10970.67	1.801949	0.103072	1.612855	1.612854728	0.689616499	0.689616	0.256724	24725	4396	11919	13680	26540	21422	25990	24650.67
gutM	470	63	-304	-3.4127	1.29E-08	-3.96321	3.963213711	1.986670765	-1.98667	6.01E-07	187	587	516	430	117	124	137	126
gutQ	1445	183	-748	-1.47969	0.001443	-1.69947	1.699473486	0.765087854	-0.76509	0.010184	1736	2907	2279	2307.333	1663	1462	1553	1559.333
gyrA	1085	489	120	1.024452	0.493738	-1.1212	1.121196272	0.165038853	-0.16504	0.697625	4285	5230	5208	4907.667	5370	4719	4994	5027.667
gyrB	2468	612	-1084	-1.17049	0.079383	-1.34121	1.341208007	0.423533001	-0.42353	0.216858	6555	8390	7381	7442	6882	5922	6270	6358
hcaT	137	60	89	1.177881	0.781859	1.034928	1.03492827	0.049530779	0.049531	0.89543	521	506	474	500.3333	611	566	591	589.3333
hcp	201	77	-10.3333	-1.0186	0.259219	-1.15141	1.151411793	0.203403894	-0.2034	0.470738	647	605	446	566	595	518	554	555.6667
hcr	102	57	3	1.010239	0.351839	-1.1204	1.120402608	0.164017247	-0.16402	0.567115	332	317	230	293	318	260	310	296
helD	632	306	194	1.094634	0.802864	-1.03615	1.036151428	0.05123486	-0.05123	0.909844	2410	1917	1823	2050	2455	2054	2223	2244
hemA	1636	727	994.3333	1.386399	0.185811	1.226563	1.226563026	0.294621368	0.294621	0.375263	3093	2502	2125	2573.333	3761	3229	3713	3567.667
hemB	442	300	-128.333	-1.03581	0.282796	-1.1791	1.179103906	0.237690858	-0.23769	0.497779	3783	3898	3456	3712.333	3777	3477	3498	3584
hemC	1569	430	-27.6667	-1.00487	0.412975	-1.13779	1.137788565	0.186232487	-0.18623	0.625818	6413	5854	4844	5703.667	5952	5424	5652	5676
hemD	381	313	-52.3333	-1.02279	0.294681	-1.16329	1.163293062	0.218214593	-0.21821	0.51085	2497	2431	2118	2348.667	2489	2116	2284	2296.333
hemE	751	295	-347	-1.14241	0.077824	-1.30309	1.303093953	0.381941106	-0.38194	0.213786	2767	2996	2588	2783.667	2680	2245	2385	2436.667
hemF	101	42	47.33333	1.134216	1	1.000554	1.000553741	0.000798658	0.000799	1	393	351	314	352.6667	415	400	385	400
hemG	138	100	-59	-1.1385	0.044523	-1.3089	1.308899662	0.388354507	-0.38835	0.143895	449	484	522	485	510	384	384	426
hemH	169	69	74.66667	1.190314	0.689222	1.050103	1.05010267	0.070530388	0.07053	0.834791	454	385	338	392.3333	507	431	463	467
hemK	84	67	17	1.046196	0.479079	-1.09541	1.095406183	0.131465929	-0.13147	0.684774	329	403	372	368	413	336	406	385
hemL	5079	2793	-3729	-1.72544	5.09E-05	-1.97416	1.974161246	0.981239831	-0.98124	0.000698	7949	9947	8712	8869.333	5397	4868	5156	5140.333
hemN	193	89	-51.3333	-1.02549	0.260974	-1.17306	1.173064058	0.230281798	-0.23028	0.472806	2002	2072	2122	2065.333	2126	1933	1983	2014
hemX	1289	519	-667.667	-1.16867	0.07151	-1.3385	1.33849699	0.420613894	-0.42061	0.201694	4222	5027	4629	4626	4328	3738	3809	3958.333
hemY	1297	274	-762.667	-1.33539	0.008812	-1.53537	1.535373133	0.618589308	-0.61859	0.042075	2556	3366	3188	3036.667	2471	2069	2282	2274
hepA	354	97	231	1.277422	0.407456	1.115049	1.11504879	0.157106838	0.157107	0.621646	780	870	848	832.6667	1134	945	1112	1063.667
hflB	2383	897	-1042	-1.07786	0.212754	-1.23292	1.232919549	0.302078664	-0.30208	0.41347	13721	15060	14493	14424.67	14184	12677	13287	13382.67
hflC	1167	686	-852.333	-1.26358	0.019896	-1.44753	1.447531331	0.533594575	-0.53359	0.077673	3853	4190	4215	4086	3486	3048	3167	3233.667

Feature ID	Experiment - Range (original values)	Experiment - IQR (original values)	Experiment - Difference (original values)	Experiment - Fold Change (original values)	EDGE test: yccT NT vs WT NT , tagwise dispersions - P-value	EDGE test: yccT NT vs WT NT , tagwise dispersions - Fold change	yccT NT vs WT NT ABS|FC|	yccT NT vs WT NT Log2FC	yccT NT vs WT NT Log2FC +-	EDGE test: yccT NT vs WT NT , tagwise dispersions - FDR p-value correction	WT NT - WT.1.S22 - Expression values	WT NT - WT.2.S23 - Expression values	WT NT - WT.3.S24 - Expression values	WT NT - Means	yccT NT - yccT.1.S28 - Expression values	yccT NT - yccT.2.S29 - Expression values	yccT NT - yccT.3.S30 - Expression values	yccT NT - Means
hflK	1330	562	-752.333	-1.16303	0.076106	-1.32999	1.329992566	0.411418182	-0.41142	0.20995	5080	5705	5316	5367	4951	4375	4518	4614.667
hflX	1430	261	289.6667	1.033642	0.547706	-1.10167	1.10167063	0.139692961	-0.13969	0.73542	8965	8770	8096	8610.333	9526	8509	8665	8900
hfq	5181	2219	-3595.33	-1.45171	0.003436	-1.66456	1.664561581	0.735142244	-0.73514	0.020217	10012	12772	11880	11554.67	8494	7793	7591	7959.333
hha	960	787	800.6667	2.04845	3.17E-06	1.81572	1.815720377	0.860542043	0.860542	7.18E-05	915	737	639	763.6667	1570	1599	1524	1564.333
hilA	23	16	14.33333	1.413462	0.1491	1.24603	1.246029677	0.31733843	0.317338	0.324649	44	31	29	34.66667	52	48	47	49
hilC	349	267	245.3333	2.28223	0.000371	2.055514	2.055514406	1.039499483	1.039499	0.003379	327	108	139	191.3333	406	457	447	436.6667
hilD	986	620	685.6667	1.532074	0.02212	1.35934	1.359340151	0.442906511	0.442907	0.084059	1555	1291	1020	1288.667	2006	1911	2006	1974.333
himA	1521	701	242.6667	1.04574	0.618585	-1.08328	1.083279699	0.115405789	-0.11541	0.785235	6301	4780	4835	5305.333	5931	5536	5177	5548
himD	1811	1387	1181.667	1.470346	0.097498	1.304695	1.304694598	0.383712141	0.383712	0.24662	3250	2198	2089	2512.333	3900	3585	3597	3694
hisA	166	46	-54.6667	-1.12913	0.047748	-1.29393	1.293934547	0.371764641	-0.37176	0.151609	428	536	470	478	476	370	424	423.3333
hisB	274	118	-8	-1.00554	0.296109	-1.14326	1.14326353	0.193157993	-0.19316	0.512524	1603	1412	1341	1452	1544	1329	1459	1444
hisC	233	48	-52.3333	-1.05593	0.142102	-1.1996	1.199598651	0.262551805	-0.26255	0.314198	1102	950	912	988	978	869	960	935.6667
hisD	198	97	-51	-1.06192	0.120074	-1.205	1.204997493	0.269030145	-0.26903	0.282854	971	880	773	874.6667	887	783	801	823.6667
hisF	133	68	-85	-1.15288	0.030018	-1.3149	1.314899683	0.394952737	-0.39495	0.107668	615	676	632	641	547	543	578	556
hisG	175	90	0	-1	0.296811	-1.13631	1.13631418	0.184361782	-0.18436	0.512736	740	702	589	677	764	612	655	677
hisH	116	65	-6	-1.01246	0.247624	-1.15169	1.151694663	0.20375828	-0.20376	0.458897	497	541	425	487.6667	522	432	491	481.6667
hisI	70	19	-36.3333	-1.06229	0.120905	-1.21324	1.213242493	0.278867934	-0.27887	0.283755	604	631	624	619.6667	604	561	585	583.3333
hisJ	807	496	-577.667	-1.25251	0.019586	-1.43344	1.433442742	0.519484278	-0.51948	0.07687	2716	2964	2916	2865.333	2486	2220	2157	2287.667
hisM	118	42	24.33333	1.064889	0.606099	-1.06698	1.066980113	0.093533287	-0.09353	0.777315	372	431	322	375	440	400	358	399.3333
hisP	145	78	27.66667	1.05003	0.535742	-1.08074	1.080739741	0.112019141	-0.11202	0.726399	589	581	489	553	634	597	511	580.6667
hisQ	119	63	-13.6667	-1.0237	0.211833	-1.16255	1.162554018	0.217297753	-0.2173	0.413012	643	604	524	590.3333	621	541	568	576.6667
hisS	1355	652	825.6667	1.227938	0.621827	1.079351	1.079351098	0.11016423	0.110164	0.787112	4017	3441	3409	3622.333	4764	4093	4487	4448
hlpA	4867	2746	-3657.67	-1.68891	0.000129	-1.94348	1.943482965	0.958644462	-0.95864	0.001452	7841	9177	9883	8967	5817	5016	5095	5309.333
hmpA	147	31	-63.6667	-1.36106	0.002194	-1.54056	1.540562571	0.62345728	-0.62346	0.013966	240	307	173	240	200	169	160	176.3333
hnr	279	47	-151.333	-1.09996	0.09311	-1.25973	1.259730931	0.333115617	-0.33312	0.239522	1545	1699	1752	1665.333	1558	1473	1511	1514
hns	8570	5199	6167.667	1.260309	0.543804	1.107349	1.10734917	0.147110206	0.14711	0.732623	24768	23716	22597	23693.67	31167	29502	28915	29861.33
hofB	45	24	18.33333	1.303867	0.357304	1.157167	1.157166592	0.210596578	0.210597	0.572592	85	52	44	60.33333	76	71	89	78.66667
hofC	59	16	16.66667	1.127877	1	-1.00025	1.000250294	0.000361052	-0.00036	1	161	128	102	130.3333	141	144	156	147
hofQ	56	19	17.66667	1.071429	0.603588	-1.06123	1.061228172	0.08573488	-0.08573	0.77698	260	259	223	247.3333	279	241	275	265
holA	239	71	89	1.078738	0.654433	-1.05797	1.057971445	0.081300689	-0.0813	0.812951	1122	1191	1078	1130.333	1317	1120	1221	1219.333
holB	213	20	94.66667	1.20867	0.612258	1.064744	1.06474423	0.090506911	0.090507	0.78182	489	494	378	453.6667	591	474	580	548.3333
holC	116	41	-56	-1.13804	0.045761	-1.30652	1.306524327	0.385733987	-0.38573	0.147039	410	469	506	461.6667	434	393	390	405.6667
holD	45	27	12.33333	1.053857	0.516792	-1.08102	1.081021078	0.112394653	-0.11239	0.715467	222	255	210	229	255	221	248	241.3333
holE	44	27	28.33333	1.374449	0.134006	1.215538	1.215538482	0.281595568	0.281596	0.302178	93	70	64	75.66667	97	108	107	104
hopD	47	28	26.66667	1.5	0.06514	1.32414	1.324140148	0.405055826	0.405056	0.189268	74	40	46	53.33333	87	68	85	80
hpaA	46	16	31	1.432558	0.071593	1.258958	1.258957869	0.332230004	0.33223	0.201799	76	72	67	71.66667	107	88	113	102.6667

Feature ID	Experiment - Range (original values)	Experiment - IQR (original values)	Experiment - Difference (original values)	Experiment - Fold Change (original values)	EDGE test: yccT NT vs WT NT , tagwise dispersions - P-value	EDGE test: yccT NT vs WT NT , tagwise dispersions - Fold change	yccT NT vs WT NT ABS|FC|	yccT NT vs WT NT Log2FC	yccT NT vs WT NT Log2FC +-	EDGE test: yccT NT vs WT NT , tagwise dispersions - FDR p-value correction	WT NT - WT.1.S22 - Expression values	WT NT - WT.2.S23 - Expression values	WT NT - WT.3.S24 - Expression values	WT NT - Means	yccT NT - yccT.1.S28 - Expression values	yccT NT - yccT.2.S29 - Expression values	yccT NT - yccT.3.S30 - Expression values	yccT NT - Means
hpaB	64	10	-5	-1.03425	0.217672	-1.17302	1.173015774	0.230222414	-0.23022	0.420998	149	184	120	151	153	139	146	146
hpaC	72	45	53	1.418421	0.069794	1.248348	1.248347862	0.320020008	0.32002	0.198497	136	120	124	126.6667	169	178	192	179.6667
hpaD	280	189	183	1.8784	0.001035	1.68434	1.684340494	0.752183812	0.752184	0.00784	311	187	127	208.3333	407	376	391	391.3333
hpaE	247	137	152	1.802817	0.002973	1.615626	1.615626317	0.692093552	0.692094	0.018022	286	169	113	189.3333	360	306	358	341.3333
hpaF	102	55	54	1.848168	0.006265	1.644093	1.644092838	0.717291766	0.717292	0.032056	102	47	42	63.66667	144	96	113	117.6667
hpaG	29	9	1.666667	1.017241	0.395269	-1.11327	1.113274236	0.154809019	-0.15481	0.611535	108	103	79	96.66667	102	100	93	98.33333
hpaH	63	41	45.66667	1.678218	0.002305	1.47539	1.475390486	0.561096837	0.561097	0.014486	65	79	58	67.33333	121	106	112	113
hpaI	82	21	52	2	0.000176	1.764273	1.764272779	0.819073637	0.819074	0.001884	60	62	34	52	116	81	115	104
hpaR	164	68	22.66667	1.075305	0.749082	-1.04526	1.045262733	0.063865618	-0.06387	0.876484	399	269	235	301	338	337	296	323.6667
hpaX	131	86	95	1.650685	0.001353	1.461489	1.461489021	0.547438992	0.547439	0.009636	176	148	114	146	245	234	244	241
hpt	90	36	51.66667	1.106749	0.813787	-1.02889	1.028886009	0.041083154	-0.04108	0.917529	497	489	466	484	556	525	526	535.6667
hrpA	884	219	-466.667	-1.16837	0.063487	-1.33665	1.336653333	0.418625344	-0.41863	0.185683	3017	3476	3222	3238.333	2925	2592	2798	2771.667
hrpB	406	173	240.6667	1.287879	0.309284	1.134043	1.134043066	0.181475428	0.181475	0.524268	938	818	752	836	1158	991	1081	1076.667
hscA	236	75	115.6667	1.105567	0.793268	-1.03427	1.03427067	0.048613791	-0.04861	0.903592	1048	1157	1082	1095.667	1284	1099	1251	1211.333
hscB	123	39	43.66667	1.067876	0.591891	-1.06983	1.069831527	0.097383625	-0.09738	0.767985	631	669	630	643.3333	720	609	732	687
hscC	773	14	-245.333	-1.2866	0.023828	-1.46363	1.463631404	0.549552276	-0.54955	0.089705	861	1593	850	1101.333	884	820	864	856
hsdM	199	154	-5.66667	-1.00704	0.294548	-1.15935	1.159353108	0.213320039	-0.21332	0.510818	697	844	890	810.3333	851	691	872	804.6667
hsdR	332	203	24	1.010348	0.407027	-1.12747	1.12746622	0.173084209	-0.17308	0.621646	2409	2376	2173	2319.333	2497	2165	2368	2343.333
hsdS	107	34	0.666667	1.001553	0.323643	-1.14658	1.146577871	0.197334341	-0.19733	0.538312	362	457	469	429.3333	450	424	416	430
hslJ	198	66	108.3333	1.113955	0.845883	-1.02574	1.025738297	0.036662695	-0.03666	0.941972	944	919	989	950.6667	1050	1010	1117	1059
hslU	1586	761	-1200.67	-1.99038	1.22E-07	-2.28542	2.28541802	1.192458069	-1.19246	4.32E-06	1958	2754	2527	2413	1272	1197	1168	1212.333
hslV	394	56	-247.667	-1.49799	0.000494	-1.72631	1.72631238	0.787693547	-0.78769	0.00433	548	864	823	745	530	492	470	497.3333
htgA	54	12	22.66667	1.235294	0.509388	1.090546	1.090545853	0.125050431	0.12505	0.709334	100	112	77	96.33333	112	114	131	119
htpG	3825	2107	-2648	-3.45034	1.33E-14	-3.91586	3.915858203	1.969328525	-1.96933	1.69E-12	3195	4844	3147	3728.667	1040	1183	1019	1080.667
htrA	831	128	-86.3333	-1.02429	0.315196	-1.17753	1.177529243	0.235762889	-0.23576	0.530032	3483	3427	4010	3640	3927	3179	3555	3553.667
htrB	507	235	287.6667	1.318568	0.222819	1.16629	1.16628985	0.221926376	0.221926	0.427037	1040	923	746	903	1253	1158	1161	1190.667
hupA	2834	492	-1343	-1.16271	0.097125	-1.33643	1.336432118	0.41838656	-0.41839	0.246061	8661	9252	10878	9597	8549	8169	8044	8254
hupB	5138	1756	-1483.67	-1.05211	0.283959	-1.19963	1.199633835	0.262594118	-0.26259	0.498898	32104	28193	29574	29957	30636	26966	27818	28473.33
hutC	412	66	187	1.232298	0.505765	1.090187	1.090186813	0.124575375	0.124575	0.706593	867	931	617	805	1029	933	1014	992
hutG	752	79	353.6667	1.283994	0.345192	1.138518	1.138517574	0.187156561	0.187157	0.559658	1426	1388	922	1245.333	1656	1467	1674	1599
hutH	2336	1379	1695.667	2.195816	6.56E-05	1.953637	1.953636971	0.966162407	0.966162	0.000848	1954	1239	1061	1418	3326	2618	3397	3113.667
hutI	797	189	259.3333	1.139677	0.9486	1.009315	1.009314659	0.013376012	0.013376	0.999617	2121	2012	1437	1856.667	2182	1932	2234	2116
hyaA	149	114	122	2.22	4.43E-08	1.966319	1.966318783	0.975497233	0.975497	1.78E-06	121	101	78	100	215	227	224	222
hyaB	381	245	290.3333	2.563734	1.35E-09	2.277925	2.277924683	1.187720047	1.18772	7.77E-08	246	177	134	185.6667	491	422	515	476
hyaC	277	185	208.3333	2.307531	3.19E-08	2.045246	2.045246486	1.032274722	1.032275	1.37E-06	209	143	126	159.3333	372	328	403	367.6667
hyaD	244	156	186.6667	2.15942	4.96E-08	1.907994	1.907994065	0.932056683	0.932057	1.96E-06	193	161	129	161	373	317	353	347.6667

Feature ID	Experiment - Range (original values)	Experiment - IQR (original values)	Experiment - Difference (original values)	Experiment - Fold Change (original values)	EDGE test: yccT NT vs WT NT , tagwise dispersions - P-value	EDGE test: yccT NT vs WT NT , tagwise dispersions - Fold change	yccT NT vs WT NT ABS|FC|	yccT NT vs WT NT Log2FC	yccT NT vs WT NT Log2FC +-	EDGE test: yccT NT vs WT NT , tagwise dispersions - FDR p-value correction	WT NT - WT.1.S22 - Expression values	WT NT - WT.2.S23 - Expression values	WT NT - WT.3.S24 - Expression values	WT NT - Means	yccT NT - yccT.1.S28 - Expression values	yccT NT - yccT.2.S29 - Expression values	yccT NT - yccT.3.S30 - Expression values	yccT NT - Means
hyaDb	55	32	21	1.496063	0.181139	1.350063	1.350062946	0.433026674	0.433027	0.369874	76	30	21	42.33333	61	67	62	63.33333
hyaE	140	93	110	1.962099	3.34E-06	1.727458	1.727457984	0.788650621	0.788651	7.5E-05	125	114	104	114.3333	222	207	244	224.3333
hyaEb	27	11	14.66667	1.564103	0.080708	1.376058	1.376058176	0.460541465	0.460541	0.21877	35	19	24	26	44	32	46	40.66667
hyaF	357	229	281.3333	2.185393	2.38E-07	1.922845	1.922845348	0.943242733	0.943243	7.99E-06	235	278	199	237.3333	536	464	556	518.6667
hyaFb	47	35	29	1.644444	0.023917	1.456673	1.4566725	0.542676557	0.542677	0.089748	67	32	36	45	79	72	71	74
hybA	1583	596	844.6667	1.155594	0.92419	1.01519	1.015189848	0.021749547	0.02175	0.984632	5889	5293	5104	5428.667	6687	5729	6404	6273.333
hybB	1148	587	278.6667	1.045109	0.570858	-1.09771	1.09771473	0.134503181	-0.1345	0.750362	5928	6051	6554	6177.667	7001	5853	6515	6456.333
hybC	4786	682	-984.667	-1.06248	0.256632	-1.22398	1.223976205	0.291575511	-0.29158	0.468728	15304	15986	18944	16744.67	17393	14158	15729	15760
hybD	1472	233	-189.333	-1.04756	0.271915	-1.20995	1.209950587	0.274948131	-0.27495	0.484852	3793	3857	4861	4170.333	4528	3389	4026	3981
hybE	676	366	130.3333	1.052646	0.577643	-1.09624	1.096236472	0.132559039	-0.13256	0.756364	2274	2282	2871	2475.667	2927	2251	2640	2606
hybF	309	164	13	1.013948	0.38465	-1.13982	1.139818331	0.1888039	-0.1888	0.601405	825	837	1134	932	1017	827	991	945
hybG	360	198	77.66667	1.088392	0.690967	-1.06405	1.064050534	0.089566669	-0.08957	0.835588	745	786	1105	878.6667	1031	854	984	956.3333
hycA	7	5	-2	-1.08824	0.296065	-1.23412	1.234122908	0.303486082	-0.30349	0.512524	26	26	22	24.66667	20	21	27	22.66667
hycB	6	2	1.333333	1.081633	0.928013	-1.05022	1.050220911	0.070692827	-0.07069	0.98657	17	15	17	16.33333	19	20	14	17.66667
hycC	23	4	11	1.197605	0.745453	1.05208	1.052079621	0.073243891	0.073244	0.874531	59	55	53	55.66667	76	57	67	66.66667
hycD	18	8	0	-1	0.541501	-1.14611	1.146108273	0.196743342	-0.19674	0.730644	27	16	33	25.33333	34	19	23	25.33333
hycE	19	11	-1	-1.01083	0.257294	-1.15161	1.151609357	0.203651416	-0.20365	0.469358	100	85	95	93.33333	100	81	96	92.33333
hycF	16	2	2.666667	1.105263	0.893337	-1.03108	1.031084993	0.04416326	-0.04416	0.968783	24	26	26	25.33333	30	19	35	28
hycG	16	7	-3	-1.06475	0.21154	-1.2134	1.21340427	0.279060293	-0.27906	0.41274	50	46	52	49.33333	56	43	40	46.33333
hycH	16	4	-6.33333	-1.18095	0.067214	-1.33979	1.339787925	0.422004655	-0.422	0.19377	47	39	38	41.33333	35	31	39	35
hycI	20	14	-4	-1.06154	0.186148	-1.2112	1.21120416	0.276442065	-0.27644	0.375601	74	57	76	69	71	56	68	65
hydG	543	269	-223	-1.07147	0.193178	-1.21713	1.217125285	0.28347768	-0.28348	0.386793	3555	3463	3012	3343.333	3294	3025	3042	3120.333
hydH	713	55	13.33333	1.005667	0.425694	-1.12442	1.12441598	0.169175863	-0.16918	0.638248	2741	2290	2028	2353	2437	2345	2317	2366.333
hydN	17	1	8.666667	1.273684	0.567657	1.117119	1.117118767	0.159782575	0.159783	0.748361	28	34	33	31.66667	43	33	45	40.33333
hypA	1045	574	-733	-1.54163	7.84E-05	-1.75689	1.756893019	0.813026346	-0.81303	0.000973	1916	2327	2016	2086.333	1342	1436	1282	1353.333
hypB	7323	3942	-4965	-1.4487	0.003089	-1.65243	1.652429314	0.724588559	-0.72459	0.018573	14946	18079	15066	16030.33	11436	10756	11004	11065.33
hypC	2312	958	-1461	-1.50876	0.000937	-1.72151	1.721507461	0.783672433	-0.78367	0.007306	3835	5129	4034	4332.667	2921	2817	2877	2871.667
hypD	6604	2053	-3972	-1.42774	0.004914	-1.63337	1.633365155	0.707847356	-0.70785	0.026336	11545	15341	12888	13258	9492	8737	9629	9286
hypE	4301	886	-2585	-1.46111	0.003473	-1.6782	1.678198531	0.746913397	-0.74691	0.020329	6550	9603	8420	8191	5852	5302	5664	5606
hypF	343	80	206	1.26788	0.371259	1.115494	1.115494396	0.157683266	0.157683	0.58839	822	797	688	769	1017	877	1031	975
hypO	4354	2867	2639.667	1.209099	0.694126	1.066953	1.066952663	0.09349617	0.093496	0.837373	14585	11891	11396	12624	15750	14758	15283	15263.67
iacP	4	1	0.333333	1.04	0.79045	-1.09226	1.09226475	0.127322588	-0.12732	0.901773	8	8	9	8.333333	10	6	10	8.666667
iadA	317	171	249	2.301394	1.35E-07	2.008058	2.008057841	1.005800826	1.005801	4.72E-06	189	181	204	191.3333	463	360	498	440.3333
iagB	18	6	8.666667	2.444444	0.010081	2.135783	2.135782537	1.094764761	1.094765	0.046603	9	5	4	6	11	11	22	14.66667
iap	291	102	161	1.280488	0.327086	1.130629	1.13062883	0.17712539	0.177125	0.541273	602	631	489	574	704	721	780	735
ibpA	1337	514	-1017.67	-2.79694	4.04E-11	-3.22453	3.224526564	1.689087355	-1.68909	2.84E-09	1064	1885	1803	1584	550	601	548	566.3333

Feature ID	Experiment - Range (original values)	Experiment - IQR (original values)	Experiment - Difference (original values)	Experiment - Fold Change (original values)	EDGE test: yccT NT vs WT NT , tagwise dispersions - P-value	EDGE test: yccT NT vs WT NT , tagwise dispersions - Fold change	yccT NT vs WT NT ABS|FC|	yccT NT vs WT NT Log2FC	yccT NT vs WT NT Log2FC +-	EDGE test: yccT NT vs WT NT , tagwise dispersions - FDR p-value correction	WT NT - WT.1.S22 - Expression values	WT NT - WT.2.S23 - Expression values	WT NT - WT.3.S24 - Expression values	WT NT - Means	yccT NT - yccT.1.S28 - Expression values	yccT NT - yccT.2.S29 - Expression values	yccT NT - yccT.3.S30 - Expression values	yccT NT - Means
ibpB	505	211	-355.667	-2.30281	4.59E-10	-2.63413	2.63412689	1.397324844	-1.39732	2.91E-08	487	754	645	628.6667	249	294	276	273
icc	1716	726	613.6667	1.097962	0.843267	-1.03169	1.031689733	0.045009164	-0.04501	0.94024	7116	6241	5436	6264.333	7152	6515	6967	6878
icdA	10077	3702	-5571.33	-1.25777	0.034742	-1.43351	1.433512762	0.519554749	-0.51955	0.119762	26391	30474	24689	27184.67	23456	20397	20987	21613.33
iciA	358	266	249	1.587726	0.010286	1.413014	1.41301389	0.498775647	0.498776	0.047207	556	392	323	423.6667	679	658	681	672.6667
iclR	655	239	259.6667	1.242982	0.46055	1.10424	1.104240419	0.143054315	0.143054	0.668862	1354	1055	797	1068.667	1452	1239	1294	1328.333
idi	435	227	160.6667	1.164617	0.810061	1.032107	1.032107304	0.04559297	0.045593	0.914492	1222	919	787	976	1217	1047	1146	1136.667
idnD	285	147	165.3333	1.377186	0.111519	1.219998	1.219997954	0.286878729	0.286879	0.269434	547	413	355	438.3333	640	560	611	603.6667
idnK	129	1	45	1.115483	0.875598	-1.01941	1.019411847	0.027737024	-0.02774	0.959152	409	410	350	389.6667	479	415	410	434.6667
idnO	290	221	161.3333	1.416882	0.115906	1.260998	1.260998393	0.334566437	0.334566	0.276564	551	303	307	387	593	524	528	548.3333
idnR	831	568	578.6667	1.717652	0.00186	1.528831	1.528830532	0.612428496	0.612428	0.012391	1047	769	603	806.3333	1434	1337	1384	1385
idnT	810	478	495	1.555972	0.021993	1.387433	1.387432977	0.47241808	0.472418	0.083802	1211	818	642	890.3333	1452	1296	1408	1385.333
ileS	1076	709	-115.667	-1.01449	0.366432	-1.15574	1.155742357	0.208819822	-0.20882	0.583568	8380	8314	7605	8099.667	8504	7428	8020	7984
ilvA	802	422	565.6667	1.635581	0.003625	1.439789	1.439788663	0.525857064	0.525857	0.020997	1011	866	793	890	1595	1288	1484	1455.667
ilvB	6318	4312	4535.333	2.625762	1.11E-05	2.352828	2.352827931	1.234395816	1.234396	0.000197	4221	2231	1917	2789.667	8235	7197	6543	7325
ilvC	579	205	152	1.139492	0.943939	1.0098	1.009800147	0.014069792	0.01407	0.99728	1285	1193	791	1089.667	1370	1075	1280	1241.667
ilvD	1471	793	949.3333	1.505144	0.053303	1.327591	1.327591182	0.408810952	0.408811	0.163449	2216	1779	1643	1879.333	3114	2572	2800	2828.667
ilvE	539	226	284.6667	1.215984	0.595385	1.071017	1.071016886	0.098981226	0.098981	0.771019	1475	1278	1201	1318	1740	1564	1504	1602.667
ilvG	1157	812	704.6667	1.904579	0.008123	1.726939	1.726938571	0.788216765	0.788217	0.039381	1345	576	416	779	1573	1490	1388	1483.667
ilvH	129	61	80.33333	1.373643	0.102341	1.211209	1.211208815	0.27644761	0.276448	0.255765	254	203	188	215	317	264	305	295.3333
ilvI	317	262	248	1.577192	0.00894	1.393724	1.393724341	0.478945245	0.478945	0.04255	523	382	384	429.6667	688	646	699	677.6667
ilvM	179	104	106.6667	2.15942	6.67E-05	1.922532	1.922531641	0.943007343	0.943007	0.000857	141	74	61	92	240	178	178	198.6667
ilvN	888	587	642	2.553226	3.26E-08	2.272587	2.2725872	1.184335652	1.184336	1.38E-06	578	349	313	413.3333	1201	1029	936	1055.333
ilvY	222	80	-118.333	-1.35465	0.000961	-1.54597	1.545973249	0.628515356	-0.62852	0.007444	402	539	415	452	362	322	317	333.6667
imp	892	102	413.6667	1.068784	0.675552	-1.06999	1.069986849	0.097593065	-0.09759	0.825117	5926	6026	6090	6014	6818	5988	6477	6427.667
infA	888	383	568.6667	1.312913	0.294325	1.155394	1.155393569	0.20838437	0.208384	0.510818	1926	1891	1635	1817.333	2523	2274	2361	2386
infB	5264	1998	-3503.67	-1.51321	0.001647	-1.74369	1.743688395	0.802142247	-0.80214	0.011315	8850	10846	11296	10330.67	7597	6032	6852	6827
infC	5108	3834	-3378	-1.17149	0.085883	-1.33929	1.33929247	0.421471045	-0.42147	0.228146	22878	23475	22876	23076.33	21686	18367	19042	19698.33
invA	42	7	17.66667	1.350993	0.25909	1.199015	1.199015342	0.261850119	0.26185	0.470697	64	57	30	50.33333	72	69	63	68
invB	25	6	-4	-1.20339	0.223818	-1.3668	1.366804673	0.450807085	-0.45081	0.428027	13	38	20	23.66667	15	23	21	19.66667
invC	28	8	19.33333	1.563107	0.050658	1.370247	1.370247083	0.454436064	0.454436	0.15774	32	36	35	34.33333	58	43	60	53.66667
invE	21	15	13	1.433333	0.14982	1.264417	1.264417147	0.338472505	0.338473	0.325833	40	26	24	30	45	43	41	43
invF	41	18	18.66667	1.57732	0.069873	1.403997	1.403996875	0.489539724	0.48954	0.198594	49	30	18	32.33333	46	48	59	51
invG	59	18	33.66667	1.554945	0.023065	1.37447	1.374470347	0.458875783	0.458876	0.087054	78	61	43	60.66667	102	79	102	94.33333
invH	25	9	8.333333	1.235849	0.656156	1.092822	1.092822091	0.128058553	0.128059	0.814634	35	44	27	35.33333	35	52	44	43.66667
invI	12	4	-4.66667	-1.93333	0.024741	-2.17124	2.171244856	1.118522431	-1.11852	0.091957	7	14	8	9.666667	9	2	4	5
invJ	17	2	-1	-1.03226	0.399252	-1.17073	1.170730732	0.227409294	-0.22741	0.614907	29	42	25	32	31	33	29	31

Feature ID	Experiment - Range (original values)	Experiment - IQR (original values)	Experiment - Difference (original values)	Experiment - Fold Change (original values)	EDGE test: yccT NT vs WT NT , tagwise dispersions - P-value	EDGE test: yccT NT vs WT NT , tagwise dispersions - Fold change	yccT NT vs WT NT ABS|FC|	yccT NT vs WT NT Log2FC	yccT NT vs WT NT Log2FC +-	EDGE test: yccT NT vs WT NT , tagwise dispersions - FDR p-value correction	WT NT - WT.1.S22 - Expression values	WT NT - WT.2.S23 - Expression values	WT NT - WT.3.S24 - Expression values	WT NT - Means	yccT NT - yccT.1.S28 - Expression values	yccT NT - yccT.2.S29 - Expression values	yccT NT - yccT.3.S30 - Expression values	yccT NT - Means
ipk	474	188	313	1.245234	0.544088	1.086794	1.086794236	0.120078819	0.120079	0.732782	1270	1183	1376	1276.333	1653	1458	1657	1589.333
iroB	42	8	14.66667	1.15493	0.915908	1.017127	1.017126666	0.024499354	0.024499	0.981236	103	97	84	94.66667	107	95	126	109.3333
iroC	163	36	76	1.27771	0.315492	1.129123	1.129122679	0.175202243	0.175202	0.530329	316	280	225	273.6667	350	311	388	349.6667
iroD	52	12	24.33333	1.300412	0.307823	1.142758	1.142757738	0.192519588	0.19252	0.523149	80	92	71	81	123	101	92	105.3333
iroE	30	14	18	1.27	0.402724	1.118548	1.118547704	0.161626786	0.161627	0.617037	72	66	62	66.66667	80	82	92	84.66667
iroN	108	93	83.33333	1.822368	0.000186	1.617162	1.617162129	0.693464324	0.693464	0.001943	135	91	78	101.3333	184	186	184	184.6667
ispA	660	266	185.3333	1.074812	0.721791	-1.05407	1.054071451	0.075972664	-0.07597	0.85563	2803	2486	2143	2477.333	2752	2485	2751	2662.667
ispB	649	230	287.3333	1.104841	0.856458	-1.02762	1.027615907	0.039301128	-0.0393	0.948357	2983	2753	2486	2740.667	3135	2910	3039	3028
ispD	152	83	43.33333	1.057522	0.530264	-1.08469	1.084694513	0.117288787	-0.11729	0.723325	714	748	798	753.3333	866	715	809	796.6667
ispF	93	37	13	1.030564	0.422302	-1.11421	1.114210763	0.156022157	-0.15602	0.634824	380	423	473	425.3333	449	412	454	438.3333
ivbL	329	108	146	1.242391	0.445495	1.10485	1.104850456	0.143851111	0.143851	0.655966	731	623	453	602.3333	724	739	782	748.3333
katE	310	48	21.66667	1.040932	0.589012	-1.08545	1.085445807	0.118287699	-0.11829	0.76537	727	417	444	529.3333	676	492	485	551
katG	27773	9135	-18251.7	-1.62299	0.00128	-1.877	1.876995035	0.908424834	-0.90842	0.0093	36782	50449	55414	47548.33	32602	27641	27647	29296.67
kbl	4846	1622	-3292	-1.57202	0.000992	-1.81152	1.811515282	0.857196976	-0.8572	0.007568	7361	9370	10410	9047	5962	5739	5564	5755
kdgK	246	74	77.66667	1.066896	0.606306	-1.06701	1.067007254	0.093569985	-0.09357	0.777334	1278	1071	1134	1161	1317	1191	1208	1238.667
kdgR	1218	684	798.3333	1.247264	0.530169	1.101075	1.101074627	0.138912253	0.138912	0.723325	3592	3201	2893	3228.667	4085	4111	3885	4027
kdgT	2125	49	-798	-5.94628	0.000303	-6.64906	6.649056735	2.733149688	-2.73315	0.002818	545	2229	104	959.3333	185	136	163	161.3333
kdpA	55	2	27	1.623077	0.03178	1.434703	1.434702889	0.520752002	0.520752	0.111941	50	51	29	43.33333	84	75	52	70.33333
kdpB	44	11	22	1.251908	0.420629	1.103967	1.103967003	0.142697052	0.142697	0.633302	98	92	72	87.33333	116	103	109	109.3333
kdpC	26	10	3.333333	1.071429	0.796839	-1.05368	1.0536766	0.075432136	-0.07543	0.905332	52	55	33	46.66667	49	59	42	50
kdpD	273	147	161.3333	1.432916	0.059897	1.272595	1.272595412	0.347773825	0.347774	0.177651	472	362	284	372.6667	557	509	536	534
kdpE	165	49	72.66667	1.303621	0.268158	1.152538	1.152537755	0.204814011	0.204814	0.480862	292	243	183	239.3333	348	255	333	312
kdsA	2799	1076	-2068.33	-2.10527	1.57E-06	-2.43301	2.433011722	1.282743271	-1.28274	3.91E-05	2859	4553	4407	3939.667	2077	1754	1783	1871.333
kdsB	281	41	-121	-1.12415	0.053879	-1.28307	1.283067125	0.359596649	-0.3596	0.164497	1057	1181	1049	1095.667	1016	900	1008	974.6667
kdtA	545	82	249.6667	1.160214	0.885871	1.019059	1.019059332	0.027238051	0.027238	0.964513	1649	1589	1437	1558.333	1982	1671	1771	1808
kdtB	153	69	91.66667	1.132339	0.945379	-1.00902	1.00901893	0.012953241	-0.01295	0.998362	692	658	728	692.6667	781	761	811	784.3333
kduD	80	43	49.33333	1.354916	0.144954	1.193898	1.193898334	0.25567999	0.25568	0.318499	168	122	127	139	202	170	193	188.3333
kduI	59	7	27.66667	1.251515	0.53233	1.095508	1.095508304	0.131600421	0.1316	0.724554	92	117	121	110	124	151	138	137.6667
kefB	146	46	-55	-1.07732	0.096597	-1.22515	1.225151685	0.29296038	-0.29296	0.245426	812	766	721	766.3333	757	711	666	711.3333
kefC	123	42	67	1.544715	0.015913	1.371009	1.371008511	0.455237528	0.455238	0.065849	147	133	89	123	175	183	212	190
kgtP	147	25	77.33333	1.274232	0.3434	1.122485	1.122484919	0.166696062	0.166696	0.557685	294	318	234	282	378	319	381	359.3333
ksgA	382	129	158	1.074063	0.675982	-1.06254	1.0625428	0.087520956	-0.08752	0.825145	2109	2238	2053	2133.333	2435	2145	2294	2291.333
kup	118	87	-26	-1.0188	0.249247	-1.1608	1.160797998	0.215116937	-0.21512	0.460365	1432	1449	1345	1408.667	1450	1332	1366	1382.667
lamB	114848	50049	-66861.7	-1.19085	0.074868	-1.35724	1.357235364	0.440670927	-0.44067	0.207531	438535	418562	394486	417194.3	382874	323687	344437	350332.7
lasT	131	27	41.66667	1.170068	0.796759	1.033044	1.033043576	0.046901112	0.046901	0.905332	264	277	194	245	325	285	250	286.6667
ldcC	462	72	193.6667	1.096688	0.803076	-1.03511	1.035110369	0.049784604	-0.04978	0.909851	2133	2067	1809	2003	2271	2180	2139	2196.667

Feature ID	Experiment - Range (original values)	Experiment - IQR (original values)	Experiment - Difference (original values)	Experiment - Fold Change (original values)	EDGE test: yccT NT vs WT NT , tagwise dispersions - P-value	EDGE test: yccT NT vs WT NT , tagwise dispersions - Fold change	yccT NT vs WT NT ABS|FC|	yccT NT vs WT NT Log2FC	yccT NT vs WT NT Log2FC +-	EDGE test: yccT NT vs WT NT , tagwise dispersions - FDR p-value correction	WT NT - WT.1.S22 - Expression values	WT NT - WT.2.S23 - Expression values	WT NT - WT.3.S24 - Expression values	WT NT - Means	yccT NT - yccT.1.S28 - Expression values	yccT NT - yccT.2.S29 - Expression values	yccT NT - yccT.3.S30 - Expression values	yccT NT - Means
ldhA	381	179	-274.667	-1.37643	0.000307	-1.56438	1.56438132	0.645592215	-0.64559	0.002852	1018	1076	919	1004.333	754	740	695	729.6667
lepA	419	240	312	1.194191	0.754151	1.044167	1.044166722	0.062352086	0.062352	0.880075	1554	1605	1661	1606.667	1973	1845	1938	1918.667
lepB	283	218	163	1.195132	0.794591	1.039009	1.039009393	0.055208697	0.055209	0.903705	775	737	994	835.3333	1020	993	982	998.3333
leuA	1424	1134	1097.667	2.899077	1.64E-07	2.601278	2.601277563	1.379220347	1.37922	5.62E-06	928	395	411	578	1819	1663	1545	1675.667
leuB	1266	837	904	2.585038	2.49E-07	2.31127	2.311269507	1.208685496	1.208685	8.22E-06	841	471	399	570.3333	1665	1450	1308	1474.333
leuC	1308	966	989	2.707135	6.18E-09	2.414016	2.41401602	1.27143525	1.271435	3.08E-07	820	499	419	579.3333	1727	1465	1513	1568.333
leuD	701	517	516.3333	2.459943	2.95E-07	2.183715	2.183715114	1.126784655	1.126785	9.41E-06	503	260	298	353.6667	961	815	834	870
leuL	25	5	13.66667	2	0.011078	1.757978	1.757977754	0.813916815	0.813917	0.049784	16	17	8	13.66667	33	28	21	27.33333
leuO	78	46	48.33333	2.510417	0.000189	2.250868	2.250868217	1.170481593	1.170482	0.001967	55	29	12	32	90	75	76	80.33333
leuS	2300	724	1249.667	1.182203	0.810585	1.03871	1.038709611	0.054792381	0.054792	0.914617	7304	6878	6394	6858.667	8694	7602	8029	8108.333
lexA	1489	335	-495	-1.14437	0.098434	-1.29504	1.295044596	0.373001779	-0.373	0.248102	4565	3804	3402	3923.667	3737	3473	3076	3428.667
lgt	240	74	-61.6667	-1.06693	0.124898	-1.21521	1.215207855	0.281203101	-0.2812	0.289893	966	1091	892	983	968	851	945	921.3333
lig	888	365	444.3333	1.224109	0.597216	1.079097	1.079097212	0.109824839	0.109825	0.772181	2278	1913	1757	1982.667	2645	2264	2372	2427
ligT	173	75	76.66667	1.211592	0.567279	1.073731	1.073730888	0.102632452	0.102632	0.748322	438	363	286	362.3333	448	410	459	439
lipA	517	174	308	1.121579	0.915241	-1.01612	1.01611731	0.023066969	-0.02307	0.980995	2541	2641	2418	2533.333	2935	2715	2874	2841.333
lipB	140	4	68.33333	1.116876	0.872903	-1.01984	1.019838144	0.028340203	-0.02834	0.958007	601	605	548	584.6667	688	602	669	653
livF	38	5	15.66667	1.195021	0.710355	1.051691	1.05169093	0.072710789	0.072711	0.847948	84	89	68	80.33333	106	86	96	96
livG	48	15	20	1.255319	0.55865	1.094991	1.094991359	0.130919485	0.130919	0.743357	77	66	92	78.33333	114	87	94	98.33333
livH	19	8	12	1.154506	0.948535	1.016229	1.016229354	0.023226043	0.023226	0.999617	79	80	74	77.66667	87	93	89	89.66667
livJ	59	9	-30.6667	-1.17692	0.010286	-1.33963	1.33962605	0.421830336	-0.42183	0.047207	213	209	190	204	185	154	181	173.3333
livK	46	22	7.333333	1.061111	0.615929	-1.0732	1.073203386	0.101923511	-0.10192	0.784022	114	134	112	120	146	136	100	127.3333
livM	40	11	26	1.22807	0.545586	1.076981	1.076980856	0.106992606	0.106993	0.733908	114	116	112	114	152	125	143	140
lldD	650	218	-75.6667	-1.11749	0.211959	-1.27667	1.27667025	0.352385941	-0.35239	0.413012	531	1116	512	719.6667	736	466	730	644
lldP	245	57	-73.6667	-1.27834	0.025354	-1.46268	1.462678933	0.548613124	-0.54861	0.093697	239	479	297	338.3333	264	234	296	264.6667
lldR	144	51	-7.66667	-1.04782	0.351734	-1.19539	1.195388835	0.257479974	-0.25748	0.567115	124	262	118	168	184	123	174	160.3333
lnt	269	142	5	1.004069	0.323233	-1.13345	1.133451333	0.180722448	-0.18072	0.538237	1225	1365	1096	1228.667	1293	1133	1275	1233.667
lolA	586	132	-137.667	-1.04232	0.267228	-1.18278	1.182783045	0.242185468	-0.24219	0.479757	3648	3461	3062	3390.333	3333	3224	3201	3252.667
lolB	304	113	169	1.211074	0.591105	1.067223	1.067223095	0.093861792	0.093862	0.767414	865	827	710	800.6667	1014	940	955	969.6667
lon	2154	590	-1432	-1.17495	0.075007	-1.33482	1.334824627	0.41665021	-0.41665	0.207723	10007	10067	8778	9617.333	8455	8188	7913	8185.333
lonH	259	97	173.6667	1.166241	0.858272	1.022791	1.022790611	0.032510822	0.032511	0.949564	1051	1075	1008	1044.667	1267	1148	1240	1218.333
lpdA	27079	1041	-6989	-1.38669	0.057893	-1.53445	1.534451518	0.617723063	-0.61772	0.173095	41982	18304	14903	25063	19225	17734	17263	18074
lpfA	33	22	21.66667	1.477941	0.070935	1.30334	1.303340284	0.382213801	0.382214	0.200455	59	37	40	45.33333	69	62	70	67
lpfB	21	9	13.66667	1.788462	0.023876	1.573225	1.573225221	0.65372522	0.653725	0.089733	21	15	16	17.33333	32	36	25	31
lpfC	64	49	11.66667	1.060241	0.568892	-1.07221	1.072210844	0.100588631	-0.10059	0.749334	200	220	161	193.6667	225	171	220	205.3333
lpfD	141	58	89.66667	1.445364	0.035588	1.274749	1.274749309	0.350213556	0.350214	0.12177	220	212	172	201.3333	313	290	270	291
lpfE	41	9	21.66667	1.369318	0.197807	1.205745	1.205744965	0.269924786	0.269925	0.39428	66	50	60	58.66667	81	91	69	80.33333

Feature ID	Experiment - Range (original values)	Experiment - IQR (original values)	Experiment - Difference (original values)	Experiment - Fold Change (original values)	EDGE test: yccT NT vs WT NT , tagwise dispersions - P-value	EDGE test: yccT NT vs WT NT , tagwise dispersions - Fold change	yccT NT vs WT NT ABS|FC|	yccT NT vs WT NT Log2FC	yccT NT vs WT NT Log2FC +-	EDGE test: yccT NT vs WT NT , tagwise dispersions - FDR p-value correction	WT NT - WT.1.S22 - Expression values	WT NT - WT.2.S23 - Expression values	WT NT - WT.3.S24 - Expression values	WT NT - Means	yccT NT - yccT.1.S28 - Expression values	yccT NT - yccT.2.S29 - Expression values	yccT NT - yccT.3.S30 - Expression values	yccT NT - Means
lplA	214	58	42	1.065015	0.641031	-1.0602	1.060197162	0.084332584	-0.08433	0.801923	759	634	545	646	680	692	692	688
lpp	54086	14963	-31626	-1.18971	0.079926	-1.34916	1.349162849	0.432064498	-0.43206	0.217673	215778	206734	172495	198335.7	176700	161692	161737	166709.7
lppB	808	289	412.6667	1.257648	0.481365	1.102967	1.102967314	0.141390037	0.14139	0.686713	1797	1369	1639	1601.667	2177	1928	1938	2014.333
lpxA	525	179	155.6667	1.037226	0.548647	-1.09785	1.097851154	0.134682468	-0.13468	0.736253	4397	4140	4008	4181.667	4533	4160	4319	4337.333
lpxB	328	146	34	1.021496	0.402452	-1.11534	1.115337762	0.157480673	-0.15748	0.617037	1669	1611	1465	1581.667	1782	1454	1611	1615.667
lpxC	11055	5783	5343.667	1.220476	0.644926	1.08216	1.082160402	0.113914357	0.113914	0.804454	29636	23277	19798	24237	30853	28829	29060	29580.67
lpxD	1331	431	-269.333	-1.04468	0.281332	-1.18575	1.185752282	0.245802645	-0.2458	0.49625	7023	6141	5728	6297.333	6233	5692	6159	6028
lpxH	94	11	53.66667	1.115495	0.853767	-1.02276	1.022764659	0.032474215	-0.03247	0.946945	475	464	455	464.6667	549	475	531	518.3333
lpxK	106	19	30	1.066323	0.571195	-1.07282	1.07282469	0.101414346	-0.10141	0.750583	443	462	452	452.3333	538	432	477	482.3333
lpxO	139	82	83.33333	1.390016	0.084808	1.231186	1.231186465	0.300049276	0.300049	0.225697	261	200	180	213.6667	282	290	319	297
lrhA	963	702	721	1.751042	0.001777	1.543157	1.543157094	0.625884936	0.625885	0.012024	1165	782	933	960	1745	1635	1663	1681
lrp	1111	363	-296.667	-1.06533	0.220549	-1.22218	1.222178058	0.289454485	-0.28945	0.424708	4804	4441	5269	4838	4891	4158	4575	4541.333
lspA	813	252	141.3333	1.051481	0.626048	-1.07641	1.076411169	0.106229266	-0.10623	0.789419	3178	2693	2365	2745.333	2982	2733	2945	2886.667
ltaA	351	161	120	1.103181	0.834917	-1.02669	1.026690161	0.038000864	-0.038	0.934693	1363	1111	1015	1163	1366	1211	1272	1283
luxS	761	119	-156	-1.04851	0.252989	-1.19187	1.191869157	0.253225866	-0.25323	0.464371	3828	3067	3221	3372	3444	3102	3102	3216
lysA	1018	764	765.3333	2.251908	9.86E-06	2.001475	2.001474664	1.001063354	1.001063	0.000181	869	437	528	611.3333	1383	1292	1455	1376.667
lysC	756	510	484	1.545045	0.031906	1.372337	1.372336752	0.456634542	0.456635	0.1122	1237	700	727	888	1456	1209	1451	1372
lysP	702	303	213.3333	1.124465	0.974711	-1.00459	1.004594438	0.006613192	-0.00661	1	2128	1588	1426	1714	2093	1798	1891	1927.333
lysR	130	55	28.66667	1.064275	0.605792	-1.06464	1.064635117	0.090359059	-0.09036	0.777315	489	481	368	446	498	434	492	474.6667
lysS	2958	508	-1539.33	-1.36712	0.01055	-1.57633	1.576326266	0.656566172	-0.65657	0.047996	4588	6553	6056	5732.333	4746	3595	4238	4193
lytB	798	202	408.6667	1.215428	0.610947	1.072999	1.072999125	0.1016489	0.101649	0.781195	2085	2001	1605	1897	2403	2203	2311	2305.667
lyxK	31	15	18	1.55102	0.051868	1.364708	1.36470789	0.448592182	0.448592	0.160046	31	43	24	32.66667	55	46	51	50.66667
maa	1003	698	768.3333	1.863296	0.000128	1.655619	1.655619445	0.727371098	0.727371	0.001446	1050	945	675	890	1654	1678	1643	1658.333
maeB	5162	2115	-3299.67	-1.421	0.004143	-1.62705	1.627049555	0.702258192	-0.70226	0.02321	10136	12289	10987	11137.33	8365	7127	8021	7837.667
malE	32981	18188	-16959	-1.10958	0.169887	-1.26694	1.266942202	0.34135071	-0.34135	0.355254	177988	166781	170395	171721.3	168734	145007	150546	154762.3
malF	4105	1067	466.3333	1.019962	0.516118	-1.11767	1.11767132	0.160495989	-0.1605	0.715467	22294	25947	21842	23361	25015	23361	23106	23827.33
malG	2752	580	-294.333	-1.02412	0.361955	-1.16455	1.16455469	0.219778393	-0.21978	0.578532	12395	13923	11171	12496.33	12785	12006	11815	12202
malK	7033	2522	-4351.67	-1.14617	0.118672	-1.30517	1.305172101	0.384240054	-0.38424	0.280518	33686	36459	32222	34122.33	30186	29426	29700	29770.67
malM	7901	2638	-4080.33	-1.16632	0.095043	-1.33387	1.333869503	0.415617529	-0.41562	0.242311	26756	31008	28074	28612.67	26372	23107	24118	24532.33
malP	2732	779	-1435.33	-1.19038	0.066431	-1.36054	1.360539791	0.444179151	-0.44418	0.192264	8157	10026	8741	8974.667	7946	7378	7294	7539.333
malQ	1886	803	-1006	-1.12934	0.120596	-1.28808	1.288084152	0.36522685	-0.36523	0.28333	8651	9279	8421	8783.667	8322	7618	7393	7777.667
malS	433	330	343.3333	1.689886	0.001505	1.4952	1.495200275	0.58033874	0.580339	0.01055	600	456	437	497.6667	870	867	786	841
malT	3324	114	609	1.043513	0.61845	-1.08523	1.085231781	0.118003203	-0.118	0.785235	15455	14401	12131	13995.67	14894	14405	14515	14604.67
malX	30	10	4	1.073171	0.693702	-1.06839	1.068387888	0.095435526	-0.09544	0.837317	48	58	58	54.66667	68	39	69	58.66667
malY	11	3	-4.66667	-1.21538	0.106869	-1.38542	1.385424399	0.470327987	-0.47033	0.262201	20	28	31	26.33333	22	20	23	21.66667
malZ	92	35	-61	-1.17151	0.016478	-1.33935	1.339353402	0.421536681	-0.42154	0.067541	398	426	426	416.6667	370	334	363	355.6667

Feature ID	Experiment - Range (original values)	Experiment - IQR (original values)	Experiment - Difference (original values)	Experiment - Fold Change (original values)	EDGE test: yccT NT vs WT NT , tagwise dispersions - P-value	EDGE test: yccT NT vs WT NT , tagwise dispersions - Fold change	yccT NT vs WT NT ABS|FC|	yccT NT vs WT NT Log2FC	yccT NT vs WT NT Log2FC +-	EDGE test: yccT NT vs WT NT , tagwise dispersions - FDR p-value correction	WT NT - WT.1.S22 - Expression values	WT NT - WT.2.S23 - Expression values	WT NT - WT.3.S24 - Expression values	WT NT - Means	yccT NT - yccT.1.S28 - Expression values	yccT NT - yccT.2.S29 - Expression values	yccT NT - yccT.3.S30 - Expression values	yccT NT - Means
manA	451	35	-229.333	-1.17243	0.036633	-1.34213	1.342129526	0.424523909	-0.42452	0.124768	1351	1744	1583	1559.333	1366	1331	1293	1330
manC	25	15	7	1.132075	1	-1.0079	1.007902652	0.011356304	-0.01136	1	41	66	52	53	65	65	50	60
manX	13610	688	-6864	-1.55564	0.005497	-1.81226	1.812264384	0.85779344	-0.85779	0.02879	12620	19870	25162	19217.33	13098	11552	12410	12353.33
manY	11183	539	-6045	-1.69099	0.001727	-1.97122	1.971216963	0.979086576	-0.97909	0.011758	9358	15911	19111	14793.33	9428	7928	8889	8748.333
manZ	15333	830	-8245.67	-1.7867	0.00096	-2.08654	2.086539682	1.061112358	-1.06111	0.007444	11464	19929	24788	18727	11355	9455	10634	10481.33
map	515	195	242.3333	1.153993	0.906494	1.015566	1.015566145	0.022284207	0.022284	0.975632	1742	1547	1432	1573.667	1947	1680	1821	1816
marA	239	162	150	1.844278	0.000743	1.641007	1.64100659	0.714581032	0.714581	0.006028	258	143	132	177.6667	371	305	307	327.6667
marB	84	48	50.33333	1.415978	0.078455	1.252257	1.252256899	0.32453056	0.324531	0.215036	154	106	103	121	187	174	153	171.3333
marC	165	133	117.3333	1.465608	0.037793	1.296304	1.296304105	0.374404205	0.374404	0.127443	315	217	224	252	369	357	382	369.3333
marR	168	126	109.6667	1.90137	0.001454	1.698207	1.698206677	0.76401205	0.764012	0.010232	191	87	87	121.6667	255	226	213	231.3333
marT	173	133	129	1.809623	0.0003	1.606038	1.606038009	0.683506037	0.683506	0.002816	214	139	125	159.3333	298	272	295	288.3333
mazG	216	105	142.3333	1.23935	0.469798	1.091466	1.091466199	0.126267453	0.126267	0.676967	637	605	542	594.6667	743	710	758	737
mdaA	62	14	19.33333	1.069795	0.61389	-1.06142	1.061424627	0.086001928	-0.086	0.782388	294	288	249	277	298	280	311	296.3333
mdaB	117	47	40.66667	1.092917	0.753407	-1.03973	1.039727938	0.056206073	-0.05621	0.87967	437	487	389	437.6667	506	484	445	478.3333
mdfA	101	71	70.33333	1.371479	0.100563	1.21114	1.211139561	0.276365118	0.276365	0.252319	224	178	166	189.3333	263	249	267	259.6667
mdh	19461	3778	1772.333	1.060515	0.76413	-1.05783	1.057825067	0.081101068	-0.0811	0.884268	39177	28969	19716	29287.33	34903	27249	31027	31059.67
mdlA	142	53	76.66667	1.211009	0.599191	1.066762	1.066761563	0.093237748	0.093238	0.773381	367	407	316	363.3333	458	442	420	440
mdlB	70	37	21.66667	1.075231	0.628766	-1.05859	1.058588461	0.082141833	-0.08214	0.792621	310	288	266	288	336	273	320	309.6667
mdoB	1753	1410	1131.333	1.498824	0.082903	1.332926	1.332925858	0.414596535	0.414597	0.22214	3074	1891	1839	2268	3592	3305	3301	3399.333
mdoC	279	120	175.6667	1.357046	0.140215	1.199415	1.199415231	0.262331198	0.262331	0.311738	527	537	412	492	647	665	691	667.6667
mdoG	1852	34	-292.333	-1.05915	0.272797	-1.21869	1.218687212	0.285327892	-0.28533	0.485837	4048	5900	5755	5234.333	4957	4923	4946	4942
mdoH	1035	483	-31.3333	-1.00964	0.376638	-1.16178	1.161780351	0.216337335	-0.21634	0.594928	2660	3695	3489	3281.333	3427	2944	3379	3250
mdtI	295	117	193.3333	1.510563	0.034624	1.316223	1.316222571	0.396403467	0.396403	0.119484	371	365	400	378.6667	660	488	568	572
mdtJ	467	323	396.6667	1.543131	0.016494	1.352693	1.352693479	0.43583496	0.435835	0.067548	762	697	732	730.3333	1164	1055	1162	1127
melA	11716	10278	6905.667	1.829642	0.054392	1.669528	1.669527851	0.73944016	0.73944	0.16553	15903	4873	4195	8323.667	15911	15151	14626	15229.33
melB	6775	5800	3849.333	1.730378	0.064279	1.567261	1.567260533	0.648245025	0.648245	0.187506	9510	3076	3225	5270.333	9851	9025	8483	9119.667
melR	1420	508	-856.667	-1.54117	0.000147	-1.75172	1.751717255	0.808769928	-0.80877	0.001628	2295	2912	2112	2439.667	1653	1604	1492	1583
menA	46	7	18.66667	1.05668	0.525928	-1.07718	1.077181827	0.107261796	-0.10726	0.720144	334	341	313	329.3333	351	334	359	348
menB	4286	944	-3006	-2.32735	2.71E-06	-2.70612	2.706121158	1.436226433	-1.43623	6.17E-05	3187	6257	6368	5270.667	2469	2082	2243	2264.667
menC	4110	903	-2696.67	-2.45268	7.99E-07	-2.86099	2.860991932	1.516515429	-1.51652	2.23E-05	2772	5181	5706	4553	2104	1596	1869	1856.333
menD	3452	1122	-1990.67	-1.39768	0.005787	-1.59812	1.598116649	0.676372717	-0.67637	0.030027	6061	8266	6662	6996.333	5264	4814	4939	5005.667
menE	2555	589	-1674.33	-2.13437	2.55E-06	-2.47923	2.479232898	1.309893804	-1.30989	5.89E-05	2148	3453	3850	3150.333	1574	1295	1559	1476
menF	242	111	30.33333	1.043877	0.512126	-1.09323	1.093229923	0.128596854	-0.1286	0.712474	622	847	605	691.3333	792	640	733	721.6667
menG	2909	1819	-2170.67	-1.29485	0.016671	-1.47441	1.474413208	0.5601409	-0.56014	0.06808	9893	9618	9087	9532.667	7834	7268	6984	7362
mepA	170	86	91.33333	1.217116	0.550612	1.075367	1.075367399	0.104829641	0.10483	0.737684	494	408	360	420.6667	528	478	530	512
mesJ	245	106	61	1.081009	0.697935	-1.0499	1.049902464	0.070255307	-0.07026	0.840366	793	839	627	753	872	732	838	814

Feature ID	Experiment - Range (original values)	Experiment - IQR (original values)	Experiment - Difference (original values)	Experiment - Fold Change (original values)	EDGE test: yccT NT vs WT NT , tagwise dispersions - P-value	EDGE test: yccT NT vs WT NT , tagwise dispersions - Fold change	yccT NT vs WT NT ABS|FC|	yccT NT vs WT NT Log2FC	yccT NT vs WT NT Log2FC +-	EDGE test: yccT NT vs WT NT , tagwise dispersions - FDR p-value correction	WT NT - WT.1.S22 - Expression values	WT NT - WT.2.S23 - Expression values	WT NT - WT.3.S24 - Expression values	WT NT - Means	yccT NT - yccT.1.S28 - Expression values	yccT NT - yccT.2.S29 - Expression values	yccT NT - yccT.3.S30 - Expression values	yccT NT - Means
metA	124	66	54	1.234783	0.465154	1.09669	1.096690094	0.133155902	0.133156	0.673121	290	224	176	230	300	294	258	284
metB	50	27	0.333333	1.00128	0.274535	-1.14565	1.145652851	0.196169953	-0.19617	0.48756	237	264	280	260.3333	287	237	258	260.6667
metC	257	113	-166.667	-1.42626	0.000172	-1.62646	1.62645878	0.70173426	-0.70173	0.00185	505	641	527	557.6667	392	384	397	391
metE	260	48	-115.333	-1.3258	0.002674	-1.52112	1.521116773	0.60513091	-0.60513	0.016457	402	554	452	469.3333	408	294	360	354
metF	286	38	-164.667	-1.92857	9.61E-06	-2.24172	2.241718932	1.164605403	-1.16461	0.00018	229	379	418	342	191	132	209	177.3333
metG	390	136	285.3333	1.15129	0.960188	1.007258	1.007258454	0.010433914	0.010434	1	1863	1886	1909	1886	2253	2022	2239	2171.333
metH	525	74	182	1.081274	0.70867	-1.05793	1.057930385	0.081244697	-0.08124	0.847948	2310	2032	2376	2239.333	2557	2302	2405	2421.333
metJ	224	48	-67.6667	-1.07297	0.130382	-1.22788	1.227881423	0.296171246	-0.29617	0.297795	930	999	1056	995	978	972	832	927.3333
metK	1925	453	-1367	-3.01821	2.62E-09	-3.51002	3.510016686	1.811477889	-1.81148	1.41E-07	1109	2564	2460	2044.333	737	656	639	677.3333
metL	181	44	-62	-1.06917	0.133354	-1.22616	1.22615797	0.294144858	-0.29414	0.301168	841	1012	1022	958.3333	920	876	893	896.3333
metR	58	45	-50.6667	-1.93827	1.43E-09	-2.20673	2.206725436	1.141907138	-1.14191	8.14E-08	106	100	108	104.6667	57	50	55	54
mfd	1016	418	492.3333	1.204542	0.691058	1.060959	1.060958578	0.085368331	0.085368	0.835588	2734	2317	2170	2407	3186	2735	2777	2899.333
mglA	528	267	155.6667	1.174645	0.854431	1.030319	1.030318754	0.043090738	0.043091	0.947443	697	1225	752	891.3333	1019	975	1147	1047
mglB	2400	229	-849.333	-1.23365	0.04404	-1.40401	1.404011829	0.489555091	-0.48956	0.142686	4163	5607	3683	4484.333	3912	3207	3786	3635
mglC	357	123	32.33333	1.059255	0.664397	-1.07288	1.07288005	0.10148879	-0.10149	0.819355	472	761	404	545.6667	595	480	659	578
mgsA	1319	849	-964	-1.36878	0.004119	-1.56131	1.561307599	0.642754796	-0.64275	0.02313	3519	3730	3485	3578	2795	2636	2411	2614
mgtA	130	19	47.66667	1.122642	0.917732	-1.01355	1.013550359	0.019417772	-0.01942	0.982476	397	410	359	388.6667	429	391	489	436.3333
mgtB	129	15	56.33333	1.204848	0.613939	1.061599	1.06159853	0.086238278	0.086238	0.782388	303	288	234	275	363	301	330	331.3333
mgtC	25	8	10.33333	1.574074	0.121755	1.387129	1.387128513	0.472101455	0.472101	0.285297	24	16	14	18	25	21	39	28.33333
miaA	1096	1003	-934	-1.22393	0.034311	-1.39804	1.398038727	0.483404325	-0.4834	0.118683	5075	5094	5146	5105	4391	4050	4072	4171
miaB	489	172	255.6667	1.145873	0.966078	1.005985	1.005985258	0.008609164	0.008609	1	1835	1754	1669	1752.667	2158	1926	1941	2008.333
miaE	167	44	58	1.155357	0.864217	1.021887	1.021886691	0.031235237	0.031235	0.95281	429	398	293	373.3333	449	385	460	431.3333
mig-14	266	47	131	1.281317	0.462269	1.111419	1.11141861	0.152402303	0.152402	0.67026	382	489	526	465.6667	648	536	606	596.6667
mig-3	72	38	41.66667	1.420875	0.068777	1.256418	1.256418108	0.329316641	0.329317	0.19636	124	93	80	99	152	139	131	140.6667
mig-3A	42	13	20.33333	1.580952	0.046441	1.394788	1.39478845	0.480046323	0.480046	0.148684	46	33	26	35	57	41	68	55.33333
minC	553	128	304	1.119231	0.89009	-1.02192	1.021923653	0.031287418	-0.03129	0.967612	2357	2639	2653	2549.667	2884	2910	2767	2853.667
minD	1830	482	-197.667	-1.03182	0.322312	-1.18825	1.188252576	0.248841529	-0.24884	0.537713	5455	6488	7285	6409.333	6533	6006	6096	6211.667
minE	694	131	-262.333	-1.13488	0.083176	-1.30444	1.304438408	0.383428826	-0.38343	0.222288	2036	2070	2516	2207.333	2074	1939	1822	1945
mioC	278	62	-92	-1.07562	0.107084	-1.22764	1.227643923	0.295892169	-0.29589	0.262439	1268	1415	1243	1308.667	1307	1137	1206	1216.667
mipA	1319	824	-962.333	-1.16696	0.076935	-1.33011	1.330109141	0.41154463	-0.41154	0.21187	6826	6692	6661	6726.333	5948	5837	5507	5764
misL	92	29	40	1.085592	0.71392	-1.04561	1.045612584	0.064348409	-0.06435	0.850143	500	471	431	467.3333	522	523	477	507.3333
mlc	264	24	106.3333	1.115079	0.888954	-1.01775	1.017751272	0.025385024	-0.02539	0.966614	993	969	810	924	1074	974	1043	1030.333
mltA	225	75	121	1.230184	0.503086	1.084396	1.084396016	0.116891719	0.116892	0.704228	580	537	460	525.6667	685	612	643	646.6667
mltB	89	59	26.66667	1.067511	0.595447	-1.06717	1.067167049	0.093786026	-0.09379	0.771019	390	437	358	395	447	378	440	421.6667
mltC	177	40	61	1.086443	0.707237	-1.04842	1.048417251	0.068212997	-0.06821	0.846965	710	739	668	705.6667	756	699	845	766.6667
mntH	124	55	39.33333	1.176383	0.747683	1.042615	1.042615389	0.060207059	0.060207	0.876079	276	230	163	223	279	221	287	262.3333

Feature ID	Experiment - Range (original values)	Experiment - IQR (original values)	Experiment - Difference (original values)	Experiment - Fold Change (original values)	EDGE test: yccT NT vs WT NT , tagwise dispersions - P-value	EDGE test: yccT NT vs WT NT , tagwise dispersions - Fold change	yccT NT vs WT NT ABS|FC|	yccT NT vs WT NT Log2FC	yccT NT vs WT NT Log2FC +-	EDGE test: yccT NT vs WT NT , tagwise dispersions - FDR p-value correction	WT NT - WT.1.S22 - Expression values	WT NT - WT.2.S23 - Expression values	WT NT - WT.3.S24 - Expression values	WT NT - Means	yccT NT - yccT.1.S28 - Expression values	yccT NT - yccT.2.S29 - Expression values	yccT NT - yccT.3.S30 - Expression values	yccT NT - Means
moaA	2485	911	902	1.186531	0.745971	1.055354	1.055354026	0.077727043	0.077727	0.874907	6016	4960	3531	4835.667	5977	5365	5871	5737.667
moaB	497	53	-244	-1.26902	0.009689	-1.45018	1.450175231	0.536227238	-0.53623	0.045383	963	1364	1126	1151	910	944	867	907
moaC	347	26	-129	-1.21778	0.027264	-1.39218	1.392184305	0.477350215	-0.47735	0.099592	565	912	687	721.3333	579	593	605	592.3333
moaD	169	16	-65	-1.29018	0.009482	-1.47456	1.474563733	0.56028818	-0.56029	0.044699	221	381	265	289	223	212	237	224
moaE	241	17	-78	-1.21196	0.022014	-1.38399	1.383985213	0.468828529	-0.46883	0.083802	375	568	395	446	385	327	392	368
mobA	434	212	-284.333	-1.27209	0.00429	-1.44892	1.448923231	0.534981158	-0.53498	0.023649	1259	1462	1267	1329.333	1028	1060	1047	1045
mobB	343	101	-181.333	-1.20059	0.015896	-1.36869	1.368693139	0.452799031	-0.4528	0.065849	1017	1220	1019	1085.333	919	877	916	904
mod	221	146	161.3333	1.20302	0.670342	1.0546	1.054599865	0.076695717	0.076696	0.822338	777	835	772	794.6667	993	923	952	956
modA	695	74	-130.667	-1.0765	0.173622	-1.23801	1.238011154	0.308024313	-0.30802	0.361351	1423	2118	1975	1838.667	1754	1680	1690	1708
modB	327	131	-23.6667	-1.03313	0.26239	-1.1928	1.192803057	0.254355861	-0.25436	0.474024	537	864	813	738	765	634	744	714.3333
modC	650	175	-84.3333	-1.06387	0.198219	-1.22413	1.224131982	0.291759114	-0.29176	0.394568	1054	1704	1456	1404.667	1384	1209	1368	1320.333
modE	215	57	49.33333	1.051586	0.546719	-1.07818	1.078176337	0.108593152	-0.10859	0.73454	1021	1028	820	956.3333	1035	964	1018	1005.667
modF	424	90	-46	-1.03481	0.227771	-1.18157	1.181567256	0.240701751	-0.2407	0.433529	1238	1626	1238	1367.333	1434	1202	1328	1321.333
moeA	157	47	-45.6667	-1.05233	0.148239	-1.20503	1.20502606	0.269064347	-0.26906	0.32341	882	929	944	918.3333	929	787	902	872.6667
moeB	145	39	-52.6667	-1.12334	0.054178	-1.28837	1.288369092	0.365545956	-0.36555	0.164992	418	529	492	479.6667	457	384	440	427
mog	326	49	-51.6667	-1.03857	0.206436	-1.17621	1.176206428	0.23414128	-0.23414	0.40564	1542	1416	1216	1391.333	1366	1336	1317	1339.667
mopA	37454	10043	-26711.7	-2.37724	8.1E-07	-2.65665	2.656649848	1.40960809	-1.40961	2.24E-05	54107	55166	29047	46106.67	19004	21469	17712	19395
mopB	5555	2623	-3561	-2.36507	5.19E-08	-2.68109	2.681092981	1.422821253	-1.42282	2.03E-06	5431	7922	5156	6169.667	2533	2926	2367	2608.667
motA	5293	1183	2151.667	1.312002	0.377956	1.171105	1.171104669	0.227870024	0.22787	0.594928	8716	7533	4440	6896.333	9733	8627	8784	9048
motB	4076	649	1832.333	1.330349	0.325366	1.182133	1.182133453	0.241392913	0.241393	0.539305	6526	6239	3875	5546.667	7951	6888	7298	7379
mpl	119	32	-15.6667	-1.00826	0.308033	-1.15113	1.151134927	0.203056945	-0.20306	0.523149	1875	1952	1907	1911.333	1947	1833	1907	1895.667
mppA	1201	277	-738.333	-1.11137	0.146889	-1.26955	1.269548031	0.344314977	-0.34431	0.321415	6931	7664	7509	7368	6654	6772	6463	6629.667
mraY	1097	371	457.3333	1.237782	0.533648	1.093692	1.093692017	0.129206533	0.129207	0.725782	2286	1915	1569	1923.333	2666	2113	2363	2380.667
mrcA	282	132	163.3333	1.204678	0.620943	1.062188	1.062188341	0.087039599	0.08704	0.787112	905	774	715	798	997	906	981	961.3333
mrcB	529	74	219	1.091568	0.770207	-1.04391	1.043905043	0.061990485	-0.06199	0.887872	2491	2421	2263	2391.667	2792	2417	2623	2610.667
mrdA	228	71	137	1.180739	0.781566	1.035773	1.035772988	0.05070784	0.050708	0.895326	750	810	714	758	922	821	942	895
mrdB	57	11	21.33333	1.082474	0.661107	-1.05532	1.055323682	0.07768556	-0.07769	0.817117	243	272	261	258.6667	300	277	263	280
mreB	1221	174	-304.333	-1.15219	0.113012	-1.33569	1.335694142	0.417589686	-0.41759	0.272151	1724	2243	2945	2304	1983	1921	2095	1999.667
mreC	446	123	-129.333	-1.17905	0.060732	-1.36788	1.367875226	0.451936638	-0.45194	0.179521	617	875	1063	851.6667	750	647	770	722.3333
mreD	74	24	-12	-1.05732	0.152127	-1.20902	1.209023919	0.273842787	-0.27384	0.328657	207	231	226	221.3333	202	176	250	209.3333
mrp	1923	597	-1160	-1.34901	0.008187	-1.54487	1.544869515	0.627484988	-0.62748	0.039646	3919	5077	4455	4483.667	3495	3154	3322	3323.667
mrr	91	16	-27.3333	-1.14138	0.038368	-1.29972	1.299718688	0.378199399	-0.3782	0.128501	207	264	191	220.6667	209	173	198	193.3333
mrsA	255	86	-59.6667	-1.02173	0.312254	-1.16545	1.165453316	0.220891216	-0.22089	0.527084	2898	2740	2779	2805.667	2900	2645	2693	2746
msbA	484	128	260.3333	1.19227	0.712408	1.048204	1.048204059	0.0679196	0.06792	0.849483	1410	1406	1246	1354	1730	1579	1534	1614.333
msbB	534	331	361.6667	1.390709	0.104033	1.228383	1.228383424	0.29676095	0.296761	0.257959	1113	853	811	925.6667	1345	1184	1333	1287.333
mscL	106	38	-40.3333	-1.07401	0.097513	-1.22142	1.221424979	0.288565255	-0.28857	0.24662	605	619	532	585.3333	570	513	552	545

Feature ID	Experiment - Range (original values)	Experiment - IQR (original values)	Experiment - Difference (original values)	Experiment - Fold Change (original values)	EDGE test: yccT NT vs WT NT , tagwise dispersions - P-value	EDGE test: yccT NT vs WT NT , tagwise dispersions - Fold change	yccT NT vs WT NT ABS|FC|	yccT NT vs WT NT Log2FC	yccT NT vs WT NT Log2FC +-	EDGE test: yccT NT vs WT NT , tagwise dispersions - FDR p-value correction	WT NT - WT.1.S22 - Expression values	WT NT - WT.2.S23 - Expression values	WT NT - WT.3.S24 - Expression values	WT NT - Means	yccT NT - yccT.1.S28 - Expression values	yccT NT - yccT.2.S29 - Expression values	yccT NT - yccT.3.S30 - Expression values	yccT NT - Means
msgA	96	52	66.33333	1.529255	0.010875	1.348177	1.348177375	0.431010319	0.43101	0.049154	146	122	108	125.3333	204	174	197	191.6667
msrA	321	129	123.3333	1.168029	0.7805	1.03649	1.036490229	0.051706516	0.051707	0.894799	876	747	579	734	900	887	785	857.3333
msyB	115	68	67.33333	1.381132	0.105504	1.223772	1.223772323	0.291335176	0.291335	0.260004	225	161	144	176.6667	259	244	229	244
mtgA	295	35	-59.6667	-1.04335	0.185136	-1.19612	1.196116616	0.258358053	-0.25836	0.374071	1418	1383	1507	1436	1518	1223	1388	1376.333
mtlA	463	152	213.3333	1.075427	0.710226	-1.05737	1.05737467	0.080486671	-0.08049	0.847948	2996	2844	2645	2828.333	3106	2911	3108	3041.667
mtlD	762	546	542	1.250539	0.515498	1.100015	1.100015351	0.137523657	0.137524	0.715369	2353	2090	2047	2163.333	2809	2636	2671	2705.333
mtlR	241	86	138	1.246429	0.482049	1.091284	1.091283769	0.126026298	0.126026	0.687466	565	568	547	560	788	651	655	698
mtr	21	5	4.666667	1.038674	0.448538	-1.09703	1.097028239	0.133600663	-0.1336	0.658687	124	119	119	120.6667	134	113	129	125.3333
mug	124	86	51.66667	1.12224	0.950134	-1.00852	1.00851634	0.012234459	-0.01223	1	501	390	377	422.6667	481	466	476	474.3333
mukB	1446	869	-857.333	-1.13844	0.098837	-1.30106	1.301063527	0.379691406	-0.37969	0.248974	7081	7132	6938	7050.333	6824	5686	6069	6193
mukE	138	65	-3.66667	-1.00369	0.299375	-1.14699	1.146991203	0.197854327	-0.19785	0.515357	920	1058	1013	997	1017	1014	949	993.3333
mukF	193	107	-9	-1.00618	0.292052	-1.15187	1.151868977	0.203976623	-0.20398	0.50842	1372	1533	1493	1466	1565	1386	1420	1457
murA	204	50	18.66667	1.016442	0.370439	-1.12487	1.124874152	0.169763605	-0.16976	0.587688	1032	1236	1138	1135.333	1172	1120	1170	1154
murB	200	41	-77.6667	-1.06347	0.130765	-1.21174	1.211744397	0.277085412	-0.27709	0.298174	1295	1352	1257	1301.333	1280	1239	1152	1223.667
murC	1703	616	249.6667	1.061373	0.698983	-1.0639	1.063901892	0.089365119	-0.08937	0.841172	4955	3997	3252	4068	4617	3860	4476	4317.667
murD	1437	548	402.6667	1.134912	0.96916	1.006263	1.006263064	0.009007514	0.009008	1	3740	2911	2303	2984.667	3718	2985	3459	3387.333
murE	1356	363	421.6667	1.121296	0.94795	-1.01022	1.010215352	0.014662871	-0.01466	0.999617	3964	3572	2893	3476.333	4249	3541	3904	3898
murF	1243	569	487.3333	1.184946	0.760341	1.048235	1.048234928	0.067962086	0.067962	0.882187	3170	2587	2148	2635	3391	2820	3156	3122.333
murG	1082	341	169	1.082546	0.794095	-1.03979	1.039788713	0.056290399	-0.05629	0.903705	2663	1898	1581	2047.333	2380	2030	2239	2216.333
murI	183	86	-0.33333	-1.0002	0.324294	-1.13804	1.138036552	0.186546896	-0.18655	0.539103	1779	1734	1597	1703.333	1780	1648	1681	1703
mutH	158	93	86.33333	1.225806	0.514347	1.083084	1.083084179	0.115145376	0.115145	0.714667	455	362	330	382.3333	482	436	488	468.6667
mutL	540	163	-113.333	-1.04124	0.254662	-1.18914	1.189135197	0.249912749	-0.24991	0.466842	2894	2930	2761	2861.667	3027	2487	2731	2748.333
mutM	170	68	-124.667	-1.64151	3.66E-06	-1.88664	1.88663623	0.915816278	-0.91582	7.99E-05	262	349	346	319	210	179	194	194.3333
mutS	816	323	357.3333	1.1497	0.918288	1.015325	1.015325235	0.021941934	0.021942	0.982488	2717	2394	2050	2387	2866	2611	2756	2744.333
mutT	40	21	-8	-1.11823	0.130817	-1.28561	1.285609894	0.362452937	-0.36245	0.298174	59	88	80	75.66667	80	48	75	67.66667
mutY	89	28	-14.6667	-1.02324	0.21187	-1.16594	1.16594208	0.221496122	-0.2215	0.413012	637	687	613	645.6667	641	598	654	631
mviM	1009	219	213.3333	1.063098	0.679491	-1.06523	1.065233495	0.091169698	-0.09117	0.827568	3829	3475	2839	3381	3848	3358	3577	3594.333
mviN	698	274	399.3333	1.401744	0.092715	1.242042	1.2420416	0.312713495	0.312713	0.238952	1196	1007	779	994	1477	1281	1422	1393.333
nadA	60	53	42.33333	1.395639	0.087421	1.234804	1.234803603	0.304281597	0.304282	0.229842	135	95	91	107	149	151	148	149.3333
nadB	86	36	25.33333	1.085202	0.694648	-1.04863	1.04863483	0.06851237	-0.06851	0.837547	313	308	271	297.3333	334	277	357	322.6667
nadC	252	53	51.33333	1.063927	0.620798	-1.06345	1.063448263	0.088749847	-0.08875	0.787112	928	805	676	803	894	811	858	854.3333
nadD	118	61	-11.3333	-1.02346	0.221914	-1.17136	1.171361775	0.22818672	-0.22819	0.42604	442	554	487	494.3333	510	436	503	483
nadE	544	281	-127	-1.04355	0.26116	-1.18516	1.185156525	0.24507761	-0.24508	0.472906	3272	3069	2788	3043	3090	2930	2728	2916
nadR	583	199	271.6667	1.136998	0.988692	1.002149	1.002148822	0.003096768	0.003097	1	2017	2216	1716	1983	2260	2205	2299	2254.667
nagA	543	122	-209.333	-1.11582	0.086459	-1.26785	1.267846668	0.342380278	-0.34238	0.228443	2017	2276	1757	2016.667	1879	1733	1810	1807.333
nagB	633	147	-338.333	-1.22367	0.015478	-1.39176	1.391757174	0.476907519	-0.47691	0.064517	1780	2100	1673	1851	1545	1526	1467	1512.667

Feature ID	Experiment - Range (original values)	Experiment - IQR (original values)	Experiment - Difference (original values)	Experiment - Fold Change (original values)	EDGE test: yccT NT vs WT NT , tagwise dispersions - P-value	EDGE test: yccT NT vs WT NT , tagwise dispersions - Fold change	yccT NT vs WT NT ABS|FC|	yccT NT vs WT NT Log2FC	yccT NT vs WT NT Log2FC +-	EDGE test: yccT NT vs WT NT , tagwise dispersions - FDR p-value correction	WT NT - WT.1.S22 - Expression values	WT NT - WT.2.S23 - Expression values	WT NT - WT.3.S24 - Expression values	WT NT - Means	yccT NT - yccT.1.S28 - Expression values	yccT NT - yccT.2.S29 - Expression values	yccT NT - yccT.3.S30 - Expression values	yccT NT - Means
nagC	332	75	36.66667	1.021388	0.418553	-1.11399	1.113994843	0.155742554	-0.15574	0.631894	1754	1834	1555	1714.333	1887	1679	1687	1751
nagD	122	85	15	1.017794	0.356745	-1.12207	1.122069398	0.166161906	-0.16616	0.572109	802	913	814	843	896	791	887	858
nagE	4634	2762	-3226	-1.42533	0.003599	-1.62709	1.627093598	0.702297244	-0.7023	0.020873	10245	11787	10400	10810.67	8118	7483	7153	7584.667
nanA	34289	7017	-19405.3	-4.07858	4.92E-10	-4.56261	4.562611678	2.189859872	-2.18986	3.03E-08	24333	39269	13524	25708.67	6507	7423	4980	6303.333
nanE	6125	426	-2917	-1.85176	0.000268	-2.08656	2.086562846	1.061128374	-1.06113	0.002563	5888	9227	3910	6341.667	3688	3484	3102	3424.667
nanK	4623	681	-2171.33	-1.95724	9.56E-05	-2.21551	2.215508684	1.147637982	-1.14764	0.001144	3713	6727	2879	4439.667	2503	2198	2104	2268.333
nanT	17318	1691	-9616.33	-2.45651	4.19E-06	-2.7481	2.748099408	1.458434192	-1.45843	8.89E-05	16892	23150	8614	16218.67	6923	7052	5832	6602.333
napA	26291	4958	-15482.3	-19.8121	0	-22.9506	22.95063224	4.520461992	-4.52046	0	5790	27043	16083	16305.33	832	752	885	823
napB	3296	620	-2270.33	-29.0288	3.22E-24	-34.0477	34.04766873	5.089484114	-5.08948	7.92E-22	705	3361	2988	2351.333	85	65	93	81
napC	4440	772	-3170.67	-26.7081	9.36E-22	-31.4269	31.42689874	4.973928006	-4.97393	1.89E-19	893	4431	4558	3294	131	118	121	123.3333
napD	1208	226	-641	-14.3542	5.34E-19	-16.35	16.34998387	4.031217307	-4.03122	8.76E-17	278	1246	543	689	38	54	52	48
napF	3325	607	-1854.67	-15.1939	1.1E-20	-17.4819	17.48186577	4.127787261	-4.12779	1.87E-18	738	3452	1766	1985.333	127	131	134	130.6667
napG	3623	762	-2207.67	-25.5296	2.02E-27	-29.6024	29.602362	4.887640389	-4.88764	5.59E-25	848	3706	2339	2297.667	101	83	86	90
napH	3715	676	-2268	-23.4554	3E-23	-27.2504	27.25042458	4.768206803	-4.76821	6.33E-21	773	3800	2534	2369	97	85	121	101
narG	679	102	-262.667	-1.18148	0.032051	-1.34636	1.346357889	0.429061958	-0.42906	0.112356	1584	2035	1511	1710	1544	1356	1442	1447.333
narH	513	88	-175	-1.2769	0.015496	-1.46064	1.460641441	0.546602068	-0.5466	0.064531	628	1091	702	807	704	578	614	632
narI	252	54	-92	-1.29838	0.008772	-1.48426	1.484255747	0.5697397	-0.56974	0.041928	322	523	356	400.3333	354	300	271	308.3333
narJ	230	36	-90.3333	-1.30381	0.006912	-1.48853	1.488534693	0.573892846	-0.57389	0.034556	313	510	340	387.6667	324	288	280	297.3333
narK	72	15	-24.3333	-1.48993	0.015837	-1.67872	1.678717234	0.747359241	-0.74736	0.065707	59	112	51	74	44	65	40	49.66667
narL	708	131	-434	-2.57246	3.17E-08	-2.96693	2.966929179	1.56897049	-1.56897	1.37E-06	407	980	743	710	280	272	276	276
narP	369	55	-41	-1.04457	0.22345	-1.20652	1.206520607	0.270852557	-0.27085	0.427692	832	850	1201	961	955	900	905	920
narQ	419	283	287	1.447273	0.044958	1.280337	1.280337103	0.356523711	0.356524	0.144967	760	622	543	641.6667	962	905	919	928.6667
narU	85	39	51.66667	1.598456	0.009565	1.41937	1.419369664	0.505250377	0.50525	0.044944	109	88	62	86.33333	127	140	147	138
narV	66	53	44	1.566524	0.020182	1.391083	1.39108266	0.476208149	0.476208	0.078652	110	65	58	77.66667	123	118	124	121.6667
narW	58	30	30.66667	1.70229	0.018767	1.512776	1.512776138	0.597198512	0.597199	0.074342	64	33	34	43.66667	61	71	91	74.33333
narX	235	35	-99.3333	-1.17447	0.028412	-1.33723	1.337229882	0.419247499	-0.41925	0.103107	603	790	613	668.6667	568	585	555	569.3333
narY	112	48	53.33333	1.487805	0.048522	1.318538	1.318538298	0.398939476	0.398939	0.152954	149	101	78	109.3333	190	137	161	162.6667
narZ	204	102	125	1.661376	0.001344	1.468212	1.468212192	0.554060488	0.55406	0.009591	227	189	151	189	355	296	291	314
ndh	435	231	-319	-1.37544	0.0005	-1.56457	1.564566574	0.645763048	-0.64576	0.004371	1181	1215	1110	1168.667	879	890	780	849.6667
ndk	856	78	-408	-1.39256	0.000905	-1.58155	1.581546792	0.66133624	-0.66134	0.00711	1385	1795	1162	1447.333	1095	939	1084	1039.333
nei	131	75	45.33333	1.046848	0.493594	-1.09082	1.090820659	0.125413929	-0.12541	0.697625	943	1018	942	967.6667	1073	943	1023	1013
nemA	731	369	-453.333	-1.45258	0.000103	-1.65866	1.658661017	0.730019071	-0.73002	0.001211	1384	1641	1340	1455	1124	910	971	1001.667
nfi	309	160	-182.333	-1.1514	0.037	-1.31307	1.313065875	0.392939297	-0.39294	0.125727	1374	1464	1322	1386.667	1296	1162	1155	1204.333
nfnB	941	506	-730.667	-1.44817	0.000562	-1.65982	1.659822331	0.731028822	-0.73103	0.004806	2121	2507	2455	2361	1710	1566	1615	1630.333
nfo	121	6	48.33333	1.05793	0.537826	-1.07922	1.079222508	0.109992342	-0.10999	0.727856	848	813	842	834.3333	934	846	868	882.6667
nhaA	211	67	-9.66667	-1.01213	0.273695	-1.14557	1.145568287	0.19606346	-0.19606	0.487114	895	840	684	806.3333	795	764	831	796.6667

Feature ID	Experiment - Range (original values)	Experiment - IQR (original values)	Experiment - Difference (original values)	Experiment - Fold Change (original values)	EDGE test: yccT NT vs WT NT , tagwise dispersions - P-value	EDGE test: yccT NT vs WT NT , tagwise dispersions - Fold change	yccT NT vs WT NT ABS|FC|	yccT NT vs WT NT Log2FC	yccT NT vs WT NT Log2FC +-	EDGE test: yccT NT vs WT NT , tagwise dispersions - FDR p-value correction	WT NT - WT.1.S22 - Expression values	WT NT - WT.2.S23 - Expression values	WT NT - WT.3.S24 - Expression values	WT NT - Means	yccT NT - yccT.1.S28 - Expression values	yccT NT - yccT.2.S29 - Expression values	yccT NT - yccT.3.S30 - Expression values	yccT NT - Means
nhaB	252	98	-5	-1.00433	0.300396	-1.15239	1.152387034	0.204625334	-0.20463	0.515535	1129	1049	1301	1159.667	1182	1084	1198	1154.667
nhaR	145	24	33.66667	1.068801	0.634737	-1.06016	1.060159893	0.084281868	-0.08428	0.797616	517	543	408	489.3333	553	520	496	523
nhoA	235	103	129	1.306413	0.246148	1.149668	1.149668371	0.201217767	0.201218	0.457309	470	413	380	421	615	516	519	550
nifJ	177	62	26.66667	1.038095	0.479717	-1.09175	1.091753934	0.126647729	-0.12665	0.685465	745	762	593	700	727	683	770	726.6667
nifS	740	175	368	1.137194	0.962003	-1.00752	1.007521336	0.01081039	-0.01081	1	2596	2663	2788	2682.333	3336	2838	2977	3050.333
nifU	270	55	125.3333	1.099947	0.768738	-1.04082	1.040816403	0.057715603	-0.05772	0.887283	1177	1268	1317	1254	1447	1368	1323	1379.333
nikR	69	18	17.66667	1.035787	0.425951	-1.10075	1.100753403	0.138491305	-0.13849	0.638279	519	469	493	493.6667	538	489	507	511.3333
nirB	366	16	-138.667	-1.42579	0.004066	-1.63037	1.630368688	0.705198249	-0.7052	0.022865	331	667	395	464.3333	346	301	330	325.6667
nirC	159	13	-81.6667	-2.28947	9.7E-06	-2.64682	2.646818009	1.404259001	-1.40426	0.000181	77	210	148	145	75	51	64	63.33333
nirD	271	41	-137.333	-2.41096	3.32E-07	-2.76397	2.763969142	1.466741509	-1.46674	1.04E-05	143	357	204	234.6667	104	86	102	97.33333
nlp	32	2	10	1.080645	0.675784	-1.05786	1.05785835	0.08114646	-0.08115	0.825131	110	128	134	124	130	130	142	134
nlpB	991	234	-322.667	-1.05365	0.248759	-1.20641	1.206406973	0.270716673	-0.27072	0.459846	6234	6218	6559	6337	6475	5568	6000	6014.333
nlpC	122	56	81.33333	1.392283	0.088501	1.223855	1.223854542	0.291432101	0.291432	0.231765	235	188	199	207.3333	301	255	310	288.6667
nlpD	7070	2289	2851.667	1.262633	0.505825	1.124039	1.12403911	0.168692234	0.168692	0.706593	13659	11267	7648	10858	14718	12855	13556	13709.67
nlpI	10473	4002	4856	1.194484	0.749294	1.055369	1.055368921	0.077747404	0.077747	0.876484	28808	24806	21292	24968.67	31765	28671	29038	29824.67
nmpC	120533	69584	-90659.7	-1.83141	4.98E-05	-2.10894	2.108943865	1.076520695	-1.07652	0.000689	177615	205685	215808	199702.7	123823	95275	108031	109043
nrdA	886	70	116.6667	1.043435	0.594548	-1.08355	1.083554645	0.115771912	-0.11577	0.770531	3105	2734	2219	2686	2968	2685	2755	2802.667
nrdB	226	21	-3.66667	-1.00281	0.310238	-1.14321	1.143208706	0.193088808	-0.19309	0.525577	1264	1442	1216	1307.333	1404	1264	1243	1303.667
nrdD	1280	329	-733.333	-1.27027	0.021276	-1.45439	1.454387779	0.540411982	-0.54041	0.081693	2984	3923	3433	3446.667	2842	2643	2655	2713.333
nrdE	137	78	79	1.33617	0.166838	1.186942	1.186942297	0.2472498	0.24725	0.351527	292	234	179	235	312	316	314	314
nrdF	67	30	39	1.473684	0.042121	1.307435	1.307434558	0.386738736	0.386739	0.138271	103	81	63	82.33333	111	130	123	121.3333
nrdG	87	11	24.66667	1.039278	0.459023	-1.09668	1.09668259	0.133146031	-0.13315	0.667738	663	583	638	628	670	649	639	652.6667
nrdH	3	0	0.333333	1.5	1	1.271688	1.271688242	0.346745033	0.346745	1	0	2	0	0.666667	3	0	0	1
nrdI	8	3	2.333333	1.636364	0.367639	1.43086	1.430859893	0.516882413	0.516882	0.584556	5	6	0	3.666667	8	7	3	6
nrfA	9320	1102	-5522	-2.61635	4.55E-06	-3.0305	3.030501894	1.599556744	-1.59956	9.38E-05	4460	12558	9797	8938.333	3358	3238	3653	3416.333
nrfB	2652	233	-1558.33	-2.79394	5.66E-07	-3.24303	3.243031613	1.697343087	-1.69734	1.67E-05	1129	3461	2691	2427	896	809	901	868.6667
nrfC	2465	287	-1449	-3.1236	6.02E-08	-3.62885	3.628850965	1.859512807	-1.85951	2.3E-06	981	3054	2359	2131.333	694	589	764	682.3333
nrfD	1785	203	-1152.33	-3.7393	4.56E-09	-4.37017	4.370168264	2.127688829	-2.12769	2.35E-07	653	2146	1920	1573	451	361	450	420.6667
nrfG	307	8	-152	-2.92405	1.43E-05	-3.4589	3.458903564	1.790314791	-1.79031	0.000248	85	230	378	231	79	71	87	79
nth	150	97	75.66667	1.367314	0.158372	1.210885	1.210885269	0.276062177	0.276062	0.338117	275	165	178	206	281	249	315	281.6667
ntpA	236	16	-83	-1.07268	0.116718	-1.22902	1.229019967	0.297508355	-0.29751	0.277604	1178	1185	1312	1225	1183	1076	1167	1142
nuoA	943	311	393	1.120306	0.92136	-1.01538	1.015378858	0.022018126	-0.02202	0.983746	3295	3606	2899	3266.667	3842	3386	3751	3659.667
nuoB	1445	467	-178	-1.03908	0.301711	-1.18643	1.18643258	0.24663012	-0.24663	0.516769	4218	5650	4330	4732.667	4774	4205	4685	4554.667
nuoC	3773	681	-1362	-1.21476	0.060044	-1.3948	1.394795276	0.480053383	-0.48005	0.177942	6286	9317	7509	7704	6967	5544	6515	6342
nuoE	1448	191	-749.333	-1.57172	0.000249	-1.80596	1.805964984	0.85276992	-0.85277	0.002399	1585	2578	2017	2060	1394	1130	1408	1310.667
nuoF	3010	439	-1543.67	-1.5498	0.001355	-1.78574	1.78574046	0.836522414	-0.83652	0.009637	3348	5334	4372	4351.333	3190	2324	2909	2807.667

Feature ID	Experiment - Range (original values)	Experiment - IQR (original values)	Experiment - Difference (original values)	Experiment - Fold Change (original values)	EDGE test: yccT NT vs WT NT , tagwise dispersions - P-value	EDGE test: yccT NT vs WT NT , tagwise dispersions - Fold change	yccT NT vs WT NT ABS|FC|	yccT NT vs WT NT Log2FC	yccT NT vs WT NT Log2FC +-	EDGE test: yccT NT vs WT NT , tagwise dispersions - FDR p-value correction	WT NT - WT.1.S22 - Expression values	WT NT - WT.2.S23 - Expression values	WT NT - WT.3.S24 - Expression values	WT NT - Means	yccT NT - yccT.1.S28 - Expression values	yccT NT - yccT.2.S29 - Expression values	yccT NT - yccT.3.S30 - Expression values	yccT NT - Means
nuoG	7086	1683	-3443.33	-1.53853	0.001765	-1.76236	1.762362631	0.81751081	-0.81751	0.011961	8288	12417	8807	9837.333	7246	5331	6605	6394
nuoH	3315	943	-1719.33	-1.68755	0.000149	-1.92648	1.926478212	0.94596587	-0.94597	0.001635	3800	5375	3485	4220	2900	2060	2542	2500.667
nuoI	1925	315	-921.333	-1.65935	9.66E-05	-1.88919	1.889190369	0.917768086	-0.91777	0.001153	2116	3062	1778	2318.667	1592	1137	1463	1397.333
nuoJ	2132	436	-1162.33	-1.79214	8.91E-06	-2.0347	2.034703912	1.02481887	-1.02482	0.000169	2541	3369	1979	2629.667	1622	1237	1543	1467.333
nuoK	782	183	-424	-1.84294	7.32E-07	-2.09426	2.094259902	1.066440495	-1.06644	2.09E-05	904	1191	686	927	597	409	503	503
nuoL	7963	1641	-4428.67	-1.99208	1.05E-05	-2.25655	2.256554752	1.174121784	-1.17412	0.00019	8734	11769	6175	8892.667	5052	3806	4534	4464
nuoM	4967	1307	-3018.67	-2.09956	1.17E-06	-2.37781	2.377814918	1.249636425	-1.24964	3.11E-05	5984	7261	4047	5764	3202	2294	2740	2745.333
nuoN	5199	1982	-3414.67	-2.46301	3.73E-09	-2.79525	2.795246869	1.482975704	-1.48298	1.94E-07	5698	7208	4340	5748.667	2635	2009	2358	2334
nupC	1788	61	-662.333	-1.57561	0.002475	-1.79667	1.796672181	0.8453272	-0.84533	0.015402	1268	2817	1354	1813	1216	1029	1207	1150.667
nupG	5195	190	-2157.33	-2.79978	4.69E-05	-3.14816	3.148162824	1.654510159	-1.65451	0.000658	2354	6348	1366	3356	1267	1153	1176	1198.667
nusA	2152	759	-805	-1.14539	0.110903	-1.32131	1.321308202	0.401967022	-0.40197	0.268238	5419	6417	7190	6342	6166	5038	5407	5537
nusB	281	149	-159	-1.16071	0.026265	-1.32694	1.326944253	0.408107762	-0.40811	0.096261	1150	1167	1128	1148.333	1103	886	979	989.3333
nusG	350	166	63.33333	1.026283	0.471088	-1.1167	1.116704077	0.159246926	-0.15925	0.677962	2356	2313	2560	2409.667	2645	2295	2479	2473
nxiA	176	134	143	1.858	2.62E-05	1.640902	1.64090155	0.714488683	0.714489	0.000415	199	158	143	166.6667	318	292	319	309.6667
oat	12	6	0.333333	1.003745	0.324935	-1.13153	1.131534823	0.178280985	-0.17828	0.539103	91	94	82	89	91	92	85	89.33333
ogt	254	136	133.3333	1.232964	0.473742	1.0934	1.093399936	0.128821196	0.128821	0.679776	692	562	463	572.3333	717	702	698	705.6667
ompA	51687	15940	-20530	-1.18709	0.087992	-1.34891	1.348907095	0.431790987	-0.43179	0.230881	135328	147970	107497	130265	123437	96283	109485	109735
ompC	30354	14822	-6569	-1.09707	0.217174	-1.24163	1.241627617	0.312232552	-0.31223	0.420585	88856	74343	59521	74240	77655	58502	66856	67671
ompF	5936	1693	-4047.67	-2.61756	7.39E-08	-3.0278	3.027804499	1.598272056	-1.59827	2.78E-06	4180	8266	7204	6550	2690	2330	2487	2502.333
ompN	264	189	133.3333	1.547945	0.050852	1.381629	1.381628657	0.466369912	0.46637	0.158038	375	186	169	243.3333	433	319	378	376.6667
ompR	336	64	-126.333	-1.04101	0.264079	-1.18449	1.184489871	0.244265861	-0.24427	0.475665	3341	3225	3055	3207	3119	3005	3118	3080.667
ompS	174	87	95.66667	1.888545	0.008645	1.706405	1.706404729	0.770959869	0.77096	0.041455	179	96	48	107.6667	205	183	222	203.3333
ompW	23713	4709	7870	1.177479	0.903793	1.02315	1.023150257	0.033018031	0.033018	0.973907	46047	34709	52274	44343.33	58422	47462	50756	52213.33
ompX	13343	238	4050.667	1.21359	0.673351	1.085936	1.085936037	0.118939129	0.118939	0.824432	23557	22284	11053	18964.67	24396	22444	22206	23015.33
opgD	514	324	400	1.599401	0.008424	1.40045	1.40044967	0.485890136	0.48589	0.040619	687	616	699	667.3333	1061	1011	1130	1067.333
oppA	48050	32478	25692.33	1.471122	0.155396	1.312922	1.312921529	0.392780691	0.392781	0.33296	78119	45451	40033	54534.33	88083	74668	77929	80226.67
oppB	4916	2850	2583.667	1.339539	0.308173	1.18762	1.187619625	0.248072839	0.248073	0.523185	9744	6894	6190	7609.333	11106	9425	10048	10193
oppC	3073	1158	1564.333	1.288339	0.416894	1.139669	1.139668936	0.188614795	0.188615	0.630463	6489	5331	4456	5425.333	7529	6398	7042	6989.667
oppD	3232	1211	1628.667	1.221376	0.670828	1.071982	1.07198185	0.100280479	0.10028	0.822706	8306	6670	7095	7357	9902	7948	9107	8985.667
oppF	2823	1375	1348.333	1.242579	0.588263	1.09277	1.092770121	0.127989943	0.12799	0.76537	6589	4872	5214	5558.333	7695	6061	6964	6906.667
orf242	309	205	203	1.452452	0.054591	1.288094	1.28809412	0.365238014	0.365238	0.165784	578	401	367	448.6667	673	606	676	651.6667
orf245	225	187	169.6667	2.275689	4.11E-05	2.029159	2.029159322	1.020882145	1.020882	0.000593	208	82	109	133	307	296	305	302.6667
orf319	3492	1851	1261	1.314699	0.383927	1.181613	1.181612964	0.24075756	0.240758	0.600699	6070	3373	2578	4007	5554	5224	5026	5268
orf32	139	74	91.33333	1.564948	0.007042	1.383525	1.383524509	0.468348201	0.468348	0.035036	195	157	133	161.6667	256	231	272	253
orf408	128	77	100.3333	2.140152	6.25E-07	1.878042	1.878042493	0.909229706	0.90923	1.82E-05	99	74	91	88	195	168	202	188.3333
orf48	113	91	63.33333	1.274964	0.32933	1.128807	1.128807166	0.174799052	0.174799	0.543082	296	197	198	230.3333	310	289	282	293.6667

Feature ID	Experiment - Range (original values)	Experiment - IQR (original values)	Experiment - Difference (original values)	Experiment - Fold Change (original values)	EDGE test: yccT NT vs WT NT , tagwise dispersions - P-value	EDGE test: yccT NT vs WT NT , tagwise dispersions - Fold change	yccT NT vs WT NT ABS|FC|	yccT NT vs WT NT Log2FC	yccT NT vs WT NT Log2FC +-	EDGE test: yccT NT vs WT NT , tagwise dispersions - FDR p-value correction	WT NT - WT.1.S22 - Expression values	WT NT - WT.2.S23 - Expression values	WT NT - WT.3.S24 - Expression values	WT NT - Means	yccT NT - yccT.1.S28 - Expression values	yccT NT - yccT.2.S29 - Expression values	yccT NT - yccT.3.S30 - Expression values	yccT NT - Means
orf70	45	21	-15.6667	-1.04083	0.160671	-1.18265	1.182651823	0.242025402	-0.24203	0.341618	414	413	371	399.3333	392	390	369	383.6667
orfB	255	164	128	1.189817	0.681582	1.053332	1.053332146	0.074960431	0.07496	0.829012	801	637	585	674.3333	820	840	747	802.3333
orfX	164	46	90.33333	1.293926	0.369567	1.131016	1.131015719	0.17761898	0.177619	0.586979	275	313	334	307.3333	359	395	439	397.6667
orgA	138	91	109.3333	1.970414	5.61E-06	1.731272	1.731271771	0.791832213	0.791832	0.000114	129	98	111	112.6667	236	202	228	222
orn	207	122	-144	-1.18462	0.017539	-1.35477	1.35477075	0.438048744	-0.43805	0.070714	892	917	963	924	814	770	756	780
osmC	129	86	59.33333	1.489011	0.090137	1.332667	1.33266691	0.414316235	0.414316	0.234663	188	98	78	121.3333	207	184	151	180.6667
osmE	871	603	383.6667	1.234898	0.515068	1.099772	1.099771627	0.137203973	0.137204	0.715219	2133	1505	1262	1633.333	2128	2108	1815	2017
osmY	332	176	157.6667	1.526726	0.06726	1.372456	1.3724555	0.456759373	0.456759	0.193776	465	261	172	299.3333	504	430	437	457
otsA	650	302	209	1.188969	0.672892	1.060688	1.060688109	0.0850005	0.085001	0.824097	1472	1024	822	1106	1326	1332	1287	1315
otsB	49	15	12.33333	1.14741	0.933936	1.012633	1.012632696	0.018110972	0.018111	0.989544	97	86	68	83.66667	93	78	117	96
oxyR	2271	2000	-1966.33	-1.39761	0.003862	-1.59813	1.598129522	0.676384337	-0.67638	0.021967	6781	6911	7043	6911.667	5283	4772	4781	4945.333
pabA	77	22	48.66667	1.22956	0.531085	1.078482	1.078481968	0.109002055	0.109002	0.723599	209	228	199	212	275	231	276	260.6667
pabB	282	100	195.3333	1.259063	0.412232	1.104865	1.104864982	0.143870079	0.14387	0.625818	788	757	717	754	999	857	992	949.3333
pabC	338	251	235	1.441176	0.053517	1.278634	1.278633641	0.354602957	0.354603	0.163977	656	500	442	532.6667	751	772	780	767.6667
pagC	168	21	-81	-1.29926	0.006101	-1.49764	1.497637953	0.582688901	-0.58269	0.031435	292	339	424	351.6667	285	271	256	270.6667
pagD	9	4	5	2.5	0.051285	2.163264	2.163264423	1.113210022	1.11321	0.159076	2	6	2	3.333333	6	11	8	8.333333
pagK	64	23	29.66667	1.194323	0.690955	1.051988	1.051988129	0.073118425	0.073118	0.835588	177	127	154	152.6667	180	191	176	182.3333
pagO	45	24	30.66667	1.766667	0.003904	1.564959	1.564959163	0.646125011	0.646125	0.02218	53	36	31	40	60	76	76	70.66667
pagP	406	354	309.3333	1.773333	0.001521	1.574996	1.574996138	0.655348291	0.655348	0.010647	546	312	342	400	718	696	714	709.3333
pal	3973	1374	-2447.67	-1.70919	5.39E-05	-1.9446	1.944599629	0.959473151	-0.95947	0.000726	5776	7000	4921	5899	3780	3027	3547	3451.333
panB	121	24	39.66667	1.064921	0.601822	-1.06581	1.065805194	0.09194377	-0.09194	0.775646	630	662	541	611	654	641	657	650.6667
panC	260	40	84	1.091008	0.756364	-1.03948	1.039478295	0.055859635	-0.05586	0.880907	999	974	796	923	1056	959	1006	1007
panD	415	299	307.3333	1.387558	0.105151	1.222907	1.222906816	0.290314476	0.290314	0.259344	893	753	733	793	1101	1052	1148	1100.333
panF	65	18	36.66667	1.169492	0.861143	1.023384	1.023384124	0.033347758	0.033348	0.951	214	207	228	216.3333	255	232	272	253
parC	402	52	164.6667	1.094781	0.757885	-1.04328	1.043277843	0.061123423	-0.06112	0.880907	1809	1646	1757	1737.333	2048	1779	1879	1902
parE	642	335	167	1.050627	0.585417	-1.09081	1.0908055	0.125393879	-0.12539	0.764059	2985	3535	3376	3298.667	3627	3200	3570	3465.667
pbpC	214	67	76	1.124183	0.923775	-1.01221	1.01221212	0.017511654	-0.01751	0.984632	663	625	548	612	762	596	706	688
pbpG	300	217	240	1.240722	0.488613	1.090419	1.090419145	0.124882798	0.124883	0.693257	1042	992	957	997	1245	1209	1257	1237
pckA	7287	2978	-3846	-1.08397	0.21787	-1.23373	1.233725314	0.303021218	-0.30302	0.421051	50810	51624	46503	49645.67	48020	45042	44337	45799.67
pcm	342	108	188	1.178143	0.761619	1.038241	1.03824088	0.054141199	0.054141	0.882712	1129	1100	937	1055.333	1279	1208	1243	1243.333
pcnB	394	219	311.3333	1.317903	0.25725	1.153493	1.153493308	0.206009635	0.20601	0.469358	967	997	974	979.3333	1361	1193	1318	1290.667
pdgL	84	52	63.66667	1.423503	0.070395	1.248779	1.248778535	0.320517645	0.320518	0.1993	160	131	160	150.3333	215	212	215	214
pdhR	242	30	-132.667	-1.48068	0.000478	-1.71142	1.711424232	0.775197423	-0.7752	0.004217	316	414	496	408.6667	286	254	288	276
pduA	241	165	202.6667	19.42424	2.36E-38	17.25817	17.25816831	4.109207447	4.109207	8.73E-36	23	5	5	11	170	246	225	213.6667
pduC	1178	760	997	11.10473	1.16E-29	9.772337	9.772336788	3.288703585	3.288704	3.44E-27	124	55	117	98.66667	877	1177	1233	1095.667
pduD	305	169	245.6667	12.16667	4.35E-35	10.70051	10.70050886	3.419607501	3.419608	1.48E-32	27	13	26	22	195	290	318	267.6667

Feature ID	Experiment - Range (original values)	Experiment - IQR (original values)	Experiment - Difference (original values)	Experiment - Fold Change (original values)	EDGE test: yccT NT vs WT NT , tagwise dispersions - P-value	EDGE test: yccT NT vs WT NT , tagwise dispersions - Fold change	yccT NT vs WT NT ABS|FC|	yccT NT vs WT NT Log2FC	yccT NT vs WT NT Log2FC +-	EDGE test: yccT NT vs WT NT , tagwise dispersions - FDR p-value correction	WT NT - WT.1.S22 - Expression values	WT NT - WT.2.S23 - Expression values	WT NT - WT.3.S24 - Expression values	WT NT - Means	yccT NT - yccT.1.S28 - Expression values	yccT NT - yccT.2.S29 - Expression values	yccT NT - yccT.3.S30 - Expression values	yccT NT - Means
pduE	145	84	106.6667	7.27451	2.16E-21	6.38943	6.389430038	2.675687243	2.675687	4.15E-19	24	9	18	17	102	115	154	123.6667
pduF	405	317	331.3333	2.964427	1.95E-12	2.630208	2.630208467	1.39517715	1.395177	1.76E-10	218	138	150	168.6667	467	543	490	500
pduG	234	172	201.6667	4.025	4.4E-21	3.541806	3.541806355	1.824485336	1.824485	8.12E-19	70	64	66	66.66667	238	269	298	268.3333
pduH	22	7	15.33333	2.642857	0.000682	2.306875	2.306875318	1.205940031	1.20594	0.005666	12	8	8	9.333333	30	15	29	24.66667
pduJ	32	9	10.66667	1.627451	0.155332	1.43726	1.43726034	0.52332141	0.523321	0.33296	24	16	11	17	25	15	43	27.66667
pduK	23	10	13	1.6	0.072706	1.409893	1.409893175	0.495585857	0.495586	0.203899	30	15	20	21.66667	38	38	28	34.66667
pduL	38	25	29.66667	2	0.000301	1.757934	1.757934042	0.813880941	0.813881	0.002817	33	25	31	29.66667	59	63	56	59.33333
pduM	24	12	12.33333	2.423077	0.007053	2.13383	2.133830024	1.093445259	1.093445	0.035036	17	4	5	8.666667	28	22	13	21
pduN	6	2	1	1.2	1	1.053853	1.053852734	0.075673278	0.075673	1	4	6	5	5	9	6	3	6
pduO	22	14	16.66667	1.943396	0.005412	1.705228	1.705227606	0.769964316	0.769964	0.028482	20	13	20	17.66667	34	34	35	34.33333
pduP	19	3	-4.66667	-1.09929	0.130964	-1.25136	1.251355664	0.323491895	-0.32349	0.298356	54	52	49	51.66667	54	35	52	47
pduQ	18	11	8.333333	1.373134	0.332332	1.210362	1.21036155	0.275438063	0.275438	0.546001	31	19	17	22.33333	35	27	30	30.66667
pduS	15	5	6.333333	1.256757	0.649521	1.101226	1.101225908	0.139110457	0.13911	0.807754	25	22	27	24.66667	36	21	36	31
pduT	13	6	7.333333	1.916667	0.072859	1.679506	1.67950553	0.748036546	0.748037	0.204078	8	12	4	8	17	15	14	15.33333
pduU	9	6	2.666667	1.285714	0.731334	1.130813	1.130812731	0.177360031	0.17736	0.863453	7	15	6	9.333333	13	13	10	12
pduV	6	3	-2	-1.17143	0.28891	-1.32709	1.327091296	0.408267623	-0.40827	0.505193	14	13	14	13.66667	9	11	15	11.66667
pduW	20	11	4	1.059701	0.658494	-1.06939	1.069391135	0.096789622	-0.09679	0.816164	80	61	60	67	72	73	68	71
pduX	329	37	157.6667	1.155235	0.883233	1.018514	1.018514087	0.026465935	0.026466	0.962948	1101	1064	882	1015.667	1209	1100	1211	1173.333
pdxA	231	46	-27.6667	-1.019	0.248544	-1.16535	1.165348732	0.220761747	-0.22076	0.459749	1419	1567	1465	1483.667	1579	1348	1441	1456
pdxB	406	95	250.3333	1.213413	0.632378	1.063979	1.063978558	0.089469077	0.089469	0.796042	1186	1207	1126	1173	1457	1281	1532	1423.333
pdxH	385	206	250	1.414365	0.0685	1.246617	1.246616825	0.318018089	0.318018	0.196057	675	603	532	603.3333	917	809	834	853.3333
pdxJ	595	337	205.3333	1.149116	0.893578	1.018462	1.018462357	0.026392659	0.026393	0.968783	1747	1232	1152	1377	1671	1507	1569	1582.333
pdxK	121	65	58.66667	1.280702	0.321919	1.13014	1.130139783	0.176501225	0.176501	0.537259	254	189	184	209	305	261	237	267.6667
pdxY	335	163	57	1.034434	0.454847	-1.1108	1.11079829	0.151596861	-0.1516	0.663842	1519	1650	1797	1655.333	1854	1560	1723	1712.333
pegA	83	39	55	1.496988	0.029925	1.309488	1.309487844	0.389002667	0.389003	0.107542	108	110	114	110.6667	191	149	157	165.6667
pegB	18	6	8	1.774194	0.113532	1.568941	1.568940538	0.649790676	0.649791	0.272958	10	15	6	10.33333	9	24	22	18.33333
pegC	53	25	30	1.708661	0.012296	1.519913	1.51991273	0.60398849	0.603988	0.054002	61	40	26	42.33333	73	79	65	72.33333
pegD	78	58	58.33333	2.367188	2.67E-07	2.091685	2.091684713	1.064665405	1.064665	8.63E-06	59	38	31	42.66667	109	96	98	101
pepA	1543	679	-1085.33	-1.35538	0.00658	-1.55562	1.555615766	0.637485761	-0.63749	0.033324	3742	4247	4429	4139.333	3213	2886	3063	3054
pepB	1403	619	-871.667	-1.47092	0.000547	-1.67781	1.677812214	0.746581253	-0.74658	0.004715	2626	3077	2465	2722.667	2033	1674	1846	1851
pepD	2129	1139	-1373.67	-1.17485	0.073875	-1.34484	1.344841588	0.427436244	-0.42744	0.205617	8954	9172	9564	9230	8319	7435	7815	7856.333
pepE	878	201	-509	-1.3153	0.006519	-1.5038	1.503799054	0.588611799	-0.58861	0.033088	1817	2428	2125	2123.333	1616	1677	1550	1614.333
pepN	2063	362	703.6667	1.104624	0.873045	-1.02564	1.025636502	0.036519514	-0.03652	0.958007	7501	6885	5791	6725.667	7854	7187	7247	7429.333
pepP	1132	94	272.6667	1.065148	0.686227	-1.06439	1.064388138	0.090024338	-0.09002	0.833008	4658	4266	3632	4185.333	4764	4258	4352	4458
pepQ	1513	677	-1038	-1.3976	0.004137	-1.6066	1.606602583	0.684013101	-0.68401	0.023203	3219	3679	4048	3648.667	2755	2535	2542	2610.667
pepT	5559	924	-3694.33	-1.45835	0.005452	-1.67739	1.677390299	0.746218417	-0.74622	0.028656	9084	13375	12804	11754.33	8204	8160	7816	8060

Feature ID	Experiment - Range (original values)	Experiment - IQR (original values)	Experiment - Difference (original values)	Experiment - Fold Change (original values)	EDGE test: yccT NT vs WT NT , tagwise dispersions - P-value	EDGE test: yccT NT vs WT NT , tagwise dispersions - Fold change	yccT NT vs WT NT ABS|FC|	yccT NT vs WT NT Log2FC	yccT NT vs WT NT Log2FC +-	EDGE test: yccT NT vs WT NT , tagwise dispersions - FDR p-value correction	WT NT - WT.1.S22 - Expression values	WT NT - WT.2.S23 - Expression values	WT NT - WT.3.S24 - Expression values	WT NT - Means	yccT NT - yccT.1.S28 - Expression values	yccT NT - yccT.2.S29 - Expression values	yccT NT - yccT.3.S30 - Expression values	yccT NT - Means
perM	190	59	87.33333	1.182071	0.728038	1.042442	1.042442236	0.059967443	0.059967	0.860708	537	484	418	479.6667	608	543	550	567
pfkA	388	176	44	1.026118	0.447677	-1.10541	1.105407894	0.14457882	-0.14458	0.657929	1921	1600	1533	1684.667	1776	1634	1776	1728.667
pfkB	1642	682	513	1.146015	0.917402	1.016817	1.016817144	0.02406026	0.02406	0.98236	4471	3240	2829	3513.333	4328	3829	3922	4026.333
pflA	931	556	602.3333	1.403258	0.110261	1.240995	1.24099547	0.311497849	0.311498	0.267562	1756	1447	1278	1493.667	2209	2076	2003	2096
pflB	28631	8013	-16649	-1.82593	0.00023	-2.12194	2.121936548	1.085381516	-1.08538	0.002252	27854	35441	47126	36807	22138	18495	19841	20158
pflC	104	58	13.33333	1.01998	0.369594	-1.12332	1.123318524	0.167767071	-0.16777	0.586979	612	695	695	667.3333	716	637	689	680.6667
pflD	202	92	-75	-1.04391	0.199607	-1.195	1.195002528	0.25701367	-0.25701	0.396797	1689	1790	1870	1783	1774	1668	1682	1708
pflE	47	4	9	1.062791	0.596255	-1.06908	1.069078565	0.096367878	-0.09637	0.771389	145	163	122	143.3333	169	146	142	152.3333
pflF	144	5	-78.3333	-1.15688	0.038292	-1.32324	1.323243802	0.404078897	-0.40408	0.128346	505	635	593	577.6667	491	501	506	499.3333
pfs	602	181	-377	-1.47382	0.000215	-1.69547	1.695468566	0.761684037	-0.76168	0.002137	978	1309	1231	1172.667	883	707	797	795.6667
pgi	995	561	-657	-1.18235	0.058364	-1.35439	1.354387706	0.437640783	-0.43764	0.174265	4036	4310	4434	4260	3895	3475	3439	3603
pgk	5828	2689	-3498	-1.24971	0.035174	-1.42651	1.426510398	0.512490263	-0.51249	0.120538	16685	19124	16709	17506	14732	13296	13996	14008
pgm	908	169	-102	-1.01453	0.359817	-1.15798	1.157983499	0.211614696	-0.21161	0.575994	7020	7488	6851	7119.667	7480	6580	6993	7017.667
pgpA	278	120	121	1.207666	0.58295	1.07371	1.073710136	0.10260457	0.102605	0.761737	722	582	444	582.6667	708	702	701	703.6667
pgpB	342	206	153.6667	1.242632	0.455075	1.105307	1.105307001	0.144447137	0.144447	0.663957	826	584	490	633.3333	832	790	739	787
pgsA	633	346	320	1.240903	0.474276	1.09997	1.099970018	0.1374642	0.137464	0.680115	1628	1282	1075	1328.333	1708	1553	1684	1648.333
pgtA	379	135	216.3333	1.27914	0.384319	1.118671	1.118671353	0.161786259	0.161786	0.601101	733	861	731	775	1110	868	996	991.3333
pgtB	1323	651	877.6667	1.837202	0.000378	1.628731	1.628731173	0.703748502	0.703749	0.00343	1287	1065	793	1048.333	2116	1716	1946	1926
pgtC	818	523	573	1.900943	0.000281	1.69548	1.695480453	0.761694151	0.761694	0.002666	875	580	453	636	1253	1103	1271	1209
pgtE	56	31	41.66667	1.698324	0.001341	1.496445	1.496444787	0.58153905	0.581539	0.009591	66	64	49	59.66667	104	95	105	101.3333
pgtP	172	107	130.3333	2.16369	1.36E-06	1.909712	1.909711579	0.933354767	0.933355	3.47E-05	148	90	98	112	260	205	262	242.3333
pheA	1987	1245	987.3333	1.506411	0.109156	1.352101	1.352101261	0.435203202	0.435203	0.266045	3007	1583	1259	1949.667	3246	2737	2828	2937
pheP	195	133	142.3333	1.51199	0.013833	1.334095	1.334095258	0.415861683	0.415862	0.05928	320	265	249	278	419	398	444	420.3333
pheS	221	71	-83.6667	-1.05414	0.161606	-1.20588	1.205879083	0.270085251	-0.27009	0.342949	1601	1615	1671	1629	1642	1450	1544	1545.333
pheT	983	282	-215	-1.04958	0.257878	-1.19099	1.190988642	0.252159655	-0.25216	0.469513	5082	4474	4099	4551.667	4614	4192	4204	4336.667
phnA	162	6	-101.333	-1.26974	0.009149	-1.45971	1.459707568	0.545679374	-0.54568	0.043356	382	518	531	477	382	376	369	375.6667
phnB	40	3	17.33333	1.287293	0.365373	1.132342	1.132341548	0.179309184	0.179309	0.582363	66	65	50	60.33333	90	63	80	77.66667
phnO	182	141	74.33333	1.333832	0.281939	1.191064	1.191064132	0.252251097	0.252251	0.496728	343	164	161	222.6667	322	264	305	297
phnR	120	62	58.33333	1.341131	0.168475	1.191081	1.191081046	0.252271584	0.252272	0.353462	225	161	127	171	247	223	218	229.3333
phnS	26	17	10	1.243902	0.579762	1.099433	1.099433084	0.136759798	0.13676	0.758915	56	36	31	41	53	43	57	51
phnT	63	22	12.66667	1.116564	0.933196	-1.01655	1.016549808	0.023680905	-0.02368	0.989486	117	117	92	108.6667	114	95	155	121.3333
phnU	14	6	3.666667	1.146667	1	1.009146	1.009145532	0.013134245	0.013134	1	23	33	19	25	32	25	29	28.66667
phnV	83	51	52.66667	1.377088	0.135663	1.210777	1.210777436	0.275933694	0.275934	0.304762	129	170	120	139.6667	194	180	203	192.3333
phnW	28	5	6	1.111801	0.932804	-1.01924	1.019238713	0.02749198	-0.02749	0.989486	64	57	40	53.66667	68	53	58	59.66667
phnX	42	20	24.33333	1.365	0.166244	1.20161	1.201610488	0.26496931	0.264969	0.350776	76	59	65	66.66667	87	85	101	91
phoB	292	194	207	1.342904	0.175087	1.18218	1.182179952	0.24144966	0.24145	0.362868	690	550	571	603.6667	842	765	825	810.6667

Feature ID	Experiment - Range (original values)	Experiment - IQR (original values)	Experiment - Difference (original values)	Experiment - Fold Change (original values)	EDGE test: yccT NT vs WT NT , tagwise dispersions - P-value	EDGE test: yccT NT vs WT NT , tagwise dispersions - Fold change	yccT NT vs WT NT ABS|FC|	yccT NT vs WT NT Log2FC	yccT NT vs WT NT Log2FC +-	EDGE test: yccT NT vs WT NT , tagwise dispersions - FDR p-value correction	WT NT - WT.1.S22 - Expression values	WT NT - WT.2.S23 - Expression values	WT NT - WT.3.S24 - Expression values	WT NT - Means	yccT NT - yccT.1.S28 - Expression values	yccT NT - yccT.2.S29 - Expression values	yccT NT - yccT.3.S30 - Expression values	yccT NT - Means
phoE	109	45	54.66667	1.261146	0.35301	1.116334	1.116333766	0.158768434	0.158768	0.567763	250	211	167	209.3333	276	256	260	264
phoH	1255	704	806.6667	2.394813	5.48E-06	2.13816	2.138159725	1.096369629	1.09637	0.000112	866	480	389	578.3333	1644	1184	1327	1385
phoL	648	354	-460	-1.23445	0.020257	-1.40805	1.408051394	0.493699993	-0.4937	0.078804	2321	2581	2364	2422	1967	1933	1986	1962
phoN	61	11	-1.66667	-1.01348	0.345692	-1.16684	1.166843075	0.222610551	-0.22261	0.560263	87	141	148	125.3333	124	129	118	123.6667
phoP	522	215	16.66667	1.005377	0.429134	-1.12848	1.128481941	0.174383332	-0.17438	0.640233	3405	3011	2883	3099.667	3180	3204	2965	3116.333
phoQ	534	85	232	1.127918	0.955496	-1.00776	1.007762469	0.011155634	-0.01116	1	1874	1944	1623	1813.667	2157	1959	2021	2045.667
phoR	203	122	150	1.339879	0.174155	1.17757	1.177570341	0.23581324	0.235813	0.361757	484	416	424	441.3333	609	546	619	591.3333
phoU	112	26	-48.6667	-1.07307	0.101436	-1.22565	1.225651177	0.293548443	-0.29355	0.253789	702	733	709	714.6667	701	621	676	666
phrB	291	162	194.3333	1.779412	0.000322	1.57734	1.577340158	0.657493815	0.657494	0.002983	319	238	191	249.3333	482	400	449	443.6667
phsA	2658	1490	1301	1.124125	0.949683	-1.01062	1.010616148	0.015235137	-0.01524	1	11731	9472	10241	10481.33	12073	12130	11144	11782.33
phsB	975	518	572.6667	1.25128	0.528106	1.100721	1.100720954	0.138448775	0.138449	0.722435	2603	1987	2247	2279	2962	2828	2765	2851.667
phsC	1759	1156	1135	1.328636	0.317523	1.173142	1.173142094	0.230377767	0.230378	0.532988	4023	3061	3277	3453.667	4513	4820	4433	4588.667
pipA	345	268	246	1.647937	0.004244	1.465958	1.465957755	0.551843529	0.551844	0.023564	504	337	298	379.6667	643	629	605	625.6667
pipB	26	14	16	1.716418	0.020721	1.513899	1.513899124	0.598269077	0.598269	0.080046	29	21	17	22.33333	37	43	35	38.33333
pipC	45	15	26.33333	1.49375	0.047719	1.31814	1.318139559	0.398503125	0.398503	0.151609	60	61	39	53.33333	84	75	80	79.66667
pipD	1414	1181	956.3333	1.58242	0.030839	1.41287	1.41287041	0.498629146	0.498629	0.10958	2306	1381	1239	1642	2653	2562	2580	2598.333
pitA	502	247	221.3333	1.092158	0.750899	-1.05074	1.050741514	0.071407805	-0.07141	0.877666	2286	2336	2583	2401.667	2744	2337	2788	2623
pldA	910	333	-541.333	-1.25539	0.0186	-1.43137	1.431374054	0.517400734	-0.5174	0.073984	2511	2908	2564	2661	2183	2178	1998	2119.667
pldB	586	188	-321	-1.24275	0.007994	-1.4156	1.415604219	0.501417967	-0.50142	0.039009	1595	1843	1492	1643.333	1406	1257	1304	1322.333
plsB	731	114	303.6667	1.0517	0.611063	-1.0845	1.084496596	0.117025525	-0.11703	0.781195	5891	6005	5725	5873.667	6456	5969	6107	6177.333
plsC	170	120	124.6667	1.259003	0.401366	1.10803	1.108030006	0.147996951	0.147997	0.616499	521	468	455	481.3333	605	588	625	606
plsX	1008	121	-548.333	-1.34051	0.006365	-1.53909	1.539089262	0.622076906	-0.62208	0.032453	1701	2569	2206	2158.667	1690	1561	1580	1610.333
pmbA	849	183	171.3333	1.072856	0.730734	-1.05278	1.052777501	0.074200562	-0.0742	0.862976	2810	2284	1961	2351.667	2658	2467	2444	2523
pmgI	3409	1078	-2313	-1.47252	0.002732	-1.68955	1.689548064	0.756637392	-0.75664	0.016718	5921	8213	7490	7208	5038	4843	4804	4895
pmrD	112	66	-54	-1.081	0.094249	-1.23627	1.236269865	0.305993704	-0.30599	0.240702	682	752	728	720.6667	713	640	647	666.6667
pmrF	407	334	261.6667	1.299733	0.276436	1.152389	1.152388925	0.2046277	0.204628	0.48944	1087	793	739	873	1146	1131	1127	1134.667
pncA	114	13	42.33333	1.128024	0.950193	-1.00816	1.00815609	0.011719025	-0.01172	1	348	351	293	330.6667	407	338	374	373
pncB	497	240	303.6667	1.349444	0.184872	1.188678	1.188678032	0.249357996	0.249358	0.373708	1042	763	802	869	1260	1040	1218	1172.667
pnp	2906	1104	68.33333	1.003969	0.450148	-1.13405	1.134051456	0.181486102	-0.18149	0.659356	18866	16329	16460	17218.33	18467	15960	17433	17286.67
pntA	1007	430	459.6667	1.080794	0.738482	-1.05429	1.054293569	0.076276642	-0.07628	0.868526	5992	5562	5514	5689.333	6521	5878	6048	6149
pntB	659	196	-45.3333	-1.01097	0.358888	-1.15596	1.155961708	0.209093608	-0.20909	0.574714	4241	4050	4239	4176.667	4505	3846	4043	4131.333
pnuC	150	81	71.66667	1.369416	0.142899	1.215	1.215000313	0.280956685	0.280957	0.315489	259	178	145	194	295	235	267	265.6667
pocR	1102	948	799.6667	1.388439	0.172861	1.229917	1.229916933	0.298560881	0.298561	0.359937	2519	1857	1800	2058.667	2868	2902	2805	2858.333
polA	2830	385	-86.3333	-1.0114	0.414308	-1.14211	1.142107387	0.191698307	-0.1917	0.627195	9349	7109	6519	7659	7967	7494	7257	7572.667
polB	308	32	44	1.05658	0.607051	-1.0679	1.067900232	0.09477687	-0.09478	0.777861	935	771	627	777.6667	860	803	802	821.6667
potA	148	45	-26.6667	-1.04442	0.158713	-1.19185	1.191848737	0.253201149	-0.2532	0.33843	614	677	590	627	635	529	637	600.3333

Feature ID	Experiment - Range (original values)	Experiment - IQR (original values)	Experiment - Difference (original values)	Experiment - Fold Change (original values)	EDGE test: yccT NT vs WT NT , tagwise dispersions - P-value	EDGE test: yccT NT vs WT NT , tagwise dispersions - Fold change	yccT NT vs WT NT ABS|FC|	yccT NT vs WT NT Log2FC	yccT NT vs WT NT Log2FC +-	EDGE test: yccT NT vs WT NT , tagwise dispersions - FDR p-value correction	WT NT - WT.1.S22 - Expression values	WT NT - WT.2.S23 - Expression values	WT NT - WT.3.S24 - Expression values	WT NT - Means	yccT NT - yccT.1.S28 - Expression values	yccT NT - yccT.2.S29 - Expression values	yccT NT - yccT.3.S30 - Expression values	yccT NT - Means
potB	57	23	30.66667	1.158076	0.908311	1.015947	1.015946872	0.02282496	0.022825	0.976638	179	209	194	194	221	236	217	224.6667
potC	295	54	-73.3333	-1.08109	0.122863	-1.23422	1.234217715	0.303596907	-0.3036	0.286982	859	1154	920	977.6667	946	901	866	904.3333
potD	2051	312	-1217	-1.7611	0.000207	-2.04718	2.047176425	1.033635439	-1.03364	0.002087	1891	2999	3558	2816	1711	1507	1579	1599
potE	5883	1029	-3444.67	-3.49734	3.35E-08	-4.10884	4.108836606	2.038729961	-2.03873	1.4E-06	2392	4841	7239	4824	1356	1363	1419	1379.333
potF	81	54	63.66667	1.672535	0.001781	1.471503	1.471502789	0.557290277	0.55729	0.01203	104	85	95	94.66667	160	166	149	158.3333
potG	53	20	31.33333	1.37751	0.136071	1.208302	1.208302313	0.272981457	0.272981	0.30528	80	92	77	83	130	100	113	114.3333
potH	31	8	11	1.221477	0.721811	1.06756	1.067560346	0.094317624	0.094318	0.85563	41	57	51	49.66667	72	49	61	60.66667
potI	93	13	41.33333	1.441281	0.073608	1.268278	1.268278028	0.342871043	0.342871	0.205036	108	98	75	93.66667	168	111	126	135
poxB	60	4	12	1.131868	0.962647	1.007327	1.007327443	0.010532724	0.010533	1	115	103	55	91	108	102	99	103
ppa	2439	436	-1573	-1.43254	0.005298	-1.65001	1.650005525	0.722470855	-0.72247	0.028016	4010	5916	5703	5209.667	3859	3574	3477	3636.667
ppc	1379	589	-330	-1.05215	0.257799	-1.20851	1.208514699	0.273235021	-0.27324	0.469513	6035	6730	7209	6658	6624	5830	6530	6328
ppdA	20	2	-1.66667	-1.05814	0.387624	-1.19772	1.197718451	0.260288812	-0.26029	0.604562	41	21	29	30.33333	27	30	29	28.66667
ppdB	42	14	18	1.367347	0.226452	1.201397	1.201396643	0.264712538	0.264713	0.431711	61	39	47	49	81	54	66	67
ppdC	17	5	0.333333	1.010309	0.556519	-1.12144	1.121443992	0.16535757	-0.16536	0.741429	43	27	27	32.33333	40	32	26	32.66667
ppdD	23	7	9.333333	1.311111	0.412933	1.159395	1.159395273	0.213372509	0.213373	0.625818	31	38	21	30	31	43	44	39.33333
pphB	10	4	3	1.409091	0.520096	1.236793	1.236793393	0.306604517	0.306605	0.716811	9	9	4	7.333333	14	5	12	10.33333
ppiA	442	331	297	1.384217	0.128437	1.22262	1.222619936	0.289975997	0.289976	0.295639	951	646	722	773	1088	1053	1069	1070
ppiB	1152	645	-787.667	-1.19007	0.054701	-1.36238	1.36238014	0.446129309	-0.44613	0.165899	4710	4909	5176	4931.667	4343	4065	4024	4144
ppiC	429	78	-251.333	-1.40472	0.001183	-1.61816	1.618159904	0.69435418	-0.69435	0.008735	711	886	1020	872.3333	639	633	591	621
ppk	498	112	29.33333	1.008604	0.429694	-1.12947	1.129466673	0.175641701	-0.17564	0.640853	3334	3696	3198	3409.333	3527	3343	3446	3438.667
pps	6951	4490	3761	1.164379	0.876915	1.026349	1.026348536	0.037520736	0.037521	0.959647	26033	21543	21064	22880	28015	26211	25697	26641
ppx	251	125	38.33333	1.024427	0.418518	-1.11505	1.115052166	0.157111206	-0.15711	0.631894	1479	1730	1499	1569.333	1714	1492	1617	1607.667
pqaA	63	35	44.33333	2.927536	3.5E-08	2.569725	2.569724852	1.361613894	1.361614	1.45E-06	29	16	24	23	64	59	79	67.33333
pqaB	193	57	-99.3333	-1.2081	0.010701	-1.376	1.376000058	0.460480531	-0.46048	0.048482	550	646	534	576.6667	477	453	502	477.3333
pqiA	107	27	40	1.116167	0.923393	-1.01273	1.012732659	0.018253383	-0.01825	0.984632	390	359	284	344.3333	391	386	376	384.3333
pqiB	271	63	37.33333	1.043973	0.517161	-1.08307	1.083072057	0.115129229	-0.11513	0.715467	990	838	719	849	905	853	901	886.3333
prc	1297	591	525	1.131392	0.913971	-1.01874	1.018742916	0.026790027	-0.02679	0.980345	3480	3958	4549	3995.667	4777	4043	4742	4520.667
prfA	529	273	346	1.277689	0.393115	1.119511	1.11951065	0.162868253	0.162868	0.61	1202	1361	1175	1246	1704	1475	1597	1592
prfB	466	192	227.6667	1.119909	0.881586	-1.0216	1.021595246	0.030823716	-0.03082	0.962382	1782	2053	1861	1898.667	2248	1887	2244	2126.333
prfC	324	49	139	1.190411	0.832157	1.032423	1.032422883	0.046034023	0.046034	0.932782	587	777	826	730	911	788	908	869
prfH	109	35	60	1.22113	0.530373	1.077072	1.077072349	0.107115162	0.107115	0.723325	287	293	234	271.3333	329	322	343	331.3333
prgH	88	16	35	1.271318	0.378277	1.122153	1.122153191	0.166269639	0.16627	0.595011	137	153	97	129	168	139	185	164
prgI	52	13	-10.6667	-1.19876	0.134405	-1.34337	1.343370016	0.425856734	-0.42586	0.302923	75	85	33	64.33333	49	50	62	53.66667
prgJ	35	20	4	1.054299	0.636299	-1.07967	1.079666565	0.110585831	-0.11059	0.798482	66	95	60	73.66667	83	63	87	77.66667
prgK	55	7	13.33333	1.102828	0.875271	-1.02504	1.025039294	0.035679215	-0.03568	0.959152	140	148	101	129.6667	133	140	156	143
priA	154	59	33.66667	1.040207	0.461711	-1.10097	1.100972861	0.138778907	-0.13878	0.66967	763	877	872	837.3333	878	818	917	871

Feature ID	Experiment - Range (original values)	Experiment - IQR (original values)	Experiment - Difference (original values)	Experiment - Fold Change (original values)	EDGE test: yccT NT vs WT NT , tagwise dispersions - P-value	EDGE test: yccT NT vs WT NT , tagwise dispersions - Fold change	yccT NT vs WT NT ABS|FC|	yccT NT vs WT NT Log2FC	yccT NT vs WT NT Log2FC +-	EDGE test: yccT NT vs WT NT , tagwise dispersions - FDR p-value correction	WT NT - WT.1.S22 - Expression values	WT NT - WT.2.S23 - Expression values	WT NT - WT.3.S24 - Expression values	WT NT - Means	yccT NT - yccT.1.S28 - Expression values	yccT NT - yccT.2.S29 - Expression values	yccT NT - yccT.3.S30 - Expression values	yccT NT - Means
priB	3021	147	-1658	-1.72255	0.000256	-1.9218	1.921801732	0.942459504	-0.94246	0.002452	5158	4221	2479	3952.667	2332	2415	2137	2294.667
priC	75	50	34.33333	1.207661	0.588711	1.071166	1.071165779	0.099181776	0.099182	0.76537	202	152	142	165.3333	178	217	204	199.6667
prkB	375	128	-27.3333	-1.01614	0.282478	-1.15328	1.153282642	0.205746127	-0.20575	0.49748	1785	1876	1501	1720.667	1718	1617	1745	1693.333
prlC	1214	227	-516.667	-1.13719	0.104111	-1.28682	1.286824016	0.363814767	-0.36381	0.258006	4861	4303	3684	4282.667	3911	3740	3647	3766
prmA	83	32	10.33333	1.034483	0.429864	-1.11023	1.110233795	0.150863514	-0.15086	0.640861	260	317	322	299.6667	343	285	302	310
proA	222	80	-86.6667	-1.05919	0.158414	-1.21343	1.213433837	0.279095447	-0.2791	0.338117	1425	1597	1631	1551	1505	1409	1479	1464.333
proB	328	162	34.33333	1.020551	0.399825	-1.12216	1.122163645	0.16628308	-0.16628	0.615134	1575	1764	1673	1670.667	1853	1525	1737	1705
proC	1707	729	673	1.225713	0.592903	1.090332	1.090332418	0.124768047	0.124768	0.769073	3825	2870	2250	2981.667	3957	3599	3408	3654.667
proP	274	170	130	1.272537	0.35601	1.129706	1.12970611	0.175947508	0.175948	0.571344	616	444	371	477	645	562	614	607
proQ	938	391	602.3333	1.225199	0.68632	1.067039	1.067038867	0.093612727	0.093613	0.833008	2474	2631	2919	2674.667	3397	3022	3412	3277
proS	1171	882	871	1.341212	0.267325	1.18142	1.181420154	0.240522129	0.240522	0.479757	2842	2451	2365	2552.667	3536	3333	3402	3423.667
proV	218	174	151.3333	1.403915	0.085353	1.241153	1.241153371	0.311681403	0.311681	0.22701	461	327	336	374.6667	545	523	510	526
proW	91	67	63.33333	1.405983	0.069489	1.243165	1.243164896	0.314017671	0.314018	0.198011	193	138	137	156	228	225	205	219.3333
proX	260	106	-105.667	-1.19813	0.01579	-1.35611	1.356109159	0.439473311	-0.43947	0.065572	750	648	519	639	608	490	502	533.3333
proY	309	106	145.3333	1.310541	0.241583	1.156634	1.156633807	0.209932176	0.209932	0.451668	559	453	392	468	701	550	589	613.3333
prpA	56	34	35.66667	1.891667	0.002212	1.673911	1.673910747	0.743222605	0.743223	0.014025	60	28	32	40	77	66	84	75.66667
prpB	743	106	-40	-1.1241	0.468719	-1.35561	1.35560508	0.438936948	-0.43894	0.676072	240	52	795	362.3333	346	241	380	322.3333
prpC	1221	182	-92	-1.18827	0.385807	-1.43897	1.438966752	0.525033258	-0.52503	0.602364	325	98	1319	580.6667	507	381	578	488.6667
prpD	2157	155	-436.333	-1.91603	0.115743	-2.35028	2.350281766	1.232833726	-1.23283	0.276448	349	116	2273	912.6667	499	344	586	476.3333
prpE	2209	145	-528.333	-2.15609	0.033443	-2.63733	2.637328322	1.399077184	-1.39908	0.116226	389	179	2388	985.3333	465	320	586	457
prpR	58	20	23.66667	1.141153	0.964546	-1.00452	1.004519184	0.006505116	-0.00651	1	163	157	183	167.6667	215	165	194	191.3333
prsA	1465	778	884.3333	1.251779	0.625101	1.086559	1.086558901	0.119766384	0.119766	0.788797	3268	3223	4046	3512.333	4662	3840	4688	4396.667
psd	150	61	-56.6667	-1.05431	0.141591	-1.20466	1.20466477	0.268631734	-0.26863	0.314008	1097	1106	1097	1100	1122	972	1036	1043.333
psiF	239	193	178.3333	3.40991	5.51E-06	3.100175	3.100174821	1.632349572	1.63235	0.000112	146	42	34	74	249	235	273	252.3333
pspA	211	73	-133.333	-1.28694	0.003962	-1.46988	1.46987671	0.55569515	-0.5557	0.022392	555	626	613	598	482	497	415	464.6667
pspB	50	28	23	1.526718	0.091452	1.362097	1.362097444	0.445829917	0.44583	0.23684	68	35	28	43.66667	59	63	78	66.66667
pspC	64	47	42	1.577982	0.012143	1.398633	1.398633092	0.484017545	0.484018	0.053512	98	66	54	72.66667	118	113	113	114.6667
pspD	41	21	19.33333	1.349398	0.241163	1.18994	1.189940326	0.250889226	0.250889	0.451455	71	45	50	55.33333	86	75	63	74.66667
pspE	350	20	-68.6667	-1.04761	0.197953	-1.19273	1.192734491	0.254272928	-0.25427	0.394392	1381	1731	1421	1511	1401	1512	1414	1442.333
pspF	115	50	51.66667	1.202086	0.601742	1.067174	1.067173852	0.093795223	0.093795	0.775646	305	255	207	255.6667	280	322	320	307.3333
pssA	645	257	-249.333	-1.05297	0.246033	-1.2021	1.202097591	0.265554024	-0.26555	0.457286	4842	5154	4873	4956.333	4996	4616	4509	4707
pstA	98	41	60.66667	1.435407	0.04793	1.267096	1.267095884	0.341525701	0.341526	0.151698	168	134	116	139.3333	211	175	214	200
pstB	87	34	11.66667	1.022293	0.367878	-1.11646	1.116460896	0.158932722	-0.15893	0.584673	510	562	498	523.3333	580	493	532	535
pstC	129	50	73	1.421965	0.081975	1.250175	1.250175061	0.322130129	0.32213	0.220943	209	151	159	173	280	198	260	246
pstS	280	150	151.3333	1.595801	0.020463	1.423952	1.423952401	0.509900922	0.509901	0.079468	368	218	176	254	416	344	456	405.3333
pta	10571	2440	-6365.67	-1.65369	0.000756	-1.91566	1.915656608	0.937838973	-0.93784	0.006109	12313	16520	19478	16103.67	10434	8907	9873	9738

Feature ID	Experiment - Range (original values)	Experiment - IQR (original values)	Experiment - Difference (original values)	Experiment - Fold Change (original values)	EDGE test: yccT NT vs WT NT , tagwise dispersions - P-value	EDGE test: yccT NT vs WT NT , tagwise dispersions - Fold change	yccT NT vs WT NT ABS|FC|	yccT NT vs WT NT Log2FC	yccT NT vs WT NT Log2FC +-	EDGE test: yccT NT vs WT NT , tagwise dispersions - FDR p-value correction	WT NT - WT.1.S22 - Expression values	WT NT - WT.2.S23 - Expression values	WT NT - WT.3.S24 - Expression values	WT NT - Means	yccT NT - yccT.1.S28 - Expression values	yccT NT - yccT.2.S29 - Expression values	yccT NT - yccT.3.S30 - Expression values	yccT NT - Means
pth	76	17	-4.66667	-1.00804	0.255627	-1.15172	1.151718246	0.203787822	-0.20379	0.467472	584	581	590	585	625	549	567	580.3333
ptpS	95	26	-9.66667	-1.02678	0.221021	-1.16059	1.160588291	0.214856279	-0.21486	0.425062	406	395	311	370.6667	346	372	365	361
ptr	158	105	1.333333	1.000685	0.342568	-1.13798	1.137976922	0.1864713	-0.18647	0.557066	1980	2007	1849	1945.333	1992	1875	1973	1946.667
ptrB	530	308	324	1.280277	0.33797	1.131837	1.131837016	0.178666226	0.178666	0.551582	1328	1149	991	1156	1521	1462	1457	1480
ptsA	1048	168	-593.667	-1.27216	0.017227	-1.43926	1.439262411	0.525329653	-0.52533	0.069795	2863	3137	2325	2775	2157	2298	2089	2181.333
ptsG	1041	115	-452	-1.29036	0.010178	-1.48087	1.480872147	0.566447089	-0.56645	0.046832	1627	2438	1961	2008.667	1694	1397	1579	1556.667
ptsH	1250	322	-785.667	-1.204	0.054433	-1.38235	1.382352916	0.467125984	-0.46713	0.165539	4001	4976	4934	4637	4075	3753	3726	3851.333
ptsI	3086	974	-1247.67	-1.08595	0.201571	-1.24342	1.243422018	0.31431603	-0.31432	0.398911	14496	16840	15957	15764.33	15385	13754	14411	14516.67
ptsJ	47	22	20	1.114068	0.884669	-1.0179	1.017898743	0.025594054	-0.02559	0.964085	196	174	156	175.3333	196	187	203	195.3333
ptsN	683	401	-337.333	-1.10982	0.121924	-1.26493	1.264931657	0.33905944	-0.33906	0.285537	3547	3468	3212	3409	3376	2864	2975	3071.667
ptsO	203	74	-18.6667	-1.04523	0.202993	-1.17914	1.179135696	0.237729755	-0.23773	0.400829	528	441	325	431.3333	447	367	424	412.6667
ptsP	1678	385	624.6667	1.131168	0.983864	-1.00328	1.003283983	0.004730023	-0.00473	1	4940	5325	4022	4762.333	5700	5047	5414	5387
ptxA	289	189	219	1.734078	0.000673	1.531836	1.531836196	0.615262034	0.615262	0.005601	341	280	274	298.3333	520	563	469	517.3333
pudB	582	376	483.6667	14.31193	2.27E-33	12.6982	12.6981969	3.666551748	3.666552	7.2E-31	56	15	38	36.33333	414	597	549	520
purA	2822	245	-1373.33	-1.20154	0.062161	-1.37883	1.378827972	0.463442472	-0.46344	0.18307	6893	9252	8418	8187.667	7129	6430	6884	6814.333
purB	186	78	36	1.052048	0.519825	-1.0939	1.093903416	0.129485363	-0.12949	0.71666	591	707	777	691.6667	759	673	751	727.6667
purC	205	32	-114	-1.2007	0.01542	-1.37845	1.378448739	0.463045618	-0.46305	0.064335	606	713	727	682	607	522	575	568
purD	199	30	-35.6667	-1.05069	0.150011	-1.20076	1.200758782	0.26394636	-0.26395	0.325833	711	808	699	739.3333	773	609	729	703.6667
purE	73	44	51.66667	1.794872	0.000282	1.582313	1.582312694	0.662034731	0.662035	0.002674	76	63	56	65	114	107	129	116.6667
purF	164	108	77	1.087269	0.705146	-1.04942	1.04942294	0.069596233	-0.0696	0.845374	851	959	837	882.3333	1001	886	991	959.3333
purG	583	327	423.6667	1.606972	0.006694	1.412191	1.412191067	0.497935295	0.497935	0.033782	804	619	671	698	1202	998	1165	1121.667
purH	147	79	102.6667	1.268059	0.41092	1.109427	1.109427294	0.149815123	0.149815	0.62527	366	394	389	383	513	468	476	485.6667
purK	98	54	71.66667	1.308465	0.210454	1.15067	1.150669652	0.202473707	0.202474	0.411346	248	232	217	232.3333	315	286	311	304
purM	197	111	126.3333	1.442757	0.051133	1.270158	1.270157787	0.345007729	0.345008	0.158775	340	244	272	285.3333	441	383	411	411.6667
purN	105	53	77	1.225806	0.548659	1.074481	1.074481074	0.103640071	0.10364	0.736253	348	337	338	341	442	391	421	418
purR	229	57	144.3333	1.265644	0.399798	1.109128	1.109128263	0.149426213	0.149426	0.615134	563	546	521	543.3333	750	603	710	687.6667
purT	35	4	4.333333	1.027484	0.398213	-1.11065	1.110654865	0.15141057	-0.15141	0.613946	155	156	162	157.6667	159	146	181	162
purU	619	269	351.3333	1.257639	0.427442	1.107551	1.107551411	0.147373669	0.147374	0.639847	1510	1355	1226	1363.667	1845	1624	1676	1715
putP	679	309	415	2.246246	0.000222	2.007287	2.007286954	1.005246874	1.005247	0.002178	335	486	178	333	644	743	857	748
pykA	13047	5421	-9485.67	-1.64127	0.000562	-1.88603	1.886033404	0.915355228	-0.91536	0.004806	19815	27372	25646	24277.67	15657	14394	14325	14792
pykF	1068	236	-222	-1.08403	0.195374	-1.25286	1.25285814	0.325223069	-0.32522	0.389604	2397	2730	3465	2864	2821	2494	2611	2642
pyrB	850	219	484.3333	1.962252	0.00708	1.708914	1.708914319	0.773080066	0.77308	0.035132	619	259	632	503.3333	1016	838	1109	987.6667
pyrC	455	306	348.6667	1.470324	0.060077	1.284755	1.284755007	0.361493274	0.361493	0.177942	741	676	807	741.3333	1092	1047	1131	1090
pyrD	174	135	149.3333	1.45761	0.044985	1.277629	1.277629143	0.353469126	0.353469	0.144967	329	318	332	326.3333	492	471	464	475.6667
pyrE	94	16	-42	-1.16689	0.028467	-1.34082	1.3408249	0.423120847	-0.42312	0.10322	258	290	333	293.6667	266	239	250	251.6667
pyrF	70	56	57	1.529412	0.012567	1.347898	1.347897528	0.430710822	0.430711	0.054968	120	102	101	107.6667	158	165	171	164.6667

Feature ID	Experiment - Range (original values)	Experiment - IQR (original values)	Experiment - Difference (original values)	Experiment - Fold Change (original values)	EDGE test: yccT NT vs WT NT , tagwise dispersions - P-value	EDGE test: yccT NT vs WT NT , tagwise dispersions - Fold change	yccT NT vs WT NT ABS|FC|	yccT NT vs WT NT Log2FC	yccT NT vs WT NT Log2FC +-	EDGE test: yccT NT vs WT NT , tagwise dispersions - FDR p-value correction	WT NT - WT.1.S22 - Expression values	WT NT - WT.2.S23 - Expression values	WT NT - WT.3.S24 - Expression values	WT NT - Means	yccT NT - yccT.1.S28 - Expression values	yccT NT - yccT.2.S29 - Expression values	yccT NT - yccT.3.S30 - Expression values	yccT NT - Means
pyrG	1494	883	1182	1.229872	0.644257	1.076841	1.07684055	0.106804643	0.106805	0.804434	5022	5303	5101	5142	6516	5984	6472	6324
pyrH	341	155	17	1.020166	0.411725	-1.13406	1.134060626	0.181497768	-0.1815	0.62552	671	846	1012	843	901	746	933	860
pyrI	575	146	314.6667	1.652835	0.050032	1.442662	1.442661542	0.528732874	0.528733	0.15639	591	262	593	482	816	737	837	796.6667
pyrL	1430	540	755.3333	1.856387	0.071895	1.635653	1.635652636	0.709866395	0.709866	0.202393	1353	258	1035	882	1688	1575	1649	1637.333
qor	506	176	-282.667	-1.2009	0.017288	-1.3652	1.365196397	0.449108512	-0.44911	0.069894	1816	1681	1572	1689.667	1515	1396	1310	1407
queA	235	56	123	1.272727	0.331369	1.123299	1.123299286	0.167742364	0.167742	0.545487	506	471	376	451	611	527	584	574
radA	298	155	211.6667	1.291954	0.302359	1.135656	1.135655866	0.183525726	0.183526	0.517679	764	727	684	725	946	882	982	936.6667
radC	31	10	3.666667	1.02439	0.396029	-1.11199	1.111988091	0.153141337	-0.15314	0.61207	138	169	144	150.3333	153	154	155	154
rarD	73	21	7.333333	1.034429	0.449891	-1.09933	1.099326137	0.136619455	-0.13662	0.659356	214	248	177	213	250	195	216	220.3333
ratA	336	172	185.3333	1.22046	0.547129	1.07767	1.077669636	0.107914981	0.107915	0.734868	979	807	736	840.6667	1072	977	1029	1026
ratB	363	19	177.3333	1.186275	0.738571	1.042905	1.042905314	0.060608181	0.060608	0.868526	1006	998	852	952	1215	1017	1156	1129.333
rbfA	384	262	-307.667	-1.57615	2.47E-06	-1.79666	1.796658204	0.845315977	-0.84532	5.75E-05	825	895	805	841.6667	548	511	543	534
rbn	169	66	-51	-1.05305	0.148607	-1.20411	1.204105629	0.267961957	-0.26796	0.323936	967	1070	1000	1012.333	1049	901	934	961.3333
rbsA	31	7	-11	-1.0631	0.103998	-1.20556	1.205561246	0.269704946	-0.2697	0.257959	196	195	165	185.3333	170	176	177	174.3333
rbsB	3241	524	459	1.030098	0.551864	-1.10482	1.104817712	0.143808353	-0.14381	0.738541	16954	13713	15084	15250.33	16269	15251	15608	15709.33
rbsC	81	41	-1	-1.0027	0.271309	-1.1388	1.138796374	0.187509804	-0.18751	0.484104	394	398	324	372	405	353	355	371
rbsD	55	7	-23.6667	-1.26493	0.00693	-1.43808	1.438081424	0.524145363	-0.52415	0.034581	103	140	96	113	94	85	89	89.33333
rbsK	893	328	-192.333	-1.02848	0.318817	-1.17202	1.172015315	0.228991422	-0.22899	0.533691	6871	7422	6543	6945.333	7072	6529	6658	6753
rbsR	587	132	-110.667	-1.03267	0.291972	-1.17714	1.177137095	0.235282353	-0.23528	0.50842	3394	3837	3262	3497.667	3570	3250	3341	3387
rcnA	370	291	274.3333	2.406838	4.83E-06	2.156111	2.156111103	1.108431521	1.108432	9.9E-05	307	148	130	195	500	439	469	469.3333
rcsA	84	16	42.66667	1.283186	0.297328	1.13482	1.13482024	0.182463787	0.182464	0.51303	169	168	115	150.6667	199	184	197	193.3333
rcsB	581	96	202.3333	1.066579	0.680739	-1.06466	1.064663714	0.090397811	-0.0904	0.828213	3122	3278	2717	3039	3298	3208	3218	3241.333
rcsC	320	232	224	1.311399	0.243194	1.155502	1.155502017	0.208519778	0.20852	0.453533	813	677	668	719.3333	988	933	909	943.3333
rcsF	163	93	120.3333	1.241633	0.493307	1.090891	1.090890838	0.125506743	0.125507	0.697625	507	486	501	498	594	649	612	618.3333
rdgC	342	183	189.6667	1.190428	0.716132	1.046946	1.046945653	0.066186554	0.066187	0.851632	1122	899	967	996	1241	1150	1166	1185.667
recA	1281	396	-657.333	-1.10292	0.150248	-1.25468	1.254680193	0.327319681	-0.32732	0.326155	7398	7099	6636	7044.333	6720	6324	6117	6387
recB	737	335	-235.333	-1.07811	0.175887	-1.22689	1.226888177	0.295003762	-0.295	0.364014	3472	3316	2957	3248.333	3292	2735	3012	3013
recC	426	117	214.6667	1.137255	1	1.000083	1.00008317	0.000119984	0.00012	1	1651	1596	1445	1564	1871	1713	1752	1778.667
recD	401	72	-101.333	-1.10007	0.081435	-1.24826	1.248259415	0.319917788	-0.31992	0.220157	1308	1053	981	1114	1136	907	995	1012.667
recF	366	84	-176.667	-1.2531	0.014646	-1.44652	1.446517673	0.532583949	-0.53258	0.062161	682	972	970	874.6667	766	606	722	698
recG	429	23	-217	-1.33626	0.003287	-1.53867	1.53867351	0.62168714	-0.62169	0.019524	683	992	912	862.3333	698	563	675	645.3333
recJ	562	221	234.6667	1.096876	0.799074	-1.03833	1.038333409	0.054269768	-0.05427	0.906631	2604	2383	2280	2422.333	2842	2387	2742	2657
recN	435	306	253.3333	1.34296	0.178711	1.1919	1.191900193	0.253263432	0.253263	0.366606	956	655	605	738.6667	1040	961	975	992
recO	132	37	75.66667	1.399648	0.116267	1.226269	1.226269464	0.294276036	0.294276	0.276977	184	198	186	189.3333	256	223	316	265
recQ	170	54	47.33333	1.054511	0.519114	-1.08432	1.084317251	0.116786924	-0.11679	0.716348	847	901	857	868.3333	991	821	935	915.6667
recR	675	217	-432.333	-1.56662	4.72E-05	-1.8046	1.804596844	0.851676568	-0.85168	0.00066	992	1327	1267	1195.333	862	652	775	763

Feature ID	Experiment - Range (original values)	Experiment - IQR (original values)	Experiment - Difference (original values)	Experiment - Fold Change (original values)	EDGE test: yccT NT vs WT NT , tagwise dispersions - P-value	EDGE test: yccT NT vs WT NT , tagwise dispersions - Fold change	yccT NT vs WT NT ABS|FC|	yccT NT vs WT NT Log2FC	yccT NT vs WT NT Log2FC +-	EDGE test: yccT NT vs WT NT , tagwise dispersions - FDR p-value correction	WT NT - WT.1.S22 - Expression values	WT NT - WT.2.S23 - Expression values	WT NT - WT.3.S24 - Expression values	WT NT - Means	yccT NT - yccT.1.S28 - Expression values	yccT NT - yccT.2.S29 - Expression values	yccT NT - yccT.3.S30 - Expression values	yccT NT - Means
relA	4262	1372	1697	1.184885	0.761607	1.052365	1.05236505	0.073635242	0.073635	0.882712	11048	9506	6982	9178.667	11244	10505	10878	10875.67
rep	191	72	-41.6667	-1.06109	0.146374	-1.21507	1.215070118	0.28103957	-0.28104	0.320447	629	820	722	723.6667	735	661	650	682
res	558	246	220	1.129057	0.949276	-1.00882	1.008815626	0.012662528	-0.01266	0.99982	1902	1577	1635	1704.667	2135	1758	1881	1924.667
rfaB	162	44	-81.3333	-1.13083	0.047885	-1.29395	1.293950296	0.3717822	-0.37178	0.151677	648	739	722	703	666	622	577	621.6667
rfaC	1375	107	-696.667	-1.32651	0.012135	-1.52153	1.52152839	0.605521253	-0.60552	0.053512	2262	3405	2824	2830.333	2216	2030	2155	2133.667
rfaD	1676	442	-1018.67	-1.12713	0.124521	-1.28152	1.281524489	0.357861048	-0.35786	0.289178	9427	9252	8416	9031.667	8314	7974	7751	8013
rfaE	199	80	114.3333	1.112349	0.840094	-1.02598	1.025977841	0.036999573	-0.037	0.938119	1035	1001	1017	1017.667	1200	1097	1099	1132
rfaF	1631	451	-801.667	-1.19992	0.049044	-1.3708	1.370803914	0.455022217	-0.45502	0.153873	4475	5378	4582	4811.667	4259	3747	4024	4010
rfaG	254	24	98.33333	1.1295	0.905603	-1.01649	1.016491499	0.02359815	-0.0236	0.974909	662	796	820	759.3333	916	840	817	857.6667
rfaH	51	17	15	1.02422	0.384998	-1.11584	1.115843906	0.158135225	-0.15814	0.601737	591	629	638	619.3333	642	621	640	634.3333
rfaI	220	85	-80	-1.10046	0.062899	-1.25161	1.251607191	0.323781852	-0.32378	0.184451	952	877	800	876.3333	871	732	786	796.3333
rfaJ	225	95	-81.6667	-1.10894	0.071292	-1.25648	1.256484966	0.329393409	-0.32939	0.201207	873	923	698	831.3333	793	758	698	749.6667
rfaK	785	512	-642.333	-1.6802	1.76E-06	-1.91924	1.919239028	0.940534401	-0.94053	4.28E-05	1471	1674	1615	1586.667	959	985	889	944.3333
rfaL	1678	126	-1083.33	-1.79095	0.000182	-2.07649	2.076491344	1.054147858	-1.05415	0.001921	1522	2873	2964	2453	1427	1396	1286	1369.667
rfaP	55	28	-12.6667	-1.02299	0.220218	-1.16778	1.167782227	0.22377126	-0.22377	0.424256	535	590	566	563.6667	544	538	571	551
rfaQ	132	31	28	1.02512	0.401858	-1.11468	1.114675959	0.156624374	-0.15662	0.616776	1043	1175	1126	1114.667	1151	1120	1157	1142.667
rfaY	310	90	-180.333	-1.34613	0.000768	-1.5289	1.528897219	0.612491424	-0.61249	0.00619	696	809	599	701.3333	555	509	499	521
rfaZ	404	37	-226.667	-1.27146	0.004863	-1.44001	1.440014006	0.526082844	-0.52608	0.026181	1087	1214	884	1061.667	847	848	810	835
rfbA	540	181	-167.333	-1.14351	0.076185	-1.32151	1.321507848	0.402184993	-0.40218	0.20995	1000	1460	1540	1333.333	1280	1119	1099	1166
rfbB	562	254	379	1.186363	0.805976	1.037952	1.037951886	0.05373957	0.05374	0.911195	1890	2108	2103	2033.667	2357	2429	2452	2412.667
rfbC	626	97	-292.333	-1.30859	0.010342	-1.51613	1.516133496	0.600396789	-0.6004	0.04734	920	1285	1514	1239.667	1017	888	937	947.3333
rfbD	420	26	161	1.101173	0.777043	-1.04123	1.041233455	0.058293572	-0.05829	0.892069	1386	1707	1681	1591.333	1757	1694	1806	1752.333
rfbE	7053	2803	-5222	-2.72022	1.28E-08	-3.13029	3.130287807	1.646295308	-1.6463	6.01E-07	5874	9686	9213	8257.667	3071	3403	2633	3035.667
rfbF	1696	734	-1153.33	-1.64241	9.34E-05	-1.88785	1.887854512	0.916747588	-0.91675	0.001124	2488	3322	3036	2948.667	2006	1626	1754	1795.333
rfbG	2671	1278	-1812.67	-1.5964	0.000242	-1.82327	1.823266398	0.866525369	-0.86653	0.00235	4357	5586	4613	4852	3124	2915	3079	3039.333
rfbH	7340	3055	-5157.33	-2.01019	4.15E-06	-2.30516	2.305164455	1.204869679	-1.20487	8.89E-05	8161	12263	10364	10262.67	5287	5106	4923	5105.333
rfbI	754	167	-488	-1.4219	0.001321	-1.63976	1.639755801	0.713480979	-0.71348	0.009503	1298	1785	1851	1644.667	1242	1097	1131	1156.667
rfbIB	29	7	17.66667	2.12766	0.003254	1.862423	1.862423382	0.897181076	0.897181	0.019354	17	12	18	15.66667	41	35	24	33.33333
rfbK	2130	974	-1573.33	-1.53129	0.000952	-1.75316	1.753157458	0.809955576	-0.80996	0.007412	3943	4841	4820	4534.667	2969	3204	2711	2961.333
rfbM	2241	1183	-1686.33	-1.63595	0.00022	-1.87217	1.872173309	0.904713993	-0.90471	0.002173	3824	4551	4639	4338	2641	2916	2398	2651.667
rfbN	1495	723	-1056.33	-1.61882	0.000142	-1.85532	1.855316659	0.891665442	-0.89167	0.001582	2446	2803	3041	2763.333	1723	1852	1546	1707
rfbP	1352	508	-936.333	-1.42139	0.00306	-1.62701	1.627006589	0.702220094	-0.70222	0.018421	2768	3399	3308	3158.333	2260	2359	2047	2222
rfbS	1573	688	-1062	-1.88994	3.82E-07	-2.15872	2.158717623	1.11017454	-1.11017	1.18E-05	1910	2688	2168	2255.333	1222	1243	1115	1193.333
rfbU	1773	1092	-1376.67	-1.78235	3.51E-05	-2.03512	2.035119595	1.025113578	-1.02511	0.000524	2726	3347	3336	3136.333	1634	2071	1574	1759.667
rfbV	2748	1608	-2040.67	-1.9814	3.58E-06	-2.25939	2.259385118	1.175930202	-1.17593	7.98E-05	3524	4646	4190	4120	1916	2424	1898	2079.333
rfbX	1403	585	-911.333	-1.83277	5.67E-07	-2.07281	2.072814451	1.051590979	-1.05159	1.67E-05	1955	2413	1649	2005.667	1064	1209	1010	1094.333

Feature ID	Experiment - Range (original values)	Experiment - IQR (original values)	Experiment - Difference (original values)	Experiment - Fold Change (original values)	EDGE test: yccT NT vs WT NT , tagwise dispersions - P-value	EDGE test: yccT NT vs WT NT , tagwise dispersions - Fold change	yccT NT vs WT NT ABS|FC|	yccT NT vs WT NT Log2FC	yccT NT vs WT NT Log2FC +-	EDGE test: yccT NT vs WT NT , tagwise dispersions - FDR p-value correction	WT NT - WT.1.S22 - Expression values	WT NT - WT.2.S23 - Expression values	WT NT - WT.3.S24 - Expression values	WT NT - Means	yccT NT - yccT.1.S28 - Expression values	yccT NT - yccT.2.S29 - Expression values	yccT NT - yccT.3.S30 - Expression values	yccT NT - Means
rfc	0	0	0	1	1	1	1	0	0	1	0	0	0	0	0	0	0	0
rfe	558	154	279	1.155866	0.893791	1.018378	1.018378474	0.026273829	0.026274	0.968783	1913	1857	1600	1790	2158	2038	2011	2069
rffG	736	85	-427.667	-1.44135	0.000733	-1.66257	1.662570937	0.733415898	-0.73342	0.005982	1096	1575	1519	1396.667	1057	839	1011	969
rhaA	26	7	-7.33333	-1.18644	0.079277	-1.34591	1.345910912	0.428582919	-0.42858	0.216704	43	61	36	46.66667	39	35	44	39.33333
rhaB	19	11	-5	-1.04478	0.177255	-1.1933	1.193297655	0.254953952	-0.25495	0.36497	104	123	123	116.6667	116	114	105	111.6667
rhaD	33	9	10	1.103093	0.858818	-1.02783	1.0278255	0.039595351	-0.0396	0.949694	102	100	89	97	106	122	93	107
rhaR	112	70	48	1.135211	0.990375	-1.00232	1.002315896	0.003337269	-0.00334	1	323	422	320	355	384	393	432	403
rhaS	199	171	144	1.644776	0.006303	1.461906	1.461905998	0.547850548	0.547851	0.032214	311	178	181	223.3333	377	352	373	367.3333
rhaT	33	18	24	1.298755	0.312214	1.138935	1.138934609	0.187684919	0.187685	0.527084	74	84	83	80.33333	105	101	107	104.3333
rhlB	617	258	-163.333	-1.03251	0.299794	-1.18097	1.180970807	0.239973303	-0.23997	0.515478	5065	5231	5264	5186.667	5357	4740	4973	5023.333
rhlE	137	15	68.66667	1.220321	0.560455	1.071454	1.071453693	0.0995695	0.099569	0.74464	324	327	284	311.6667	421	339	381	380.3333
rho	6962	4699	5306.667	1.343755	0.318529	1.181305	1.181304975	0.24038147	0.240381	0.533578	16987	14659	14666	15437.33	21621	19365	21246	20744
rhtC	162	47	56	1.128637	0.976297	-1.0044	1.004395293	0.006327173	-0.00633	1	496	438	372	435.3333	534	455	485	491.3333
rhuM	519	128	99	1.074399	0.715034	-1.04949	1.049485873	0.069682746	-0.06968	0.850783	1606	1299	1087	1330.667	1454	1427	1408	1429.667
ribA	610	207	204.3333	1.107506	0.87103	-1.02284	1.022839714	0.032580083	-0.03258	0.957463	2212	1832	1658	1900.667	2268	2008	2039	2105
ribB	1832	143	-471	-1.20108	0.078258	-1.34639	1.346394115	0.429100775	-0.4291	0.214712	3953	2366	2121	2813.333	2459	2223	2345	2342.333
ribD	618	444	307.6667	1.291535	0.317676	1.145152	1.14515248	0.195539709	0.19554	0.532988	1378	934	854	1055.333	1472	1213	1404	1363
ribE	97	23	21	1.026923	0.403401	-1.11068	1.110684137	0.151448593	-0.15145	0.617647	793	770	777	780	851	798	754	801
ribF	536	91	199.6667	1.110191	0.873438	-1.02187	1.021871202	0.031213368	-0.03121	0.958007	1974	1883	1579	1812	2115	1894	2026	2011.667
ribH	78	35	-18.3333	-1.04215	0.156052	-1.18751	1.18750673	0.24793569	-0.24794	0.33372	460	470	430	453.3333	479	425	401	435
rihC	548	224	295.6667	1.264224	0.376808	1.116419	1.116418693	0.158878186	0.158878	0.594928	1274	1129	954	1119	1502	1353	1389	1414.667
rimI	98	31	-35	-1.10038	0.080524	-1.26139	1.261388927	0.335013174	-0.33501	0.218628	327	425	399	383.6667	364	333	349	348.6667
rimJ	1046	332	134	1.033422	0.56253	-1.09455	1.094549707	0.130337474	-0.13034	0.745608	4498	4078	3452	4009.333	4254	4254	3922	4143.333
rimK	151	36	84.33333	1.191088	0.734916	1.043881	1.043880701	0.061956844	0.061957	0.865693	459	413	452	441.3333	525	488	564	525.6667
rimL	155	57	64	1.154714	0.911963	1.015217	1.015216931	0.021788035	0.021788	0.979221	478	343	420	413.6667	498	477	458	477.6667
rimM	12927	4225	-7651	-2.65534	4.9E-08	-3.0392	3.039202948	1.603693017	-1.60369	1.96E-06	8784	17307	10728	12273	4927	4380	4559	4622
rlpA	312	74	156.6667	1.157824	0.898341	1.016635	1.016635384	0.02380235	0.023802	0.971085	1065	991	922	992.6667	1221	993	1234	1149.333
rlpB	547	134	257.3333	1.142068	0.988236	1.002217	1.002216834	0.003194676	0.003195	1	1894	1822	1718	1811.333	2265	1956	1985	2068.667
rluA	183	38	69	1.112807	0.876187	-1.01947	1.019468454	0.027817135	-0.02782	0.959152	669	631	535	611.6667	718	647	677	680.6667
rluC	115	58	69.66667	1.200384	0.65887	1.054013	1.054013393	0.075893199	0.075893	0.816174	374	329	340	347.6667	444	398	410	417.3333
rluD	417	137	85	1.055471	0.585339	-1.07375	1.073749508	0.102657471	-0.10266	0.764059	1754	1506	1337	1532.333	1703	1506	1643	1617.333
rmbA	8	2	3	1.5625	0.450184	1.367279	1.367278725	0.451307372	0.451307	0.659356	7	5	4	5.333333	12	6	7	8.333333
rmf	1765	1432	974.6667	1.792197	0.035632	1.623926	1.623925532	0.699485477	0.699485	0.121825	2249	696	746	1230.333	2178	1976	2461	2205
rna	964	637	530.6667	1.286074	0.362933	1.140319	1.140319228	0.189437758	0.189438	0.579307	2339	1702	1524	1855	2488	2239	2430	2385.667
rna-AM93	13	4	7.333333	1.758621	0.094323	1.544643	1.544643125	0.627273556	0.627274	0.240751	11	12	6	9.666667	15	17	19	17
rna-AM93	121	91	72.33333	1.226514	0.516535	1.082509	1.082509227	0.114379323	0.114379	0.715467	381	285	292	319.3333	406	383	386	391.6667

Feature ID	Experiment - Range (original values)	Experiment - IQR (original values)	Experiment - Difference (original values)	Experiment - Fold Change (original values)	EDGE test: yccT NT vs WT NT , tagwise dispersions - P-value	EDGE test: yccT NT vs WT NT , tagwise dispersions - Fold change	yccT NT vs WT NT ABS|FC|	yccT NT vs WT NT Log2FC	yccT NT vs WT NT Log2FC +-	EDGE test: yccT NT vs WT NT , tagwise dispersions - FDR p-value correction	WT NT - WT.1.S22 - Expression values	WT NT - WT.2.S23 - Expression values	WT NT - WT.3.S24 - Expression values	WT NT - Means	yccT NT - yccT.1.S28 - Expression values	yccT NT - yccT.2.S29 - Expression values	yccT NT - yccT.3.S30 - Expression values	yccT NT - Means
rna-AM93	372	98	110.6667	1.145232	0.900069	1.016896	1.016896107	0.024172291	0.024172	0.971561	934	772	580	762	870	796	952	872.6667
rna-AM93	223	145	112.3333	1.251118	0.412782	1.11173	1.11173029	0.152806827	0.152807	0.625818	569	411	362	447.3333	538	556	585	559.6667
rna-AM93	111	46	39	1.164095	0.805098	1.031828	1.031828141	0.045202699	0.045203	0.911195	291	242	180	237.6667	288	252	290	276.6667
rna-AM93	218	23	71.66667	1.157164	0.860801	1.022658	1.022658281	0.032324153	0.032324	0.950937	502	493	373	456	513	479	591	527.6667
rna-AM93	18	5	9	1.9	0.047765	1.67379	1.673789573	0.743118165	0.743118	0.151609	15	10	5	10	22	23	12	19
rna-AM93	34	14	20.66667	1.659574	0.013331	1.461816	1.46181648	0.547762203	0.547762	0.057629	36	35	23	31.33333	57	50	49	52
rna-AM93	8	1	3.333333	1.208333	0.858773	1.063261	1.063261452	0.088496393	0.088496	0.949694	17	15	16	16	17	18	23	19.33333
rna-AM93	12	4	4	1.631579	0.297181	1.439011	1.439010839	0.525077459	0.525077	0.512976	12	6	1	6.333333	8	10	13	10.33333
rna-AM93	6	3	-2	-1.15385	0.316325	-1.30613	1.306134985	0.385304003	-0.3853	0.531326	15	17	13	15	11	16	12	13
rna-AM93	107	62	39.33333	1.25486	0.478716	1.112581	1.112581215	0.153910652	0.153911	0.684774	216	109	138	154.3333	210	200	171	193.6667
rna-AM93	47	23	14.33333	1.146259	0.942262	1.013115	1.013115145	0.018798152	0.018798	0.995984	126	79	89	98	113	112	112	112.3333
rna-AM93	9	2	4.333333	2.625	0.075194	2.254444	2.254443699	1.172771481	1.172771	0.208109	4	2	2	2.666667	8	2	11	7
rna-AM93	33	17	14	1.857143	0.052357	1.649453	1.64945314	0.721987792	0.721988	0.161108	29	12	8	16.33333	31	19	41	30.33333
rna-AM93	14	5	6.666667	2.25	0.053847	1.954619	1.954618959	0.966887391	0.966887	0.164497	5	2	9	5.333333	10	10	16	12
rna-AM93	37	13	21.33333	1.64	0.022004	1.441401	1.441401494	0.527472246	0.527472	0.083802	41	30	29	33.33333	66	43	55	54.66667
rna-AM93	18	6	6.666667	1.47619	0.280816	1.309147	1.309147133	0.388627249	0.388627	0.495537	23	14	5	14	20	20	22	20.66667
rna-AM93	84	23	32	1.446512	0.168746	1.274542	1.274542482	0.349979461	0.349979	0.353697	87	64	64	71.66667	92	71	148	103.6667
rna-AM93	18	6	9	1.254717	0.507398	1.107236	1.107235786	0.146962477	0.146962	0.707446	42	36	28	35.33333	42	46	45	44.33333
rna-AM93	91	9	30.66667	1.363636	0.26016	1.220987	1.220986766	0.288047563	0.288048	0.471672	103	106	44	84.33333	113	135	97	115
rna-AM93	706	121	308.6667	1.256794	0.423054	1.11436	1.114359777	0.15621509	0.156215	0.635442	1349	1321	936	1202	1442	1448	1642	1510.667
rna-AM93	210	57	104.3333	1.377563	0.115644	1.224042	1.224042114	0.291653195	0.291653	0.276385	327	296	206	276.3333	353	373	416	380.6667
rna-AM93	14	8	6.666667	1.298507	0.481215	1.144195	1.144194666	0.194332524	0.194333	0.686713	29	17	21	22.33333	30	31	26	29
rna-AM93	0	0	0	1	1	1	1	0	0	1	0	0	0	0	0	0	0	0
rna-AM93	88	38	4.333333	1.019939	0.441755	-1.10733	1.107334724	0.147091384	-0.14709	0.652901	239	243	170	217.3333	201	206	258	221.6667
rna-AM93	469	278	197	1.231492	0.505579	1.094575	1.094575052	0.13037088	0.130371	0.706593	1104	799	650	851	1077	948	1119	1048
rna-AM93	715	306	379	1.439675	0.080688	1.285538	1.285538394	0.362372698	0.362373	0.21877	1109	894	583	862	1225	1200	1298	1241
rna-AM93	220	65	109	1.326347	0.20003	1.177842	1.177842299	0.23614639	0.236146	0.3973	394	367	241	334	461	436	432	443
rna-AM93	0	0	0	1	1	1	1	0	0	1	0	0	0	0	0	0	0	0
rna-AM93	0	0	0	1	1	1	1	0	0	1	0	0	0	0	0	0	0	0
rna-AM93	5	1	1.666667	1.555556	0.686372	1.355942	1.355942132	0.43929561	0.439296	0.833008	3	4	2	3	4	3	7	4.666667
rna-AM93	33	14	16.66667	1.684932	0.043305	1.484458	1.484457515	0.569935804	0.569936	0.141091	35	21	17	24.33333	50	27	46	41
rna-AM93	55	15	26.66667	1.615385	0.027913	1.433986	1.43398598	0.520030919	0.520031	0.101547	56	48	26	43.33333	63	66	81	70
rna-AM93	53	19	23.66667	1.275194	0.353973	1.129608	1.129607551	0.175821638	0.175822	0.568899	105	86	67	86	111	120	98	109.6667
rna-AM93	43	2	16.33333	1.385827	0.287244	1.211114	1.211113647	0.276334249	0.276334	0.503523	49	27	51	42.33333	70	51	55	58.66667
rna-AM93	14	5	8.666667	1.298851	0.457426	1.144473	1.144472681	0.194683026	0.194683	0.665853	32	30	25	29	35	39	39	37.66667
rna-AM93	7	2	-1	-1.42857	0.468371	-1.5864	1.586398366	0.665755097	-0.66576	0.676072	7	0	3	3.333333	2	1	4	2.333333

Feature ID	Experiment - Range (original values)	Experiment - IQR (original values)	Experiment - Difference (original values)	Experiment - Fold Change (original values)	EDGE test: yccT NT vs WT NT , tagwise dispersions - P-value	EDGE test: yccT NT vs WT NT , tagwise dispersions - Fold change	yccT NT vs WT NT ABS|FC|	yccT NT vs WT NT Log2FC	yccT NT vs WT NT Log2FC +-	EDGE test: yccT NT vs WT NT , tagwise dispersions - FDR p-value correction	WT NT - WT.1.S22 - Expression values	WT NT - WT.2.S23 - Expression values	WT NT - WT.3.S24 - Expression values	WT NT - Means	yccT NT - yccT.1.S28 - Expression values	yccT NT - yccT.2.S29 - Expression values	yccT NT - yccT.3.S30 - Expression values	yccT NT - Means
rna-AM93	157	11	4.333333	1.01357	0.428197	-1.10898	1.108976477	0.149228764	-0.14923	0.639913	389	337	232	319.3333	317	326	328	323.6667
rna-AM93	318	156	183	1.30585	0.241504	1.155787	1.155786709	0.208875185	0.208875	0.451668	695	608	492	598.3333	810	770	764	781.3333
rna-AM93	1	0	0.333333	#DIV/0!	1	3.506332	3.506332206	1.80996269	1.809963	1	0	0	0	0	1	0	0	0.333333
rna-AM93	0	0	0	1	1	1	1	0	0	1	0	0	0	0	0	0	0	0
rna-AM93	28	3	10.33333	1.206667	0.758104	1.060604	1.060604308	0.084886514	0.084887	0.880907	45	53	52	50	58	73	50	60.33333
rna-AM93	22	12	11.66667	1.625	0.072233	1.430275	1.43027544	0.516293006	0.516293	0.202957	27	14	15	18.66667	36	26	29	30.33333
rna-AM93	21	1	-8	-1.13953	0.085479	-1.29755	1.297549686	0.375789783	-0.37579	0.227209	71	52	73	65.33333	57	58	57	57.33333
rna-AM93	14	5	2	1.111111	1	-1.01878	1.018780255	0.026842905	-0.02684	1	24	19	11	18	25	20	15	20
rna-AM93	0	0	0	1	1	1	1	0	0	1	0	0	0	0	0	0	0	0
rna-AM93	12	4	7.333333	2.692308	0.012057	2.33862	2.338620484	1.225657757	1.225658	0.053372	6	4	3	4.333333	12	15	8	11.66667
rna-AM93	31	12	9.666667	1.113725	0.887189	-1.02389	1.023890086	0.034060852	-0.03406	0.965169	90	77	88	85	98	78	108	94.66667
rna-AM93	53	8	21	1.161954	0.839001	1.025807	1.025806912	0.036759197	0.036759	0.937824	144	137	108	129.6667	161	155	136	150.6667
rna-AM93	284	45	6.666667	1.007927	0.362056	-1.12005	1.120048432	0.163561117	-0.16356	0.578532	958	891	674	841	846	826	871	847.6667
rna-AM93	284	100	109.3333	1.200244	0.621095	1.067092	1.067092164	0.093684786	0.093685	0.787112	657	556	425	546	601	656	709	655.3333
rna-AM93	70	30	20.33333	1.111111	0.899396	-1.01691	1.016907396	0.024188306	-0.02419	0.971377	208	183	158	183	176	206	228	203.3333
rna-AM93	0	0	0	1	1	1	1	0	0	1	0	0	0	0	0	0	0	0
rna-AM93	0	0	0	1	1	1	1	0	0	1	0	0	0	0	0	0	0	0
rna-AM93	0	0	0	1	1	1	1	0	0	1	0	0	0	0	0	0	0	0
rna-AM93	0	0	0	1	1	1	1	0	0	1	0	0	0	0	0	0	0	0
rna-AM93	11	4	6	2	0.071006	1.746696	1.746695634	0.804628237	0.804628	0.200528	4	9	5	6	12	15	9	12
rna-AM93	547	144	198.6667	1.201829	0.625957	1.070439	1.070439027	0.098202621	0.098203	0.789419	1212	1063	678	984.3333	1207	1117	1225	1183
rna-AM93	246	81	126.3333	1.123856	0.930399	-1.01103	1.011032479	0.015829344	-0.01583	0.988159	1097	1016	947	1020	1155	1091	1193	1146.333
rna-AM93	474	122	229	1.437301	0.108911	1.284635	1.284635118	0.361358641	0.361359	0.265887	599	656	316	523.6667	721	790	747	752.6667
rna-AM93	1	0	-0.33333	#DIV/0!	1	-3.8476	3.847601003	1.943959199	-1.94396	1	0	1	0	0.333333	0	0	0	0
rna-AM93	9	6	-1	-1.03125	0.394222	-1.17196	1.17196153	0.228925214	-0.22893	0.610555	36	28	35	33	30	29	37	32
rna-AM93	120	46	35.33333	1.034823	0.450344	-1.09867	1.098667738	0.135755149	-0.13576	0.659356	1073	1018	953	1014.667	1062	1016	1072	1050
rna-AM93	340	6	130	1.161424	0.832852	1.026935	1.026935309	0.038345302	0.038345	0.933318	890	884	642	805.3333	982	888	936	935.3333
rna-AM93	93	36	18.66667	1.094435	0.773811	-1.04012	1.040117096	0.056745956	-0.05675	0.889719	231	164	198	197.6667	214	178	257	216.3333
rna-AM93	34	12	13.33333	1.408163	0.244541	1.241931	1.241931325	0.3125854	0.312585	0.455235	45	22	31	32.66667	43	39	56	46
rna-AM93	89	23	25.33333	1.171558	0.763007	1.043968	1.043968083	0.062077606	0.062078	0.883198	188	156	99	147.6667	178	186	155	173
rna-AM93	177	28	74.33333	1.242128	0.445366	1.105685	1.105684634	0.144939955	0.14494	0.655966	364	340	217	307	368	394	382	381.3333
rna-AM93	63	5	0.333333	1.003356	0.437488	-1.12036	1.12036262	0.163965755	-0.16397	0.648548	130	101	67	99.33333	101	102	96	99.66667
rna-AM93	16	3	5.666667	1.333333	0.492029	1.176964	1.17696359	0.23506969	0.23507	0.696989	19	16	16	17	13	26	29	22.66667
rna-AM93	25	7	3.666667	1.036913	0.461412	-1.10217	1.102172119	0.140349538	-0.14035	0.66967	88	108	102	99.33333	113	95	101	103
rna-AM93	48	12	3.666667	1.020992	0.393451	-1.11091	1.110907805	0.151739091	-0.15174	0.61	180	193	151	174.6667	168	168	199	178.3333
rna-AM93	41	5	8	1.065574	0.617291	-1.06376	1.063759052	0.089171409	-0.08917	0.78418	127	139	100	122	127	122	141	130

Feature ID	Experiment - Range (original values)	Experiment - IQR (original values)	Experiment - Difference (original values)	Experiment - Fold Change (original values)	EDGE test: yccT NT vs WT NT , tagwise dispersions - P-value	EDGE test: yccT NT vs WT NT , tagwise dispersions - Fold change	yccT NT vs WT NT ABS|FC|	yccT NT vs WT NT Log2FC	yccT NT vs WT NT Log2FC +-	EDGE test: yccT NT vs WT NT , tagwise dispersions - FDR p-value correction	WT NT - WT.1.S22 - Expression values	WT NT - WT.2.S23 - Expression values	WT NT - WT.3.S24 - Expression values	WT NT - Means	yccT NT - yccT.1.S28 - Expression values	yccT NT - yccT.2.S29 - Expression values	yccT NT - yccT.3.S30 - Expression values	yccT NT - Means
rna-AM93	53	7	8	1.034582	0.426956	-1.09904	1.099040653	0.136244752	-0.13624	0.639352	229	259	206	231.3333	255	235	228	239.3333
rna-AM93	47	5	17	1.102616	0.815489	-1.02916	1.029160867	0.041468506	-0.04147	0.918821	173	178	146	165.6667	179	176	193	182.6667
rna-AM93	146	15	13.66667	1.040877	0.520895	-1.08633	1.086327254	0.119458778	-0.11946	0.717243	349	400	254	334.3333	366	344	334	348
rna-AM93	16	3	5.666667	1.326923	0.536104	1.163133	1.163132821	0.218015851	0.218016	0.726494	19	11	22	17.33333	27	23	19	23
rna-AM93	155	33	86	1.078827	0.671054	-1.05448	1.054483358	0.076536327	-0.07654	0.822756	1118	1116	1039	1091	1194	1188	1149	1177
rna-AM93	134	68	36	1.026852	0.434613	-1.11005	1.110050557	0.150625386	-0.15063	0.645366	1304	1385	1333	1340.667	1380	1438	1312	1376.667
rna-AM93	101	14	53.66667	1.098111	0.778683	-1.03552	1.035522183	0.050358459	-0.05036	0.892946	563	553	525	547	609	626	567	600.6667
rna-AM93	97	11	-26.3333	-1.17753	0.052235	-1.36121	1.361212792	0.444892614	-0.44489	0.160955	151	147	226	174.6667	158	129	158	148.3333
rna-AM93	16	6	2	1.071429	0.806278	-1.05983	1.059833548	0.0838377	-0.08384	0.911195	30	24	30	28	30	38	22	30
rna-AM93	38	31	29	2.191781	0.000126	1.93584	1.935840473	0.95296007	0.95296	0.001431	36	19	18	24.33333	50	54	56	53.33333
rnb	768	321	478	1.293432	0.339614	1.138569	1.138568826	0.187221504	0.187222	0.553593	1762	1630	1495	1629	2107	1951	2263	2107
rnc	291	217	227	1.355428	0.193373	1.185874	1.185873976	0.245950701	0.245951	0.387008	642	583	691	638.6667	864	874	859	865.6667
rnd	186	136	145.6667	1.559539	0.016464	1.364531	1.364531175	0.448405356	0.448405	0.067541	267	226	288	260.3333	403	403	412	406
rne	6198	386	-3156.33	-1.33912	0.021833	-1.54943	1.549432254	0.631739677	-0.63174	0.08347	9446	13237	14708	12463.67	9832	8510	9580	9307.333
rnfA	305	197	197.6667	1.412665	0.070153	1.247553	1.247553106	0.31910123	0.319101	0.199134	563	457	417	479	722	654	654	676.6667
rnfB	77	49	48.33333	1.328798	0.167258	1.175383	1.175382528	0.233130358	0.23313	0.351801	176	144	121	147	198	193	195	195.3333
rnfC	153	66	63.66667	1.143178	0.989998	-1.00194	1.001938677	0.002794212	-0.00279	1	396	502	436	444.6667	549	442	534	508.3333
rnfD	61	30	8.333333	1.051867	0.535005	-1.08445	1.084446718	0.116959171	-0.11696	0.726399	162	175	145	160.6667	188	129	190	169
rnfE	90	34	50.66667	1.363636	0.116102	1.204663	1.204663151	0.268629795	0.26863	0.276734	164	142	112	139.3333	202	176	192	190
rnfG	75	32	30.33333	1.188797	0.675145	1.050752	1.050751517	0.071421539	0.071422	0.825117	192	160	130	160.6667	197	171	205	191
rnhA	85	13	55	1.186863	0.742634	1.042244	1.042244024	0.0596931	0.059693	0.872147	296	300	287	294.3333	372	367	309	349.3333
rnhB	142	18	10.33333	1.016667	0.343472	-1.12543	1.12543107	0.170477698	-0.17048	0.557685	611	629	620	620	699	557	635	630.3333
rnk	216	53	98	1.232227	0.487954	1.089334	1.089334481	0.123447003	0.123447	0.692606	477	434	355	422	502	487	571	520
rnpA	176	86	53	1.06085	0.564036	-1.07481	1.074807236	0.10407794	-0.10408	0.745911	872	927	814	871	990	841	941	924
rnt	159	90	104	1.174302	0.794322	1.032638	1.032637841	0.046334372	0.046334	0.903705	652	572	566	596.6667	725	662	715	700.6667
rob	1525	757	731.3333	1.237112	0.553615	1.096709	1.096709326	0.133181202	0.133181	0.739768	3760	2982	2511	3084.333	4036	3739	3672	3815.667
rof	622	270	193.6667	1.098009	0.847059	-1.02796	1.027957199	0.039780196	-0.03978	0.942334	2344	1862	1722	1976	2132	2283	2094	2169.667
rpe	409	95	-202.667	-1.15338	0.035974	-1.31468	1.314682935	0.394714903	-0.39471	0.12276	1514	1640	1418	1524	1410	1231	1323	1321.333
rph	81	22	38.33333	1.116989	0.894017	-1.01663	1.016630606	0.02379557	-0.0238	0.968792	335	357	291	327.6667	369	357	372	366
rpiA	1347	470	-956	-1.3925	0.004764	-1.60101	1.601007351	0.678979932	-0.67898	0.025804	2855	3603	3717	3391.667	2552	2385	2370	2435.667
rplA	12219	4863	-7181.33	-2.20722	1.17E-06	-2.51573	2.515731126	1.330977739	-1.33098	3.11E-05	10846	17767	10777	13130	6384	5548	5914	5948.667
rplB	15335	6662	-9774.67	-3.43434	5.1E-12	-3.922	3.921998792	1.971589091	-1.97159	4.11E-10	10829	18958	11583	13790	4256	3623	4167	4015.333
rplC	6349	2205	-3458.67	-1.67521	0.000222	-1.9189	1.91890245	0.940281372	-0.94028	0.002178	7499	10528	7716	8581	5894	4179	5294	5122.333
rplD	4091	1552	-2508	-2.03196	1.32E-06	-2.32603	2.326034668	1.217872599	-1.21787	3.4E-05	4158	6161	4496	4938.333	2615	2070	2606	2430.333
rplE	5267	1174	-2761.67	-1.23922	0.0436	-1.42451	1.424508962	0.510464699	-0.51046	0.141947	12371	15621	14927	14306.33	12727	10354	11553	11544.67
rplF	7118	2501	-4015	-1.62888	0.000398	-1.85621	1.856211066	0.892360766	-0.89236	0.0036	9430	12955	8813	10399.33	7004	5837	6312	6384.333

Feature ID	Experiment - Range (original values)	Experiment - IQR (original values)	Experiment - Difference (original values)	Experiment - Fold Change (original values)	EDGE test: yccT NT vs WT NT , tagwise dispersions - P-value	EDGE test: yccT NT vs WT NT , tagwise dispersions - Fold change	yccT NT vs WT NT ABS|FC|	yccT NT vs WT NT Log2FC	yccT NT vs WT NT Log2FC +-	EDGE test: yccT NT vs WT NT , tagwise dispersions - FDR p-value correction	WT NT - WT.1.S22 - Expression values	WT NT - WT.2.S23 - Expression values	WT NT - WT.3.S24 - Expression values	WT NT - Means	yccT NT - yccT.1.S28 - Expression values	yccT NT - yccT.2.S29 - Expression values	yccT NT - yccT.3.S30 - Expression values	yccT NT - Means
rplI	6092	745	-3676.67	-1.89953	5E-05	-2.11919	2.119188418	1.083511864	-1.08351	0.000689	9978	8487	4827	7764	4082	4294	3886	4087.333
rplJ	37962	12489	-24013.3	-3.31417	5.39E-10	-3.81263	3.812631756	1.930787195	-1.93079	3.27E-08	22970	47368	32832	34390	11243	9406	10481	10376.67
rplK	3946	1541	-2323.67	-1.83166	2.29E-05	-2.08641	2.086413572	1.061025159	-1.06103	0.000371	4448	6591	4314	5117.667	2964	2645	2773	2794
rplL	32812	11277	-22051.7	-3.78876	1.61E-11	-4.36965	4.3696545	2.127519213	-2.12752	1.23E-09	19248	40098	30531	29959	8465	7286	7971	7907.333
rplM	2965	1101	-2043	-1.34171	0.011688	-1.54151	1.541509812	0.624344073	-0.62434	0.052101	6872	8682	8511	8021.667	6448	5717	5771	5978.667
rplN	3801	487	-1539.67	-1.21597	0.055034	-1.39259	1.392591924	0.477772562	-0.47777	0.166683	7426	10297	8283	8668.667	7689	6496	7202	7129
rplO	12934	4098	-7749	-3.08531	3.24E-10	-3.49532	3.495317485	1.805423504	-1.80542	2.14E-08	10223	16297	7875	11465	4008	3363	3777	3716
rplP	16589	7787	-10949.7	-6.64319	0	-7.55862	7.558621283	2.918123106	-2.91812	0	9729	18484	10457	12890	1895	1984	1942	1940.333
rplQ	5810	1887	-3478.67	-1.95734	1.04E-05	-2.24207	2.242073934	1.164833853	-1.16483	0.000188	5563	9173	6601	7112.333	3862	3363	3676	3633.667
rplR	4735	1731	-2847.67	-2.04899	1.54E-06	-2.32945	2.329446828	1.2199874	-1.21999	3.86E-05	5012	7255	4420	5562.333	2935	2520	2689	2714.667
rplS	9593	3385	-5858	-4.16136	1.68E-13	-4.76599	4.765986046	2.252774728	-2.25277	1.96E-11	5347	11221	6565	7711	1962	1628	1969	1853
rplT	11044	3282	-7343	-1.79173	0.000111	-2.06457	2.064568258	1.045840117	-1.04584	0.001288	12792	19399	17662	16617.67	9959	8355	9510	9274.667
rplU	3737	881	-2493.33	-1.51519	0.002422	-1.74778	1.747781517	0.805524851	-0.80552	0.015096	5575	8327	8097	7333	5235	4590	4694	4839.667
rplV	9904	4475	-6556.33	-4.99858	0	-5.71014	5.710139759	2.513526057	-2.51353	0	6134	11456	6998	8196	1708	1552	1659	1639.667
rplW	3596	1493	-2264.33	-2.50721	2.22E-09	-2.8698	2.86979967	1.520950031	-1.52095	1.24E-07	3093	4865	3342	3766.667	1638	1269	1600	1502.333
rplX	2835	903	-1288.33	-1.17397	0.080319	-1.34493	1.344934146	0.427535534	-0.42754	0.218296	7700	9785	8597	8694	8085	6950	7182	7405.667
rplY	2685	125	-1476.67	-1.66576	0.001076	-1.92859	1.928591275	0.947547427	-0.94755	0.008089	2335	4672	4077	3694.667	2396	1987	2271	2218
rpmA	4266	1118	-2552	-1.58563	0.000965	-1.82525	1.825248027	0.86809252	-0.86809	0.007449	5307	8302	7120	6909.667	4848	4036	4189	4357.667
rpmB	3318	733	-1955	-1.55127	0.00129	-1.78303	1.783026735	0.834328335	-0.83433	0.009358	4213	6688	5603	5501.333	3789	3370	3480	3546.333
rpmC	6538	3055	-4221	-6.89525	0	-7.83045	7.830451676	2.969095527	-2.9691	0	3757	7238	3816	4937	702	746	700	716
rpmD	2857	922	-1695.33	-2.89071	1.79E-11	-3.27327	3.273273918	1.710734337	-1.71073	1.34E-09	2244	3721	1811	2592	937	864	889	896.6667
rpmE	4695	1472	-2728.33	-2.36599	3.19E-07	-2.71379	2.713793424	1.440310906	-1.44031	1.01E-05	3364	6542	4271	4725.667	2253	1892	1847	1997.333
rpmE2	43	29	-13	-1.15	0.072308	-1.29444	1.294443053	0.372331498	-0.37233	0.20304	117	107	75	99.66667	82	104	74	86.66667
rpmF	8702	2689	-5422.67	-2.12628	3.73E-06	-2.44473	2.444727811	1.289673849	-1.28967	8.1E-05	7401	13209	10102	10237.33	5225	4507	4712	4814.667
rpmG	2255	432	-1590.67	-1.85796	0.000105	-2.15258	2.15257972	1.106066668	-1.10607	0.001222	2237	4041	4056	3444.667	1956	1801	1805	1854
rpmH	569	66	-341.667	-1.42549	0.001428	-1.64581	1.645814765	0.718801971	-0.7188	0.010103	876	1212	1346	1144.667	822	777	810	803
rpmI	8489	2562	-5766	-1.65957	0.000547	-1.9172	1.917195845	0.938997719	-0.939	0.004715	11264	15737	16523	14508	9490	8034	8702	8742
rpmJ	7281	3162	-4512.33	-2.06156	1.3E-06	-2.34874	2.34874327	1.231889028	-1.23189	3.36E-05	7647	11266	7376	8763	4553	3985	4214	4250.667
rpmJ2	84	51	36	1.208494	0.608229	1.064994	1.064993698	0.090844894	0.090845	0.77892	212	151	155	172.6667	235	185	206	208.6667
rpoA	21593	7252	-12376.3	-1.74663	0.000215	-1.99795	1.997950692	0.998520979	-0.99852	0.002137	23865	36791	26202	28952.67	17918	15198	16613	16576.33
rpoB	17243	5296	-10582.7	-1.68866	0.00036	-1.94022	1.94021575	0.956217088	-0.95622	0.003304	21004	30918	25927	25949.67	16718	13675	15708	15367
rpoC	45475	19817	-31928	-2.01955	4.19E-06	-2.28782	2.287823685	1.193975872	-1.19398	8.89E-05	62691	75794	51246	63243.67	32199	31429	30319	31315.67
rpoD	1012	735	-173	-1.02585	0.323144	-1.17606	1.176055406	0.233956029	-0.23396	0.538237	6359	7146	7094	6866.333	7140	6134	6806	6693.333
rpoE	2746	494	-1564	-1.48496	0.003808	-1.72006	1.720062371	0.782460879	-0.78246	0.021714	3600	4915	5852	4789	3463	3106	3106	3225
rpoH	1069	353	-206.333	-1.02541	0.335305	-1.16916	1.169159009	0.225471155	-0.22547	0.549723	8230	8874	7877	8327	8653	7805	7904	8120.667
rpoN	526	86	17.33333	1.002932	0.422257	-1.13549	1.135490128	0.183315163	-0.18332	0.634824	6179	5868	5690	5912.333	6216	5782	5791	5929.667

Feature ID	Experiment - Range (original values)	Experiment - IQR (original values)	Experiment - Difference (original values)	Experiment - Fold Change (original values)	EDGE test: yccT NT vs WT NT , tagwise dispersions - P-value	EDGE test: yccT NT vs WT NT , tagwise dispersions - Fold change	yccT NT vs WT NT ABS|FC|	yccT NT vs WT NT Log2FC	yccT NT vs WT NT Log2FC +-	EDGE test: yccT NT vs WT NT , tagwise dispersions - FDR p-value correction	WT NT - WT.1.S22 - Expression values	WT NT - WT.2.S23 - Expression values	WT NT - WT.3.S24 - Expression values	WT NT - Means	yccT NT - yccT.1.S28 - Expression values	yccT NT - yccT.2.S29 - Expression values	yccT NT - yccT.3.S30 - Expression values	yccT NT - Means
rpoS	22800	14609	13497.67	1.738263	0.025029	1.557409	1.557409388	0.639148228	0.639148	0.09273	27158	15453	12238	18283	35038	30062	30242	31780.67
rpoZ	468	90	-216.667	-1.11657	0.087371	-1.27846	1.278461137	0.354408306	-0.35441	0.229842	1906	2216	2104	2075.333	1959	1748	1869	1858.667
rpsA	7391	3056	-2054.33	-1.11587	0.182841	-1.28719	1.287192918	0.364228294	-0.36423	0.371904	15185	22576	21593	19784.67	18721	15707	18763	17730.33
rpsB	22870	3939	-11969	-2.46529	5.94E-06	-2.82907	2.829067224	1.500326458	-1.50033	0.000119	12085	30629	17698	20137.33	8600	7759	8146	8168.333
rpsC	25178	11811	-16775	-6.02797	0	-6.87	6.870000038	2.780310107	-2.78031	0	15106	28468	16760	20111.33	3290	3295	3424	3336.333
rpsD	8122	3183	-4573	-1.48027	0.002557	-1.69052	1.690515264	0.757463042	-0.75746	0.015869	12706	16817	12761	14094.67	10347	8695	9523	9521.667
rpsE	9043	3166	-5571.33	-2.36141	5.93E-08	-2.67962	2.679623721	1.422030428	-1.42203	2.29E-06	8886	12798	7307	9663.667	4381	3755	4141	4092.333
rpsF	4981	177	-2564.67	-1.60487	0.00196	-1.78794	1.787943119	0.83830084	-0.8383	0.012906	9042	7311	4061	6804.667	4251	4323	4146	4240
rpsG	3397	225	-1796	-1.47677	0.004669	-1.70472	1.704721142	0.769535763	-0.76954	0.025444	3970	6763	5956	5563	4080	3366	3855	3767
rpsH	3186	920	-1901.33	-1.42701	0.003633	-1.63731	1.637308744	0.711326394	-0.71133	0.021016	5463	7211	6388	6354	4790	4025	4543	4452.667
rpsI	3225	976	-1970	-1.477	0.00212	-1.69882	1.698818249	0.764531511	-0.76453	0.013652	5120	6874	6306	6100	4597	3649	4144	4130
rpsJ	1413	354	-677.667	-1.30752	0.010134	-1.49348	1.493480255	0.578678165	-0.57868	0.046726	2674	3348	2622	2881.333	2408	1935	2268	2203.667
rpsK	3917	733	-2070	-1.30312	0.017133	-1.48112	1.481123369	0.566691813	-0.56669	0.06952	8772	10322	7603	8899	7212	6405	6870	6829
rpsL	2291	227	-1054.33	-1.37601	0.011926	-1.58397	1.583972228	0.663547041	-0.66355	0.052899	2803	4857	3915	3858.333	3030	2566	2816	2804
rpsM	5902	1706	-3437.33	-1.33797	0.012501	-1.52187	1.521871708	0.605846746	-0.60585	0.054788	13345	15522	11957	13608	10641	9620	10251	10170.67
rpsN	3650	656	-1858.67	-1.32009	0.014801	-1.51593	1.515929151	0.600202329	-0.6002	0.062518	6622	8690	7684	7665.333	6414	5040	5966	5806.667
rpsO	1329	175	-592.333	-1.19575	0.052972	-1.37005	1.370051167	0.454229774	-0.45423	0.162781	3128	4148	3579	3618.333	3217	2819	3042	3026
rpsP	3082	1021	-1716.33	-2.21324	3.96E-07	-2.52175	2.521745289	1.334422562	-1.33442	1.22E-05	2470	4404	2519	3131	1473	1322	1449	1414.667
rpsQ	17314	7384	-11497.7	-7.25213	0	-8.28595	8.28595471	3.050667935	-3.05067	0	9219	19110	11681	13336.67	1835	1886	1796	1839
rpsR	2943	54	-1586.67	-1.73321	0.000379	-1.93103	1.931030574	0.949371006	-0.94937	0.003432	4943	4114	2195	3750.667	2243	2249	2000	2164
rpsS	5652	2559	-3885	-4.53718	0	-5.2011	5.201101822	2.378817282	-2.37882	0	3672	6670	4608	4983.333	1164	1018	1113	1098.333
rpsT	780	434	-85	-1.02163	0.331527	-1.17167	1.171670511	0.228566922	-0.22857	0.545487	3570	4350	4124	4014.667	4231	3690	3868	3929.667
rpsU	378	203	137	1.058765	0.610628	-1.07886	1.078855914	0.1095022	-0.1095	0.781195	2259	2473	2262	2331.333	2637	2303	2465	2468.333
rpsV	965	725	473.3333	1.298257	0.333531	1.158412	1.158412243	0.212148756	0.212149	0.547444	2197	1332	1232	1587	2055	2069	2057	2060.333
rrmA	99	44	60	1.326087	0.203109	1.16747	1.167470094	0.223385594	0.223386	0.40088	215	171	166	184	260	207	265	244
rsd	87	42	-52	-1.08342	0.096697	-1.23328	1.233276058	0.30249577	-0.3025	0.245539	673	685	668	675.3333	598	646	626	623.3333
rseA	5906	1209	-3684.67	-1.72357	0.000474	-1.99664	1.996644004	0.997577127	-0.99758	0.004196	6296	9055	10980	8777	5074	5087	5116	5092.333
rseB	1166	215	-621.667	-1.22974	0.034847	-1.41406	1.414062278	0.49984566	-0.49985	0.119883	2967	3313	3703	3327.667	2829	2537	2752	2706
rseC	160	7	-86.6667	-1.16982	0.02912	-1.34096	1.340963515	0.423269984	-0.42327	0.10499	517	648	626	597	519	488	524	510.3333
rsmC	122	29	47	1.176692	0.755757	1.038522	1.038522072	0.054531878	0.054532	0.880907	302	273	223	266	319	275	345	313
rspA	17	7	12	1.507042	0.120409	1.324598	1.324598432	0.405555055	0.405555	0.283195	21	26	24	23.66667	31	38	38	35.66667
rspB	11	5	4	1.266667	0.661011	1.115107	1.115107369	0.157182628	0.157183	0.817117	14	18	13	15	20	24	13	19
rstA	425	341	325	1.744275	0.000731	1.546736	1.546736314	0.629227269	0.629227	0.005982	554	396	360	436.6667	785	737	763	761.6667
rstB	101	77	78.33333	1.633423	0.002188	1.438456	1.438455849	0.524520941	0.524521	0.013966	144	111	116	123.6667	212	193	201	202
rsuA	279	179	198.6667	1.538879	0.011251	1.361542	1.361542488	0.445242003	0.445242	0.050466	425	374	307	368.6667	563	553	586	567.3333
rtcA	170	57	31.66667	1.06363	0.620991	-1.06316	1.063162123	0.088361612	-0.08836	0.787112	565	533	395	497.6667	557	487	544	529.3333

Feature ID	Experiment - Range (original values)	Experiment - IQR (original values)	Experiment - Difference (original values)	Experiment - Fold Change (original values)	EDGE test: yccT NT vs WT NT , tagwise dispersions - P-value	EDGE test: yccT NT vs WT NT , tagwise dispersions - Fold change	yccT NT vs WT NT ABS|FC|	yccT NT vs WT NT Log2FC	yccT NT vs WT NT Log2FC +-	EDGE test: yccT NT vs WT NT , tagwise dispersions - FDR p-value correction	WT NT - WT.1.S22 - Expression values	WT NT - WT.2.S23 - Expression values	WT NT - WT.3.S24 - Expression values	WT NT - Means	yccT NT - yccT.1.S28 - Expression values	yccT NT - yccT.2.S29 - Expression values	yccT NT - yccT.3.S30 - Expression values	yccT NT - Means
rtcB	181	7	70.66667	1.177554	0.777107	1.036163	1.036163012	0.051250989	0.051251	0.892069	421	425	348	398	529	449	428	468.6667
rtcR	366	118	-185.333	-1.20801	0.013344	-1.37641	1.376414426	0.460914918	-0.46091	0.057629	1037	1209	983	1076.333	965	865	843	891
rthB	74	16	26.66667	1.107672	0.844107	-1.02502	1.025018763	0.035650319	-0.03565	0.94094	250	271	222	247.6667	261	266	296	274.3333
rtn	918	397	492.6667	1.329396	0.226639	1.179366	1.17936569	0.238011129	0.238011	0.431744	1801	1520	1166	1495.667	2084	1917	1964	1988.333
ruvA	150	111	93.66667	1.25	0.411206	1.105873	1.105872827	0.145185489	0.145185	0.625275	447	354	323	374.6667	473	465	467	468.3333
ruvB	279	210	198	1.341772	0.164908	1.184297	1.184297273	0.24403126	0.244031	0.348787	663	556	519	579.3333	798	768	766	777.3333
ruvC	344	122	-148	-1.17323	0.042108	-1.35333	1.353330596	0.436514309	-0.43651	0.138271	795	1081	1131	1002.333	917	787	859	854.3333
safA	15	3	5.666667	1.85	0.124098	1.613279	1.613278675	0.689995668	0.689996	0.288461	10	1	9	6.666667	14	7	16	12.33333
safB	44	21	26.66667	1.544218	0.032803	1.363455	1.363454577	0.447266638	0.447267	0.11445	55	49	43	49	70	87	70	75.66667
safC	114	37	58	1.172107	0.769739	1.035446	1.03544568	0.050251872	0.050252	0.887872	374	351	286	337	397	388	400	395
safD	24	4	11	1.25	0.598076	1.095476	1.095476383	0.131558382	0.131558	0.772841	42	43	47	44	55	44	66	55
sanA	585	226	314	1.351624	0.16698	1.200174	1.200173676	0.263243192	0.263243	0.351653	1086	920	673	893	1258	1146	1217	1207
sapA	610	226	391.6667	1.297996	0.296546	1.142512	1.142511677	0.19220891	0.192209	0.512736	1374	1405	1164	1314.333	1774	1600	1744	1706
sapB	83	52	62	1.226277	0.537966	1.076633	1.07663306	0.106526632	0.106527	0.727856	272	286	264	274	337	324	347	336
sapC	105	26	-33	-1.13904	0.040503	-1.30344	1.303436219	0.382319989	-0.38232	0.134406	235	318	258	270.3333	267	213	232	237.3333
sapD	286	61	-134.333	-1.37912	0.001147	-1.58353	1.583526505	0.663141015	-0.66314	0.008501	425	568	473	488.6667	417	282	364	354.3333
sapF	115	35	-23	-1.03896	0.170946	-1.18431	1.184311489	0.244048578	-0.24405	0.356643	602	671	567	613.3333	643	556	572	590.3333
sbcB	503	137	251	1.269699	0.361745	1.121472	1.121471565	0.165393042	0.165393	0.578453	1056	966	770	930.6667	1273	1103	1169	1181.667
sbcC	312	90	162.3333	1.306482	0.300409	1.14605	1.146049604	0.196669489	0.196669	0.515535	611	437	541	529.6667	749	631	696	692
sbcD	185	41	98	1.224599	0.528329	1.078122	1.07812192	0.108520336	0.10852	0.722435	469	455	385	436.3333	570	496	537	534.3333
sbmA	225	123	126.6667	1.20731	0.616723	1.062987	1.062986738	0.088123598	0.088124	0.78418	682	588	563	611	788	711	714	737.6667
sbmC	633	299	146.3333	1.087363	0.78633	-1.0383	1.038300588	0.054224164	-0.05422	0.898692	2068	1522	1435	1675	1914	1821	1729	1821.333
sbp	45	22	14.66667	1.119565	0.93001	-1.0128	1.012804361	0.018355522	-0.01836	0.987982	143	122	103	122.6667	148	121	143	137.3333
scsA	186	90	90.66667	1.534381	0.051385	1.355345	1.355345379	0.438660536	0.438661	0.159076	240	119	150	169.6667	305	211	265	260.3333
scsB	89	20	46.66667	1.350877	0.179883	1.184827	1.184827157	0.244676614	0.244677	0.368158	151	117	131	133	206	151	182	179.6667
scsC	21	6	4	1.103448	0.918617	-1.02667	1.026674222	0.037978467	-0.03798	0.982488	52	33	31	38.66667	51	39	38	42.66667
scsD	29	9	2	1.040816	0.58773	-1.09325	1.093247234	0.128619698	-0.12862	0.76537	61	41	45	49	66	37	50	51
sdaA	931	791	835	1.389398	0.185978	1.218508	1.218508074	0.28511581	0.285116	0.37543	2181	2108	2144	2144.333	3039	2964	2935	2979.333
sdaB	2997	677	438.6667	1.093679	0.757665	-1.06496	1.06495838	0.09079705	-0.0908	0.880907	3137	4777	6134	4682.667	5328	4651	5385	5121.333
sdaC	4500	956	-1128.67	-1.16315	0.135152	-1.34917	1.349166451	0.432068349	-0.43207	0.304144	5585	8470	10085	8046.667	6770	6514	7470	6918
sdhA	1362	983	500.6667	1.159346	0.920998	1.017165	1.017165041	0.024553784	0.024554	0.983597	2736	4026	2664	3142	3719	3211	3998	3642.667
sdhB	1263	619	206	1.079804	0.754983	-1.054	1.054004621	0.075881192	-0.07588	0.880582	2325	3341	2078	2581.333	2944	2408	3010	2787.333
sdhC	356	67	-57	-1.03871	0.219724	-1.18573	1.185725139	0.245769619	-0.24577	0.423487	1402	1717	1469	1529.333	1426	1361	1630	1472.333
sdhD	276	175	133.6667	1.173069	0.82947	1.029801	1.029801086	0.042365697	0.042366	0.930686	691	924	702	772.3333	877	874	967	906
sdiA	3351	1117	1421.667	1.39316	0.236259	1.24975	1.249749555	0.321639013	0.321639	0.444591	4985	3748	2115	3616	5466	4865	4782	5037.667
secA	3226	591	-1988.67	-1.33869	0.013136	-1.54147	1.541470703	0.62430747	-0.62431	0.056897	6555	8358	8668	7860.333	6209	5442	5964	5871.667

Feature ID	Experiment - Range (original values)	Experiment - IQR (original values)	Experiment - Difference (original values)	Experiment - Fold Change (original values)	EDGE test: yccT NT vs WT NT , tagwise dispersions - P-value	EDGE test: yccT NT vs WT NT , tagwise dispersions - Fold change	yccT NT vs WT NT ABS|FC|	yccT NT vs WT NT Log2FC	yccT NT vs WT NT Log2FC +-	EDGE test: yccT NT vs WT NT , tagwise dispersions - FDR p-value correction	WT NT - WT.1.S22 - Expression values	WT NT - WT.2.S23 - Expression values	WT NT - WT.3.S24 - Expression values	WT NT - Means	yccT NT - yccT.1.S28 - Expression values	yccT NT - yccT.2.S29 - Expression values	yccT NT - yccT.3.S30 - Expression values	yccT NT - Means
secB	3055	1255	-2165.33	-1.33236	0.012521	-1.52677	1.526765283	0.610478287	-0.61048	0.054822	7874	8883	9284	8680.333	6697	6619	6229	6515
secD	623	131	238	1.05586	0.613406	-1.08264	1.082642798	0.114557325	-0.11456	0.782388	4294	4166	4322	4260.667	4789	4191	4516	4498.667
secE	433	293	307	1.18873	0.73732	1.045952	1.045952437	0.064817249	0.064817	0.867977	1756	1587	1537	1626.667	1951	1880	1970	1933.667
secF	361	23	15.66667	1.007409	0.387169	-1.13043	1.130429807	0.176871412	-0.17687	0.604066	2315	1954	2075	2114.667	2270	2052	2069	2130.333
secG	1474	462	690.3333	1.155154	0.890941	1.021741	1.021741129	0.031029717	0.03103	0.968063	4987	4633	3728	4449.333	5202	5095	5122	5139.667
secY	42327	13483	-25086.7	-2.57188	8.2E-08	-2.91621	2.916213221	1.544096207	-1.5441	3E-06	36431	57087	29621	41046.33	16981	14760	16138	15959.67
selA	2016	685	-1201	-1.32095	0.01036	-1.50272	1.502720169	0.587576381	-0.58758	0.047376	4808	5633	4388	4943	3906	3617	3703	3742
selB	3176	1469	-1995.33	-1.45514	0.002044	-1.6627	1.66269568	0.733524139	-0.73352	0.013319	5797	7312	6029	6379.333	4688	4136	4328	4384
selD	335	191	-105	-1.04559	0.232833	-1.19182	1.191821836	0.253168585	-0.25317	0.439951	2415	2541	2269	2408.333	2480	2206	2224	2303.333
SEN0013	22	11	8	1.147239	0.969597	1.013345	1.013345211	0.019125733	0.019126	1	61	50	52	54.33333	50	72	65	62.33333
SEN0014	4	1	1.666667	2.25	0.396003	1.901906	1.901905743	0.927445749	0.927446	0.61207	1	2	1	1.333333	2	2	5	3
SEN0015	5	1	-0.33333	-1.04545	0.7769	-1.1823	1.182300775	0.241597101	-0.2416	0.892069	10	7	6	7.666667	5	7	10	7.333333
SEN0016	9	3	5	2.363636	0.076151	2.048004	2.048004197	1.034218672	1.034219	0.20995	4	5	2	3.666667	11	8	7	8.666667
SEN0017	130	87	77.66667	1.336705	0.155177	1.182217	1.182216699	0.241494504	0.241495	0.33296	285	211	196	230.6667	326	298	301	308.3333
SEN0018	116	61	75	1.339367	0.141207	1.182447	1.182447145	0.241775697	0.241776	0.313373	253	220	190	221	301	281	306	296
SEN0019	51	40	40	1.681818	0.001899	1.483572	1.483571579	0.569074536	0.569075	0.012558	70	57	49	58.66667	100	97	99	98.66667
SEN0028	8	6	-1.66667	-1.05682	0.30767	-1.20349	1.203489303	0.267223319	-0.26722	0.523149	27	33	33	31	34	28	26	29.33333
SEN0029	11	5	6.666667	2.176471	0.036823	1.902377	1.902377391	0.927803474	0.927803	0.125222	8	7	2	5.666667	12	13	12	12.33333
SEN0030	10	4	6	1.45	0.309069	1.275244	1.275244357	0.350773717	0.350774	0.524245	14	13	13	13.33333	17	23	18	19.33333
SEN0031	8	4	0	-1	0.871567	-1.13612	1.136115218	0.184109152	-0.18411	0.957816	7	3	7	5.666667	10	2	5	5.666667
SEN0032	86	52	55	1.959302	0.000349	1.744166	1.744166046	0.802537393	0.802537	0.003212	83	54	35	57.33333	110	106	121	112.3333
SEN0033	158	81	102	1.638831	0.00269	1.448541	1.448540976	0.534600496	0.5346	0.016532	195	151	133	159.6667	262	232	291	261.6667
SEN0034	6	1	1.333333	1.285714	0.866246	1.130821	1.130821347	0.177371023	0.177371	0.954335	5	5	4	4.666667	4	10	4	6
SEN0035	82	54	51.66667	1.375303	0.114234	1.212033	1.21203334	0.277429385	0.277429	0.273902	165	119	129	137.6667	201	183	184	189.3333
SEN0036	121	64	76.66667	1.415913	0.059674	1.252438	1.252438106	0.324739309	0.324739	0.177341	214	182	157	184.3333	246	259	278	261
SEN0037	48	34	39.33333	2.787879	2.59E-08	2.44426	2.444260063	1.289397793	1.289398	1.15E-06	24	20	22	22	68	56	60	61.33333
SEN0038	98	73	76	1.616216	0.002328	1.426685	1.426685348	0.512667187	0.512667	0.014579	145	119	106	123.3333	204	192	202	199.3333
SEN0041	207	144	130.6667	1.352518	0.147363	1.197971	1.197970529	0.260592418	0.260592	0.321896	453	350	309	370.6667	516	494	494	501.3333
SEN0042	52	17	28.66667	1.632353	0.011802	1.436571	1.436570867	0.522629164	0.522629	0.052451	52	48	36	45.33333	88	65	69	74
SEN0053	231	78	144	1.339623	0.174669	1.177679	1.177678815	0.235946131	0.235946	0.362341	469	417	386	424	617	495	592	568
SEN0054	394	217	264.3333	1.763234	0.000523	1.564934	1.56493417	0.64610197	0.646102	0.004544	413	373	253	346.3333	647	590	595	610.6667
SEN0055	13	2	5.666667	2.7	0.018108	2.343618	2.343617581	1.228737177	1.228737	0.072547	4	6	0	3.333333	5	13	9	9
SEN0056	8	6	6.333333	4.166667	0.002007	3.525113	3.525113421	1.817669677	1.81767	0.013125	4	1	1	2	9	9	7	8.333333
SEN0057	18	7	11	2.434783	0.005106	2.12004	2.120039777	1.084091334	1.084091	0.027159	7	5	11	7.666667	23	19	14	18.66667
SEN0058	193	130	130	2.308725	0.000292	2.051963	2.051962769	1.037004555	1.037005	0.002755	159	48	91	99.33333	241	221	226	229.3333
SEN0081	31	15	22	2.047619	0.001015	1.8068	1.806800254	0.853437022	0.853437	0.0077	27	20	16	21	35	47	47	43

Feature ID	Experiment - Range (original values)	Experiment - IQR (original values)	Experiment - Difference (original values)	Experiment - Fold Change (original values)	EDGE test: yccT NT vs WT NT , tagwise dispersions - P-value	EDGE test: yccT NT vs WT NT , tagwise dispersions - Fold change	yccT NT vs WT NT ABS|FC|	yccT NT vs WT NT Log2FC	yccT NT vs WT NT Log2FC +-	EDGE test: yccT NT vs WT NT , tagwise dispersions - FDR p-value correction	WT NT - WT.1.S22 - Expression values	WT NT - WT.2.S23 - Expression values	WT NT - WT.3.S24 - Expression values	WT NT - Means	yccT NT - yccT.1.S28 - Expression values	yccT NT - yccT.2.S29 - Expression values	yccT NT - yccT.3.S30 - Expression values	yccT NT - Means
SEN0083	121	103	98.33333	2.282609	5.32E-08	2.017229	2.017229445	1.012375189	1.012375	2.07E-06	101	65	64	76.66667	185	168	172	175
SEN0084	108	67	64	1.876712	0.001709	1.668599	1.668599307	0.738637552	0.738638	0.011666	112	56	51	73	159	123	129	137
SEN0085	100	46	63.33333	1.568862	0.0106	1.380562	1.380561579	0.46525524	0.465255	0.048122	133	93	108	111.3333	177	154	193	174.6667
SEN0100	34	10	20	1.689655	0.01821	1.484283	1.484283048	0.569766235	0.569766	0.07277	34	23	30	29	57	50	40	49
SEN0101	12	4	6	1.514286	0.294205	1.32841	1.328410116	0.409700615	0.409701	0.510818	11	9	15	11.66667	13	19	21	17.66667
SEN0110A	375	254	242.3333	1.401657	0.09069	1.2431	1.243099874	0.313942211	0.313942	0.235825	741	552	517	603.3333	839	892	806	845.6667
SEN0110B	84	57	62.66667	1.839286	0.000301	1.630331	1.630330647	0.705164587	0.705165	0.002817	96	67	61	74.66667	124	145	143	137.3333
SEN0139	65	17	44	1.199396	0.6755	1.05189	1.051890167	0.072984074	0.072984	0.825117	227	220	215	220.6667	280	237	277	264.6667
SEN0141	403	72	181.6667	1.230444	0.495546	1.090214	1.090213514	0.124610709	0.124611	0.699065	907	850	608	788.3333	1011	922	977	970
SEN0152	218	83	81.33333	1.209622	0.589802	1.073024	1.073024362	0.101682831	0.101683	0.765947	480	397	287	388	505	418	485	469.3333
SEN0153	76	29	36.33333	1.52657	0.048035	1.356183	1.35618271	0.439551557	0.439552	0.151921	96	67	44	69	109	87	120	105.3333
SEN0159	18	8	4	1.153846	0.94761	1.01999	1.019989996	0.028555003	0.028555	0.999617	37	22	19	26	32	30	28	30
SEN0160	61	27	36.66667	1.384615	0.095699	1.223577	1.223577483	0.291105463	0.291105	0.243815	108	102	76	95.33333	129	130	137	132
SEN0163	93	40	-2.33333	-1.00778	0.263525	-1.14581	1.14581328	0.196371964	-0.19637	0.475513	347	280	280	302.3333	320	254	326	300
SEN0164	35	13	15.33333	1.442308	0.159472	1.280959	1.280958632	0.357223886	0.357224	0.339884	48	34	22	34.66667	46	57	47	50
SEN0167	2999	24	-1123.33	-4.89595	5.62E-05	-5.46158	5.461583702	2.449319352	-2.44932	0.00075	808	3213	214	1411.667	287	277	301	288.3333
SEN0168	2520	24	-942	-2.86658	0.000237	-3.21094	3.210944676	1.682997808	-1.683	0.002317	948	2956	436	1446.667	520	498	496	504.6667
SEN0169	1158	129	85.33333	1.037786	0.633424	-1.08125	1.081248947	0.112698728	-0.1127	0.796785	2643	2645	1487	2258.333	2421	2318	2292	2343.667
SEN0206	77	20	36	1.183362	0.712317	1.044733	1.044733073	0.063134384	0.063134	0.849483	222	202	165	196.3333	242	222	233	232.3333
SEN0216	2113	1653	934.3333	1.306708	0.399177	1.163767	1.163766877	0.218802089	0.218802	0.614907	4418	2305	2416	3046.333	4286	3587	4069	3980.667
SEN0218	636	355	370.3333	1.33872	0.184218	1.184804	1.184804497	0.244649022	0.244649	0.373404	1330	1034	916	1093.333	1552	1389	1450	1463.667
SEN0222A	1626	199	-799	-1.92656	3.08E-05	-2.20628	2.206280612	1.141616296	-1.14162	0.000472	1080	2451	1453	1661.333	881	825	881	862.3333
SEN0240	217	117	104.6667	1.286758	0.305896	1.14085	1.140849652	0.190108677	0.190109	0.521303	459	342	294	365	511	466	432	469.6667
SEN0249	1045	661	690	1.460205	0.063121	1.295504	1.295503953	0.373513417	0.373513	0.184736	1837	1459	1202	1499.333	2247	2120	2201	2189.333
SEN0259	19	12	7.333333	1.101852	0.860389	-1.02955	1.02954928	0.042012886	-0.04201	0.950937	84	67	65	72	82	77	79	79.33333
SEN0267	37	12	-5.33333	-1.02671	0.184025	-1.16989	1.16989487	0.226378891	-0.22638	0.373404	201	221	193	205	210	184	205	199.6667
SEN0268	84	65	41.33333	1.234405	0.495837	1.090718	1.090717625	0.125277652	0.125278	0.699254	225	146	158	176.3333	223	200	230	217.6667
SEN0269	13	2	5.333333	1.64	0.247825	1.436932	1.436932253	0.522992045	0.522992	0.459078	9	10	6	8.333333	19	11	11	13.66667
SEN0270	16	1	5	1.3125	0.55665	1.157667	1.157667321	0.211220725	0.211221	0.741429	18	20	10	16	18	19	26	21
SEN0271	19	5	11	2.571429	0.002656	2.251131	2.251130891	1.170649944	1.17065	0.016402	10	10	1	7	19	15	20	18
SEN0272	222	137	150.3333	1.334322	0.168996	1.177747	1.177747298	0.236030022	0.23603	0.353886	518	435	396	449.6667	618	572	610	600
SEN0273	90	16	46.33333	1.532567	0.040525	1.367094	1.36709361	0.451112033	0.451112	0.134406	107	100	54	87	116	144	140	133.3333
SEN0274	15	13	9	1.818182	0.068613	1.60278	1.602779986	0.6805764	0.680576	0.196057	20	6	7	11	21	18	21	20
SEN0276	7	3	3.333333	2.111111	0.201435	1.821418	1.821418478	0.865062425	0.865062	0.398911	2	2	5	3	9	4	6	6.333333
SEN0277	18	2	7	2.4	0.03031	2.080339	2.080338884	1.05681856	1.056819	0.108477	5	7	3	5	21	7	8	12
SEN0277A	4	3	3.333333	3.5	0.053845	2.9116	2.911600337	1.541812336	1.541812	0.164497	1	1	2	1.333333	5	4	5	4.666667

Feature ID	Experiment - Range (original values)	Experiment - IQR (original values)	Experiment - Difference (original values)	Experiment - Fold Change (original values)	EDGE test: yccT NT vs WT NT , tagwise dispersions - P-value	EDGE test: yccT NT vs WT NT , tagwise dispersions - Fold change	yccT NT vs WT NT ABS|FC|	yccT NT vs WT NT Log2FC	yccT NT vs WT NT Log2FC +-	EDGE test: yccT NT vs WT NT , tagwise dispersions - FDR p-value correction	WT NT - WT.1.S22 - Expression values	WT NT - WT.2.S23 - Expression values	WT NT - WT.3.S24 - Expression values	WT NT - Means	yccT NT - yccT.1.S28 - Expression values	yccT NT - yccT.2.S29 - Expression values	yccT NT - yccT.3.S30 - Expression values	yccT NT - Means
SEN0278	10	0	4.333333	2.083333	0.119958	1.808163	1.808163144	0.854524852	0.854525	0.282732	4	4	4	4	13	9	3	8.333333
SEN0280	2	0	0.333333	2	1	1.563037	1.56303737	0.644352272	0.644352	1	1	0	0	0.333333	2	0	0	0.666667
SEN0286	6	2	-0.66667	-1.25	0.495445	-1.3998	1.399801572	0.485222333	-0.48522	0.699065	6	1	3	3.333333	6	2	0	2.666667
SEN0288	34	11	21.66667	1.541667	0.047396	1.353305	1.353304613	0.43648661	0.436487	0.151081	42	36	42	40	70	62	53	61.66667
SEN0289	116	36	45.66667	1.15729	0.90008	1.017928	1.017928365	0.025636037	0.025636	0.971561	343	227	301	290.3333	339	337	332	336
SEN0290	179	144	119	1.277821	0.325457	1.130677	1.130676845	0.177186657	0.177187	0.539305	521	385	379	428.3333	555	558	529	547.3333
SEN0297	227	100	159.6667	1.983573	4.5E-06	1.74832	1.748319619	0.805968955	0.805969	9.32E-05	179	179	129	162.3333	356	279	331	322
SEN0306	225	110	93.66667	1.212718	0.565169	1.077276	1.077275984	0.107387897	0.107388	0.746428	551	441	329	440.3333	551	497	554	534
SEN0307	51	26	31.33333	1.301282	0.276881	1.145234	1.14523388	0.195642257	0.195642	0.489961	122	94	96	104	145	117	144	135.3333
SEN0308	31	8	14.33333	1.167969	0.886725	1.024926	1.024926403	0.035520317	0.03552	0.964901	82	91	83	85.33333	100	86	113	99.66667
SEN0310	1376	342	-448	-1.1641	0.086042	-1.31098	1.310984161	0.390650255	-0.39065	0.228294	3827	3179	2528	3178	2870	2869	2451	2730
SEN0311	147	24	51	1.243243	0.484163	1.105032	1.105032349	0.144088604	0.144089	0.688948	241	249	139	209.6667	286	271	225	260.6667
SEN0312	89	4	42.33333	1.389571	0.122848	1.231716	1.231716031	0.300669684	0.30067	0.286982	125	125	76	108.6667	165	159	129	151
SEN0313	43	16	9.666667	1.119835	0.953067	-1.01223	1.012234207	0.017543135	-0.01754	1	101	77	64	80.66667	107	74	90	90.33333
SEN0314	43	13	22.66667	1.441558	0.143411	1.262325	1.262325175	0.336083596	0.336084	0.315855	45	53	56	51.33333	68	66	88	74
SEN0315	173	107	120.3333	1.406074	0.07037	1.241149	1.241149282	0.311676649	0.311677	0.1993	352	277	260	296.3333	433	384	433	416.6667
SEN0316	187	113	120.6667	1.615646	0.003228	1.424401	1.424401035	0.510355389	0.510355	0.019226	236	179	173	196	360	298	292	316.6667
SEN0317	24	12	12.33333	1.72549	0.042321	1.521504	1.52150361	0.605497757	0.605498	0.138701	26	14	11	17	35	28	25	29.33333
SEN0318	22	6	5	1.119048	1	-1.01218	1.01218188	0.017468552	-0.01747	1	43	46	37	42	44	59	38	47
SEN0324	78	34	48.66667	1.660633	0.002236	1.464893	1.464893457	0.55079574	0.550796	0.014132	87	77	57	73.66667	135	111	121	122.3333
SEN0325	43	25	29	1.966667	0.000339	1.729613	1.729612824	0.790449125	0.790449	0.003128	36	29	25	30	68	55	54	59
SEN0326	162	103	107	1.736239	0.000584	1.539918	1.539917751	0.622853297	0.622853	0.004946	189	137	110	145.3333	272	245	240	252.3333
SEN0327	121	66	95	1.505319	0.017637	1.321204	1.321203914	0.401853149	0.401853	0.070967	198	185	181	188	296	251	302	283
SEN0328	37	27	28.66667	1.597222	0.019535	1.403644	1.403643707	0.489176777	0.489177	0.076742	44	53	47	48	75	81	74	76.66667
SEN0329	9	4	3.666667	1.6875	0.310525	1.478223	1.478222952	0.563863879	0.563864	0.525768	4	10	2	5.333333	8	11	8	9
SEN0330	32	11	20.33333	2.794118	6.96E-05	2.4645	2.464500195	1.301295095	1.301295	0.000889	15	15	4	11.33333	26	36	33	31.66667
SEN0331	4	0	-0.33333	-1.06667	0.72928	-1.20689	1.206890061	0.271294263	-0.27129	0.861947	6	5	5	5.333333	7	3	5	5
SEN0332	17	5	5	1.112782	0.961328	-1.0209	1.020904596	0.029848051	-0.02985	1	52	38	43	44.33333	55	48	45	49.33333
SEN0333	44	13	18.66667	1.352201	0.218884	1.194433	1.194433497	0.25632653	0.256327	0.42242	65	52	42	53	67	62	86	71.66667
SEN0334	37	5	14	1.164063	0.894767	1.0225	1.02250026	0.03210121	0.032101	0.969131	89	73	94	85.33333	95	110	93	99.33333
SEN0335	7	2	4	1.461538	0.410916	1.282512	1.282511885	0.358972195	0.358972	0.62527	9	7	10	8.666667	11	13	14	12.66667
SEN0336	48	33	22	1.153488	0.898187	1.017247	1.017246836	0.024669793	0.02467	0.971085	169	131	130	143.3333	178	164	154	165.3333
SEN0337	90	47	45	1.372928	0.129091	1.220685	1.220684543	0.287690418	0.28769	0.296682	161	113	88	120.6667	159	160	178	165.6667
SEN0338	49	24	27.66667	1.338776	0.179438	1.181856	1.181855885	0.241054125	0.241054	0.367586	99	77	69	81.66667	109	101	118	109.3333
SEN0339	143	98	112.6667	1.806683	6.17E-05	1.59052	1.590519811	0.669498341	0.669498	0.000806	154	136	129	139.6667	251	234	272	252.3333
SEN0342	13	7	7.666667	2	0.043996	1.754344	1.754344121	0.810931766	0.810932	0.142686	11	6	6	7.666667	13	19	14	15.33333

Feature ID	Experiment - Range (original values)	Experiment - IQR (original values)	Experiment - Difference (original values)	Experiment - Fold Change (original values)	EDGE test: yccT NT vs WT NT , tagwise dispersions - P-value	EDGE test: yccT NT vs WT NT , tagwise dispersions - Fold change	yccT NT vs WT NT ABS|FC|	yccT NT vs WT NT Log2FC	yccT NT vs WT NT Log2FC +-	EDGE test: yccT NT vs WT NT , tagwise dispersions - FDR p-value correction	WT NT - WT.1.S22 - Expression values	WT NT - WT.2.S23 - Expression values	WT NT - WT.3.S24 - Expression values	WT NT - Means	yccT NT - yccT.1.S28 - Expression values	yccT NT - yccT.2.S29 - Expression values	yccT NT - yccT.3.S30 - Expression values	yccT NT - Means
SEN0343	54	21	31.33333	1.696296	0.008349	1.499461	1.499460738	0.584443747	0.584444	0.040301	61	42	32	45	80	63	86	76.33333
SEN0344	65	33	20.33333	1.078811	0.668628	-1.05215	1.052151548	0.07334252	-0.07334	0.821302	295	237	242	258	275	258	302	278.3333
SEN0345	22	11	-2.66667	-1.03704	0.222319	-1.17873	1.178731056	0.237234585	-0.23723	0.426632	75	85	64	74.66667	79	63	74	72
SEN0346	131	96	70	1.326594	0.202297	1.175268	1.175268275	0.232990114	0.23299	0.399813	282	186	175	214.3333	306	261	286	284.3333
SEN0364	55	24	18.33333	1.212355	0.605298	1.078823	1.07882343	0.109458759	0.109459	0.777315	118	78	63	86.33333	102	114	98	104.6667
SEN0365	82	30	52.33333	1.726852	0.001157	1.524342	1.524342424	0.608187022	0.608187	0.00856	87	75	54	72	132	105	136	124.3333
SEN0385	5695	1114	567	1.036185	0.60382	-1.09087	1.0908732	0.125483416	-0.12548	0.77698	18969	14765	13274	15669.33	17377	15453	15879	16236.33
SEN0392	165	92	80.33333	1.307791	0.24077	1.16126	1.161260032	0.21569106	0.215691	0.451455	339	247	197	261	362	317	345	341.3333
SEN0393	177	142	128	1.408946	0.07007	1.242467	1.242466503	0.313206958	0.313207	0.199027	367	283	289	313	460	431	432	441
SEN0419	57	43	46	2.159664	3.66E-06	1.902718	1.902717605	0.928061457	0.928061	7.99E-05	46	39	34	39.66667	82	84	91	85.66667
SEN0440	59	19	-14.6667	-1.10577	0.078899	-1.24979	1.249785789	0.321680841	-0.32168	0.215936	167	176	117	153.3333	146	143	127	138.6667
SEN0460	372	293	182.6667	1.23795	0.477128	1.098233	1.098232938	0.135184086	0.135184	0.683086	982	678	643	767.6667	1015	971	865	950.3333
SEN0478	116	46	63.33333	1.844444	0.000916	1.620974	1.620974241	0.696861165	0.696861	0.007171	74	95	56	75	172	120	123	138.3333
SEN0490	175	37	-66	-1.2093	0.013157	-1.38291	1.382913372	0.467710786	-0.46771	0.056932	342	449	353	381.3333	356	274	316	315.3333
SEN0501	22	6	12	1.433735	0.181261	1.259908	1.259907724	0.333318074	0.333318	0.369879	32	26	25	27.66667	47	31	41	39.66667
SEN0519	233	55	110	1.237752	0.464345	1.092673	1.092673261	0.127862061	0.127862	0.67239	530	475	383	462.6667	616	513	589	572.6667
SEN0520	92	58	48	1.55814	0.044521	1.391974	1.391973574	0.477131823	0.477132	0.143895	127	64	67	86	121	156	125	134
SEN0532	3	2	0.333333	1.0625	1	-1.06622	1.066224905	0.092511786	-0.09251	1	7	5	4	5.333333	6	7	4	5.666667
SEN0534A	10	3	3	1.333333	0.643197	1.172296	1.172296181	0.229337113	0.229337	0.803497	14	4	9	9	14	10	12	12
SEN0535	10	1	2.666667	1.123077	0.94029	-1.0143	1.014298178	0.020481831	-0.02048	0.994611	21	20	24	21.66667	30	22	21	24.33333
SEN0537A	37	12	21	2.188679	0.000983	1.925149	1.925149493	0.944970479	0.94497	0.007535	24	16	13	17.66667	38	28	50	38.66667
SEN0538	16	6	7.666667	1.479167	0.255198	1.29929	1.299290384	0.377723901	0.377724	0.467339	21	12	15	16	28	17	26	23.66667
SEN0538A	54	19	28.33333	1.400943	0.1047	1.238522	1.23852246	0.308620031	0.30862	0.258888	84	71	57	70.66667	90	96	111	99
SEN0539	98	30	57.66667	1.295726	0.25743	1.140266	1.140265532	0.189369822	0.18937	0.469398	215	196	174	195	272	226	260	252.6667
SEN0540	55	26	32.33333	1.309904	0.22158	1.157459	1.157459056	0.21096116	0.210961	0.425584	121	108	84	104.3333	139	134	137	136.6667
SEN0541	2	0	-0.33333	-1.16667	0.794462	-1.30531	1.305305417	0.384387409	-0.38439	0.903705	2	2	3	2.333333	1	2	3	2
SEN0542	47	24	34.66667	1.571429	0.014333	1.379163	1.379163317	0.463793307	0.463793	0.061068	59	66	57	60.66667	104	83	99	95.33333
SEN0543	710	506	506	1.440128	0.064067	1.275586	1.275586161	0.351160351	0.35116	0.187011	1366	1122	961	1149.667	1668	1628	1671	1655.667
SEN0550	137	78	87	1.353659	0.136365	1.196529	1.196528986	0.258855346	0.258855	0.305632	300	229	209	246	346	307	346	333
SEN0551	33	23	14	1.291667	0.439273	1.138533	1.13853251	0.187175487	0.187175	0.650977	65	37	42	48	70	48	68	62
SEN0579	26	9	16.66667	4.333333	1.02E-05	3.75578	3.755780457	1.909112733	1.909113	0.000186	10	3	2	5	28	12	25	21.66667
SEN0580	70	17	25	1.07485	0.646093	-1.05526	1.055264172	0.077604205	-0.0776	0.805331	362	341	299	334	358	350	369	359
SEN0581	18	5	5.333333	1.181818	0.903772	1.035663	1.035662778	0.050554323	0.050554	0.973907	25	33	30	29.33333	43	28	33	34.66667
SEN0582	286	197	192	1.445131	0.043841	1.277011	1.277010718	0.352770633	0.352771	0.142418	515	404	375	431.3333	661	608	601	623.3333
SEN0618	25	6	14.33333	2.482759	0.003741	2.184906	2.18490595	1.12757118	1.127571	0.021417	14	8	7	9.666667	28	32	12	24
SEN0619	87	68	64	2.381295	1.27E-06	2.109456	2.109455838	1.076870885	1.076871	3.31E-05	69	36	34	46.33333	121	106	104	110.3333

Feature ID	Experiment - Range (original values)	Experiment - IQR (original values)	Experiment - Difference (original values)	Experiment - Fold Change (original values)	EDGE test: yccT NT vs WT NT , tagwise dispersions - P-value	EDGE test: yccT NT vs WT NT , tagwise dispersions - Fold change	yccT NT vs WT NT ABS|FC|	yccT NT vs WT NT Log2FC	yccT NT vs WT NT Log2FC +-	EDGE test: yccT NT vs WT NT , tagwise dispersions - FDR p-value correction	WT NT - WT.1.S22 - Expression values	WT NT - WT.2.S23 - Expression values	WT NT - WT.3.S24 - Expression values	WT NT - Means	yccT NT - yccT.1.S28 - Expression values	yccT NT - yccT.2.S29 - Expression values	yccT NT - yccT.3.S30 - Expression values	yccT NT - Means
SEN0620	242	140	144.3333	1.560881	0.012956	1.383415	1.383414904	0.468233904	0.468234	0.05645	340	227	205	257.3333	447	367	391	401.6667
SEN0621	894	562	562	1.45189	0.060919	1.286669	1.286669138	0.363641118	0.363641	0.179847	1560	1127	1044	1243.667	1938	1790	1689	1805.667
SEN0629	2143	159	-932.667	-2.14672	5.41E-05	-2.42196	2.421958409	1.27617409	-1.27617	0.000726	1353	2933	952	1746	857	790	793	813.3333
SEN0641	136	22	55	1.128505	0.993232	-1.002	1.002004458	0.002888927	-0.00289	1	473	457	354	428	480	479	490	483
SEN0655	38	12	23.33333	1.267176	0.43023	1.109724	1.109724188	0.150201153	0.150201	0.641005	82	89	91	87.33333	120	101	111	110.6667
SEN0656	184	86	117.3333	1.428745	0.048256	1.262108	1.262107621	0.335834935	0.335835	0.152295	305	286	230	273.6667	387	372	414	391
SEN0663	2286	419	-1303.67	-4.72831	3.41E-11	-5.57766	5.577657044	2.47965923	-2.47966	2.44E-09	767	1571	2622	1653.333	348	336	365	349.6667
SEN0681	1058	124	-453.333	-1.23801	0.027654	-1.39708	1.397081001	0.482415669	-0.48242	0.100769	2478	2827	1769	2358	1952	1934	1828	1904.667
SEN0706	198	140	124.3333	1.555887	0.019327	1.381472	1.381471714	0.466206023	0.466206	0.076258	309	177	185	223.6667	375	344	325	348
SEN0707	122	81	70	1.357143	0.170721	1.201858	1.201858111	0.265266584	0.265267	0.356487	256	157	175	196	279	277	242	266
SEN0708	127	33	1.666667	1.001854	0.326585	-1.13342	1.1334186	0.180680783	-0.18068	0.540769	975	848	874	899	890	955	857	900.6667
SEN0709	84	35	51.33333	1.37561	0.118703	1.209205	1.209204721	0.274058516	0.274059	0.280518	153	122	135	136.6667	206	188	170	188
SEN0712	3	1	-0.33333	-1.2	1	-1.33675	1.336753136	0.418733061	-0.41873	1	2	1	3	2	2	3	0	1.666667
SEN0713	9	2	3.333333	1.625	0.306176	1.424343	1.424342917	0.510296523	0.510297	0.521303	5	8	3	5.333333	7	12	7	8.666667
SEN0714	21	5	7	1.241379	0.672382	1.091118	1.091118206	0.125807404	0.125807	0.823701	34	27	26	29	47	29	32	36
SEN0715	80	16	-45.3333	-1.73913	0.000184	-2.00407	2.004071142	1.002933723	-1.00293	0.001935	74	109	137	106.6667	58	69	57	61.33333
SEN0716	73	21	-24.3333	-1.19211	0.031178	-1.36197	1.361974999	0.445700221	-0.4457	0.110344	123	186	144	151	123	113	144	126.6667
SEN0722	17	8	10	1.405405	0.231504	1.235837	1.235837056	0.305488538	0.305489	0.438562	28	23	23	24.66667	40	33	31	34.66667
SEN0725	137	17	-37	-1.08831	0.088761	-1.23003	1.230026627	0.298689547	-0.29869	0.232036	516	473	379	456	414	413	430	419
SEN0735	2399	1452	1744	1.844552	0.002096	1.636759	1.636759447	0.710842306	0.710842	0.013516	2626	1949	1620	2065	4007	3401	4019	3809
SEN0744A	9	5	-0.33333	-1.05556	0.762016	-1.19216	1.192160775	0.253578811	-0.25358	0.882712	6	11	2	6.333333	9	7	2	6
SEN0755	33	20	22	2.157895	0.000513	1.897374	1.897373943	0.924004039	0.924004	0.004472	26	13	18	19	39	38	46	41
SEN0756	29	14	17.66667	1.5	0.069634	1.320544	1.320544486	0.401132903	0.401133	0.198295	37	41	28	35.33333	57	51	51	53
SEN0764	257	35	59	1.066591	0.635249	-1.06134	1.061335873	0.085881288	-0.08588	0.797616	971	953	734	886	991	918	926	945
SEN0780	86	6	35.33333	1.147632	0.983174	1.004246	1.004246099	0.006112857	0.006113	1	253	207	258	239.3333	293	259	272	274.6667
SEN0784	22	7	13	2.21875	0.004743	1.943732	1.943732134	0.958829414	0.958829	0.025759	15	8	9	10.66667	25	16	30	23.66667
SEN0785	16	5	10	2.111111	0.01604	1.842063	1.842063163	0.881322531	0.881323	0.066236	9	6	12	9	21	14	22	19
SEN0786	5	2	1.666667	2.25	0.396805	1.900895	1.90089494	0.926678798	0.926679	0.612972	1	3	0	1.333333	5	1	3	3
SEN0800	22	6	11.66667	1.564516	0.129642	1.373993	1.373993241	0.458374907	0.458375	0.296753	20	18	24	20.66667	31	40	26	32.33333
SEN0801	31	10	9	1.219512	0.711982	1.074707	1.074706989	0.103943373	0.103943	0.849432	51	27	45	41	41	51	58	50
SEN0802	34	18	21	1.512195	0.062892	1.332561	1.332561044	0.414201624	0.414202	0.184451	39	52	32	41	63	66	57	62
SEN0803	80	52	51.33333	1.933333	0.000539	1.715273	1.715272927	0.77843815	0.778438	0.004661	79	42	44	55	96	101	122	106.3333
SEN0804	75	61	56.33333	1.988304	0.000153	1.765295	1.765295132	0.819909401	0.819909	0.001676	82	45	44	57	106	115	119	113.3333
SEN0805	165	109	129	1.987245	3.77E-06	1.756067	1.756067141	0.812348005	0.812348	8.14E-05	153	124	115	130.6667	266	280	233	259.6667
SEN0806	215	151	153	2.153266	1.09E-05	1.920944	1.920943599	0.94181516	0.941815	0.000196	192	115	91	132.6667	285	266	306	285.6667
SEN0807	84	36	54.33333	1.605948	0.005404	1.414351	1.414351452	0.50014066	0.500141	0.028472	100	90	79	89.66667	143	126	163	144

Feature ID	Experiment - Range (original values)	Experiment - IQR (original values)	Experiment - Difference (original values)	Experiment - Fold Change (original values)	EDGE test: yccT NT vs WT NT , tagwise dispersions - P-value	EDGE test: yccT NT vs WT NT , tagwise dispersions - Fold change	yccT NT vs WT NT ABS|FC|	yccT NT vs WT NT Log2FC	yccT NT vs WT NT Log2FC +-	EDGE test: yccT NT vs WT NT , tagwise dispersions - FDR p-value correction	WT NT - WT.1.S22 - Expression values	WT NT - WT.2.S23 - Expression values	WT NT - WT.3.S24 - Expression values	WT NT - Means	yccT NT - yccT.1.S28 - Expression values	yccT NT - yccT.2.S29 - Expression values	yccT NT - yccT.3.S30 - Expression values	yccT NT - Means
SEN0813	122	14	-35.6667	-1.07477	0.097055	-1.22852	1.228521682	0.296923319	-0.29692	0.246061	487	550	501	512.6667	509	428	494	477
SEN0814	208	92	96.66667	1.250648	0.419057	1.10581	1.105809769	0.145103222	0.145103	0.632022	465	372	320	385.6667	528	455	464	482.3333
SEN0815	64	42	22.66667	1.104135	0.822894	-1.02844	1.028444198	0.040463517	-0.04046	0.924739	249	207	197	217.6667	250	210	261	240.3333
SEN0816	524	449	405.6667	1.94268	9.11E-05	1.731698	1.731697819	0.792187202	0.792187	0.001103	590	374	327	430.3333	851	834	823	836
SEN0829	33	20	24.33333	2.586957	1.94E-05	2.264578	2.26457841	1.179242493	1.179242	0.000318	16	15	15	15.33333	48	36	35	39.66667
SEN0830	25	9	14.66667	2.222222	0.003451	1.942065	1.942064855	0.95759138	0.957591	0.020257	13	10	13	12	35	22	23	26.66667
SEN0831	217	144	153.3333	1.484721	0.026113	1.31437	1.31437008	0.394371544	0.394372	0.096099	386	291	272	316.3333	489	485	435	469.6667
SEN0840	305	24	155	1.17864	0.783404	1.035382	1.035382201	0.050163422	0.050163	0.896736	900	921	782	867.6667	1084	897	1087	1022.667
SEN0853A	34	6	11	1.172775	0.805705	1.037991	1.037991014	0.053793955	0.053794	0.911195	75	67	49	63.66667	73	83	68	74.66667
SEN0853B	58	21	-11.3333	-1.11644	0.109545	-1.2672	1.267203767	0.341648529	-0.34165	0.266554	108	131	87	108.6667	118	101	73	97.33333
SEN0854	677	480	462	2.29291	0.000157	2.06442	2.064419594	1.045736229	1.045736	0.00171	599	256	217	357.3333	894	736	828	819.3333
SEN0855	410	261	310.6667	3.765579	8.16E-08	3.397663	3.397663279	1.764542883	1.764543	3E-06	208	70	59	112.3333	469	331	469	423
SEN0856	116	5	13.66667	1.044711	0.522663	-1.08065	1.080649164	0.111898224	-0.1119	0.718393	361	311	245	305.6667	329	316	313	319.3333
SEN0875	59	24	38.33333	2.095238	7.04E-05	1.851033	1.851032663	0.888330353	0.88833	0.000896	43	40	22	35	81	75	64	73.33333
SEN0876	95	11	-14.3333	-1.11111	0.165594	-1.29401	1.294012472	0.371851522	-0.37185	0.349903	107	121	202	143.3333	156	121	110	129
SEN0893	2126	495	200	1.056791	0.727653	-1.06028	1.060281364	0.084447159	-0.08445	0.860484	4713	3265	2587	3521.667	3847	3760	3558	3721.667
SEN0905	261	197	190.6667	1.77193	0.000694	1.575776	1.575775711	0.656062202	0.656062	0.005749	333	213	195	247	456	447	410	437.6667
SEN0906	28	27	24.66667	2.104478	0.000242	1.851367	1.851367085	0.888590978	0.888591	0.00235	28	20	19	22.33333	47	47	47	47
SEN0907	16	11	13	1.975	0.010963	1.733867	1.733866757	0.793993036	0.793993	0.049415	15	14	11	13.33333	27	27	25	26.33333
SEN0908A	34	16	14	1.344262	0.344266	1.190268	1.190268465	0.25128701	0.251287	0.55873	59	25	38	40.66667	52	58	54	54.66667
SEN0909	32	7	19	1.564356	0.045801	1.379194	1.379194149	0.463825559	0.463826	0.147062	38	35	28	33.66667	56	60	42	52.66667
SEN0910	27	14	10.66667	1.4	0.270944	1.243927	1.243926937	0.31490175	0.314902	0.484093	42	23	15	26.66667	37	36	39	37.33333
SEN0912	11	3	6.666667	2.538462	0.021948	2.201169	2.20116943	1.138270198	1.13827	0.083795	6	2	5	4.333333	12	8	13	11
SEN0912A	40	18	29.66667	2.098765	0.000186	1.838824	1.838823521	0.878783025	0.878783	0.001943	25	30	26	27	61	44	65	56.66667
SEN0913	14	2	3.333333	1.232558	0.855617	1.082676	1.082676474	0.114602201	0.114602	0.947992	15	15	13	14.33333	19	10	24	17.66667
SEN0914	16	11	9	2.588235	0.014264	2.272802	2.272802241	1.184472159	1.184472	0.060833	14	0	3	5.666667	15	13	16	14.66667
SEN0916A	51	35	25.33333	1.415301	0.144039	1.257886	1.257886452	0.331001697	0.331002	0.317057	90	50	43	61	94	85	80	86.33333
SEN0917	34	7	19.66667	1.475806	0.104505	1.295985	1.295984751	0.374048743	0.374049	0.25867	47	40	37	41.33333	69	43	71	61
SEN0920	55	20	31.33333	1.190283	0.702701	1.04875	1.048750305	0.06867123	0.068671	0.843813	170	171	153	164.6667	190	208	190	196
SEN0921	5	0	-2	-1.35294	0.287386	-1.52375	1.52374555	0.607622007	-0.60762	0.503523	10	6	7	7.666667	6	5	6	5.666667
SEN0925	184	66	120.3333	1.423212	0.054177	1.253814	1.253814387	0.326323789	0.326324	0.164992	315	292	246	284.3333	426	358	430	404.6667
SEN0943	249	69	-4	-1.00413	0.306358	-1.13685	1.136852509	0.185045097	-0.18505	0.521303	1104	956	855	971.6667	1008	982	913	967.6667
SEN0946	97	31	31	1.122208	0.921679	-1.01156	1.011556752	0.016577262	-0.01658	0.983818	289	252	220	253.6667	317	254	283	284.6667
SEN0947	327	79	128	1.135498	0.97916	1.003514	1.003513747	0.005060381	0.00506	1	1066	975	793	944.6667	1120	1044	1054	1072.667
SEN0957	205	33	97.66667	1.329584	0.333582	1.15355	1.153549839	0.206080336	0.20608	0.547444	239	309	341	296.3333	396	342	444	394
SEN0961	400	273	268	1.375525	0.114407	1.216553	1.216552833	0.282798976	0.282799	0.274031	863	663	615	713.6667	1015	936	994	981.6667

Feature ID	Experiment - Range (original values)	Experiment - IQR (original values)	Experiment - Difference (original values)	Experiment - Fold Change (original values)	EDGE test: yccT NT vs WT NT , tagwise dispersions - P-value	EDGE test: yccT NT vs WT NT , tagwise dispersions - Fold change	yccT NT vs WT NT ABS|FC|	yccT NT vs WT NT Log2FC	yccT NT vs WT NT Log2FC +-	EDGE test: yccT NT vs WT NT , tagwise dispersions - FDR p-value correction	WT NT - WT.1.S22 - Expression values	WT NT - WT.2.S23 - Expression values	WT NT - WT.3.S24 - Expression values	WT NT - Means	yccT NT - yccT.1.S28 - Expression values	yccT NT - yccT.2.S29 - Expression values	yccT NT - yccT.3.S30 - Expression values	yccT NT - Means
SEN0973	89	53	57.66667	1.301394	0.226524	1.149542	1.149542211	0.201059442	0.201059	0.431711	218	192	164	191.3333	249	245	253	249
SEN0974	53	30	36.33333	2.112245	0.000119	1.869555	1.86955545	0.902695262	0.902695	0.001356	46	31	21	32.66667	74	72	61	69
SEN0986	230	75	138.6667	1.501205	0.020875	1.323457	1.323457368	0.404311723	0.404312	0.080571	295	301	234	276.6667	412	370	464	415.3333
SEN0986A	2594	1109	1570	2.259358	0.000167	2.021815	2.0218155	1.01565135	1.015651	0.001805	1412	1633	695	1246.667	2521	2640	3289	2816.667
SEN0988	484	261	327	1.343608	0.202619	1.178627	1.178626884	0.237107079	0.237107	0.40027	909	1068	878	951.6667	1304	1170	1362	1278.667
SEN0989	8037	4082	-5514	-3.44813	4.92E-13	-3.92605	3.926053732	1.973079918	-1.97308	4.84E-11	6376	9999	6924	7766.333	2294	2501	1962	2252.333
SEN0990	6232	3336	-4346.67	-5.19023	0	-5.87497	5.874970352	2.554581571	-2.55458	0	4716	7057	4379	5384	1043	1244	825	1037.333
SEN0991	1606	768	-1188.33	-5.56466	9.2E-24	-6.38019	6.380189105	2.673599185	-2.6736	2.15E-21	1042	1811	1493	1448.667	274	302	205	260.3333
SEN0992	521	300	-430	-4.69628	8.58E-21	-5.38143	5.381434218	2.42799072	-2.42799	1.52E-18	407	615	617	546.3333	107	146	96	116.3333
SEN0993	254	143	-191	-4.08065	1.26E-23	-4.65737	4.657369082	2.219515216	-2.21952	2.79E-21	206	313	240	253	63	59	64	62
SEN0994	343	17	-126	-1.40909	0.001867	-1.58183	1.581825367	0.661590335	-0.66159	0.012405	602	360	340	434	341	324	259	308
SEN0995	49	40	30.66667	1.14557	1	1.000222	1.000221743	0.000319872	0.00032	1	198	198	236	210.6667	247	239	238	241.3333
SEN0996	73	37	45.66667	1.58547	0.009954	1.397131	1.397130517	0.4824668	0.482467	0.046135	95	67	72	78	122	109	140	123.6667
SEN0998	7	1	0.666667	1.08	0.89774	-1.05605	1.056050911	0.078679387	-0.07868	0.970927	5	8	12	8.333333	12	8	7	9
SEN0999	9	5	-0.66667	-1.125	0.543726	-1.2771	1.277102072	0.352873836	-0.35287	0.732623	2	7	9	6	5	10	1	5.333333
SEN1000	16	3	-2.33333	-1.19444	0.285109	-1.37352	1.373517121	0.457874894	-0.45787	0.50072	6	15	22	14.33333	14	11	11	12
SEN1001	16	8	3.333333	1.069444	0.727098	-1.06281	1.06281419	0.087889394	-0.08789	0.860317	50	43	51	48	43	52	59	51.33333
SEN1002	9	1	2.333333	1.132075	1	-1.00565	1.005648918	0.008126734	-0.00813	1	14	18	21	17.66667	18	23	19	20
SEN1003	25	1	7.666667	1.186992	0.815554	1.043185	1.043185474	0.060995685	0.060996	0.918821	45	34	44	41	44	43	59	48.66667
SEN1004	79	32	46.33333	1.208709	0.588542	1.06577	1.065770073	0.091896227	0.091896	0.76537	237	232	197	222	264	265	276	268.3333
SEN1005	12	4	3	1.230769	0.851231	1.08092	1.080920411	0.1122603	0.11226	0.945315	8	18	13	13	16	20	12	16
SEN1006	8	4	5	3.142857	0.016415	2.687943	2.687943007	1.426502549	1.426503	0.067409	5	1	1	2.333333	9	9	4	7.333333
SEN1007	14	2	6	3.571429	0.016698	3.036296	3.036295665	1.602312283	1.602312	0.068131	4	1	2	2.333333	15	6	4	8.333333
SEN1008	6	3	2	1.545455	0.585944	1.35193	1.351930388	0.435020868	0.435021	0.764523	7	1	3	3.666667	5	6	6	5.666667
SEN1009	7	4	3.666667	2.222222	0.099242	1.926254	1.926254379	0.945798236	0.945798	0.249491	6	2	1	3	5	8	7	6.666667
SEN1010	69	25	35.33333	2.039216	0.002086	1.823627	1.823627345	0.866810947	0.866811	0.013493	50	32	20	34	62	89	57	69.33333
SEN1013	57	31	35.33333	1.595506	0.009795	1.409955	1.409955427	0.495649556	0.49565	0.045637	74	60	44	59.33333	101	92	91	94.66667
SEN1013A	2	1	1	4	0.378682	2.867909	2.867909423	1.51999946	1.519999	0.595321	1	0	0	0.333333	1	1	2	1.333333
SEN1013B	8	2	2.666667	1.727273	0.381707	1.50353	1.503530413	0.588354051	0.588354	0.597859	3	6	2	3.666667	10	5	4	6.333333
SEN1016	80	30	28	1.298932	0.351001	1.155178	1.155178257	0.208115492	0.208115	0.566409	131	91	59	93.66667	139	105	121	121.6667
SEN1022	237	124	134	1.302711	0.248403	1.156255	1.156254716	0.20945925	0.209459	0.459749	548	424	356	442.6667	593	590	547	576.6667
SEN1023	149	49	89.33333	1.41875	0.056436	1.24793	1.247930271	0.319537324	0.319537	0.17009	240	211	189	213.3333	338	260	310	302.6667
SEN1026	39	8	8.666667	1.06701	0.587819	-1.06769	1.067690696	0.094493766	-0.09449	0.76537	134	131	123	129.3333	161	131	122	138
SEN1041A	16	3	-2.66667	-1.125	0.278685	-1.28594	1.285941883	0.362825443	-0.36283	0.492563	22	17	33	24	20	19	25	21.33333
SEN1063	41	25	34.33333	2.256098	1.97E-05	1.984735	1.984735136	0.988946492	0.988946	0.000322	27	29	26	27.33333	52	66	67	61.66667
SEN1065	205	98	131	1.275596	0.345011	1.123995	1.123994933	0.168635531	0.168636	0.559569	489	524	413	475.3333	614	587	618	606.3333

Feature ID	Experiment - Range (original values)	Experiment - IQR (original values)	Experiment - Difference (original values)	Experiment - Fold Change (original values)	EDGE test: yccT NT vs WT NT , tagwise dispersions - P-value	EDGE test: yccT NT vs WT NT , tagwise dispersions - Fold change	yccT NT vs WT NT ABS|FC|	yccT NT vs WT NT Log2FC	yccT NT vs WT NT Log2FC +-	EDGE test: yccT NT vs WT NT , tagwise dispersions - FDR p-value correction	WT NT - WT.1.S22 - Expression values	WT NT - WT.2.S23 - Expression values	WT NT - WT.3.S24 - Expression values	WT NT - Means	yccT NT - yccT.1.S28 - Expression values	yccT NT - yccT.2.S29 - Expression values	yccT NT - yccT.3.S30 - Expression values	yccT NT - Means
SEN1066	141	66	97	1.460443	0.042127	1.276822	1.276821509	0.35255686	0.352557	0.138271	210	206	216	210.6667	347	276	300	307.6667
SEN1071	135	38	-83.3333	-1.87108	6.29E-06	-2.13802	2.138019232	1.096274831	-1.09627	0.000126	131	216	190	179	81	113	93	95.66667
SEN1072	39	14	4.666667	1.040698	0.484176	-1.09651	1.096505207	0.132912663	-0.13291	0.688948	125	96	123	114.6667	135	114	109	119.3333
SEN1092	95	16	-45	-1.22804	0.007191	-1.40505	1.405050169	0.490621645	-0.49062	0.035641	225	260	242	242.3333	209	165	218	197.3333
SEN1093	88	28	32.33333	1.151563	0.873471	1.020142	1.020141623	0.028769451	0.028769	0.958007	256	216	168	213.3333	253	240	244	245.6667
SEN1107	13	4	6	1.642857	0.199455	1.443481	1.443480703	0.529551821	0.529552	0.396672	9	13	6	9.333333	16	19	11	15.33333
SEN1126	153	105	106.6667	1.661157	0.003224	1.467514	1.467513582	0.553373856	0.553374	0.019226	206	129	149	161.3333	282	268	254	268
SEN1129	709	170	-107.667	-1.07283	0.166593	-1.21371	1.213707383	0.279420639	-0.27942	0.351334	1639	1914	1205	1586	1565	1395	1475	1478.333
SEN1130	242	29	17	1.027041	0.445601	-1.10561	1.105607805	0.144839706	-0.14484	0.655966	630	749	507	628.6667	726	601	610	645.6667
SEN1131	96	42	56.66667	1.584192	0.008503	1.397212	1.397212139	0.482551082	0.482551	0.040907	122	89	80	97	176	131	154	153.6667
SEN1132	13	3	7	2	0.07423	1.744203	1.744202714	0.802567722	0.802568	0.206344	7	6	8	7	19	10	13	14
SEN1133	302	118	169.6667	1.255522	0.39713	1.108282	1.108281847	0.148324819	0.148325	0.61313	750	672	570	664	872	790	839	833.6667
SEN1134	158	37	80.66667	1.204392	0.614494	1.06317	1.063169564	0.088371709	0.088372	0.782871	443	406	335	394.6667	490	443	493	475.3333
SEN1135	122	45	9	1.035294	0.526556	-1.08903	1.089030134	0.123043875	-0.12304	0.720782	299	289	177	255	298	250	244	264
SEN1136	90	37	52.66667	1.210948	0.572439	1.068103	1.068102985	0.095050756	0.095051	0.751561	265	259	225	249.6667	296	315	296	302.3333
SEN1137	26	2	8.333333	1.078125	0.707542	-1.05153	1.051531002	0.072491385	-0.07249	0.8471	112	117	91	106.6667	114	117	114	115
SEN1138	14	4	4.333333	1.342105	0.571598	1.177053	1.177052692	0.235178906	0.235179	0.75089	11	17	10	12.66667	24	12	15	17
SEN1139	31	11	20.33333	1.398693	0.138354	1.228805	1.228804737	0.297255683	0.297256	0.30853	50	57	46	51	76	61	77	71.33333
SEN1140	1723	1625	1442.667	2.261808	1.1E-05	2.016655	2.016654722	1.011964097	1.011964	0.000197	1608	896	926	1143.333	2588	2619	2551	2586
SEN1141	78	58	57.66667	1.729958	0.000992	1.528473	1.528472664	0.61209075	0.612091	0.007568	96	69	72	79	130	147	133	136.6667
SEN1142	286	130	183	1.729084	0.000718	1.532324	1.532324249	0.615721613	0.615722	0.005913	284	279	190	251	417	409	476	434
SEN1143	124	73	88.33333	2.007605	8.38E-06	1.775527	1.775526582	0.82824696	0.828247	0.000161	113	82	68	87.66667	181	155	192	176
SEN1144	12	8	9.333333	2.866667	0.002208	2.489258	2.489257787	1.315715643	1.315716	0.014025	6	4	5	5	14	13	16	14.33333
SEN1145	25	7	7.333333	1.213592	0.703916	1.068452	1.068452389	0.095522622	0.095523	0.845043	39	36	28	34.33333	53	40	32	41.66667
SEN1146	19	7	11.33333	1.653846	0.057687	1.456798	1.456798092	0.542800938	0.542801	0.172826	20	21	11	17.33333	29	30	27	28.66667
SEN1150	74	18	37.66667	1.312155	0.246656	1.160056	1.160056479	0.214195047	0.214195	0.45756	139	121	102	120.6667	169	176	130	158.3333
SEN1151	19	6	4	1.2	0.885782	1.054099	1.054099348	0.076010847	0.076011	0.964513	25	15	20	20	34	22	16	24
SEN1154	6	3	3.666667	3.2	0.046992	2.701949	2.701948719	1.434000294	1.434	0.150016	4	1	0	1.666667	6	4	6	5.333333
SEN1155	28	15	14	1.545455	0.11045	1.362361	1.362360656	0.446108676	0.446109	0.267581	36	21	20	25.66667	42	29	48	39.66667
SEN1156	1	0	0	-1	1	-1.0967	1.096698864	0.133167439	-0.13317	1	0	1	0	0.333333	0	1	0	0.333333
SEN1157	7	4	5	3.5	0.011509	2.969487	2.969487369	1.570213896	1.570214	0.051513	3	2	1	2	7	8	6	7
SEN1158	42	27	30.66667	2.957447	2.59E-07	2.59232	2.592319965	1.374243798	1.374244	8.5E-06	21	13	13	15.66667	55	40	44	46.33333
SEN1160	14	4	6.666667	1.740741	0.13105	1.531009	1.531008794	0.61448257	0.614483	0.298366	12	9	6	9	13	20	14	15.66667
SEN1162	14	5	4.333333	1.52	0.313245	1.343243	1.343242992	0.425720311	0.42572	0.527952	15	6	4	8.333333	9	11	18	12.66667
SEN1163	9	0	4.666667	2.076923	0.126149	1.805577	1.805577162	0.852460076	0.85246	0.29195	5	3	5	4.333333	12	10	5	9
SEN1164	24	19	13.33333	1.555556	0.10497	1.380037	1.380037302	0.464707263	0.464707	0.259265	38	19	15	24	35	38	39	37.33333

Feature ID	Experiment - Range (original values)	Experiment - IQR (original values)	Experiment - Difference (original values)	Experiment - Fold Change (original values)	EDGE test: yccT NT vs WT NT , tagwise dispersions - P-value	EDGE test: yccT NT vs WT NT , tagwise dispersions - Fold change	yccT NT vs WT NT ABS|FC|	yccT NT vs WT NT Log2FC	yccT NT vs WT NT Log2FC +-	EDGE test: yccT NT vs WT NT , tagwise dispersions - FDR p-value correction	WT NT - WT.1.S22 - Expression values	WT NT - WT.2.S23 - Expression values	WT NT - WT.3.S24 - Expression values	WT NT - Means	yccT NT - yccT.1.S28 - Expression values	yccT NT - yccT.2.S29 - Expression values	yccT NT - yccT.3.S30 - Expression values	yccT NT - Means
SEN1170A	7	4	4.333333	7.5	0.005015	5.763159	5.763158753	2.526859761	2.52686	0.026706	0	2	0	0.666667	7	4	4	5
SEN1171	8	5	4	2.333333	0.073149	2.020002	2.020002455	1.014357046	1.014357	0.204494	7	0	2	3	6	7	8	7
SEN1171A	17	6	10	2.034483	0.0209	1.783203	1.78320264	0.834470657	0.834471	0.0806	12	11	6	9.666667	23	17	19	19.66667
SEN1171B	5	3	2.333333	2.166667	0.249774	1.860925	1.860924849	0.896019795	0.89602	0.460953	4	1	1	2	4	6	3	4.333333
SEN1172	20	2	10	1.428571	0.2332	1.251903	1.251903083	0.324122879	0.324123	0.440457	21	25	24	23.33333	41	26	33	33.33333
SEN1173	7	4	4	2.714286	0.088913	2.320856	2.320856026	1.214657028	1.214657	0.232091	1	1	5	2.333333	3	8	8	6.333333
SEN1175	9	1	2.333333	1.241379	0.817216	1.090848	1.090847992	0.125450078	0.12545	0.920224	10	11	8	9.666667	17	10	9	12
SEN1176	107	31	50.66667	1.550725	0.021956	1.37012	1.370119713	0.454301953	0.454302	0.083795	116	93	67	92	174	124	130	142.6667
SEN1177	281	158	168	1.626866	0.00741	1.435806	1.435805614	0.521860443	0.52186	0.036646	349	208	247	268	489	405	414	436
SEN1178	25	9	13	1.590909	0.08396	1.39872	1.39872034	0.484107539	0.484108	0.224231	28	14	24	22	33	33	39	35
SEN1179	30	7	16.33333	1.283237	0.41179	1.127093	1.127092895	0.172606428	0.172606	0.62552	61	52	60	57.66667	73	67	82	74
SEN1180	165	107	112.6667	1.877922	0.0001	1.664957	1.664957266	0.735485149	0.735485	0.001187	174	113	98	128.3333	263	220	240	241
SEN1181	143	60	80	2.6	0.000258	2.324429	2.324428605	1.216876114	1.216876	0.002472	81	22	47	50	107	165	118	130
SEN1183	25	5	-8.33333	-1.07225	0.104789	-1.2183	1.218304628	0.284874914	-0.28487	0.258962	136	113	122	123.6667	117	118	111	115.3333
SEN1186	104	73	59.66667	1.890547	0.014731	1.708988	1.708988342	0.773142556	0.773143	0.062404	121	49	31	67	135	122	123	126.6667
SEN1188	156	82	14.33333	1.014626	0.352818	-1.12406	1.124055461	0.16871322	-0.16871	0.567661	976	1043	921	980	1068	912	1003	994.3333
SEN1193	826	453	519.3333	1.375784	0.142221	1.21314	1.213139705	0.2787457	0.278746	0.314245	1625	1290	1231	1382	2057	1743	1904	1901.333
SEN1194	217	130	156	1.844765	0.00018	1.631341	1.631340727	0.70605814	0.706058	0.001904	242	158	154	184.6667	363	288	371	340.6667
SEN1196	173	90	102	1.836066	0.001924	1.647953	1.647952681	0.720674818	0.720675	0.012707	174	123	69	122	217	242	213	224
SEN1198	738	464	418.3333	1.351541	0.203401	1.194355	1.194355485	0.2562323	0.256232	0.401278	1519	996	1055	1190	1734	1442	1649	1608.333
SEN1201	400	247	272.6667	1.422739	0.063032	1.252851	1.252851444	0.325215358	0.325215	0.184672	723	625	587	645	987	872	894	917.6667
SEN1204	115	50	73.66667	1.267879	0.393243	1.110236	1.110236484	0.150867008	0.150867	0.61	260	303	262	275	375	312	359	348.6667
SEN1210	81	11	44.66667	1.301802	0.244868	1.145832	1.145832478	0.196396136	0.196396	0.455504	160	157	127	148	208	168	202	192.6667
SEN1227	177	116	113.6667	1.704545	0.008435	1.516579	1.516578781	0.600820443	0.60082	0.040624	237	110	137	161.3333	287	285	253	275
SEN1228	212	84	129.6667	1.192574	0.691326	1.050701	1.050701124	0.071352347	0.071352	0.835588	722	668	630	673.3333	815	842	752	803
SEN1229	57	12	20.33333	1.12249	0.924904	-1.01289	1.012891142	0.018479133	-0.01848	0.984919	183	150	165	166	177	175	207	186.3333
SEN1242	7783	2316	-4238.33	-2.50384	5.33E-07	-2.85235	2.852354556	1.512153324	-1.51215	1.58E-05	5035	10375	5760	7056.667	2592	2719	3144	2818.333
SEN1244	76	57	48.33333	1.421512	0.082121	1.260079	1.260079122	0.333514326	0.333514	0.221068	153	96	95	114.6667	171	168	150	163
SEN1245	70	42	47	1.60515	0.00566	1.415794	1.415794459	0.501611835	0.501612	0.029508	94	71	68	77.66667	123	113	138	124.6667
SEN1249	35	32	25	1.487013	0.056539	1.313696	1.313695532	0.393630948	0.393631	0.170194	68	42	44	51.33333	77	76	76	76.33333
SEN1250	166	142	126.3333	1.915459	0.000158	1.705611	1.705611032	0.770288674	0.770289	0.001718	197	112	105	138	271	268	254	264.3333
SEN1251	76	61	58	1.701613	0.001006	1.502377	1.50237722	0.587247093	0.587247	0.007648	100	76	72	82.66667	137	137	148	140.6667
SEN1253	24	8	14.33333	1.364407	0.246962	1.201923	1.201923116	0.265344613	0.265345	0.457862	42	39	37	39.33333	53	61	47	53.66667
SEN1254	51	4	14.66667	1.261905	0.485834	1.119263	1.119262886	0.162548928	0.162549	0.690863	70	61	37	56	64	60	88	70.66667
SEN1279	228	144	117	1.224856	0.512985	1.086	1.085999644	0.119023631	0.119024	0.713221	637	493	431	520.3333	659	653	600	637.3333
SEN1294	547	253	359	1.394795	0.115609	1.224462	1.224461946	0.292147938	0.292148	0.276385	1026	807	895	909.3333	1354	1148	1303	1268.333

Feature ID	Experiment - Range (original values)	Experiment - IQR (original values)	Experiment - Difference (original values)	Experiment - Fold Change (original values)	EDGE test: yccT NT vs WT NT , tagwise dispersions - P-value	EDGE test: yccT NT vs WT NT , tagwise dispersions - Fold change	yccT NT vs WT NT ABS|FC|	yccT NT vs WT NT Log2FC	yccT NT vs WT NT Log2FC +-	EDGE test: yccT NT vs WT NT , tagwise dispersions - FDR p-value correction	WT NT - WT.1.S22 - Expression values	WT NT - WT.2.S23 - Expression values	WT NT - WT.3.S24 - Expression values	WT NT - Means	yccT NT - yccT.1.S28 - Expression values	yccT NT - yccT.2.S29 - Expression values	yccT NT - yccT.3.S30 - Expression values	yccT NT - Means
SEN1301	197	97	137	1.299345	0.298001	1.137182	1.137182207	0.185463431	0.185463	0.513591	467	432	474	457.6667	629	564	591	594.6667
SEN1303	1417	715	118.3333	1.026045	0.502718	-1.12443	1.1244258	0.169188462	-0.16919	0.704228	3780	4653	5197	4543.333	4847	4132	5006	4661.667
SEN1335	8	2	1.333333	1.040816	0.640149	-1.09362	1.093616127	0.129106422	-0.12911	0.801498	30	31	37	32.66667	33	38	31	34
SEN1336	551	234	287	1.236668	0.491322	1.094961	1.0949613	0.130879881	0.13088	0.69621	1425	1191	1022	1212.667	1573	1548	1378	1499.667
SEN1355	218	118	-30	-1.03328	0.201906	-1.17432	1.174319153	0.231824553	-0.23182	0.399218	954	1029	811	931.3333	941	823	940	901.3333
SEN1356	215	108	120	1.519481	0.032366	1.357042	1.357041684	0.440465037	0.440465	0.113101	317	222	154	231	369	354	330	351
SEN1357	266	138	201.3333	1.760705	0.000461	1.543668	1.543668226	0.626362713	0.626363	0.004108	289	241	264	264.6667	489	402	507	466
SEN1358	26	10	1.666667	1.022523	0.448638	-1.11759	1.117589892	0.160390878	-0.16039	0.658687	61	86	75	74	75	65	87	75.66667
SEN1359	68	12	22	1.147321	0.958158	1.008694	1.008693597	0.012488005	0.012488	1	154	166	128	149.3333	196	153	165	171.3333
SEN1360	69	17	36	1.285714	0.287308	1.132246	1.132245874	0.179187281	0.179187	0.503523	136	134	108	126	177	151	158	162
SEN1360A	625	569	547.3333	2.043865	3.51E-06	1.805547	1.805546868	0.85243587	0.852436	7.85E-05	635	459	479	524.3333	1083	1048	1084	1071.667
SEN1361	7	3	2.666667	1.571429	0.427575	1.376996	1.376996116	0.46152449	0.461524	0.639847	7	3	4	4.666667	4	8	10	7.333333
SEN1362	4	2	1.666667	1.454545	0.709926	1.271637	1.271636779	0.346686649	0.346687	0.847948	3	5	3	3.666667	5	4	7	5.333333
SEN1363	46	23	29.66667	1.649635	0.014791	1.460643	1.460642789	0.546603399	0.546603	0.062518	58	40	39	45.66667	63	85	78	75.33333
SEN1364	5	1	2.666667	1.444444	0.559456	1.267165	1.267165085	0.341604489	0.341604	0.744053	7	6	5	6	10	10	6	8.666667
SEN1365	40	16	17.66667	1.504762	0.137933	1.321194	1.321194025	0.40184235	0.401842	0.3079	28	47	30	35	46	44	68	52.66667
SEN1365A	82	27	48	1.195918	0.72719	1.047198	1.047198455	0.066534874	0.066535	0.860317	223	265	247	245	305	300	274	293
SEN1366	99	30	35	1.217391	0.594098	1.075213	1.075212678	0.104622055	0.104622	0.770171	160	202	121	161	190	178	220	196
SEN1367	137	81	97.33333	1.362733	0.125437	1.196638	1.196638048	0.258986841	0.258987	0.290848	285	263	257	268.3333	394	344	359	365.6667
SEN1374	135	79	101	2.01	1.65E-06	1.772431	1.772431207	0.825729634	0.82573	4.07E-05	118	100	82	100	217	179	207	201
SEN1375	285	156	182.6667	1.411103	0.07346	1.248674	1.248674286	0.320397202	0.320397	0.204975	544	420	369	444.3333	654	576	651	627
SEN1378	29	6	11.33333	1.62963	0.132175	1.431201	1.43120076	0.517226058	0.517226	0.299576	16	22	16	18	21	22	45	29.33333
SEN1379	21	3	9.666667	1.337209	0.335467	1.176226	1.17622568	0.234164894	0.234165	0.549723	32	31	23	28.66667	44	29	42	38.33333
SEN1380	3	1	0.666667	1.222222	1	1.07449	1.074489724	0.103651686	0.103652	1	5	2	2	3	3	3	5	3.666667
SEN1381	4	1	0.333333	1.333333	1	1.155671	1.155671168	0.208730956	0.208731	1	1	1	1	1	0	0	4	1.333333
SEN1382	90	35	68.66667	1.469248	0.042061	1.290369	1.290369374	0.367784103	0.367784	0.138271	150	142	147	146.3333	231	232	182	215
SEN1383	90	23	-8	-1.02746	0.20677	-1.16544	1.165441782	0.220876938	-0.22088	0.406117	353	268	277	299.3333	291	263	320	291.3333
SEN1384	49	27	31	2.328571	0.000164	2.049882	2.049881674	1.035540635	1.035541	0.001768	36	12	22	23.33333	61	49	53	54.33333
SEN1385	506	433	406	2.298507	2.65E-07	2.044433	2.044433337	1.031701022	1.031701	8.63E-06	418	257	263	312.6667	696	763	697	718.6667
SEN1386	382	298	306.3333	2.181234	3.4E-07	1.932361	1.932361306	0.950364869	0.950365	1.06E-05	317	232	229	259.3333	556	611	530	565.6667
SEN1387	5982	3671	3327.667	1.285686	0.439695	1.138934	1.138933939	0.18768407	0.187684	0.651166	14160	10740	10044	11648	14490	14411	16026	14975.67
SEN1388	28	14	15	1.517241	0.082575	1.339156	1.339155804	0.421323821	0.421324	0.221887	40	26	21	29	45	38	49	44
SEN1389	48	23	29.66667	1.712	0.003685	1.506415	1.506415255	0.591119515	0.59112	0.021262	50	40	35	41.66667	83	63	68	71.33333
SEN1390	11	2	3	1.5625	0.382412	1.374546	1.374545905	0.458955089	0.458955	0.598752	8	6	2	5.333333	7	13	5	8.333333
SEN1391	14	1	8	1.533333	0.181029	1.343925	1.343925072	0.426452706	0.426453	0.369874	15	14	16	15	25	16	28	23
SEN1392	51	39	36	1.864	0.000965	1.649561	1.649561384	0.722082465	0.722082	0.007449	59	35	31	41.66667	77	74	82	77.66667

Feature ID	Experiment - Range (original values)	Experiment - IQR (original values)	Experiment - Difference (original values)	Experiment - Fold Change (original values)	EDGE test: yccT NT vs WT NT , tagwise dispersions - P-value	EDGE test: yccT NT vs WT NT , tagwise dispersions - Fold change	yccT NT vs WT NT ABS|FC|	yccT NT vs WT NT Log2FC	yccT NT vs WT NT Log2FC +-	EDGE test: yccT NT vs WT NT , tagwise dispersions - FDR p-value correction	WT NT - WT.1.S22 - Expression values	WT NT - WT.2.S23 - Expression values	WT NT - WT.3.S24 - Expression values	WT NT - Means	yccT NT - yccT.1.S28 - Expression values	yccT NT - yccT.2.S29 - Expression values	yccT NT - yccT.3.S30 - Expression values	yccT NT - Means
SEN1393	3	0	0.333333	1.125	1	-1.00918	1.009180032	0.013183566	-0.01318	1	4	2	2	2.666667	5	2	2	3
SEN1394	7	1	3	1.45	0.492767	1.272066	1.272065932	0.347173448	0.347173	0.697625	7	7	6	6.666667	8	8	13	9.666667
SEN1395	6	4	3.333333	2.428571	0.103388	2.088018	2.088017865	1.062134056	1.062134	0.257222	5	1	1	2.333333	6	7	4	5.666667
SEN1396	5	1	2.333333	3.333333	0.152967	2.732744	2.73274441	1.450350533	1.450351	0.329885	2	1	0	1	5	2	3	3.333333
SEN1398	91	46	51.33333	1.709677	0.004186	1.521254	1.521253813	0.605260879	0.605261	0.023331	100	69	48	72.33333	115	117	139	123.6667
SEN1399	398	228	239	1.446451	0.054082	1.286069	1.286069408	0.362968506	0.362969	0.164926	671	525	410	535.3333	762	753	808	774.3333
SEN1402	49	40	0	-1	0.391603	-1.14634	1.146335372	0.19702918	-0.19703	0.60865	102	142	145	129.6667	138	150	101	129.6667
SEN1403	85	48	19	1.050622	0.501291	-1.08885	1.088852392	0.122808391	-0.12281	0.70377	380	346	400	375.3333	400	352	431	394.3333
SEN1409	45	23	26.66667	1.160321	0.855423	1.022989	1.022989089	0.032790758	0.032791	0.947992	181	169	149	166.3333	194	193	192	193
SEN1410	45	16	11.66667	1.060659	0.532419	-1.07802	1.078016183	0.108378836	-0.10838	0.724554	183	200	194	192.3333	228	184	200	204
SEN1414	130	50	79.33333	1.565321	0.03193	1.362362	1.362362198	0.446110309	0.44611	0.1122	110	153	158	140.3333	216	203	240	219.6667
SEN1415	18	4	8.333333	1.480769	0.250983	1.303505	1.303504984	0.382396099	0.382396	0.462222	14	21	17	17.33333	17	32	28	25.66667
SEN1417	9	5	6.333333	1.575758	0.1846	1.382932	1.382931921	0.467730137	0.46773	0.373669	11	12	10	11	19	17	16	17.33333
SEN1418	21	10	13.66667	2.518519	0.001294	2.211054	2.211053648	1.14473403	1.144734	0.009367	13	9	5	9	19	26	23	22.66667
SEN1419	15	4	4	1.266667	0.604466	1.121538	1.121537885	0.165478355	0.165478	0.777246	22	15	8	15	15	19	23	19
SEN1420	15	4	-2	-1.0438	0.255238	-1.18394	1.183938095	0.243593648	-0.24359	0.467339	57	44	42	47.66667	49	42	46	45.66667
SEN1421	28	6	9.333333	1.622222	0.1505	1.432701	1.432701324	0.518737881	0.518738	0.326308	21	15	9	15	21	15	37	24.33333
SEN1423	4	1	1.666667	1.714286	0.491125	1.484806	1.484805564	0.570274022	0.570274	0.696153	3	2	2	2.333333	6	4	2	4
SEN1423A	38	19	25.66667	2.425926	8.16E-05	2.138403	2.138402506	1.096533434	1.096533	0.001004	28	16	10	18	48	35	48	43.66667
SEN1424	19	3	0.666667	1.021053	0.573635	-1.11841	1.118407288	0.161445667	-0.16145	0.752825	26	40	29	31.66667	44	25	28	32.33333
SEN1425	289	124	-163	-1.49344	7.96E-05	-1.70257	1.702569933	0.767714058	-0.76771	0.000983	479	578	423	493.3333	403	299	289	330.3333
SEN1428	281	222	216.6667	1.425671	0.062265	1.254729	1.254729496	0.327376371	0.327376	0.183077	567	476	484	509	757	714	706	725.6667
SEN1429	126	41	77.33333	1.370607	0.13596	1.20184	1.201839559	0.265244315	0.265244	0.305187	233	179	214	208.6667	305	255	298	286
SEN1431	84	45	58.66667	5.756757	6.78E-09	5.122289	5.122288871	2.356788616	2.356789	3.34E-07	27	5	5	12.33333	74	50	89	71
SEN1432	394	213	230	1.297799	0.265987	1.146205	1.146205257	0.196865418	0.196865	0.478128	908	739	670	772.3333	1064	952	991	1002.333
SEN1433	44	24	-2.66667	-1.03042	0.269067	-1.16707	1.167074485	0.222896639	-0.2229	0.481907	112	90	69	90.33333	102	68	93	87.66667
SEN1434	28	11	-13	-1.29771	0.009057	-1.47184	1.471843124	0.55762391	-0.55762	0.043058	64	58	48	56.66667	53	42	36	43.66667
SEN1435	39	9	12.66667	1.136201	1	1.004001	1.004000992	0.005760695	0.005761	1	105	101	73	93	112	109	96	105.6667
SEN1436	75	41	-33	-1.16473	0.017626	-1.32675	1.326750202	0.407896769	-0.4079	0.070967	240	251	209	233.3333	233	176	192	200.3333
SEN1437	108	53	64.66667	1.244332	0.419131	1.099052	1.099052334	0.136260085	0.13626	0.632022	303	259	232	264.6667	340	336	312	329.3333
SEN1438	168	117	127	1.487836	0.018921	1.314253	1.314252924	0.394242945	0.394243	0.074856	295	263	223	260.3333	391	380	391	387.3333
SEN1439	151	92	99.66667	1.925697	9.04E-05	1.701682	1.701681834	0.766961319	0.766961	0.001098	137	87	99	107.6667	191	193	238	207.3333
SEN1440	140	81	97.33333	1.869048	0.000468	1.658399	1.658398822	0.729790997	0.729791	0.004162	149	85	102	112	183	225	220	209.3333
SEN1441	255	211	193.6667	1.897991	9.52E-05	1.687475	1.687475021	0.754866146	0.754866	0.001143	292	185	170	215.6667	407	396	425	409.3333
SEN1442	547	463	418.3333	2.772599	1.09E-06	2.489012	2.489011666	1.315572992	1.315573	2.95E-05	390	157	161	236	704	624	635	654.3333
SEN1443	151	131	117	2.897297	7.45E-06	2.607924	2.607924261	1.382901972	1.382902	0.000145	112	34	39	61.66667	181	185	170	178.6667

Feature ID	Experiment - Range (original values)	Experiment - IQR (original values)	Experiment - Difference (original values)	Experiment - Fold Change (original values)	EDGE test: yccT NT vs WT NT , tagwise dispersions - P-value	EDGE test: yccT NT vs WT NT , tagwise dispersions - Fold change	yccT NT vs WT NT ABS|FC|	yccT NT vs WT NT Log2FC	yccT NT vs WT NT Log2FC +-	EDGE test: yccT NT vs WT NT , tagwise dispersions - FDR p-value correction	WT NT - WT.1.S22 - Expression values	WT NT - WT.2.S23 - Expression values	WT NT - WT.3.S24 - Expression values	WT NT - Means	yccT NT - yccT.1.S28 - Expression values	yccT NT - yccT.2.S29 - Expression values	yccT NT - yccT.3.S30 - Expression values	yccT NT - Means
SEN1444	231	219	173	1.804651	0.001862	1.612784	1.612784314	0.689553512	0.689554	0.012391	320	164	161	215	383	389	392	388
SEN1449	293	151	38	1.014388	0.441658	-1.12568	1.125679249	0.170795804	-0.1708	0.652901	2493	2786	2644	2641	2742	2723	2572	2679
SEN1450	74	16	37.33333	1.155989	0.909787	1.014489	1.014489027	0.020753261	0.020753	0.977276	245	243	230	239.3333	304	267	259	276.6667
SEN1469	133	60	68.66667	1.273572	0.320067	1.13148	1.131480418	0.178211617	0.178212	0.534974	308	248	197	251	302	330	327	319.6667
SEN1470	9	1	5	1.227273	0.713212	1.079565	1.079565372	0.110450607	0.110451	0.849756	22	23	21	22	30	29	22	27
SEN1472	304	78	-93	-1.09248	0.094869	-1.23534	1.235337096	0.304904775	-0.3049	0.242007	1220	1160	916	1098.667	1038	1019	960	1005.667
SEN1480	129	73	38.66667	1.245243	0.497223	1.112707	1.11270728	0.154074113	0.154074	0.699966	240	122	111	157.6667	207	187	195	196.3333
SEN1482	444	294	364.3333	1.712052	0.000982	1.504352	1.504352005	0.589142184	0.589142	0.007535	569	482	484	511.6667	926	778	924	876
SEN1493	501	336	274	1.73722	0.016233	1.569015	1.56901531	0.649859429	0.649859	0.066846	604	268	243	371.6667	626	744	567	645.6667
SEN1494	8	4	5.666667	1.242857	0.666149	1.093412	1.093412146	0.128837306	0.128837	0.819919	24	24	22	23.33333	30	28	29	29
SEN1495	33	30	19	1.2375	0.497022	1.092714	1.092713534	0.127915233	0.127915	0.699966	101	69	70	80	102	95	100	99
SEN1496	85	52	45.66667	1.303097	0.242262	1.153353	1.153352743	0.205833816	0.205834	0.452335	190	138	124	150.6667	209	193	187	196.3333
SEN1497	227	131	110.6667	1.243223	0.468424	1.09668	1.096680332	0.133143061	0.133143	0.676072	553	390	422	455	617	557	523	565.6667
SEN1498	278	160	150.6667	1.296978	0.287089	1.143441	1.143440596	0.193381417	0.193381	0.503523	610	448	464	507.3333	726	624	624	658
SEN1499	454	276	246.6667	1.36963	0.151747	1.222808	1.22280786	0.290197731	0.290198	0.328155	898	622	482	667.3333	934	936	872	914
SEN1500	608	131	90	1.09003	0.84291	-1.02888	1.028882609	0.041078386	-0.04108	0.94008	1335	937	727	999.6667	1145	1068	1056	1089.667
SEN1501	28	5	9.666667	1.345238	0.377906	1.192273	1.192272927	0.253714526	0.253715	0.594928	32	27	25	28	27	53	33	37.66667
SEN1502	10	3	6	3	0.021475	2.575506	2.57550629	1.364856063	1.364856	0.082316	2	4	3	3	12	6	9	9
SEN1503	15	2	7	1.5	0.248603	1.31603	1.316029668	0.396192013	0.396192	0.459749	14	16	12	14	27	16	20	21
SEN1504	55	17	20.66667	1.141553	1	1.001252	1.001251513	0.001804423	0.001804	1	141	161	136	146	191	158	151	166.6667
SEN1505	47	25	-31.6667	-1.25066	0.004845	-1.42376	1.423756365	0.509702292	-0.5097	0.026119	155	156	163	158	116	133	130	126.3333
SEN1506	27	12	17	1.520408	0.062893	1.342319	1.342319029	0.424727598	0.424728	0.184451	40	33	25	32.66667	45	52	52	49.66667
SEN1507	53	20	30.66667	1.736	0.008746	1.515942	1.515942187	0.600214735	0.600215	0.041887	33	47	45	41.66667	86	65	66	72.33333
SEN1509	95	50	44.66667	1.465278	0.086333	1.293553	1.2935527	0.371338832	0.371339	0.228443	133	72	83	96	167	118	137	140.6667
SEN1510	153	63	47	1.111199	0.881085	-1.01934	1.019342971	0.027639546	-0.02764	0.962171	491	415	362	422.6667	478	416	515	469.6667
SEN1511	286	116	120	1.092426	0.761823	-1.04021	1.040209846	0.056874599	-0.05687	0.882712	1397	1281	1217	1298.333	1503	1441	1311	1418.333
SEN1512	378	267	245.6667	1.967192	0.000627	1.765457	1.765456837	0.82004155	0.820042	0.005272	391	213	158	254	536	483	480	499.6667
SEN1517	101	28	60.33333	1.780172	0.001727	1.571826	1.571826187	0.652441693	0.652442	0.011758	99	75	58	77.33333	151	103	159	137.6667
SEN1520	134	69	98.33333	1.996622	2.36E-06	1.753355	1.753355123	0.810118228	0.810118	5.56E-05	106	101	89	98.66667	223	170	198	197
SEN1521	36	15	19.33333	1.674419	0.041463	1.464546	1.464546033	0.55045354	0.550454	0.136901	20	40	26	28.66667	56	41	47	48
SEN1522	71	18	-41.3333	-1.3769	0.000173	-1.56477	1.564770273	0.645950868	-0.64595	0.001855	163	159	131	151	124	92	113	109.6667
SEN1523	26	9	12.66667	1.612903	0.057524	1.422725	1.422725493	0.508657329	0.508657	0.17269	28	22	12	20.66667	38	31	31	33.33333
SEN1524	130	97	92.66667	2.077519	2.13E-05	1.847999	1.847998549	0.885963624	0.885964	0.000347	124	74	60	86	190	175	171	178.6667
SEN1538	19	9	12	1.537313	0.089871	1.352477	1.352477318	0.4356044	0.435604	0.234108	27	20	20	22.33333	39	35	29	34.33333
SEN1543A	70	46	42.33333	1.607656	0.015252	1.423794	1.423794049	0.509740477	0.50974	0.063816	99	56	54	69.66667	124	102	110	112
SEN1556	69	17	33	1.502538	0.068041	1.340273	1.340273378	0.4225273	0.422527	0.195264	87	72	38	65.66667	89	100	107	98.66667

Feature ID	Experiment - Range (original values)	Experiment - IQR (original values)	Experiment - Difference (original values)	Experiment - Fold Change (original values)	EDGE test: yccT NT vs WT NT , tagwise dispersions - P-value	EDGE test: yccT NT vs WT NT , tagwise dispersions - Fold change	yccT NT vs WT NT ABS|FC|	yccT NT vs WT NT Log2FC	yccT NT vs WT NT Log2FC +-	EDGE test: yccT NT vs WT NT , tagwise dispersions - FDR p-value correction	WT NT - WT.1.S22 - Expression values	WT NT - WT.2.S23 - Expression values	WT NT - WT.3.S24 - Expression values	WT NT - Means	yccT NT - yccT.1.S28 - Expression values	yccT NT - yccT.2.S29 - Expression values	yccT NT - yccT.3.S30 - Expression values	yccT NT - Means
SEN1557	200	55	16	1.031873	0.480404	-1.10237	1.102373109	0.140612601	-0.14061	0.686186	448	629	429	502	503	484	567	518
SEN1558	547	179	-129.667	-1.06351	0.177623	-1.21089	1.210886771	0.276063966	-0.27606	0.365421	2115	2463	1936	2171.333	2170	1916	2039	2041.667
SEN1559	0	0	0	1	1	1	1	0	0	1	0	0	0	0	0	0	0	0
SEN1560	0	0	0	1	1	1	1	0	0	1	0	0	0	0	0	0	0	0
SEN1564A	43	28	19	1.208029	0.6166	1.069083	1.069083306	0.096374277	0.096374	0.78418	117	83	74	91.33333	115	111	105	110.3333
SEN1565	310	121	165.6667	1.293042	0.297656	1.137134	1.137134163	0.185402479	0.185402	0.513396	642	538	516	565.3333	826	659	708	731
SEN1568	209	54	118	1.374207	0.167597	1.198095	1.198095263	0.260742624	0.260743	0.352288	282	348	316	315.3333	491	370	439	433.3333
SEN1576	132	60	88.66667	2.385417	1.79E-06	2.115291	2.11529066	1.080855916	1.080856	4.34E-05	91	59	42	64	165	119	174	152.6667
SEN1606	86	47	30.66667	1.078767	0.658689	-1.05557	1.055566188	0.078017044	-0.07802	0.816174	384	431	353	389.3333	439	384	437	420
SEN1635	19	5	10.33333	2.033333	0.032016	1.773458	1.773457673	0.826564898	0.826565	0.112356	8	9	13	10	20	14	27	20.33333
SEN1677	422	25	-212.667	-1.56812	0.001879	-1.82596	1.825955791	0.868651836	-0.86865	0.012461	342	655	764	587	390	365	368	374.3333
SEN1679	193	63	88.66667	1.146556	0.971206	1.005142	1.00514176	0.007398986	0.007399	1	648	585	582	605	775	614	692	693.6667
SEN1713	117	69	79	2.044053	2.67E-05	1.803059	1.803058809	0.850446453	0.850446	0.000421	98	57	72	75.66667	149	141	174	154.6667
SEN1715	25	11	12	1.467532	0.146265	1.29788	1.297880011	0.376157012	0.376157	0.320382	36	25	16	25.66667	36	36	41	37.66667
SEN1719	5473	3436	2702.667	1.290308	0.422667	1.151613	1.151613191	0.203656219	0.203656	0.635075	12276	8814	6839	9309.667	12250	12312	11475	12012.33
SEN1726	254	136	-178.667	-1.53546	2.49E-05	-1.76354	1.763538356	0.818472954	-0.81847	0.000401	466	506	565	512.3333	360	311	330	333.6667
SEN1734	327	150	186.6667	1.580311	0.009719	1.39906	1.399059875	0.484457707	0.484458	0.045473	411	301	253	321.6667	580	451	494	508.3333
SEN1742	1083	405	447.6667	1.336086	0.26375	1.196329	1.196328912	0.258614091	0.258614	0.475513	1806	1381	809	1332	1892	1661	1786	1779.667
SEN1743	630	245	280.6667	1.359368	0.212416	1.21987	1.219869519	0.286726841	0.286727	0.413271	1061	816	466	781	1096	1093	996	1061.667
SEN1751	167	113	132.6667	1.65353	0.000856	1.455709	1.455709067	0.541722052	0.541722	0.006774	219	204	186	203	337	317	353	335.6667
SEN1752	225	157	172	1.499516	0.018669	1.322824	1.322823571	0.403620658	0.403621	0.074125	385	344	304	344.3333	529	501	519	516.3333
SEN1753	222	97	134.6667	1.535099	0.034915	1.350304	1.350304039	0.433284286	0.433284	0.120023	316	180	259	251.6667	401	356	402	386.3333
SEN1754	372	100	191.6667	1.560429	0.05141	1.367545	1.367545231	0.451588551	0.451589	0.159076	432	221	373	342	593	473	535	533.6667
SEN1755	386	172	222	1.635496	0.014521	1.437837	1.43783718	0.523900315	0.5239	0.061807	445	242	361	349.3333	628	533	553	571.3333
SEN1756	280	173	191	1.690361	0.005737	1.489669	1.48966873	0.574991542	0.574992	0.029838	353	196	281	276.6667	476	454	473	467.6667
SEN1757	272	188	175.6667	2.111814	0.001993	1.888459	1.888458543	0.917209113	0.917209	0.013063	265	78	131	158	350	332	319	333.6667
SEN1758	399	318	285	1.846535	0.001215	1.646468	1.646468076	0.719374539	0.719375	0.008929	491	250	269	336.6667	649	629	587	621.6667
SEN1764	144	19	57.33333	1.09712	0.739449	-1.04235	1.042348557	0.05983779	-0.05984	0.869098	600	575	596	590.3333	719	581	643	647.6667
SEN1765	85	36	53.66667	1.442308	0.043823	1.27335	1.273350167	0.34862921	0.348629	0.142418	133	126	105	121.3333	162	173	190	175
SEN1775	505	389	246.3333	1.296787	0.317643	1.156037	1.156037383	0.209188051	0.209188	0.532988	1151	693	646	830	1119	1082	1028	1076.333
SEN1778	10	3	4	1.210526	0.747425	1.067559	1.067559441	0.0943164	0.094316	0.876079	23	19	15	19	22	25	22	23
SEN1779	65	45	42	1.6	0.010515	1.418022	1.418021917	0.503879832	0.50388	0.047886	94	61	55	70	106	110	120	112
SEN1783	84	18	37	1.412639	0.156019	1.246053	1.246053147	0.317365604	0.317366	0.33372	107	66	96	89.66667	116	150	114	126.6667
SEN1784	12	3	2.666667	1.333333	0.618654	1.171883	1.171883025	0.22882857	0.228829	0.785235	10	10	4	8	16	7	9	10.66667
SEN1785	56	30	40.33333	2.052174	2.51E-05	1.808365	1.808364775	0.854685721	0.854686	0.000402	46	41	28	38.33333	84	71	81	78.66667
SEN1786	393	124	-89.3333	-1.06872	0.154714	-1.2075	1.207495265	0.272017531	-0.27202	0.332785	1522	1517	1129	1389.333	1340	1342	1218	1300

Feature ID	Experiment - Range (original values)	Experiment - IQR (original values)	Experiment - Difference (original values)	Experiment - Fold Change (original values)	EDGE test: yccT NT vs WT NT , tagwise dispersions - P-value	EDGE test: yccT NT vs WT NT , tagwise dispersions - Fold change	yccT NT vs WT NT ABS|FC|	yccT NT vs WT NT Log2FC	yccT NT vs WT NT Log2FC +-	EDGE test: yccT NT vs WT NT , tagwise dispersions - FDR p-value correction	WT NT - WT.1.S22 - Expression values	WT NT - WT.2.S23 - Expression values	WT NT - WT.3.S24 - Expression values	WT NT - Means	yccT NT - yccT.1.S28 - Expression values	yccT NT - yccT.2.S29 - Expression values	yccT NT - yccT.3.S30 - Expression values	yccT NT - Means
SEN1787	19	5	12	2.5	0.00305	2.185973	2.185973376	1.12827583	1.128276	0.018387	10	10	4	8	22	15	23	20
SEN1789	110	32	-66.3333	-1.76834	2.31E-06	-2.02885	2.028850933	1.020662869	-1.02066	5.48E-05	120	183	155	152.6667	98	73	88	86.33333
SEN1790	22	7	-5.66667	-1.06746	0.129369	-1.21039	1.210390595	0.275472683	-0.27547	0.296753	101	88	80	89.66667	87	86	79	84
SEN1791	78	19	-4	-1.02532	0.289385	-1.18351	1.183507616	0.24306899	-0.24307	0.505626	134	140	212	162	159	159	156	158
SEN1792	519	51	-250	-2.79856	4.41E-06	-3.30635	3.30635395	1.725241175	-1.72524	9.23E-05	182	346	639	389	131	120	166	139
SEN1793	859	51	-412	-2.7913	7.9E-06	-3.3055	3.305495242	1.724866438	-1.72487	0.000152	279	587	1060	642	228	201	261	230
SEN1794	406	31	-172.667	-2.09746	0.000407	-2.48586	2.48585937	1.313744683	-1.31374	0.003674	146	307	537	330	177	131	164	157.3333
SEN1795	1662	63	-689.667	-3.23434	3.8E-05	-3.86596	3.865961327	1.950827205	-1.95083	0.000555	344	720	1931	998.3333	269	297	360	308.6667
SEN1796	7172	99	-2974	-3.92909	2.87E-05	-4.71797	4.717973305	2.238167256	-2.23817	0.00045	1113	2782	8073	3989.333	901	1023	1122	1015.333
SEN1797	1011	455	-479.333	-1.18223	0.072862	-1.34451	1.344506979	0.427077243	-0.42708	0.204078	3267	2840	3222	3109.667	2590	3045	2256	2630.333
SEN1798	141	7	-4.33333	-1.01243	0.303849	-1.16718	1.167176958	0.223023307	-0.22302	0.519227	292	334	433	353	339	341	366	348.6667
SEN1799	130	79	97.33333	1.563707	0.00784	1.37331	1.373310038	0.457657365	0.457657	0.038345	192	154	172	172.6667	284	251	275	270
SEN1800	1096	622	592	1.800361	0.008294	1.626208	1.626207737	0.701511563	0.701512	0.040079	1152	563	504	739.6667	1185	1600	1210	1331.667
SEN1801	299	175	202	1.926606	0.000757	1.674141	1.674141145	0.743421165	0.743421	0.006109	223	160	271	218	459	398	403	420
SEN1802	2613	1997	1909.333	1.861872	0.003732	1.655525	1.655524691	0.727288528	0.727289	0.021393	3025	1686	1935	2215.333	4143	4299	3932	4124.667
SEN1805	176	94	107.3333	2.009404	0.000458	1.804287	1.804286738	0.85142863	0.851429	0.004093	158	102	59	106.3333	196	210	235	213.6667
SEN1809	9	2	1.666667	1.108696	1	-1.02445	1.024449215	0.034848467	-0.03485	1	13	20	13	15.33333	14	15	22	17
SEN1810	72	28	43.33333	1.31477	0.210609	1.156696	1.156695578	0.210009222	0.210009	0.411468	144	146	123	137.6667	195	172	176	181
SEN1813	44	25	24.33333	1.205634	0.679559	1.057255	1.057254836	0.08032316	0.080323	0.827568	106	136	113	118.3333	150	138	140	142.6667
SEN1821	1501	196	-647	-1.23697	0.04141	-1.42056	1.420559256	0.506459012	-0.50646	0.136828	2671	4045	3416	3377.333	2867	2544	2780	2730.333
SEN1828	298	96	-139.333	-1.07642	0.131588	-1.22732	1.227323922	0.295516063	-0.29552	0.299316	2026	1949	1913	1962.667	1919	1728	1823	1823.333
SEN1840	192	34	-89	-1.09478	0.080724	-1.24946	1.249459745	0.321304421	-0.3213	0.21877	993	1087	1004	1028	963	895	959	939
SEN1861	146	105	2	1.003358	0.328598	-1.14606	1.146062148	0.196685279	-0.19669	0.54248	510	628	649	595.6667	614	523	656	597.6667
SEN1890	14	9	10.33333	1.574074	0.105177	1.38278	1.382780093	0.467571739	0.467572	0.259344	19	18	17	18	31	27	27	28.33333
SEN1901	165	42	20.66667	1.034216	0.456471	-1.09565	1.095654551	0.131793003	-0.13179	0.665118	704	569	539	604	668	611	595	624.6667
SEN1914	78	45	25.66667	1.137011	0.957261	1.006905	1.006905032	0.00992762	0.009928	1	223	185	154	187.3333	229	232	178	213
SEN1914A	7	3	1.333333	1.2	0.886405	1.058567	1.058567299	0.082112992	0.082113	0.96479	11	5	4	6.666667	7	9	8	8
SEN1914B	40	24	13.66667	1.234286	0.56461	1.095145	1.09514512	0.131122057	0.131122	0.745911	83	49	43	58.33333	73	68	75	72
SEN1915	147	108	92.33333	1.585624	0.025773	1.413839	1.413839432	0.499618285	0.499618	0.095086	233	115	125	157.6667	258	262	230	250
SEN1916	27	14	18.66667	2.866667	3.74E-05	2.511735	2.511735223	1.328684389	1.328684	0.000551	12	12	6	10	26	27	33	28.66667
SEN1917	14	7	6.666667	1.666667	0.127378	1.470314	1.470314281	0.556124566	0.556125	0.293659	17	9	4	10	16	16	18	16.66667
SEN1918	21	3	7.666667	1.676471	0.135702	1.482037	1.482036881	0.56758135	0.567581	0.304762	14	14	6	11.33333	11	19	27	19
SEN1919	99	18	29	1.225974	0.548876	1.093347	1.093346728	0.128750989	0.128751	0.736321	171	137	77	128.3333	176	155	141	157.3333
SEN1919A	0	0	0	1	1	1	1	0	0	1	0	0	0	0	0	0	0	0
SEN1920	0	0	0	1	1	1	1	0	0	1	0	0	0	0	0	0	0	0
SEN1921	0	0	0	1	1	1	1	0	0	1	0	0	0	0	0	0	0	0

Feature ID	Experiment - Range (original values)	Experiment - IQR (original values)	Experiment - Difference (original values)	Experiment - Fold Change (original values)	EDGE test: yccT NT vs WT NT , tagwise dispersions - P-value	EDGE test: yccT NT vs WT NT , tagwise dispersions - Fold change	yccT NT vs WT NT ABS|FC|	yccT NT vs WT NT Log2FC	yccT NT vs WT NT Log2FC +-	EDGE test: yccT NT vs WT NT , tagwise dispersions - FDR p-value correction	WT NT - WT.1.S22 - Expression values	WT NT - WT.2.S23 - Expression values	WT NT - WT.3.S24 - Expression values	WT NT - Means	yccT NT - yccT.1.S28 - Expression values	yccT NT - yccT.2.S29 - Expression values	yccT NT - yccT.3.S30 - Expression values	yccT NT - Means
SEN1922	0	0	0	1	1	1	1	0	0	1	0	0	0	0	0	0	0	0
SEN1923	0	0	0	1	1	1	1	0	0	1	0	0	0	0	0	0	0	0
SEN1924	0	0	0	1	1	1	1	0	0	1	0	0	0	0	0	0	0	0
SEN1925	0	0	0	1	1	1	1	0	0	1	0	0	0	0	0	0	0	0
SEN1926	0	0	0	1	1	1	1	0	0	1	0	0	0	0	0	0	0	0
SEN1927	0	0	0	1	1	1	1	0	0	1	0	0	0	0	0	0	0	0
SEN1928	0	0	0	1	1	1	1	0	0	1	0	0	0	0	0	0	0	0
SEN1929	0	0	0	1	1	1	1	0	0	1	0	0	0	0	0	0	0	0
SEN1930	0	0	0	1	1	1	1	0	0	1	0	0	0	0	0	0	0	0
SEN1931	0	0	0	1	1	1	1	0	0	1	0	0	0	0	0	0	0	0
SEN1932	0	0	0	1	1	1	1	0	0	1	0	0	0	0	0	0	0	0
SEN1932A	0	0	0	1	1	1	1	0	0	1	0	0	0	0	0	0	0	0
SEN1933	0	0	0	1	1	1	1	0	0	1	0	0	0	0	0	0	0	0
SEN1934	0	0	0	1	1	1	1	0	0	1	0	0	0	0	0	0	0	0
SEN1935	0	0	0	1	1	1	1	0	0	1	0	0	0	0	0	0	0	0
SEN1935A	0	0	0	1	1	1	1	0	0	1	0	0	0	0	0	0	0	0
SEN1936	0	0	0	1	1	1	1	0	0	1	0	0	0	0	0	0	0	0
SEN1937	0	0	0	1	1	1	1	0	0	1	0	0	0	0	0	0	0	0
SEN1938	0	0	0	1	1	1	1	0	0	1	0	0	0	0	0	0	0	0
SEN1939	0	0	0	1	1	1	1	0	0	1	0	0	0	0	0	0	0	0
SEN1940	0	0	0	1	1	1	1	0	0	1	0	0	0	0	0	0	0	0
SEN1941	0	0	0	1	1	1	1	0	0	1	0	0	0	0	0	0	0	0
SEN1942	0	0	0	1	1	1	1	0	0	1	0	0	0	0	0	0	0	0
SEN1943	0	0	0	1	1	1	1	0	0	1	0	0	0	0	0	0	0	0
SEN1944	0	0	0	1	1	1	1	0	0	1	0	0	0	0	0	0	0	0
SEN1945	0	0	0	1	1	1	1	0	0	1	0	0	0	0	0	0	0	0
SEN1946	0	0	0	1	1	1	1	0	0	1	0	0	0	0	0	0	0	0
SEN1947	0	0	0	1	1	1	1	0	0	1	0	0	0	0	0	0	0	0
SEN1948	0	0	0	1	1	1	1	0	0	1	0	0	0	0	0	0	0	0
SEN1949	0	0	0	1	1	1	1	0	0	1	0	0	0	0	0	0	0	0
SEN1949A	0	0	0	1	1	1	1	0	0	1	0	0	0	0	0	0	0	0
SEN1950	0	0	0	1	1	1	1	0	0	1	0	0	0	0	0	0	0	0
SEN1951	0	0	0	1	1	1	1	0	0	1	0	0	0	0	0	0	0	0
SEN1952	0	0	0	1	1	1	1	0	0	1	0	0	0	0	0	0	0	0
SEN1953	0	0	0	1	1	1	1	0	0	1	0	0	0	0	0	0	0	0
SEN1954	0	0	0	1	1	1	1	0	0	1	0	0	0	0	0	0	0	0

Feature ID	Experiment - Range (original values)	Experiment - IQR (original values)	Experiment - Difference (original values)	Experiment - Fold Change (original values)	EDGE test: yccT NT vs WT NT , tagwise dispersions - P-value	EDGE test: yccT NT vs WT NT , tagwise dispersions - Fold change	yccT NT vs WT NT ABS|FC|	yccT NT vs WT NT Log2FC	yccT NT vs WT NT Log2FC +-	EDGE test: yccT NT vs WT NT , tagwise dispersions - FDR p-value correction	WT NT - WT.1.S22 - Expression values	WT NT - WT.2.S23 - Expression values	WT NT - WT.3.S24 - Expression values	WT NT - Means	yccT NT - yccT.1.S28 - Expression values	yccT NT - yccT.2.S29 - Expression values	yccT NT - yccT.3.S30 - Expression values	yccT NT - Means
SEN1955	0	0	0	1	1	1	1	0	0	1	0	0	0	0	0	0	0	0
SEN1957	0	0	0	1	1	1	1	0	0	1	0	0	0	0	0	0	0	0
SEN1957A	0	0	0	1	1	1	1	0	0	1	0	0	0	0	0	0	0	0
SEN1959	0	0	0	1	1	1	1	0	0	1	0	0	0	0	0	0	0	0
SEN1960	0	0	0	1	1	1	1	0	0	1	0	0	0	0	0	0	0	0
SEN1961	0	0	0	1	1	1	1	0	0	1	0	0	0	0	0	0	0	0
SEN1962	0	0	0	1	1	1	1	0	0	1	0	0	0	0	0	0	0	0
SEN1963	0	0	0	1	1	1	1	0	0	1	0	0	0	0	0	0	0	0
SEN1964	0	0	0	1	1	1	1	0	0	1	0	0	0	0	0	0	0	0
SEN1965	0	0	0	1	1	1	1	0	0	1	0	0	0	0	0	0	0	0
SEN1965A	0	0	0	1	1	1	1	0	0	1	0	0	0	0	0	0	0	0
SEN1966	0	0	0	1	1	1	1	0	0	1	0	0	0	0	0	0	0	0
SEN1970	550	258	326.6667	1.425717	0.068358	1.262982	1.262981563	0.336833578	0.336834	0.195795	931	759	612	767.3333	1162	1017	1103	1094
SEN1971	1157	314	477	1.18679	0.797408	1.042652	1.04265182	0.06025747	0.060257	0.905746	2941	2093	2627	2553.667	3250	3120	2722	3030.667
SEN1974	19	3	10.33333	1.442857	0.205129	1.270577	1.270576565	0.345483315	0.345483	0.403789	25	27	18	23.33333	37	36	28	33.66667
SEN1975	100	42	-31.3333	-1.07599	0.130571	-1.22525	1.225253434	0.293080191	-0.29308	0.298073	424	460	447	443.6667	405	466	366	412.3333
SEN1976	10	1	3	1.642857	0.357537	1.440802	1.440801553	0.526871641	0.526872	0.572758	5	5	4	4.666667	2	12	9	7.666667
SEN1977	21	5	10	1.20979	0.683977	1.064138	1.064137783	0.08968496	0.089685	0.83124	48	53	42	47.66667	60	50	63	57.66667
SEN1978	6	1	2.666667	3	0.127338	2.510257	2.51025744	1.327835328	1.327835	0.293659	1	1	2	1.333333	3	7	2	4
SEN1979	5	1	0	-1	1	-1.12982	1.12982409	0.176098167	-0.1761	1	2	3	4	3	1	6	2	3
SEN1980	43	28	33.66667	1.515306	0.02208	1.33435	1.334349742	0.416136857	0.416137	0.083981	68	68	60	65.33333	98	103	96	99
SEN1981	34	14	10	1.097403	0.7697	-1.03723	1.037228105	0.052733203	-0.05273	0.887872	110	96	102	102.6667	128	116	94	112.6667
SEN1981A	10	2	0.666667	1.057143	0.914999	-1.07002	1.070016182	0.097632615	-0.09763	0.980973	18	8	9	11.66667	10	11	16	12.33333
SEN1982	10	1	-1.33333	-1.06557	0.377339	-1.20643	1.206428057	0.270741886	-0.27074	0.594928	27	21	17	21.66667	20	21	20	20.33333
SEN1983	11	3	-0.33333	-1.04167	0.606464	-1.18801	1.188009718	0.248546637	-0.24855	0.777334	8	6	11	8.333333	15	5	4	8
SEN1984	7	3	3.666667	1.733333	0.290153	1.515348	1.51534771	0.599648871	0.599649	0.506767	8	2	5	5	9	9	8	8.666667
SEN1985	274	31	-157.333	-1.46918	0.00074	-1.69135	1.691351337	0.758176375	-0.75818	0.006017	381	528	569	492.6667	350	361	295	335.3333
SEN1986	130	52	85.33333	1.311436	0.217291	1.156461	1.156460613	0.209716131	0.209716	0.420627	295	284	243	274	373	369	336	359.3333
SEN1987	6	2	-0.33333	-1.04348	0.782167	-1.17955	1.179550038	0.238236621	-0.23824	0.89555	11	8	5	8	6	7	10	7.666667
SEN1988	5	3	2.333333	3.333333	0.098911	2.738018	2.738018039	1.453131952	1.453132	0.249019	3	0	0	1	3	5	2	3.333333
SEN1989	3	1	0.333333	1.25	1	1.094393	1.094393382	0.130131411	0.130131	1	3	1	0	1.333333	1	2	2	1.666667
SEN1990	28	4	-0.33333	-1.00592	0.389533	-1.14197	1.141966389	0.191520189	-0.19152	0.606472	52	73	45	56.66667	56	52	61	56.33333
SEN1991	20	4	-6.33333	-1.15079	0.113266	-1.31468	1.314684879	0.394717037	-0.39472	0.272466	44	41	60	48.33333	40	46	40	42
SEN1992	7	3	2.666667	1.444444	0.474285	1.267789	1.267788515	0.342314104	0.342314	0.680115	5	9	4	6	8	11	7	8.666667
SEN1993	72	14	25.66667	1.088202	0.700777	-1.05208	1.052082218	0.073247453	-0.07325	0.842873	249	321	303	291	320	313	317	316.6667
SEN1994	250	63	-165	-1.60961	0.000103	-1.84813	1.848127311	0.886064143	-0.88606	0.001206	330	490	487	435.6667	267	305	240	270.6667

Feature ID	Experiment - Range (original values)	Experiment - IQR (original values)	Experiment - Difference (original values)	Experiment - Fold Change (original values)	EDGE test: yccT NT vs WT NT , tagwise dispersions - P-value	EDGE test: yccT NT vs WT NT , tagwise dispersions - Fold change	yccT NT vs WT NT ABS|FC|	yccT NT vs WT NT Log2FC	yccT NT vs WT NT Log2FC +-	EDGE test: yccT NT vs WT NT , tagwise dispersions - FDR p-value correction	WT NT - WT.1.S22 - Expression values	WT NT - WT.2.S23 - Expression values	WT NT - WT.3.S24 - Expression values	WT NT - Means	yccT NT - yccT.1.S28 - Expression values	yccT NT - yccT.2.S29 - Expression values	yccT NT - yccT.3.S30 - Expression values	yccT NT - Means
SEN1995	1273	318	-870	-4.22621	9.56E-13	-4.88997	4.889966206	2.289824495	-2.28982	9.01E-11	583	1521	1315	1139.667	265	296	248	269.6667
SEN1996	4	0	1	1.1875	1	1.043514	1.04351384	0.061449735	0.06145	1	5	6	5	5.333333	9	5	5	6.333333
SEN1997	3	0	-1.66667	-1.625	0.20065	-1.80739	1.807394036	0.853911067	-0.85391	0.397976	3	5	5	4.333333	3	3	2	2.666667
SEN1998	8	2	3.666667	2.222222	0.151671	1.921892	1.921891927	0.942527212	0.942527	0.328152	5	0	4	3	6	8	6	6.666667
SEN1999	435	259	190	1.285	0.335778	1.148601	1.148600765	0.199877427	0.199877	0.549828	907	621	472	666.6667	880	897	793	856.6667
SEN2001	17	10	9	1.692308	0.080352	1.487847	1.487846702	0.573225889	0.573226	0.218296	20	9	10	13	26	17	23	22
SEN2002	4	0	2	2.2	0.311577	1.874419	1.874419233	0.906443663	0.906444	0.526544	2	1	2	1.666667	4	2	5	3.666667
SEN2003	5	3	2.666667	2	0.322516	1.725773	1.725772665	0.787242432	0.787242	0.53785	2	1	5	2.666667	6	5	5	5.333333
SEN2004	11	0	6	1.128571	1	-1.00869	1.008692163	0.012485954	-0.01249	1	47	46	47	46.66667	57	47	54	52.66667
SEN2005	23	3	11	1.168367	0.855782	1.027459	1.02745917	0.039081064	0.039081	0.947992	66	69	61	65.33333	76	69	84	76.33333
SEN2006	50	37	21.66667	1.338542	0.297078	1.189062	1.189061528	0.249823369	0.249823	0.512976	97	47	48	64	94	78	85	85.66667
SEN2006A	108	12	1	1.005263	0.407556	-1.12317	1.12316865	0.167574573	-0.16757	0.621646	198	240	132	190	200	186	187	191
SEN2008	142	22	59.33333	1.155323	0.876247	1.020128	1.020128233	0.028750515	0.028751	0.959152	396	412	338	382	426	480	418	441.3333
SEN2008A	139	63	80.66667	1.200997	0.649365	1.058415	1.058415056	0.081905489	0.081905	0.807754	403	440	361	401.3333	466	500	480	482
SEN2122	357	119	167.3333	1.621287	0.020238	1.44858	1.448580408	0.534639768	0.53464	0.0788	381	268	159	269.3333	516	387	407	436.6667
SEN2128	50	34	35.33333	2.152174	5.85E-05	1.89602	1.896020448	0.922974523	0.922975	0.000776	43	24	25	30.66667	74	59	65	66
SEN2129	16	7	10.66667	1.542373	0.123114	1.354588	1.354588472	0.437854623	0.437855	0.287266	22	16	21	19.66667	32	28	31	30.33333
SEN2130	98	29	57.33333	1.508876	0.024543	1.32174	1.321739799	0.402438192	0.402438	0.091387	112	122	104	112.6667	202	141	167	170
SEN2133	28	10	14	1.442105	0.150525	1.266746	1.266746056	0.341127337	0.341127	0.326308	36	29	30	31.66667	57	40	40	45.66667
SEN2134	48	23	37	2.09901	0.000207	1.845342	1.845342201	0.883888375	0.883888	0.002087	34	30	37	33.66667	57	77	78	70.66667
SEN2135	2	0	1	1.6	0.777941	1.382156	1.382156099	0.466920561	0.466921	0.892789	2	2	1	1.666667	3	2	3	2.666667
SEN2144	276	130	192.3333	1.316164	0.23469	1.159478	1.159477766	0.213475156	0.213475	0.442703	652	604	569	608.3333	823	845	734	800.6667
SEN2150	101	48	66	1.611111	0.004222	1.428274	1.428273924	0.514272696	0.514273	0.023503	127	114	83	108	162	184	176	174
SEN2155	10	3	-1.66667	-1.16667	0.360839	-1.32037	1.320368513	0.40094064	-0.40094	0.577421	14	12	9	11.66667	10	5	15	10
SEN2168	292	126	142	1.380357	0.124147	1.223135	1.223134937	0.290583572	0.290584	0.288461	478	352	290	373.3333	582	462	502	515.3333
SEN2169	106	14	37.33333	1.286445	0.3488	1.137175	1.137175148	0.185454475	0.185454	0.564062	152	149	90	130.3333	196	138	169	167.6667
SEN2170	113	44	45.66667	1.292735	0.293409	1.14805	1.148049545	0.199184904	0.199185	0.510042	204	152	112	156	225	196	184	201.6667
SEN2171	146	58	55.66667	1.41133	0.186444	1.259964	1.259964287	0.333382842	0.333383	0.376028	195	137	74	135.3333	220	143	210	191
SEN2172	29	4	13	1.453488	0.162626	1.281657	1.281657383	0.358010648	0.358011	0.344784	32	36	18	28.66667	44	34	47	41.66667
SEN2173	60	9	18.66667	1.229508	0.559763	1.085488	1.085488363	0.118344259	0.118344	0.744167	97	88	59	81.33333	119	86	95	100
SEN2179	517	294	262	1.218333	0.605257	1.074352	1.074351521	0.103466111	0.103466	0.777315	1157	1466	977	1200	1494	1441	1451	1462
SEN2188	35	12	-11.3333	-1.04468	0.129459	-1.18837	1.188371312	0.248985684	-0.24899	0.296753	276	268	251	265	257	241	263	253.6667
SEN2189	107	39	59.33333	1.340344	0.155278	1.186659	1.186658731	0.246905093	0.246905	0.33296	204	184	135	174.3333	236	223	242	233.6667
SEN2190	22	11	13.66667	2.366667	0.002055	2.079747	2.079747234	1.056408199	1.056408	0.013368	14	9	7	10	20	29	22	23.66667
SEN2191	23	10	13	1.322314	0.331988	1.162336	1.162335525	0.217026584	0.217027	0.545839	38	48	35	40.33333	58	48	54	53.33333
SEN2201	18	9	12	2.241379	0.004836	1.963921	1.963921046	0.973736931	0.973737	0.026102	12	11	6	9.666667	24	20	21	21.66667

Feature ID	Experiment - Range (original values)	Experiment - IQR (original values)	Experiment - Difference (original values)	Experiment - Fold Change (original values)	EDGE test: yccT NT vs WT NT , tagwise dispersions - P-value	EDGE test: yccT NT vs WT NT , tagwise dispersions - Fold change	yccT NT vs WT NT ABS|FC|	yccT NT vs WT NT Log2FC	yccT NT vs WT NT Log2FC +-	EDGE test: yccT NT vs WT NT , tagwise dispersions - FDR p-value correction	WT NT - WT.1.S22 - Expression values	WT NT - WT.2.S23 - Expression values	WT NT - WT.3.S24 - Expression values	WT NT - Means	yccT NT - yccT.1.S28 - Expression values	yccT NT - yccT.2.S29 - Expression values	yccT NT - yccT.3.S30 - Expression values	yccT NT - Means
SEN2202	2	0	0.666667	#DIV/0!	0.501525	6.018176	6.018176136	2.589326331	2.589326	0.70377	0	0	0	0	0	2	0	0.666667
SEN2203	53	40	31.66667	1.306452	0.253863	1.157523	1.157522641	0.211040413	0.21104	0.46559	133	94	83	103.3333	134	135	136	135
SEN2204	65	56	12.66667	1.028107	0.394458	-1.11492	1.114916701	0.156935926	-0.15694	0.610706	427	441	484	450.6667	483	421	486	463.3333
SEN2218	194	146	173.6667	1.254022	0.446487	1.099361	1.099360993	0.136665196	0.136665	0.656559	685	683	683	683.6667	866	829	877	857.3333
SEN2227	29	17	18	1.981818	0.004291	1.745272	1.745271895	0.803451811	0.803452	0.023649	27	13	15	18.33333	35	32	42	36.33333
SEN2228	12	6	6.666667	1.666667	0.191882	1.456378	1.456377502	0.54238436	0.542384	0.384545	8	8	14	10	20	18	12	16.66667
SEN2232	0	0	0	1	1	1	1	0	0	1	0	0	0	0	0	0	0	0
SEN2234	0	0	0	1	1	1	1	0	0	1	0	0	0	0	0	0	0	0
SEN2235	0	0	0	1	1	1	1	0	0	1	0	0	0	0	0	0	0	0
SEN2236	0	0	0	1	1	1	1	0	0	1	0	0	0	0	0	0	0	0
SEN2237	1381	219	-971	-16.5775	1.14E-17	-19.5023	19.50227469	4.2855705	-4.28557	1.8E-15	282	1384	1434	1033.333	63	53	71	62.33333
SEN2255	59	8	31.33333	1.394958	0.178213	1.226993	1.226993246	0.295127308	0.295127	0.366023	90	60	88	79.33333	96	117	119	110.6667
SEN2256	5	2	2	1.193548	0.909305	1.04916	1.049160334	0.069235169	0.069235	0.976995	9	12	10	10.33333	14	11	12	12.33333
SEN2257	88	67	57.33333	1.632353	0.010302	1.454086	1.454085554	0.540112156	0.540112	0.047207	130	77	65	90.66667	147	153	144	148
SEN2262	40	20	25.33333	1.299213	0.305866	1.140373	1.140373046	0.189505845	0.189506	0.521303	83	89	82	84.66667	122	105	103	110
SEN2263	120	54	-82.3333	-1.19887	0.012602	-1.36771	1.367711586	0.451764037	-0.45176	0.055067	476	517	496	496.3333	423	422	397	414
SEN2269	141	15	12	1.025899	0.462842	-1.1102	1.110197253	0.150816028	-0.15082	0.670871	401	535	454	463.3333	445	542	439	475.3333
SEN2270	31	17	0	-1	0.381213	-1.14339	1.143385101	0.193311396	-0.19331	0.597719	87	72	103	87.33333	93	93	76	87.33333
SEN2271	71	25	23.66667	1.056937	0.534114	-1.08016	1.08015811	0.111242505	-0.11124	0.725969	403	440	404	415.6667	474	415	429	439.3333
SEN2275	289	157	-19	-1.01134	0.288653	-1.14845	1.148454483	0.199693679	-0.19969	0.505144	1817	1740	1528	1695	1788	1657	1583	1676
SEN2282	298	12	-153.667	-1.18008	0.01821	-1.33775	1.337747981	0.419806351	-0.41981	0.07277	1104	1042	875	1007	871	806	883	853.3333
SEN2284	76	17	-37.3333	-1.35	0.000997	-1.53351	1.533513363	0.616840738	-0.61684	0.007588	157	159	116	144	127	83	110	106.6667
SEN2285	173	126	81	1.131779	0.98796	-1.00238	1.002381508	0.003431705	-0.00343	1	714	568	562	614.6667	735	658	694	695.6667
SEN2296	6635	1370	1279.667	1.100933	0.91863	-1.01883	1.018832076	0.026916286	-0.02692	0.982488	14890	14890	8255	12678.33	14653	13938	13283	13958
SEN2311	18	9	7.666667	1.348485	0.419208	1.18573	1.185729799	0.245775289	0.245775	0.632022	29	17	20	22	34	20	35	29.66667
SEN2314	102	58	40.33333	1.078014	0.675509	-1.0526	1.052596654	0.073952714	-0.07395	0.825117	577	499	475	517	571	544	557	557.3333
SEN2318	701	541	426.3333	1.574831	0.030635	1.408959	1.408959435	0.494630076	0.49463	0.109032	1102	588	535	741.6667	1236	1139	1129	1168
SEN2322	13580	132	-5387	-9.22443	7.44E-07	-10.2864	10.28642515	3.362669783	-3.36267	2.11E-05	3151	14159	816	6042	684	702	579	655
SEN2323	12772	186	-5137.33	-12.747	2.48E-06	-14.2373	14.23726426	3.831600048	-3.8316	5.75E-05	2923	13181	620	5574.667	469	409	434	437.3333
SEN2324	17908	176	-7165	-15.1229	2.18E-06	-16.8632	16.86322139	4.075808256	-4.07581	5.19E-05	3950	18390	677	7672.333	539	501	482	507.3333
SEN2325	4311	68	-1729	-17.625	4.27E-07	-19.6336	19.6336311	4.295255108	-4.29526	1.3E-05	913	4413	173	1833	105	102	105	104
SEN2326	1907	11	-754.333	-17.1643	1.38E-06	-19.1702	19.17023156	4.260795858	-4.2608	3.48E-05	399	1945	59	801	54	38	48	46.66667
SEN2327	660	72	-63	-1.0467	0.244621	-1.17949	1.179494665	0.238168893	-0.23817	0.455235	1548	1674	1014	1412	1376	1304	1367	1349
SEN2328	460	237	299.3333	1.297253	0.273908	1.143795	1.143794992	0.193828494	0.193828	0.487228	1114	1014	893	1007	1353	1251	1315	1306.333
SEN2339	252	93	146.3333	1.30807	0.246639	1.153599	1.153598902	0.206141696	0.206142	0.45756	518	489	418	475	612	582	670	621.3333
SEN2340	115	83	90	1.830769	7.31E-05	1.613141	1.6131406	0.689872188	0.689872	0.000928	126	100	99	108.3333	214	198	183	198.3333

Feature ID	Experiment - Range (original values)	Experiment - IQR (original values)	Experiment - Difference (original values)	Experiment - Fold Change (original values)	EDGE test: yccT NT vs WT NT , tagwise dispersions - P-value	EDGE test: yccT NT vs WT NT , tagwise dispersions - Fold change	yccT NT vs WT NT ABS|FC|	yccT NT vs WT NT Log2FC	yccT NT vs WT NT Log2FC +-	EDGE test: yccT NT vs WT NT , tagwise dispersions - FDR p-value correction	WT NT - WT.1.S22 - Expression values	WT NT - WT.2.S23 - Expression values	WT NT - WT.3.S24 - Expression values	WT NT - Means	yccT NT - yccT.1.S28 - Expression values	yccT NT - yccT.2.S29 - Expression values	yccT NT - yccT.3.S30 - Expression values	yccT NT - Means
SEN2341	100	55	68.33333	1.931818	2.18E-05	1.705013	1.705013122	0.769782843	0.769783	0.000354	88	76	56	73.33333	156	131	138	141.6667
SEN2342	75	14	37.66667	1.801418	0.005342	1.597015	1.597014775	0.67537766	0.675378	0.028211	61	52	28	47	103	85	66	84.66667
SEN2343	762	145	-353.333	-1.75071	6.2E-05	-1.99556	1.99555714	0.996791589	-0.99679	0.000808	615	1206	651	824	498	444	470	470.6667
SEN2354	396	188	277.6667	1.746416	0.000563	1.5434	1.543400257	0.626112251	0.626112	0.004811	448	367	301	372	697	555	697	649.6667
SEN2355	31	7	17.33333	1.825397	0.018352	1.609013	1.609013197	0.686176159	0.686176	0.073193	28	12	23	21	30	43	42	38.33333
SEN2356	9	2	3.333333	1.4	0.54496	1.228424	1.228424064	0.296808679	0.296809	0.733458	10	7	8	8.333333	16	10	9	11.66667
SEN2357	13	5	7	2.05	0.039731	1.796485	1.796485304	0.845177133	0.845177	0.132467	10	6	4	6.666667	13	17	11	13.66667
SEN2358	26	5	8.333333	1.223214	0.744412	1.073512	1.073512058	0.102338396	0.102338	0.873541	45	24	43	37.33333	47	40	50	45.66667
SEN2359	92	9	-39.3333	-1.53636	0.002024	-1.77922	1.779223617	0.831247843	-0.83125	0.013207	85	98	155	112.6667	81	76	63	73.33333
SEN2361	350	167	-231.333	-1.21426	0.010613	-1.3898	1.389803614	0.474881037	-0.47488	0.048132	1266	1320	1347	1311	1143	997	1099	1079.667
SEN2380	10	3	6	1.857143	0.117494	1.626857	1.626856834	0.702087297	0.702087	0.278805	6	9	6	7	9	16	14	13
SEN2386	409	273	267.6667	1.816887	0.001126	1.625661	1.625661451	0.701026843	0.701027	0.008417	462	308	213	327.6667	622	583	581	595.3333
SEN2390	206	64	74.33333	1.23061	0.503179	1.093043	1.093043093	0.128350281	0.12835	0.704228	404	336	227	322.3333	433	357	400	396.6667
SEN2391	234	96	97	1.1769	0.754093	1.039813	1.039813243	0.056324434	0.056324	0.880075	654	522	469	548.3333	703	615	618	645.3333
SEN2392	417	166	188.6667	1.216526	0.556699	1.079258	1.07925805	0.110039854	0.11004	0.741429	1052	886	676	871.3333	1088	999	1093	1060
SEN2407	87	46	51	1.254153	0.379214	1.106969	1.106968925	0.146614724	0.146615	0.595639	229	196	177	200.6667	249	242	264	251.6667
SEN2415	48	21	20	1.3125	0.336401	1.15442	1.154420222	0.207168477	0.207168	0.550237	81	51	60	64	99	69	84	84
SEN2420	172	60	93.33333	1.339394	0.167867	1.177408	1.177407964	0.235614292	0.235614	0.352687	301	276	248	275	420	336	349	368.3333
SEN2427	802	482	-476	-1.10426	0.141737	-1.26202	1.262019811	0.335734558	-0.33573	0.31408	4970	5121	5034	5041.667	4890	4319	4488	4565.667
SEN2428	446	74	-204.667	-1.44949	0.00373	-1.68751	1.687505876	0.754892526	-0.75489	0.021393	430	729	821	660	504	375	487	455.3333
SEN2430	84	31	-16.3333	-1.0349	0.176287	-1.18457	1.184573293	0.244367465	-0.24437	0.364166	460	502	491	484.3333	504	420	480	468
SEN2433	26	15	16.33333	1.401639	0.200175	1.231483	1.231483188	0.300396932	0.300397	0.3973	34	51	37	40.66667	59	60	52	57
SEN2454	93	64	73	1.279693	0.305044	1.126016	1.126015959	0.171227274	0.171227	0.520667	274	265	244	261	337	329	336	334
SEN2457	505	455	426.6667	2.028939	6.76E-06	1.796308	1.796307637	0.845034449	0.845034	0.000133	518	356	370	414.6667	825	838	861	841.3333
SEN2463	98	13	-14.6667	-1.03123	0.184321	-1.17876	1.178755404	0.237264384	-0.23726	0.373404	465	517	471	484.3333	512	419	478	469.6667
SEN2465	425	56	67	1.064054	0.644821	-1.06152	1.061520189	0.086131811	-0.08613	0.804454	1267	1029	842	1046	1216	1038	1085	1113
SEN2471	391	205	196.6667	1.219005	0.555368	1.077306	1.077306205	0.107428369	0.107428	0.740992	1063	856	775	898	1166	1061	1057	1094.667
SEN2472	34	7	4	1.064171	0.687701	-1.065	1.065003365	0.090857989	-0.09086	0.833936	75	65	47	62.33333	81	58	60	66.33333
SEN2473	164	104	80.33333	1.307791	0.251609	1.161062	1.161062084	0.215445118	0.215445	0.462913	340	236	207	261	371	347	306	341.3333
SEN2475	1012	562	506	1.240723	0.523198	1.099423	1.099423404	0.136747096	0.136747	0.718594	2573	2011	1722	2102	2734	2480	2610	2608
SEN2484	761	463	567.3333	1.72704	0.00183	1.517453	1.517452796	0.601651638	0.601652	0.012248	887	659	795	780.3333	1420	1365	1258	1347.667
SEN2485	882	542	625.3333	1.651389	0.002674	1.458856	1.458855959	0.544837445	0.544837	0.016457	1135	928	817	960	1699	1470	1587	1585.333
SEN2488	202	113	144.3333	2.3003	3.66E-08	2.037484	2.037483949	1.026788694	1.026789	1.5E-06	141	114	78	111	280	227	259	255.3333
SEN2489	113	74	76.33333	1.812057	0.000446	1.610135	1.610134645	0.687181337	0.687181	0.003996	124	83	75	94	157	188	166	170.3333
SEN2507	13	1	5.333333	1.108844	0.85375	-1.02653	1.026532129	0.037778782	-0.03778	0.946945	50	48	49	49	61	50	52	54.33333
SEN2508	20	7	-9.66667	-1.40278	0.013093	-1.58781	1.587811269	0.66703944	-0.66704	0.056766	36	33	32	33.66667	16	25	31	24

Feature ID	Experiment - Range (original values)	Experiment - IQR (original values)	Experiment - Difference (original values)	Experiment - Fold Change (original values)	EDGE test: yccT NT vs WT NT , tagwise dispersions - P-value	EDGE test: yccT NT vs WT NT , tagwise dispersions - Fold change	yccT NT vs WT NT ABS|FC|	yccT NT vs WT NT Log2FC	yccT NT vs WT NT Log2FC +-	EDGE test: yccT NT vs WT NT , tagwise dispersions - FDR p-value correction	WT NT - WT.1.S22 - Expression values	WT NT - WT.2.S23 - Expression values	WT NT - WT.3.S24 - Expression values	WT NT - Means	yccT NT - yccT.1.S28 - Expression values	yccT NT - yccT.2.S29 - Expression values	yccT NT - yccT.3.S30 - Expression values	yccT NT - Means
SEN2509	15	3	7	1.141892	1	1.004027	1.004027344	0.00579856	0.005799	1	49	51	48	49.33333	62	60	47	56.33333
SEN2510	269	65	1.333333	1.001347	0.331505	-1.13627	1.136271178	0.184307184	-0.18431	0.545487	932	1153	884	989.6667	997	964	1012	991
SEN2512	1637	616	509	1.117979	0.945864	-1.01085	1.01085367	0.01557417	-0.01557	0.998362	5228	4124	3591	4314.333	5204	4526	4740	4823.333
SEN2514	70	36	48	1.447205	0.037391	1.275679	1.275679442	0.351265848	0.351266	0.126588	119	110	93	107.3333	157	146	163	155.3333
SEN2525	907	294	350	1.12454	0.965847	-1.00661	1.006611842	0.009507476	-0.00951	1	3171	2877	2383	2810.333	3227	2964	3290	3160.333
SEN2527	263	182	163	1.657258	0.013425	1.488488	1.48848795	0.573847542	0.573848	0.057828	372	219	153	248	416	416	401	411
SEN2531	98	49	50.33333	1.250415	0.445003	1.102442	1.102441687	0.140702347	0.140702	0.655958	240	168	195	201	244	244	266	251.3333
SEN2532	72	53	46	1.242958	0.437012	1.097921	1.097920666	0.134773811	0.134774	0.64806	225	174	169	189.3333	241	238	227	235.3333
SEN2550	380	103	-36.6667	-1.05705	0.218549	-1.18686	1.186860343	0.247150184	-0.24715	0.421958	886	646	506	679.3333	697	667	564	642.6667
SEN2551	218	67	-30.6667	-1.0725	0.152222	-1.20838	1.208379532	0.273073652	-0.27307	0.328701	545	489	327	453.6667	465	398	406	423
SEN2553	49	30	35.66667	1.652439	0.003773	1.458079	1.458079119	0.544069006	0.544069	0.02157	64	56	44	54.66667	93	92	86	90.33333
SEN2554	19	7	10	1.555556	0.117927	1.37282	1.372819893	0.457142363	0.457142	0.27943	25	19	10	18	29	26	29	28
SEN2555	85	64	66	1.530831	0.012989	1.350139	1.350139337	0.433108304	0.433108	0.056491	144	111	118	124.3333	182	193	196	190.3333
SEN2560	333	192	244.6667	1.354932	0.212601	1.181059	1.181058754	0.240080737	0.240081	0.413354	637	683	748	689.3333	970	875	957	934
SEN2582	20	1	9.333333	1.571429	0.123298	1.387482	1.387482392	0.472469463	0.472469	0.287393	20	19	10	16.33333	20	27	30	25.66667
SEN2589	157	128	89	1.355053	0.168269	1.202355	1.202354672	0.265862527	0.265863	0.353198	336	208	208	250.6667	365	338	316	339.6667
SEN2601	117	38	75.66667	1.165211	0.860787	1.023325	1.02332451	0.033263716	0.033264	0.950937	447	466	461	458	499	564	538	533.6667
SEN2609	253	68	127.6667	1.179559	0.747325	1.039979	1.039979052	0.056554468	0.056554	0.876079	789	721	623	711	876	785	855	838.6667
SEN2610	227	106	128	1.336252	0.178262	1.181475	1.181474605	0.24058862	0.240589	0.366023	465	359	318	380.6667	545	464	517	508.6667
SEN2611	22	3	9.333333	1.152174	1	1.009972	1.009972076	0.014315406	0.014315	1	57	65	62	61.33333	79	62	71	70.66667
SEN2612	64	42	17.66667	1.128329	1	1.000583	1.000583381	0.000841396	0.000841	1	180	117	116	137.6667	159	165	142	155.3333
SEN2613	44	16	22	1.573913	0.050228	1.386814	1.386814158	0.47177447	0.471774	0.156843	47	32	36	38.33333	53	52	76	60.33333
SEN2614	1922	1198	1306	1.683412	0.010497	1.496107	1.496106752	0.58121312	0.581213	0.047853	2475	1785	1473	1911	3395	2983	3273	3217
SEN2616	130	76	96.33333	1.39589	0.103666	1.224736	1.224735923	0.29247071	0.292471	0.25748	256	218	256	243.3333	339	348	332	339.6667
SEN2624	195	151	150.3333	2.193122	1.75E-06	1.947712	1.947712296	0.961780587	0.961781	4.27E-05	179	102	97	126	292	253	284	276.3333
SEN2630	193	121	125.3333	2.212903	1.15E-05	1.973103	1.973102568	0.980465953	0.980466	0.000203	151	86	73	103.3333	213	207	266	228.6667
SEN2631	62	17	34	2.073684	0.000817	1.837593	1.837593211	0.877817432	0.877817	0.006524	39	40	16	31.66667	63	56	78	65.66667
SEN2632	343	78	156.6667	1.256971	0.400519	1.115164	1.115163784	0.157255614	0.157256	0.615787	725	647	457	609.6667	790	709	800	766.3333
SEN2633	67	21	37.33333	2.154639	0.000195	1.904956	1.90495648	0.929758039	0.929758	0.002008	38	37	22	32.33333	58	62	89	69.66667
SEN2641	136	87	72.33333	1.450207	0.06661	1.291084	1.291084352	0.368583261	0.368583	0.192656	227	140	115	160.6667	251	219	229	233
SEN2644	2149	1827	1617.333	2.17767	0.000177	1.948277	1.948276693	0.962198582	0.962199	0.001885	2061	1007	1052	1373.333	2937	2879	3156	2990.667
SEN2647	127	46	71.33333	1.349673	0.136916	1.195012	1.195011876	0.257024956	0.257025	0.306556	238	217	157	204	284	263	279	275.3333
SEN2648	15	9	-5.33333	-1.13223	0.115795	-1.28106	1.281055387	0.357332853	-0.35733	0.276448	50	51	36	45.66667	38	46	37	40.33333
SEN2649	5	2	0	-1	0.869448	-1.13231	1.132314258	0.179274414	-0.17927	0.956436	7	6	4	5.666667	6	3	8	5.666667
SEN2657	128	41	45	1.219156	0.55117	1.077752	1.077752327	0.108025677	0.108026	0.737835	250	208	158	205.3333	286	216	249	250.3333
SEN2661	59	22	33.33333	1.473934	0.05568	1.303699	1.303699294	0.382611142	0.382611	0.168238	85	65	61	70.33333	104	120	87	103.6667

Feature ID	Experiment - Range (original values)	Experiment - IQR (original values)	Experiment - Difference (original values)	Experiment - Fold Change (original values)	EDGE test: yccT NT vs WT NT , tagwise dispersions - P-value	EDGE test: yccT NT vs WT NT , tagwise dispersions - Fold change	yccT NT vs WT NT ABS|FC|	yccT NT vs WT NT Log2FC	yccT NT vs WT NT Log2FC +-	EDGE test: yccT NT vs WT NT , tagwise dispersions - FDR p-value correction	WT NT - WT.1.S22 - Expression values	WT NT - WT.2.S23 - Expression values	WT NT - WT.3.S24 - Expression values	WT NT - Means	yccT NT - yccT.1.S28 - Expression values	yccT NT - yccT.2.S29 - Expression values	yccT NT - yccT.3.S30 - Expression values	yccT NT - Means
SEN2669	39	16	26	1.134948	0.988865	-1.0018	1.001804271	0.002600668	-0.0026	1	197	198	183	192.6667	221	222	213	218.6667
SEN2681	100	32	26.66667	1.171674	0.767037	1.042572	1.042572197	0.060147292	0.060147	0.886011	206	154	106	155.3333	190	170	186	182
SEN2685	21	2	10.33333	1.131915	0.975857	-1.00637	1.006373659	0.009166066	-0.00917	1	73	80	82	78.33333	82	90	94	88.66667
SEN2710	127	66	83.66667	1.919414	8.84E-05	1.690171	1.690170602	0.757168876	0.757169	0.001081	112	72	89	91	170	155	199	174.6667
SEN2712	119	80	84.33333	1.587007	0.004322	1.404005	1.404004984	0.489548057	0.489548	0.023788	179	135	117	143.6667	236	215	233	228
SEN2742	8	5	3	1.25	0.756557	1.101448	1.101448495	0.139402035	0.139402	0.880907	17	9	10	12	15	15	15	15
SEN2743	48	10	25	1.158898	0.863273	1.021838	1.021837544	0.031165848	0.031166	0.952243	169	163	140	157.3333	186	188	173	182.3333
SEN2744	125	94	93	1.420814	0.046743	1.254932	1.254932323	0.327609563	0.32761	0.149543	257	211	195	221	320	317	305	314
SEN2746	12	6	3	1.123288	1	-1.01269	1.012694065	0.018198402	-0.0182	1	21	31	21	24.33333	33	27	22	27.33333
SEN2746A	69	48	50.66667	1.47352	0.024811	1.301132	1.301131804	0.379767114	0.379767	0.092075	124	106	91	107	160	159	154	157.6667
SEN2746B	20	1	8.333333	1.170068	0.892278	1.029799	1.029799453	0.042363409	0.042363	0.968565	51	52	44	49	57	51	64	57.33333
SEN2747	51	42	40.66667	1.460377	0.034958	1.28768	1.287679709	0.36477379	0.364774	0.120078	101	84	80	88.33333	130	131	126	129
SEN2749	27	12	-0.66667	-1.01136	0.364969	-1.15146	1.151457234	0.203460829	-0.20346	0.581929	57	72	49	59.33333	70	45	61	58.66667
SEN2750	47	11	20.66667	1.462687	0.108575	1.300234	1.30023369	0.378770942	0.378771	0.265358	58	47	29	44.66667	53	67	76	65.33333
SEN2751	266	168	153.6667	1.579874	0.026109	1.415842	1.415842249	0.501660532	0.501661	0.096099	394	226	175	265	441	427	388	418.6667
SEN2752	5333	3745	2048	1.663284	0.177113	1.527674	1.527673861	0.611336579	0.611337	0.36497	6635	1326	1302	3087.667	5678	4658	5071	5135.667
SEN2753	2731	1990	1323	1.628802	0.110427	1.472895	1.472894939	0.558654527	0.558655	0.267581	3823	1092	1397	2104	3651	3387	3243	3427
SEN2759	121	85	52	1.131757	1	-1.00077	1.000774703	0.001117228	-0.00112	1	474	357	353	394.6667	471	427	442	446.6667
SEN2760	7	1	3.333333	1.357143	0.563884	1.191337	1.191336793	0.252581323	0.252581	0.745911	10	7	11	9.333333	14	13	11	12.66667
SEN2761	50	14	11.66667	1.083135	0.713036	-1.04643	1.046429899	0.065475667	-0.06548	0.849756	159	142	120	140.3333	150	136	170	152
SEN2762	40	26	25.33333	1.378109	0.123877	1.217298	1.217298215	0.283682644	0.283683	0.288136	82	65	54	67	94	91	92	92.33333
SEN2777	105	48	30	1.03861	0.455634	-1.1	1.100004818	0.137509843	-0.13751	0.664329	797	728	806	777	830	758	833	807
SEN2779	298	86	41	1.049878	0.549618	-1.07749	1.077486412	0.107669676	-0.10767	0.737094	988	788	690	822	926	789	874	863
SEN2781	166	17	-6.66667	-1.01003	0.277127	-1.14585	1.145845024	0.196411932	-0.19641	0.490199	678	751	585	671.3333	667	672	655	664.6667
SEN2782	301	110	-187.333	-1.30879	0.002164	-1.50086	1.500859346	0.58578878	-0.58579	0.013867	706	862	814	794	663	561	596	606.6667
SEN2789	538	91	-2.66667	-1.00146	0.367673	-1.13124	1.131235381	0.177899149	-0.1779	0.584556	2084	1854	1546	1828	1831	1882	1763	1825.333
SEN2794	15	1	-5.33333	-1.20513	0.088931	-1.3678	1.367803909	0.451861418	-0.45186	0.232091	28	38	28	31.33333	27	28	23	26
SEN2795	36	5	14	1.194444	0.694174	1.053681	1.05368059	0.075437598	0.075438	0.837373	79	82	55	72	91	77	90	86
SEN2796	12	1	4.333333	1.265306	0.667948	1.115732	1.115732428	0.157991085	0.157991	0.820992	18	19	12	16.33333	19	24	19	20.66667
SEN2797	3	0	1.333333	1.8	0.432822	1.546425	1.546424937	0.628936807	0.628937	0.644217	1	2	2	1.666667	2	4	3	3
SEN2798	5	2	-1.66667	-1.3125	0.344367	-1.47645	1.476446375	0.562128959	-0.56213	0.55873	9	6	6	7	4	8	4	5.333333
SEN2799	10	1	5	4.75	0.006039	3.919457	3.919456504	1.970653615	1.970654	0.031186	1	2	1	1.333333	11	6	2	6.333333
SEN2800	17	7	10	1.588235	0.122575	1.393393	1.3933934	0.478602635	0.478603	0.286611	14	21	16	17	27	23	31	27
SEN2804	4331	1293	1875.667	1.628013	0.177158	1.43446	1.434459637	0.520507374	0.520507	0.36497	4605	1043	3312	2986.667	5374	4247	4966	4862.333
SEN2808	670	81	20	1.01476	0.452996	-1.10878	1.10878167	0.148975313	-0.14898	0.662013	1729	1277	1059	1355	1452	1358	1315	1375
SEN2819A	185	60	-17.6667	-1.0351	0.229544	-1.1688	1.168796341	0.225023566	-0.22502	0.435593	544	602	417	521	465	520	525	503.3333

Feature ID	Experiment - Range (original values)	Experiment - IQR (original values)	Experiment - Difference (original values)	Experiment - Fold Change (original values)	EDGE test: yccT NT vs WT NT , tagwise dispersions - P-value	EDGE test: yccT NT vs WT NT , tagwise dispersions - Fold change	yccT NT vs WT NT ABS|FC|	yccT NT vs WT NT Log2FC	yccT NT vs WT NT Log2FC +-	EDGE test: yccT NT vs WT NT , tagwise dispersions - FDR p-value correction	WT NT - WT.1.S22 - Expression values	WT NT - WT.2.S23 - Expression values	WT NT - WT.3.S24 - Expression values	WT NT - Means	yccT NT - yccT.1.S28 - Expression values	yccT NT - yccT.2.S29 - Expression values	yccT NT - yccT.3.S30 - Expression values	yccT NT - Means
SEN2830	419	56	-18.3333	-1.02241	0.29594	-1.16975	1.169749884	0.226200086	-0.2262	0.512524	658	1077	774	836.3333	849	775	830	818
SEN2855	1102	434	626	1.360184	0.189114	1.2063	1.20630034	0.270589148	0.270589	0.380029	2117	1739	1358	1738	2459	2173	2460	2364
SEN2863	69	23	-13.6667	-1.05791	0.12896	-1.19653	1.196529246	0.25885566	-0.25886	0.296535	277	264	208	249.6667	241	245	222	236
SEN2864	966	596	616.6667	2.548117	3.28E-05	2.301413	2.301413363	1.202520133	1.20252	0.000495	663	328	204	398.3333	1170	951	924	1015
SEN2865	337	201	239.6667	1.89875	3.56E-05	1.68179	1.681790007	0.749997578	0.749998	0.000527	336	255	209	266.6667	546	456	517	506.3333
SEN2868	56	44	31.66667	1.307443	0.264573	1.158837	1.158837133	0.21267782	0.212678	0.47636	134	90	85	103	134	141	129	134.6667
SEN2869	28	11	12.66667	1.826087	0.032253	1.612417	1.612416546	0.689224493	0.689224	0.112885	25	14	7	15.33333	35	21	28	28
SEN2870	36	18	20.66667	1.666667	0.019749	1.467411	1.46741148	0.553273477	0.553273	0.077235	41	25	27	31	61	49	45	51.66667
SEN2874	136	81	90.66667	1.777143	0.000521	1.580725	1.580725268	0.660586647	0.660587	0.004537	148	120	82	116.6667	201	218	203	207.3333
SEN2875	25	10	18	1.574468	0.043067	1.384472	1.384472194	0.469336079	0.469336	0.140702	32	33	29	31.33333	54	52	42	49.33333
SEN2876	45	25	32.66667	2.076923	8.73E-05	1.831144	1.831144098	0.872745326	0.872745	0.001071	36	34	21	30.33333	66	64	59	63
SEN2877	716	314	392.3333	1.369661	0.131692	1.212977	1.212977092	0.278552304	0.278552	0.299398	1290	1041	853	1061.333	1569	1355	1437	1453.667
SEN2878	445	230	274.6667	1.39313	0.106287	1.236008	1.236008127	0.305688229	0.305688	0.261207	866	653	577	698.6667	1022	1015	883	973.3333
SEN2879	17	8	10	1.428571	0.21909	1.255679	1.255678567	0.328467206	0.328467	0.422448	26	22	22	23.33333	39	31	30	33.33333
SEN2880	514	403	291	1.429626	0.110895	1.280518	1.280517988	0.356727519	0.356728	0.268238	988	570	474	677.3333	988	973	944	968.3333
SEN2881	495	353	270.3333	1.224095	0.535399	1.08637	1.08637037	0.119516037	0.119516	0.726399	1496	1122	1001	1206.333	1475	1489	1466	1476.667
SEN2895	40	4	-2.33333	-1.01049	0.282872	-1.14835	1.148351691	0.199564545	-0.19956	0.497779	212	247	215	224.6667	208	248	211	222.3333
SEN2914	156	70	-16	-1.06443	0.232351	-1.22967	1.229668077	0.298268943	-0.29827	0.439582	170	326	297	264.3333	243	216	286	248.3333
SEN2915	299	53	-36.6667	-1.09499	0.208422	-1.27359	1.273588232	0.34889891	-0.3489	0.408999	249	471	548	422.6667	375	365	418	386
SEN2916	261	103	-61	-1.14055	0.096348	-1.32228	1.322284945	0.403033103	-0.40303	0.245074	348	528	609	495	439	380	483	434
SEN2917	167	75	-51.6667	-1.10238	0.093508	-1.26961	1.269611965	0.34438763	-0.34439	0.239915	463	586	620	556.3333	538	453	523	504.6667
SEN2918	158	46	-35.6667	-1.10918	0.103014	-1.28185	1.281853053	0.358230886	-0.35823	0.256724	279	371	437	362.3333	338	298	344	326.6667
SEN2923	24	8	10	1.714286	0.145548	1.505786	1.505786378	0.590517114	0.590517	0.319268	18	10	14	14	33	30	9	24
SEN2924	38	20	26.33333	1.461988	0.06721	1.28461	1.284610363	0.36133084	0.361331	0.19377	55	65	51	57	89	75	86	83.33333
SEN2925	40	26	27	1.28223	0.325188	1.129467	1.129466695	0.17564173	0.175642	0.539262	107	88	92	95.66667	118	122	128	122.6667
SEN2926	32	8	10.66667	1.191617	0.730286	1.053007	1.053007058	0.074515106	0.074515	0.862735	65	56	46	55.66667	57	64	78	66.33333
SEN2928	609	339	319	1.19736	0.677772	1.057589	1.057589442	0.080779679	0.08078	0.826875	1880	1541	1428	1616.333	2037	1921	1848	1935.333
SEN2929	40	13	24.33333	1.608333	0.017492	1.416258	1.416257808	0.50208391	0.502084	0.07059	46	43	31	40	71	56	66	64.33333
SEN2935	57	13	18.33333	1.087719	0.757114	-1.04042	1.040423622	0.05717106	-0.05717	0.880907	225	220	182	209	212	239	231	227.3333
SEN2941	110	48	69.66667	1.377256	0.091659	1.215121	1.215121142	0.281100152	0.2811	0.23703	213	184	157	184.6667	267	232	264	254.3333
SEN2960	118	63	72.66667	1.721854	0.001142	1.527363	1.527363224	0.611043192	0.611043	0.00849	128	98	76	100.6667	161	165	194	173.3333
SEN2961	294	182	175.6667	1.412041	0.084004	1.254888	1.254887848	0.327558433	0.327558	0.224231	557	383	339	426.3333	633	608	565	602
SEN2962	187	115	122	1.618243	0.004906	1.438607	1.438607107	0.524672636	0.524673	0.026336	260	188	144	197.3333	331	303	324	319.3333
SEN2963	293	161	123	1.362475	0.213199	1.222073	1.2220728	0.28933023	0.28933	0.413972	509	293	216	339.3333	495	454	438	462.3333
SEN2964	54	20	6.666667	1.02448	0.375181	-1.10829	1.108289241	0.148334445	-0.14833	0.5933	297	273	247	272.3333	301	278	258	279
SEN2965	737	363	414.3333	1.411181	0.09415	1.257655	1.257654978	0.330736191	0.330736	0.240702	1282	1016	725	1007.667	1462	1425	1379	1422

Feature ID	Experiment - Range (original values)	Experiment - IQR (original values)	Experiment - Difference (original values)	Experiment - Fold Change (original values)	EDGE test: yccT NT vs WT NT , tagwise dispersions - P-value	EDGE test: yccT NT vs WT NT , tagwise dispersions - Fold change	yccT NT vs WT NT ABS|FC|	yccT NT vs WT NT Log2FC	yccT NT vs WT NT Log2FC +-	EDGE test: yccT NT vs WT NT , tagwise dispersions - FDR p-value correction	WT NT - WT.1.S22 - Expression values	WT NT - WT.2.S23 - Expression values	WT NT - WT.3.S24 - Expression values	WT NT - Means	yccT NT - yccT.1.S28 - Expression values	yccT NT - yccT.2.S29 - Expression values	yccT NT - yccT.3.S30 - Expression values	yccT NT - Means
SEN2966	83	66	59	1.650735	0.003171	1.45877	1.458770387	0.544752818	0.544753	0.018986	118	77	77	90.66667	160	143	146	149.6667
SEN2967	41	12	23.33333	1.752688	0.006745	1.546359	1.546358638	0.628874954	0.628875	0.034002	35	35	23	31	47	52	64	54.33333
SEN2968	88	49	59	1.907692	8.86E-05	1.688339	1.688338924	0.755604546	0.755605	0.001081	85	63	47	65	125	112	135	124
SEN2969	143	76	91.33333	1.83792	0.000215	1.630621	1.630620956	0.705421461	0.705421	0.002137	141	109	77	109	220	196	185	200.3333
SEN2970	97	55	64.66667	1.613924	0.003358	1.425081	1.425080751	0.511043671	0.511044	0.019867	126	101	89	105.3333	168	156	186	170
SEN2971	110	53	69	1.915929	0.000119	1.699301	1.699300936	0.764941368	0.764941	0.001356	96	79	51	75.33333	140	132	161	144.3333
SEN2972	135	43	80.33333	1.247179	0.421195	1.098662	1.098662415	0.135748158	0.135748	0.633939	353	335	287	325	422	378	416	405.3333
SEN2973	107	70	84	1.7875	0.000244	1.567873	1.567872531	0.648808272	0.648808	0.002365	101	116	103	106.6667	191	173	208	190.6667
SEN2974	186	135	146.3333	2.233146	1.79E-08	1.975914	1.975913887	0.982520073	0.98252	8.19E-07	137	119	100	118.6667	254	286	255	265
SEN2975	74	41	52.33333	1.813472	0.000147	1.595283	1.595283084	0.673812454	0.673812	0.001628	69	68	56	64.33333	130	111	109	116.6667
SEN2976	21	7	6.666667	1.363636	0.436431	1.209194	1.209194012	0.27404574	0.274046	0.647414	26	21	8	18.33333	29	27	19	25
SEN2977	4999	540	-2463.33	-1.90675	0.00012	-2.1503	2.150299875	1.104537868	-1.10454	0.001368	4702	7562	3276	5180	2851	2736	2563	2716.667
SEN2978	5328	545	-2680	-1.81278	0.000196	-2.0435	2.043497171	1.031040246	-1.03104	0.002014	5608	8366	3958	5977.333	3413	3441	3038	3297.333
SEN2979	4648	1331	-2438	-2.21033	2.72E-06	-2.51223	2.512226414	1.328966493	-1.32897	6.17E-05	3501	6527	3329	4452.333	2166	1998	1879	2014.333
SEN2980	3285	1014	-1809.33	-1.8383	4.5E-05	-2.09642	2.096424861	1.067931123	-1.06793	0.000634	3146	5388	3369	3967.667	2240	2103	2132	2158.333
SEN2981	1358	849	890.3333	1.41488	0.133934	1.256176	1.256176036	0.329038652	0.329039	0.302169	2614	2097	1727	2146	3085	3078	2946	3036.333
SEN2984	89	24	-52.6667	-1.38725	0.000699	-1.58982	1.589821449	0.668864747	-0.66886	0.005776	155	217	194	188.6667	149	128	131	136
SEN2985	194	65	-69	-1.35264	0.017665	-1.58067	1.58066583	0.660532399	-0.66053	0.070967	159	282	353	264.6667	231	166	190	195.6667
SEN2995	1065	621	793.6667	1.635781	0.004962	1.442448	1.442448065	0.528519376	0.528519	0.026491	1376	1274	1095	1248.333	2071	1895	2160	2042
SEN2997	699	500	522.6667	1.988028	1.75E-05	1.768256	1.768256349	0.822327442	0.822327	0.000293	670	525	392	529	1091	1025	1039	1051.667
SEN2998	528	362	370	1.925771	0.000127	1.717966	1.717965783	0.780701302	0.780701	0.001433	541	376	282	399.6667	810	761	738	769.6667
SEN2999	596	364	395	1.855596	0.000297	1.653355	1.65335529	0.725396779	0.725397	0.002793	603	461	321	461.6667	917	825	828	856.6667
SEN3003	15	5	1.333333	1.039604	0.613499	-1.099	1.099001984	0.13619399	-0.13619	0.782388	27	36	38	33.66667	42	32	31	35
SEN3009	18	14	9	1.341772	0.37979	1.183134	1.183134198	0.242613722	0.242614	0.596121	36	21	22	26.33333	36	31	39	35.33333
SEN3010	365	193	218	1.32651	0.198715	1.176672	1.176671816	0.234711996	0.234712	0.395378	808	649	546	667.6667	904	911	842	885.6667
SEN3012	157	123	109	1.787952	0.00112	1.592219	1.592219286	0.671039043	0.671039	0.008385	194	109	112	138.3333	235	266	241	247.3333
SEN3013	22	11	12.66667	1.567164	0.07896	1.377921	1.377921096	0.462493277	0.462493	0.215971	28	19	20	22.33333	41	31	33	35
SEN3018	248	62	-88.6667	-1.13123	0.042671	-1.28898	1.288981977	0.366232092	-0.36623	0.139538	757	853	683	764.3333	742	605	680	675.6667
SEN3034	370	215	218.6667	1.535948	0.030837	1.35766	1.357659907	0.44112213	0.441122	0.10958	542	303	379	408	673	594	613	626.6667
SEN3035	173	130	88.66667	1.492593	0.102496	1.334163	1.334163072	0.415935015	0.415935	0.255931	285	112	143	180	278	273	255	268.6667
SEN3036	147	82	48	1.182278	0.732655	1.049407	1.04940669	0.069573892	0.069574	0.864336	354	207	229	263.3333	305	311	318	311.3333
SEN3040	37	16	20.66667	1.413333	0.119518	1.245903	1.245902864	0.317191594	0.317192	0.281854	52	60	38	50	75	69	68	70.66667
SEN3058	1239	696	608.3333	1.361458	0.2725	1.205713	1.205712555	0.269886006	0.269886	0.485699	1545	2349	1155	1683	2241	2239	2394	2291.333
SEN3091	48	11	23.33333	1.286885	0.389893	1.131062	1.131062264	0.17767835	0.177678	0.60682	92	63	89	81.33333	103	111	100	104.6667
SEN3092	167	18	65.33333	1.150192	0.90902	1.014408	1.014408015	0.020638049	0.020638	0.976926	478	460	367	435	534	463	504	500.3333
SEN3093	110	69	10	1.027347	0.409382	-1.10583	1.105832953	0.145133468	-0.14513	0.623787	392	398	307	365.6667	417	323	387	375.6667

Feature ID	Experiment - Range (original values)	Experiment - IQR (original values)	Experiment - Difference (original values)	Experiment - Fold Change (original values)	EDGE test: yccT NT vs WT NT , tagwise dispersions - P-value	EDGE test: yccT NT vs WT NT , tagwise dispersions - Fold change	yccT NT vs WT NT ABS|FC|	yccT NT vs WT NT Log2FC	yccT NT vs WT NT Log2FC +-	EDGE test: yccT NT vs WT NT , tagwise dispersions - FDR p-value correction	WT NT - WT.1.S22 - Expression values	WT NT - WT.2.S23 - Expression values	WT NT - WT.3.S24 - Expression values	WT NT - Means	yccT NT - yccT.1.S28 - Expression values	yccT NT - yccT.2.S29 - Expression values	yccT NT - yccT.3.S30 - Expression values	yccT NT - Means
SEN3094	65	47	-2	-1.00542	0.271314	-1.14232	1.14232392	0.191971803	-0.19197	0.484104	381	399	334	371.3333	397	377	334	369.3333
SEN3095	387	159	0.333333	1.000225	0.32918	-1.13389	1.133888295	0.18127852	-0.18128	0.543037	1567	1632	1245	1481.333	1584	1408	1453	1481.667
SEN3096	50	12	-11.6667	-1.09115	0.082655	-1.23778	1.237782619	0.307757969	-0.30776	0.221967	141	164	114	139.6667	134	122	128	128
SEN3097	283	129	161.3333	1.126569	0.955242	-1.00746	1.007458078	0.010719806	-0.01072	1	1360	1298	1166	1274.667	1432	1427	1449	1436
SEN3112	6	2	-1.33333	-1.0625	0.39264	-1.2049	1.204895247	0.268907725	-0.26891	0.609811	22	26	20	22.66667	20	24	20	21.33333
SEN3113	10	2	4.333333	1.565217	0.332268	1.37298	1.372979562	0.457310149	0.45731	0.546001	8	6	9	7.666667	10	16	10	12
SEN3125	855	229	-551.333	-1.62017	5.28E-05	-1.85806	1.858060868	0.893797763	-0.8938	0.000718	1108	1714	1499	1440.333	879	929	859	889
SEN3135	137	22	59.66667	1.169829	0.825975	1.02721	1.027209984	0.038731131	0.038731	0.927966	368	360	326	351.3333	463	388	382	411
SEN3166	64	17	-20.3333	-1.05398	0.115985	-1.19997	1.199974349	0.263003567	-0.263	0.276605	418	395	378	397	398	354	378	376.6667
SEN3167	1534	107	-610	-1.51724	0.002181	-1.72277	1.722766269	0.784726982	-0.78473	0.013946	1410	2663	1295	1789.333	1221	1129	1188	1179.333
SEN3176	898	392	197	1.145245	0.877401	1.023637	1.023636636	0.033703688	0.033704	0.959942	1877	1213	979	1356.333	1624	1605	1431	1553.333
SEN3183	37	10	-2.33333	-1.02047	0.221208	-1.16143	1.161429341	0.215901386	-0.2159	0.425237	127	116	106	116.3333	132	95	115	114
SEN3184	23	1	7	1.355932	0.42355	1.20277	1.202769567	0.26636027	0.26636	0.635971	24	27	8	19.66667	24	25	31	26.66667
SEN3185	47	10	19	1.802817	0.02948	1.583858	1.583858408	0.663443369	0.663443	0.106113	32	22	17	23.66667	64	32	32	42.66667
SEN3186	7	1	2.666667	2.142857	0.303251	1.841652	1.841651657	0.881000206	0.881	0.518605	2	3	2	2.333333	6	1	8	5
SEN3187	10	9	-0.66667	-1.01681	0.376674	-1.15611	1.156108612	0.209276939	-0.20928	0.594928	43	34	44	40.33333	41	44	34	39.66667
SEN3188	26	5	1.333333	1.026846	0.560063	-1.10718	1.107182306	0.146892792	-0.14689	0.744343	62	40	47	49.66667	66	45	42	51
SEN3189	23	7	14.33333	1.68254	0.043154	1.474399	1.474399308	0.560127299	0.560127	0.140702	21	20	22	21	43	28	35	35.33333
SEN3190	80	58	56.33333	1.19403	0.665777	1.052405	1.052405319	0.073690446	0.07369	0.819919	317	286	268	290.3333	344	348	348	346.6667
SEN3191	115	36	62.33333	1.142097	0.97028	1.005185	1.005184569	0.007460429	0.00746	1	474	438	404	438.6667	512	472	519	501
SEN3195	47	22	23	1.2749	0.364014	1.122278	1.122277805	0.16642984	0.16643	0.580823	100	73	78	83.66667	120	99	101	106.6667
SEN3211	505	44	-320	-1.3781	0.003328	-1.59008	1.59008132	0.66910055	-0.6691	0.019716	866	1303	1330	1166.333	879	825	835	846.3333
SEN3212	89	33	-19.3333	-1.03856	0.166668	-1.18244	1.182437176	0.241763534	-0.24176	0.351334	515	568	479	520.6667	534	488	482	501.3333
SEN3222	93	48	39	1.118541	0.904142	-1.01506	1.015063319	0.021569725	-0.02157	0.974047	369	321	297	329	381	333	390	368
SEN3239	203	161	156	1.680233	0.001825	1.483705	1.483705362	0.569204627	0.569205	0.012235	283	190	215	229.3333	393	387	376	385.3333
SEN3273	21325	9193	-13344.3	-2.55305	3.23E-08	-2.9152	2.915199978	1.543594853	-1.54359	1.38E-06	17571	29449	18790	21936.67	9275	8124	8378	8592.333
SEN3288	776	153	-236.667	-1.09105	0.16331	-1.23953	1.239533687	0.30979748	-0.3098	0.345901	3165	2566	2777	2836	2690	2719	2389	2599.333
SEN3334	128	90	54.66667	1.164493	0.822164	1.029979	1.02997945	0.042615553	0.042616	0.924153	410	282	305	332.3333	395	408	358	387
SEN3342	196	71	-78	-1.06331	0.129386	-1.21058	1.210576603	0.275694373	-0.27569	0.296753	1361	1311	1258	1310	1301	1230	1165	1232
SEN3343	94	17	33.33333	1.082576	0.710838	-1.04623	1.046226917	0.065195793	-0.0652	0.848296	443	417	351	403.6667	434	432	445	437
SEN3346	160	99	100.3333	1.796296	0.000671	1.595719	1.595719018	0.674206637	0.674207	0.005601	175	112	91	126	251	211	217	226.3333
SEN3352	445	114	4.333333	1.002328	0.362825	-1.13501	1.135010231	0.182705302	-0.18271	0.579307	1808	2111	1666	1861.667	1904	1790	1904	1866
SEN3353	198	64	92	1.247756	0.422356	1.108079	1.108079289	0.148061117	0.148061	0.634824	449	385	280	371.3333	478	463	449	463.3333
SEN3354	44	14	26	1.312	0.281695	1.156198	1.156198387	0.209388965	0.209389	0.496497	90	80	80	83.33333	110	124	94	109.3333
SEN3354A	57	36	39.66667	1.748428	0.00173	1.540186	1.540185759	0.623104362	0.623104	0.01176	68	42	49	53	99	85	94	92.66667
SEN3355	134	50	74.33333	1.648256	0.011951	1.446782	1.44678216	0.532847714	0.532848	0.052954	144	79	121	114.6667	213	171	183	189

Feature ID	Experiment - Range (original values)	Experiment - IQR (original values)	Experiment - Difference (original values)	Experiment - Fold Change (original values)	EDGE test: yccT NT vs WT NT , tagwise dispersions - P-value	EDGE test: yccT NT vs WT NT , tagwise dispersions - Fold change	yccT NT vs WT NT ABS|FC|	yccT NT vs WT NT Log2FC	yccT NT vs WT NT Log2FC +-	EDGE test: yccT NT vs WT NT , tagwise dispersions - FDR p-value correction	WT NT - WT.1.S22 - Expression values	WT NT - WT.2.S23 - Expression values	WT NT - WT.3.S24 - Expression values	WT NT - Means	yccT NT - yccT.1.S28 - Expression values	yccT NT - yccT.2.S29 - Expression values	yccT NT - yccT.3.S30 - Expression values	yccT NT - Means
SEN3356	155	47	78	1.230542	0.576899	1.074851	1.074850606	0.104136153	0.104136	0.755614	304	379	332	338.3333	459	373	417	416.3333
SEN3357	221	90	-150.667	-1.11011	0.075278	-1.26659	1.266593476	0.340953553	-0.34095	0.208213	1458	1589	1510	1519	1368	1368	1369	1368.333
SEN3370	145	27	-71.6667	-1.97727	1.91E-05	-2.25206	2.252061282	1.171246086	-1.17125	0.000316	98	213	124	145	71	81	68	73.33333
SEN3371	155	21	-72.6667	-1.83206	4.1E-05	-2.08949	2.089488963	1.063150138	-1.06315	0.000593	113	231	136	160	94	92	76	87.33333
SEN3372	53	12	-26.3333	-1.54861	0.000738	-1.76236	1.762356031	0.817505406	-0.81751	0.006009	59	100	64	74.33333	50	47	47	48
SEN3373	83	18	-31.6667	-1.17399	0.020095	-1.34171	1.34170696	0.424069609	-0.42407	0.07838	188	249	204	213.6667	199	166	181	182
SEN3381	100	56	54	1.640316	0.016941	1.466827	1.466827215	0.552698938	0.552699	0.068868	123	71	59	84.33333	127	159	129	138.3333
SEN3389	28	2	-9	-1.13366	0.054494	-1.28629	1.286287227	0.363212831	-0.36321	0.165612	89	71	69	76.33333	71	61	70	67.33333
SEN3403	530	169	-202.333	-1.05617	0.236291	-1.20043	1.200427311	0.263548047	-0.26355	0.444591	3903	3988	3522	3804.333	3691	3657	3458	3602
SEN3418	155	91	-90.3333	-1.1615	0.023864	-1.32952	1.329518231	0.41090356	-0.4109	0.089733	639	639	671	649.6667	614	516	548	559.3333
SEN3421	1663	1099	1324.333	4.366949	4.02E-13	3.895762	3.895762416	1.961905697	1.961906	4.15E-11	620	257	303	393.3333	1831	1402	1920	1717.667
SEN3422	3448	2237	2743.667	6.7924	9.65E-08	6.21852	6.218520231	2.636571315	2.636571	3.5E-06	1043	165	213	473.6667	3613	2450	3589	3217.333
SEN3423	4957	3639	4010.333	6.973684	1.06E-06	6.426158	6.426157804	2.683956409	2.683956	2.88E-05	1550	197	267	671.3333	5154	3906	4985	4681.667
SEN3424	9631	6923	7406	4.610335	1.89E-06	4.197833	4.197832588	2.069644632	2.069645	4.56E-05	4160	889	1105	2051.333	10520	8028	9824	9457.333
SEN3425	227	112	120.6667	1.279321	0.32601	1.128507	1.128506802	0.174415114	0.174415	0.540018	511	400	385	432	612	534	512	552.6667
SEN3427	1175	490	167	1.043015	0.599977	-1.08599	1.085994131	0.119016306	-0.11902	0.774169	4192	4315	3140	3882.333	4295	3702	4151	4049.333
SEN3428	23	15	12.33333	1.770833	0.047889	1.560319	1.560319256	0.641841248	0.641841	0.151677	26	11	11	16	34	20	31	28.33333
SEN3447A	89	19	46	1.479167	0.042406	1.307146	1.307145851	0.386420125	0.38642	0.138879	117	98	73	96	147	117	162	142
SEN3448	68	35	47	1.550781	0.009087	1.367914	1.367913753	0.451977271	0.451977	0.043155	95	90	71	85.33333	139	133	125	132.3333
SEN3455	57	11	18.33333	1.115063	0.934674	-1.01365	1.01364555	0.019553261	-0.01955	0.989852	173	176	129	159.3333	165	182	186	177.6667
SEN3456	63	19	30.33333	1.32852	0.204808	1.176746	1.176746453	0.234803505	0.234804	0.403336	115	96	66	92.33333	129	115	124	122.6667
SEN3457	49	44	41.33333	1.426117	0.057582	1.255008	1.255007512	0.327696	0.327696	0.172732	106	92	93	97	141	137	137	138.3333
SEN3473	376	127	139	1.224677	0.597527	1.081051	1.081050648	0.112434116	0.112434	0.772358	758	470	628	618.6667	755	846	672	757.6667
SEN3474	889	583	673.3333	2.027467	1.09E-05	1.801156	1.801155554	0.848922783	0.848923	0.000196	818	657	491	655.3333	1380	1240	1366	1328.667
SEN3475	282	221	219.6667	2.209174	5.12E-07	1.964699	1.964699189	0.974308442	0.974308	1.53E-05	241	159	145	181.6667	397	427	380	401.3333
SEN3476	36	20	23	1.621622	0.016282	1.427925	1.42792472	0.513919923	0.51392	0.066986	46	34	31	37	67	54	59	60
SEN3476A	8	4	3.333333	1.625	0.372567	1.425012	1.425011877	0.510973944	0.510974	0.59022	9	5	2	5.333333	10	9	7	8.666667
SEN3476B	11	8	8	2.411765	0.011404	2.104043	2.104042532	1.073163868	1.073164	0.051093	8	5	4	5.666667	13	15	13	13.66667
SEN3479	85	44	52	1.360277	0.147704	1.198724	1.198723908	0.261499413	0.261499	0.322402	140	164	129	144.3333	191	214	184	196.3333
SEN3492	15	3	7.666667	1.396552	0.334161	1.227126	1.227125574	0.29528289	0.295283	0.548192	20	16	22	19.33333	23	27	31	27
SEN3500	272	235	206.6667	1.319423	0.25201	1.158355	1.158354823	0.212077242	0.212077	0.463289	605	733	603	647	846	840	875	853.6667
SEN3501	218	161	150.3333	1.384812	0.097193	1.226739	1.226738823	0.294828127	0.294828	0.246092	468	379	325	390.6667	543	540	540	541
SEN3503	39	14	-8	-1.04706	0.131957	-1.18873	1.188727894	0.249418512	-0.24942	0.29957	185	194	155	178	175	161	174	170
SEN3510	25	6	6.333333	1.063758	0.609756	-1.06568	1.06567779	0.091771301	-0.09177	0.780424	108	102	88	99.33333	99	105	113	105.6667
SEN3512	9	5	5.666667	3.428571	0.006917	2.927672	2.927671667	1.549753767	1.549754	0.034556	4	2	1	2.333333	7	10	7	8
SEN3513	181	49	60.66667	1.07913	0.688895	-1.05248	1.052480104	0.073792961	-0.07379	0.834791	764	847	689	766.6667	799	813	870	827.3333

Feature ID	Experiment - Range (original values)	Experiment - IQR (original values)	Experiment - Difference (original values)	Experiment - Fold Change (original values)	EDGE test: yccT NT vs WT NT , tagwise dispersions - P-value	EDGE test: yccT NT vs WT NT , tagwise dispersions - Fold change	yccT NT vs WT NT ABS|FC|	yccT NT vs WT NT Log2FC	yccT NT vs WT NT Log2FC +-	EDGE test: yccT NT vs WT NT , tagwise dispersions - FDR p-value correction	WT NT - WT.1.S22 - Expression values	WT NT - WT.2.S23 - Expression values	WT NT - WT.3.S24 - Expression values	WT NT - Means	yccT NT - yccT.1.S28 - Expression values	yccT NT - yccT.2.S29 - Expression values	yccT NT - yccT.3.S30 - Expression values	yccT NT - Means
SEN3518	349	296	240.3333	1.476852	0.045099	1.3131	1.313100353	0.392977177	0.392977	0.145229	675	430	407	504	756	726	751	744.3333
SEN3519	33	23	23.33333	1.22082	0.617166	1.070297	1.070296728	0.098010823	0.098011	0.78418	105	98	114	105.6667	128	128	131	129
SEN3520	162	61	44.66667	1.053472	0.518195	-1.08649	1.086488443	0.119672829	-0.11967	0.715749	767	896	843	835.3333	929	825	886	880
SEN3558	62	38	-9	-1.02761	0.181309	-1.16868	1.168680779	0.224880916	-0.22488	0.369879	361	341	303	335	365	303	310	326
SEN3559	33	3	-6.66667	-1.05479	0.140662	-1.20186	1.20186165	0.265270833	-0.26527	0.312575	117	149	119	128.3333	129	116	120	121.6667
SEN3572	24	14	10	1.3	0.433542	1.149341	1.149340545	0.200806327	0.200806	0.644422	43	29	28	33.33333	43	52	35	43.33333
SEN3577A	33	10	10.33333	1.182353	0.761579	1.047225	1.047224534	0.066570801	0.066571	0.882712	73	57	40	56.66667	69	67	65	67
SEN3588	46	13	11	1.053485	0.535178	-1.07629	1.076288194	0.106064434	-0.10606	0.726399	226	210	181	205.6667	227	218	205	216.6667
SEN3589	110	22	-45.6667	-1.42154	0.001485	-1.6214	1.621397271	0.69723762	-0.69724	0.010431	126	205	131	154	126	104	95	108.3333
SEN3590	344	81	-172.667	-2.37401	1.14E-07	-2.70622	2.706217531	1.436277811	-1.43628	4.09E-06	212	446	237	298.3333	144	131	102	125.6667
SEN3591	368	137	-223.333	-2.88202	3.73E-12	-3.28151	3.281511314	1.714360407	-1.71436	3.11E-10	254	483	289	342	117	124	115	118.6667
SEN3592	379	126	-226.667	-3.89362	1.41E-14	-4.45491	4.454909178	2.15539602	-2.1554	1.73E-12	202	449	264	305	89	76	70	78.33333
SEN3593	480	143	-266	-4	1.21E-11	-4.53894	4.538942083	2.18235608	-2.18236	9.57E-10	224	548	292	354.6667	81	117	68	88.66667
SEN3594	439	99	-231.667	-6.22556	1.93E-13	-7.05938	7.05938476	2.819542455	-2.81954	2.2E-11	143	474	211	276	35	54	44	44.33333
SEN3595	555	361	-390.667	-1.18873	0.036736	-1.355	1.354995513	0.438288074	-0.43829	0.125022	2527	2478	2377	2460.667	2222	2016	1972	2070
SEN3596	16	4	2	1.092308	1	-1.03403	1.034030052	0.048278115	-0.04828	1	30	21	14	21.66667	24	27	20	23.66667
SEN3597	1200	490	-791.333	-2.03038	1E-08	-2.31982	2.319824729	1.214015809	-1.21402	4.87E-07	1290	1903	1485	1559.333	800	801	703	768
SEN3599	84	22	-41.6667	-1.24704	0.004283	-1.42186	1.421857801	0.50777719	-0.50778	0.023649	191	244	196	210.3333	177	160	169	168.6667
SEN3600	143	28	-66.3333	-1.19664	0.013724	-1.3658	1.365804226	0.449750703	-0.44975	0.058926	365	465	381	403.6667	353	322	337	337.3333
SEN3607	510	372	-414.667	-1.42823	0.000175	-1.62977	1.629772568	0.704670653	-0.70467	0.001876	1368	1367	1414	1383	995	1006	904	968.3333
SEN3608	679	354	-521	-1.69777	2.37E-06	-1.95044	1.950438524	0.963798526	-0.9638	5.56E-05	1093	1325	1385	1267.667	795	739	706	746.6667
SEN3609	665	314	-468.667	-1.71407	1.23E-06	-1.962	1.9619951	0.972321439	-0.97232	3.24E-05	989	1247	1139	1125	712	675	582	656.3333
SEN3610	862	382	508	1.61254	0.007207	1.428788	1.428788189	0.51479206	0.514792	0.03568	1013	824	651	829.3333	1513	1293	1206	1337.333
SEN3614	127	91	-10.6667	-1.02766	0.213436	-1.16527	1.165267644	0.220661358	-0.22066	0.414251	430	443	316	396.3333	427	336	394	385.6667
SEN3616	91	65	61	1.215294	0.572449	1.070294	1.070294318	0.098007574	0.098008	0.751561	317	259	274	283.3333	339	344	350	344.3333
SEN3628	15	4	3.333333	1.27027	0.710325	1.120906	1.120905999	0.164665297	0.164665	0.847948	21	7	9	12.33333	22	13	12	15.66667
SEN3629	0	0	0	1	1	1	1	0	0	1	0	0	0	0	0	0	0	0
SEN3631	0	0	0	1	1	1	1	0	0	1	0	0	0	0	0	0	0	0
SEN3633	0	0	0	1	1	1	1	0	0	1	0	0	0	0	0	0	0	0
SEN3636	532	405	434	2.656489	4.49E-10	2.346048	2.346047739	1.230232371	1.230232	2.88E-08	334	207	245	262	699	650	739	696
SEN3645	46	14	-17	-1.29651	0.011059	-1.47424	1.474244552	0.559975862	-0.55998	0.049747	70	91	62	74.33333	71	56	45	57.33333
SEN3649	30	14	18.66667	1.474576	0.091688	1.302756	1.302755662	0.381566525	0.381567	0.23703	48	36	34	39.33333	50	64	60	58
SEN3650	107	60	66	1.511628	0.028069	1.339208	1.339208381	0.421380462	0.42138	0.10203	164	109	114	129	195	216	174	195
SEN3651	631	235	-252.333	-1.10235	0.137288	-1.25034	1.250335399	0.322315146	-0.32232	0.30677	2831	2972	2350	2717.667	2585	2470	2341	2465.333
SEN3658	76	25	6.666667	1.027663	0.416776	-1.11067	1.110668498	0.151428279	-0.15143	0.630463	215	291	217	241	275	226	242	247.6667
SEN3672	158	74	0	-1	0.300949	-1.13392	1.133918643	0.181317132	-0.18132	0.515966	676	595	518	596.3333	629	543	617	596.3333

Feature ID	Experiment - Range (original values)	Experiment - IQR (original values)	Experiment - Difference (original values)	Experiment - Fold Change (original values)	EDGE test: yccT NT vs WT NT , tagwise dispersions - P-value	EDGE test: yccT NT vs WT NT , tagwise dispersions - Fold change	yccT NT vs WT NT ABS|FC|	yccT NT vs WT NT Log2FC	yccT NT vs WT NT Log2FC +-	EDGE test: yccT NT vs WT NT , tagwise dispersions - FDR p-value correction	WT NT - WT.1.S22 - Expression values	WT NT - WT.2.S23 - Expression values	WT NT - WT.3.S24 - Expression values	WT NT - Means	yccT NT - yccT.1.S28 - Expression values	yccT NT - yccT.2.S29 - Expression values	yccT NT - yccT.3.S30 - Expression values	yccT NT - Means
SEN3673	10	3	-1	-1.03448	0.367203	-1.17876	1.178762135	0.237272623	-0.23727	0.584228	28	28	34	30	32	24	31	29
SEN3674	205	18	-82.6667	-1.25152	0.006973	-1.42661	1.426610468	0.512591465	-0.51259	0.034757	371	501	362	411.3333	344	296	346	328.6667
SEN3677	61	23	31.66667	1.203426	0.624175	1.059411	1.059410869	0.083262215	0.083262	0.788628	173	150	144	155.6667	205	186	171	187.3333
SEN3712	40	24	-1	-1.00511	0.29444	-1.14893	1.148926776	0.200286854	-0.20029	0.510818	174	202	214	196.6667	178	201	208	195.6667
SEN3713	45	16	-0.33333	-1.00106	0.271305	-1.13782	1.137823774	0.18627713	-0.18628	0.484104	319	333	288	313.3333	322	314	303	313
SEN3717	7	3	0	-1	0.467409	-1.13449	1.134491052	0.18204523	-0.18205	0.675502	33	31	26	30	29	28	33	30
SEN3728	217	82	-126.333	-1.63484	9.55E-06	-1.88357	1.883566103	0.913466665	-0.91347	0.000179	295	301	380	325.3333	213	163	221	199
SEN3732	150	52	-105	-1.38698	0.00044	-1.59162	1.591623136	0.670498775	-0.6705	0.003949	326	398	405	376.3333	285	255	274	271.3333
SEN3742	28	11	5.666667	1.099415	0.876031	-1.03061	1.030608076	0.043495802	-0.0435	0.959152	64	66	41	57	69	54	65	62.66667
SEN3761	86	10	13.33333	1.040486	0.459555	-1.09336	1.093359764	0.12876819	-0.12877	0.668074	377	294	317	329.3333	380	321	327	342.6667
SEN3775	110	56	60.66667	1.191781	0.766869	1.040585	1.040584654	0.057394337	0.057394	0.886011	313	283	353	316.3333	393	369	369	377
SEN3797	39	19	18.33333	1.297297	0.31863	1.143499	1.14349908	0.193455205	0.193455	0.533578	76	56	53	61.66667	92	75	73	80
SEN3798	39	6	20.33333	2	0.005205	1.778695	1.778695271	0.830819367	0.830819	0.027592	27	23	11	20.33333	29	50	43	40.66667
SEN3799	26	9	13.33333	1.909091	0.016757	1.680643	1.680642623	0.749012978	0.749013	0.068244	19	15	10	14.66667	24	24	36	28
SEN3800	69	35	49.66667	1.716346	0.001252	1.512605	1.512604608	0.597034919	0.597035	0.009143	71	77	60	69.33333	122	129	106	119
SEN3801	23	14	16	2.021277	0.003586	1.777766	1.777766167	0.830065577	0.830066	0.020823	21	12	14	15.66667	28	32	35	31.66667
SEN3802	22	5	12.66667	1.283582	0.441234	1.126696	1.126696197	0.172098559	0.172099	0.65279	45	42	47	44.66667	58	50	64	57.33333
SEN3818	253	103	158.3333	1.273775	0.394656	1.11427	1.114269738	0.156098517	0.156099	0.6108	583	557	595	578.3333	810	714	686	736.6667
SEN3819	144	86	97.33333	1.528029	0.013429	1.351596	1.351595742	0.434663711	0.434664	0.057828	210	179	164	184.3333	265	308	272	281.6667
SEN3820	58	10	13.66667	1.057992	0.574734	-1.06961	1.069606508	0.097080148	-0.09708	0.753445	260	245	202	235.6667	242	254	252	249.3333
SEN3821	33	21	9.333333	1.077562	0.660296	-1.06169	1.061694939	0.08636929	-0.08637	0.816798	111	118	132	120.3333	144	134	111	129.6667
SEN3822	51	12	16	1.046198	0.4835	-1.08687	1.086868189	0.120176986	-0.12018	0.68865	354	363	322	346.3333	363	351	373	362.3333
SEN3828	176	70	114.3333	1.702869	0.000672	1.501812	1.501811552	0.586703794	0.586704	0.005601	183	175	130	162.6667	306	245	280	277
SEN3831	36	4	-20	-1.36364	0.001639	-1.54601	1.546012069	0.628551582	-0.62855	0.011298	80	85	60	75	60	49	56	55
SEN3832	63	8	-22.3333	-1.17268	0.031914	-1.33524	1.33524467	0.417104125	-0.4171	0.1122	131	183	141	151.6667	130	138	120	129.3333
SEN3833	159	22	34.33333	1.049615	0.520645	-1.08085	1.080851323	0.112168086	-0.11217	0.717174	763	703	610	692	769	694	716	726.3333
SEN3835	1341	654	632.6667	1.207205	0.654423	1.072632	1.072631593	0.101154652	0.101155	0.812951	3717	2995	2448	3053.333	3649	3789	3620	3686
SEN3842	20	3	4.666667	1.451613	0.452638	1.270414	1.270414262	0.345299014	0.345299	0.661709	12	9	10	10.33333	25	15	5	15
SEN3843	25	9	10.33333	1.215278	0.603585	1.07505	1.075049558	0.104403167	0.104403	0.77698	58	49	37	48	52	62	61	58.33333
SEN3844	5	0	-1.66667	-1.04762	0.28716	-1.19018	1.190177935	0.251177276	-0.25118	0.503523	35	40	35	36.66667	35	35	35	35
SEN3847	302	41	-154.667	-1.39523	0.003025	-1.61358	1.613576756	0.690262207	-0.69026	0.018286	402	575	661	546	387	359	428	391.3333
SEN3855	30	13	15.66667	1.208889	0.625199	1.066089	1.066089102	0.092328022	0.092328	0.788797	86	73	66	75	96	94	82	90.66667
SEN3856	25	14	6.333333	1.072519	0.65773	-1.05857	1.05857427	0.082122493	-0.08212	0.815446	89	97	76	87.33333	101	97	83	93.66667
SEN3857	382	130	-251.667	-1.55271	5.38E-05	-1.78238	1.782379265	0.833804354	-0.8338	0.000726	572	818	731	707	488	442	436	455.3333
SEN3858	501	333	382.6667	1.55352	0.011772	1.368263	1.368263471	0.452346061	0.452346	0.052369	806	632	636	691.3333	1133	969	1120	1074
SEN3861	4224	2879	2857.667	3.808978	0.003471	3.545445	3.545445439	1.825966895	1.825967	0.020329	2548	259	245	1017.333	4469	3138	4018	3875

Feature ID	Experiment - Range (original values)	Experiment - IQR (original values)	Experiment - Difference (original values)	Experiment - Fold Change (original values)	EDGE test: yccT NT vs WT NT , tagwise dispersions - P-value	EDGE test: yccT NT vs WT NT , tagwise dispersions - Fold change	yccT NT vs WT NT ABS|FC|	yccT NT vs WT NT Log2FC	yccT NT vs WT NT Log2FC +-	EDGE test: yccT NT vs WT NT , tagwise dispersions - FDR p-value correction	WT NT - WT.1.S22 - Expression values	WT NT - WT.2.S23 - Expression values	WT NT - WT.3.S24 - Expression values	WT NT - Means	yccT NT - yccT.1.S28 - Expression values	yccT NT - yccT.2.S29 - Expression values	yccT NT - yccT.3.S30 - Expression values	yccT NT - Means
SEN3870	8352	4433	5627	3.740422	0.000129	3.418062	3.418061527	1.773178367	1.773178	0.001452	4335	994	831	2053.333	9183	5427	8431	7680.333
SEN3887	78	16	12.33333	1.062925	0.574582	-1.07481	1.074808679	0.104079876	-0.10408	0.753445	202	200	186	196	237	159	229	208.3333
SEN3888	39	6	15.66667	1.096907	0.75822	-1.03697	1.036969349	0.052373251	-0.05237	0.880907	165	170	150	161.6667	179	164	189	177.3333
SEN3892	46	26	31.66667	1.44186	0.067338	1.263776	1.263776242	0.33774105	0.337741	0.193875	70	71	74	71.66667	116	97	97	103.3333
SEN3893	6	1	0.666667	1.032787	0.686589	-1.09938	1.099380588	0.136690911	-0.13669	0.833044	19	24	18	20.33333	20	20	23	21
SEN3894	160	15	-13.3333	-1.02438	0.243716	-1.17019	1.17019403	0.226747763	-0.22675	0.454314	485	645	551	560.3333	548	557	536	547
SEN3895	53	37	24.33333	1.132727	0.98861	-1.00385	1.003845324	0.005536991	-0.00554	1	175	214	161	183.3333	212	199	212	207.6667
SEN3896	27	8	2.666667	1.047619	0.582458	-1.09022	1.090215433	0.124613248	-0.12461	0.761737	51	63	54	56	72	45	59	58.66667
SEN3897	15	8	1.666667	1.027322	0.486174	-1.10712	1.107121617	0.14681371	-0.14681	0.691125	58	70	55	61	63	55	70	62.66667
SEN3898	40	7	-22	-1.06999	0.093316	-1.21833	1.218327112	0.284901538	-0.2849	0.239562	337	348	324	336.3333	317	308	318	314.3333
SEN3939	95	30	-23.6667	-1.19888	0.041819	-1.34663	1.346634027	0.429357825	-0.42936	0.137974	189	145	94	142.6667	135	117	105	119
SEN3940	18	8	-2.33333	-1.18421	0.39974	-1.31695	1.316954087	0.39720505	-0.39721	0.615134	22	19	4	15	9	17	12	12.66667
SEN3941	57	25	-11.6667	-1.05452	0.162903	-1.20342	1.203421501	0.267142038	-0.26714	0.345205	196	253	228	225.6667	209	230	203	214
SEN3943	12	5	8	1.347826	0.397766	1.186224	1.186224186	0.246376693	0.246377	0.61347	24	25	20	23	32	32	29	31
SEN3947	7	2	2	1.26087	0.791811	1.109143	1.109142644	0.149444919	0.149445	0.902424	11	4	8	7.666667	10	10	9	9.666667
SEN3968	85	43	35.33333	1.172358	0.766238	1.03562	1.035619794	0.050494445	0.050494	0.886011	245	198	172	205	257	223	241	240.3333
SEN3974	3	2	1.666667	1.625	0.671512	1.41362	1.413619957	0.499394313	0.499394	0.82303	4	2	2	2.666667	4	4	5	4.333333
SEN3975	147	59	79.33333	1.204467	0.71425	1.05266	1.052660323	0.074039976	0.07404	0.850307	350	388	426	388	447	497	458	467.3333
SEN3978	246	171	165.3333	1.297006	0.267294	1.144819	1.144819453	0.195120092	0.19512	0.479757	647	521	502	556.6667	726	692	748	722
SEN3979	42	13	13.66667	1.196172	0.680402	1.059675	1.059674654	0.08362139	0.083621	0.828031	92	67	50	69.66667	91	80	79	83.33333
SEN3980	23	8	4.333333	1.034031	0.427836	-1.09802	1.098017452	0.134900984	-0.1349	0.639913	139	127	116	127.3333	135	126	134	131.6667
SEN3981	80	38	26.33333	1.165966	0.833886	1.02909	1.029090074	0.041369264	0.041369	0.934012	206	127	143	158.6667	207	167	181	185
SEN3982	188	63	105.6667	1.56912	0.033318	1.377051	1.377051061	0.461582055	0.461582	0.11588	207	136	214	185.6667	270	324	280	291.3333
SEN3984	13	5	4	1.4	0.46512	1.234674	1.234673548	0.304129639	0.30413	0.673121	17	4	9	10	12	14	16	14
SEN3985	43	6	16.33333	1.398374	0.291635	1.224869	1.224868707	0.292627116	0.292627	0.508353	50	26	47	41	59	44	69	57.33333
SEN4008	14	3	-1.66667	-1.03937	0.296585	-1.18128	1.181283398	0.240355119	-0.24036	0.512736	42	46	44	44	36	41	50	42.33333
SEN4011	121	62	74	1.225839	0.519761	1.079686	1.079686304	0.110612208	0.110612	0.71666	365	313	305	327.6667	426	404	375	401.6667
SEN4021	85	37	28	1.125561	0.980881	-1.00485	1.00485424	0.006986245	-0.00699	1	272	210	187	223	268	238	247	251
SEN4022	28	14	13.66667	1.221622	0.622717	1.075615	1.075615281	0.105162157	0.105162	0.787112	58	72	55	61.66667	68	75	83	75.33333
SEN4026	15	7	11.33333	3.615385	0.000241	3.127603	3.12760274	1.645057277	1.645057	0.00235	5	5	3	4.333333	18	17	12	15.66667
SEN4027	29	5	13	1.672414	0.071969	1.471033	1.471032567	0.556829186	0.556829	0.202438	24	19	15	19.33333	44	33	20	32.33333
SEN4028	21	12	15	1.833333	0.013004	1.610344	1.610343895	0.687368814	0.687369	0.056491	18	21	15	18	36	33	30	33
SEN4029	14	4	-5.66667	-1.24638	0.06128	-1.413	1.412997915	0.498759336	-0.49876	0.180779	33	29	24	28.66667	27	19	23	23
SEN4030	433	240	74	1.020967	0.471266	-1.11735	1.1173499	0.160081039	-0.16008	0.677981	3491	3726	3371	3529.333	3804	3383	3623	3603.333
SEN4032	13	6	-0.33333	-1.00725	0.417246	-1.14733	1.147332428	0.198283458	-0.19828	0.63078	41	47	51	46.33333	39	47	52	46
SEN4037	61	9	19.66667	1.109057	0.891393	-1.01926	1.019262696	0.027525927	-0.02753	0.968079	205	192	144	180.3333	205	201	194	200

Feature ID	Experiment - Range (original values)	Experiment - IQR (original values)	Experiment - Difference (original values)	Experiment - Fold Change (original values)	EDGE test: yccT NT vs WT NT , tagwise dispersions - P-value	EDGE test: yccT NT vs WT NT , tagwise dispersions - Fold change	yccT NT vs WT NT ABS|FC|	yccT NT vs WT NT Log2FC	yccT NT vs WT NT Log2FC +-	EDGE test: yccT NT vs WT NT , tagwise dispersions - FDR p-value correction	WT NT - WT.1.S22 - Expression values	WT NT - WT.2.S23 - Expression values	WT NT - WT.3.S24 - Expression values	WT NT - Means	yccT NT - yccT.1.S28 - Expression values	yccT NT - yccT.2.S29 - Expression values	yccT NT - yccT.3.S30 - Expression values	yccT NT - Means
SEN4040	161	16	2.333333	1.003684	0.324557	-1.12712	1.127116391	0.172636503	-0.17264	0.539103	708	645	547	633.3333	632	646	629	635.6667
SEN4041	26	7	-8.66667	-1.18841	0.057616	-1.34397	1.34397214	0.426503232	-0.4265	0.172732	56	67	41	54.66667	43	50	45	46
SEN4042	20	4	-5.33333	-1.07547	0.136289	-1.22739	1.227394491	0.295599014	-0.2956	0.305615	72	75	81	76	71	61	80	70.66667
SEN4046	12	7	2.333333	1.051852	0.605844	-1.08482	1.084821799	0.117458074	-0.11746	0.777315	39	47	49	45	49	42	51	47.33333
SEN4051	1107	108	-738.667	-3.80506	1.44E-08	-4.46394	4.46393858	2.158317177	-2.15832	6.66E-07	384	1329	1293	1002	292	222	276	263.3333
SEN4052	421	45	-238.333	-4.70466	2.6E-08	-5.58637	5.586368327	2.481910698	-2.48191	1.15E-06	100	337	471	302.6667	88	55	50	64.33333
SEN4074	385	102	-90.3333	-1.0939	0.114137	-1.24091	1.240908972	0.311397289	-0.3114	0.273818	1207	924	1026	1052.333	1008	1056	822	962
SEN4080	51	27	-3.33333	-1.01592	0.276142	-1.16254	1.162537051	0.217276697	-0.21728	0.489434	180	227	231	212.6667	193	215	220	209.3333
SEN4081	44	6	18.66667	1.143959	0.934379	1.009517	1.009516575	0.013664599	0.013665	0.989776	141	135	113	129.6667	151	157	137	148.3333
SEN4082	34	19	21	1.875	0.004771	1.658489	1.658488574	0.729869073	0.729869	0.025812	35	21	16	24	40	45	50	45
SEN4084	8	2	3.333333	1.588235	0.381656	1.391536	1.391535733	0.476677956	0.476678	0.597859	8	6	3	5.666667	11	7	9	9
SEN4085	4	2	2	1.75	0.535508	1.516584	1.516583877	0.600825291	0.600825	0.726399	2	4	2	2.666667	6	2	6	4.666667
SEN4086	14	8	9.666667	1.644444	0.07992	1.443768	1.443768243	0.529839176	0.529839	0.217673	14	18	13	15	25	22	27	24.66667
SEN4087	26	7	8.333333	1.714286	0.171326	1.538123	1.538122782	0.621170673	0.621171	0.357245	20	8	7	11.66667	12	33	15	20
SEN4088	122	55	76.66667	1.387205	0.080968	1.224679	1.224678501	0.292403066	0.292403	0.219295	222	204	168	198	290	275	259	274.6667
SEN4089	212	81	103	1.342572	0.167057	1.190964	1.19096439	0.252130277	0.25213	0.351653	366	294	242	300.6667	375	382	454	403.6667
SEN4091	4	2	3	3.25	0.082948	2.712673	2.712673002	1.439715149	1.439715	0.22214	1	2	1	1.333333	3	5	5	4.333333
SEN4105	69	55	38.33333	1.344311	0.194242	1.192466	1.19246589	0.253947999	0.253948	0.388046	149	91	94	111.3333	151	160	138	149.6667
SEN4121	179	25	18.66667	1.047059	0.553898	-1.08149	1.081488957	0.113018934	-0.11302	0.739849	411	479	300	396.6667	456	386	404	415.3333
SEN4138	17	9	10	1.365854	0.318244	1.201474	1.201473636	0.264804991	0.264805	0.533336	26	32	24	27.33333	36	35	41	37.33333
SEN4156	6	3	4	1.218182	0.806318	1.071044	1.071044026	0.099017785	0.099018	0.911195	17	19	19	18.33333	22	23	22	22.33333
SEN4165	64	31	40	1.6	0.008754	1.41395	1.413950132	0.49973124	0.499731	0.041887	85	64	51	66.66667	110	95	115	106.6667
SEN4166	431	281	332.6667	2.115084	6.67E-07	1.875504	1.8755039	0.907278263	0.907278	1.93E-05	353	319	223	298.3333	654	600	639	631
SEN4181	259	161	120.3333	1.183249	0.747904	1.041533	1.041532797	0.058708269	0.058708	0.876079	771	589	610	656.6667	848	703	780	777
SEN4182	466	200	165.6667	1.557175	0.230885	1.398181	1.398180718	0.483550845	0.483551	0.437764	554	88	250	297.3333	499	440	450	463
SEN4189	36	11	-12.6667	-1.29688	0.016775	-1.4645	1.464502293	0.550410452	-0.55041	0.068255	72	54	40	55.33333	51	41	36	42.66667
SEN4190	91	30	-51.6667	-1.54196	5.62E-05	-1.7595	1.75949644	0.815162595	-0.81516	0.00075	123	181	137	147	103	93	90	95.33333
SEN4191	83	27	-43.3333	-1.69519	1.79E-05	-1.93064	1.930635457	0.94907578	-0.94908	0.000299	90	135	92	105.6667	63	52	72	62.33333
SEN4192	139	49	-83.3333	-1.53648	1.81E-05	-1.74652	1.746523649	0.804486177	-0.80449	0.000301	220	290	206	238.6667	157	151	158	155.3333
SEN4193	133	46	-80.6667	-1.39803	0.000127	-1.58771	1.587710844	0.666948191	-0.66695	0.001433	297	313	240	283.3333	234	194	180	202.6667
SEN4194	182	42	-85.3333	-1.3103	0.002728	-1.49214	1.492141559	0.57738441	-0.57738	0.016718	319	444	318	360.3333	276	262	287	275
SEN4195	175	2	-76.6667	-1.33873	0.00364	-1.53067	1.530670966	0.614164193	-0.61416	0.02103	238	380	291	303	238	236	205	226.3333
SEN4196	70	6	-35.3333	-1.20866	0.006637	-1.37153	1.371529299	0.455785442	-0.45579	0.033532	209	232	173	204.6667	176	162	170	169.3333
SEN4198	313	120	144	1.16308	0.826207	1.027945	1.027944856	0.039762873	0.039763	0.927991	1012	892	745	883	1053	970	1058	1027
SEN4199	118	23	3	1.008789	0.350196	-1.13651	1.136508672	0.184608693	-0.18461	0.5657	352	277	395	341.3333	360	329	344	344.3333
SEN4200	178	6	72.33333	1.162182	0.896421	1.018046	1.018045827	0.025802505	0.025803	0.970448	480	384	474	446	513	480	562	518.3333

Feature ID	Experiment - Range (original values)	Experiment - IQR (original values)	Experiment - Difference (original values)	Experiment - Fold Change (original values)	EDGE test: yccT NT vs WT NT , tagwise dispersions - P-value	EDGE test: yccT NT vs WT NT , tagwise dispersions - Fold change	yccT NT vs WT NT ABS|FC|	yccT NT vs WT NT Log2FC	yccT NT vs WT NT Log2FC +-	EDGE test: yccT NT vs WT NT , tagwise dispersions - FDR p-value correction	WT NT - WT.1.S22 - Expression values	WT NT - WT.2.S23 - Expression values	WT NT - WT.3.S24 - Expression values	WT NT - Means	yccT NT - yccT.1.S28 - Expression values	yccT NT - yccT.2.S29 - Expression values	yccT NT - yccT.3.S30 - Expression values	yccT NT - Means
SEN4203	48	7	-28.6667	-2.32308	4.58E-05	-2.69058	2.69058366	1.427919166	-1.42792	0.000644	27	58	66	50.33333	27	18	20	21.66667
SEN4208	178	93	72.33333	1.057743	0.546419	-1.08211	1.082108858	0.113845638	-0.11385	0.734359	1238	1189	1331	1252.667	1367	1253	1355	1325
SEN4213	8617	1473	4104.333	1.756095	0.123437	1.537981	1.537980951	0.621037635	0.621038	0.287506	7879	1769	6637	5428.333	10102	8110	10386	9532.667
SEN4214	37660	10970	19347.33	1.853082	0.073236	1.632677	1.632676795	0.707239223	0.707239	0.204609	34534	8099	25405	22679.33	43946	36375	45759	42026.67
SEN4216	61173	14035	29994	1.746714	0.112192	1.533349	1.533348718	0.616685836	0.616686	0.27047	58276	14893	47335	40168	73050	61370	76066	70162
SEN4217	41899	11054	21793	1.786609	0.069681	1.569396	1.569396478	0.650209868	0.65021	0.198302	39422	12276	31417	27705	51848	42471	54175	49498
SEN4218	71323	19763	36784.67	1.678617	0.107704	1.472419	1.472419319	0.558188584	0.558189	0.263665	73846	25389	63381	54205.33	93114	83144	96712	90990
SEN4231	119	46	-73.3333	-1.27466	0.003102	-1.45848	1.458475663	0.544461313	-0.54446	0.018628	318	343	360	340.3333	288	272	241	267
SEN4239A	50	14	7.666667	1.044146	0.514841	-1.08617	1.08617483	0.119256338	-0.11926	0.715129	171	200	150	173.6667	172	185	187	181.3333
SEN4241	136	56	69.66667	1.402697	0.096547	1.247113	1.247113323	0.318592566	0.318593	0.245426	214	162	143	173	218	279	231	242.6667
SEN4242	445	294	293.6667	1.657463	0.004567	1.474739	1.474739357	0.560459998	0.56046	0.024981	575	395	370	446.6667	717	815	689	740.3333
SEN4243	600	447	450	1.758853	0.000815	1.560497	1.56049671	0.642005316	0.642005	0.006522	739	531	509	593	1042	1109	978	1043
SEN4243A	44	38	30	1.666667	0.017762	1.481414	1.481414264	0.566975134	0.566975	0.071291	68	34	33	45	72	77	76	75
SEN4244	39	29	14.66667	1.109726	0.857282	-1.0249	1.024899223	0.035482059	-0.03548	0.948917	153	124	124	133.6667	163	124	158	148.3333
SEN4246	29	14	14	1.347107	0.253276	1.186843	1.186842993	0.247129095	0.247129	0.464707	51	37	33	40.33333	62	45	56	54.33333
SEN4247	8703	6650	6317.333	2.742072	0.000204	2.500803	2.500802559	1.32239116	1.322391	0.002069	6350	2883	1646	3626.333	9533	9949	10349	9943.667
SEN4248	12	3	7	2.235294	0.029531	1.953648	1.953648279	0.966170758	0.966171	0.106212	6	8	3	5.666667	9	15	14	12.66667
SEN4249	68	14	29.33333	1.30137	0.318182	1.150155	1.15015496	0.201828248	0.201828	0.533336	98	112	82	97.33333	122	150	108	126.6667
SEN4250	8	6	-2	-1.2069	0.300685	-1.3601	1.360102594	0.44371548	-0.44372	0.51581	14	14	7	11.66667	6	13	10	9.666667
SEN4251	12	4	6.333333	1.513514	0.212855	1.332747	1.332746997	0.414402931	0.414403	0.413485	16	13	8	12.33333	17	19	20	18.66667
SEN4252	18	6	10.66667	1.25	0.528624	1.100914	1.100914324	0.138702199	0.138702	0.722496	44	47	37	42.66667	55	55	50	53.33333
SEN4253	34	16	18	1.244344	0.487997	1.097317	1.09731718	0.133980598	0.133981	0.692606	74	86	61	73.66667	90	95	90	91.66667
SEN4254	44	8	8	1.052288	0.553116	-1.07612	1.07612215	0.105841847	-0.10584	0.739323	178	147	134	153	155	152	176	161
SEN4262	216	24	73	1.141564	0.933891	1.011147	1.011146513	0.015992056	0.015992	0.989544	564	589	394	515.6667	610	588	568	588.6667
SEN4263	32	3	-3.66667	-1.05851	0.264878	-1.20617	1.206165842	0.270428284	-0.27043	0.476522	57	82	60	66.33333	58	50	80	62.66667
SEN4264	32	14	22.33333	1.705263	0.009745	1.497128	1.49712757	0.582197159	0.582197	0.045499	35	29	31	31.66667	61	45	56	54
SEN4265	247	155	163.3333	1.556818	0.00986	1.381534	1.381534339	0.466271422	0.466271	0.045847	366	283	231	293.3333	454	438	478	456.6667
SEN4266	299	170	201.6667	1.465385	0.034758	1.299277	1.299276795	0.377708812	0.377709	0.119762	506	451	343	433.3333	642	642	621	635
SEN4269	8	1	2.666667	9	0.024924	6.128078	6.128077647	2.615434577	2.615435	0.092419	1	0	0	0.333333	1	0	8	3
SEN4270	616	516	503	1.981144	9.05E-06	1.755649	1.755649344	0.812004724	0.812005	0.000171	625	480	433	512.6667	996	1002	1049	1015.667
SEN4271	333	237	249.6667	2.053446	1.59E-05	1.821787	1.821787464	0.86535466	0.865355	0.000271	323	195	193	237	502	432	526	486.6667
SEN4279	83	44	27	1.198044	0.669832	1.063279	1.063279206	0.088520483	0.08852	0.822256	187	104	118	136.3333	156	162	172	163.3333
SEN4280	162	105	81.33333	1.36858	0.156147	1.222668	1.222668475	0.290033271	0.290033	0.333761	307	202	153	220.6667	308	315	283	302
SEN4281	7	2	2.333333	1.636364	0.478929	1.425568	1.425568328	0.51153719	0.511537	0.684774	5	0	6	3.666667	7	7	4	6
SEN4283	217	173	134	1.250467	0.420096	1.107095	1.107095412	0.146779563	0.14678	0.632714	657	486	462	535	679	659	669	669
SEN4290	136	41	71.33333	1.262255	0.385662	1.111036	1.11103576	0.151905252	0.151905	0.602364	303	262	251	272	387	346	297	343.3333

Feature ID	Experiment - Range (original values)	Experiment - IQR (original values)	Experiment - Difference (original values)	Experiment - Fold Change (original values)	EDGE test: yccT NT vs WT NT , tagwise dispersions - P-value	EDGE test: yccT NT vs WT NT , tagwise dispersions - Fold change	yccT NT vs WT NT ABS|FC|	yccT NT vs WT NT Log2FC	yccT NT vs WT NT Log2FC +-	EDGE test: yccT NT vs WT NT , tagwise dispersions - FDR p-value correction	WT NT - WT.1.S22 - Expression values	WT NT - WT.2.S23 - Expression values	WT NT - WT.3.S24 - Expression values	WT NT - Means	yccT NT - yccT.1.S28 - Expression values	yccT NT - yccT.2.S29 - Expression values	yccT NT - yccT.3.S30 - Expression values	yccT NT - Means
SEN4291	165	109	111.3333	1.4	0.090857	1.235977	1.235976982	0.305651875	0.305652	0.235984	338	235	262	278.3333	400	398	371	389.6667
SEN4292	99	49	56.33333	1.147084	0.941817	1.009857	1.009857048	0.014151084	0.014151	0.995751	405	370	374	383	426	469	423	439.3333
SEN4293	161	109	86.66667	1.229277	0.517628	1.086889	1.086888773	0.12020431	0.120204	0.715578	457	329	348	378	468	490	436	464.6667
SEN4294	171	100	53.66667	1.149907	0.872413	1.021137	1.021137199	0.030176718	0.030177	0.958007	463	319	292	358	425	419	391	411.6667
SEN4299	3737	1723	1773.333	1.293097	0.401538	1.152658	1.152658281	0.204964872	0.204965	0.616499	7816	5954	4381	6050.333	8118	7676	7677	7823.667
SEN4300	1092	362	-471.333	-1.68046	0.005202	-1.89441	1.89441163	0.921749843	-0.92175	0.027592	1561	620	1311	1164	627	982	469	692.6667
SEN4301	2723	562	-293.667	-1.18586	0.362453	-1.30854	1.308537719	0.387955511	-0.38796	0.578958	3381	658	1582	1873.667	1465	2255	1020	1580
SEN4302	5300	934	12.33333	1.004008	0.806549	-1.08491	1.084914017	0.117580709	-0.11758	0.911223	6273	973	1986	3077.333	2912	4379	1978	3089.667
SEN4303	9660	3812	1111	1.242895	0.714865	1.155766	1.155766112	0.208849474	0.208849	0.850783	10708	1048	1966	4574	5778	7408	3869	5685
SEN4304	14872	7298	2465	1.391125	0.555941	1.304398	1.304398192	0.383384347	0.383384	0.741429	15958	1086	1863	6302.333	9161	10728	6413	8767.333
SEN4305	19766	13161	6073	1.781328	0.309152	1.677445	1.677445097	0.746265548	0.746266	0.524245	20854	1088	1376	7772.667	15677	14537	11323	13845.67
SEN4310	111	26	37.66667	1.088767	0.690667	-1.05236	1.052357198	0.073624477	-0.07362	0.835588	410	427	436	424.3333	500	389	497	462
SEN4314	181	109	150	1.48913	0.031781	1.309616	1.309615863	0.389143702	0.389144	0.111941	309	304	307	306.6667	416	485	469	456.6667
SEN4316	367	154	223.3333	1.577586	0.009424	1.396175	1.396175364	0.48148016	0.48148	0.044518	483	372	305	386.6667	672	526	632	610
SEN4317	64	4	19	1.251101	0.49055	1.108194	1.10819374	0.148210123	0.14821	0.695561	87	84	56	75.66667	80	84	120	94.66667
SEN4331A	71	13	7.666667	1.042672	0.502935	-1.08842	1.088422231	0.12223833	-0.12224	0.704228	174	218	147	179.6667	187	175	200	187.3333
SEN4351	38	15	20.66667	1.521008	0.056466	1.344225	1.34422463	0.426774243	0.426774	0.17009	53	38	28	39.66667	62	53	66	60.33333
SEN4352	242	189	179.3333	1.835404	0.000184	1.623676	1.623675695	0.699263504	0.699264	0.001935	279	177	188	214.6667	419	377	386	394
SEN4353	265	167	173	1.746763	0.002326	1.562169	1.562169233	0.643550752	0.643551	0.014579	321	227	147	231.6667	412	394	408	404.6667
seqA	369	155	87.33333	1.035248	0.528803	-1.09789	1.097887618	0.134730385	-0.13473	0.722518	2548	2600	2285	2477.667	2654	2598	2443	2565
serA	961	492	633.6667	1.628222	0.007799	1.445492	1.445491547	0.531560172	0.53156	0.038253	1278	977	771	1008.667	1726	1469	1732	1642.333
serB	204	109	137.6667	1.291461	0.332997	1.134063	1.134062829	0.18150057	0.181501	0.546891	511	416	490	472.3333	599	620	611	610
serC	603	284	436.6667	1.208566	0.691244	1.059845	1.059845131	0.083853467	0.083853	0.835588	2100	2146	2035	2093.667	2569	2384	2638	2530.333
serS	4216	1656	-1111.33	-1.05999	0.270711	-1.20206	1.202059682	0.265508527	-0.26551	0.483873	20993	21067	16851	19637	19442	17786	18349	18525.67
setB	68	12	39	1.493671	0.04128	1.31174	1.311740279	0.391482098	0.391482	0.136501	82	82	73	79	119	94	141	118
sfbA	521	282	280	1.331492	0.208568	1.183233	1.183232751	0.242733891	0.242734	0.409104	1083	801	650	844.6667	1171	1059	1144	1124.667
sfbB	329	98	202.6667	1.44904	0.04995	1.277112	1.277112127	0.352885195	0.352885	0.156305	506	468	380	451.3333	709	566	687	654
sfbC	123	11	53	1.127608	0.948633	-1.00833	1.008328866	0.01196625	-0.01197	0.999617	440	432	374	415.3333	497	429	479	468.3333
sfcA	1304	26	-668	-1.32912	0.01414	-1.52738	1.527377911	0.611057065	-0.61106	0.060359	2046	3255	2792	2697.667	2072	1951	2066	2029.667
sfsA	412	215	230.3333	1.282502	0.321163	1.131218	1.131217795	0.17787672	0.177877	0.536199	941	784	721	815.3333	1133	999	1005	1045.667
sgaB	161	141	141.6667	1.745614	0.000221	1.537365	1.537365467	0.620460168	0.62046	0.002177	212	176	182	190	335	337	323	331.6667
sgaE	168	94	108.6667	1.302974	0.263433	1.145708	1.145708158	0.196239598	0.19624	0.475513	406	321	349	358.6667	489	443	470	467.3333
sgaH	271	196	214	1.493846	0.024472	1.311156	1.311156152	0.390839513	0.39084	0.091299	460	415	425	433.3333	686	621	635	647.3333
sgaT	359	260	259	1.561822	0.01016	1.384461	1.384460741	0.469324144	0.469324	0.046796	556	437	390	461	714	749	697	720
sgaU	293	166	209	1.421371	0.109967	1.239375	1.239375167	0.309612967	0.309613	0.267141	486	448	554	496	722	652	741	705
sgbE	73	19	41.66667	1.287356	0.305647	1.131313	1.131313218	0.177998413	0.177998	0.521303	145	163	127	145	200	164	196	186.6667

Feature ID	Experiment - Range (original values)	Experiment - IQR (original values)	Experiment - Difference (original values)	Experiment - Fold Change (original values)	EDGE test: yccT NT vs WT NT , tagwise dispersions - P-value	EDGE test: yccT NT vs WT NT , tagwise dispersions - Fold change	yccT NT vs WT NT ABS|FC|	yccT NT vs WT NT Log2FC	yccT NT vs WT NT Log2FC +-	EDGE test: yccT NT vs WT NT , tagwise dispersions - FDR p-value correction	WT NT - WT.1.S22 - Expression values	WT NT - WT.2.S23 - Expression values	WT NT - WT.3.S24 - Expression values	WT NT - Means	yccT NT - yccT.1.S28 - Expression values	yccT NT - yccT.2.S29 - Expression values	yccT NT - yccT.3.S30 - Expression values	yccT NT - Means
sgbH	16	7	7	1.6	0.159875	1.405872	1.405871944	0.49146519	0.491465	0.340415	11	18	6	11.66667	22	16	18	18.66667
sgbU	15	8	6	1.36	0.418189	1.198175	1.198175388	0.260839105	0.260839	0.631774	24	12	14	16.66667	27	22	19	22.66667
shdA	188	138	37.33333	1.053614	0.543509	-1.07839	1.078386577	0.108874444	-0.10887	0.732623	791	681	617	696.3333	805	629	767	733.6667
sicA	34	8	10.33333	1.234848	0.603696	1.089401	1.089400739	0.123534751	0.123535	0.77698	49	51	32	44	54	43	66	54.33333
sicP	10	2	1.666667	1.138889	1	1.005154	1.005154184	0.007416819	0.007417	1	16	13	7	12	13	11	17	13.66667
sifA	46	18	24	1.32287	0.256398	1.165256	1.165256338	0.22064736	0.220647	0.468495	91	59	73	74.33333	105	99	91	98.33333
sifB	12	7	6.666667	2.052632	0.073057	1.78315	1.783150229	0.834428254	0.834428	0.204366	4	4	11	6.333333	16	14	9	13
sinH	41	7	21	1.851351	0.006086	1.629248	1.629248016	0.704206238	0.704206	0.031394	29	30	15	24.66667	56	36	45	45.66667
sinI	48	19	25.33333	1.370732	0.147345	1.214529	1.214528665	0.28039654	0.280397	0.321896	86	72	47	68.33333	95	91	95	93.66667
sinR	68	41	48	1.770053	0.000787	1.565268	1.565268039	0.646409728	0.64641	0.006315	77	58	52	62.33333	99	112	120	110.3333
sipA	148	58	93.66667	1.700748	0.000923	1.503745	1.503745301	0.588560229	0.58856	0.007216	156	143	102	133.6667	231	201	250	227.3333
sipB	177	56	52.33333	1.176207	0.751627	1.04364	1.043640177	0.061624389	0.061624	0.878286	359	327	205	297	382	303	363	349.3333
sipC	228	35	92	1.362681	0.191552	1.207445	1.207445017	0.271957496	0.271957	0.384405	281	316	164	253.6667	392	299	346	345.6667
sipD	69	19	41	1.773585	0.001344	1.559103	1.559102528	0.640715804	0.640716	0.009591	60	56	43	53	112	75	95	94
sirC	31	4	-6.33333	-1.076	0.139141	-1.22579	1.225792966	0.293715332	-0.29372	0.310128	79	107	83	89.66667	91	83	76	83.33333
sitA	47	19	27.66667	1.196217	0.684633	1.051731	1.051731392	0.072766293	0.072766	0.83181	155	131	137	141	172	156	178	168.6667
sitB	17	5	10.66667	1.275862	0.483027	1.122982	1.122982271	0.167335151	0.167335	0.68864	42	37	37	38.66667	54	54	40	49.33333
sitC	28	20	22.33333	1.848101	0.002739	1.624522	1.624522323	0.700015569	0.700016	0.016743	31	24	24	26.33333	52	44	50	48.66667
sitD	64	36	42.33333	1.693989	0.002216	1.499053	1.499053038	0.584051428	0.584051	0.014025	74	62	47	61	98	111	101	103.3333
sixA	1354	603	729.3333	1.201492	0.727318	1.055942	1.055942388	0.078531123	0.078531	0.860317	4082	3298	3479	3619.667	4652	4039	4356	4349
slp	134	26	58.33333	1.118483	0.85245	-1.02429	1.024290162	0.034624462	-0.03462	0.94643	453	504	520	492.3333	587	535	530	550.6667
slpA	777	407	327.6667	1.151838	0.896957	1.019207	1.019207318	0.027447541	0.027448	0.97046	2546	2132	1796	2158	2573	2345	2539	2485.667
slrB	616	101	-436	-5.28852	2.97E-11	-6.17398	6.173981879	2.626201248	-2.6262	2.16E-09	201	700	712	537.6667	100	109	96	101.6667
slrP	518	311	339.3333	1.477262	0.032206	1.308591	1.308591324	0.388014611	0.388015	0.112812	858	687	588	711	1106	1047	998	1050.333
slsA	284	227	203.3333	2.788856	9.76E-05	2.5352	2.535200408	1.342099797	1.3421	0.00116	218	68	55	113.6667	339	317	295	317
slt	497	140	93.66667	1.046966	0.564344	-1.08275	1.08275313	0.114704343	-0.1147	0.745911	2281	1918	1784	1994.333	2194	2012	2058	2088
slyA	371	45	109.6667	1.059077	0.596211	-1.07639	1.076392564	0.106204329	-0.1062	0.771389	2003	1686	1880	1856.333	2057	1916	1925	1966
slyB	741	250	73.33333	1.026862	0.524261	-1.10156	1.101557327	0.139544578	-0.13954	0.719276	3091	2749	2350	2730	2916	2872	2622	2803.333
slyD	3721	1766	-2775	-1.4459	0.003213	-1.66079	1.660789439	0.731869174	-0.73187	0.019212	7857	9351	9787	8998.333	6513	6091	6066	6223.333
slyX	59	44	-4	-1.00842	0.262298	-1.1517	1.151696394	0.203760449	-0.20376	0.474024	491	444	502	479	503	475	447	475
smf	571	428	308.6667	1.35547	0.200832	1.207318	1.207317654	0.27180531	0.271805	0.39816	1184	691	730	868.3333	1158	1262	1111	1177
smg	5924	4772	2681	1.252353	0.545087	1.115314	1.11531431	0.157450337	0.15745	0.733458	14556	8684	8632	10624	13730	13456	12729	13305
smp	47	38	38.66667	1.233871	0.478947	1.085391	1.08539134	0.118215303	0.118215	0.684774	174	164	158	165.3333	205	202	205	204
smpA	747	272	314.3333	1.118901	0.925903	-1.01414	1.014143322	0.020261553	-0.02026	0.985354	2930	2617	2384	2643.667	3131	2889	2854	2958
smpB	660	280	362	1.194345	0.7102	1.052766	1.052765908	0.074184675	0.074185	0.847948	2076	1818	1694	1862.667	2354	2222	2098	2224.667
smtA	151	118	8	1.006159	0.337024	-1.13171	1.13170804	0.178501817	-0.1785	0.550703	1332	1353	1212	1299	1363	1220	1338	1307

Feature ID	Experiment - Range (original values)	Experiment - IQR (original values)	Experiment - Difference (original values)	Experiment - Fold Change (original values)	EDGE test: yccT NT vs WT NT , tagwise dispersions - P-value	EDGE test: yccT NT vs WT NT , tagwise dispersions - Fold change	yccT NT vs WT NT ABS|FC|	yccT NT vs WT NT Log2FC	yccT NT vs WT NT Log2FC +-	EDGE test: yccT NT vs WT NT , tagwise dispersions - FDR p-value correction	WT NT - WT.1.S22 - Expression values	WT NT - WT.2.S23 - Expression values	WT NT - WT.3.S24 - Expression values	WT NT - Means	yccT NT - yccT.1.S28 - Expression values	yccT NT - yccT.2.S29 - Expression values	yccT NT - yccT.3.S30 - Expression values	yccT NT - Means
smvA	115	58	67.33333	1.372694	0.111478	1.213484	1.213483604	0.279154616	0.279155	0.269434	225	167	150	180.6667	265	225	254	248
sodA	9684	1929	-4729.33	-10.4776	2.44E-15	-11.8198	11.8197701	3.56313007	-3.56313	3.61E-13	3089	10156	2440	5228.333	514	511	472	499
sodB	11145	4065	2789.667	1.114886	0.949263	-1.01117	1.011174615	0.016032151	-0.01603	0.99982	30987	22017	19842	24282	29338	26082	25795	27071.67
sodC	2619	1167	1071.667	1.19255	0.7711	1.049577	1.049577463	0.069808648	0.069809	0.887927	6587	4720	5390	5565.667	7339	6557	6016	6637.333
sodCb	176	87	-99.6667	-1.14972	0.027419	-1.31288	1.312877835	0.392732677	-0.39273	0.100002	772	782	742	765.3333	736	606	655	665.6667
sohB	274	58	90.33333	1.087363	0.750598	-1.04072	1.040719543	0.057581338	-0.05758	0.877546	1160	1056	886	1034	1146	1114	1113	1124.333
solA	1223	919	822.6667	1.432224	0.110106	1.272	1.271999855	0.347098507	0.347099	0.26733	2384	1787	1539	1903.333	2762	2706	2710	2726
sopA	723	617	558.6667	2.093281	3.28E-05	1.872814	1.872813622	0.905207333	0.905207	0.000495	735	436	362	511	1071	1053	1085	1069.667
sopB	165	67	97	1.697842	0.005854	1.516265	1.516264984	0.600521902	0.600522	0.030341	178	160	79	139	237	227	244	236
sopD	23	19	-8.66667	-1.09962	0.08297	-1.24883	1.248832506	0.320579995	-0.32058	0.22214	99	100	88	95.66667	80	79	102	87
sopE2	31	11	18.66667	2.244444	0.001049	1.978985	1.978985491	0.984761036	0.984761	0.007931	20	16	9	15	27	34	40	33.66667
soxR	24	5	-4	-1.06897	0.188752	-1.21259	1.212588953	0.278090584	-0.27809	0.37964	75	54	57	62	59	51	64	58
soxS	86	32	48	1.389189	0.093246	1.228842	1.228841626	0.297298992	0.297299	0.239522	138	127	105	123.3333	164	191	159	171.3333
spaO	17	4	-10	-1.6	0.003536	-1.81013	1.810133739	0.856096293	-0.8561	0.020624	27	32	21	26.66667	18	15	17	16.66667
spaP	9	1	-3.33333	-1.2381	0.153345	-1.40424	1.404240214	0.489789749	-0.48979	0.330483	16	20	16	17.33333	16	11	15	14
spaQ	8	1	3	1.818182	0.301613	1.577946	1.577946214	0.65804803	0.658048	0.516769	1	5	5	3.666667	7	4	9	6.666667
spaR	5	1	1.333333	1.16	1	1.021536	1.021536455	0.030740689	0.030741	1	10	8	7	8.333333	9	8	12	9.666667
spaS	3	1	0	-1	0.882402	-1.13386	1.13385789	0.181239834	-0.18124	0.962777	8	6	7	7	9	6	6	7
speA	449	299	101.3333	1.041026	0.538157	-1.09968	1.099680149	0.137083964	-0.13708	0.727892	2250	2699	2461	2470	2693	2361	2660	2571.333
speB	2166	535	-1032.33	-1.38679	0.006809	-1.58254	1.582539015	0.662241067	-0.66224	0.03422	3140	4713	3251	3701.333	2855	2547	2605	2669
speC	358	159	237.6667	1.440123	0.052285	1.272284	1.272284014	0.347420761	0.347421	0.160996	584	596	440	540	798	743	792	777.6667
speD	169	21	-9	-1.00975	0.264036	-1.15331	1.153313114	0.205784245	-0.20578	0.475665	889	1022	885	932	1010	853	906	923
speE	101	21	-34.3333	-1.05362	0.147375	-1.20213	1.202132068	0.265595401	-0.2656	0.321896	634	729	661	674.6667	638	628	655	640.3333
speF	18186	2334	-10971	-5.99818	2.5E-10	-7.11414	7.114136157	2.830688586	-2.83069	1.71E-08	4713	14895	19890	13166	2502	1704	2379	2195
speG	667	353	366.3333	1.179253	0.778518	1.041789	1.041788706	0.059062703	0.059063	0.892946	2330	1977	1824	2043.667	2481	2491	2258	2410
spoT	1309	457	-743.333	-1.16245	0.086199	-1.33553	1.335534278	0.417417005	-0.41742	0.228418	4597	5679	5681	5319	4906	4372	4449	4575.667
spoU	246	112	-88.6667	-1.13387	0.059372	-1.30316	1.303155873	0.382009658	-0.38201	0.176679	617	856	780	751	729	610	648	662.3333
sppA	206	83	67.33333	1.066821	0.593321	-1.07443	1.074429582	0.103570932	-0.10357	0.76939	919	1061	1043	1007.667	1122	978	1125	1075
spr	1193	689	807.6667	1.184259	0.805699	1.040024	1.04002399	0.056616807	0.056617	0.911195	4674	4044	4432	4383.333	5121	5215	5237	5191
sprB	105	67	68	2.127072	0.001344	1.901806	1.901806305	0.927370318	0.92737	0.009591	100	32	49	60.33333	116	137	132	128.3333
sptP	45	9	0.666667	1.005181	0.322926	-1.12537	1.125365973	0.170394248	-0.17039	0.538129	149	133	104	128.6667	129	124	135	129.3333
spy	599	455	388	2.032831	0.003452	1.843537	1.843537073	0.882476429	0.882476	0.020257	657	277	193	375.6667	792	732	767	763.6667
srfA	200	84	-151.333	-1.50557	6.01E-05	-1.72508	1.725082994	0.786665772	-0.78667	0.000795	387	476	489	450.6667	289	303	306	299.3333
srfB	862	590	-691.667	-2.00679	5.73E-10	-2.28654	2.286539744	1.193165996	-1.19317	3.43E-08	1284	1531	1321	1378.667	669	698	694	687
srfC	845	240	-495.667	-1.5481	3.91E-05	-1.75597	1.755965541	0.812264534	-0.81226	0.00057	1336	1696	1168	1400	928	934	851	904.3333
srlA	1349	203	-905.333	-7.22936	9.85E-13	-8.42656	8.426559737	3.074943751	-3.07494	9.09E-11	343	1487	1322	1050.667	138	158	140	145.3333

Feature ID	Experiment - Range (original values)	Experiment - IQR (original values)	Experiment - Difference (original values)	Experiment - Fold Change (original values)	EDGE test: yccT NT vs WT NT , tagwise dispersions - P-value	EDGE test: yccT NT vs WT NT , tagwise dispersions - Fold change	yccT NT vs WT NT ABS|FC|	yccT NT vs WT NT Log2FC	yccT NT vs WT NT Log2FC +-	EDGE test: yccT NT vs WT NT , tagwise dispersions - FDR p-value correction	WT NT - WT.1.S22 - Expression values	WT NT - WT.2.S23 - Expression values	WT NT - WT.3.S24 - Expression values	WT NT - Means	yccT NT - yccT.1.S28 - Expression values	yccT NT - yccT.2.S29 - Expression values	yccT NT - yccT.3.S30 - Expression values	yccT NT - Means
srlD	2298	407	-1558	-3.9601	4.87E-10	-4.631	4.630995364	2.211322313	-2.21132	3.03E-08	933	2499	2821	2084.333	523	530	526	526.3333
srlE	1896	286	-1327	-5.98872	1.42E-11	-6.99489	6.994892708	2.806301929	-2.8063	1.11E-09	549	2148	2082	1593	252	283	263	266
srlR	2274	345	-1338	-1.63163	0.000726	-1.87558	1.875579702	0.907336571	-0.90734	0.005956	2447	4374	3548	3456.333	2153	2100	2102	2118.333
srmB	716	344	380.3333	1.179374	0.795819	1.038122	1.038121559	0.053975387	0.053975	0.904869	2337	2071	1953	2120.333	2669	2415	2418	2500.667
ssaB	10	1	4	2.333333	0.074377	2.023165	2.023164949	1.016613948	1.016614	0.206495	4	5	0	3	4	10	7	7
ssaC	17	4	10	2.071429	0.020549	1.810395	1.810395353	0.856304786	0.856305	0.079681	11	7	10	9.333333	24	14	20	19.33333
ssaD	25	15	19	2.78125	3.62E-05	2.434008	2.434008162	1.283334006	1.283334	0.000535	11	14	7	10.66667	32	26	31	29.66667
ssaE	11	5	7	2.5	0.018213	2.169027	2.169027037	1.117048036	1.117048	0.07277	5	3	6	4.666667	14	10	11	11.66667
ssaG	12	6	7.666667	2.916667	0.006855	2.522838	2.52283819	1.335047677	1.335048	0.034402	6	1	5	4	13	11	11	11.66667
ssaH	22	3	8.333333	2.470588	0.040026	2.123026	2.123026361	1.086122285	1.086122	0.13325	5	4	8	5.666667	26	8	8	14
ssaI	5	1	0.333333	1.066667	1	-1.06521	1.065210032	0.091137921	-0.09114	1	5	4	6	5	8	3	5	5.333333
ssaJ	26	7	13.66667	2.518519	0.001666	2.200024	2.200024294	1.137519455	1.137519	0.011427	12	9	6	9	32	16	20	22.66667
ssaK	11	6	7.666667	2.210526	0.028549	1.928582	1.928582083	0.94754055	0.947541	0.103434	8	4	7	6.333333	15	13	14	14
ssaL	67	34	46	2.104	0.000198	1.855623	1.855623055	0.891903676	0.891904	0.002027	60	30	35	41.66667	97	69	97	87.66667
ssaM	10	1	4	1.857143	0.221306	1.618962	1.618961905	0.695069039	0.695069	0.425241	6	5	3	4.666667	13	6	7	8.666667
ssaN	90	46	61	1.642105	0.003576	1.452233	1.452232663	0.538272607	0.538273	0.020792	101	110	74	95	147	164	157	156
ssaO	11	4	6	1.409091	0.377137	1.240237	1.240236862	0.310615675	0.310616	0.594928	16	14	14	14.66667	18	25	19	20.66667
ssaP	31	6	17	2.378378	0.001646	2.092361	2.092360878	1.065131701	1.065132	0.011315	15	18	4	12.33333	32	21	35	29.33333
ssaQ	77	33	53	1.913793	0.000101	1.685924	1.685923931	0.753539444	0.753539	0.001189	71	57	46	58	123	90	120	111
ssaR	26	17	18.33333	1.887097	0.004557	1.659506	1.659505765	0.730753642	0.730754	0.024981	27	16	19	20.66667	39	36	42	39
ssaS	4	1	1.333333	1.307692	0.860204	1.148807	1.148807012	0.20013646	0.200136	0.950937	5	5	3	4.333333	4	7	6	5.666667
ssaT	2	0	1	1.125	1	-1.00991	1.009909382	0.014225847	-0.01423	1	8	8	8	8	9	8	10	9
ssaU	77	42	35.66667	1.781022	0.053904	1.620107	1.620106699	0.696088831	0.696089	0.164497	85	33	19	45.66667	75	96	73	81.33333
ssaV	64	32	47.33333	2.494737	4.7E-07	2.188428	2.188427713	1.12989473	1.129895	1.42E-05	39	25	31	31.66667	89	63	85	79
ssb	710	184	-389.333	-1.15385	0.07862	-1.32089	1.32089303	0.401513638	-0.40151	0.215307	2640	3152	2968	2920	2667	2483	2442	2530.667
sscA	26	14	19.66667	2.134615	0.000831	1.873992	1.873991699	0.906114563	0.906115	0.006608	20	18	14	17.33333	40	32	39	37
sscB	18	5	10.66667	1.727273	0.05617	1.514554	1.514553588	0.598892624	0.598893	0.169544	15	16	13	14.66667	31	20	25	25.33333
sseA	890	392	-507.667	-1.14612	0.082964	-1.30466	1.304661961	0.383676052	-0.38368	0.22214	4237	3906	3803	3982	3665	3347	3411	3474.333
sseAb	18	9	12	2.44	0.002747	2.134416	2.134416265	1.093841565	1.093842	0.016768	7	10	8	8.333333	17	25	19	20.33333
sseB	249	63	-104	-1.15058	0.032432	-1.31678	1.316779346	0.397013612	-0.39701	0.113245	734	872	778	794.6667	756	623	693	690.6667
sseBb	26	16	18.33333	1.785714	0.010086	1.572749	1.572748788	0.653288251	0.653288	0.046603	30	18	22	23.33333	38	44	43	41.66667
sseC	67	37	48	1.488136	0.023542	1.309865	1.309864769	0.389417875	0.389418	0.088703	107	98	90	98.33333	147	135	157	146.3333
sseD	17	9	10.66667	1.4	0.275964	1.226903	1.22690337	0.295021627	0.295022	0.489314	21	28	31	26.66667	37	37	38	37.33333
sseE	25	9	18.33333	2.122449	0.001579	1.860276	1.860275846	0.895516563	0.895517	0.010931	17	18	14	16.33333	39	26	39	34.66667
sseF	20	11	14	1.976744	0.007531	1.732853	1.732852786	0.793149096	0.793149	0.03716	16	13	14	14.33333	33	25	27	28.33333
sseG	36	7	16.66667	1.675676	0.030496	1.477235	1.477235261	0.562899605	0.5629	0.108798	31	28	15	24.66667	51	38	35	41.33333

Feature ID	Experiment - Range (original values)	Experiment - IQR (original values)	Experiment - Difference (original values)	Experiment - Fold Change (original values)	EDGE test: yccT NT vs WT NT , tagwise dispersions - P-value	EDGE test: yccT NT vs WT NT , tagwise dispersions - Fold change	yccT NT vs WT NT ABS|FC|	yccT NT vs WT NT Log2FC	yccT NT vs WT NT Log2FC +-	EDGE test: yccT NT vs WT NT , tagwise dispersions - FDR p-value correction	WT NT - WT.1.S22 - Expression values	WT NT - WT.2.S23 - Expression values	WT NT - WT.3.S24 - Expression values	WT NT - Means	yccT NT - yccT.1.S28 - Expression values	yccT NT - yccT.2.S29 - Expression values	yccT NT - yccT.3.S30 - Expression values	yccT NT - Means
sseI	34	7	19.33333	1.423358	0.118489	1.254029	1.254029122	0.326570852	0.326571	0.280369	50	52	35	45.66667	69	57	69	65
sseJ	21	7	7	1.567568	0.225061	1.393029	1.393029169	0.478225468	0.478225	0.429662	20	8	9	12.33333	16	29	13	19.33333
sspA	1155	589	-720.333	-1.10457	0.150632	-1.2626	1.262598193	0.336395591	-0.3364	0.326382	7449	7706	7672	7609	7255	6551	6860	6888.667
sspB	713	208	-438.333	-1.18231	0.050822	-1.35502	1.35501654	0.438310462	-0.43831	0.158038	2638	2894	2996	2842.667	2500	2283	2430	2404.333
sspH2	233	118	134.3333	1.404618	0.087448	1.24938	1.249380176	0.321212543	0.321213	0.229842	419	334	243	332	471	452	476	466.3333
ssrA	131	99	92.66667	1.978873	6.64E-05	1.760257	1.760256684	0.81578582	0.815786	0.000855	134	79	71	94.66667	178	182	202	187.3333
ssrB	52	32	21.33333	1.260163	0.450583	1.119172	1.119172321	0.162432188	0.162432	0.659356	112	69	65	82	92	101	117	103.3333
stbA	10	1	4	2.333333	0.114011	2.010951	2.010951301	1.007878144	1.007878	0.273811	4	0	5	3	10	6	5	7
stbB	4	1	1.666667	1.833333	0.46419	1.57996	1.579959567	0.659887639	0.659888	0.672385	3	1	2	2	4	2	5	3.666667
stbC	158	62	79.66667	1.364885	0.133188	1.206066	1.206066378	0.270309311	0.270309	0.301153	268	206	181	218.3333	306	249	339	298
stbD	78	62	58.66667	1.961749	0.000117	1.741055	1.741054925	0.799961717	0.799962	0.001341	85	51	47	61	113	125	121	119.6667
stbE	17	4	5.333333	1.340426	0.450485	1.18361	1.183609934	0.24319371	0.243194	0.659356	23	16	8	15.66667	25	18	20	21
stdA	36	5	7.333333	1.138365	1	1.003077	1.003077115	0.004432523	0.004433	1	70	34	55	53	64	60	57	60.33333
stdB	73	6	28	1.25	0.432486	1.102177	1.102177276	0.140356288	0.140356	0.643934	125	122	89	112	162	130	128	140
stdC	17	8	4.666667	1.229508	0.677699	1.09032	1.090320145	0.124751809	0.124752	0.826875	24	24	13	20.33333	16	29	30	25
stfA	14	3	-7.66667	-1.60526	0.008641	-1.81803	1.818033018	0.862378401	-0.86238	0.041455	16	26	19	20.33333	13	12	13	12.66667
stfC	83	32	44	1.445946	0.066414	1.284226	1.28422645	0.360899618	0.3609	0.192264	124	107	65	98.66667	148	139	141	142.6667
stfD	16	5	3.333333	1.185185	0.881176	1.052435	1.052434551	0.073730517	0.073731	0.962171	27	14	13	18	19	29	16	21.33333
stfE	8	0	3.333333	1.714286	0.278607	1.494804	1.494804393	0.579956709	0.579957	0.492563	2	6	6	4.666667	6	8	10	8
stfF	14	2	6.666667	1.487805	0.246435	1.310994	1.310993866	0.390660935	0.390661	0.45756	16	14	11	13.66667	16	25	20	20.33333
stfG	15	7	5.333333	1.457143	0.335601	1.284288	1.284287738	0.360968467	0.360968	0.549741	18	10	7	11.66667	22	17	12	17
sthA	44	20	29.66667	2.390625	1.52E-05	2.0981	2.098099865	1.069083349	1.069083	0.000261	26	21	17	21.33333	61	41	51	51
sthB	123	63	83.33333	1.932836	5.2E-05	1.712666	1.712666227	0.776244019	0.776244	0.000708	117	88	63	89.33333	181	151	186	172.6667
sthD	57	32	37.33333	1.751678	0.001604	1.547384	1.547383673	0.629830957	0.629831	0.011088	62	51	36	49.66667	93	85	83	87
sthE	103	56	72.66667	1.495455	0.019871	1.314845	1.314845094	0.394892841	0.394893	0.077643	165	133	142	146.6667	224	198	236	219.3333
stiA	83	30	45	1.652174	0.013072	1.470655	1.470654891	0.556458738	0.556459	0.056731	90	79	38	69	121	109	112	114
stiB	29	7	13.33333	1.454545	0.151195	1.287539	1.287539092	0.364616236	0.364616	0.327441	37	33	18	29.33333	40	47	41	42.66667
stiC	67	19	41.33333	1.571429	0.016199	1.386637	1.386636929	0.471590088	0.47159	0.066771	76	84	57	72.33333	124	122	95	113.6667
stiH	95	29	47.33333	1.239057	0.433207	1.095156	1.095155647	0.131135924	0.131136	0.644357	229	204	161	198	256	233	247	245.3333
stpA	181	112	130.6667	1.637398	0.001209	1.446803	1.44680322	0.532868714	0.532869	0.008901	233	214	168	205	349	326	332	335.6667
sucA	9803	2594	4585	1.311615	0.406363	1.160023	1.160023126	0.214153568	0.214154	0.620895	15416	18010	10715	14713.67	20518	17074	20304	19298.67
sucB	6059	2691	338.3333	1.033187	0.649377	-1.08899	1.088994593	0.122996791	-0.123	0.807754	11709	12467	6408	10194.67	11600	8909	11090	10533
sucC	7513	2594	-374.667	-1.0434	0.473293	-1.16743	1.167427811	0.223333342	-0.22333	0.679572	10706	11916	4403	9008.333	9744	7150	9007	8633.667
sucD	5919	1938	-1331.33	-1.23091	0.148626	-1.37303	1.373029688	0.45736282	-0.45736	0.323936	9179	8852	3260	7097	6681	4743	5873	5765.667
sufA	24	8	13	2.147059	0.006008	1.889717	1.889716602	0.918169891	0.91817	0.031066	13	14	7	11.33333	21	31	21	24.33333
sufB	76	27	43	1.335065	0.174419	1.180556	1.180555636	0.239466033	0.239466	0.36199	144	135	106	128.3333	170	182	162	171.3333

Feature ID	Experiment - Range (original values)	Experiment - IQR (original values)	Experiment - Difference (original values)	Experiment - Fold Change (original values)	EDGE test: yccT NT vs WT NT , tagwise dispersions - P-value	EDGE test: yccT NT vs WT NT , tagwise dispersions - Fold change	yccT NT vs WT NT ABS|FC|	yccT NT vs WT NT Log2FC	yccT NT vs WT NT Log2FC +-	EDGE test: yccT NT vs WT NT , tagwise dispersions - FDR p-value correction	WT NT - WT.1.S22 - Expression values	WT NT - WT.2.S23 - Expression values	WT NT - WT.3.S24 - Expression values	WT NT - Means	yccT NT - yccT.1.S28 - Expression values	yccT NT - yccT.2.S29 - Expression values	yccT NT - yccT.3.S30 - Expression values	yccT NT - Means
sufC	28	9	9.666667	1.152632	0.940799	1.015374	1.015374414	0.022011812	0.022012	0.994912	71	67	52	63.33333	80	62	77	73
sufD	138	14	65.66667	1.289706	0.275314	1.137328	1.137328237	0.185648681	0.185649	0.488552	253	243	184	226.6667	322	257	298	292.3333
sufI	437	201	335	1.267501	0.428085	1.109585	1.109584925	0.150020092	0.15002	0.639913	1265	1247	1245	1252.333	1682	1448	1632	1587.333
sufS	120	73	77.33333	1.412811	0.073593	1.246426	1.2464258	0.317797001	0.317797	0.205036	188	223	151	187.3333	271	261	262	264.6667
sugE	181	52	101	1.220845	0.530714	1.078483	1.078482929	0.10900334	0.109003	0.723366	497	487	388	457.3333	569	567	539	558.3333
suhB	446	178	249.6667	1.472854	0.044432	1.310548	1.310547783	0.390169956	0.39017	0.143811	657	550	377	528	782	728	823	777.6667
sulA	418	64	101.3333	1.106108	0.894357	-1.01813	1.018132036	0.025924669	-0.02592	0.968923	1142	999	724	955	1117	1058	994	1056.333
sun	484	344	376.3333	1.374213	0.130014	1.207391	1.207391297	0.271893308	0.271893	0.29726	1090	957	970	1005.667	1441	1314	1391	1382
surA	821	521	-220.333	-1.05854	0.234651	-1.21697	1.216973672	0.283297957	-0.2833	0.442703	3545	4066	4342	3984.333	4071	3521	3700	3764
surE	327	62	124.3333	1.120439	0.920064	-1.01283	1.012828924	0.018390511	-0.01839	0.98331	1114	1090	893	1032.333	1198	1052	1220	1156.667
syd	281	74	96	1.155005	0.851007	1.024378	1.024377551	0.034747541	0.034748	0.945302	751	624	483	619.3333	764	698	684	715.3333
tag	261	66	19.66667	1.007697	0.411145	-1.13488	1.134877535	0.182536625	-0.18254	0.625275	2514	2445	2706	2555	2603	2541	2580	2574.667
talA	41	19	4	1.025052	0.397325	-1.10729	1.107294955	0.147039571	-0.14704	0.613217	173	166	140	159.6667	181	163	147	163.6667
talB	14949	3717	-9902	-1.81022	0.000182	-2.08542	2.085415188	1.06033464	-1.06033	0.001921	16005	26678	23687	22123.33	12647	11729	12288	12221.33
talC	814	359	-563	-1.47671	0.000208	-1.68741	1.687406244	0.754807345	-0.75481	0.002099	1527	1939	1766	1744	1168	1250	1125	1181
tas	508	106	-170.667	-1.07515	0.176598	-1.21827	1.218270337	0.284834306	-0.28483	0.364635	2640	2553	2132	2441.667	2322	2275	2216	2271
tatA	1138	58	-405.333	-1.12872	0.123778	-1.29825	1.298254335	0.376573043	-0.37657	0.288056	3166	3354	4143	3554.333	3224	3218	3005	3149
tatB	650	84	-129.333	-1.04076	0.275556	-1.19462	1.194623786	0.256556353	-0.25656	0.488786	3020	3216	3670	3302	3234	3132	3152	3172.667
tatC	547	163	-173.333	-1.08769	0.145713	-1.2509	1.250897208	0.322963241	-0.32296	0.319474	1825	2253	2372	2150	2061	1898	1971	1976.667
tbpA	58	26	37.33333	1.186978	0.741481	1.041914	1.041913681	0.05923576	0.059236	0.871255	211	189	199	199.6667	247	225	239	237
tcp	3885	1395	2028.667	1.355387	0.257909	1.201534	1.201534105	0.264877599	0.264878	0.469513	6739	5971	4415	5708.333	8300	7366	7545	7737
tctD	62	40	44.66667	1.582609	0.009885	1.395095	1.395094519	0.480362869	0.480363	0.045863	75	91	64	76.66667	115	123	126	121.3333
tctE	190	113	133.3333	1.504414	0.015226	1.327513	1.327512699	0.408725662	0.408726	0.063766	294	267	232	264.3333	391	380	422	397.6667
tdcA	10456	3130	-7303.33	-1.6057	0.001323	-1.84697	1.846974997	0.885164336	-0.88516	0.009503	15073	21003	22007	19361	11551	12679	11943	12057.67
tdcB	109151	17400	-62946.3	-5.32334	7.73E-10	-6.28434	6.284335088	2.651760109	-2.65176	4.57E-08	32091	77325	123102	77506	14691	15037	13951	14559.67
tdcC	117438	39864	-69991.3	-3.85667	1.31E-09	-4.49433	4.494330488	2.168106216	-2.16811	7.62E-08	62867	81120	139490	94492.33	23003	28448	22052	24501
tdcD	61191	18781	-34987.3	-2.97056	4.01E-07	-3.43658	3.436582725	1.780974688	-1.78097	1.22E-05	46667	34852	76708	52742.33	16071	21677	15517	17755
tdcE	58308	15841	-11028.7	-1.21541	0.212372	-1.39123	1.391233254	0.476364322	-0.47636	0.413271	75903	26235	84543	62227	46924	61256	45415	51198.33
tdcG	82964	40612	51008	2.851649	0.00304	2.532423	2.532422785	1.340518281	1.340518	0.018354	43945	7438	31259	27547.33	71871	90402	73393	78555.33
tdh	10726	6177	-8699.67	-1.90789	1.29E-05	-2.19334	2.193341265	1.1331303	-1.13313	0.000226	15455	19789	19602	18282	10406	9278	9063	9582.333
tdk	309	198	203.6667	1.293891	0.272753	1.14271	1.142709801	0.192459067	0.192459	0.485837	790	678	611	693	920	894	876	896.6667
tehA	175	74	110.6667	1.318618	0.224138	1.159376	1.159375981	0.213348502	0.213349	0.428125	384	331	327	347.3333	467	405	502	458
tehB	237	69	93.33333	1.099045	0.777112	-1.03667	1.036669143	0.051955525	-0.05196	0.892069	1022	872	933	942.3333	1109	1002	996	1035.667
tesA	199	166	126.3333	1.249014	0.428439	1.104951	1.104951075	0.143982492	0.143982	0.640058	621	455	446	507.3333	640	616	645	633.6667
tesB	337	182	163	1.216276	0.552477	1.079026	1.07902573	0.109729267	0.109729	0.738781	925	732	604	753.6667	941	895	914	916.6667
tgt	602	179	-181	-1.09993	0.117138	-1.26682	1.266819391	0.341210855	-0.34121	0.278454	1763	1957	2257	1992.333	1942	1655	1837	1811.333

Feature ID	Experiment - Range (original values)	Experiment - IQR (original values)	Experiment - Difference (original values)	Experiment - Fold Change (original values)	EDGE test: yccT NT vs WT NT , tagwise dispersions - P-value	EDGE test: yccT NT vs WT NT , tagwise dispersions - Fold change	yccT NT vs WT NT ABS|FC|	yccT NT vs WT NT Log2FC	yccT NT vs WT NT Log2FC +-	EDGE test: yccT NT vs WT NT , tagwise dispersions - FDR p-value correction	WT NT - WT.1.S22 - Expression values	WT NT - WT.2.S23 - Expression values	WT NT - WT.3.S24 - Expression values	WT NT - Means	yccT NT - yccT.1.S28 - Expression values	yccT NT - yccT.2.S29 - Expression values	yccT NT - yccT.3.S30 - Expression values	yccT NT - Means
thiC	57	14	16.66667	1.05291	0.535632	-1.07658	1.076576615	0.106450993	-0.10645	0.726399	334	329	282	315	320	339	336	331.6667
thiD	67	55	38.33333	1.396552	0.13073	1.238584	1.238584225	0.308691976	0.308692	0.298174	135	80	75	96.66667	142	122	141	135
thiE	11	4	-4	-1.09449	0.166159	-1.24348	1.243478085	0.314381082	-0.31438	0.350761	43	51	45	46.33333	40	46	41	42.33333
thiF	16	4	7	1.156716	0.961603	1.016886	1.01688555	0.024157314	0.024157	1	49	40	45	44.66667	56	47	52	51.66667
thiG	37	7	-4	-1.07547	0.248301	-1.21262	1.212618945	0.278126267	-0.27813	0.459749	78	52	41	57	52	45	62	53
thiH	48	17	11	1.083969	0.732667	-1.04599	1.045988908	0.064867553	-0.06487	0.864336	159	111	123	131	147	139	140	142
thiI	610	95	318.3333	1.186596	0.759497	1.042511	1.042510662	0.060062139	0.060062	0.881438	1760	1810	1548	1706	2158	1855	2060	2024.333
thiJ	163	101	65.66667	1.090616	0.760744	-1.03854	1.0385355	0.054550532	-0.05455	0.882424	820	697	657	724.6667	798	813	760	790.3333
thiL	102	44	48.33333	1.165525	0.846859	1.024638	1.024637754	0.035113955	0.035114	0.942334	327	283	266	292	368	286	367	340.3333
thiM	143	78	82.66667	1.478764	0.037161	1.316504	1.31650425	0.396712179	0.396712	0.12608	225	170	123	172.6667	248	252	266	255.3333
thrA	23141	18513	16143	3.602171	0.006473	3.368497	3.368497056	1.752105038	1.752105	0.032932	15627	1714	1270	6203.667	24411	20227	22402	22346.67
thrB	9653	7340	6774	3.233923	0.001198	2.984304	2.984304216	1.577394609	1.577395	0.008831	6662	1424	1011	3032.333	10664	8764	9991	9806.333
thrC	14849	11379	11296.33	3.796814	2.97E-05	3.474481	3.474481076	1.796797523	1.796798	0.000459	8197	2145	1775	4039	16624	13524	15858	15335.33
thrL	266	221	200	1.895522	0.00033	1.685189	1.685189089	0.75291048	0.75291	0.003055	313	167	190	223.3333	426	433	411	423.3333
thrS	16127	3057	426	1.008287	0.495394	-1.12452	1.124516614	0.169304976	-0.1693	0.699065	61454	47429	45327	51403.33	57947	47242	50299	51829.33
thyA	492	129	-293.667	-1.23862	0.012145	-1.41962	1.419624738	0.505509619	-0.50551	0.053512	1338	1673	1562	1524.333	1302	1181	1209	1230.667
tig	2604	970	-674.667	-1.08564	0.207526	-1.25164	1.251636896	0.323816093	-0.32382	0.40742	7142	8769	9746	8552.333	8288	7318	8027	7877.667
tktA	16420	3482	-11276.3	-1.83772	0.000201	-2.12772	2.12772471	1.089311504	-1.08931	0.002053	17201	27998	29012	24737	14071	12592	13719	13460.67
tktB	252	115	137.6667	1.217483	0.546024	1.077494	1.077493995	0.10767983	0.10768	0.734255	727	635	537	633	773	789	750	770.6667
tldD	720	277	-287.667	-1.09062	0.158578	-1.23821	1.238206513	0.308251953	-0.30825	0.338304	3736	3542	3108	3462	3385	3122	3016	3174.333
tmk	165	48	104	1.22724	0.554177	1.075602	1.075602259	0.105144691	0.105145	0.739849	450	492	431	457.6667	596	498	591	561.6667
tnpA_5	44	29	22	1.273859	0.378743	1.123394	1.123393624	0.16786352	0.167864	0.595321	101	72	68	80.33333	112	85	110	102.3333
tolA	108	54	70	1.332278	0.220738	1.165742	1.165742275	0.22124887	0.221249	0.424878	209	196	227	210.6667	275	263	304	280.6667
tolB	746	210	-337.667	-1.1497	0.070711	-1.3114	1.311395637	0.391103	-0.3911	0.199949	2515	2855	2410	2593.333	2434	2109	2224	2255.667
tolC	2411	410	126.3333	1.012873	0.493413	-1.11801	1.11800877	0.160931505	-0.16093	0.697625	11022	9809	8611	9814	10299	9966	9556	9940.333
tolQ	396	205	297.6667	1.723663	0.001262	1.506806	1.506806447	0.591494111	0.591494	0.009194	428	378	428	411.3333	774	633	720	709
tolR	124	60	89	1.41784	0.080097	1.238748	1.238747518	0.308882167	0.308882	0.217869	211	201	227	213	325	271	310	302
tonB	131	53	82.66667	1.629442	0.002293	1.437415	1.437415032	0.523476678	0.523477	0.014452	146	144	104	131.3333	235	197	210	214
topA	649	273	-28	-1.00814	0.366656	-1.15814	1.158144888	0.211815751	-0.21182	0.583568	3156	3437	3805	3466	3597	3222	3495	3438
topB	447	115	-25.6667	-1.01174	0.331478	-1.15505	1.155052457	0.207958374	-0.20796	0.545487	2072	2488	2074	2211.333	2329	2041	2187	2185.667
torA	876	755	755.6667	2.096759	1.74E-06	1.847541	1.847541243	0.885606571	0.885607	4.27E-05	797	623	647	689	1499	1402	1433	1444.667
torC	242	187	203.6667	1.862994	6.96E-05	1.634607	1.634607162	0.708943961	0.708944	0.000889	225	250	233	236	420	432	467	439.6667
torD	129	89	103.6667	2.072414	2.19E-07	1.825822	1.825821891	0.868546038	0.868546	7.4E-06	109	98	83	96.66667	212	187	202	200.3333
torR	282	68	-6.66667	-1.00718	0.313392	-1.13668	1.13668154	0.184828116	-0.18483	0.528001	1061	966	779	935.3333	898	966	922	928.6667
torS	470	92	-179.667	-1.09928	0.109262	-1.25474	1.254740008	0.327388458	-0.32739	0.266158	1833	2237	1898	1989.333	1877	1767	1785	1809.667
torT	249	118	145	1.189791	0.693533	1.049846	1.049845636	0.070177217	0.070177	0.837317	840	775	677	764	926	908	893	909

Feature ID	Experiment - Range (original values)	Experiment - IQR (original values)	Experiment - Difference (original values)	Experiment - Fold Change (original values)	EDGE test: yccT NT vs WT NT , tagwise dispersions - P-value	EDGE test: yccT NT vs WT NT , tagwise dispersions - Fold change	yccT NT vs WT NT ABS|FC|	yccT NT vs WT NT Log2FC	yccT NT vs WT NT Log2FC +-	EDGE test: yccT NT vs WT NT , tagwise dispersions - FDR p-value correction	WT NT - WT.1.S22 - Expression values	WT NT - WT.2.S23 - Expression values	WT NT - WT.3.S24 - Expression values	WT NT - Means	yccT NT - yccT.1.S28 - Expression values	yccT NT - yccT.2.S29 - Expression values	yccT NT - yccT.3.S30 - Expression values	yccT NT - Means
tpiA	9848	2288	-5775	-1.72972	0.000474	-2.00848	2.008480117	1.006104179	-1.0061	0.004196	10076	13776	17215	13689	8587	7367	7788	7914
tpx	2125	361	-971	-1.41313	0.006182	-1.61557	1.615569223	0.692042567	-0.69204	0.031741	2666	4339	2959	3321.333	2532	2214	2305	2350.333
treA	451	217	115.6667	1.155675	0.844507	1.028664	1.028663639	0.040771315	0.040771	0.94115	1029	622	578	743	923	814	839	858.6667
treB	311	34	-161.333	-1.24872	0.014897	-1.43785	1.437852048	0.523915233	-0.52392	0.062627	651	838	941	810	630	641	675	648.6667
treC	2466	223	-1571	-3.1972	1.42E-07	-3.75191	3.751908011	1.907624456	-1.90762	4.93E-06	974	2777	3107	2286	753	641	751	715
treF	538	238	310	1.326087	0.201533	1.172407	1.172406818	0.229473263	0.229473	0.398911	1106	954	792	950.6667	1330	1192	1260	1260.667
treR	96	9	13	1.046595	0.520683	-1.07979	1.079788614	0.110748909	-0.11075	0.717174	319	295	223	279	303	287	286	292
trg	2853	913	1248.667	1.277051	0.472195	1.130549	1.130548801	0.177023268	0.177023	0.678436	4672	5585	3264	4507	6117	5473	5677	5755.667
trkA	310	157	197	1.27983	0.39179	1.118668	1.118667858	0.161781752	0.161782	0.608703	713	646	753	704	956	877	870	901
trkH	161	54	-100.333	-1.11419	0.062213	-1.27538	1.27537513	0.350921653	-0.35092	0.18307	911	1009	1017	979	917	856	863	878.6667
trmA	224	12	-86	-1.07871	0.095994	-1.22943	1.229433656	0.297993885	-0.29799	0.244312	1223	1184	1129	1178.667	1141	999	1138	1092.667
trmD	14985	4882	-8671	-3.31907	3.71E-10	-3.79912	3.799116221	1.925663847	-1.92566	2.42E-08	8788	18216	10226	12410	4080	3231	3906	3739
trmE	70	15	-28.6667	-1.11862	0.045389	-1.27728	1.27727821	0.3530728	-0.35307	0.145951	246	302	263	270.3333	254	232	239	241.6667
trmU	708	276	424	1.298662	0.311347	1.141428	1.141428427	0.190840399	0.19084	0.526523	1579	1369	1311	1419.667	2019	1645	1867	1843.667
trpA	208	76	2	1.003289	0.337058	-1.12868	1.128679245	0.174635551	-0.17464	0.550703	741	550	533	608	651	553	626	610
trpB	270	79	44.33333	1.057501	0.587546	-1.07029	1.070285137	0.097995199	-0.098	0.76537	931	721	661	771	888	758	800	815.3333
trpC	226	200	116.6667	1.180227	0.739111	1.04356	1.043559861	0.061513359	0.061513	0.868931	796	570	576	647.3333	776	729	787	764
trpD	76	27	44.33333	1.401813	0.079621	1.236808	1.236807667	0.306621167	0.306621	0.217107	127	110	94	110.3333	170	157	137	154.6667
trpE	42	19	25.66667	1.403141	0.094201	1.237726	1.237726246	0.307692262	0.307692	0.240702	73	66	52	63.66667	94	89	85	89.33333
trpH	358	130	179.6667	1.20683	0.603246	1.067182	1.067181976	0.093806205	0.093806	0.77698	991	884	731	868.6667	1089	1042	1014	1048.333
trpR	93	50	-26	-1.05376	0.125691	-1.19755	1.197546836	0.260082081	-0.26008	0.291133	551	514	464	509.6667	518	458	475	483.6667
trpS	539	180	-300.667	-1.22505	0.016404	-1.40767	1.407666383	0.493305456	-0.49331	0.067409	1415	1775	1720	1636.667	1476	1236	1296	1336
trpS2	184	14	78.66667	1.205039	0.609731	1.065702	1.06570218	0.091804321	0.091804	0.780424	425	425	301	383.6667	485	439	463	462.3333
truA	127	5	54.33333	1.243647	0.445755	1.095867	1.095867073	0.132072812	0.132073	0.655975	243	245	181	223	308	240	284	277.3333
truB	828	250	-468.333	-1.42966	0.00027	-1.62845	1.628449243	0.703498754	-0.7035	0.002569	1537	1787	1351	1558.333	1210	959	1101	1090
truD	177	70	66.66667	1.049298	0.519793	-1.08921	1.089208942	0.123280731	-0.12328	0.71666	1299	1407	1351	1352.333	1476	1337	1444	1419
trxA	4551	1699	-3140	-1.38201	0.008085	-1.58246	1.582455296	0.662164745	-0.66216	0.039279	10131	12177	11771	11359.67	8601	8432	7626	8219.667
trxB	430	185	-171	-1.07356	0.182888	-1.23095	1.230953472	0.299776232	-0.29978	0.371904	2231	2643	2613	2495.667	2416	2213	2345	2324.667
trxC	104	43	26	1.111111	0.892751	-1.01774	1.017736295	0.025363794	-0.02536	0.968605	280	241	181	234	285	226	269	260
tsf	14621	2655	-7697.33	-3.08186	1.28E-07	-3.54021	3.54020832	1.823834257	-1.82383	4.52E-06	6421	17984	9779	11394.67	3963	3363	3766	3697.333
tsr	3927	393	569.3333	1.077038	0.781113	-1.04959	1.049587659	0.069822661	-0.06982	0.895038	7512	9293	5366	7390.333	8500	7493	7886	7959.667
tsx	17357	3517	-8214.33	-5.8225	1.72E-10	-6.62722	6.627215057	2.728402736	-2.7284	1.19E-08	5223	18877	5653	9917.667	1884	1520	1706	1703.333
ttdA	276	110	35.33333	1.063511	0.670089	-1.05946	1.059464407	0.083335121	-0.08334	0.822256	734	477	458	556.3333	624	564	587	591.6667
ttk	257	60	-83.6667	-1.11947	0.081862	-1.28945	1.289445231	0.366750496	-0.36675	0.220943	622	851	879	784	728	668	705	700.3333
ttrA	404	230	271	1.464571	0.045328	1.290397	1.290396784	0.367814748	0.367815	0.145858	689	513	548	583.3333	868	778	917	854.3333
ttrB	118	72	76	1.525346	0.012149	1.347966	1.347965717	0.430783805	0.430784	0.053512	179	135	120	144.6667	238	207	217	220.6667

Feature ID	Experiment - Range (original values)	Experiment - IQR (original values)	Experiment - Difference (original values)	Experiment - Fold Change (original values)	EDGE test: yccT NT vs WT NT , tagwise dispersions - P-value	EDGE test: yccT NT vs WT NT , tagwise dispersions - Fold change	yccT NT vs WT NT ABS|FC|	yccT NT vs WT NT Log2FC	yccT NT vs WT NT Log2FC +-	EDGE test: yccT NT vs WT NT , tagwise dispersions - FDR p-value correction	WT NT - WT.1.S22 - Expression values	WT NT - WT.2.S23 - Expression values	WT NT - WT.3.S24 - Expression values	WT NT - Means	yccT NT - yccT.1.S28 - Expression values	yccT NT - yccT.2.S29 - Expression values	yccT NT - yccT.3.S30 - Expression values	yccT NT - Means
ttrC	90	54	52	1.508143	0.032037	1.336379	1.336379088	0.418329312	0.418329	0.112356	134	87	86	102.3333	141	146	176	154.3333
ttrR	155	53	-81.6667	-1.16873	0.020423	-1.33431	1.334309798	0.416093668	-0.41609	0.079383	533	617	547	565.6667	510	462	480	484
ttrS	1396	407	531.6667	1.110411	0.90107	-1.01974	1.01973627	0.028196082	-0.0282	0.971919	5419	4869	4158	4815.333	5554	5211	5276	5347
tufA	18280	9255	-13143	-1.83967	4.14E-05	-2.11058	2.110583332	1.077641792	-1.07764	0.000594	24246	33177	28964	28795.67	17070	14897	14991	15652.67
tus	237	51	-57.6667	-1.08733	0.099267	-1.23344	1.233437368	0.30268446	-0.30268	0.249491	723	834	597	718	690	639	652	660.3333
typA	1204	379	568.3333	1.176996	0.882109	1.024404	1.024404411	0.034785369	0.034785	0.962716	3131	2992	3510	3211	4196	3359	3783	3779.333
tyrA	454	215	218	1.236614	0.494161	1.090763	1.090762832	0.125337447	0.125337	0.698001	1088	873	803	921.3333	1257	1034	1127	1139.333
tyrB	476	326	-385	-1.32563	0.002008	-1.51599	1.515986865	0.600257253	-0.60026	0.013125	1494	1603	1605	1567.333	1250	1168	1129	1182.333
tyrP	271	124	-19.3333	-1.01209	0.28576	-1.14846	1.148461639	0.199702669	-0.1997	0.501665	1714	1698	1443	1618.333	1593	1540	1664	1599
tyrR	548	155	54.66667	1.018306	0.468716	-1.11509	1.11509118	0.157161683	-0.15716	0.676072	3206	3085	2668	2986.333	3216	2930	2977	3041
tyrS	1077	484	-159	-1.03655	0.298508	-1.1921	1.192099912	0.253505156	-0.25351	0.514264	3834	4784	4911	4509.667	4565	4081	4406	4350.667
ubiA	240	64	39.66667	1.046961	0.517351	-1.08338	1.083376766	0.115535056	-0.11554	0.715475	900	916	718	844.6667	958	859	836	884.3333
ubiB	444	255	-159.667	-1.09164	0.127479	-1.25557	1.255571576	0.328344275	-0.32834	0.293738	1621	2035	2050	1902	1876	1606	1745	1742.333
ubiC	272	75	74.66667	1.093101	0.77313	-1.03688	1.036875919	0.052243261	-0.05224	0.889421	881	858	667	802	939	806	885	876.6667
ubiE	928	524	-566.333	-1.15081	0.084422	-1.31629	1.316289945	0.396477313	-0.39648	0.225075	4318	4175	4472	4321.667	4071	3651	3544	3755.333
ubiF	679	50	18.33333	1.008494	0.429989	-1.12087	1.120871672	0.164621114	-0.16462	0.640861	2489	2176	1810	2158.333	2261	2143	2126	2176.667
ubiG	817	357	420	1.324826	0.223047	1.175967	1.175967325	0.233847975	0.233848	0.427289	1585	1291	1003	1293	1820	1671	1648	1713
ubiH	919	372	103.6667	1.034823	0.556194	-1.0944	1.094399967	0.130140091	-0.13014	0.741429	3283	3226	2422	2977	3341	2854	3047	3080.667
ubiX	206	155	152.3333	1.391267	0.087376	1.228618	1.228617592	0.297035946	0.297036	0.229842	444	379	345	389.3333	540	534	551	541.6667
ucpA	3654	1303	1242	1.10105	0.862502	-1.02875	1.028749689	0.040891994	-0.04089	0.951631	14084	12136	10653	12291	14307	12853	13439	13533
udg	347	186	238	1.440741	0.071687	1.268296	1.268295831	0.342891295	0.342891	0.201935	609	461	550	540	736	808	790	778
udhA	1146	200	-556.333	-1.20506	0.043139	-1.37805	1.378050747	0.462629016	-0.46263	0.140702	2956	3683	3169	3269.333	2846	2537	2756	2713
udk	305	194	234	1.359447	0.174964	1.188539	1.188538798	0.249188998	0.249189	0.362783	650	616	687	651	921	844	890	885
udp	22975	2740	-9994.33	-3.74444	1.17E-06	-4.22711	4.227110102	2.07967169	-2.07967	3.11E-05	8071	26389	6448	13636	3803	3708	3414	3641.667
ugpA	50	26	31.33333	1.311258	0.252216	1.153588	1.153587627	0.206127596	0.206128	0.463289	112	91	99	100.6667	130	125	141	132
ugpB	161	102	107	1.127888	0.943212	-1.00903	1.009032556	0.012972723	-0.01297	0.99675	875	829	806	836.6667	967	931	933	943.6667
ugpC	41	7	7.666667	1.063361	0.567611	-1.07553	1.075526377	0.105042908	-0.10504	0.748361	115	126	122	121	154	113	119	128.6667
ugpE	35	23	17.66667	1.248826	0.518195	1.097295	1.097294706	0.13395105	0.133951	0.715749	65	88	60	71	94	77	95	88.66667
ugpQ	191	57	103	1.293169	0.274398	1.139686	1.139686417	0.188636923	0.188637	0.487547	385	362	307	351.3333	498	446	419	454.3333
ugtL	5	2	-0.33333	-1.05	0.766103	-1.18959	1.189586499	0.250460179	-0.25046	0.886011	6	8	7	7	5	5	10	6.666667
uhpA	106	15	-37.3333	-1.12174	0.059183	-1.28175	1.281745334	0.358109646	-0.35811	0.176236	296	402	334	344	311	297	312	306.6667
uhpB	59	14	-5.66667	-1.05075	0.216867	-1.19746	1.197455888	0.259972511	-0.25997	0.420174	96	155	101	117.3333	116	104	115	111.6667
uhpC	35	14	-9	-1.12676	0.081958	-1.28982	1.289816915	0.367166295	-0.36717	0.220943	66	94	80	80	84	59	70	71
uhpT	83	66	-28.6667	-1.12573	0.059866	-1.29214	1.292140322	0.369762751	-0.36976	0.177651	267	221	282	256.6667	283	200	201	228
umuC	60	33	26	1.386139	0.186833	1.2301	1.230100161	0.298775792	0.298776	0.376469	95	57	50	67.33333	90	80	110	93.33333
ung	391	180	136.6667	1.106743	0.867812	-1.02202	1.02202196	0.031426196	-0.03143	0.955348	1504	1224	1113	1280.333	1458	1389	1404	1417

Feature ID	Experiment - Range (original values)	Experiment - IQR (original values)	Experiment - Difference (original values)	Experiment - Fold Change (original values)	EDGE test: yccT NT vs WT NT , tagwise dispersions - P-value	EDGE test: yccT NT vs WT NT , tagwise dispersions - Fold change	yccT NT vs WT NT ABS|FC|	yccT NT vs WT NT Log2FC	yccT NT vs WT NT Log2FC +-	EDGE test: yccT NT vs WT NT , tagwise dispersions - FDR p-value correction	WT NT - WT.1.S22 - Expression values	WT NT - WT.2.S23 - Expression values	WT NT - WT.3.S24 - Expression values	WT NT - Means	yccT NT - yccT.1.S28 - Expression values	yccT NT - yccT.2.S29 - Expression values	yccT NT - yccT.3.S30 - Expression values	yccT NT - Means
upp	468	51	-227.333	-1.23631	0.019447	-1.42352	1.423522283	0.509465076	-0.50947	0.076597	968	1199	1401	1189.333	951	1002	933	962
uppS	534	189	317.3333	1.122886	0.92072	-1.01514	1.015137412	0.021675028	-0.02168	0.983597	2650	2593	2504	2582.333	3038	2782	2879	2899.667
uraA	199	12	-81	-1.47002	0.003825	-1.71376	1.713758961	0.77716421	-0.77716	0.021784	170	236	354	253.3333	180	155	182	172.3333
usg	202	88	129.3333	1.21307	0.634019	1.062653	1.062652835	0.087670351	0.08767	0.797185	579	662	580	607	760	668	781	736.3333
ushA	645	134	339.3333	1.113388	0.873107	-1.02512	1.025120061	0.035792887	-0.03579	0.958007	3131	2774	3073	2992.667	3419	3207	3370	3332
ushB	105	37	-45.6667	-1.09601	0.065709	-1.24588	1.2458781	0.317162919	-0.31716	0.190423	549	535	480	521.3333	510	473	444	475.6667
uspA	10826	5169	2336.333	1.074172	0.770806	-1.05147	1.051471015	0.072409081	-0.07241	0.887872	38147	29028	27321	31498.67	35001	34197	32307	33835
uspB	275	66	98.33333	1.114474	0.902161	-1.01575	1.015753623	0.02255051	-0.02255	0.972858	967	878	732	859	1007	944	921	957.3333
uup	202	37	99	1.164725	0.898763	1.01708	1.017080368	0.024433682	0.024434	0.971085	596	590	617	601	792	633	675	700
uvrA	717	273	-207.333	-1.09487	0.152993	-1.26227	1.262274234	0.336025375	-0.33603	0.329885	2046	2369	2763	2392.667	2323	2050	2183	2185.333
uvrB	438	90	151.6667	1.087165	0.752175	-1.04312	1.04312359	0.060910099	-0.06091	0.878462	1898	1795	1527	1740	1965	1825	1885	1891.667
uvrC	2581	1373	1939.667	1.478536	0.092078	1.300281	1.300280905	0.378823328	0.378823	0.237899	4297	4174	3689	4053.333	6270	5547	6162	5993
uvrD	495	212	-237.667	-1.09068	0.152728	-1.23998	1.239984874	0.310322522	-0.31032	0.329634	2958	3022	2596	2858.667	2774	2562	2527	2621
uvrY	3979	3305	2857.667	1.483858	0.095743	1.314885	1.31488464	0.394936232	0.394936	0.243815	7357	5302	5059	5906	9038	8646	8607	8763.667
uxuR	363	44	-189.667	-1.13273	0.052975	-1.28642	1.286416263	0.36335755	-0.36336	0.162781	1662	1758	1436	1618.667	1468	1395	1424	1429
vacB	2401	261	-470.333	-1.03252	0.331861	-1.1737	1.173698748	0.231062162	-0.23106	0.545833	16318	14371	14110	14933	15232	14239	13917	14462.67
vacJ	603	390	406.3333	1.244436	0.49748	1.095905	1.095904966	0.132122697	0.132123	0.699966	1854	1593	1540	1662.333	2143	1983	2080	2068.667
valS	1127	256	-516.333	-1.12203	0.120413	-1.28777	1.287766888	0.36487146	-0.36487	0.283195	4338	4902	5003	4747.667	4537	3876	4281	4231.333
virK	409	296	301.6667	2.272855	7.51E-07	2.016199	2.016199079	1.011638097	1.011638	2.12E-05	330	189	192	237	598	488	530	538.6667
visC	518	122	-47.6667	-1.021	0.304057	-1.16473	1.164728955	0.219994264	-0.21999	0.519382	2234	2600	2118	2317.333	2487	2082	2240	2269.667
vsr	113	43	60.66667	1.273273	0.336234	1.118329	1.118328502	0.161344033	0.161344	0.550167	243	215	208	222	321	258	269	282.6667
wcaA	27	6	11	1.270492	0.499043	1.115921	1.115921377	0.158235385	0.158235	0.701765	46	36	40	40.66667	63	45	47	51.66667
wcaB	10	3	0.333333	1.018182	0.666682	-1.11256	1.112558406	0.153881075	-0.15388	0.820118	24	17	14	18.33333	21	19	16	18.66667
wcaC	32	19	24.33333	1.715686	0.006252	1.510659	1.510659307	0.595178332	0.595178	0.032056	37	34	31	34	53	59	63	58.33333
wcaD	26	13	17	1.836066	0.011721	1.611889	1.611889206	0.688752583	0.688753	0.052197	21	16	24	20.33333	34	42	36	37.33333
wcaE	16	4	8.333333	2.041667	0.027021	1.789181	1.789181311	0.839299594	0.8393	0.098786	11	9	4	8	20	16	13	16.33333
wcaF	8	0	0.666667	1.133333	1	1.001796	1.001796045	0.002588821	0.002589	1	9	5	1	5	5	5	7	5.666667
wcaG	18	10	5.333333	1.190476	0.80582	1.050521	1.050521496	0.071105683	0.071106	0.911195	39	21	24	28	34	29	37	33.33333
wcaH	13	8	0	-1	0.659155	-1.13391	1.133911498	0.181308043	-0.18131	0.816299	18	21	9	16	22	10	16	16
wcaI	11	2	3.333333	1.107527	0.90101	-1.02747	1.027470762	0.039097341	-0.0391	0.971919	32	30	31	31	41	30	32	34.33333
wcaJ	20	5	10	1.344828	0.342117	1.184932	1.184932362	0.24480471	0.244805	0.55671	30	35	22	29	42	41	34	39
wcaK	46	31	20.66667	1.319588	0.305964	1.167259	1.167259154	0.223124902	0.223125	0.521303	89	50	55	64.66667	96	86	74	85.33333
wcaL	16	3	6	1.136364	1	1.000561	1.000561386	0.000809682	0.00081	1	47	39	46	44	44	55	51	50
wcaM	31	7	9.666667	1.142857	1	1.001121	1.001121112	0.001616516	0.001617	1	57	72	74	67.66667	67	77	88	77.33333
wecB	694	194	-155.333	-1.06628	0.21361	-1.22464	1.224643807	0.292362196	-0.29236	0.414406	2078	2772	2647	2499	2393	2222	2416	2343.667
wecC	740	118	-379	-1.28836	0.015031	-1.4888	1.488804736	0.57415455	-0.57415	0.063071	1249	1864	1967	1693.333	1349	1227	1367	1314.333

Feature ID	Experiment - Range (original values)	Experiment - IQR (original values)	Experiment - Difference (original values)	Experiment - Fold Change (original values)	EDGE test: yccT NT vs WT NT , tagwise dispersions - P-value	EDGE test: yccT NT vs WT NT , tagwise dispersions - Fold change	yccT NT vs WT NT ABS|FC|	yccT NT vs WT NT Log2FC	yccT NT vs WT NT Log2FC +-	EDGE test: yccT NT vs WT NT , tagwise dispersions - FDR p-value correction	WT NT - WT.1.S22 - Expression values	WT NT - WT.2.S23 - Expression values	WT NT - WT.3.S24 - Expression values	WT NT - Means	yccT NT - yccT.1.S28 - Expression values	yccT NT - yccT.2.S29 - Expression values	yccT NT - yccT.3.S30 - Expression values	yccT NT - Means
wecD	209	70	-123.333	-1.32371	0.001051	-1.5138	1.513795438	0.598170264	-0.59817	0.007933	466	552	495	504.3333	404	343	396	381
wecE	415	155	-236	-1.26687	0.004836	-1.45256	1.452563956	0.538601686	-0.5386	0.026102	1058	1173	1130	1120.333	992	758	903	884.3333
wecF	89	12	28	1.048696	0.516742	-1.08215	1.082149079	0.113899261	-0.1139	0.715467	611	592	522	575	607	604	598	603
wecG	113	34	-56.6667	-1.12811	0.046922	-1.28218	1.282181795	0.35860083	-0.3586	0.149901	489	537	471	499	424	466	437	442.3333
wraB	350	228	-132.333	-1.04185	0.259085	-1.18758	1.187580064	0.24802478	-0.24802	0.470697	3379	3314	3191	3294.667	3372	3086	3029	3162.333
wza	11	6	4	1.307692	0.640609	1.147047	1.147047026	0.197924539	0.197925	0.801621	11	17	11	13	20	10	21	17
wzb	4	1	1	1.333333	1	1.16542	1.165420124	0.220850128	0.22085	1	2	2	5	3	3	3	6	4
wzc	23	12	11.33333	1.290598	0.415971	1.136256	1.136255889	0.184287772	0.184288	0.629498	48	33	36	39	56	46	49	50.33333
wzxC	26	8	15.33333	1.582278	0.05179	1.3948	1.394799528	0.480057781	0.480058	0.159963	28	28	23	26.33333	36	49	40	41.66667
wzxE	359	111	-200.667	-1.36441	0.00072	-1.5612	1.56120018	0.642655534	-0.64266	0.005922	689	834	731	751.3333	578	475	599	550.6667
wzzB	1032	611	679.6667	1.370997	0.182977	1.214792	1.214792146	0.280709486	0.280709	0.371913	2131	1848	1517	1832	2527	2549	2459	2511.667
wzzE	268	120	149	1.086897	0.725546	-1.04841	1.048405907	0.068197387	-0.0682	0.859138	1692	1798	1654	1714.667	1857	1812	1922	1863.667
xapA	14	5	6.333333	2.583333	0.030361	2.249301	2.24930124	1.169476888	1.169477	0.108477	8	1	3	4	8	15	8	10.33333
xapB	48	11	25.66667	1.712963	0.012112	1.514016	1.514015775	0.598380237	0.59838	0.053512	46	41	21	36	69	52	64	61.66667
xapR	80	62	59.66667	2.132911	4.42E-06	1.886027	1.88602659	0.915350016	0.91535	9.23E-05	72	46	40	52.66667	120	109	108	112.3333
xerC	800	209	-445.333	-1.37804	0.001399	-1.57841	1.578411401	0.658473282	-0.65847	0.009915	1385	1900	1585	1623.333	1258	1100	1176	1178
xerD	297	118	138	1.109235	0.842022	-1.02592	1.025917759	0.036915085	-0.03692	0.939636	1358	1240	1192	1263.333	1489	1324	1391	1401.333
xseA	990	303	545.6667	1.549513	0.02294	1.378119	1.378118607	0.462700058	0.4627	0.086709	1252	1044	683	993	1673	1347	1596	1538.667
xseB	264	106	80	1.093422	0.786326	-1.03426	1.034255822	0.048593079	-0.04859	0.898692	994	845	730	856.3333	951	906	952	936.3333
xthA	247	42	-34	-1.02704	0.228977	-1.16425	1.164252012	0.219403375	-0.2194	0.435075	1355	1383	1136	1291.333	1236	1258	1278	1257.333
xylA	99	49	-24.6667	-1.03996	0.165074	-1.18424	1.184235981	0.243956593	-0.24396	0.348971	643	691	592	642	664	594	594	617.3333
xylB	232	103	101.6667	1.201586	0.654015	1.058352	1.058351882	0.081819376	0.081819	0.812887	592	466	455	504.3333	687	562	569	606
xylR	690	461	489.6667	1.484179	0.030555	1.314598	1.314597623	0.394621281	0.394621	0.108832	1191	1010	833	1011.333	1523	1471	1509	1501
yaaA	1444	954	1059	1.746476	0.001946	1.548486	1.548485609	0.630857976	0.630858	0.012828	1761	1353	1142	1418.667	2586	2307	2540	2477.667
yaaH	188	45	31.33333	1.061924	0.61543	-1.06482	1.064818795	0.090607942	-0.09061	0.783838	569	553	396	506	584	508	520	537.3333
yaaI	35	20	23.66667	1.788889	0.003959	1.575097	1.575097312	0.655440963	0.655441	0.022392	39	27	24	30	59	47	55	53.66667
yaaJ	272	67	76	1.089167	0.75877	-1.03909	1.039091647	0.055322905	-0.05532	0.881222	948	899	710	852.3333	935	868	982	928.3333
yaaU	101	73	71.33333	1.425447	0.049068	1.259369	1.259369369	0.332701483	0.332701	0.153873	199	160	144	167.6667	245	233	239	239
yaaY	37	31	14	1.145329	0.959204	1.010306	1.010306045	0.014792385	0.014792	1	119	88	82	96.33333	119	93	119	110.3333
yabB	1098	398	575.6667	1.256498	0.473475	1.114028	1.114027651	0.155785042	0.155785	0.679614	2590	2361	1782	2244.333	2880	2821	2759	2820
yabC	1248	629	711	1.215934	0.645052	1.072928	1.072927877	0.1015531	0.101553	0.804454	3780	3175	2923	3292.667	4171	3804	4036	4003.667
yabF	30	1	8.333333	1.240385	0.641878	1.095051	1.095050507	0.130997413	0.130997	0.802528	40	27	37	34.66667	36	57	36	43
yabI	191	53	-65.3333	-1.13032	0.065326	-1.29468	1.294680398	0.372596001	-0.3726	0.189685	470	661	569	566.6667	503	474	527	501.3333
yabJ	53	26	-4.66667	-1.02991	0.242348	-1.18035	1.180349052	0.239213555	-0.23921	0.452335	129	182	171	160.6667	150	145	173	156
yabK	42	5	19.33333	1.164306	0.839406	1.024929	1.024929094	0.035524105	0.035524	0.937824	123	128	102	117.6667	143	124	144	137
yabN	242	96	116	1.241331	0.43959	1.101097	1.101096845	0.138941365	0.138941	0.651166	578	489	375	480.6667	617	585	588	596.6667

Feature ID	Experiment - Range (original values)	Experiment - IQR (original values)	Experiment - Difference (original values)	Experiment - Fold Change (original values)	EDGE test: yccT NT vs WT NT , tagwise dispersions - P-value	EDGE test: yccT NT vs WT NT , tagwise dispersions - Fold change	yccT NT vs WT NT ABS|FC|	yccT NT vs WT NT Log2FC	yccT NT vs WT NT Log2FC +-	EDGE test: yccT NT vs WT NT , tagwise dispersions - FDR p-value correction	WT NT - WT.1.S22 - Expression values	WT NT - WT.2.S23 - Expression values	WT NT - WT.3.S24 - Expression values	WT NT - Means	yccT NT - yccT.1.S28 - Expression values	yccT NT - yccT.2.S29 - Expression values	yccT NT - yccT.3.S30 - Expression values	yccT NT - Means
yacA	285	67	147.6667	1.099238	0.770852	-1.0397	1.039696613	0.056162607	-0.05616	0.887872	1433	1549	1482	1488	1718	1545	1644	1635.667
yacC	379	126	170.3333	1.232062	0.489343	1.091645	1.09164481	0.126503521	0.126504	0.694071	871	749	582	734	961	877	875	904.3333
yacE	301	219	207	1.384758	0.101176	1.226638	1.226638052	0.294709611	0.29471	0.253568	640	519	455	538	741	756	738	745
yacF	355	235	272.3333	1.331171	0.205346	1.172314	1.172314251	0.229359351	0.229359	0.40388	842	875	750	822.3333	1077	1102	1105	1094.667
yacG	101	35	58	1.264036	0.433449	1.104965	1.104965219	0.144000959	0.144001	0.644422	194	239	226	219.6667	295	277	261	277.6667
yacH	126	31	60	1.3	0.280128	1.144311	1.144310971	0.194479164	0.194479	0.494717	231	200	169	200	295	207	278	260
yacL	373	98	66.33333	1.105347	0.932759	-1.01313	1.013129382	0.018818425	-0.01882	0.989486	792	678	419	629.6667	727	732	629	696
yadB	101	38	47.66667	1.124348	0.93179	-1.01105	1.011052265	0.015857577	-0.01586	0.989398	385	423	342	383.3333	443	412	438	431
yadE	1016	540	641.6667	1.492958	0.038951	1.32533	1.325330232	0.40635188	0.406352	0.130161	1609	1289	1007	1301.667	2023	1829	1978	1943.333
yadF	343	27	133.3333	1.076614	0.698535	-1.05295	1.052946634	0.074432319	-0.07443	0.840861	1845	1839	1537	1740.333	1866	1880	1875	1873.667
yadG	147	77	87.66667	1.250715	0.461596	1.096132	1.096132175	0.132421774	0.132422	0.66967	334	383	332	349.6667	479	422	411	437.3333
yadH	64	32	34	1.137838	0.983678	-1.00377	1.003773055	0.005433124	-0.00543	1	219	275	246	246.6667	283	278	281	280.6667
yadI	688	323	322.3333	1.332874	0.229371	1.1905	1.190500175	0.251567833	0.251568	0.435451	1289	966	650	968.3333	1338	1228	1306	1290.667
yadQ	273	110	99.66667	1.179904	0.727084	1.045886	1.045885506	0.064724928	0.064725	0.860317	692	533	437	554	710	608	643	653.6667
yadR	661	361	392.6667	1.412899	0.097651	1.245463	1.245462986	0.316682147	0.316682	0.246689	1169	789	895	951	1450	1256	1325	1343.667
yadS	391	222	253	1.385867	0.109533	1.221929	1.221928518	0.289159891	0.28916	0.266554	783	596	588	655.6667	979	818	929	908.6667
yaeB	175	30	83	1.186937	0.731893	1.043704	1.043703863	0.061712425	0.061712	0.863883	467	449	416	444	511	479	591	527
yaeC	1332	440	-635.667	-1.21007	0.040462	-1.38024	1.380235438	0.46491438	-0.46491	0.134398	3474	4145	3366	3661.667	3339	2926	2813	3026
yaeD	187	92	-26	-1.03814	0.183377	-1.17659	1.176594793	0.234617557	-0.23462	0.372556	785	740	598	707.6667	738	646	661	681.6667
yaeE	106	21	-37	-1.26812	0.021765	-1.44716	1.447158776	0.533223217	-0.53322	0.083354	131	221	173	175	115	147	152	138
yaeH	1697	327	140.3333	1.024524	0.525006	-1.10932	1.109324545	0.149681503	-0.14968	0.719276	6642	4945	5580	5722.333	6307	5804	5477	5862.667
yaeJ	176	70	89.66667	1.173436	0.785954	1.033795	1.033795363	0.047950636	0.047951	0.898692	580	510	461	517	637	553	630	606.6667
yaeL	1366	587	-417.667	-1.09176	0.179768	-1.25742	1.257421221	0.330468016	-0.33047	0.368093	4260	5094	5554	4969.333	4847	4188	4620	4551.667
yaeP	532	114	178.3333	1.141347	0.919454	1.013809	1.013809207	0.019786171	0.019786	0.982895	1475	1317	993	1261.667	1431	1525	1364	1440
yaeQ	78	25	-31	-1.07116	0.093151	-1.2189	1.218899808	0.285579543	-0.28558	0.239522	494	464	442	466.6667	458	416	433	435.6667
yaeR	172	28	7	1.011878	0.364496	-1.11981	1.119807943	0.163251319	-0.16325	0.581383	614	663	491	589.3333	614	586	589	596.3333
yaeT	3694	1021	-1662	-1.1407	0.117562	-1.30933	1.309332403	0.388831403	-0.38883	0.278805	12871	12985	14566	13474	12714	10872	11850	11812
yafA	1114	729	595	1.275123	0.417412	1.133848	1.13384814	0.181227429	0.181227	0.630816	2750	2021	1717	2162.667	2831	2805	2637	2757.667
yafB	69	15	25	1.122349	0.979183	-1.00634	1.006341148	0.009119459	-0.00912	1	233	216	164	204.3333	224	231	233	229.3333
yafC	100	26	25.66667	1.108146	0.88835	-1.01868	1.018684009	0.026706604	-0.02671	0.966195	261	260	191	237.3333	263	291	235	263
yafD	1196	358	393	1.169689	0.807696	1.038458	1.038458282	0.054443259	0.054443	0.912287	2873	2334	1741	2316	2937	2498	2692	2709
yafE	505	92	10	1.005778	0.379629	-1.12448	1.124481035	0.169259329	-0.16926	0.596079	1953	1791	1448	1730.667	1779	1687	1756	1740.667
yafH	579	259	365	1.677599	0.00487	1.495741	1.495741434	0.580860801	0.580861	0.026187	652	613	351	538.6667	909	872	930	903.6667
yafJ	487	155	271	1.171302	0.812627	1.031741	1.031741258	0.045081215	0.045081	0.916688	1677	1651	1418	1582	1905	1806	1848	1853
yafK	560	280	373	1.42131	0.073621	1.25454	1.254540233	0.327158739	0.327159	0.205036	1042	848	766	885.3333	1326	1128	1321	1258.333
yafS	269	145	135.6667	1.215344	0.550816	1.078717	1.078716936	0.109316339	0.109316	0.737684	765	620	505	630	769	774	754	765.6667

Feature ID	Experiment - Range (original values)	Experiment - IQR (original values)	Experiment - Difference (original values)	Experiment - Fold Change (original values)	EDGE test: yccT NT vs WT NT , tagwise dispersions - P-value	EDGE test: yccT NT vs WT NT , tagwise dispersions - Fold change	yccT NT vs WT NT ABS|FC|	yccT NT vs WT NT Log2FC	yccT NT vs WT NT Log2FC +-	EDGE test: yccT NT vs WT NT , tagwise dispersions - FDR p-value correction	WT NT - WT.1.S22 - Expression values	WT NT - WT.2.S23 - Expression values	WT NT - WT.3.S24 - Expression values	WT NT - Means	yccT NT - yccT.1.S28 - Expression values	yccT NT - yccT.2.S29 - Expression values	yccT NT - yccT.3.S30 - Expression values	yccT NT - Means
yafV	374	39	25	1.02343	0.435678	-1.10445	1.10444852	0.143326174	-0.14333	0.646513	1272	1031	898	1067	1172	1070	1034	1092
yahN	669	316	359	1.422187	0.082093	1.263117	1.263116841	0.336988097	0.336988	0.221068	1082	823	646	850.3333	1315	1139	1174	1209.333
yahO	300	222	198.6667	1.850214	0.004186	1.659795	1.659794868	0.731004952	0.731005	0.023331	375	159	167	233.6667	459	449	389	432.3333
yaiA	97	45	24.66667	1.049598	0.507554	-1.08175	1.081754549	0.113373188	-0.11337	0.707446	553	483	456	497.3333	542	496	528	522
yaiB	849	623	594.6667	2.222755	3.28E-05	1.991301	1.991301346	0.993711563	0.993712	0.000495	722	353	384	486.3333	1007	1202	1034	1081
yaiC	363	178	201.3333	1.40866	0.091454	1.250205	1.250205466	0.322165215	0.322165	0.23684	637	459	382	492.6667	745	632	705	694
yaiE	264	184	129	1.116813	0.913635	-1.01405	1.014053016	0.02013308	-0.02013	0.980345	1252	1065	996	1104.333	1249	1260	1191	1233.333
yaiI	156	41	82	1.219839	0.550891	1.075144	1.075143924	0.104529798	0.10453	0.737684	394	401	324	373	480	450	435	455
yaiU	928	607	639.6667	1.495098	0.044984	1.318252	1.318251868	0.398626041	0.398626	0.144967	1570	1080	1226	1292	2008	1833	1954	1931.667
yaiV	94	52	73	2.710938	2.64E-09	2.400453	2.400453349	1.263306899	1.263307	1.41E-07	50	48	30	42.66667	100	124	123	115.6667
yaiW	132	92	99.33333	1.223221	0.536139	1.077034	1.077033801	0.107063528	0.107064	0.726494	471	450	414	445	545	546	542	544.3333
yaiY	350	196	199	1.468971	0.051763	1.31203	1.312029762	0.391800446	0.3918	0.159963	568	385	320	424.3333	581	670	619	623.3333
yaiZ	234	43	97	1.088936	0.737865	-1.0422	1.04220293	0.059636216	-0.05964	0.868252	1168	1125	979	1090.667	1213	1160	1190	1187.667
yajB	122	59	33.66667	1.133421	0.975719	1.004702	1.004702036	0.006767705	0.006768	1	322	235	200	252.3333	294	257	307	286
yajC	802	194	-446.667	-1.21509	0.034477	-1.39491	1.394905839	0.480167738	-0.48017	0.119165	2275	2504	2791	2523.333	2160	2081	1989	2076.667
yajD	333	244	160.3333	1.206615	0.605947	1.069097	1.069096968	0.096392713	0.096393	0.777315	984	682	662	776	995	926	888	936.3333
yajF	182	73	114	1.161473	0.883404	1.018612	1.018612393	0.026605175	0.026605	0.962948	739	687	692	706	869	765	826	820
yajG	220	93	132	1.239855	0.540364	1.085232	1.08523195	0.118003427	0.118003	0.729763	569	480	602	550.3333	685	700	662	682.3333
yajI	59	9	25.66667	1.278986	0.351976	1.134415	1.134415007	0.181948522	0.181949	0.567129	106	106	64	92	115	123	115	117.6667
yajO	2409	1314	1078.333	1.281794	0.433953	1.144185	1.144184982	0.194320314	0.19432	0.644817	5174	3541	2765	3826.667	5148	4855	4712	4905
yajQ	837	217	-507	-1.38092	0.00197	-1.58815	1.588151951	0.667348953	-0.66735	0.012952	1488	2095	1931	1838	1464	1258	1271	1331
yajR	253	151	193.3333	1.243902	0.496098	1.089048	1.089048315	0.12306796	0.123068	0.699398	782	798	798	792.6667	1035	949	974	986
yaoF	246	129	93.66667	1.25476	0.428924	1.123908	1.12390751	0.168523316	0.168523	0.640136	510	329	264	367.6667	421	505	458	461.3333
ybaB	462	147	-286	-1.34416	0.001894	-1.54218	1.542183435	0.624974377	-0.62497	0.012544	948	1256	1147	1117	898	794	801	831
ybaD	457	282	285	1.390768	0.10357	1.232316	1.232316484	0.301372817	0.301373	0.257387	912	666	610	729.3333	1028	948	1067	1014.333
ybaE	438	132	123	1.153112	0.837399	1.028093	1.028093083	0.039970891	0.039971	0.936875	1030	788	592	803.3333	920	953	906	926.3333
ybaJ	1976	1137	1325.667	2.022628	0.000149	1.810106	1.810105772	0.856074002	0.856074	0.001635	1680	1278	931	1296.333	2415	2907	2544	2622
ybaK	143	27	74	1.089192	0.718283	-1.04542	1.045423376	0.064087323	-0.06409	0.853045	846	847	796	829.6667	939	899	873	903.6667
ybaL	85	59	13	1.020344	0.369809	-1.119	1.119001615	0.162212118	-0.16221	0.58711	610	690	617	639	682	605	669	652
ybaM	24	1	10.66667	1.146789	1	1.007346	1.007345672	0.010558832	0.010559	1	77	65	76	72.66667	89	85	76	83.33333
ybaN	32	8	11	1.147982	0.946121	1.012023	1.012022925	0.017241971	0.017242	0.998395	85	77	61	74.33333	93	77	86	85.33333
ybaO	100	49	40.33333	1.206838	0.587107	1.071747	1.07174671	0.099963989	0.099964	0.76537	243	187	155	195	236	255	215	235.3333
ybaP	307	142	186.3333	1.352237	0.144981	1.194567	1.19456707	0.256487858	0.256488	0.318499	621	518	448	529	755	660	731	715.3333
ybaV	108	66	56	1.767123	0.04209	1.579258	1.579258086	0.659246959	0.659247	0.138271	128	32	59	73	140	122	125	129
ybaW	379	216	207.3333	1.600386	0.035989	1.441456	1.44145603	0.52752683	0.527527	0.12276	528	317	191	345.3333	570	533	555	552.6667
ybaX	206	68	107.3333	1.211564	0.586128	1.067997	1.067997478	0.09490824	0.094908	0.764537	572	504	446	507.3333	652	568	624	614.6667

Feature ID	Experiment - Range (original values)	Experiment - IQR (original values)	Experiment - Difference (original values)	Experiment - Fold Change (original values)	EDGE test: yccT NT vs WT NT , tagwise dispersions - P-value	EDGE test: yccT NT vs WT NT , tagwise dispersions - Fold change	yccT NT vs WT NT ABS|FC|	yccT NT vs WT NT Log2FC	yccT NT vs WT NT Log2FC +-	EDGE test: yccT NT vs WT NT , tagwise dispersions - FDR p-value correction	WT NT - WT.1.S22 - Expression values	WT NT - WT.2.S23 - Expression values	WT NT - WT.3.S24 - Expression values	WT NT - Means	yccT NT - yccT.1.S28 - Expression values	yccT NT - yccT.2.S29 - Expression values	yccT NT - yccT.3.S30 - Expression values	yccT NT - Means
ybaY	793	482	351.3333	1.310823	0.288452	1.169825	1.169825035	0.226292769	0.226293	0.504991	1562	1043	786	1130.333	1579	1341	1525	1481.667
ybaZ	256	101	124.3333	1.318259	0.224101	1.169344	1.169344044	0.225699461	0.225699	0.428125	489	388	295	390.6667	551	463	531	515
ybbA	180	103	95	1.443235	0.067838	1.285666	1.285666473	0.362516428	0.362516	0.194934	296	193	154	214.3333	311	283	334	309.3333
ybbB	460	151	180.6667	1.102983	0.846301	-1.02663	1.026632349	0.037919626	-0.03792	0.942201	1956	1791	1516	1754.333	1976	1887	1942	1935
ybbJ	542	340	313.3333	1.453449	0.064637	1.29329	1.293289739	0.371045522	0.371046	0.18793	933	611	529	691	1071	951	991	1004.333
ybbK	1109	828	654.3333	1.596294	0.036549	1.432439	1.432438881	0.518473583	0.518474	0.124577	1683	855	754	1097.333	1863	1752	1640	1751.667
ybbL	128	98	85.33333	1.393241	0.081735	1.232993	1.232992999	0.302164608	0.302165	0.220833	269	195	187	217	299	293	315	302.3333
ybbM	159	97	103.6667	1.278674	0.311952	1.130119	1.130118749	0.176474374	0.176474	0.526976	420	371	325	372	468	484	475	475.6667
ybbN	306	45	-163	-1.14977	0.038178	-1.31626	1.316260297	0.396444817	-0.39644	0.128269	1141	1328	1285	1251.333	1099	1022	1144	1088.333
ybbO	199	94	149.6667	1.17705	0.79847	1.03275	1.032750274	0.046491443	0.046491	0.906398	861	853	822	845.3333	1017	947	1021	995
ybbP	179	97	118.6667	1.3324	0.188619	1.172687	1.172687274	0.229818335	0.229818	0.37955	407	335	329	357	508	432	487	475.6667
ybbS	245	189	160.6667	1.491335	0.037969	1.329113	1.329113197	0.41046398	0.410464	0.127843	444	290	247	327	492	492	479	487.6667
ybbY	37	10	19.66667	1.460938	0.10166	1.282262	1.282261707	0.358690743	0.358691	0.254208	48	38	42	42.66667	75	52	60	62.33333
ybcI	65	7	17.33333	1.213115	0.637461	1.083242	1.083241505	0.115354922	0.115355	0.799487	108	93	43	81.33333	107	98	91	98.66667
ybcJ	122	70	77	1.273373	0.352721	1.121659	1.12165868	0.165633732	0.165634	0.567661	321	247	277	281.6667	347	360	369	358.6667
ybdA	35	8	2.666667	1.041885	0.529118	-1.09591	1.095913182	0.132133513	-0.13213	0.722725	68	60	63	63.66667	82	47	70	66.33333
ybdB	15	0	0.666667	1.02439	0.653723	-1.10862	1.108619629	0.148764457	-0.14876	0.812751	25	32	25	27.33333	22	25	37	28
ybdD	295	178	187	1.367387	0.137185	1.204691	1.204690717	0.268662808	0.268663	0.30677	603	449	475	509	744	653	691	696
ybdF	71	57	57.33333	1.270866	0.338946	1.118406	1.11840636	0.16144447	0.161444	0.552768	225	207	203	211.6667	269	274	264	269
ybdG	484	291	357.6667	1.556535	0.014865	1.36312	1.363120422	0.446913019	0.446913	0.062611	679	587	662	642.6667	1071	953	977	1000.333
ybdH	134	98	20.33333	1.039028	0.466161	-1.09668	1.096676792	0.133138404	-0.13314	0.674358	503	597	463	521	574	476	574	541.3333
ybdJ	30	19	18	1.335404	0.240835	1.176721	1.176720934	0.234772217	0.234772	0.451455	66	45	50	53.66667	75	69	71	71.66667
ybdK	44	29	31.66667	1.524862	0.024624	1.341618	1.34161778	0.423973714	0.423974	0.091611	70	52	59	60.33333	96	88	92	92
ybdL	217	47	-94.6667	-1.12684	0.059022	-1.28997	1.289966274	0.367333347	-0.36733	0.176111	736	937	850	841	783	736	720	746.3333
ybdM	90	14	19.66667	1.091473	0.7732	-1.037	1.037003456	0.052420702	-0.05242	0.889421	245	231	169	215	259	217	228	234.6667
ybdN	127	118	112	1.685714	0.00079	1.48498	1.48498017	0.570443666	0.570444	0.006328	184	150	156	163.3333	275	277	274	275.3333
ybdO	362	289	264.3333	1.551077	0.01481	1.377175	1.377175159	0.461712063	0.461712	0.062518	616	425	398	479.6667	758	760	714	744
ybdQ	5958	3610	2065.667	1.22422	0.621537	1.094571	1.094570965	0.130365493	0.130365	0.787112	12807	7982	6849	9212.667	11592	11937	10306	11278.33
ybdR	69	36	35.33333	1.369338	0.154492	1.210682	1.210681836	0.275819779	0.27582	0.33247	122	79	86	95.66667	121	124	148	131
ybdZ	2	2	-0.66667	-1.4	0.778265	-1.54426	1.544259798	0.626915485	-0.62692	0.892929	3	3	1	2.333333	1	3	1	1.666667
ybeA	238	62	122	1.135255	0.979773	-1.0034	1.003403592	0.004902008	-0.0049	1	956	894	856	902	1094	941	1037	1024
ybeB	292	125	153	1.153975	0.92099	1.012861	1.012860805	0.018435921	0.018436	0.983597	1055	970	956	993.6667	1248	1095	1097	1146.667
ybeC	703	117	-34	-1.02334	0.295432	-1.18173	1.181733322	0.240904504	-0.2409	0.511951	1397	1186	1889	1490.667	1507	1373	1490	1456.667
ybeD	86	33	-29.6667	-1.05147	0.139768	-1.19598	1.195981529	0.258195109	-0.2582	0.310899	621	638	559	606	592	552	585	576.3333
ybeF	238	136	152.3333	1.90495	0.000191	1.696116	1.696115555	0.762234463	0.762234	0.001987	234	150	121	168.3333	317	286	359	320.6667
ybeJ	72	30	32.66667	1.427948	0.105717	1.268134	1.268134484	0.34270775	0.342708	0.260385	104	74	51	76.33333	123	108	96	109

Feature ID	Experiment - Range (original values)	Experiment - IQR (original values)	Experiment - Difference (original values)	Experiment - Fold Change (original values)	EDGE test: yccT NT vs WT NT , tagwise dispersions - P-value	EDGE test: yccT NT vs WT NT , tagwise dispersions - Fold change	yccT NT vs WT NT ABS|FC|	yccT NT vs WT NT Log2FC	yccT NT vs WT NT Log2FC +-	EDGE test: yccT NT vs WT NT , tagwise dispersions - FDR p-value correction	WT NT - WT.1.S22 - Expression values	WT NT - WT.2.S23 - Expression values	WT NT - WT.3.S24 - Expression values	WT NT - Means	yccT NT - yccT.1.S28 - Expression values	yccT NT - yccT.2.S29 - Expression values	yccT NT - yccT.3.S30 - Expression values	yccT NT - Means
ybeK	3380	165	-1395.67	-2.07912	0.000193	-2.3489	2.348899925	1.231985249	-1.23199	0.001994	2042	4575	1450	2689	1400	1195	1285	1293.333
ybeL	1996	507	-640.667	-1.14336	0.118513	-1.28826	1.288255984	0.365419294	-0.36542	0.280369	5898	5529	3902	5109.667	4689	4536	4182	4469
ybeM	123	66	74.66667	1.668657	0.004044	1.473144	1.473144264	0.55889872	0.558899	0.022798	149	90	96	111.6667	213	162	184	186.3333
ybeQ	557	162	191.3333	1.169371	0.772652	1.039177	1.039176951	0.055441337	0.055441	0.889421	1411	1124	854	1129.667	1407	1286	1270	1321
ybeR	20	12	13.66667	2.171429	0.003307	1.909362	1.909362273	0.933090859	0.933091	0.019615	17	10	8	11.66667	22	26	28	25.33333
ybeS	39	25	27.66667	1.709402	0.003961	1.507464	1.50746409	0.592123636	0.592124	0.022392	48	39	30	39	69	64	67	66.66667
ybeU	23	4	11.33333	1.5	0.184838	1.31085	1.310849768	0.390502352	0.390502	0.373708	22	20	26	22.66667	43	23	36	34
ybeV	58	14	30	1.232558	0.483763	1.088178	1.0881777	0.121914169	0.121914	0.688803	142	140	105	129	163	154	160	159
ybeX	674	272	-195.333	-1.06499	0.205358	-1.2126	1.212595752	0.278098673	-0.2781	0.40388	3278	3409	2916	3201	3188	2735	3094	3005.667
ybeY	449	108	-237.667	-1.2562	0.005721	-1.4283	1.428297878	0.514296891	-0.5143	0.02979	1156	1308	1032	1165.333	1000	924	859	927.6667
ybfA	1415	840	800.3333	1.83426	0.014129	1.663124	1.663123998	0.733895736	0.733896	0.060359	1610	789	479	959.3333	1894	1756	1629	1759.667
ybfE	160	72	76.33333	1.223851	0.503641	1.085975	1.085974926	0.118990793	0.118991	0.704429	414	342	267	341	427	420	405	417.3333
ybfF	135	33	37.66667	1.109073	0.896711	-1.01776	1.017759647	0.025396896	-0.0254	0.97046	378	377	281	345.3333	345	388	416	383
ybfM	657	264	374	1.174332	0.819483	1.033878	1.033877739	0.04806559	0.048066	0.922543	2256	2189	1991	2145.333	2453	2457	2648	2519.333
ybfN	88	17	56	1.215109	0.613745	1.063647	1.063646804	0.089019167	0.089019	0.782388	266	250	265	260.3333	338	282	329	316.3333
ybgC	325	210	233.3333	1.696517	0.00153	1.496495	1.496494882	0.581587345	0.581587	0.010658	406	288	311	335	571	521	613	568.3333
ybgE	2461	1113	1388	1.352076	0.279039	1.189514	1.189513991	0.250372241	0.250372	0.492992	4677	3414	3736	3942.333	5875	4849	5267	5330.333
ybgF	455	247	-32	-1.01747	0.294184	-1.1537	1.153704056	0.206273196	-0.20627	0.510818	2007	2019	1564	1863.333	1930	1683	1881	1831.333
ybgH	179	63	87.66667	1.346966	0.154384	1.189015	1.189015422	0.249767428	0.249767	0.332399	301	245	212	252.6667	391	322	308	340.3333
ybgI	187	102	-39	-1.03341	0.19175	-1.18265	1.182648044	0.242020792	-0.24202	0.384465	1170	1223	1226	1206.333	1284	1097	1121	1167.333
ybgJ	144	17	-10.6667	-1.01422	0.236293	-1.16074	1.160740453	0.215045415	-0.21505	0.444591	755	750	778	761	814	670	767	750.3333
ybgK	284	113	-117.333	-1.10009	0.072002	-1.25806	1.258056725	0.331196974	-0.3312	0.202438	1322	1252	1295	1289.667	1297	1038	1182	1172.333
ybgL	135	53	-74.6667	-1.14053	0.031731	-1.30192	1.301924472	0.380645757	-0.38065	0.111941	630	580	608	606	572	495	527	531.3333
ybgR	235	131	116.3333	1.398857	0.138109	1.254136	1.254135839	0.326693619	0.326694	0.308137	416	278	181	291.6667	409	402	413	408
ybgS	123	84	78.66667	1.708709	0.004988	1.523749	1.523748975	0.607625251	0.607625	0.026598	163	92	78	111	201	192	176	189.6667
ybgT	4160	585	809.3333	1.068757	0.718668	-1.06404	1.064038984	0.089551009	-0.08955	0.853274	13942	9782	11589	11771	13864	12174	11703	12580.33
ybhA	147	43	85.66667	1.1538	0.918173	1.013171	1.013171154	0.018877907	0.018878	0.982488	568	578	525	557	672	611	645	642.6667
ybhB	395	195	277.3333	1.741533	0.0005	1.534525	1.534524926	0.617792081	0.617792	0.004371	438	361	323	374	718	556	680	651.3333
ybhC	642	173	-277	-1.17806	0.042048	-1.35618	1.356181908	0.439550704	-0.43955	0.138271	1513	2026	1959	1832.667	1686	1384	1597	1555.667
ybhE	2202	854	832	1.175232	0.802172	1.041333	1.041332569	0.058430893	0.058431	0.909293	5789	4590	3865	4748	6067	5444	5229	5580
ybhF	329	92	-22	-1.01873	0.250174	-1.15879	1.158787933	0.212616566	-0.21262	0.461231	1183	1368	1039	1196.667	1257	1091	1176	1174.667
ybhK	576	212	277	1.248282	0.428629	1.109155	1.10915498	0.149460965	0.149461	0.640126	1349	1154	844	1115.667	1420	1366	1392	1392.667
ybhL	1314	520	-743.333	-1.29412	0.012887	-1.47529	1.475291995	0.561000526	-0.561	0.056203	3086	3674	3052	3270.667	2690	2532	2360	2527.333
ybhM	121	87	99.33333	2.083636	2.44E-07	1.837795	1.837794536	0.877975484	0.877975	8.13E-06	108	90	77	91.66667	198	177	198	191
ybhN	76	27	35.33333	1.422311	0.097105	1.253157	1.253157047	0.325567226	0.325567	0.246061	105	78	68	83.66667	144	95	118	119
ybhO	41	7	21	1.65625	0.030248	1.471979	1.47197931	0.557757393	0.557757	0.108351	42	35	19	32	42	57	60	53

Feature ID	Experiment - Range (original values)	Experiment - IQR (original values)	Experiment - Difference (original values)	Experiment - Fold Change (original values)	EDGE test: yccT NT vs WT NT , tagwise dispersions - P-value	EDGE test: yccT NT vs WT NT , tagwise dispersions - Fold change	yccT NT vs WT NT ABS|FC|	yccT NT vs WT NT Log2FC	yccT NT vs WT NT Log2FC +-	EDGE test: yccT NT vs WT NT , tagwise dispersions - FDR p-value correction	WT NT - WT.1.S22 - Expression values	WT NT - WT.2.S23 - Expression values	WT NT - WT.3.S24 - Expression values	WT NT - Means	yccT NT - yccT.1.S28 - Expression values	yccT NT - yccT.2.S29 - Expression values	yccT NT - yccT.3.S30 - Expression values	yccT NT - Means
ybhP	18	8	10.66667	1.8	0.048187	1.58433	1.584330223	0.663873069	0.663873	0.152294	19	10	11	13.33333	27	28	17	24
ybhQ	554	165	-297.333	-1.20412	0.014824	-1.36863	1.368627227	0.452729554	-0.45273	0.062518	1920	1735	1607	1754	1562	1366	1442	1456.667
ybhR	144	84	-99.3333	-1.15676	0.024758	-1.31943	1.319429557	0.399914329	-0.39991	0.091957	736	739	724	733	640	595	666	633.6667
ybhS	89	53	-18.6667	-1.05318	0.13197	-1.19715	1.197146803	0.259600077	-0.2596	0.29957	378	410	321	369.6667	379	325	349	351
ybiB	243	73	82.66667	1.079107	0.675587	-1.05345	1.05345013	0.075122018	-0.07512	0.825117	1062	1142	931	1045	1174	1074	1135	1127.667
ybiF	188	137	125	1.470514	0.037763	1.305249	1.305248756	0.384324784	0.384325	0.127443	340	234	223	265.6667	390	411	371	390.6667
ybiH	153	16	58.33333	1.197517	0.646381	1.059204	1.059203796	0.082980197	0.08298	0.805432	323	339	224	295.3333	377	336	348	353.6667
ybiI	45	10	23.66667	1.15368	0.903446	1.016556	1.016555782	0.023689383	0.023689	0.973907	163	157	142	154	179	187	167	177.6667
ybiJ	83	21	37.66667	1.2251	0.537948	1.077102	1.077102264	0.107155232	0.107155	0.727856	169	190	143	167.3333	226	182	207	205
ybiK	61	19	19.33333	1.022481	0.380557	-1.11492	1.114923097	0.156944202	-0.15694	0.596902	863	868	849	860	909	881	848	879.3333
ybiN	209	31	104.6667	1.249206	0.424208	1.101386	1.101386306	0.139320575	0.139321	0.636644	458	449	353	420	562	480	532	524.6667
ybiO	171	65	105	1.269692	0.345887	1.119156	1.11915634	0.162411587	0.162412	0.560375	432	393	343	389.3333	511	458	514	494.3333
ybiP	1281	911	914	1.855805	0.000485	1.65669	1.656690419	0.728304036	0.728304	0.004268	1420	1022	762	1068	1970	1933	2043	1982
ybiR	72	30	30.66667	1.080631	0.679345	-1.05075	1.050745401	0.071413141	-0.07141	0.827568	410	380	351	380.3333	423	411	399	411
ybiS	499	307	322.3333	1.399422	0.092411	1.238716	1.238715745	0.308845162	0.308845	0.238483	978	767	676	807	1175	1074	1139	1129.333
ybiT	152	42	65	1.088798	0.704805	-1.04789	1.04788556	0.067481168	-0.06748	0.845374	727	769	700	732	852	737	802	797
ybiU	58	15	15.66667	1.136628	0.966425	1.008081	1.008081386	0.011612118	0.011612	1	140	122	82	114.6667	140	133	118	130.3333
ybiV(1)	57	43	3	1.008007	0.311394	-1.13436	1.134356298	0.181873857	-0.18187	0.526523	346	403	375	374.6667	389	346	398	377.6667
ybjC	43	19	24.33333	1.424419	0.104383	1.255264	1.25526429	0.327991149	0.327991	0.258535	71	49	52	57.33333	82	71	92	81.66667
ybjD	486	314	303.6667	1.272836	0.351017	1.124597	1.12459743	0.169408655	0.169409	0.566409	1301	1053	985	1113	1471	1412	1367	1416.667
ybjE	79	40	49.33333	1.080566	0.679712	-1.05295	1.05295428	0.074442796	-0.07444	0.827568	628	606	603	612.3333	646	682	657	661.6667
ybjF	148	41	88.66667	1.438221	0.047401	1.26528	1.265279887	0.339456553	0.339457	0.151081	215	211	181	202.3333	292	252	329	291
ybjG	332	234	216.3333	1.411541	0.088007	1.255531	1.255530532	0.328297113	0.328297	0.230881	679	467	431	525.6667	701	762	763	742
ybjM	440	391	253.3333	1.43603	0.110325	1.282725	1.282724601	0.359211459	0.359211	0.26757	854	446	443	581	883	837	783	834.3333
ybjN	59	40	36.66667	1.086751	0.689163	-1.05232	1.052322141	0.073576416	-0.07358	0.834791	411	406	451	422.6667	464	449	465	459.3333
ybjO	108	54	66.33333	1.429806	0.046202	1.265213	1.265213246	0.339380565	0.339381	0.148241	189	151	123	154.3333	226	205	231	220.6667
ybjP	85	40	-7	-1.00841	0.262153	-1.14738	1.147382075	0.198345885	-0.19835	0.474024	878	847	793	839.3333	863	807	827	832.3333
ybjR	361	189	177.6667	1.163347	0.850377	1.024095	1.024095388	0.034350099	0.03435	0.94484	1229	1040	994	1087.667	1355	1162	1279	1265.333
ybjT	166	138	17.33333	1.019817	0.376889	-1.11522	1.11522293	0.15733213	-0.15733	0.594928	909	939	776	874.6667	942	798	936	892
ybjX	1689	1163	1296.667	1.445335	0.113969	1.272458	1.272458251	0.347618323	0.347618	0.273811	3257	2785	2693	2911.667	4382	3948	4295	4208.333
ybjY	524	364	336	1.407273	0.087455	1.247452	1.247451742	0.318984005	0.318984	0.229842	1025	767	683	825	1207	1131	1145	1161
ybjZ	380	199	267.6667	1.308965	0.259492	1.150011	1.150010503	0.201647038	0.201647	0.470847	904	882	813	866.3333	1193	1081	1128	1134
ycaD	292	143	173.6667	1.199617	0.709207	1.050379	1.050378967	0.070909933	0.07091	0.847948	805	974	831	870	1073	961	1097	1043.667
ycaI	129	55	57.66667	1.236016	0.449225	1.097484	1.097484195	0.134200163	0.1342	0.659111	302	247	184	244.3333	313	302	291	302
ycaJ	631	368	70.66667	1.020693	0.483318	-1.11247	1.112465767	0.153760942	-0.15376	0.68865	3635	3606	3004	3415	3624	3238	3595	3485.667
ycaL	141	55	79.66667	1.367692	0.111705	1.212968	1.212967745	0.278541187	0.278541	0.269735	258	227	165	216.6667	306	301	282	296.3333

Feature ID	Experiment - Range (original values)	Experiment - IQR (original values)	Experiment - Difference (original values)	Experiment - Fold Change (original values)	EDGE test: yccT NT vs WT NT , tagwise dispersions - P-value	EDGE test: yccT NT vs WT NT , tagwise dispersions - Fold change	yccT NT vs WT NT ABS|FC|	yccT NT vs WT NT Log2FC	yccT NT vs WT NT Log2FC +-	EDGE test: yccT NT vs WT NT , tagwise dispersions - FDR p-value correction	WT NT - WT.1.S22 - Expression values	WT NT - WT.2.S23 - Expression values	WT NT - WT.3.S24 - Expression values	WT NT - Means	yccT NT - yccT.1.S28 - Expression values	yccT NT - yccT.2.S29 - Expression values	yccT NT - yccT.3.S30 - Expression values	yccT NT - Means
ycaM	38	24	9.333333	1.06278	0.580216	-1.06949	1.069485574	0.096917023	-0.09692	0.759284	153	164	129	148.6667	167	140	167	158
ycaO	172	36	101	1.29735	0.292132	1.137392	1.137391915	0.185729454	0.185729	0.50842	367	331	321	339.6667	493	364	465	440.6667
ycaP	33	10	-2.66667	-1.05479	0.346311	-1.21172	1.211724526	0.277061753	-0.27706	0.560856	31	64	59	51.33333	50	53	43	48.66667
ycaQ	92	7	15.33333	1.062246	0.561776	-1.07878	1.078783756	0.109405703	-0.10941	0.745379	220	276	243	246.3333	312	236	237	261.6667
ycaR	51	15	-21.6667	-1.09985	0.051923	-1.25289	1.252891474	0.325261454	-0.32526	0.160105	245	247	224	238.6667	235	196	220	217
ycbB	1403	518	-35.6667	-1.00868	0.403395	-1.14039	1.140386444	0.189522795	-0.18952	0.617647	4746	4344	3343	4144.333	4357	3826	4143	4108.667
ycbC	376	49	21	1.018601	0.409844	-1.10925	1.109254134	0.149589929	-0.14959	0.624275	1331	1101	955	1129	1150	1107	1193	1150
ycbG	345	200	104	1.106412	0.881088	-1.02007	1.020073131	0.028672585	-0.02867	0.962171	1182	913	837	977.3333	1113	1128	1003	1081.333
ycbK	851	489	554	1.257475	0.56158	1.095764	1.095763843	0.131936905	0.131937	0.745379	2087	1967	2401	2151.667	2818	2576	2723	2705.667
ycbL	703	254	455	1.285028	0.410221	1.122067	1.122067129	0.166158989	0.166159	0.624636	1609	1499	1681	1596.333	2202	1863	2089	2051.333
ycbW	614	428	456	1.992743	1.63E-05	1.766929	1.766929444	0.821244432	0.821244	0.000276	586	413	379	459.3333	912	841	993	915.3333
ycbY	341	138	203.3333	1.340402	0.177177	1.181167	1.181167249	0.24021326	0.240213	0.36497	692	573	527	597.3333	868	711	823	800.6667
yccA	1871	1477	1271.333	1.587311	0.038058	1.409651	1.409650683	0.495337701	0.495338	0.128045	2919	1766	1809	2164.667	3637	3286	3385	3436
yccD	220	96	0.333333	1.000341	0.313881	-1.1339	1.133897026	0.181289628	-0.18129	0.528623	1060	1029	840	976.3333	1057	940	933	976.6667
yccF	68	52	40	1.243902	0.421904	1.100044	1.100043552	0.137560643	0.137561	0.63479	201	149	142	164	210	207	195	204
yccJ	582	73	161.3333	1.063359	0.630722	-1.07776	1.077755063	0.10802934	-0.10803	0.794634	2542	2345	2752	2546.333	2927	2581	2615	2707.667
yccK	209	122	162.6667	1.403974	0.102523	1.228195	1.228194865	0.296539476	0.296539	0.255931	409	374	425	402.6667	582	531	583	565.3333
yccR	199	109	114	1.367347	0.123029	1.214691	1.214691046	0.280589414	0.280589	0.287219	391	302	238	310.3333	425	411	437	424.3333
yccS	405	174	219	1.252014	0.426771	1.104812	1.104812139	0.143801076	0.143801	0.63929	1013	839	755	869	1160	995	1109	1088
yccT	89	59	-74.6667	#DIV/0!	1.13E-51	-638.021	638.0207583	9.317459553	-9.31746	4.53E-49	76	89	59	74.66667	0	0	0	0
yccV	207	72	15.33333	1.020646	0.406196	-1.10941	1.109410699	0.149793544	-0.14979	0.620853	869	697	662	742.6667	810	768	696	758
yccW	81	24	17	1.030723	0.419075	-1.10311	1.103107464	0.141573344	-0.14157	0.632022	566	584	510	553.3333	572	548	591	570.3333
yccX	139	41	65.33333	1.220472	0.53073	1.076037	1.076036513	0.105727034	0.105727	0.723366	335	298	256	296.3333	395	339	351	361.6667
ycdC	95	61	72.33333	1.363484	0.111896	1.201486	1.201485864	0.264819675	0.26482	0.270049	214	202	181	199	276	275	263	271.3333
ycdW	594	224	-452.333	-1.59051	4.14E-05	-1.82919	1.829190081	0.871205001	-0.87121	0.000594	987	1318	1350	1218.333	779	756	763	766
ycdX	275	76	-89.3333	-1.0679	0.13307	-1.21161	1.211611642	0.276927346	-0.27693	0.301153	1541	1408	1266	1405	1364	1288	1295	1315.667
ycdY	267	135	-3.33333	-1.00295	0.292433	-1.14079	1.140791072	0.190034596	-0.19003	0.508745	1209	1177	1015	1133.667	1282	1067	1042	1130.333
ycdZ	501	38	-180.667	-1.29666	0.012765	-1.46897	1.468970339	0.554805266	-0.55481	0.055727	695	1086	588	789.6667	626	616	585	609
yceA	28	21	2.666667	1.033473	0.506365	-1.09794	1.097940922	0.134800428	-0.1348	0.707124	88	87	64	79.66667	88	67	92	82.33333
yceB	882	640	442.3333	1.237091	0.522866	1.098032	1.098031565	0.134919528	0.13492	0.718393	2388	1664	1545	1865.667	2427	2304	2193	2308
yceD	2586	531	-1447	-1.24727	0.034278	-1.43031	1.430306196	0.516324029	-0.51632	0.118683	6294	8162	7441	7299	6217	5576	5763	5852
yceE	212	111	141	1.677885	0.001129	1.486424	1.486424401	0.57184609	0.571846	0.008425	250	212	162	208	374	350	323	349
yceF	222	89	123	1.235332	0.508978	1.085695	1.085694731	0.118618513	0.118619	0.708985	595	465	508	522.6667	687	597	653	645.6667
yceG	164	146	122.6667	1.310811	0.232213	1.156514	1.156513634	0.209782274	0.209782	0.439582	459	358	367	394.6667	517	513	522	517.3333
yceH	1135	97	220.6667	1.052956	0.639258	-1.07585	1.075847817	0.105474018	-0.10547	0.800609	4745	4146	3610	4167	4689	4231	4243	4387.667
yceI	79	26	-24.6667	-1.24916	0.031419	-1.40522	1.405221226	0.490797273	-0.4908	0.111019	144	153	74	123.6667	114	95	88	99

Feature ID	Experiment - Range (original values)	Experiment - IQR (original values)	Experiment - Difference (original values)	Experiment - Fold Change (original values)	EDGE test: yccT NT vs WT NT , tagwise dispersions - P-value	EDGE test: yccT NT vs WT NT , tagwise dispersions - Fold change	yccT NT vs WT NT ABS|FC|	yccT NT vs WT NT Log2FC	yccT NT vs WT NT Log2FC +-	EDGE test: yccT NT vs WT NT , tagwise dispersions - FDR p-value correction	WT NT - WT.1.S22 - Expression values	WT NT - WT.2.S23 - Expression values	WT NT - WT.3.S24 - Expression values	WT NT - Means	yccT NT - yccT.1.S28 - Expression values	yccT NT - yccT.2.S29 - Expression values	yccT NT - yccT.3.S30 - Expression values	yccT NT - Means
yceK	97	61	67.33333	1.448889	0.031159	1.278972	1.278971602	0.354984231	0.354984	0.110344	169	151	130	150	213	212	227	217.3333
yceL	294	163	206.3333	1.633572	0.003709	1.443908	1.443908253	0.529979075	0.529979	0.021344	402	303	272	325.6667	566	466	564	532
yceO	43	13	23.33333	1.546875	0.040905	1.369115	1.36911495	0.45324358	0.453244	0.135565	54	48	26	42.66667	69	61	68	66
yceP	5063	4385	2537	1.268295	0.494363	1.129135	1.129135047	0.175218046	0.175218	0.698064	12562	8101	7705	9456	12486	12768	10725	11993
ycfC	183	68	110	1.267857	0.407974	1.108962	1.108961735	0.149209586	0.14921	0.622069	414	405	413	410.6667	588	481	493	520.6667
ycfD	420	110	184.3333	1.144916	0.952268	1.007923	1.007923488	0.011386127	0.011386	1	1395	1285	1136	1272	1556	1338	1475	1456.333
ycfF	213	80	-108	-1.11942	0.062221	-1.28395	1.283949622	0.360588597	-0.36059	0.18307	927	1022	1088	1012.333	959	875	879	904.3333
ycfH	118	77	-27.6667	-1.07028	0.137041	-1.23239	1.232391042	0.301460101	-0.30146	0.306682	344	458	462	421.3333	432	355	394	393.6667
ycfJ	550	156	307	1.465387	0.053668	1.303257	1.303256828	0.382121419	0.382121	0.164229	732	752	495	659.6667	888	1045	967	966.6667
ycfL	99	41	-44.3333	-1.06355	0.120838	-1.21445	1.214454632	0.280308597	-0.28031	0.283748	713	777	736	742	728	678	687	697.6667
ycfM	313	6	-168.667	-1.16574	0.033223	-1.33646	1.336459645	0.418416276	-0.41842	0.115641	1037	1283	1239	1186.333	1040	970	1043	1017.667
ycfN	110	6	-45.6667	-1.08354	0.094003	-1.2359	1.235897513	0.305559112	-0.30556	0.240628	554	650	573	592.3333	552	540	548	546.6667
ycfP	2674	739	-1770.33	-1.50877	0.001688	-1.73224	1.732240727	0.792639433	-0.79264	0.011545	4184	6100	5466	5250	3568	3426	3445	3479.667
ycfQ	642	301	401.3333	1.356424	0.161369	1.19989	1.199890228	0.262902427	0.262902	0.342609	1261	1194	923	1126	1565	1495	1522	1527.333
ycfR	8	5	2	1.133333	1	-1.00249	1.002490641	0.003588768	-0.00359	1	18	14	13	15	20	12	19	17
ycfS	36	12	14	1.13125	1	-1.00138	1.001382099	0.001992571	-0.00199	1	124	106	90	106.6667	126	118	118	120.6667
ycfU	284	190	192.6667	1.339003	0.181553	1.17773	1.177729649	0.236008403	0.236008	0.370207	636	529	540	568.3333	813	730	740	761
ycfV	161	46	77	1.223188	0.615882	1.067029	1.067028531	0.093598753	0.093599	0.784022	341	327	367	345	488	387	391	422
ycfW	146	34	92	1.147043	0.969144	1.00548	1.005479518	0.007883693	0.007884	1	633	627	617	625.6667	729	661	763	717.6667
ycfX	297	101	104.3333	1.105815	0.833057	-1.02684	1.026837895	0.038208443	-0.03821	0.933318	1104	969	885	986	1182	1019	1070	1090.333
ycgB	1218	256	-723.667	-1.26213	0.019689	-1.43237	1.432366833	0.518401018	-0.5184	0.077083	3558	3835	3060	3484.333	2861	2804	2617	2760.667
ycgL	450	236	303.3333	1.40319	0.088592	1.23759	1.237590401	0.307533912	0.307534	0.231868	853	722	682	752.3333	1132	1077	958	1055.667
ycgM	1118	652	714	1.403466	0.123477	1.243187	1.243186937	0.31404325	0.314043	0.287506	2142	1709	1458	1769.667	2576	2361	2514	2483.667
ycgN	486	250	287	1.361917	0.139558	1.205213	1.205212516	0.269287561	0.269288	0.310589	956	766	657	793	1143	1016	1081	1080
ycgO	806	4	298	1.145059	0.932173	1.012645	1.012645152	0.018128718	0.018129	0.989486	2250	2249	1664	2054.333	2470	2334	2253	2352.333
ycgQ	276	145	128.6667	1.21326	0.567815	1.074298	1.074298109	0.103394385	0.103394	0.748361	732	587	491	603.3333	767	681	748	732
ycgR	424	70	194	1.260636	0.406038	1.116986	1.116986411	0.159611634	0.159612	0.620853	818	865	550	744.3333	974	888	953	938.3333
ychA	327	208	243.6667	1.491925	0.041972	1.303436	1.303436009	0.382319756	0.38232	0.138271	492	457	537	495.3333	784	733	700	739
ychE	249	146	143.3333	1.415058	0.078166	1.255196	1.255195656	0.327912265	0.327912	0.214594	441	319	276	345.3333	525	476	465	488.6667
ychF	306	100	109.3333	1.053006	0.56406	-1.08589	1.085894818	0.118884368	-0.11888	0.745911	2080	1994	2114	2062.667	2300	2014	2202	2172
ychH	1298	268	-263.667	-1.07503	0.21906	-1.21254	1.212536744	0.278028467	-0.27803	0.422448	4319	3993	3021	3777.667	3596	3607	3339	3514
ychJ	136	101	107.6667	1.202762	0.660635	1.056239	1.056239168	0.078936547	0.078937	0.81699	554	513	526	531	649	640	627	638.6667
ychK	434	168	-253.333	-1.09647	0.141233	-1.24782	1.247818609	0.31940823	-0.31941	0.313373	3016	2869	2753	2879.333	2711	2582	2585	2626
ychM	1147	161	297.3333	1.112385	0.929044	-1.01432	1.014315387	0.020506307	-0.02051	0.98743	2957	3010	1970	2645.667	3117	2796	2916	2943
ychN	124	85	45	1.087719	0.748157	-1.04083	1.040832095	0.057737354	-0.05774	0.876079	590	483	466	513	568	585	521	558
ychP	302	88	72.33333	1.078481	0.717911	-1.04793	1.047925512	0.067536172	-0.06754	0.853045	1021	1021	723	921.6667	1024	933	1025	994

Feature ID	Experiment - Range (original values)	Experiment - IQR (original values)	Experiment - Difference (original values)	Experiment - Fold Change (original values)	EDGE test: yccT NT vs WT NT , tagwise dispersions - P-value	EDGE test: yccT NT vs WT NT , tagwise dispersions - Fold change	yccT NT vs WT NT ABS|FC|	yccT NT vs WT NT Log2FC	yccT NT vs WT NT Log2FC +-	EDGE test: yccT NT vs WT NT , tagwise dispersions - FDR p-value correction	WT NT - WT.1.S22 - Expression values	WT NT - WT.2.S23 - Expression values	WT NT - WT.3.S24 - Expression values	WT NT - Means	yccT NT - yccT.1.S28 - Expression values	yccT NT - yccT.2.S29 - Expression values	yccT NT - yccT.3.S30 - Expression values	yccT NT - Means
yciA	81	44	49.66667	1.647826	0.004759	1.456336	1.456335936	0.542343183	0.542343	0.025804	100	68	62	76.66667	143	124	112	126.3333
yciB	329	168	231.3333	1.316317	0.256915	1.15134	1.151339556	0.203313379	0.203313	0.469052	727	731	736	731.3333	1056	899	933	962.6667
yciC	473	165	301.6667	1.285219	0.32497	1.129973	1.129973431	0.176288851	0.176289	0.539103	1150	1054	969	1057.667	1442	1219	1417	1359.333
yciE	21	2	9	1.818182	0.059899	1.605957	1.605956602	0.683432907	0.683433	0.177651	13	15	5	11	15	26	19	20
yciF	13	9	9.666667	2.380952	0.00946	2.077464	2.077464271	1.054823665	1.054824	0.044642	5	9	7	7	16	18	16	16.66667
yciG	15	5	8	1.774194	0.074891	1.556552	1.55655202	0.638353792	0.638354	0.207531	13	6	12	10.33333	17	21	17	18.33333
yciH	66	21	32.33333	1.557471	0.038833	1.369131	1.369131468	0.453260985	0.453261	0.129864	56	75	43	58	109	77	85	90.33333
yciI	239	37	29.33333	1.021907	0.412628	-1.11403	1.11402688	0.155784043	-0.15578	0.625818	1457	1218	1342	1339	1404	1369	1332	1368.333
yciK	462	136	243.3333	1.145854	0.938933	1.010459	1.010458753	0.015010431	0.01501	0.993427	1761	1737	1507	1668.333	1893	1969	1873	1911.667
yciL	325	37	176.3333	1.140244	0.996232	1.000909	1.000909497	0.00131153	0.001312	1	1302	1285	1185	1257.333	1510	1322	1469	1433.667
yciM	609	244	306.6667	1.208098	0.622789	1.066059	1.066058805	0.09228702	0.092287	0.787112	1666	1466	1289	1473.667	1898	1733	1710	1780.333
yciN	190	27	-42.6667	-1.03998	0.178581	-1.18659	1.186589839	0.246821334	-0.24682	0.366508	1079	1183	1068	1110	1157	1052	993	1067.333
yciO	432	111	227.6667	1.171522	0.815269	1.030571	1.03057128	0.043444294	0.043444	0.918821	1397	1378	1207	1327.333	1639	1489	1537	1555
yciR	409	278	267.3333	1.598954	0.00884	1.420693	1.420692663	0.506594492	0.506594	0.042166	591	399	349	446.3333	758	677	706	713.6667
yciS	449	155	222.6667	1.212671	0.587955	1.069646	1.069645738	0.097133061	0.097133	0.76537	1175	1048	918	1047	1367	1239	1203	1269.667
yciT	1008	663	668.6667	1.46597	0.055434	1.299606	1.299606436	0.378074793	0.378075	0.167664	1761	1370	1174	1435	2182	2033	2096	2103.667
yciW	78	38	44.66667	1.439344	0.064434	1.274489	1.274489084	0.349919016	0.349919	0.187585	123	94	88	101.6667	141	166	132	146.3333
ycjE	28	14	-5	-1.05338	0.170424	-1.20836	1.208356246	0.273045851	-0.27305	0.356034	85	99	112	98.66667	105	84	92	93.66667
ycjF	182	80	62.33333	1.090821	0.705643	-1.04997	1.049971078	0.070349589	-0.07035	0.845636	649	729	681	686.3333	821	639	786	748.6667
ycjG	147	10	53.66667	1.067961	0.59712	-1.06898	1.068975264	0.096228469	-0.09623	0.772181	819	741	809	789.6667	888	814	828	843.3333
ycjI	135	92	60.33333	1.076275	0.656961	-1.05624	1.056236332	0.078932673	-0.07893	0.814931	865	760	748	791	883	819	852	851.3333
ycjX	350	100	192	1.168077	0.868088	1.022025	1.022024815	0.031430226	0.03143	0.955415	1100	1185	1142	1142.333	1450	1311	1242	1334.333
ydaA	5867	1011	403.6667	1.025316	0.570736	-1.10016	1.100157824	0.137710502	-0.13771	0.750362	18994	15714	13127	15945	16891	16583	15572	16348.67
ydaL	194	33	65	1.080313	0.701089	-1.04832	1.048318209	0.068076702	-0.06808	0.84302	874	848	706	809.3333	882	900	841	874.3333
ydaO	141	60	93	1.398003	0.07026	1.232479	1.232478629	0.301562631	0.301563	0.1993	263	236	202	233.6667	341	296	343	326.6667
ydbH	261	96	159	1.307345	0.242677	1.151849	1.151848817	0.203951372	0.203951	0.452759	557	543	452	517.3333	713	639	677	676.3333
ydbL	53	21	35.66667	1.363946	0.143351	1.196947	1.196947186	0.259359496	0.259359	0.315855	104	94	96	98	147	117	137	133.6667
ydcF	236	77	-81.3333	-1.13864	0.041254	-1.28715	1.287148914	0.364178973	-0.36418	0.136501	771	698	535	668	615	538	607	586.6667
ydcI	473	101	235	1.378829	0.143421	1.217662	1.217662291	0.284114069	0.284114	0.315855	653	744	464	620.3333	937	754	875	855.3333
ydcK	90	53	54.33333	1.206853	0.588187	1.064832	1.064831655	0.090625365	0.090625	0.76537	299	254	235	262.6667	319	307	325	317
ydcN	82	44	25	1.101626	0.831523	-1.02733	1.027330242	0.03890002	-0.0389	0.932307	296	228	214	246	281	260	272	271
ydcP	315	263	252.3333	1.6056	0.004915	1.416197	1.416197384	0.502022357	0.502022	0.026336	489	380	381	416.6667	695	644	668	669
ydcR	123	43	72	1.209709	0.612106	1.063843	1.06384341	0.089285812	0.089286	0.78182	350	353	327	343.3333	393	403	450	415.3333
ydcW	153	74	66	1.255155	0.399955	1.109124	1.109124468	0.149421276	0.149421	0.615134	319	245	212	258.6667	365	289	320	324.6667
ydcX	137	23	28.66667	1.064131	0.599122	-1.06616	1.066157329	0.092420347	-0.09242	0.773381	483	476	382	447	519	455	453	475.6667
ydcY	69	39	28.66667	1.2134	0.56956	1.073331	1.073331299	0.102095453	0.102095	0.749798	168	125	110	134.3333	164	146	179	163

Feature ID	Experiment - Range (original values)	Experiment - IQR (original values)	Experiment - Difference (original values)	Experiment - Fold Change (original values)	EDGE test: yccT NT vs WT NT , tagwise dispersions - P-value	EDGE test: yccT NT vs WT NT , tagwise dispersions - Fold change	yccT NT vs WT NT ABS|FC|	yccT NT vs WT NT Log2FC	yccT NT vs WT NT Log2FC +-	EDGE test: yccT NT vs WT NT , tagwise dispersions - FDR p-value correction	WT NT - WT.1.S22 - Expression values	WT NT - WT.2.S23 - Expression values	WT NT - WT.3.S24 - Expression values	WT NT - Means	yccT NT - yccT.1.S28 - Expression values	yccT NT - yccT.2.S29 - Expression values	yccT NT - yccT.3.S30 - Expression values	yccT NT - Means
ydcZ	176	111	111.6667	1.360215	0.12855	1.204445	1.204445456	0.268369062	0.268369	0.295746	379	289	262	310	438	427	400	421.6667
yddE	250	119	111.3333	1.11261	0.88102	-1.0193	1.019299398	0.027577875	-0.02758	0.962171	1108	964	894	988.6667	1144	1083	1073	1100
yddG	54	44	45.66667	1.362434	0.126817	1.197905	1.197905223	0.260513768	0.260514	0.293127	133	122	123	126	176	172	167	171.6667
yddX	94	26	49	1.325942	0.194068	1.174559	1.17455914	0.232119356	0.232119	0.388046	170	155	126	150.3333	181	220	197	199.3333
ydeA	161	119	117.6667	1.729339	0.001297	1.535054	1.535054372	0.618289757	0.61829	0.009372	212	134	138	161.3333	257	285	295	279
ydeD	84	30	43.66667	1.208931	0.612587	1.062671	1.062670595	0.087694461	0.087694	0.782014	231	201	195	209	279	231	248	252.6667
ydeE	336	214	187.6667	1.344553	0.176267	1.196413	1.196413147	0.258715669	0.258716	0.364166	711	506	417	544.6667	753	724	720	732.3333
ydeI	42	28	13	1.22807	0.624699	1.09243	1.092429883	0.127540685	0.127541	0.788712	82	49	40	57	81	77	52	70
ydeJ	26	6	12	1.514286	0.155471	1.3241	1.324099913	0.405011988	0.405012	0.33296	16	30	24	23.33333	42	30	34	35.33333
ydeV	16925	11014	11341.33	4.07381	0.005589	3.806717	3.806717052	1.92854734	1.928547	0.029202	9607	752	710	3689.667	17635	11766	15692	15031
ydeW	9096	6650	6270.333	3.693828	0.002825	3.430851	3.430851174	1.778566545	1.778567	0.017196	5674	652	657	2327.667	9748	7307	8739	8598
ydeY	6344	4047	4377.667	4.907468	0.013782	4.618238	4.618237602	2.2073424	2.207342	0.059119	3095	150	116	1120.333	6460	4197	5837	5498
ydeZ	8302	5333	5828.667	5.185256	0.004151	4.873845	4.873844812	2.285060315	2.28506	0.023225	3802	214	162	1392.667	8464	5547	7653	7221.333
ydfG	1686	1273	-1321.67	-1.38759	0.003565	-1.58288	1.582878308	0.662550345	-0.66255	0.020759	4845	4805	4545	4731.667	3799	3272	3159	3410
ydfH	646	84	214	1.137121	0.977364	1.003909	1.00390887	0.005628315	0.005628	1	1751	1624	1307	1560.667	1953	1708	1663	1774.667
ydfI	123	75	89.66667	1.47695	0.027421	1.299477	1.299477206	0.377931327	0.377931	0.100002	216	168	180	188	287	255	291	277.6667
ydfZ	274	103	153.3333	1.551559	0.073007	1.352968	1.35296766	0.436127355	0.436127	0.204355	304	180	350	278	407	433	454	431.3333
ydgA	1191	197	-574.333	-1.32412	0.007804	-1.51922	1.519220112	0.60333091	-0.60333	0.038253	1915	2824	2300	2346.333	1940	1633	1743	1772
ydgC	34	9	12.33333	1.303279	0.441898	1.149452	1.149451553	0.200945661	0.200946	0.652901	54	28	40	40.66667	49	48	62	53
ydgH	1089	196	-580.333	-1.13901	0.10276	-1.30635	1.30635128	0.385542892	-0.38554	0.256379	4373	4777	5115	4755	4321	4026	4177	4174.667
ydgI	55	26	27	1.26129	0.38256	1.117362	1.117362247	0.160096981	0.160097	0.598772	129	103	78	103.3333	133	127	131	130.3333
ydgJ	612	183	271.6667	1.160813	0.857474	1.024497	1.024497334	0.03491623	0.034916	0.948917	1887	1720	1461	1689.333	2073	1903	1907	1961
ydgK	240	76	133.3333	1.225479	0.516876	1.082885	1.082885114	0.114880191	0.11488	0.715467	648	632	494	591.3333	732	708	734	724.6667
ydgR	1106	570	752.6667	1.222178	0.646119	1.073275	1.073274733	0.102019419	0.102019	0.805331	3673	3220	3270	3387.667	4326	3840	4255	4140.333
ydgT	121	66	80.66667	1.4	0.123183	1.226269	1.226269156	0.294275674	0.294276	0.287275	204	176	225	201.6667	270	297	280	282.3333
ydhA	326	128	179.6667	1.30332	0.249733	1.154653	1.154652844	0.207459158	0.207459	0.460953	695	602	480	592.3333	806	780	730	772
ydhB	51	19	28	1.226415	0.500694	1.082886	1.082885636	0.114880888	0.114881	0.70364	137	131	103	123.6667	151	150	154	151.6667
ydhC	128	89	96.33333	1.236691	0.541511	1.081223	1.081222729	0.112663744	0.112664	0.730644	398	387	436	407	508	487	515	503.3333
ydhD	2497	962	-1457	-1.86606	8.67E-06	-2.12397	2.123969767	1.086763231	-1.08676	0.000166	2726	4108	2584	3139.333	1814	1622	1611	1682.333
ydhE	566	436	421.3333	2.016895	2.95E-05	1.789948	1.789948261	0.839917886	0.839918	0.000458	563	341	339	414.3333	905	777	825	835.6667
ydhF	831	229	-481	-1.23114	0.022737	-1.39943	1.399429531	0.484838841	-0.48484	0.086181	2571	2830	2285	2562	2188	2056	1999	2081
ydhH	606	523	436.6667	1.452661	0.057745	1.28698	1.286979862	0.363989479	0.363989	0.172883	1225	835	834	964.6667	1440	1358	1406	1401.333
ydhI	58	40	37	1.351266	0.151272	1.191789	1.191789484	0.253129423	0.253129	0.327447	126	96	94	105.3333	152	139	136	142.3333
ydhJ	95	69	70.33333	1.353434	0.127108	1.195015	1.195014815	0.257028504	0.257029	0.293493	226	192	179	199	261	273	274	269.3333
ydhL	22	4	8.666667	1.376812	0.316159	1.209292	1.209292391	0.274163111	0.274163	0.531248	26	24	19	23	41	22	32	31.66667
ydhM	145	31	24	1.065395	0.622363	-1.06256	1.062555371	0.087538023	-0.08754	0.787112	425	383	293	367	438	352	383	391

Feature ID	Experiment - Range (original values)	Experiment - IQR (original values)	Experiment - Difference (original values)	Experiment - Fold Change (original values)	EDGE test: yccT NT vs WT NT , tagwise dispersions - P-value	EDGE test: yccT NT vs WT NT , tagwise dispersions - Fold change	yccT NT vs WT NT ABS|FC|	yccT NT vs WT NT Log2FC	yccT NT vs WT NT Log2FC +-	EDGE test: yccT NT vs WT NT , tagwise dispersions - FDR p-value correction	WT NT - WT.1.S22 - Expression values	WT NT - WT.2.S23 - Expression values	WT NT - WT.3.S24 - Expression values	WT NT - Means	yccT NT - yccT.1.S28 - Expression values	yccT NT - yccT.2.S29 - Expression values	yccT NT - yccT.3.S30 - Expression values	yccT NT - Means
ydhO	168	42	81	1.18465	0.790673	1.036283	1.036282512	0.051417365	0.051417	0.901795	451	388	477	438.6667	510	493	556	519.6667
ydiA	779	491	366.6667	1.289398	0.341625	1.147832	1.147831977	0.198911471	0.198911	0.556113	1719	1113	969	1267	1748	1549	1604	1633.667
ydiB	41	23	26.66667	1.588235	0.015736	1.395675	1.395674686	0.480962707	0.480963	0.06541	51	41	44	45.33333	82	67	67	72
ydiD	425	264	230	1.195081	0.671649	1.054967	1.054966659	0.077197406	0.077197	0.82303	1378	1114	1045	1179	1470	1337	1420	1409
ydiE	37	6	-15.6667	-1.53409	0.005768	-1.7284	1.728395552	0.789433423	-0.78943	0.029964	48	62	25	45	32	26	30	29.33333
ydiF	22	5	9	1.290323	0.443581	1.138066	1.138065842	0.186584026	0.186584	0.654515	38	33	22	31	44	37	39	40
ydiH	299	61	146.3333	1.143887	0.996831	-1.00067	1.000666952	0.000961887	-0.00096	1	1041	908	1102	1017	1196	1087	1207	1163.333
ydiJ	2578	1410	1699.333	1.380675	0.212465	1.218104	1.218103636	0.284636883	0.284637	0.413271	5198	4256	3938	4464	6516	5666	6308	6163.333
ydiK	295	178	178.6667	1.328633	0.186558	1.174618	1.174617791	0.232191394	0.232191	0.376087	641	526	464	543.6667	759	704	704	722.3333
ydiL	29	12	13.66667	1.41	0.188834	1.245836	1.24583587	0.317114016	0.317114	0.37964	45	33	22	33.33333	51	43	47	47
ydiM	59	28	40	2.333333	0.000115	2.073089	2.073088507	1.051781711	1.051782	0.001321	44	21	25	30	53	80	77	70
ydiN	26	11	15.33333	3	0.000548	2.636533	2.636532729	1.398641906	1.398642	0.004715	16	2	5	7.666667	28	25	16	23
ydiO	99	48	52.33333	1.496835	0.04877	1.336609	1.33660915	0.418577656	0.418578	0.153262	147	102	67	105.3333	150	157	166	157.6667
ydiP	91	50	57.66667	1.703252	0.001276	1.50547	1.505470122	0.590214077	0.590214	0.009284	101	83	62	82	153	133	133	139.6667
ydiQ	52	25	22.66667	1.250923	0.474697	1.101623	1.101622951	0.139630521	0.139631	0.680486	112	74	85	90.33333	126	103	110	113
ydiR	25	18	19	1.508929	0.048968	1.328428	1.328427578	0.409719579	0.40972	0.153776	42	37	33	37.33333	58	55	56	56.33333
ydiS	52	11	28.33333	1.364807	0.149073	1.20183	1.201829861	0.265232673	0.265233	0.324649	88	78	67	77.66667	110	89	119	106
ydiT	9	3	4.666667	1.424242	0.401362	1.252956	1.252955624	0.32533532	0.325335	0.616499	13	11	9	11	15	18	14	15.66667
ydiU	187	10	-105.333	-1.27288	0.002753	-1.44258	1.442576471	0.528647798	-0.52865	0.016776	522	547	405	491.3333	403	360	395	386
ydiV	92	50	67.66667	1.692833	0.001818	1.483342	1.48334176	0.568851031	0.568851	0.012207	95	97	101	97.66667	162	147	187	165.3333
ydiY	235	116	153	1.898239	5.1E-05	1.680137	1.680136883	0.748578776	0.748579	0.000698	212	163	136	170.3333	320	279	371	323.3333
ydiZ	435	235	259.6667	1.343323	0.173809	1.188852	1.188852373	0.249569578	0.24957	0.361571	919	700	650	756.3333	1085	1028	935	1016
ydjA	281	50	-141.667	-1.23123	0.008078	-1.39962	1.399624328	0.485039647	-0.48504	0.039279	731	875	657	754.3333	637	594	607	612.6667
ydjM	55	23	31.33333	1.333333	0.187638	1.179067	1.179066733	0.237645374	0.237645	0.37792	108	96	78	94	119	133	124	125.3333
ydjN	374	206	235	1.480573	0.02901	1.308434	1.308434155	0.387841324	0.387841	0.104678	578	479	410	489	784	685	703	724
yeaA	253	77	-46	-1.05418	0.168678	-1.20536	1.205355907	0.269459196	-0.26946	0.353697	777	1030	878	895	835	801	911	849
yeaB	112	59	74.66667	1.308116	0.238479	1.148139	1.148138658	0.199296884	0.199297	0.447945	261	225	241	242.3333	337	300	314	317
yeaC	273	30	26.33333	1.035142	0.47049	-1.10664	1.106636117	0.146180915	-0.14618	0.677524	614	887	747	749.3333	797	753	777	775.6667
yeaD	2292	1098	-1663.67	-1.82333	9.98E-06	-2.09162	2.091624433	1.064623828	-1.06462	0.000183	3709	3121	4223	3684.333	2023	1931	2108	2020.667
yeaG	1100	787	598.6667	1.766866	0.022899	1.595733	1.595733057	0.67421933	0.674219	0.086649	1353	538	451	780.6667	1551	1262	1325	1379.333
yeaH	972	614	604.6667	1.428234	0.089208	1.270073	1.270072531	0.344910888	0.344911	0.232655	1836	1302	1098	1412	2070	1916	2064	2016.667
yeaJ	331	269	267.3333	1.709735	0.000732	1.509981	1.509981187	0.594530575	0.594531	0.005982	426	359	345	376.6667	628	676	628	644
yeaK	114	43	53	1.147907	0.996648	-1.00073	1.000726552	0.001047813	-0.00105	1	330	351	394	358.3333	435	355	444	411.3333
yeaL	112	36	60.33333	1.285489	0.291469	1.130682	1.130681929	0.177193143	0.177193	0.508353	227	216	191	211.3333	303	260	252	271.6667
yeaM	112	39	61.33333	1.411633	0.068026	1.251015	1.25101493	0.323099007	0.323099	0.195264	178	161	108	149	220	200	211	210.3333
yeaN	123	68	78.66667	1.503198	0.018264	1.331677	1.331676511	0.413243667	0.413244	0.072908	196	152	121	156.3333	244	220	241	235

Feature ID	Experiment - Range (original values)	Experiment - IQR (original values)	Experiment - Difference (original values)	Experiment - Fold Change (original values)	EDGE test: yccT NT vs WT NT , tagwise dispersions - P-value	EDGE test: yccT NT vs WT NT , tagwise dispersions - Fold change	yccT NT vs WT NT ABS|FC|	yccT NT vs WT NT Log2FC	yccT NT vs WT NT Log2FC +-	EDGE test: yccT NT vs WT NT , tagwise dispersions - FDR p-value correction	WT NT - WT.1.S22 - Expression values	WT NT - WT.2.S23 - Expression values	WT NT - WT.3.S24 - Expression values	WT NT - Means	yccT NT - yccT.1.S28 - Expression values	yccT NT - yccT.2.S29 - Expression values	yccT NT - yccT.3.S30 - Expression values	yccT NT - Means
yeaO	402	129	196.6667	1.280418	0.336821	1.127743	1.127742723	0.173437976	0.173438	0.550703	809	680	615	701.3333	1017	804	873	898
yeaQ	83	37	38.33333	1.257848	0.37751	1.113593	1.113593498	0.155222693	0.155223	0.594928	183	146	117	148.6667	200	183	178	187
yeaR	21	5	12.66667	1.666667	0.05086	1.464327	1.464327082	0.55023784	0.550238	0.158038	21	20	16	19	33	25	37	31.66667
yeaS	518	394	429	1.402691	0.104554	1.229621	1.229620802	0.298213477	0.298213	0.25867	1090	1037	1069	1065.333	1555	1465	1463	1494.333
yeaZ	268	92	141	1.22681	0.521734	1.080475	1.080474777	0.111665394	0.111665	0.717998	675	638	552	621.6667	820	730	738	762.6667
yebA	2099	1416	1461.333	1.518632	0.069125	1.339465	1.339464916	0.421656794	0.421657	0.1971	3448	2321	2684	2817.667	4420	4100	4317	4279
yebB	40	11	15	1.16791	0.904565	1.022101	1.022100945	0.031537687	0.031538	0.974265	71	104	93	89.33333	105	111	97	104.3333
yebC	829	166	-381	-1.19233	0.053035	-1.37471	1.374714509	0.459132041	-0.45913	0.162854	1967	2421	2698	2362	2120	1869	1954	1981
yebE	293	163	181	1.399853	0.107326	1.235259	1.235258703	0.304813221	0.304813	0.262887	564	379	415	452.6667	672	578	651	633.6667
yebF	132	21	12	1.04461	0.570591	-1.07642	1.076419659	0.106240644	-0.10624	0.750362	343	253	211	269	274	295	274	281
yebG	61	32	-7	-1.04595	0.177095	-1.1844	1.184401416	0.244158121	-0.24416	0.36497	185	169	124	159.3333	176	137	144	152.3333
yebK	915	412	-638.333	-1.38749	0.00125	-1.5767	1.576698994	0.656907263	-0.65691	0.009143	2251	2543	2063	2285.667	1651	1663	1628	1647.333
yebN	327	145	212.3333	1.504355	0.019693	1.328914	1.328914304	0.410248075	0.410248	0.077083	458	466	339	421	666	603	631	633.3333
yebR	172	64	-33	-1.02896	0.209312	-1.17209	1.172094714	0.229089155	-0.22909	0.4102	1159	1259	1099	1172.333	1168	1087	1163	1139.333
yebS	171	52	52	1.134251	0.965502	1.005799	1.005798587	0.008341432	0.008341	1	466	401	295	387.3333	453	406	459	439.3333
yebU	90	53	58	1.191419	0.693678	1.048973	1.048972681	0.068977105	0.068977	0.837317	303	332	274	303	356	364	363	361
yebW	36	26	17.66667	1.630952	0.088746	1.460435	1.460434923	0.546398073	0.546398	0.232036	49	19	16	28	45	52	40	45.66667
yecA	2417	1103	699.3333	1.082088	0.788102	-1.04479	1.044790578	0.063213792	-0.06321	0.900252	9859	8257	7442	8519.333	9360	9447	8849	9218.667
yecC	154	70	90.66667	1.201481	0.661709	1.055825	1.055825046	0.078370794	0.078371	0.817313	440	506	404	450	558	510	554	540.6667
yecD	204	135	103.6667	1.241835	0.475386	1.096988	1.096987885	0.133547593	0.133548	0.681253	532	359	395	428.6667	563	530	504	532.3333
yecE	154	127	100.3333	1.411202	0.082224	1.251718	1.251717972	0.323909541	0.32391	0.221211	318	208	206	244	338	335	360	344.3333
yecF	87	31	4.666667	1.0076	0.319861	-1.13661	1.136610426	0.184737855	-0.18474	0.534832	576	619	647	614	663	605	588	618.6667
yecG	1611	772	536.6667	1.262343	0.47613	1.132735	1.13273498	0.179810361	0.17981	0.682098	2948	1852	1337	2045.667	2682	2624	2441	2582.333
yecH	175	71	108.6667	1.285464	0.350896	1.124314	1.124314224	0.169045297	0.169045	0.566409	368	399	375	380.6667	543	446	479	489.3333
yecM	36	21	-6.66667	-1.04902	0.16989	-1.19886	1.198856625	0.261659132	-0.26166	0.355254	126	153	149	142.6667	147	144	117	136
yecN	224	170	156	1.531215	0.01608	1.357921	1.357921424	0.4414	0.4414	0.066342	376	262	243	293.6667	450	432	467	449.6667
yecO	288	233	217.3333	1.342797	0.164414	1.185005	1.185004891	0.244893013	0.244893	0.347907	719	609	574	634	862	850	842	851.3333
yecP	148	44	92	1.166969	0.8759	1.020494	1.020494471	0.029268366	0.029268	0.959152	544	549	560	551	692	593	644	643
yecS	188	35	95.66667	1.206326	0.613056	1.061998	1.06199755	0.086780438	0.08678	0.782388	497	485	409	463.6667	597	520	561	559.3333
yedA	207	67	99.33333	1.341743	0.167286	1.189629	1.189628859	0.25051155	0.250512	0.351801	361	294	217	290.6667	424	353	393	390
yedD	93	26	-50.6667	-1.13831	0.039676	-1.3041	1.304096046	0.383050127	-0.38305	0.132383	385	418	448	417	385	359	355	366.3333
yedE	225	42	-118	-1.58033	0.002658	-1.83779	1.837792456	0.87797385	-0.87797	0.016402	172	395	397	321.3333	204	182	224	203.3333
yedF	95	18	-49.6667	-2.02055	0.000532	-2.34111	2.341107301	1.22719106	-1.22719	0.004615	50	108	137	98.33333	42	61	43	48.66667
yedI	170	59	85.66667	1.188003	0.695463	1.048475	1.048475063	0.068292549	0.068293	0.838074	512	453	402	455.6667	572	548	504	541.3333
yedJ	119	72	61.66667	1.279035	0.303214	1.133084	1.1330835	0.180254182	0.180254	0.518605	276	204	183	221	278	268	302	282.6667
yedO	493	84	198	1.200676	0.643119	1.06048	1.060479531	0.084716774	0.084717	0.803497	1118	1034	808	986.6667	1301	1106	1147	1184.667

Feature ID	Experiment - Range (original values)	Experiment - IQR (original values)	Experiment - Difference (original values)	Experiment - Fold Change (original values)	EDGE test: yccT NT vs WT NT , tagwise dispersions - P-value	EDGE test: yccT NT vs WT NT , tagwise dispersions - Fold change	yccT NT vs WT NT ABS|FC|	yccT NT vs WT NT Log2FC	yccT NT vs WT NT Log2FC +-	EDGE test: yccT NT vs WT NT , tagwise dispersions - FDR p-value correction	WT NT - WT.1.S22 - Expression values	WT NT - WT.2.S23 - Expression values	WT NT - WT.3.S24 - Expression values	WT NT - Means	yccT NT - yccT.1.S28 - Expression values	yccT NT - yccT.2.S29 - Expression values	yccT NT - yccT.3.S30 - Expression values	yccT NT - Means
yedP	45	34	31	1.392405	0.107872	1.227908	1.227908378	0.296202916	0.296203	0.263932	97	66	74	79	111	108	111	110
yeeA	434	177	224.3333	1.239161	0.473069	1.095654	1.095653785	0.131791994	0.131792	0.679471	1101	925	788	938	1222	1102	1163	1162.333
yeeF	347	149	245.6667	1.428488	0.0758	1.251062	1.251061722	0.323152968	0.323153	0.209394	595	545	580	573.3333	836	729	892	819
yeeI	2292	1801	1289.667	1.416828	0.182856	1.26536	1.265360387	0.339548337	0.339548	0.371904	4427	2523	2332	3094	4624	4324	4203	4383.667
yeeN	463	125	-19.6667	-1.00889	0.348987	-1.15502	1.155022014	0.207920349	-0.20792	0.56416	2246	1995	2458	2233	2291	2228	2121	2213.333
yeeO	183	74	96.33333	1.460191	0.04391	1.292802	1.292801502	0.37050078	0.370501	0.142538	270	196	162	209.3333	345	267	305	305.6667
yeeX	706	291	137.6667	1.03936	0.562382	-1.09309	1.093091216	0.128413796	-0.12841	0.745608	3662	3660	3171	3497.667	3877	3660	3369	3635.333
yeeY	368	144	155	1.213695	0.564564	1.078774	1.078774198	0.10939292	0.109393	0.745911	894	734	548	725.3333	916	878	847	880.3333
yeeZ	733	214	198	1.141294	0.912039	1.015286	1.015285817	0.021885923	0.021886	0.979221	1779	1379	1046	1401.333	1668	1537	1593	1599.333
yegB	90	14	37	1.197509	0.65487	1.055024	1.055023995	0.077275811	0.077276	0.813265	209	195	158	187.3333	248	198	227	224.3333
yegD	60	27	-2	-1.0176	0.291632	-1.15916	1.15916101	0.213080974	-0.21308	0.508353	121	132	94	115.6667	144	84	113	113.6667
yegE	367	67	169.3333	1.204674	0.616923	1.066047	1.066047401	0.092271588	0.092272	0.78418	903	917	662	827.3333	991	970	1029	996.6667
yegH	259	149	125.3333	1.173672	0.766807	1.037198	1.037198044	0.052691391	0.052691	0.886011	847	698	620	721.6667	879	804	858	847
yegN	156	49	80	1.404722	0.084153	1.239891	1.239891357	0.310213713	0.310214	0.224491	241	192	160	197.6667	316	234	283	277.6667
yegO	77	30	18.66667	1.075168	0.639233	-1.05576	1.0557569	0.078277676	-0.07828	0.800609	281	248	216	248.3333	293	239	269	267
yegQ	214	104	136.6667	1.365745	0.137893	1.201485	1.201485129	0.264818792	0.264819	0.3079	421	345	355	373.6667	513	459	559	510.3333
yegS	194	135	145	1.725	0.00077	1.53171	1.531710249	0.615143411	0.615143	0.006195	251	185	164	200	320	358	357	345
yegT	84	35	45.33333	1.356955	0.13185	1.20094	1.200939512	0.264163488	0.264163	0.29957	151	126	104	127	161	168	188	172.3333
yegU	109	33	42.33333	1.171159	0.762845	1.036136	1.036136023	0.051213411	0.051213	0.883198	290	257	195	247.3333	304	269	296	289.6667
yegV	106	5	48.33333	1.209235	0.582751	1.066447	1.066447116	0.092812426	0.092812	0.761737	252	250	191	231	297	255	286	279.3333
yegW	239	141	102	1.176064	0.752079	1.040766	1.040765974	0.057645701	0.057646	0.878462	718	531	489	579.3333	728	644	672	681.3333
yehE	104	73	82	2.629139	6.19E-09	2.326033	2.326033383	1.217871802	1.217872	3.08E-07	67	40	44	50.33333	117	136	144	132.3333
yehR	92	40	60.33333	2.064706	1.29E-05	1.824449	1.824448846	0.867460701	0.867461	0.000226	65	66	39	56.66667	115	105	131	117
yehS	149	31	55.66667	1.101335	0.798595	-1.03175	1.031752696	0.045097208	-0.0451	0.906398	578	585	485	549.3333	634	554	627	605
yehT	132	57	77	1.295396	0.357203	1.132108	1.132107838	0.179011388	0.179011	0.572592	220	292	270	260.6667	327	334	352	337.6667
yehU	152	33	46.33333	1.100798	0.813395	-1.03107	1.031065234	0.044135612	-0.04414	0.917321	455	520	404	459.6667	474	488	556	506
yehV	108	89	60.33333	1.331502	0.225453	1.182547	1.182547494	0.241898126	0.241898	0.43004	252	144	150	182	251	237	239	242.3333
yehW	51	14	8.333333	1.046211	0.518922	-1.0854	1.085403051	0.118230869	-0.11823	0.716307	172	210	159	180.3333	176	186	204	188.6667
yehX	60	14	29.33333	1.263473	0.393445	1.111195	1.111194897	0.152111879	0.152112	0.61	114	124	96	111.3333	156	128	138	140.6667
yehY	32	8	-2.66667	-1.01905	0.200219	-1.15635	1.15635376	0.209582825	-0.20958	0.3973	160	140	128	142.6667	146	133	141	140
yehZ	64	21	31.66667	1.248691	0.395907	1.103262	1.10326166	0.141774995	0.141775	0.61207	145	132	105	127.3333	155	153	169	159
yeiA	676	347	287	1.184605	0.770459	1.041803	1.041803115	0.059082655	0.059083	0.887872	1479	1885	1300	1554.667	1976	1723	1826	1841.667
yeiB	233	19	105.3333	1.262677	0.377317	1.11482	1.114819661	0.156810352	0.15681	0.594928	448	435	320	401	553	454	512	506.3333
yeiE	676	179	97.33333	1.062688	0.664738	-1.06098	1.060977379	0.085393896	-0.08539	0.819548	1949	1436	1273	1552.667	1734	1601	1615	1650
yeiG	656	234	-334.333	-1.20689	0.017996	-1.37907	1.379070066	0.463695757	-0.4637	0.072165	1883	2109	1859	1950.333	1770	1453	1625	1616
yeiH	215	83	112	1.267516	0.347022	1.123958	1.123957942	0.168588052	0.168588	0.561804	490	438	328	418.6667	528	521	543	530.6667

Feature ID	Experiment - Range (original values)	Experiment - IQR (original values)	Experiment - Difference (original values)	Experiment - Fold Change (original values)	EDGE test: yccT NT vs WT NT , tagwise dispersions - P-value	EDGE test: yccT NT vs WT NT , tagwise dispersions - Fold change	yccT NT vs WT NT ABS|FC|	yccT NT vs WT NT Log2FC	yccT NT vs WT NT Log2FC +-	EDGE test: yccT NT vs WT NT , tagwise dispersions - FDR p-value correction	WT NT - WT.1.S22 - Expression values	WT NT - WT.2.S23 - Expression values	WT NT - WT.3.S24 - Expression values	WT NT - Means	yccT NT - yccT.1.S28 - Expression values	yccT NT - yccT.2.S29 - Expression values	yccT NT - yccT.3.S30 - Expression values	yccT NT - Means
yeiR	417	106	62.66667	1.044901	0.537713	-1.08473	1.084734024	0.117341339	-0.11734	0.727856	1481	1560	1146	1395.667	1563	1375	1437	1458.333
yeiU	88	45	3.333333	1.006075	0.314879	-1.13091	1.130912472	0.177487275	-0.17749	0.529699	568	571	507	548.6667	595	538	523	552
yejA	349	25	145	1.13146	0.988446	-1.00212	1.002123927	0.003060929	-0.00306	1	1176	1188	945	1103	1294	1201	1249	1248
yejB	118	23	30.33333	1.065942	0.6013	-1.06454	1.064539315	0.090229231	-0.09023	0.775425	496	484	400	460	518	465	488	490.3333
yejE	98	34	20.66667	1.058601	0.568101	-1.07092	1.070918175	0.098848253	-0.09885	0.748515	380	384	294	352.6667	392	347	381	373.3333
yejF	526	132	-239	-1.26575	0.005657	-1.44776	1.447758494	0.533820961	-0.53382	0.029508	1088	1272	1055	1138.333	1029	746	923	899.3333
yejG	283	24	-109.333	-1.21257	0.014889	-1.38273	1.382725542	0.467514823	-0.46751	0.062627	561	755	555	623.6667	540	472	531	514.3333
yejH	268	44	48	1.0553	0.573751	-1.07336	1.073360974	0.10213534	-0.10214	0.752825	920	976	708	868	963	876	909	916
yejK	640	271	359	1.224937	0.557798	1.081469	1.081469247	0.112992641	0.112993	0.742446	1820	1572	1396	1596	1986	1843	2036	1955
yejL	281	171	161.3333	1.414739	0.092755	1.260649	1.26064895	0.334166587	0.334167	0.238952	512	341	314	389	512	595	544	550.3333
yejM	717	334	397.3333	1.395357	0.10139	1.235171	1.235170624	0.304710347	0.30471	0.253789	1234	963	818	1005	1535	1297	1375	1402.333
yfaE	32	16	-8.66667	-1.04483	0.132176	-1.18747	1.187465314	0.247885372	-0.24789	0.299576	206	216	184	202	202	192	186	193.3333
yfaO	238	118	-104.667	-1.08662	0.091341	-1.23932	1.239319947	0.309548686	-0.30955	0.236823	1401	1217	1321	1313	1290	1163	1172	1208.333
yfaV	99	25	-34.3333	-1.07785	0.102993	-1.23071	1.230710677	0.299491644	-0.29949	0.256724	437	525	464	475.3333	461	436	426	441
yfaW	436	151	200.3333	1.193061	0.66255	1.05731	1.057310325	0.080398876	0.080399	0.81776	1208	1057	848	1037.667	1283	1284	1147	1238
yfaX	101	31	-33.6667	-1.13289	0.044924	-1.27814	1.278144444	0.354050886	-0.35405	0.144967	328	306	227	287	257	267	236	253.3333
yfaZ	79	67	60	1.318584	0.201685	1.161798	1.161798272	0.216359589	0.21636	0.398959	212	175	178	188.3333	246	245	254	248.3333
yfbB	1063	286	-618.333	-1.58096	9.76E-05	-1.81203	1.812033608	0.857609714	-0.85761	0.00116	1335	2080	1633	1682.667	1127	1017	1049	1064.333
yfbE	719	569	461	1.380049	0.160614	1.225456	1.225456423	0.293319183	0.293319	0.341618	1600	976	1063	1213	1632	1695	1695	1674
yfbG	877	277	-314.667	-1.10716	0.133253	-1.25507	1.255068546	0.32776616	-0.32777	0.301153	3656	3263	2834	3251	3111	2779	2919	2936.333
yfbK	902	145	217.6667	1.144501	0.907403	1.018115	1.018114506	0.025899829	0.0259	0.976372	1733	1820	966	1506.333	1868	1588	1716	1724
yfbQ	381	231	283.3333	1.601557	0.004708	1.408954	1.408953918	0.494624427	0.494624	0.025626	529	452	432	471	813	683	767	754.3333
yfbS	607	152	156.6667	1.090177	0.79166	-1.03628	1.036283348	0.051418528	-0.05142	0.902424	2058	1703	1451	1737.333	1985	1855	1842	1894
yfbT	544	182	-94.6667	-1.02722	0.303652	-1.16925	1.169253792	0.225588107	-0.22559	0.51909	3809	3546	3364	3573	3691	3265	3479	3478.333
yfbU	437	75	100.3333	1.04292	0.56127	-1.08839	1.088387238	0.122191945	-0.12219	0.745379	2568	2314	2131	2337.667	2542	2383	2389	2438
yfcA	98	20	60	1.3	0.260998	1.142063	1.142063371	0.191642705	0.191643	0.472806	213	203	184	200	275	223	282	260
yfcB	201	83	126.6667	1.297572	0.312894	1.136813	1.136812993	0.184994949	0.184995	0.527563	413	438	426	425.6667	534	509	614	552.3333
yfcC	890	58	-279.333	-1.83466	0.005472	-2.06708	2.067084457	1.047597336	-1.0476	0.028696	430	1151	261	614	355	297	352	334.6667
yfcE	409	228	267.3333	1.491723	0.026015	1.31909	1.319089665	0.399542635	0.399543	0.0959	655	522	454	543.6667	863	750	820	811
yfcF	307	166	148.6667	1.262199	0.374416	1.120506	1.12050613	0.164150541	0.164151	0.592301	715	542	444	567	751	688	708	715.6667
yfcG	57	28	30.66667	1.442308	0.074334	1.280448	1.280448113	0.356648793	0.356649	0.206495	92	68	48	69.33333	96	99	105	100
yfcH	1111	683	508	1.394716	0.17883	1.249663	1.249662704	0.32153875	0.321539	0.36668	1909	1084	868	1287	1979	1767	1639	1795
yfcL	109	5	-43	-1.14191	0.034301	-1.30507	1.30506952	0.38412666	-0.38413	0.118683	312	388	338	346	313	279	317	303
yfcM	51	3	-3.33333	-1.01325	0.212339	-1.15689	1.156887049	0.210248016	-0.21025	0.413271	260	251	254	255	276	225	254	251.6667
yfcN	268	98	94.33333	1.112929	0.873422	-1.01988	1.019875541	0.028393106	-0.02839	0.958007	944	820	742	835.3333	1010	861	918	929.6667
yfcX	1326	617	779.3333	1.323285	0.297994	1.172254	1.172254034	0.229285244	0.229285	0.513591	2839	2440	1953	2410.667	3279	3057	3234	3190

Feature ID	Experiment - Range (original values)	Experiment - IQR (original values)	Experiment - Difference (original values)	Experiment - Fold Change (original values)	EDGE test: yccT NT vs WT NT , tagwise dispersions - P-value	EDGE test: yccT NT vs WT NT , tagwise dispersions - Fold change	yccT NT vs WT NT ABS|FC|	yccT NT vs WT NT Log2FC	yccT NT vs WT NT Log2FC +-	EDGE test: yccT NT vs WT NT , tagwise dispersions - FDR p-value correction	WT NT - WT.1.S22 - Expression values	WT NT - WT.2.S23 - Expression values	WT NT - WT.3.S24 - Expression values	WT NT - Means	yccT NT - yccT.1.S28 - Expression values	yccT NT - yccT.2.S29 - Expression values	yccT NT - yccT.3.S30 - Expression values	yccT NT - Means
yfcY	444	95	215.3333	1.360894	0.16101	1.209592	1.209592492	0.27452109	0.274521	0.342012	733	639	418	596.6667	862	734	840	812
yfcZ	1109	385	-618.333	-1.17638	0.060453	-1.33982	1.339816301	0.42203521	-0.42204	0.178817	4115	4415	3842	4124	3754	3457	3306	3505.667
yfdC	54	24	16.66667	1.147929	0.880697	1.018896	1.018896105	0.02700695	0.027007	0.962171	143	106	89	112.6667	121	137	130	129.3333
yfdH	31	18	21	2.615385	0.00014	2.283182	2.283181771	1.191045721	1.191046	0.001561	14	7	18	13	38	32	32	34
yfdZ	2245	617	313.6667	1.099651	0.8826	-1.02759	1.027586379	0.039259671	-0.03926	0.962777	2864	4412	2167	3147.667	3481	3366	3537	3461.333
yfeA	254	199	193	1.581325	0.007542	1.397365	1.397364519	0.482708414	0.482708	0.037173	406	295	295	332	532	494	549	525
yfeC	393	101	124	1.077226	0.701917	-1.0512	1.051201562	0.072039324	-0.07204	0.843099	1782	1646	1389	1605.667	1753	1689	1747	1729.667
yfeD	462	182	261	1.164669	0.845301	1.026067	1.026067085	0.037125059	0.037125	0.941797	1689	1637	1429	1585	1891	1819	1828	1846
yfeH	104	35	56.66667	1.131274	0.977633	-1.00398	1.003977996	0.005727651	-0.00573	1	462	438	395	431.6667	499	473	493	488.3333
yfeJ	84	30	12	1.034318	0.425786	-1.09848	1.098475803	0.135503091	-0.1355	0.638248	389	347	313	349.6667	397	329	359	361.6667
yfeK	74	23	-20	-1.10363	0.078473	-1.26867	1.268669774	0.343316594	-0.34332	0.215036	189	214	236	213	212	162	205	193
yfeN	22	14	5.333333	1.225352	0.722583	1.08708	1.087079993	0.120458106	0.120458	0.856087	38	16	17	23.66667	35	31	21	29
yfeR	36	9	20.66667	1.397436	0.13951	1.231253	1.231253292	0.300127582	0.300128	0.310589	60	54	42	52	78	63	77	72.66667
yfeZ	87	42	4.333333	1.014874	0.371145	-1.12854	1.128537805	0.174454748	-0.17445	0.58839	255	318	301	291.3333	286	259	342	295.6667
yffB	94	32	53.33333	1.080241	0.666087	-1.05689	1.056892908	0.0798292	-0.07983	0.819919	634	681	679	664.6667	715	728	711	718
yffH	99	60	71	1.251179	0.457389	1.096373	1.096372695	0.132738304	0.132738	0.665853	268	296	284	282.6667	350	344	367	353.6667
yfgA	2144	1551	1210	1.323616	0.328771	1.174659	1.174658754	0.232241705	0.232242	0.542565	4852	3301	3064	3739	5208	4653	4986	4949
yfgB	498	318	383.3333	1.401957	0.086225	1.234392	1.234391543	0.303800082	0.3038	0.228418	1025	966	870	953.6667	1368	1284	1359	1337
yfgD	279	77	78.66667	1.082144	0.705099	-1.04888	1.048876976	0.068845472	-0.06885	0.845374	1092	917	864	957.6667	1143	994	972	1036.333
yfgE	246	93	147.3333	1.146116	0.982526	1.003195	1.003195371	0.004602597	0.004603	1	1007	1009	1009	1008.333	1253	1102	1112	1155.667
yfgJ	30	20	14.33333	1.240223	0.539962	1.092128	1.092127855	0.127141761	0.127142	0.729442	74	51	54	59.66667	81	61	80	74
yfgL	399	167	122	1.07388	0.64431	-1.06462	1.064616592	0.090333956	-0.09033	0.804434	1737	1540	1677	1651.333	1939	1570	1811	1773.333
yfgM	403	113	116.6667	1.081452	0.666511	-1.05867	1.058669023	0.082251623	-0.08225	0.820118	1475	1362	1460	1432.333	1739	1336	1572	1549
yfhA	424	112	267.3333	1.218946	0.604201	1.069228	1.069227862	0.096569337	0.096569	0.777131	1267	1235	1161	1221	1585	1347	1533	1488.333
yfhB	692	290	222.6667	1.231944	0.525132	1.100736	1.100735818	0.138468257	0.138468	0.719276	1362	848	670	960	1283	1127	1138	1182.667
yfhC	211	52	33	1.081214	0.766454	-1.04065	1.040648761	0.057483213	-0.05748	0.886011	517	396	306	406.3333	453	417	448	439.3333
yfhD	141	68	69.33333	1.292958	0.266799	1.146926	1.146925645	0.197771865	0.197772	0.479394	298	230	182	236.6667	323	293	302	306
yfhF	185	57	72.33333	1.047724	0.524946	-1.08652	1.086515178	0.119708328	-0.11971	0.719276	1584	1505	1458	1515.667	1643	1559	1562	1588
yfhG	368	190	244	1.304746	0.257528	1.150217	1.150217104	0.201906196	0.201906	0.469398	908	778	716	800.6667	1082	968	1084	1044.667
yfhH	75	8	27.66667	1.170431	0.77313	1.034668	1.034668427	0.049168511	0.049169	0.889421	184	178	125	162.3333	200	176	194	190
yfhJ	100	23	-50.6667	-1.15606	0.021265	-1.31893	1.318931168	0.399369276	-0.39937	0.081693	383	379	364	375.3333	341	283	350	324.6667
yfhK	617	383	404.6667	1.641649	0.010041	1.459932	1.459932117	0.545901289	0.545901	0.046492	883	519	490	630.6667	1107	902	1097	1035.333
yfhL	199	112	125.3333	1.474149	0.029974	1.307916	1.307915929	0.387269809	0.38727	0.10763	329	259	205	264.3333	394	371	404	389.6667
yfhP	1471	888	894.6667	1.616303	0.024514	1.433685	1.433685355	0.519728436	0.519728	0.091355	1994	1078	1283	1451.667	2549	2319	2171	2346.333
yfiA	45766	4588	-24181	-1.32555	0.024161	-1.4966	1.4965959	0.581684728	-0.58168	0.090496	103690	113197	78491	98459.33	75408	79996	67431	74278.33
yfiC	83	31	26	1.038633	0.443738	-1.09835	1.098345852	0.135332408	-0.13533	0.654528	678	686	655	673	732	649	716	699

Feature ID	Experiment - Range (original values)	Experiment - IQR (original values)	Experiment - Difference (original values)	Experiment - Fold Change (original values)	EDGE test: yccT NT vs WT NT , tagwise dispersions - P-value	EDGE test: yccT NT vs WT NT , tagwise dispersions - Fold change	yccT NT vs WT NT ABS|FC|	yccT NT vs WT NT Log2FC	yccT NT vs WT NT Log2FC +-	EDGE test: yccT NT vs WT NT , tagwise dispersions - FDR p-value correction	WT NT - WT.1.S22 - Expression values	WT NT - WT.2.S23 - Expression values	WT NT - WT.3.S24 - Expression values	WT NT - Means	yccT NT - yccT.1.S28 - Expression values	yccT NT - yccT.2.S29 - Expression values	yccT NT - yccT.3.S30 - Expression values	yccT NT - Means
yfiD	10351	1458	-5238.33	-1.67586	0.004264	-1.92608	1.926080917	0.945668313	-0.94567	0.023616	14718	7022	17227	12989	7896	8480	6876	7750.667
yfiE	160	65	69.33333	1.235828	0.458346	1.097369	1.097369258	0.134049066	0.134049	0.666973	365	290	227	294	355	348	387	363.3333
yfiF	650	137	-378.333	-1.32226	0.003537	-1.51961	1.519613216	0.603704164	-0.6037	0.020624	1327	1706	1624	1552.333	1276	1056	1190	1174
yfiH	676	271	254.3333	1.178063	0.759112	1.041826	1.041825646	0.059113857	0.059114	0.881222	1730	1375	1180	1428.333	1856	1546	1646	1682.667
yfiK	78	6	-25	-1.35885	0.028789	-1.58083	1.580833516	0.66068544	-0.66069	0.104048	68	74	142	94.66667	74	71	64	69.66667
yfiM	53	8	-15.6667	-1.05054	0.131103	-1.19745	1.19744876	0.259963923	-0.25996	0.298366	336	320	321	325.6667	313	283	334	310
yfiN	515	386	402	1.734023	0.000566	1.535086	1.535085842	0.618319334	0.618319	0.004823	654	533	456	547.6667	971	919	959	949.6667
yfiO	385	204	-145	-1.05018	0.234887	-1.20175	1.20175305	0.265140465	-0.26514	0.442888	2978	3008	3118	3034.667	3125	2740	2804	2889.667
yfiP	124	69	96	1.279612	0.348752	1.119984	1.119983955	0.163478064	0.163478	0.564062	339	342	349	343.3333	463	411	444	439.3333
yfiQ	2679	769	1267.333	1.149785	0.935865	1.013081	1.01308051	0.018748831	0.018749	0.990876	9300	8531	7552	8461	10231	9244	9710	9728.333
yfiR	559	348	421.6667	1.640831	0.002341	1.450052	1.450051612	0.536104251	0.536104	0.014628	752	670	552	658	1110	1018	1111	1079.667
yfjB	662	427	388.3333	1.402001	0.10584	1.248499	1.248498711	0.320194332	0.320194	0.260542	1279	877	742	966	1404	1355	1304	1354.333
yfjD	315	39	158.3333	1.203426	0.646885	1.057372	1.057372048	0.080483094	0.080483	0.805833	834	795	706	778.3333	1021	827	962	936.6667
yfjF	360	129	166	1.192056	0.689348	1.051216	1.051216368	0.072059644	0.07206	0.834791	987	858	748	864.3333	1108	935	1048	1030.333
yfjG	708	304	332.3333	1.244603	0.459908	1.104322	1.104322016	0.143160918	0.143161	0.668359	1658	1347	1071	1358.667	1779	1651	1643	1691
ygaA	221	40	-50.3333	-1.12032	0.082304	-1.27935	1.279353519	0.355414974	-0.35541	0.221291	394	598	414	468.6667	444	377	434	418.3333
ygaC	824	601	612.3333	1.85641	0.000271	1.643501	1.643501317	0.716772613	0.716773	0.002574	917	597	631	715	1329	1232	1421	1327.333
ygaD	89	31	-13	-1.0283	0.204514	-1.1778	1.177799455	0.236093912	-0.23609	0.403114	464	441	512	472.3333	472	423	483	459.3333
ygaE	125	94	61	1.407572	0.145376	1.25271	1.252710027	0.325052504	0.325053	0.31905	224	113	112	149.6667	237	188	207	210.6667
ygaF	58	25	37.33333	1.973913	0.000364	1.748026	1.748025665	0.805726367	0.805726	0.003329	47	38	30	38.33333	63	88	76	75.66667
ygaM	221	131	135	1.576923	0.009099	1.398965	1.398965442	0.484360324	0.48436	0.043167	306	212	184	234	405	359	343	369
ygaP	511	256	247.6667	1.375632	0.143044	1.224618	1.224617641	0.29233137	0.292331	0.315494	879	623	476	659.3333	987	838	896	907
ygaU	78	43	46.66667	1.636364	0.007932	1.45051	1.45051006	0.536560301	0.53656	0.038749	101	64	55	73.33333	133	120	107	120
ygbA	128	14	-74	-1.49115	0.001846	-1.72202	1.722015791	0.784098373	-0.7841	0.012334	143	270	261	224.6667	157	153	142	150.6667
ygbD	85	48	35.66667	1.26817	0.385772	1.128028	1.12802845	0.173803455	0.173803	0.602364	183	118	98	133	175	166	165	168.6667
ygbE	62	45	40	1.659341	0.010495	1.472447	1.472446804	0.558215513	0.558216	0.047853	85	47	50	60.66667	95	109	98	100.6667
ygbF	23	2	7.666667	1.036918	0.434316	-1.09637	1.096365064	0.132728262	-0.13273	0.64514	212	212	199	207.6667	214	222	210	215.3333
ygbI	167	33	97	1.126083	0.92748	-1.01201	1.012005261	0.01721679	-0.01722	0.98624	782	743	783	769.3333	874	910	815	866.3333
ygbJ	2002	207	-997.667	-2.17419	7.61E-05	-2.5341	2.534097876	1.341472248	-1.34147	0.000952	1013	1895	2634	1847.333	855	1062	632	849.6667
ygbK	1850	203	-788.667	-1.5918	0.00199	-1.84894	1.848937477	0.88669644	-0.8867	0.013063	1555	1845	2964	2121.333	1352	1532	1114	1332.667
ygbL	584	43	-235.333	-1.25487	0.018695	-1.44381	1.44380561	0.529876514	-0.52988	0.074161	1117	927	1432	1158.667	952	970	848	923.3333
ygbM	586	239	-116.667	-1.07757	0.176796	-1.23077	1.230772149	0.299563703	-0.29956	0.364874	1802	1237	1823	1620.667	1565	1593	1354	1504
ygbQ	87	52	65.66667	1.099949	0.774553	-1.03665	1.036651621	0.051931141	-0.05193	0.890284	655	659	657	657	717	709	742	722.6667
ygcA	1063	127	237	1.099302	0.885933	-1.02257	1.022570806	0.032200743	-0.0322	0.964513	2871	2481	1808	2386.667	2590	2673	2608	2623.667
ygcB	406	279	147	1.1171	0.885225	-1.02032	1.020324334	0.029027819	-0.02903	0.964454	1146	1513	1107	1255.333	1481	1301	1425	1402.333
ygcF	513	73	117	1.067721	0.661819	-1.06105	1.061048986	0.085491264	-0.08549	0.817313	1852	1895	1436	1727.667	1949	1779	1806	1844.667

Feature ID	Experiment - Range (original values)	Experiment - IQR (original values)	Experiment - Difference (original values)	Experiment - Fold Change (original values)	EDGE test: yccT NT vs WT NT , tagwise dispersions - P-value	EDGE test: yccT NT vs WT NT , tagwise dispersions - Fold change	yccT NT vs WT NT ABS|FC|	yccT NT vs WT NT Log2FC	yccT NT vs WT NT Log2FC +-	EDGE test: yccT NT vs WT NT , tagwise dispersions - FDR p-value correction	WT NT - WT.1.S22 - Expression values	WT NT - WT.2.S23 - Expression values	WT NT - WT.3.S24 - Expression values	WT NT - Means	yccT NT - yccT.1.S28 - Expression values	yccT NT - yccT.2.S29 - Expression values	yccT NT - yccT.3.S30 - Expression values	yccT NT - Means
ygcH	171	82	22.33333	1.034131	0.452248	-1.09734	1.097340708	0.13401153	-0.13401	0.661357	765	604	594	654.3333	725	619	686	676.6667
ygcY	17156	9617	8484.667	1.875039	0.088296	1.68018	1.680180147	0.748615926	0.748616	0.231502	17937	2832	8320	9696.333	19988	15779	18776	18181
ygdB	22	6	10.66667	1.181818	0.816637	1.039341	1.039340766	0.055668745	0.055669	0.919807	64	54	58	58.66667	76	70	62	69.33333
ygdD	62	26	0	-1	0.275313	-1.13993	1.13992781	0.188942463	-0.18894	0.488552	217	205	190	204	229	167	216	204
ygdE	210	42	123.3333	1.149013	0.975861	1.004406	1.004405857	0.006342347	0.006342	1	793	850	840	827.6667	1003	882	968	951
ygdH	7986	4012	-5747.33	-1.34278	0.011527	-1.52545	1.525445036	0.609230199	-0.60923	0.051541	24062	23128	20353	22514.33	17884	16341	16076	16767
ygdI	683	363	481	1.533851	0.015079	1.354481	1.354481323	0.4377405	0.437741	0.063211	1019	924	760	901	1443	1287	1416	1382
ygdK	130	54	83	1.331558	0.169259	1.176058	1.17605798	0.233959187	0.233959	0.354268	279	255	217	250.3333	344	347	309	333.3333
ygdL	175	40	52	1.116942	0.908103	-1.01513	1.015130151	0.021664709	-0.02166	0.976638	481	478	375	444.6667	499	441	550	496.6667
ygdP	732	214	266	1.114507	0.924474	-1.01429	1.014287233	0.020466262	-0.02047	0.984698	2686	2329	1954	2323	2683	2541	2543	2589
ygdQ	220	160	173	1.377729	0.109696	1.213472	1.213472035	0.279140861	0.279141	0.266774	498	455	421	458	641	615	637	631
ygdR	960	283	-131	-1.05938	0.255582	-1.19084	1.190843756	0.251984138	-0.25198	0.467472	2728	2515	1768	2337	2181	2360	2077	2206
ygeA	169	62	41	1.057423	0.564277	-1.07364	1.073638688	0.102508565	-0.10251	0.745911	759	744	639	714	808	760	697	755
ygeD	124	54	12.33333	1.026353	0.408385	-1.10445	1.104445329	0.143322006	-0.14332	0.622482	534	460	410	468	497	443	501	480.3333
ygfA	437	69	202.6667	1.255785	0.40475	1.111755	1.111755357	0.152839357	0.152839	0.619284	916	847	614	792.3333	1051	908	1026	995
ygfB	294	102	-114.333	-1.06667	0.149975	-1.21219	1.212191556	0.277617698	-0.27762	0.325833	1930	1866	1692	1829.333	1794	1715	1636	1715
ygfE	1494	451	74.66667	1.013867	0.48764	-1.11632	1.116322451	0.158753812	-0.15875	0.692606	6200	5247	4706	5384.333	5792	5518	5067	5459
ygfX	674	203	-57.3333	-1.02461	0.314238	-1.15999	1.159990351	0.214112805	-0.21411	0.529023	2731	2372	2057	2386.667	2508	2311	2169	2329.333
ygfY	259	76	-39	-1.0303	0.23659	-1.16707	1.16706512	0.222885063	-0.22289	0.444961	1469	1299	1210	1326	1283	1355	1223	1287
ygfZ	643	36	48.66667	1.017321	0.47215	-1.11406	1.114062951	0.155830756	-0.15583	0.678436	3119	2834	2476	2809.667	2970	2807	2798	2858.333
yggA	479	157	243.3333	1.285826	0.312791	1.141504	1.141504097	0.190936038	0.190936	0.527563	1015	858	681	851.3333	1122	1160	1002	1094.667
yggB	114	27	38	1.067019	0.588434	-1.07056	1.070564248	0.098371379	-0.09837	0.76537	544	600	557	567	658	584	573	605
yggE	1237	588	770.3333	1.462015	0.06858	1.296529	1.296529095	0.374654581	0.374655	0.196057	1874	1761	1367	1667.333	2360	2604	2349	2437.667
yggG	227	58	-2.33333	-1.00302	0.308448	-1.13539	1.135392978	0.183191724	-0.18319	0.523452	834	859	632	775	802	744	772	772.6667
yggH	212	57	91	1.120053	0.913756	-1.01371	1.013708503	0.019642857	-0.01964	0.980345	822	768	684	758	896	826	825	849
yggJ	126	9	62.33333	1.133858	0.972608	-1.0046	1.004598256	0.006618675	-0.00662	1	480	484	433	465.6667	559	475	550	528
yggL	241	111	-87.3333	-1.05219	0.177639	-1.20176	1.201764509	0.265154222	-0.26515	0.365421	1768	1731	1783	1760.667	1802	1657	1561	1673.333
yggM	952	171	-472	-1.29034	0.010112	-1.47276	1.472760632	0.558522968	-0.55852	0.046673	1793	2548	1952	2097.667	1622	1596	1659	1625.667
yggN	2057	529	-1393.33	-1.96114	8.8E-06	-2.27249	2.272485221	1.184270912	-1.18427	0.000167	1994	3119	3416	2843	1525	1359	1465	1449.667
yggR	71	20	36.66667	1.359477	0.142265	1.204095	1.204095187	0.267949446	0.267949	0.314245	120	106	80	102	126	139	151	138.6667
yggS	183	98	126	1.211055	0.648692	1.059598	1.059598304	0.08351744	0.083517	0.807403	597	575	619	597	758	695	716	723
yggT	185	22	95.33333	1.166279	0.900652	1.017068	1.017068293	0.024416554	0.024417	0.971919	544	577	599	573.3333	729	587	690	668.6667
yggU	103	27	37.66667	1.150667	1	-1.00046	1.000455843	0.000657492	-0.00066	1	211	251	288	250	314	271	278	287.6667
yggV	176	54	61.66667	1.099356	0.747962	-1.04246	1.042460976	0.059993378	-0.05999	0.876079	577	657	628	620.6667	753	603	691	682.3333
yggW	188	118	-13.3333	-1.01597	0.250237	-1.16567	1.165673569	0.221163839	-0.22116	0.461231	739	927	879	848.3333	880	761	864	835
yggX	196	89	-133.667	-1.32443	0.000901	-1.51358	1.513581472	0.597966334	-0.59797	0.007094	515	579	543	545.6667	427	383	426	412

Feature ID	Experiment - Range (original values)	Experiment - IQR (original values)	Experiment - Difference (original values)	Experiment - Fold Change (original values)	EDGE test: yccT NT vs WT NT , tagwise dispersions - P-value	EDGE test: yccT NT vs WT NT , tagwise dispersions - Fold change	yccT NT vs WT NT ABS|FC|	yccT NT vs WT NT Log2FC	yccT NT vs WT NT Log2FC +-	EDGE test: yccT NT vs WT NT , tagwise dispersions - FDR p-value correction	WT NT - WT.1.S22 - Expression values	WT NT - WT.2.S23 - Expression values	WT NT - WT.3.S24 - Expression values	WT NT - Means	yccT NT - yccT.1.S28 - Expression values	yccT NT - yccT.2.S29 - Expression values	yccT NT - yccT.3.S30 - Expression values	yccT NT - Means
yghA	511	280	335	1.690722	0.001532	1.496783	1.496782551	0.581864645	0.581865	0.010658	600	467	388	485	899	747	814	820
yghB	851	216	352.6667	1.167246	0.8375	1.030299	1.030299037	0.04306313	0.043063	0.936875	2379	2163	1784	2108.667	2635	2291	2458	2461.333
yghJ	578	126	3.666667	1.001999	0.377874	-1.12827	1.128273024	0.17411622	-0.17412	0.594928	2129	1824	1551	1834.667	1949	1846	1720	1838.333
yghU	418	138	-123	-1.09858	0.093889	-1.24288	1.242875903	0.313682255	-0.31368	0.240615	1595	1340	1177	1370.667	1324	1233	1186	1247.667
yghW	74	25	39.33333	1.320652	0.289339	1.154052	1.154052102	0.206708359	0.206708	0.505626	103	145	120	122.6667	177	142	167	162
ygiB	2367	1542	1040.667	1.24309	0.550769	1.106313	1.106312501	0.145758962	0.145759	0.737684	5766	3678	3399	4281	5558	5187	5220	5321.667
ygiC	2102	1605	1108.333	1.201296	0.706642	1.062864	1.062864334	0.08795746	0.087957	0.84648	6747	4982	4789	5506	6891	6365	6587	6614.333
ygiD	136	14	51	1.192211	0.669949	1.054714	1.054714237	0.07685217	0.076852	0.822256	303	291	202	265.3333	338	289	322	316.3333
ygiE	953	155	185.6667	1.085012	0.801679	-1.03845	1.038445811	0.054425935	-0.05443	0.908966	2659	2187	1706	2184	2531	2342	2236	2369.667
ygiF	451	302	242	1.206016	0.617195	1.065735	1.065735267	0.091849112	0.091849	0.78418	1402	1100	1022	1174.667	1473	1330	1447	1416.667
ygiH	464	310	173.6667	1.167632	0.798062	1.035411	1.035410505	0.050202861	0.050203	0.906256	1342	878	888	1036	1268	1198	1163	1209.667
ygiK	130	114	118.6667	1.467806	0.031092	1.289482	1.289482105	0.366791752	0.366792	0.110305	263	248	250	253.6667	378	375	364	372.3333
ygiM	2127	191	-1316.67	-1.74234	0.000422	-2.0173	2.017296942	1.012423461	-1.01242	0.00379	2006	3459	3806	3090.333	1679	1827	1815	1773.667
ygiN	284	228	-122.667	-1.11131	0.063058	-1.26564	1.265644943	0.339872735	-0.33987	0.184672	1299	1254	1121	1224.667	1265	1026	1015	1102
ygiR	171	84	112.6667	1.433333	0.04643	1.262936	1.262935956	0.336781482	0.336781	0.148684	285	261	234	260	368	345	405	372.6667
ygiW	135	99	-86	-1.06854	0.128227	-1.21869	1.218693953	0.285335872	-0.28534	0.295309	1356	1349	1317	1340.667	1321	1222	1221	1254.667
ygiX	147	71	22.33333	1.08396	0.804805	-1.03669	1.036693649	0.051989629	-0.05199	0.911112	364	217	217	266	288	299	278	288.3333
ygiY	145	16	-0.33333	-1.00069	0.298859	-1.13315	1.133146994	0.180335022	-0.18034	0.514669	557	478	412	482.3333	517	462	467	482
ygjO	223	32	-16.6667	-1.0206	0.245087	-1.16314	1.163135683	0.218019402	-0.21802	0.455719	776	962	739	825.6667	871	762	794	809
ygjP	95	24	4	1.010213	0.339938	-1.1211	1.121097179	0.16491134	-0.16491	0.553593	440	390	345	391.6667	407	402	378	395.6667
ygjQ	34	17	-1	-1.00838	0.270498	-1.14441	1.144412589	0.194607273	-0.19461	0.483691	132	128	101	120.3333	135	111	112	119.3333
ygjR	1601	559	-1063.33	-1.23001	0.033552	-1.39574	1.395744415	0.481034783	-0.48103	0.11651	6139	5782	5138	5686.333	4752	4579	4538	4623
ygjT	48	28	20.33333	1.133479	1	1.001617	1.001616899	0.002330808	0.002331	1	181	143	133	152.3333	171	171	176	172.6667
ygjU	903	495	410.6667	1.282051	0.355278	1.142004	1.14200373	0.191567363	0.191567	0.570376	1912	1370	1086	1456	1865	1746	1989	1866.667
yhaH	325	259	230.6667	1.484933	0.030367	1.314064	1.31406383	0.394035356	0.394035	0.108477	585	432	410	475.6667	735	691	693	706.3333
yhaJ	298	124	182.6667	1.434231	0.048253	1.264681	1.264680728	0.338773218	0.338773	0.152295	472	432	358	420.6667	656	556	598	603.3333
yhaK	71	23	36.66667	1.30303	0.244112	1.154505	1.154504595	0.207273915	0.207274	0.45467	142	130	91	121	158	162	153	157.6667
yhaL	409	70	-58.6667	-1.07875	0.161002	-1.20972	1.209722484	0.274676124	-0.27468	0.342012	997	826	588	803.6667	720	790	725	745
yhaN	2243	209	-1013	-1.47686	0.003918	-1.68614	1.686139589	0.753723977	-0.75372	0.022227	2402	4192	2818	3137.333	2193	2231	1949	2124.333
yhaO	3571	662	-1713.33	-1.5956	0.001858	-1.81841	1.818414026	0.862680717	-0.86268	0.012391	3416	6247	4107	4590	2754	3200	2676	2876.667
yhbC	384	241	180.6667	1.090439	0.744246	-1.04771	1.047710468	0.067240087	-0.06724	0.873541	1876	2179	1938	1997.667	2260	2032	2243	2178.333
yhbG	632	163	-321.667	-1.16378	0.062372	-1.33765	1.337653608	0.419704572	-0.4197	0.183268	1997	2403	2457	2285.667	2115	1825	1952	1964
yhbH	5390	4324	3390.667	1.472567	0.135271	1.3156	1.315599553	0.395720423	0.39572	0.304252	10165	5841	5519	7175	10843	10909	9945	10565.67
yhbJ	594	313	-150.667	-1.04937	0.240738	-1.19327	1.193268342	0.254918513	-0.25492	0.451455	3422	3266	2920	3202.667	3233	2828	3095	3052
yhbL	517	220	149	1.054625	0.622797	-1.07784	1.077841677	0.108145278	-0.10815	0.787112	3029	2512	2642	2727.667	2973	2862	2795	2876.667
yhbN	405	80	-237	-1.12582	0.082832	-1.28979	1.289792054	0.367138487	-0.36714	0.22214	1895	2256	2211	2120.667	1940	1851	1860	1883.667

Feature ID	Experiment - Range (original values)	Experiment - IQR (original values)	Experiment - Difference (original values)	Experiment - Fold Change (original values)	EDGE test: yccT NT vs WT NT , tagwise dispersions - P-value	EDGE test: yccT NT vs WT NT , tagwise dispersions - Fold change	yccT NT vs WT NT ABS|FC|	yccT NT vs WT NT Log2FC	yccT NT vs WT NT Log2FC +-	EDGE test: yccT NT vs WT NT , tagwise dispersions - FDR p-value correction	WT NT - WT.1.S22 - Expression values	WT NT - WT.2.S23 - Expression values	WT NT - WT.3.S24 - Expression values	WT NT - Means	yccT NT - yccT.1.S28 - Expression values	yccT NT - yccT.2.S29 - Expression values	yccT NT - yccT.3.S30 - Expression values	yccT NT - Means
yhbO	158	18	-62	-1.18693	0.017331	-1.35223	1.352230311	0.435340891	-0.43534	0.070005	367	465	349	393.6667	353	307	335	331.6667
yhbP	488	125	-244	-1.32162	0.002717	-1.50435	1.504349062	0.589139362	-0.58914	0.016675	897	1231	880	1002.667	778	755	743	758.6667
yhbQ	601	126	-337	-1.28407	0.004262	-1.4578	1.457803852	0.543796617	-0.5438	0.023616	1562	1723	1285	1523.333	1278	1122	1159	1186.333
yhbS	2561	885	-1631	-1.27643	0.019398	-1.45098	1.450976723	0.537024376	-0.53702	0.076469	7602	8278	6714	7531.333	6155	5717	5829	5900.333
yhbT	3163	1718	-2205.67	-1.35302	0.008035	-1.54171	1.54171043	0.624531818	-0.62453	0.039127	8152	9091	8118	8453.667	6400	6416	5928	6248
yhbU	887	183	-437.667	-1.60759	0.004274	-1.8741	1.87410498	0.90620177	-0.9062	0.023642	572	1443	1459	1158	694	642	825	720.3333
yhbV	829	123	-401	-1.58032	0.003791	-1.84436	1.844362245	0.883122038	-0.88312	0.021646	564	1319	1393	1092	716	617	740	691
yhbW	123	15	-59.6667	-1.39254	0.002388	-1.60628	1.606279847	0.683723262	-0.68372	0.0149	162	239	234	211.6667	163	116	177	152
yhbY	244	165	109.6667	1.052531	0.587498	-1.07991	1.079907044	0.110907133	-0.11091	0.76537	2224	2051	1988	2087.667	2232	2216	2144	2197.333
yhbZ	334	135	239.3333	1.322407	0.241091	1.158124	1.158124204	0.211789985	0.21179	0.451455	765	717	745	742.3333	1014	880	1051	981.6667
yhcB	1461	943	-1034.33	-1.28951	0.014979	-1.47147	1.471466876	0.557255067	-0.55726	0.062913	4565	4746	4510	4607	3866	3567	3285	3572.667
yhcC	56	19	9.333333	1.046205	0.493697	-1.09453	1.094530596	0.130312283	-0.13031	0.697625	176	215	215	202	206	196	232	211.3333
yhcG	111	30	54	1.196602	0.694314	1.052239	1.052239466	0.073463066	0.073463	0.837373	309	232	283	274.6667	330	343	313	328.6667
yhcH	2853	440	-1253.67	-1.73875	0.000481	-1.9748	1.974797552	0.981704762	-0.9817	0.004241	2292	4402	2158	2950.667	1824	1718	1549	1697
yhcK	387	213	-18	-1.00881	0.32265	-1.14561	1.145605949	0.19611089	-0.19611	0.537872	2214	2145	1827	2062	2171	1932	2029	2044
yhcM	202	132	-115	-1.09027	0.092928	-1.24657	1.246574186	0.317968743	-0.31797	0.239258	1375	1373	1419	1389	1364	1217	1241	1274
yhcN	48	29	35.66667	1.514423	0.024478	1.336735	1.336734572	0.418713026	0.418713	0.091299	79	68	61	69.33333	97	109	109	105
yhcO	18	8	4	1.113208	0.91306	-1.02082	1.020824007	0.029734163	-0.02973	0.98008	44	26	36	35.33333	42	34	42	39.33333
yhcP	154	38	73.66667	1.291173	0.276477	1.139996	1.139995557	0.189028201	0.189028	0.48944	296	258	205	253	359	287	334	326.6667
yhcQ	65	11	30.33333	1.294498	0.290472	1.148713	1.148713371	0.200018859	0.200019	0.507124	120	117	72	103	128	137	135	133.3333
yhcR	22	3	6.666667	1.12987	1	-1.0058	1.005802753	0.008347408	-0.00835	1	54	55	45	51.33333	67	55	52	58
yhcS	170	29	30	1.050392	0.515451	-1.08311	1.083108019	0.115177131	-0.11518	0.715369	640	606	540	595.3333	710	589	577	625.3333
yhdA	348	59	-182	-1.13791	0.063636	-1.30616	1.306157524	0.385328898	-0.38533	0.185997	1274	1622	1609	1501.667	1336	1282	1341	1319.667
yhdE	165	45	-9	-1.01017	0.259466	-1.15435	1.154348101	0.207078343	-0.20708	0.470847	860	940	881	893.6667	905	792	957	884.6667
yhdG	417	166	265.6667	1.259694	0.45578	1.101132	1.101131764	0.138987115	0.138987	0.664329	966	1089	1014	1023	1383	1180	1303	1288.667
yhdH	2906	705	-2099.67	-1.60157	0.000847	-1.84615	1.846149016	0.884519008	-0.88452	0.006727	4188	6302	6280	5590	3592	3483	3396	3490.333
yhdJ	58	23	31.66667	1.157546	0.892259	1.017284	1.017284283	0.0247229	0.024723	0.968565	218	195	190	201	248	212	238	232.6667
yhdN	95	69	51	1.282288	0.314401	1.138533	1.138532799	0.187175853	0.187176	0.529096	231	161	150	180.6667	220	245	230	231.6667
yhdP	363	134	-223	-1.16997	0.028304	-1.33758	1.337582064	0.419627407	-0.41963	0.102801	1470	1595	1540	1535	1368	1232	1336	1312
yhdT	30	4	15.33333	1.359375	0.232691	1.19897	1.198970281	0.2617959	0.261796	0.43987	48	47	33	42.66667	63	60	51	58
yhdU	49	17	14.33333	1.069021	0.591871	-1.06318	1.063176519	0.088381147	-0.08838	0.767985	223	212	188	207.6667	237	206	223	222
yhdV	179	67	103.6667	1.421409	0.073425	1.261131	1.261131442	0.334718649	0.334719	0.204975	267	290	181	246	334	360	355	349.6667
yheL	108	21	-54	-1.17551	0.025384	-1.3508	1.350796464	0.433810308	-0.43381	0.093731	309	374	402	361.6667	325	294	304	307.6667
yheM	50	29	-21.3333	-1.06504	0.116552	-1.21955	1.219546574	0.286344855	-0.28634	0.277508	316	366	366	349.3333	347	319	318	328
yheN	240	59	-92	-1.26641	0.020938	-1.46882	1.468821666	0.554659245	-0.55466	0.080675	312	452	548	437.3333	357	308	371	345.3333
yheO	1222	46	-494.667	-1.27558	0.031046	-1.47765	1.477652289	0.563306824	-0.56331	0.110228	1594	2459	2816	2289.667	1825	1781	1779	1795

Feature ID	Experiment - Range (original values)	Experiment - IQR (original values)	Experiment - Difference (original values)	Experiment - Fold Change (original values)	EDGE test: yccT NT vs WT NT , tagwise dispersions - P-value	EDGE test: yccT NT vs WT NT , tagwise dispersions - Fold change	yccT NT vs WT NT ABS|FC|	yccT NT vs WT NT Log2FC	yccT NT vs WT NT Log2FC +-	EDGE test: yccT NT vs WT NT , tagwise dispersions - FDR p-value correction	WT NT - WT.1.S22 - Expression values	WT NT - WT.2.S23 - Expression values	WT NT - WT.3.S24 - Expression values	WT NT - Means	yccT NT - yccT.1.S28 - Expression values	yccT NT - yccT.2.S29 - Expression values	yccT NT - yccT.3.S30 - Expression values	yccT NT - Means
yheR	34	14	-14	-1.26582	0.016619	-1.43484	1.434842078	0.520891959	-0.52089	0.067933	70	80	50	66.66667	63	49	46	52.66667
yheS	373	14	-206	-1.12115	0.081952	-1.28242	1.282420718	0.358869638	-0.35887	0.220943	1724	2052	1943	1906.333	1710	1679	1712	1700.333
yheT	301	37	101.6667	1.070099	0.638597	-1.06168	1.061684869	0.086355606	-0.08636	0.800233	1512	1535	1304	1450.333	1605	1498	1553	1552
yheU	64	33	-5	-1.01531	0.217465	-1.15671	1.156713691	0.210031814	-0.21003	0.420781	338	352	305	331.6667	366	302	312	326.6667
yheV	146	29	-87	-1.37286	0.000623	-1.57443	1.574431833	0.654831296	-0.65483	0.005252	270	359	332	320.3333	246	213	241	233.3333
yhfA	578	330	-366	-1.13389	0.092495	-1.29428	1.294284636	0.372154926	-0.37215	0.23856	3010	3246	3043	3099.667	2853	2668	2680	2733.667
yhfC	53	18	13.33333	1.133333	1	1.001351	1.001350579	0.00194716	0.001947	1	101	126	73	100	124	117	99	113.3333
yhfG	52	39	31.33333	1.415929	0.103367	1.255089	1.255089226	0.327789931	0.32779	0.257222	103	64	59	75.33333	106	103	111	106.6667
yhfK	654	57	-96.3333	-1.03299	0.29296	-1.17696	1.17695755	0.235062287	-0.23506	0.50946	2879	3412	2758	3016.333	3073	2822	2865	2920
yhfL	13	3	1.333333	1.051948	0.701618	-1.08808	1.088075036	0.121778052	-0.12178	0.843099	21	22	34	25.66667	31	25	25	27
yhgE	83	11	36.66667	1.091211	0.720462	-1.04465	1.044649008	0.063018293	-0.06302	0.854717	411	404	391	402	474	427	415	438.6667
yhgF	247	142	192.6667	1.233065	0.531228	1.081556	1.08155594	0.113108286	0.113108	0.723599	860	808	812	826.6667	1055	954	1049	1019.333
yhgG	144	39	-7.33333	-1.0242	0.2918	-1.15785	1.157847804	0.211445627	-0.21145	0.50842	299	388	244	310.3333	269	308	332	303
yhgH	58	19	-32.3333	-1.21133	0.006324	-1.3772	1.377204027	0.461742304	-0.46174	0.032282	188	202	166	185.3333	167	148	144	153
yhgI	680	251	-486.333	-1.57714	3.35E-05	-1.8081	1.808099784	0.854474298	-0.85447	0.000503	1132	1445	1410	1329	881	882	765	842.6667
yhhA	344	267	184	1.418816	0.114126	1.262618	1.262617703	0.336417884	0.336418	0.273818	619	347	352	439.3333	691	628	551	623.3333
yhhF	133	75	86.66667	1.30445	0.228511	1.149593	1.149593118	0.20112333	0.201123	0.434562	315	282	257	284.6667	390	367	357	371.3333
yhhJ	276	150	173.3333	1.395738	0.081155	1.230787	1.230787288	0.299581448	0.299581	0.219669	503	426	385	438	661	576	597	611.3333
yhhK	42	19	-23	-1.12042	0.037403	-1.27572	1.275719963	0.351311674	-0.35131	0.126588	226	206	210	214	202	184	187	191
yhhL	57	18	21.66667	1.098336	0.786071	-1.03364	1.033635691	0.047727791	-0.04773	0.898692	223	245	193	220.3333	241	235	250	242
yhhM	77	29	14.66667	1.056338	0.559509	-1.07732	1.077321756	0.107449194	-0.10745	0.744053	238	310	233	260.3333	267	262	296	275
yhhN	475	285	192.3333	1.19317	0.666023	1.058778	1.058777622	0.082399608	0.0824	0.819919	1285	892	810	995.6667	1257	1130	1177	1188
yhhP	281	119	173	1.367044	0.120474	1.207085	1.207084713	0.271526927	0.271527	0.283195	532	486	396	471.3333	677	605	651	644.3333
yhhQ	153	39	72.66667	1.334356	0.189721	1.176942	1.176942207	0.23504348	0.235043	0.380903	228	255	169	217.3333	322	267	281	290
yhhS	221	55	93	1.102611	0.80348	-1.03136	1.031355419	0.044541591	-0.04454	0.910076	967	912	840	906.3333	1061	959	978	999.3333
yhhT	44	25	2.666667	1.020833	0.4554	-1.10494	1.104939336	0.143967164	-0.14397	0.664213	145	138	101	128	113	137	142	130.6667
yhhV	29	13	12.33333	1.393617	0.254756	1.23032	1.230319687	0.299033235	0.299033	0.466842	44	22	28	31.33333	41	39	51	43.66667
yhhW	87	27	-7.33333	-1.02256	0.210055	-1.15831	1.158314259	0.21202672	-0.21203	0.410933	366	352	279	332.3333	337	310	328	325
yhhX	337	158	-203.333	-1.19922	0.012297	-1.36415	1.364149747	0.448002022	-0.448	0.054002	1333	1159	1180	1224	1065	996	1001	1020.667
yhhY	170	65	57.33333	1.130898	0.98697	1.00239	1.002389576	0.003443317	0.003443	1	528	428	358	438	502	493	491	495.3333
yhiH	1286	870	851.3333	1.487591	0.056831	1.321656	1.321655895	0.402346607	0.402347	0.170955	2184	1679	1375	1746	2661	2549	2582	2597.333
yhiI	499	328	253.3333	1.240051	0.469187	1.098888	1.09888829	0.136044733	0.136045	0.676399	1318	972	876	1055.333	1375	1251	1300	1308.667
yhiN	97	54	45.33333	1.118056	0.873282	-1.02042	1.020421517	0.029165226	-0.02917	0.958007	362	423	367	384	459	421	408	429.3333
yhiP	14644	7225	-10276.7	-1.35666	0.011253	-1.54319	1.543185654	0.625911637	-0.62591	0.050466	39447	42386	35437	39090	30486	28212	27742	28813.33
yhiQ	1076	269	-574.667	-1.21677	0.032319	-1.38343	1.383430719	0.468250396	-0.46825	0.113026	3407	3460	2810	3225.667	2919	2384	2650	2651
yhiR	140	66	-6.66667	-1.00612	0.270496	-1.14814	1.148140369	0.199299034	-0.1993	0.483691	1083	1145	1058	1095.333	1137	1005	1124	1088.667

Feature ID	Experiment - Range (original values)	Experiment - IQR (original values)	Experiment - Difference (original values)	Experiment - Fold Change (original values)	EDGE test: yccT NT vs WT NT , tagwise dispersions - P-value	EDGE test: yccT NT vs WT NT , tagwise dispersions - Fold change	yccT NT vs WT NT ABS|FC|	yccT NT vs WT NT Log2FC	yccT NT vs WT NT Log2FC +-	EDGE test: yccT NT vs WT NT , tagwise dispersions - FDR p-value correction	WT NT - WT.1.S22 - Expression values	WT NT - WT.2.S23 - Expression values	WT NT - WT.3.S24 - Expression values	WT NT - Means	yccT NT - yccT.1.S28 - Expression values	yccT NT - yccT.2.S29 - Expression values	yccT NT - yccT.3.S30 - Expression values	yccT NT - Means
yhjB	75	64	59.66667	2.584071	2.85E-07	2.292494	2.292493516	1.196917653	1.196918	9.14E-06	58	30	25	37.66667	100	94	98	97.33333
yhjC	31	12	4.333333	1.030806	0.406164	-1.10909	1.109086687	0.149372132	-0.14937	0.620853	133	144	145	140.6667	154	125	156	145
yhjD	68	50	-3.66667	-1.01407	0.264846	-1.14627	1.146271339	0.196948591	-0.19695	0.476522	285	288	220	264.3333	261	286	235	260.6667
yhjE	61	8	20.66667	1.132479	1	-1.00219	1.00219021	0.003156349	-0.00316	1	165	170	133	156	194	174	162	176.6667
yhjG	709	348	468	1.407311	0.105032	1.239786	1.239785908	0.310091011	0.310091	0.259273	1162	1315	970	1149	1662	1510	1679	1617
yhjH	3494	1674	2184.667	1.666938	0.02525	1.490381	1.490381444	0.575681617	0.575682	0.093391	4125	3642	2060	3275.667	5554	5316	5511	5460.333
yhjJ	1299	38	-670	-1.27258	0.0262	-1.46801	1.46801413	0.553865855	-0.55387	0.096176	2542	3214	3628	3128	2541	2329	2504	2458
yhjK	211	53	72.33333	1.086145	0.693188	-1.04953	1.049532618	0.069747004	-0.06975	0.837317	882	843	794	839.6667	1005	829	902	912
yhjQ	264	155	201	1.961722	1.55E-05	1.716537	1.716536528	0.779500558	0.779501	0.000265	223	183	221	209	447	376	407	410
yhjR	78	45	60.66667	1.896552	7.63E-05	1.662941	1.66294118	0.73373714	0.733737	0.000952	66	68	69	67.66667	128	113	144	128.3333
yhjV	287	161	209.3333	1.878322	6.24E-05	1.653105	1.653104656	0.725178062	0.725178	0.000811	283	208	224	238.3333	463	385	495	447.6667
yhjW	584	85	-353	-1.16452	0.066725	-1.33635	1.336350345	0.418298282	-0.4183	0.192862	2191	2619	2686	2498.667	2210	2102	2125	2145.667
yhjY	74	60	42	1.205546	0.605932	1.066598	1.066597551	0.093015921	0.093016	0.777315	245	185	183	204.3333	234	257	248	246.3333
yiaB	59	40	43.33333	1.81761	0.000552	1.601641	1.601641089	0.679550891	0.679551	0.004738	66	44	49	53	103	97	89	96.33333
yiaC	205	90	75	1.044213	0.506681	-1.096	1.096001253	0.132249448	-0.13225	0.70721	1654	1651	1784	1696.333	1856	1714	1744	1771.333
yiaD	531	251	-332.333	-1.83431	1.14E-07	-2.08835	2.088350437	1.062363824	-1.06236	4.09E-06	651	901	640	730.6667	389	370	436	398.3333
yiaE	1064	299	-405	-1.11106	0.129644	-1.2624	1.262395722	0.336164221	-0.33616	0.296753	4511	3908	3736	4051.667	3896	3447	3597	3646.667
yiaF	453	248	-251	-1.12029	0.090776	-1.28177	1.281765258	0.358132071	-0.35813	0.235911	2324	2310	2379	2337.667	2272	1926	2062	2086.667
yiaG	109	83	70	1.921053	0.004744	1.723011	1.723010945	0.784931866	0.784932	0.025759	129	50	49	76	158	147	133	146
yiaH	72	49	46	1.303297	0.227575	1.152327	1.152327211	0.204550438	0.20455	0.433341	183	144	128	151.6667	200	193	200	197.6667
yiaJ	90	41	49.33333	1.15678	0.948146	1.009898	1.00989756	0.014208959	0.014209	0.999617	296	306	342	314.6667	386	359	347	364
yiaK	18	7	7	1.168	0.883408	1.030322	1.030322452	0.043095918	0.043096	0.962948	47	40	38	41.66667	47	56	43	48.66667
yiaL	14	1	5	1.348837	0.487445	1.18781	1.187810375	0.248304539	0.248305	0.692606	15	14	14	14.33333	12	20	26	19.33333
yiaM	11	1	6.333333	3.375	0.012996	2.887346	2.887346079	1.52974404	1.529744	0.056491	4	3	1	2.666667	12	4	11	9
yiaN	14	9	10	1.666667	0.067785	1.465074	1.465074027	0.550973563	0.550974	0.194909	15	18	12	15	24	26	25	25
yiaO	13	9	3	1.236842	0.78393	1.087496	1.087496053	0.121010165	0.12101	0.896874	10	19	9	12.66667	21	18	8	15.66667
yibD	45	9	17	1.168317	0.846805	1.028704	1.028704219	0.040828227	0.040828	0.942334	109	100	94	101	109	106	139	118
yibF	332	98	-193.333	-1.17802	0.02069	-1.34054	1.340537783	0.422811882	-0.42281	0.079997	1264	1380	1194	1279.333	1114	1096	1048	1086
yibK	77	41	18	1.137056	0.987574	-1.00399	1.003989302	0.005743896	-0.00574	1	102	179	113	131.3333	154	132	162	149.3333
yibL	115	26	-39.6667	-1.10128	0.079583	-1.26114	1.261141709	0.334730395	-0.33473	0.217107	406	426	462	431.3333	428	400	347	391.6667
yibN	573	122	-253.667	-1.15934	0.054981	-1.33514	1.335142458	0.416993684	-0.41699	0.166637	1578	1852	2107	1845.667	1682	1534	1560	1592
yibP	230	84	-74	-1.05074	0.162525	-1.20328	1.203283429	0.266976505	-0.26698	0.344734	1472	1542	1583	1532.333	1564	1353	1458	1458.333
yibR	64	13	-16	-1.11852	0.072521	-1.27674	1.276735319	0.352459469	-0.35246	0.203508	128	186	139	151	141	122	142	135
yicC	566	424	-457.667	-1.30302	0.003493	-1.4897	1.489695132	0.575017112	-0.57502	0.02042	1909	2009	1986	1968	1603	1443	1485	1510.333
yicE	93	20	-46.6667	-1.11336	0.046817	-1.26791	1.267906335	0.342448172	-0.34245	0.149672	474	471	430	458.3333	437	381	417	411.6667
yicF	47	28	22.66667	1.108626	0.862288	-1.02269	1.022688599	0.032366923	-0.03237	0.951631	234	205	187	208.6667	234	227	233	231.3333

Feature ID	Experiment - Range (original values)	Experiment - IQR (original values)	Experiment - Difference (original values)	Experiment - Fold Change (original values)	EDGE test: yccT NT vs WT NT , tagwise dispersions - P-value	EDGE test: yccT NT vs WT NT , tagwise dispersions - Fold change	yccT NT vs WT NT ABS|FC|	yccT NT vs WT NT Log2FC	yccT NT vs WT NT Log2FC +-	EDGE test: yccT NT vs WT NT , tagwise dispersions - FDR p-value correction	WT NT - WT.1.S22 - Expression values	WT NT - WT.2.S23 - Expression values	WT NT - WT.3.S24 - Expression values	WT NT - Means	yccT NT - yccT.1.S28 - Expression values	yccT NT - yccT.2.S29 - Expression values	yccT NT - yccT.3.S30 - Expression values	yccT NT - Means
yicH	1218	853	476	1.246675	0.515876	1.112215	1.112214923	0.1534356	0.153436	0.715445	2719	1569	1501	1929.667	2521	2422	2274	2405.667
yicI	179	123	93.66667	1.271498	0.339952	1.130092	1.130091935	0.176440144	0.17644	0.553593	447	317	271	345	450	440	426	438.6667
yicJ	31	17	19	1.337278	0.215515	1.180144	1.18014357	0.238962381	0.238962	0.417919	66	57	46	56.33333	75	74	77	75.33333
yicL	266	97	123.3333	1.276946	0.323418	1.129754	1.129754449	0.176009238	0.176009	0.538312	535	438	363	445.3333	629	548	529	568.6667
yicM	113	49	-73.6667	-1.37649	0.000213	-1.56562	1.565622146	0.646736069	-0.64674	0.002131	261	297	250	269.3333	201	202	184	195.6667
yicN	60	36	-42.6667	-1.15842	0.022954	-1.32152	1.321515734	0.402193602	-0.40219	0.086709	306	306	324	312	264	274	270	269.3333
yidA	299	209	-104.333	-1.05581	0.178093	-1.20098	1.200975673	0.264206928	-0.26421	0.366016	2038	2091	1792	1973.667	2008	1799	1801	1869.333
yidC	622	358	42.33333	1.016735	0.445536	-1.13104	1.131039994	0.177649945	-0.17765	0.655966	2156	2746	2687	2529.667	2778	2329	2609	2572
yidE	1578	889	715.3333	1.273167	0.440092	1.135594	1.135594413	0.183447657	0.183448	0.651536	3480	2474	1902	2618.667	3422	3217	3363	3334
yidF	509	135	163.6667	1.11076	0.908814	-1.01612	1.016120282	0.023071189	-0.02307	0.976926	1577	1643	1213	1477.667	1508	1722	1694	1641.333
yidG	74	11	32.66667	1.143695	1	1.002326	1.002325696	0.003351374	0.003351	1	236	218	228	227.3333	292	249	239	260
yidH	96	52	50.66667	1.218391	0.540674	1.073825	1.073825356	0.102759376	0.102759	0.729959	271	219	206	232	302	253	293	282.6667
yidQ	245	32	-126	-1.14958	0.035258	-1.30558	1.305575481	0.384685869	-0.38469	0.120734	976	1047	882	968.3333	850	875	802	842.3333
yidR	133	62	-72.3333	-1.11555	0.053549	-1.27428	1.274283547	0.349686334	-0.34969	0.163977	673	728	694	698.3333	672	611	595	626
yidY	97	29	29.66667	1.137558	0.955291	1.007509	1.007508692	0.010792285	0.010792	1	225	254	168	215.6667	221	250	265	245.3333
yidZ	118	39	43	1.098398	0.808804	-1.03006	1.030061122	0.042729947	-0.04273	0.913305	492	445	374	437	487	484	469	480
yieE	190	34	61.33333	1.1133	0.895457	-1.0168	1.01679525	0.024029196	-0.02403	0.969641	619	549	456	541.3333	646	583	579	602.6667
yieF	462	299	-358.667	-1.38008	0.000342	-1.57223	1.572228648	0.652811043	-0.65281	0.003149	1361	1251	1295	1302.333	980	952	899	943.6667
yieG	206	135	148.6667	1.249441	0.433192	1.099514	1.099513652	0.136865518	0.136866	0.644357	643	588	557	596	763	723	748	744.6667
yieH	86	35	58.33333	1.233957	0.503557	1.082094	1.082093803	0.113825567	0.113826	0.704429	258	246	244	249.3333	330	281	312	307.6667
yieM	299	9	-95	-1.07081	0.133697	-1.22112	1.221120045	0.288205035	-0.28821	0.301789	1362	1591	1357	1436.667	1380	1292	1353	1341.667
yieN	386	157	-59.6667	-1.02081	0.320432	-1.16031	1.16030852	0.214508461	-0.21451	0.535181	3100	2967	2714	2927	2975	2810	2817	2867.333
yieO	285	71	-83	-1.07897	0.109145	-1.22789	1.227894487	0.296186595	-0.29619	0.266045	1091	1296	1015	1134	1086	1011	1056	1051
yieP	312	56	-131	-1.12351	0.058114	-1.26994	1.269939288	0.344759528	-0.34476	0.173636	1302	1283	990	1191.667	1062	1088	1032	1060.667
yifA	451	227	10.33333	1.004005	0.41368	-1.12957	1.129565425	0.175767835	-0.17577	0.626673	2797	2598	2346	2580.333	2662	2675	2435	2590.667
yifB	170	102	100	1.323974	0.23244	1.170304	1.17030433	0.226883743	0.226884	0.439582	384	250	292	308.6667	394	412	420	408.6667
yifE	157	17	-34	-1.04533	0.174048	-1.19053	1.190534088	0.251608929	-0.25161	0.361728	751	849	752	784	768	790	692	750
yifK	95	8	40.33333	1.079974	0.674324	-1.05162	1.05161559	0.072607435	-0.07261	0.825117	525	532	456	504.3333	551	533	550	544.6667
yifL	123	87	-90.3333	-1.09018	0.087608	-1.24455	1.244550036	0.315624233	-0.31562	0.230108	1086	1078	1112	1092	1025	991	989	1001.667
yifZ	73	35	37	1.380137	0.125559	1.221489	1.221488824	0.288640664	0.288641	0.29098	123	89	80	97.33333	126	124	153	134.3333
yigA	786	166	-447.333	-1.25359	0.01462	-1.43514	1.435141156	0.521192643	-0.52119	0.062112	1931	2489	2214	2211.333	1824	1703	1765	1764
yigB	359	68	-188	-1.38657	0.001613	-1.59257	1.59256976	0.671356569	-0.67136	0.01113	535	798	690	674.3333	544	476	439	486.3333
yigC	803	74	-294.333	-1.10271	0.141836	-1.25953	1.259532071	0.332887857	-0.33289	0.31408	2911	3532	3037	3160	2971	2729	2897	2865.667
yigCb	36	10	23.66667	1.238255	0.502323	1.090867	1.090866958	0.125475161	0.125475	0.704228	100	104	94	99.33333	110	130	129	123
yigI	8	2	-1.33333	-1.09524	0.380468	-1.24174	1.241738916	0.312361869	-0.31236	0.596902	13	19	14	15.33333	11	15	16	14
yigL	488	94	-297.333	-1.37621	0.001685	-1.57897	1.578967982	0.658981917	-0.65898	0.011541	875	1263	1125	1087.667	815	781	775	790.3333

Feature ID	Experiment - Range (original values)	Experiment - IQR (original values)	Experiment - Difference (original values)	Experiment - Fold Change (original values)	EDGE test: yccT NT vs WT NT , tagwise dispersions - P-value	EDGE test: yccT NT vs WT NT , tagwise dispersions - Fold change	yccT NT vs WT NT ABS|FC|	yccT NT vs WT NT Log2FC	yccT NT vs WT NT Log2FC +-	EDGE test: yccT NT vs WT NT , tagwise dispersions - FDR p-value correction	WT NT - WT.1.S22 - Expression values	WT NT - WT.2.S23 - Expression values	WT NT - WT.3.S24 - Expression values	WT NT - Means	yccT NT - yccT.1.S28 - Expression values	yccT NT - yccT.2.S29 - Expression values	yccT NT - yccT.3.S30 - Expression values	yccT NT - Means
yigM	77	9	-10.3333	-1.0775	0.170957	-1.22947	1.229469319	0.298035733	-0.29804	0.356643	113	190	128	143.6667	132	123	145	133.3333
yigN	381	100	-201.333	-1.16219	0.030033	-1.31891	1.318905327	0.399341009	-0.39934	0.107668	1573	1477	1278	1442.667	1316	1192	1216	1241.333
yigP	563	265	-366	-1.18869	0.03727	-1.3616	1.361596419	0.445299147	-0.4453	0.126351	2176	2375	2366	2305.667	2096	1812	1911	1939.667
yigQ	94	36	47	1.076672	0.642206	-1.05886	1.058856121	0.082506566	-0.08251	0.802712	608	644	587	613	681	621	678	660
yigW	205	77	-70.6667	-1.13359	0.057858	-1.30673	1.306734271	0.385965794	-0.38597	0.173095	511	610	678	599.6667	588	473	526	529
yigZ	485	155	-290.333	-1.39591	0.000686	-1.6079	1.60789943	0.685177172	-0.68518	0.005694	888	1029	1154	1023.667	798	669	733	733.3333
yihA	298	149	-203.667	-1.14666	0.038245	-1.30618	1.306177796	0.385351289	-0.38535	0.128346	1620	1621	1536	1592.333	1456	1387	1323	1388.667
yihD	661	85	-289.667	-1.12813	0.097651	-1.28135	1.28134661	0.357660784	-0.35766	0.246689	2806	2371	2474	2550.333	2286	2351	2145	2260.667
yihE	933	151	153	1.030553	0.534592	-1.10234	1.102335388	0.140563233	-0.14056	0.726308	5453	4980	4590	5007.667	5523	5055	4904	5160.667
yihG	63	13	16.33333	1.05062	0.51382	-1.08133	1.081326403	0.112802073	-0.1128	0.714158	323	354	291	322.6667	336	335	346	339
yihI	195	49	104	1.191176	0.756008	1.041313	1.041313312	0.058404215	0.058404	0.880907	559	495	578	544	690	608	646	648
yihO	38	10	4.333333	1.048872	0.563645	-1.08191	1.081909401	0.113579693	-0.11358	0.745911	95	101	70	88.66667	108	85	86	93
yihP	38	25	-11.3333	-1.08153	0.107017	-1.2401	1.240096976	0.310452944	-0.31045	0.262419	127	164	160	150.3333	152	126	139	139
yihQ	70	23	-20.3333	-1.08777	0.088458	-1.24635	1.246348511	0.31770754	-0.31771	0.231765	246	239	271	252	271	223	201	231.6667
yihR	35	11	-14.6667	-1.10732	0.06829	-1.26583	1.265825879	0.340078968	-0.34008	0.195726	133	164	157	151.3333	137	129	144	136.6667
yihS	39	25	-26	-1.38049	0.000417	-1.57298	1.572984789	0.653504719	-0.6535	0.003756	92	99	92	94.33333	78	60	67	68.33333
yihT	21	9	-11.6667	-1.36842	0.00653	-1.55838	1.558378796	0.640045953	-0.64005	0.033105	41	45	44	43.33333	39	24	32	31.66667
yihU	7	2	-3.33333	-1.16393	0.160518	-1.3207	1.320700217	0.401303029	-0.4013	0.341618	23	25	23	23.66667	21	22	18	20.33333
yihV	45	9	18.66667	1.149333	0.91666	1.014653	1.014653368	0.020986949	0.020987	0.981803	137	128	110	125	143	155	133	143.6667
yihW	537	167	-244	-1.13341	0.070385	-1.28235	1.282353897	0.358794464	-0.35879	0.1993	2274	2208	1737	2073	1904	1846	1737	1829
yihX	384	77	-129.667	-1.1186	0.064634	-1.27497	1.274966633	0.350459491	-0.35046	0.18793	1148	1420	1101	1223	1173	1036	1071	1093.333
yihZ	73	24	-27	-1.05136	0.150307	-1.20296	1.202956662	0.266584669	-0.26658	0.326155	533	543	582	552.6667	549	509	519	525.6667
yiiD	320	114	-88.6667	-1.05397	0.172608	-1.20551	1.205510935	0.269644737	-0.26964	0.359581	1606	1866	1723	1731.667	1720	1546	1663	1643
yiiG	16	3	9	2.8	0.004049	2.439761	2.439760957	1.286739802	1.28674	0.022798	7	6	2	5	15	18	9	14
yiiL	258	145	108.6667	1.124809	0.964636	-1.006	1.00599839	0.008627996	-0.00863	1	1011	839	762	870.6667	1020	984	934	979.3333
yiiM	191	6	-105.333	-1.31537	0.005525	-1.5167	1.516702551	0.600938179	-0.60094	0.028906	338	475	505	439.3333	344	314	344	334
yiiP	278	160	131	1.170795	0.78761	1.034281	1.034280926	0.048628096	0.048628	0.899922	905	728	668	767	946	860	888	898
yiiQ	123	35	48.66667	1.212518	0.63683	1.065779	1.065779323	0.091908749	0.091909	0.798922	275	176	236	229	299	271	263	277.6667
yiiR	142	52	31	1.097587	0.830005	-1.02821	1.028205043	0.040127993	-0.04013	0.930841	399	297	257	317.6667	368	329	349	348.6667
yiiU	1691	622	-1100.33	-1.33625	0.00929	-1.52662	1.526619861	0.610340866	-0.61034	0.043929	4003	4736	4379	4372.667	3381	3391	3045	3272.333
yijC	447	70	-5	-1.00314	0.32855	-1.13739	1.137392359	0.185730018	-0.18573	0.54248	1876	1491	1429	1598.667	1725	1495	1561	1593.667
yijD	247	101	-124	-1.0904	0.09161	-1.24321	1.2432116	0.31407187	-0.31407	0.23703	1552	1472	1463	1495.667	1448	1305	1362	1371.667
yijF	719	280	-421	-1.50259	0.000114	-1.71268	1.712676986	0.776253082	-0.77625	0.001313	1105	1542	1129	1258.667	865	825	823	837.6667
yijO	91	15	32.66667	1.088368	0.697933	-1.04673	1.046728221	0.065886901	-0.06589	0.840366	387	373	349	369.6667	440	372	395	402.3333
yijP	491	370	351	1.385997	0.121986	1.22429	1.22428968	0.291944955	0.291945	0.285537	1080	793	855	909.3333	1225	1284	1272	1260.333
yjaB	74	14	-17.3333	-1.03506	0.184732	-1.17839	1.178387846	0.236814455	-0.23681	0.373708	519	515	501	511.6667	515	521	447	494.3333

Feature ID	Experiment - Range (original values)	Experiment - IQR (original values)	Experiment - Difference (original values)	Experiment - Fold Change (original values)	EDGE test: yccT NT vs WT NT , tagwise dispersions - P-value	EDGE test: yccT NT vs WT NT , tagwise dispersions - Fold change	yccT NT vs WT NT ABS|FC|	yccT NT vs WT NT Log2FC	yccT NT vs WT NT Log2FC +-	EDGE test: yccT NT vs WT NT , tagwise dispersions - FDR p-value correction	WT NT - WT.1.S22 - Expression values	WT NT - WT.2.S23 - Expression values	WT NT - WT.3.S24 - Expression values	WT NT - Means	yccT NT - yccT.1.S28 - Expression values	yccT NT - yccT.2.S29 - Expression values	yccT NT - yccT.3.S30 - Expression values	yccT NT - Means
yjaD	110	31	-45	-1.06341	0.127252	-1.21101	1.211008666	0.276209189	-0.27621	0.293659	737	800	727	754.6667	690	704	735	709.6667
yjaG	206	76	-69.6667	-1.04417	0.184235	-1.19355	1.193552497	0.255262024	-0.25526	0.373404	1589	1702	1650	1647	1662	1496	1574	1577.333
yjaH	1492	497	-438	-1.07497	0.211314	-1.23592	1.235918751	0.305583904	-0.30558	0.412482	5667	6203	6970	6280	6164	5478	5884	5842
yjbA	38	31	21.33333	1.188791	0.701905	1.050156	1.050155716	0.070603265	0.070603	0.843099	137	103	99	113	134	132	137	134.3333
yjbB	231	20	20.33333	1.020212	0.393928	-1.11177	1.111767643	0.152855299	-0.15286	0.610313	1119	1011	888	1006	1097	991	991	1026.333
yjbC	375	151	244.6667	1.248645	0.467734	1.094727	1.094726945	0.130571067	0.130571	0.675751	1033	966	953	984	1328	1117	1241	1228.667
yjbD	164	82	-39.3333	-1.04419	0.170182	-1.19016	1.190159097	0.251154441	-0.25115	0.355696	1011	847	930	929.3333	960	862	848	890
yjbE	30	3	5.333333	1.166667	0.836756	1.036497	1.03649716	0.051716164	0.051716	0.936516	47	32	17	32	43	35	34	37.33333
yjbF	15	5	8.666667	2.3	0.015308	2.011623	2.011623264	1.008360143	1.00836	0.063928	11	3	6	6.666667	17	18	11	15.33333
yjbG	10	6	6.333333	1.527778	0.25163	1.341512	1.341511636	0.423859568	0.42386	0.462913	10	16	10	12	20	20	15	18.33333
yjbH	113	61	43	1.160248	0.820786	1.028871	1.028871008	0.041062119	0.041062	0.923541	329	260	216	268.3333	324	321	289	311.3333
yjbJ	341	159	173.3333	1.306243	0.258685	1.162646	1.162646183	0.217412122	0.217412	0.470584	716	557	425	566	766	765	687	739.3333
yjbN	45	13	3	1.006198	0.306264	-1.13013	1.13012821	0.176486452	-0.17649	0.521303	499	499	454	484	494	481	486	487
yjbO	27	11	4.333333	1.074713	0.756237	-1.0515	1.051502925	0.072452863	-0.07245	0.880907	72	57	45	58	65	68	54	62.33333
yjbQ	343	198	158.6667	1.16568	0.799761	1.033289	1.033289096	0.047243951	0.047244	0.907024	1151	914	808	957.6667	1104	1133	1112	1116.333
yjbR	210	140	94.66667	1.144162	0.933143	1.010724	1.010724374	0.015389625	0.01539	0.989486	777	620	573	656.6667	783	711	760	751.3333
yjcB	24	7	16.33333	1.521277	0.080087	1.334714	1.334713527	0.416530126	0.41653	0.217869	32	29	33	31.33333	51	39	53	47.66667
yjcC	116	66	78.33333	1.693215	0.001054	1.499039	1.499039302	0.584038208	0.584038	0.007943	136	118	85	113	201	189	184	191.3333
yjcD	133	55	90.66667	1.412747	0.07619	1.235545	1.235544818	0.305147344	0.305147	0.20995	222	211	226	219.6667	344	277	310	310.3333
yjcE	417	102	33.33333	1.032144	0.497606	-1.09309	1.093094573	0.128418226	-0.12842	0.699966	1204	1120	787	1037	1149	1018	1044	1070.333
yjcG	237	134	133.6667	1.628527	0.009768	1.45416	1.454160023	0.540186039	0.540186	0.045559	298	189	151	212.6667	323	328	388	346.3333
yjcH	51	25	30	2.139241	0.002329	1.910885	1.910884775	0.934240787	0.934241	0.014579	44	19	16	26.33333	44	58	67	56.33333
yjcO	140	57	58.33333	1.135659	0.996259	1.001224	1.00122423	0.00176511	0.001765	1	483	423	384	430	524	480	461	488.3333
yjdB	236	113	93	1.138256	0.970561	1.005266	1.005265769	0.007576967	0.007577	1	794	573	651	672.6667	724	809	764	765.6667
yjdC	152	82	-11.3333	-1.01579	0.246696	-1.16069	1.160685932	0.214977648	-0.21498	0.45756	784	645	758	729	797	676	680	717.6667
yjdE	188	91	107.6667	1.371692	0.109111	1.216652	1.216652419	0.282917069	0.282917	0.266045	356	287	226	289.6667	400	378	414	397.3333
yjdL	1669	701	-1189	-2.13816	1.26E-08	-2.45707	2.457066099	1.296936669	-1.29694	6.01E-07	1734	2666	2301	2233.667	1104	997	1033	1044.667
yjeA	123	9	57.33333	1.068308	0.605847	-1.06586	1.065858672	0.092016156	-0.09202	0.777315	858	849	811	839.3333	934	905	851	896.6667
yjeB	760	391	367.6667	1.162493	0.856539	1.027185	1.027185203	0.038696325	0.038696	0.948357	2598	2207	1983	2262.667	2743	2656	2492	2630.333
yjeE	198	71	22.66667	1.031865	0.453771	-1.09787	1.097871028	0.134708585	-0.13471	0.662928	839	654	641	711.3333	781	725	696	734
yjeF	181	45	-47.6667	-1.06578	0.118294	-1.20858	1.20858329	0.2733169	-0.27332	0.280149	875	748	694	772.3333	765	706	703	724.6667
yjeH	80	6	-22	-1.08098	0.086893	-1.22574	1.225740553	0.293653642	-0.29365	0.229317	297	332	252	293.6667	268	273	274	271.6667
yjeI	1116	266	168.6667	1.040079	0.58897	-1.08839	1.088385769	0.122189998	-0.12219	0.76537	4806	4129	3690	4208.333	4571	4395	4165	4377
yjeJ	333	205	185.3333	1.258124	0.39776	1.113258	1.113258394	0.15478849	0.154788	0.61347	867	674	613	718	946	885	879	903.3333
yjeK	296	213	214	1.399751	0.084743	1.235961	1.235961027	0.305633252	0.305633	0.225659	630	501	475	535.3333	771	714	763	749.3333
yjeM	200	120	137.3333	1.570637	0.008023	1.381052	1.381052325	0.465767981	0.465768	0.039109	276	211	235	240.6667	411	368	355	378

Feature ID	Experiment - Range (original values)	Experiment - IQR (original values)	Experiment - Difference (original values)	Experiment - Fold Change (original values)	EDGE test: yccT NT vs WT NT , tagwise dispersions - P-value	EDGE test: yccT NT vs WT NT , tagwise dispersions - Fold change	yccT NT vs WT NT ABS|FC|	yccT NT vs WT NT Log2FC	yccT NT vs WT NT Log2FC +-	EDGE test: yccT NT vs WT NT , tagwise dispersions - FDR p-value correction	WT NT - WT.1.S22 - Expression values	WT NT - WT.2.S23 - Expression values	WT NT - WT.3.S24 - Expression values	WT NT - Means	yccT NT - yccT.1.S28 - Expression values	yccT NT - yccT.2.S29 - Expression values	yccT NT - yccT.3.S30 - Expression values	yccT NT - Means
yjeO	38	16	-9	-1.05389	0.137268	-1.1974	1.197399159	0.259904163	-0.2599	0.30677	194	159	175	176	169	176	156	167
yjeP	191	95	-20	-1.01847	0.236047	-1.1669	1.166901608	0.22268292	-0.22268	0.444591	1026	1161	1121	1102.667	1163	972	1113	1082.667
yjeQ	333	143	-92.6667	-1.04464	0.21789	-1.18729	1.187285312	0.247666666	-0.24767	0.421051	2316	2190	1999	2168.333	2142	1983	2102	2075.667
yjeS	148	56	-25	-1.04	0.189418	-1.17975	1.179751974	0.238483586	-0.23848	0.380466	652	723	575	650	623	656	596	625
yjfC	91	52	63.33333	1.405117	0.056339	1.239279	1.239279324	0.309501397	0.309501	0.169936	179	154	136	156.3333	227	206	226	219.6667
yjfH	457	354	-236	-1.13705	0.067044	-1.30587	1.305871687	0.385013147	-0.38501	0.193532	1779	2036	2059	1958	1959	1605	1602	1722
yjfI	6	4	3	1.5	0.471868	1.316813	1.316812591	0.397050036	0.39705	0.678436	9	5	4	6	8	9	10	9
yjfJ	17	4	7	1.4375	0.293552	1.263796	1.263796022	0.33776363	0.337764	0.510089	19	15	14	16	22	16	31	23
yjfK	13	2	4.333333	1.203125	0.821584	1.06027	1.060270092	0.084431822	0.084432	0.924153	24	25	15	21.33333	28	23	26	25.66667
yjfL	21	15	17.66667	1.688312	0.020566	1.482307	1.482307338	0.567844603	0.567845	0.079681	24	27	26	25.66667	44	41	45	43.33333
yjfM	49	36	24.33333	1.321586	0.2705	1.168252	1.168252275	0.224351847	0.224352	0.483691	103	60	64	75.66667	109	91	100	100
yjfN	3441	523	-1551.33	-1.15487	0.099906	-1.31003	1.310026881	0.389596416	-0.3896	0.250812	12925	11167	10613	11568.33	10477	10090	9484	10017
yjfO	2057	1003	-1367.67	-1.24282	0.029284	-1.41517	1.415169137	0.50097449	-0.50097	0.105493	7499	6597	6904	7000	5861	5594	5442	5632.333
yjfP	247	176	118.6667	1.189462	0.69239	1.051719	1.051718779	0.072748991	0.072749	0.836646	772	563	544	626.3333	791	705	739	745
yjfQ	71	12	24.33333	1.03976	0.461337	-1.09394	1.093943951	0.129538823	-0.12954	0.66967	635	614	587	612	626	625	658	636.3333
yjfR	20	4	-1.33333	-1.0109	0.256154	-1.15144	1.151438815	0.203437752	-0.20344	0.468242	118	134	119	123.6667	122	114	131	122.3333
yjfY	54	27	35	1.530303	0.015941	1.349551	1.349551382	0.432479906	0.43248	0.065891	76	69	53	66	107	96	100	101
yjgA	99	70	-80.6667	-1.21704	0.009532	-1.38746	1.387456538	0.47244258	-0.47244	0.044883	438	461	458	452.3333	362	385	368	371.6667
yjgB	136	16	29.33333	1.059946	0.57005	-1.0702	1.070199803	0.097880168	-0.09788	0.75019	532	514	422	489.3333	558	498	500	518.6667
yjgD	546	222	-378.667	-1.335	0.002166	-1.52929	1.52928791	0.61286004	-0.61286	0.013867	1371	1565	1591	1509	1197	1149	1045	1130.333
yjgF	6362	3245	-4159	-1.4979	0.002069	-1.72392	1.72391792	0.785691086	-0.78569	0.01342	11438	11826	14272	12512	8956	7910	8193	8353
yjgK	316	108	-162	-1.31015	0.003387	-1.50303	1.503031197	0.587874954	-0.58787	0.019983	649	646	758	684.3333	587	538	442	522.3333
yjgM	134	87	85.33333	1.334641	0.174018	1.17736	1.177359702	0.235555154	0.235555	0.361728	302	226	237	255	337	324	360	340.3333
yjgN	286	161	176.6667	1.782866	0.00097	1.580812	1.580811718	0.660665547	0.660666	0.007472	310	191	176	225.6667	462	352	393	402.3333
yjgP	653	74	2.333333	1.001004	0.396889	-1.1308	1.130799621	0.177343304	-0.17734	0.612972	2657	2309	2004	2323.333	2453	2289	2235	2325.667
yjgQ	313	64	119.3333	1.098759	0.79921	-1.03273	1.032728538	0.046461078	-0.04646	0.906631	1251	1315	1059	1208.333	1372	1327	1284	1327.667
yjhP	580	136	289	1.210693	0.621842	1.066153	1.066152977	0.092414458	0.092414	0.787112	1442	1479	1194	1371.667	1774	1578	1630	1660.667
yjiA	463	81	-245.333	-1.20236	0.017001	-1.36077	1.360769534	0.444422746	-0.44442	0.069048	1662	1412	1299	1457.667	1199	1218	1220	1212.333
yjiE	209	142	157.3333	1.900763	1.83E-05	1.684424	1.684423913	0.752255262	0.752255	0.000303	219	170	135	174.6667	344	312	340	332
yjiG	185	87	116	1.881013	6.07E-05	1.662343	1.66234283	0.733217945	0.733218	0.0008	149	137	109	131.6667	224	225	294	247.6667
yjiH	250	143	176	2.454545	1.84E-08	2.166551	2.166550902	1.115400132	1.1154	8.34E-07	153	102	108	121	288	251	352	297
yjiJ	117	76	88.33333	1.462478	0.03297	1.285649	1.285648588	0.362496358	0.362496	0.114941	206	182	185	191	278	261	299	279.3333
yjiN	69	10	29	1.170923	0.855323	1.024886	1.024885973	0.035463407	0.035463	0.947992	176	167	166	169.6667	233	164	199	198.6667
yjiO	242	106	161.3333	1.773163	0.000972	1.550487	1.550486869	0.632721308	0.632721	0.007475	194	218	214	208.6667	354	320	436	370
yjiS	11	5	7.333333	1.578947	0.164393	1.38814	1.388139816	0.473152887	0.473153	0.347907	14	12	12	12.66667	17	23	20	20
yjiX	148	11	-60	-1.38627	0.001529	-1.55556	1.555561154	0.637435113	-0.63744	0.010658	297	183	166	215.3333	155	162	149	155.3333

Feature ID	Experiment - Range (original values)	Experiment - IQR (original values)	Experiment - Difference (original values)	Experiment - Fold Change (original values)	EDGE test: yccT NT vs WT NT , tagwise dispersions - P-value	EDGE test: yccT NT vs WT NT , tagwise dispersions - Fold change	yccT NT vs WT NT ABS|FC|	yccT NT vs WT NT Log2FC	yccT NT vs WT NT Log2FC +-	EDGE test: yccT NT vs WT NT , tagwise dispersions - FDR p-value correction	WT NT - WT.1.S22 - Expression values	WT NT - WT.2.S23 - Expression values	WT NT - WT.3.S24 - Expression values	WT NT - Means	yccT NT - yccT.1.S28 - Expression values	yccT NT - yccT.2.S29 - Expression values	yccT NT - yccT.3.S30 - Expression values	yccT NT - Means
yjiY	2936	268	-760.333	-2.05553	0.034209	-2.19622	2.196219581	1.135022304	-1.13502	0.118606	3364	645	433	1480.667	701	1032	428	720.3333
yjjA	157	13	3	1.003589	0.307975	-1.13536	1.135364364	0.183155365	-0.18316	0.523149	811	927	770	836	894	805	818	839
yjjG	130	68	-2.33333	-1.00499	0.299762	-1.15643	1.156430687	0.209678798	-0.20968	0.515478	386	509	516	470.3333	503	435	466	468
yjjI	5161	2011	-3300.33	-1.6591	0.00022	-1.89512	1.895116876	0.922286825	-0.92229	0.002173	7055	10008	7860	8307.667	5131	5044	4847	5007.333
yjjK	1670	44	-918	-1.34738	0.012443	-1.55611	1.556106379	0.63794069	-0.63794	0.054589	2764	3872	4046	3560.667	2798	2376	2754	2642.667
yjjP	136	70	16.66667	1.025304	0.402607	-1.12014	1.120144756	0.163685184	-0.16369	0.617037	585	670	721	658.6667	691	621	714	675.3333
yjjQ	12	7	4.333333	1.65	0.268523	1.458932	1.458931579	0.544912225	0.544912	0.481141	14	4	2	6.666667	8	11	14	11
yjjU	220	102	131.6667	1.233314	0.557136	1.079061	1.079061023	0.109776454	0.109776	0.741788	519	638	536	564.3333	739	620	729	696
yjjV	271	69	-96	-1.20339	0.024361	-1.38398	1.383975021	0.468817904	-0.46882	0.091093	457	671	576	568	526	400	490	472
yjjW	1577	668	-993.667	-1.6582	3.06E-05	-1.8952	1.895202572	0.922352062	-0.92235	0.000471	2141	3032	2337	2503.333	1601	1455	1473	1509.667
yjjX	37	6	-14	-1.05237	0.12659	-1.20109	1.201089938	0.264344185	-0.26434	0.292757	266	297	281	281.3333	272	260	270	267.3333
yjjY	200	18	0	-1	0.338248	-1.1378	1.137800931	0.186248166	-0.18625	0.551833	1565	1738	1538	1613.667	1575	1683	1583	1613.667
ylaB	291	140	158	1.283493	0.300025	1.139282	1.139281522	0.188124289	0.188124	0.51552	669	562	441	557.3333	702	712	732	715.3333
ylaC	533	238	310	1.359073	0.14985	1.202789	1.202789002	0.266383581	0.266384	0.325833	1046	836	708	863.3333	1241	1074	1205	1173.333
ylbA	282	179	142.3333	1.181393	0.743671	1.042596	1.042595645	0.060179738	0.06018	0.873134	945	675	734	784.6667	957	913	911	927
ylbE	22	10	7	1.189189	0.793105	1.049565	1.049565429	0.069792106	0.069792	0.903592	46	39	26	37	48	36	48	44
ylbF	20	7	11.33333	1.68	0.068146	1.473282	1.473282429	0.559034022	0.559034	0.19544	17	14	19	16.66667	26	24	34	28
yliA	224	104	71.66667	1.089583	0.701656	-1.05191	1.051906414	0.073006357	-0.07301	0.843099	749	853	798	800	958	734	923	871.6667
yliB	206	99	-27.3333	-1.02482	0.222696	-1.17378	1.173775281	0.231156231	-0.23116	0.426986	1141	1042	1203	1128.667	1168	997	1139	1101.333
yliC	125	28	49	1.146123	0.998333	1.001732	1.001731934	0.002496491	0.002496	1	349	321	336	335.3333	442	317	394	384.3333
yliD	103	65	67	1.237589	0.477929	1.089062	1.08906202	0.123086115	0.123086	0.684013	304	271	271	282	336	337	374	349
yliG	209	124	150	1.582147	0.004179	1.395639	1.395639383	0.480926214	0.480926	0.023331	298	252	223	257.6667	432	376	415	407.6667
yliH	1927	1585	1332.333	1.727918	0.015267	1.546334	1.546333698	0.628851685	0.628852	0.063818	2693	1388	1410	1830.333	3178	3315	2995	3162.667
yliJ	431	123	77.33333	1.039462	0.527998	-1.09083	1.090831618	0.125428423	-0.12543	0.722435	2176	1958	1745	1959.667	2166	2034	1911	2037
yljA	723	306	-97	-1.03963	0.296598	-1.17304	1.173043948	0.230257065	-0.23026	0.512736	2778	2789	2066	2544.333	2410	2619	2313	2447.333
ymbA	145	63	-36	-1.10445	0.101243	-1.24228	1.242279805	0.312990156	-0.31299	0.253594	431	425	286	380.6667	333	382	319	344.6667
ymdA	85	43	-58	-1.61268	9.79E-06	-1.83701	1.837011	0.877360265	-0.87736	0.000181	135	170	153	152.6667	92	107	85	94.66667
ymdC	466	333	311	1.287785	0.300167	1.138831	1.138831475	0.187554273	0.187554	0.51552	1258	1044	940	1080.667	1392	1377	1406	1391.667
ymdF	203	147	73.33333	1.28133	0.402687	1.145281	1.145280537	0.195701031	0.195701	0.617037	396	193	193	260.6667	368	340	294	334
ymfB	107	40	60	1.21102	0.635943	1.060727	1.06072724	0.085053723	0.085054	0.798261	270	305	278	284.3333	377	338	318	344.3333
ymfC	146	50	69.33333	1.231368	0.476993	1.088667	1.088667337	0.122563178	0.122563	0.683086	351	301	247	299.6667	376	338	393	369
ymgE	34	18	10.66667	1.244275	0.565984	1.104636	1.104635846	0.14357085	0.143571	0.747261	64	37	30	43.66667	59	55	49	54.33333
ynaF	12060	9018	8198.667	1.742252	0.025171	1.555746	1.555745685	0.637606245	0.637606	0.093177	16312	7805	9020	11045.67	19865	19830	18038	19244.33
ynaI	256	171	184.6667	1.298491	0.274417	1.142866	1.142866297	0.192656634	0.192657	0.487547	675	598	583	618.6667	802	769	839	803.3333
ynaJ	542	272	244.3333	1.21039	0.58288	1.074789	1.074788922	0.104053357	0.104053	0.761737	1434	1132	918	1161.333	1460	1404	1353	1405.667
ynbE	13	8	8.666667	1.252427	0.570833	1.102326	1.102325987	0.14055093	0.140551	0.750362	38	31	34	34.33333	42	43	44	43

Feature ID	Experiment - Range (original values)	Experiment - IQR (original values)	Experiment - Difference (original values)	Experiment - Fold Change (original values)	EDGE test: yccT NT vs WT NT , tagwise dispersions - P-value	EDGE test: yccT NT vs WT NT , tagwise dispersions - Fold change	yccT NT vs WT NT ABS|FC|	yccT NT vs WT NT Log2FC	yccT NT vs WT NT Log2FC +-	EDGE test: yccT NT vs WT NT , tagwise dispersions - FDR p-value correction	WT NT - WT.1.S22 - Expression values	WT NT - WT.2.S23 - Expression values	WT NT - WT.3.S24 - Expression values	WT NT - Means	yccT NT - yccT.1.S28 - Expression values	yccT NT - yccT.2.S29 - Expression values	yccT NT - yccT.3.S30 - Expression values	yccT NT - Means
yncA	359	171	172.6667	1.31413	0.237662	1.168732	1.168732147	0.224944327	0.224944	0.446686	705	534	410	549.6667	769	706	692	722.3333
yncB	123	36	24.66667	1.032918	0.428154	-1.10069	1.100694158	0.138413653	-0.13841	0.639913	778	787	683	749.3333	806	742	774	774
yncC	20	7	14	2.135135	0.005464	1.873308	1.873308061	0.905588167	0.905588	0.028685	14	13	10	12.33333	30	29	20	26.33333
yncD	57	22	28.33333	1.34	0.193593	1.189133	1.189132726	0.249909751	0.24991	0.387274	104	82	64	83.33333	100	121	114	111.6667
yncJ	20	8	10.66667	1.680851	0.068627	1.477219	1.477219029	0.562883752	0.562884	0.196057	15	20	12	15.66667	24	23	32	26.33333
yneA	19204	11921	13143	4.747648	0.006915	4.460022	4.460021696	2.157050728	2.157051	0.034556	9573	521	427	3507	19631	12442	17877	16650
yneB	19324	11344	12900.33	4.548272	0.005974	4.259977	4.259976822	2.090845581	2.090846	0.030922	9764	600	543	3635.667	19867	11944	17797	16536
yneC	3279	1855	2202.667	4.84186	0.002565	4.525957	4.52595669	2.178222779	2.178223	0.015874	1522	108	90	573.3333	3369	1963	2996	2776
yneG	21	2	-8	-1.06138	0.113265	-1.21042	1.210424755	0.275513398	-0.27551	0.272466	136	140	139	138.3333	135	119	137	130.3333
yneH	57	15	16.66667	1.076336	0.656963	-1.05612	1.056120794	0.078774852	-0.07877	0.814931	216	243	196	218.3333	231	221	253	235
yneI	181	16	40.33333	1.084675	0.758244	-1.0401	1.040103048	0.05672647	-0.05673	0.880907	555	499	375	476.3333	505	489	556	516.6667
yneJ	130	95	75.66667	1.253348	0.391368	1.110063	1.110062955	0.150641499	0.150641	0.60865	369	274	253	298.6667	383	375	365	374.3333
ynfA	35	14	8.333333	1.115207	0.923462	-1.01979	1.019786975	0.028267817	-0.02827	0.984632	87	65	65	72.33333	99	64	79	80.66667
ynfB	629	283	324.6667	1.225567	0.53858	1.08535	1.085350414	0.118160904	0.118161	0.728242	1697	1414	1207	1439.333	1836	1799	1657	1764
ynfC	343	279	162	1.190663	0.695057	1.052596	1.052596231	0.073952134	0.073952	0.837812	1062	748	739	849.6667	1082	926	1027	1011.667
ynfD	126	21	-23.3333	-1.04164	0.18111	-1.18326	1.18326342	0.242771285	-0.24277	0.369874	623	568	560	583.6667	581	603	497	560.3333
ynfI	698	141	-131.667	-1.11052	0.126176	-1.28646	1.286461332	0.363408093	-0.36341	0.29195	1219	1026	1724	1323	1303	1078	1193	1191.333
ynfK	732	263	-174.333	-1.05082	0.249948	-1.19515	1.195147826	0.257189074	-0.25719	0.461082	3568	3989	3257	3604.667	3581	3405	3305	3430.333
ynfL	77	25	39.66667	1.32337	0.194366	1.169276	1.169276219	0.225615779	0.225616	0.388119	146	122	100	122.6667	163	147	177	162.3333
ynfM	351	273	237.6667	2.250877	0.002068	2.042895	2.042895288	1.030615258	1.030615	0.01342	358	113	99	190	447	386	450	427.6667
ynhA	63	29	39.33333	1.379421	0.099103	1.21805	1.21805041	0.284573841	0.284574	0.249361	119	108	84	103.6667	147	137	145	143
ynhG	893	698	593.3333	1.614641	0.013462	1.440298	1.440297936	0.526367274	0.526367	0.057911	1358	798	740	965.3333	1633	1547	1496	1558.667
yniB	1066	398	421.3333	1.222731	0.567011	1.090376	1.090375961	0.124825661	0.124826	0.748192	2411	1919	1345	1891.667	2381	2317	2241	2313
yniC	153	94	87	1.172619	0.776345	1.036494	1.036493935	0.051711674	0.051712	0.892069	576	482	454	504	564	607	602	591
yoaA	290	130	212	1.230101	0.566137	1.076284	1.076284255	0.106059155	0.106059	0.747261	909	928	927	921.3333	1199	1057	1144	1133.333
yoaB	205	85	-121.667	-1.11673	0.060397	-1.27586	1.275859232	0.351469163	-0.35147	0.178771	1148	1134	1210	1164	1073	1049	1005	1042.333
yoaE	171	91	62.33333	1.103659	0.790269	-1.03457	1.034567218	0.049027383	-0.04903	0.901773	564	682	558	601.3333	729	607	655	663.6667
yoaG	23	13	16.33333	1.266304	0.442863	1.114049	1.114048988	0.155812674	0.155813	0.653672	65	57	62	61.33333	75	80	78	77.66667
yoaH	140	34	37.33333	1.132231	1	-1.00073	1.000728371	0.001050435	-0.00105	1	335	283	229	282.3333	369	278	312	319.6667
yobA	422	98	-6.33333	-1.00699	0.351626	-1.13605	1.136054253	0.184031734	-0.18403	0.567115	998	1080	658	912	951	853	913	905.6667
yobF	1976	302	-1030.67	-1.14811	0.106163	-1.31896	1.318958179	0.399398821	-0.3994	0.261184	7007	8151	8811	7989.667	7172	6835	6870	6959
yobG	195	147	138.6667	1.370107	0.12921	1.207461	1.207461476	0.271977161	0.271977	0.296753	438	328	358	374.6667	523	505	512	513.3333
yodD	50	25	35	1.801527	0.001319	1.5895	1.589500496	0.668573467	0.668573	0.009503	51	44	36	43.66667	81	86	69	78.66667
yohC	38	12	6.666667	1.138889	0.968486	1.010574	1.010574151	0.015175183	0.015175	1	69	44	31	48	61	47	56	54.66667
yohD	47	10	-20.3333	-1.16095	0.022452	-1.31453	1.314527942	0.394544809	-0.39454	0.08525	159	164	117	146.6667	133	123	123	126.3333
yohF	39	14	11.33333	1.085642	0.730336	-1.04402	1.044017184	0.062145458	-0.06215	0.862735	141	142	114	132.3333	150	153	128	143.6667

Feature ID	Experiment - Range (original values)	Experiment - IQR (original values)	Experiment - Difference (original values)	Experiment - Fold Change (original values)	EDGE test: yccT NT vs WT NT , tagwise dispersions - P-value	EDGE test: yccT NT vs WT NT , tagwise dispersions - Fold change	yccT NT vs WT NT ABS|FC|	yccT NT vs WT NT Log2FC	yccT NT vs WT NT Log2FC +-	EDGE test: yccT NT vs WT NT , tagwise dispersions - FDR p-value correction	WT NT - WT.1.S22 - Expression values	WT NT - WT.2.S23 - Expression values	WT NT - WT.3.S24 - Expression values	WT NT - Means	yccT NT - yccT.1.S28 - Expression values	yccT NT - yccT.2.S29 - Expression values	yccT NT - yccT.3.S30 - Expression values	yccT NT - Means
yohG	37	31	31.33333	1.764228	0.00132	1.553519	1.553518761	0.635539664	0.63554	0.009503	47	40	36	41	73	71	73	72.33333
yohI	33	20	18.66667	1.341463	0.228766	1.182002	1.182002263	0.241232798	0.241233	0.434861	67	48	49	54.66667	81	70	69	73.33333
yohJ	118	44	69	1.570248	0.011884	1.391824	1.391823703	0.476976482	0.476976	0.052762	155	122	86	121	204	166	200	190
yohK	153	76	77	1.340206	0.168929	1.193404	1.193404153	0.255082703	0.255083	0.353886	293	217	169	226.3333	300	322	288	303.3333
yohL	175	96	105	1.701559	0.006624	1.522748	1.522748171	0.606677372	0.606677	0.033508	221	131	97	149.6667	272	265	227	254.6667
yojI	300	123	-70	-1.10304	0.087448	-1.25299	1.252991609	0.325376753	-0.32538	0.229842	736	906	606	749.3333	739	613	686	679.3333
yojN	1358	784	873.6667	1.481713	0.064387	1.314688	1.314688189	0.394720669	0.394721	0.187578	2195	1818	1428	1813.667	2786	2602	2674	2687.333
ypeC	164	28	74.66667	1.215592	0.546176	1.075971	1.075971426	0.105639766	0.10564	0.734255	385	380	274	346.3333	417	408	438	421
ypfG	146	56	86.33333	1.519038	0.015916	1.343989	1.343989039	0.426521373	0.426521	0.065849	198	169	132	166.3333	278	255	225	252.6667
ypfI	224	110	20.66667	1.018742	0.37541	-1.11688	1.116876778	0.159470026	-0.15947	0.593451	1142	1177	989	1102.667	1213	1032	1125	1123.333
yqaA	209	52	45.33333	1.034179	0.446143	-1.10472	1.104719634	0.143680276	-0.14368	0.656327	1360	1284	1335	1326.333	1493	1285	1337	1371.667
yqaB	586	365	291.6667	1.161767	0.863822	1.023771	1.023771307	0.033893479	0.033893	0.952612	2068	1703	1638	1803	2224	1964	2096	2094.667
yqaE	29	14	11.33333	1.472222	0.205452	1.304861	1.304861247	0.383896405	0.383896	0.403885	37	21	14	24	35	28	43	35.33333
yqcB	59	7	24.66667	1.159827	0.937647	1.013304	1.01330434	0.019067544	0.019068	0.992526	141	158	164	154.3333	180	157	200	179
yqcC	48	10	22.33333	1.468531	0.141941	1.282579	1.282578655	0.359047303	0.359047	0.314156	52	33	58	47.66667	81	62	67	70
yqcD	264	90	118.6667	1.161013	0.822079	1.028891	1.028890808	0.041089882	0.04109	0.924153	850	760	601	737	842	860	865	855.6667
yqeF	150	100	74	1.110668	0.842784	-1.02547	1.025467589	0.036281894	-0.03628	0.94008	645	745	616	668.6667	757	705	766	742.6667
yqfA	1647	174	-553.667	-1.15617	0.094112	-1.30411	1.304112855	0.383068722	-0.38307	0.240702	4996	3820	3481	4099	3632	3655	3349	3545.333
yqfB	201	25	52	1.071494	0.664226	-1.05686	1.056861298	0.079786051	-0.07979	0.819355	757	783	642	727.3333	760	843	735	779.3333
yqgA	93	47	59.33333	1.504249	0.017254	1.330229	1.330229208	0.411674854	0.411675	0.069821	141	119	93	117.6667	179	166	186	177
yqgB	65	20	42	1.309582	0.261275	1.148409	1.148409267	0.199636877	0.199637	0.472921	140	130	137	135.6667	181	157	195	177.6667
yqgD	22	9	-2.66667	-1.07273	0.281605	-1.23254	1.232544367	0.30163958	-0.30164	0.496497	27	42	49	39.33333	41	32	37	36.66667
yqgE	338	84	158.6667	1.151737	0.938946	1.010256	1.010255962	0.014720866	0.014721	0.993427	1130	949	1058	1045.667	1287	1184	1142	1204.333
yqgF	169	74	102.3333	1.184384	0.734655	1.042364	1.042363927	0.059859063	0.059859	0.865693	591	559	515	555	684	655	633	657.3333
yqhA	329	124	200.6667	1.485876	0.026192	1.314187	1.314187142	0.394170732	0.394171	0.096176	466	450	323	413	652	574	615	613.6667
yqhC	170	99	85	1.1875	0.75901	1.040389	1.040388593	0.057122487	0.057122	0.881222	422	521	417	453.3333	587	493	535	538.3333
yqhD	255	73	8	1.006612	0.339903	-1.12687	1.126866177	0.172316196	-0.17232	0.553593	1374	1137	1119	1210	1263	1210	1181	1218
yqhE	209	19	22	1.022642	0.401489	-1.10959	1.109593873	0.150031727	-0.15003	0.616499	1081	962	872	971.6667	1064	968	949	993.6667
yqiA	461	119	177.3333	1.112664	0.89261	-1.01829	1.018292318	0.026151771	-0.02615	0.968605	1641	1760	1321	1574	1767	1705	1782	1751.333
yqiB	121	61	-48.6667	-1.07476	0.116637	-1.22657	1.226568928	0.29462831	-0.29463	0.277562	657	739	703	699.6667	618	637	698	651
yqiC	563	255	124	1.102677	0.879234	-1.02093	1.020932067	0.029886872	-0.02989	0.961635	1544	1098	981	1207.667	1395	1353	1247	1331.667
yqiE	151	39	-74.6667	-1.1381	0.048703	-1.2985	1.298498555	0.376844408	-0.37684	0.153246	557	676	613	615.3333	529	568	525	540.6667
yqiK	375	92	196.3333	1.241295	0.4711	1.093006	1.093005657	0.128300868	0.128301	0.677962	898	826	717	813.6667	1092	918	1020	1010
yqjA	346	119	137.3333	1.137977	0.986686	1.002402	1.00240196	0.003461141	0.003461	1	1129	959	898	995.3333	1244	1078	1076	1132.667
yqjB	87	35	4	1.007059	0.319457	-1.12996	1.129960359	0.176272162	-0.17627	0.534417	589	573	538	566.6667	563	531	618	570.6667
yqjC	213	1	51.33333	1.035889	0.483365	-1.09501	1.095014239	0.13094963	-0.13095	0.68865	1520	1464	1307	1430.333	1464	1518	1463	1481.667

Feature ID	Experiment - Range (original values)	Experiment - IQR (original values)	Experiment - Difference (original values)	Experiment - Fold Change (original values)	EDGE test: yccT NT vs WT NT , tagwise dispersions - P-value	EDGE test: yccT NT vs WT NT , tagwise dispersions - Fold change	yccT NT vs WT NT ABS|FC|	yccT NT vs WT NT Log2FC	yccT NT vs WT NT Log2FC +-	EDGE test: yccT NT vs WT NT , tagwise dispersions - FDR p-value correction	WT NT - WT.1.S22 - Expression values	WT NT - WT.2.S23 - Expression values	WT NT - WT.3.S24 - Expression values	WT NT - Means	yccT NT - yccT.1.S28 - Expression values	yccT NT - yccT.2.S29 - Expression values	yccT NT - yccT.3.S30 - Expression values	yccT NT - Means
yqjE	180	80	-57	-1.0285	0.250735	-1.17278	1.172780399	0.229932896	-0.22993	0.461957	2065	2109	1996	2056.667	2085	1929	1985	1999.667
yqjF	14	4	-1.66667	-1.03937	0.280592	-1.18079	1.180791737	0.23975453	-0.23975	0.49534	52	38	42	44	50	38	39	42.33333
yqjG	93	39	45.33333	1.136959	0.988025	1.002607	1.002606722	0.003755812	0.003756	1	367	328	298	331	391	379	359	376.3333
yqjH	18	4	-3.33333	-1.04292	0.192483	-1.18623	1.186225327	0.24637808	-0.24638	0.385576	79	86	78	81	83	82	68	77.66667
yqjI	210	124	-146.333	-1.339	0.000572	-1.52623	1.526231607	0.609973909	-0.60997	0.004859	556	631	547	578	451	421	423	431.6667
yqjK	159	47	-55	-1.06504	0.122125	-1.21806	1.21806207	0.284587651	-0.28459	0.285711	858	931	913	900.6667	860	772	905	845.6667
yraL	231	37	91.33333	1.16924	0.810318	1.030882	1.030882347	0.043879689	0.04388	0.914549	567	604	448	539.6667	647	567	679	631
yraM	521	100	310.6667	1.193723	0.73454	1.046524	1.04652386	0.065605205	0.065605	0.865693	1655	1623	1533	1603.667	2054	1723	1966	1914.333
yraN	74	16	27	1.092784	0.710278	-1.0463	1.046298894	0.065295041	-0.0653	0.847948	277	306	290	291	351	293	310	318
yraO	139	27	-26.6667	-1.02015	0.24088	-1.16267	1.162674229	0.217446923	-0.21745	0.451455	1416	1309	1325	1350	1395	1277	1298	1323.333
yraP	442	220	-285.333	-1.16405	0.042571	-1.33082	1.330818263	0.412313569	-0.41231	0.139314	1987	1963	2124	2024.667	1793	1743	1682	1739.333
yraR	57	8	-21.3333	-1.0575	0.133212	-1.20262	1.202624608	0.266186385	-0.26619	0.301153	390	404	383	392.3333	347	387	379	371
yrbA	80	21	15.66667	1.036805	0.463869	-1.09985	1.099847005	0.137302851	-0.1373	0.672139	389	469	419	425.6667	429	455	440	441.3333
yrbB	138	34	-70.3333	-1.12211	0.046352	-1.27747	1.277470238	0.35328968	-0.35329	0.148614	641	690	608	646.3333	574	552	602	576
yrbC	251	47	-103.667	-1.06984	0.134658	-1.21975	1.219754504	0.28659081	-0.28659	0.30334	1545	1675	1544	1588	1532	1497	1424	1484.333
yrbD	83	28	4.666667	1.010542	0.32803	-1.13174	1.131744378	0.17854814	-0.17855	0.542148	405	488	435	442.6667	482	416	444	447.3333
yrbE	89	60	59.33333	1.154783	0.907715	1.015254	1.015254142	0.021840913	0.021841	0.976471	412	358	380	383.3333	441	447	440	442.6667
yrbF	115	10	-11.6667	-1.01989	0.231166	-1.15619	1.156185918	0.209373406	-0.20937	0.438109	654	602	539	598.3333	588	591	581	586.6667
yrbG	166	36	7	1.009758	0.342587	-1.12387	1.123868808	0.168473636	-0.16847	0.557066	742	788	622	717.3333	749	706	718	724.3333
yrbH	317	98	-155.333	-1.1028	0.086451	-1.25943	1.259431476	0.332772629	-0.33277	0.228443	1573	1790	1636	1666.333	1579	1481	1473	1511
yrbI	272	23	-47	-1.0301	0.229367	-1.17596	1.175955371	0.23383331	-0.23383	0.435451	1517	1771	1537	1608.333	1671	1514	1499	1561.333
yrbK	270	134	-129.333	-1.08824	0.108661	-1.24662	1.246619912	0.318021662	-0.31802	0.265424	1469	1657	1659	1595	1571	1437	1389	1465.667
yrbL	115	10	50	1.116369	0.861365	-1.02221	1.02220563	0.031685442	-0.03169	0.951	442	414	433	429.6667	529	478	432	479.6667
yrdA	320	35	-125.667	-1.11397	0.061369	-1.26925	1.269245646	0.343971311	-0.34397	0.180921	1186	1367	1132	1228.333	1148	1047	1113	1102.667
yrdB	155	56	54.33333	1.103822	0.780719	-1.03675	1.036753512	0.052072935	-0.05207	0.894818	485	572	513	523.3333	640	524	569	577.6667
yrdC	719	170	367	1.196397	0.716114	1.051723	1.051723424	0.072755363	0.072755	0.851632	1922	2009	1675	1868.667	2394	2092	2221	2235.667
yrdD	2219	1410	1274	1.321447	0.319492	1.174534	1.17453419	0.232088709	0.232089	0.534417	4997	3763	3130	3963.333	5349	5173	5190	5237.333
yrfA	12	6	7.666667	1.69697	0.093946	1.491876	1.491876183	0.577127805	0.577128	0.240622	14	10	9	11	16	21	19	18.66667
yrfB	16	8	4.333333	1.232143	0.762189	1.083966	1.083966014	0.116319524	0.11632	0.882712	24	13	19	18.66667	29	24	16	23
yrfC	32	22	14	1.451613	0.209578	1.291015	1.291014799	0.368505539	0.368506	0.410358	52	21	20	31	51	41	43	45
yrfD	25	11	14.33333	1.346774	0.265451	1.186035	1.18603508	0.246146682	0.246147	0.477359	48	34	42	41.33333	53	59	55	55.66667
yrfE	117	96	72.66667	1.220202	0.541904	1.078241	1.078241408	0.10868022	0.10868	0.730911	398	290	302	330	404	407	397	402.6667
yrfF	587	148	160.6667	1.06437	0.668756	-1.06658	1.066577364	0.092988615	-0.09299	0.821302	2513	2781	2194	2496	2661	2620	2689	2656.667
yrfG	160	26	46.33333	1.081765	0.709538	-1.04848	1.04847731	0.068295641	-0.0683	0.847948	584	611	505	566.6667	574	600	665	613
yrfH	43	19	-2	-1.00922	0.26378	-1.14275	1.142751999	0.192512342	-0.19251	0.475513	234	232	191	219	210	229	212	217
yrfI	98	32	-40.3333	-1.08806	0.077137	-1.24098	1.240983339	0.311483746	-0.31148	0.212293	483	536	476	498.3333	485	438	451	458

Feature ID	Experiment - Range (original values)	Experiment - IQR (original values)	Experiment - Difference (original values)	Experiment - Fold Change (original values)	EDGE test: yccT NT vs WT NT , tagwise dispersions - P-value	EDGE test: yccT NT vs WT NT , tagwise dispersions - Fold change	yccT NT vs WT NT ABS|FC|	yccT NT vs WT NT Log2FC	yccT NT vs WT NT Log2FC +-	EDGE test: yccT NT vs WT NT , tagwise dispersions - FDR p-value correction	WT NT - WT.1.S22 - Expression values	WT NT - WT.2.S23 - Expression values	WT NT - WT.3.S24 - Expression values	WT NT - Means	yccT NT - yccT.1.S28 - Expression values	yccT NT - yccT.2.S29 - Expression values	yccT NT - yccT.3.S30 - Expression values	yccT NT - Means
ysaA	470	42	-267.333	-1.22415	0.010474	-1.38906	1.38906451	0.474113601	-0.47411	0.047846	1568	1561	1251	1460	1261	1098	1219	1192.667
yshA	5	0	2	1.6	0.569583	1.39448	1.394479796	0.479727031	0.479727	0.749798	2	4	4	3.333333	4	7	5	5.333333
ytfB	799	358	235.3333	1.141824	0.919234	1.01456	1.014560359	0.020854697	0.020855	0.982895	2130	1517	1331	1659.333	1970	1875	1839	1894.667
ytfE	70	25	36.33333	1.244944	0.41968	1.101053	1.101052589	0.138883377	0.138883	0.632303	169	157	119	148.3333	183	182	189	184.6667
ytfF	80	17	-46.6667	-1.23217	0.004031	-1.39853	1.398527559	0.483908683	-0.48391	0.022752	262	264	217	247.6667	201	184	218	201
ytfG	164	37	-94.3333	-1.81556	3.65E-06	-2.07915	2.079148124	1.055992544	-1.05599	7.99E-05	154	271	205	210	123	117	107	115.6667
ytfH	409	99	-226.667	-1.98837	7.91E-07	-2.27523	2.275231305	1.18601322	-1.18601	2.22E-05	326	628	414	456	242	227	219	229.3333
ytfJ	331	21	87.66667	1.112345	0.921872	-1.01338	1.013378551	0.019173199	-0.01917	0.983818	914	837	590	780.3333	921	852	831	868
ytfK	2418	627	-1293	-1.77735	5.31E-05	-2.01722	2.017222291	1.012370073	-1.01237	0.00072	2552	3985	2332	2956.333	1718	1705	1567	1663.333
ytfL	475	127	214	1.160661	0.853478	1.024207	1.024206886	0.034507165	0.034507	0.946945	1477	1368	1151	1332	1626	1495	1517	1546
ytfM	633	452	324.6667	1.158994	0.879383	1.022715	1.022714555	0.032403537	0.032404	0.961635	2407	1861	1858	2042	2491	2296	2313	2366.667
ytfN	763	167	234.3333	1.067746	0.68248	-1.06422	1.064216525	0.089791711	-0.08979	0.829877	3653	3620	3104	3459	3867	3486	3727	3693.333
ytfP	886	609	512.3333	1.288693	0.355269	1.13979	1.139790126	0.1887682	0.188768	0.570376	2179	1599	1546	1774.667	2432	2208	2221	2287
ytgA	53	40	35.33333	1.861789	0.003168	1.649598	1.649598397	0.722114836	0.722115	0.018986	62	27	34	41	75	74	80	76.33333
zipA	1616	1067	796	1.243029	0.544687	1.100275	1.100274559	0.137863574	0.137864	0.733366	4073	3006	2747	3275.333	4363	3753	4098	4071.333
zntA	766	356	-466.667	-1.32879	0.002123	-1.51476	1.514764301	0.599093326	-0.59909	0.013652	1792	2132	1734	1886	1514	1366	1378	1419.333
zntR	275	198	196.3333	1.509516	0.020662	1.337678	1.337678064	0.419730947	0.419731	0.079959	472	355	329	385.3333	588	604	553	581.6667
znuA	458	203	173.3333	1.10486	0.84006	-1.02836	1.028357473	0.040341855	-0.04034	0.938119	1893	1435	1631	1653	1838	1807	1834	1826.333
znuC	357	111	13	1.012504	0.393831	-1.11783	1.117828327	0.16069864	-0.1607	0.610313	1110	1183	826	1039.667	1111	999	1048	1052.667
zraP	225	135	131.3333	1.232448	0.510796	1.084984	1.084984399	0.117674299	0.117674	0.710846	656	521	518	565	743	614	732	696.3333
zunB	226	64	-35.3333	-1.05458	0.154956	-1.20334	1.203341708	0.267046377	-0.26705	0.33296	641	792	615	682.6667	697	566	679	647.3333
zur	282	22	60	1.059445	0.603909	-1.06759	1.067587145	0.094353839	-0.09435	0.77698	1135	1040	853	1009.333	1090	1062	1056	1069.333
zwf	2145	619	-1398.67	-1.66933	0.00021	-1.92042	1.920416957	0.94141958	-0.94142	0.002105	2673	4138	3654	3488.333	2222	1993	2054	2089.667
16S rRNA	0	0	0	1	1	1	1	0	0	1	0	0	0	0	0	0	0	0
16S rRNA-[illegible]	1	0	0	-1	1	-1.09684	1.096837723	0.133350094	-0.13335	1	1	0	0	0.333333	1	0	0	0.333333
16S rRNA-[illegible]	2	0	-0.66667	#DIV/0!	0.501581	-6.70375	6.703753929	2.744969194	-2.74497	0.70377	0	0	2	0.666667	0	0	0	0
16S rRNA-[illegible]	17	8	-1	-1.09677	0.621985	-1.22389	1.223887607	0.291471078	-0.29147	0.787112	22	7	5	11.33333	13	13	5	10.33333
16S rRNA-[illegible]	0	0	0	1	1	1	1	0	0	1	0	0	0	0	0	0	0	0
16S rRNA-[illegible]	2	0	-1	#DIV/0!	0.252295	-9.53365	9.533647807	3.25302833	-3.25303	0.463289	2	1	0	1	0	0	0	0
16S rRNA-[illegible]	0	0	0	1	1	1	1	0	0	1	0	0	0	0	0	0	0	0
23S rRNA	84	16	-10.3333	-1.24031	0.307715	-1.36055	1.360551129	0.444191173	-0.44419	0.523149	108	28	24	53.33333	44	41	44	43
23S rRNA-[illegible]	40	3	-9.33333	-1.82353	0.086243	-1.98344	1.983442325	0.988006447	-0.98801	0.228418	47	8	7	20.66667	11	10	13	11.33333
23S rRNA-[illegible]	4	2	2.333333	4.5	0.114473	3.521321	3.521321023	1.816116757	1.816117	0.274031	2	0	0	0.666667	4	2	3	3
23S rRNA-[illegible]	43	7	-3.66667	-1.15942	0.440554	-1.28	1.280003955	0.356148268	-0.35615	0.652002	54	15	11	26.66667	29	22	18	23
23S rRNA-[illegible]	2	0	-0.66667	#DIV/0!	0.501523	-6.68891	6.688906295	2.741770335	-2.74177	0.70377	2	0	0	0.666667	0	0	0	0
23S rRNA-[illegible]	777	112	-145.333	-1.73032	0.13644	-1.84666	1.846655976	0.884915123	-0.88492	0.305644	854	77	102	344.3333	166	217	214	199

Feature ID	Experiment - Range (original values)	Experiment - IQR (original values)	Experiment - Difference (original values)	Experiment - Fold Change (original values)	EDGE test: yccT NT vs WT NT , tagwise dispersions - P-value	EDGE test: yccT NT vs WT NT , tagwise dispersions - Fold change	yccT NT vs WT NT ABS|FC|	yccT NT vs WT NT Log2FC	yccT NT vs WT NT Log2FC +-	EDGE test: yccT NT vs WT NT , tagwise dispersions - FDR p-value correction	WT NT - WT.1.S22 - Expression values	WT NT - WT.2.S23 - Expression values	WT NT - WT.3.S24 - Expression values	WT NT - Means	yccT NT - yccT.1.S28 - Expression values	yccT NT - yccT.2.S29 - Expression values	yccT NT - yccT.3.S30 - Expression values	yccT NT - Means
23S rRNA-1	2	1	0	-1	1	-1.11317	1.113170758	0.154674916	-0.15467	1	0	1	1	0.666667	0	2	0	0.666667
5S rRNA	2	1	0.666667	2	0.690279	1.648086	1.648086467	0.720791936	0.720792	0.835588	1	0	1	0.666667	0	2	2	1.333333
5S rRNA-2	1	0	-0.33333	#DIV/0!	1	-3.8476	3.847601379	1.94395934	-1.94396	1	0	1	0	0.333333	0	0	0	0
5S rRNA-3	0	0	0	1	1	1	1	0	0	1	0	0	0	0	0	0	0	0
5S rRNA-4	1	0	0.333333	#DIV/0!	1	3.506333	3.506333446	1.8099632	1.809963	1	0	0	0	0	1	0	0	0.333333
5S rRNA-5	5	0	1	1.333333	0.828727	1.170223	1.170223137	0.226783648	0.226784	0.930121	5	4	0	3	4	4	4	4
5S rRNA-6	0	0	0	1	1	1	1	0	0	1	0	0	0	0	0	0	0	0
5S rRNA-7	1	0	0.333333	#DIV/0!	1	3.507335	3.507335327	1.810375369	1.810375	1	0	0	0	0	0	0	1	0.333333
5S rRNA-8	4	1	1.333333	5	0.22345	3.521622	3.521621515	1.816239865	1.81624	0.427692	0	1	0	0.333333	1	4	0	1.666667
SEN0050	330	129	183.3333	1.331325	0.184385	1.17824	1.178240033	0.236633477	0.236633	0.373404	647	568	445	553.3333	775	697	738	736.6667
SEN0275	5	1	2	2.2	0.312753	1.874937	1.874936731	0.906841913	0.906842	0.527563	2	2	1	1.666667	4	1	6	3.666667
SEN0280A	8	3	5	3.5	0.023393	2.966276	2.96627582	1.568652754	1.568653	0.088218	2	1	3	2	7	9	5	7
SEN0420	31	10	16.33333	1.335616	0.369979	1.164562	1.164561712	0.219787093	0.219787	0.58717	44	46	56	48.66667	75	48	72	65
SEN0534	70	41	47	1.762162	0.00096	1.557948	1.557947643	0.63964675	0.639647	0.007444	81	57	47	61.66667	111	98	117	108.6667
SEN0710	19	9	11.66667	1.777778	0.038183	1.562859	1.562858677	0.644187328	0.644187	0.128269	20	10	15	15	27	29	24	26.66667
SEN0918	19	14	9.666667	1.3625	0.335399	1.203364	1.203364436	0.267073625	0.267074	0.549723	39	20	21	26.66667	39	35	35	36.33333
SEN1011	94	44	54	1.625483	0.008203	1.443188	1.443188477	0.529259725	0.52926	0.039681	116	83	60	86.33333	140	127	154	140.3333
SEN1147	127	93	91.66667	1.67402	0.00249	1.481376	1.481376092	0.566937958	0.566938	0.015477	175	109	124	136	217	230	236	227.6667
SEN1152	119	48	80.33333	1.266298	0.365978	1.112239	1.112238585	0.153466292	0.153466	0.583116	328	291	286	301.6667	405	339	402	382
SEN1161	10	0	2.333333	1.35	0.679834	1.183776	1.183776381	0.243396577	0.243397	0.827568	9	5	6	6.666667	15	6	6	9
SEN1165	19	5	13.66667	2.078947	0.008118	1.827539	1.82753856	0.869901847	0.869902	0.039381	13	13	12	12.66667	18	31	30	26.33333
SEN1714	6	2	0	-1	1	-1.12559	1.12559313	0.170685428	-0.17069	1	6	0	1	2.333333	2	0	5	2.333333
SEN1759	26	16	2.333333	1.054688	0.674621	-1.08651	1.086510578	0.11970222	-0.1197	0.825117	35	51	42	42.66667	54	28	53	45
SEN1808A	1	0	0.666667	#DIV/0!	0.501425	6.013057	6.013056887	2.588098607	2.588099	0.70377	0	0	0	0	1	0	1	0.666667
SEN1968	47	32	35.33333	1.69281	0.002993	1.490165	1.490165369	0.57547244	0.575472	0.018119	59	47	47	51	86	79	94	86.33333
SEN1971A	17	9	10.33333	2.107143	0.018774	1.848542	1.848542052	0.886387863	0.886388	0.074342	16	5	7	9.333333	22	16	21	19.66667
SEN1972	14	3	5.666667	1.2	0.750406	1.059164	1.05916411	0.082926143	0.082926	0.877546	34	31	20	28.33333	34	34	34	34
SEN2225	15	9	8.666667	2	0.035088	1.754798	1.754797672	0.811304697	0.811305	0.120431	14	7	5	8.666667	20	16	16	17.33333
SEN2226	65	43	47.33333	1.940397	7.94E-05	1.712894	1.712894396	0.776436208	0.776436	0.000983	66	46	39	50.33333	104	89	100	97.66667
SEN2375A	58	21	26.66667	1.540541	0.083081	1.373032	1.373032406	0.457365676	0.457366	0.222167	72	51	25	49.33333	83	65	80	76
SEN2380A	9	3	4.666667	1.875	0.153486	1.637145	1.637144991	0.711182098	0.711182	0.330626	7	5	4	5.333333	13	9	8	10
SEN2922	55	29	35.33333	1.352159	0.145161	1.194676	1.194675939	0.256619334	0.256619	0.318735	115	102	84	100.3333	131	137	139	135.6667
SEN3573	152	22	55.33333	1.120116	0.915521	-1.01337	1.013371891	0.019163717	-0.01916	0.981058	499	484	399	460.6667	551	477	520	516
SEN3578	7	3	4.333333	2.083333	0.112109	1.808593	1.808593218	0.854867959	0.854868	0.270418	4	3	5	4	10	7	8	8.333333
SEN3742A	38	6	2.333333	1.017677	0.373243	-1.11616	1.116156183	0.158538917	-0.15854	0.590868	128	153	115	132	136	133	134	134.3333
SEN3859	722	496	515.3333	3.50974	0.000203	3.218804	3.218803927	1.686524698	1.686525	0.002069	449	87	80	205.3333	802	583	777	720.6667

Feature ID	Experiment - Range (original values)	Experiment - IQR (original values)	Experiment - Difference (original values)	Experiment - Fold Change (original values)	EDGE test: yccT NT vs WT NT , tagwise dispersions - P-value	EDGE test: yccT NT vs WT NT , tagwise dispersions - Fold change	yccT NT vs WT NT ABS|FC|	yccT NT vs WT NT Log2FC	yccT NT vs WT NT Log2FC +-	EDGE test: yccT NT vs WT NT , tagwise dispersions - FDR p-value correction	WT NT - WT.1.S22 - Expression values	WT NT - WT.2.S23 - Expression values	WT NT - WT.3.S24 - Expression values	WT NT - Means	yccT NT - yccT.1.S28 - Expression values	yccT NT - yccT.2.S29 - Expression values	yccT NT - yccT.3.S30 - Expression values	yccT NT - Means
SEN4240	132	21	32.33333	1.076498	0.683505	-1.05213	1.05212731	0.073309285	-0.07331	0.830895	451	465	352	422.6667	451	430	484	455
SEN4255	31	10	16.66667	1.078003	0.640486	-1.05886	1.058861191	0.082513475	-0.08251	0.801621	203	216	222	213.6667	231	234	226	230.3333
SEN4259	196	75	125.3333	1.54023	0.010882	1.361997	1.361997403	0.445723952	0.445724	0.049154	269	246	181	232	374	321	377	357.3333
