# Supplementary figures and images for: Transcriptional Profiling and Molecular Characterization of the yccT Mutant Link: A Novel STY1099 Protein with the Peroxide Stress Response and Cell Division of Salmonella enterica Serovar Enteritidis
Source: Biology (Basel). 2019 Nov 13;8(4):86. doi: 10.3390/biology8040086 (PMC6955953; doi:10.3390/biology8040086)

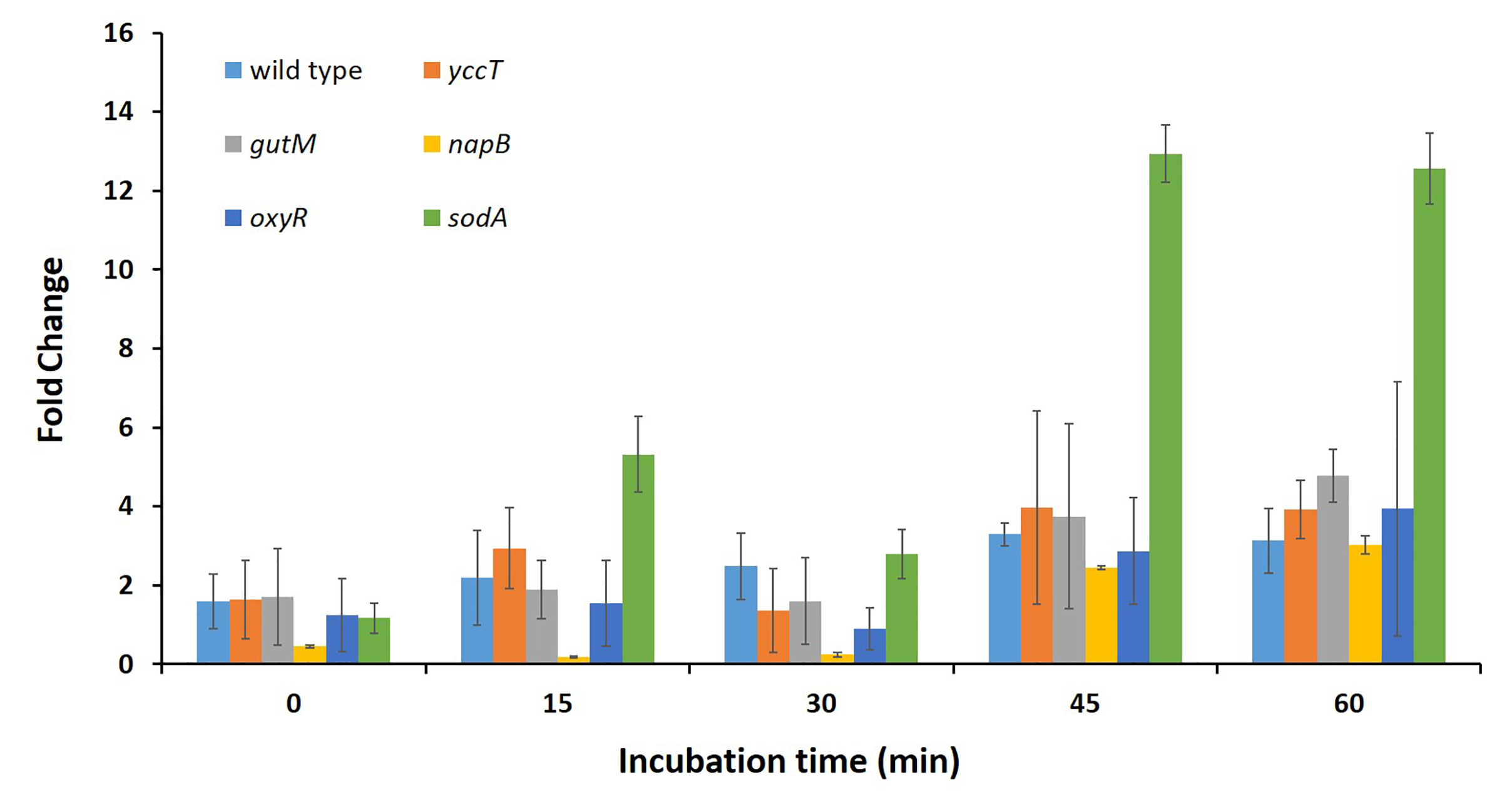

Supplement: Supplementary file 1 [file biology-08-00086-s001.zip › supplementary files/Figue S1.tif]
